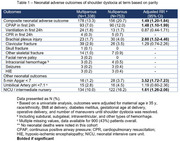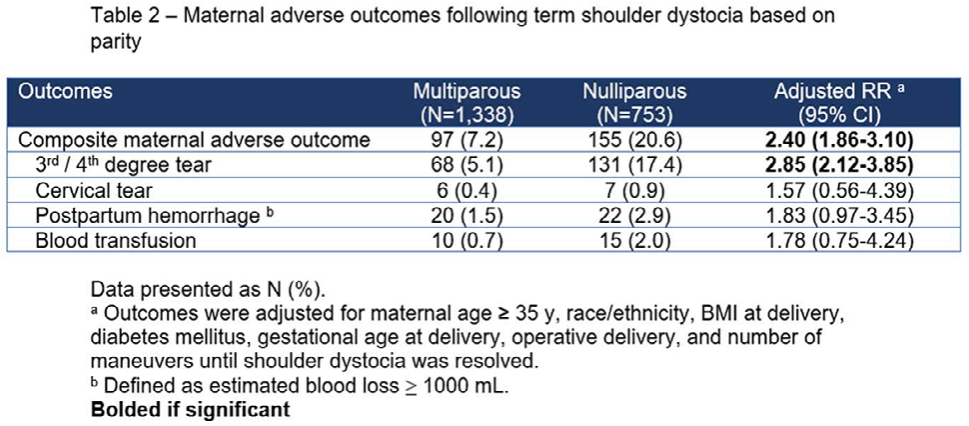# Poster Session 1: Wednesday, February 11, 2026 10:30 AM – 12:00 PM

**DOI:** 10.1002/pmf2.70173

**Published:** 2026-02-09

**10:30 AM – 12:00 PM**

## 98 | Perinatal Depression Screening Among Individuals Using WIC: A PRAMS Secondary Analysis

Anna R. Whelan; Abigail Howell

UMass Chan Medical School, Worcester, MA

**Objective**: Pregnant people who use the WIC (Women Infants and Children) program have been demonstrated to have higher rates of perinatal depression compared to those not using WIC. Individuals on WIC have higher rates of risk factors for depression including less social support, lower income, and lower education. As such, regular screening for depression is essential for people on WIC. This study aimed to compare rates of depression screening among patients using WIC to those not using WIC throughout the perinatal period

**Study Design**: This is a secondary analysis of data from the CDC's Pregnancy Risk Assessment Monitoring System (PRAMS) from 2016–2022. Participants who answered “yes” to using WIC were compared to those who answered “no” to using WIC. The primary outcome was screening for perinatal depression. Stata v18 was used for all analyses utilizing complex survey weighting.

**Results**: Of the 246,130 PRAMS participants, 88,945 (36.1%) reported using WIC. Demographic differences between groups were seen by race, ethnicity, education level (Table 1). Patients with WIC delivered earlier, were less likely to breastfeed, and had higher rates of depression and anxiety before pregnancy (Table 1). Participants using WIC were more likely to be asked about depression prior to (aOR 1.28, 95% CI 1.24–1.34) and during pregnancy (aOR 1.34, 95% CI 1.29–1.39) but less likely to be asked about depression at their postpartum visit (aOR 0.91, 95% CI 0.86–0.95) even when adjusting for non‐white race, ethnicity, marital status, education and history of depression.

**Conclusion**: ndividuals using WIC are at higher risk of perinatal depression and despite increased screening prior to conception and during pregnancy, postpartum screening falls short. While obstetrics providers should continue to work to improve screening in the postpartum period, individuals using WIC are also in contact with public health professionals including nurses. This may represent the opportunity for future development of community‐based systems for depression screening and referral.

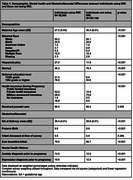

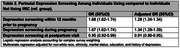

**10:30 AM – 12:00 PM**

## 99 | Does Experience Matter? Complication and Success Rates in CVS by Operator Role

Adam J. Schettler^1^; Christopher S. Ennen^1^; Ryan D. Farias^1^; Enkhbileg Dendev^2^; Ashley Lin^1^; Nina Jannatifar^3^; Bridget Donovan^1^

^1^University of Virginia School of Medicine, Charlottesville, VA; ^2^Unvieristy of Virginia School of Medicine, Charlottesville, VA; ^3^University of Virginia, Charlottesville, VA

**Objective**: Chorionic villus sampling (CVS) requires experienced proceduralists to ensure safety and success, and hands‐on training is critical to maintain this skillset across future physician generations. Limited data exist on how trainee involvement impacts CVS outcomes. The primary objective of this study was to determine whether Maternal‐Fetal Medicine (MFM) fellow performance of CVS procedures impacted success and complication rates.

**Study Design**: This was a retrospective cohort study of patients who had a transabdominal, transcervical, or combined CVS procedure at a single tertiary center between January 2016 and June 2025. The exposure was whether an MFM fellow or faculty physician performed the CVS procedure. Collected data included maternal characteristics, procedural details, complication rates, and unscheduled visits within four weeks post‐procedure.

**Results**: During the study period there were 137 total CVS procedures performed. MFM fellows performed 58% (*n* = 59) of those procedures. There was no significant difference in the maternal BMI (27.9 vs 27.0 *p* = 0.376) or placental location between the fellow and faculty groups. Procedural data is presented in Table 1. Transabdominal procedures comprised 42% of the fellow group and 32% of the faculty group (*p* = 0.214). There was no difference in the number of procedures requiring more than one pass (16.9% vs 12.8% *p* = 0.498) or the milligram quantity of villi collected (50.0 mg vs 57.1 mg *p* = 0.473) in the fellow versus faculty groups. Complications of CVS procedures by operator are presented in Table 2. Total complication rates were low and there were no statistically significant differences between the two groups.

**Conclusion**: Fellow‐performance of the CVS was not associated with increased risk of the procedure to either fetus or pregnant patient, with no difference in overall procedure success rate. This study provides reassurance to learners, teaching faculty, and patients to continue the imperative hands‐on CVS skill learning during fellowship without compromising patient safety or outcomes.

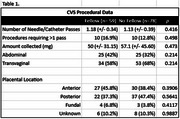

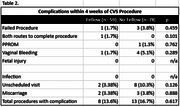

**10:30 AM – 12:00 PM**

## 100 | Time Interval From Diagnosis to Delivery in Patients With Early‐Onset Preeclampsia With Severe Features

Adam Wright, Jr.^1^; Erez Lenchner^2^; Eran Bornstein^3^; Vincenzo Berghella^4^; Moti Gulersen^5^

^1^ChristianaCare, Philadelphia, PA; ^2^New York University Rory Meyers College of Nursing, New York, NY; ^3^Northwell, New Hyde Park, NY; ^4^Thomas Jefferson University, Philadelphia, PA; ^5^Thomas Jefferson University, PHILADELPHIA, PA

**Objective**: To evaluate characteristics associated with delivery ≤7 days after diagnosis in patients with early‐onset preeclampsia with severe features (EoPE with SF) to inform timing of ACS administration.

**Study Design**: Multicenter retrospective cohort study including all patients diagnosed with EoPE with SF between 23 0/7–33 6/7 weeks from 1/1/2018–12/31/2023. Patients with postpartum diagnosis, intrauterine fetal demise, preterm prelabor rupture of membranes, missing data, or declined recommended delivery were excluded. Cases were stratified into two groups based on time interval from diagnosis to delivery: ≤7 versus >7 days. Multivariable logistic regression was performed to evaluate associations between patient characteristics, comorbidities, and clinical presentation with delivery ≤7 days. Data are presented as adjusted odds ratios (aOR) with 95% confidence intervals (CI).

**Results**: Of the 249 patients included, 180 (72.3%) delivered <7 days after diagnosis. Patient characteristics, comorbidities, and clinical presentation data compared between the two groups are displayed in Table 1. Among patients who were diagnosed with EoPE with SF based on severe hypertension alone (*n* = 176), hypertension with additional symptoms (*n* = 47), and hypertension with abnormal lab evaluation (*n* = 23), the rates of delivery <7 days were 65.9%, 89.3%, and 82.6%, respectively. Patients diagnosed based on hypertension and additional symptoms, as well as those presenting with proteinuria had higher odds of delivery <7 days (aOR 4.57, 95% CI 1.39–14.97, and 2.06, 95% CI 1.04–4.07, respectively) (Table).

**Conclusion**: Most patients diagnosed with EoPE with SF deliver <7 days after diagnosis, with diagnosis by severe hypertension with symptoms and presenting with proteinuria being associated with higher odds of delivery <7 days. The likelihood of delivery <7 days after diagnosis remained >65% regardless of the criteria for diagnosis or clinical presentation at the time of diagnosis. Therefore, ACS administration should not be delayed in this population.

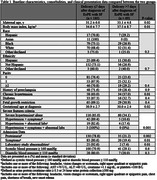

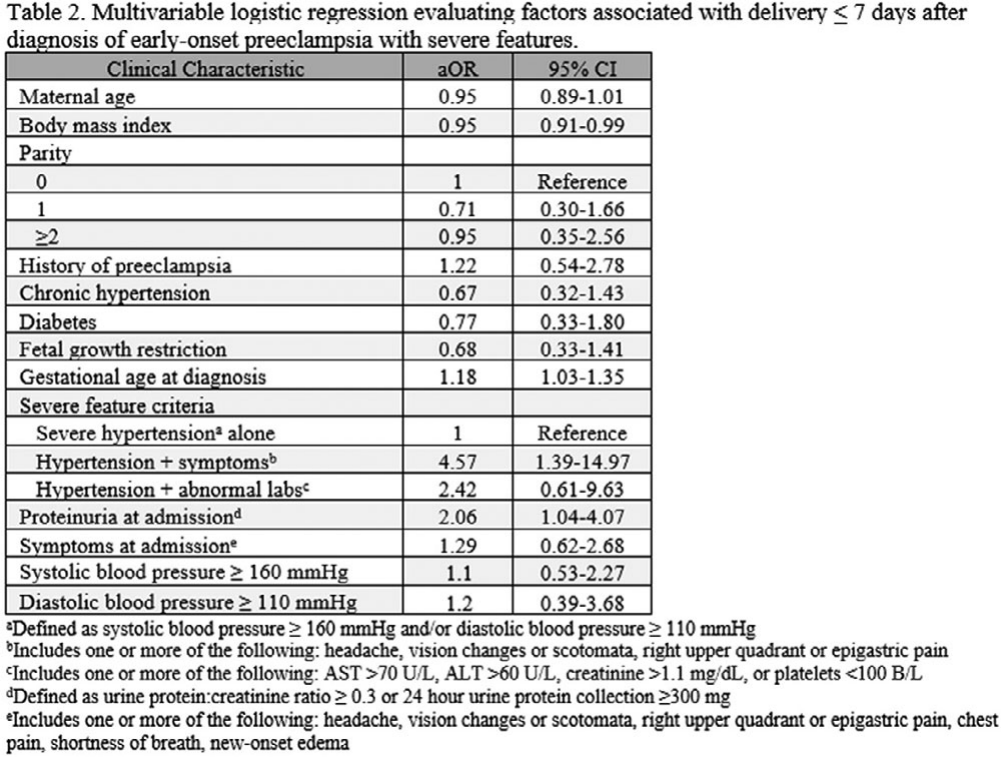

**10:30 AM – 12:00 PM**

## 101 | Prenatal and Infant Exposure to Urban Air Pollution and Respiratory Outcomes

Adi Malkoff Rabin^1^; Elkana Kohn^2^; Ariela Hazan^3^; Yael Dubowski^4^; Matitiahu Berkovitch^3^; Ori Hochwald^5^; Liron Borenstein‐Levin^5^; Tomer Ziv Baran^6^; Liisa Jantunen^7^; Miriam L Diamond^7^; Ido Solt^5^

^1^Hillel Yaffe Medical center, Binyamina, HaZafon; ^2^Shamir Medical Center, Rison Letzion, HaMerkaz; ^3^Shamir Medical Center, Rishon Lezion, HaMerkaz; ^4^Technion‐ Israel Institute of Technology, Haifa, HaZafon; ^5^Rambam Health Care Campus, Haifa, HaZafon; ^6^Tel Aviv University, Tel Aviv, HaMerkaz; ^7^University of Toronto, Toronto, ON

**Objective**: To investigate the relationship between prenatal and postnatal exposure to environmental pollutants and subsequent respiratory morbidity in toddlers, using silicone wristbands as a novel personal monitoring device. The study is based on a longitudinal maternal–fetal cohort initiated in pregnancy, providing rare insight into the continuum of environmental exposures from womb to early childhood.

**Study Design**: This is a prospective follow‐up of a cohort of pregnant women recruited at delivery in two urban regions: one adjacent to heavy industry, and the other non‐industrial, traffic‐exposed. 2–3 years later, their children (now toddlers) individual exposure to airborne pollutants was assessed using silicone wristbands worn for 7 days. Pollutant analysis, included PAHs (e.g., pyrene), phthalates (DEP, DMP), and flame retardants (OPEs). Respiratory health was evaluated through validated maternal questionnaires (ISAAC and MoBa). Pollutants were analyzed via GC‐MSD; exposure‐health associations were tested using correlation and regression models.

**Results**: 94 child‐mother dyads were analyzed. Children from the industrial area exhibited significantly higher rates of rhinitis (*p* = 0.016), general health issues (*p* = 0.033), and combined wheezing/allergic events (*p* < 0.001). Measured levels of indoor pollutants such as DEP and DMP were significantly higher in the non‐industrial urban area (*p* = 0.0003 and *p* = 0.0002, respectively). DEP levels correlated positively with respiratory symptoms (*p* = 0.029), while pyrene exposure was associated with increased use of steroids and antihistamines (*p* = 0.011).

**Conclusion**: This study highlights the feasibility and utility of non‐invasive, personalized exposure assessment in pediatric follow‐up of maternal cohorts. Despite lower pollutant levels, the industrial region was associated with greater respiratory morbidity, suggesting that **in utero and early‐life cumulative exposures**—rather than single pollutant concentrations—may drive adverse child health outcomes. These findings underscore the importance of environmental monitoring during pregnancy as part of maternal‐fetal risk stratification.

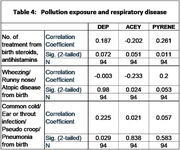

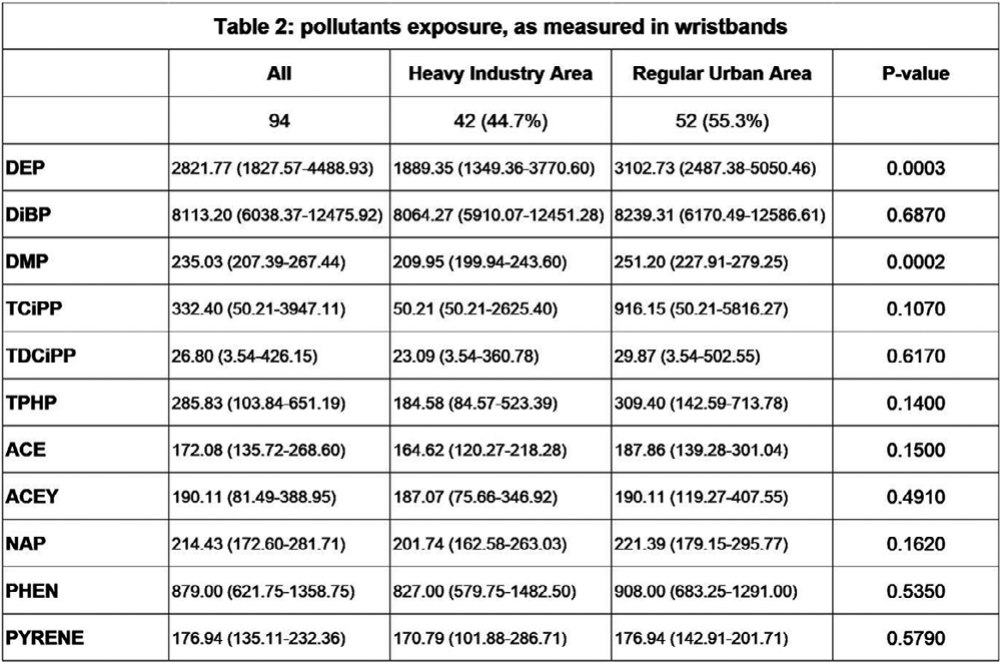

**10:30 AM – 12:00 PM**

## 102 | Evaluating Patterns and Potential Benefits of Antidepressant Initiation Before vs. at Delivery

Adina R. Kern‐Goldberger^1^; Sarah Nazeer^1^; Megan R. Ansbro^2^; Stacey Ehrenberg^3^

^1^Cleveland Clinic Lerner College of Medicine, Cleveland Clinic Lerner College of Medicine, OH; ^2^Cleveland Clinic Foundation, Cleveland Clinic Foundation, Cleveland, OH; ^3^Cleveland Clinic Foundation, Cleveland Clinic Foundation, OH

**Objective**: Perinatal depression is a common complication that often worsens following delivery. Typical management involves initiating treatment with onset of symptoms though some patients may be hesitant to take medication during pregnancy. This study investigates whether initiating treatment before delivery is associated with improved depression symptoms postpartum compared to initiating during the delivery admission.

**Study Design**: This retrospective cohort included all deliveries of patients who were not prescribed antidepressants or anxiolytics at the initial prenatal visit but later initiated treatment during pregnancy or at delivery, and who delivered in a single health system from 1/1/2021–6/30/2025. The primary exposure was psychotropic medication prescription at the time of the last prenatal visit prior to delivery and the comparison group initiated medication at postpartum discharge. Patient characteristics and clinical outcomes were compared in bivariate analysis. Multivariable logistic regression with propensity scoring evaluated odds of elevated initial postnatal EPDS score >10 as well as secondary outcomes (42‐day readmission, postpartum visits within 8 weeks).

**Results**: Among 5198 patients, 78.2% (*N* = 4065) initiated medication prior to delivery. Patients who started medication at delivery admission were younger, had lower BMI, and initiated prenatal care later (Table 1). They also had higher rates of preterm birth, longer postpartum stay, and higher incidence of SMM and ICU admission. Elevated EPDS scores were observed in 10.5% (*N* = 426) of patients who started treatment pre‐delivery, compared to 7.1% (*N* = 81) at delivery (*p* < 0.01). Multivariable analysis demonstrated aOR of 0.97 for elevated EPDS scores with initiation of treatment at delivery (95% CI 0.79–1.19) and 1.21 (95% CI 1.06–1.39) for short‐term postpartum follow‐up.

**Conclusion**: These data did not demonstrate measurable differences in depression symptoms based on timing of medication initiation, though initiation at delivery was associated with deliveries complicated by SMM and with higher odds of short term postpartum follow up.

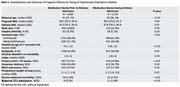

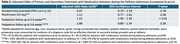

**10:30 AM – 12:00 PM**

## 103 | Assessing Maternal Outcomes in Patients Taking GLP‐1 Receptor Agonists Prior to Pregnancy

Adina R. Kern‐Goldberger^1^; Rachel Cevigney^2^; Sarah Nazeer^3^; Stacey Ehrenberg^4^; Jodi Meridieth^5^; Cara D. Dolin^6^

^1^Cleveland Clinic Lerner College of Medicine, Cleveland, OH; ^2^Cleveland Clinic Foundation, Cleveland, OH; ^3^Cleveland Clinic Lerner College of Medicine, Cleveland Clinic Lerner College of Medicine, OH; ^4^Cleveland Clinic Foundation, Cleveland Clinic Foundation, OH; ^5^Cleveland Clinic, Cleveland, OH; ^6^Cleveland Clinic, Clevelend, OH

**Objective**: Maternal comorbidities such as obesity and diabetes increase risk for severe maternal morbidity (SMM). Glucagon‐like peptide‐1 (GLP‐1) receptor agonists are a promising treatment strategy to optimize metabolic health outside of pregnancy. It is unknown whether pre‐pregnancy GLP‐1 agonist use can optimize maternal pregnancy outcomes. This study evaluates risk of SMM in pregnant patients with obesity by exposure to GLP‐1s.

**Study Design**: This retrospective cohort study included patients with pregravid BMI > 30 who delivered at >20 weeks in a multihospital health system from 1/1/2021–6/30/2025. Patient medications in the EHR were evaluated for 6 months prior to pregnancy to ascertain GLP‐1 prescriptions. Maternal demographics and clinical characteristics were extracted from the EHR and compared in bivariate analyses. Multivariable logistic regression was used to assess odds of the primary outcome, SMM without transfusion as defined by the CDC, as well as secondary outcomes (gestational hypertension/mild preeclampsia, severe preeclampsia, ICU admission, 42‐day readmission).

**Results**: 8669 patients were included: 3.4% were prescribed pre‐pregnancy GLP‐1 agonists (*N* = 291). The GLP‐1 exposed group were more likely to be older, have higher BMI, and higher obstetric comorbidity index (Table 1). There were no differences in race, ethnicity, or insurance type. Multivariable logistic regression demonstrated aOR of 1.53 (95% CO 0.88–2.66) for SMM with pre‐pregnancy GLP‐1 exposure (Table 2). No differences in secondary outcomes were observed other than for readmission, which was significantly associated with prior GLP‐1 use.

**Conclusion**: Pre‐pregnancy GLP‐1 use was not associated with increased risk for SMM or most other adverse maternal outcomes despite higher baseline risk. Optimizing maternal health with novel medications like GLP‐1 agonists is an emerging potential strategy to reduce maternal morbidity.

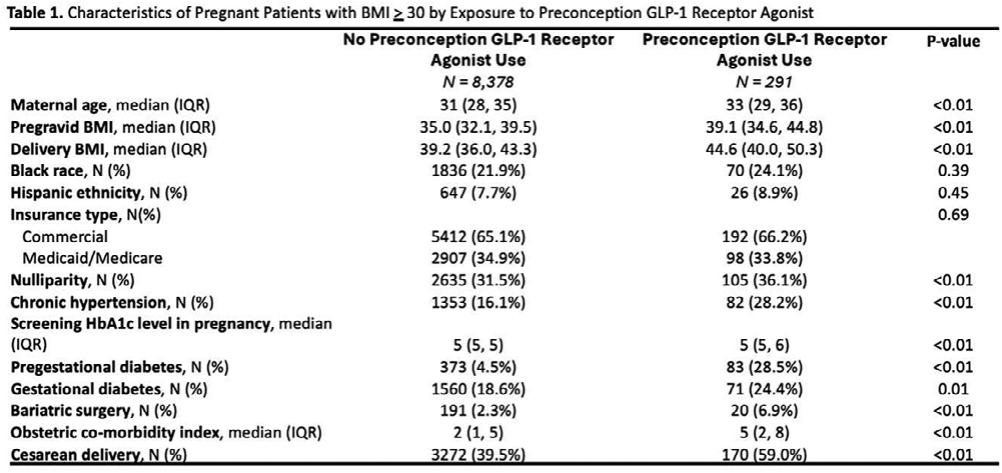

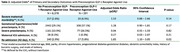

**10:30 AM – 12:00 PM**

## 104 | TTTS Phenotypes: How Concurrent TAPS and sFGR Affect Laser Surgery Outcomes

Ahmet A. Baschat^1^; Jena L. Miller^2^; Michelle Kush^3^; Mara Rosner^2^

^1^Johns Hopkins University, Johns Hopkins University, MD; ^2^Johns Hopkins Center for Fetal Therapy, Johns Hopkins University/Baltimore, MD; ^3^Johns Hopkins Center for Fetal Therapy, Johns Hopkins University/BAltimore, MD

**Objective**: Monochorionic twins are at risk for twin‐to‐twin transfusion syndrome (TTTS), anemia‐polycythemia sequence (TAPS), and selective growth restriction (sFGR). This study quantifies concurrence of these complications with TTTS and evaluates their impact on laser survival.

**Study Design**: This single‐center study categorized TTTS cases into 4 phenotypes: (1) isolated TTTS (maximum recipient and donor amniotic fluid pockets >8 cm/<2 cm, respectively), (2) TTTS+TAPS (middle cerebral artery peak systolic velocity discordance >0.5 multiples of median), (3) TTTS+TAPS+sFGR (fetal weight discordance ≥25%), and (4) TTTS + sFGR. We determined proportional distribution of these phenotypes and associations with Quintero stage, critical Doppler abnormalities [umbilical artery absent end‐diastolic velocity (UA‐AEDV), ductus venosus absent/reversed a‐wave (DV‐RAV)], and 48‐hour dual laser survival.

**Results**: In 500 patients, 273 (54.6%) had isolated TTTS, 53 (10.6%) coexisting TAPS, 30 (6%) TAPS+sFGR and 144 (18.8%) coexisting sFGR (Figure 1A). Concurrent TAPS or sFGR affected distribution of Quintero stage. Stage III was highest with coexisting sFGR and stage IV with coexisting TAPS (*p* < 0.001, Figure 1B). Critical Doppler abnormalities differed significantly: DV RAV occurred in 27 (32.5%) recipients with concurrent TAPS (Chi square *p* = 0.004) and UA AEDV in 67 (38.5%) of donors with coexisting sFGR (Figure 1C). 48‐hour dual survival of >90% in patients with isolated TTTS or TAPS decreased to 81% with concurrent sFGR and to 73% when TTTS, TAPS and sFGR co‐occurred (Figure 1A, *p* = 0.003). Recipient demise was unaffected by phenotype but donor demise was determined by sFGR and UA‐AEDV (*p* < 0.001).

**Conclusion**: TTTS often presents with concurrent complications which progressively worsen laser survival. Coexisting sFGR compromises donor survival, while TAPS primarily affects recipients, each producing distinct hemodynamic profiles and disease severity patterns. These findings challenge treating TTTS as a uniform entity supporting comprehensive assessment followed by phenotype‐specific risk‐stratification and counselling.

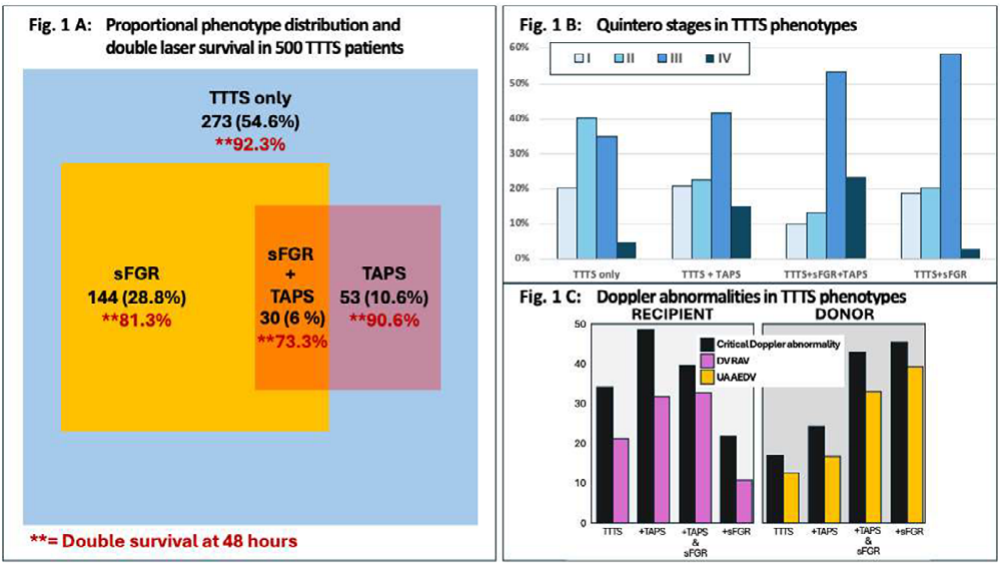

**10:30 AM – 12:00 PM**

## 105 | Impact of Vaginal Bleeding on Transfusion Needs in Placenta Accreta Spectrum

Alesha M. White^1^; Jessica E. Pruszynski^2^; Anne M. Ambia^2^; Catherine Y. Spong^2^; Christina L. Herrera^2^

^1^University of Texas Southwestern Medical Center, Grand Prairie, TX; ^2^University of Texas Southwestern Medical Center, Dallas, TX

**Objective**: Vaginal bleeding is commonly a reason for early delivery for patients with prenatal suspicion of placenta accreta spectrum (PAS). The impact of vaginal bleeding on transfusion needs is unknown in PAS cases. The objective of this study is to understand the impact of vaginal bleeding on transfusion requirements in cases of prenatally diagnosed PAS.

**Study Design**: Retrospective cohort study that included all cases of prenatally suspected PAS diagnosed between 2019 and 2025 and who delivered at a single institution with a multidisciplinary PAS team. Maternal demographics, delivery characteristics and transfusion trends were compared between patients delivered for vaginal bleeding to those who did not experience vaginal bleeding.

**Results**: Of 169 prenatally suspected PAS patients, 34 (20%) were delivered because of vaginal bleeding. Patients with vaginal bleeding were more likely to deliver at earlier gestational ages (33.5 (31–35) vs 36 (36–36), *p* < 0.001) but there were no other differences noted in terms of maternal demographics. Degree of PAS suspected based on pathology, rate of hysterectomy, median estimated blood loss, rate of transfusion and massive transfusion did not differ. Patients with vaginal bleeding were more likely to receive a platelet transfusion (18% vs 4%, *p* = 0.016). Of those transfused, total number of all products transfused and timing of transfusion did not differ (Table 2).

**Conclusion**: Despite early delivery due to vaginal bleeding, compared to those without bleeding, transfusion needs only differed by rate and quantity of platelets transfused. This may reflect a higher frequency of consumptive coagulopathy. Platelet transfusion should be readily available in patients with vaginal bleeding in the setting of PAS.

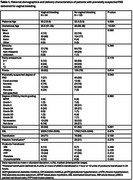

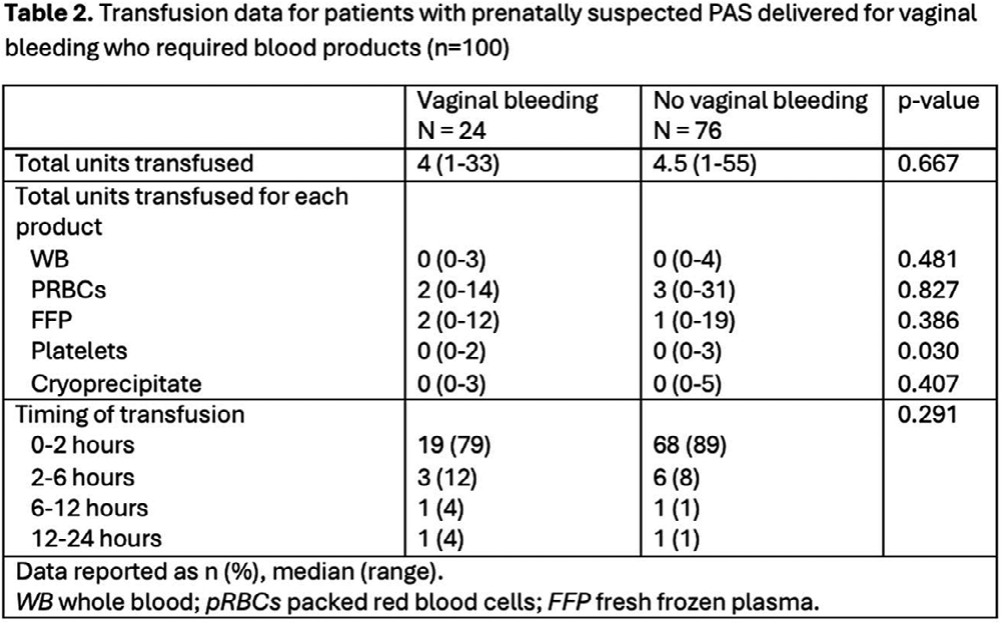

**10:30 AM – 12:00 PM**

## 106 | Association of Inpatient Surveillance With Perinatal Outcomes in Gestational Diabetes Requiring Insulin

Alesha M. White^1^; Ashlyn Lafferty^2^; Sryea Gandra^2^; So‐Van Maxwell^2^; Jessica E. Pruszynski^2^; Elaine L. Duryea^2^; Catherine Y. Spong^2^

^1^University of Texas Southwestern Medical Center, Grand Prairie, TX; ^2^University of Texas Southwestern Medical Center, Dallas, TX

**Objective**: Although data is limited, antenatal testing is recommended for patients with gestational diabetes mellitus (GDM) managed with insulin starting at 32 weeks. The purpose of this study was to explore the relationship between inpatient surveillance with use of antenatal testing and perinatal outcomes in a GDM population.

**Study Design**: Retrospective cohort study of patients with GDM requiring insulin who delivered between 2022 and 2024 at a large county hospital. At our institution, outpatient antenatal testing in the form of non‐stress tests (NSTs) is not performed. Hospital admission is recommended at 34 weeks for all patients on insulin to enhance glycemic control and fetal monitoring. Patients who decline admission are followed outpatient. Perinatal outcomes for patients who did and did not undergo inpatient surveillance were compared. A neonatal composite outcome including rates of large for gestational age (LGA), hypoglycemia, admission to the neonatal intensive care unit, neonatal death and stillbirth was compared. Patients who were admitted for less than 1 week, who did not receive prenatal care with our institution, or who delivered prior to 34 weeks were excluded.

**Results**: 530 patients with GDM requiring insulin were included. 155 (29%) were admitted for inpatient surveillance. Despite similar A1c's, patients who were admitted had improved glycemic control (Table 1). Although gestational age was significantly different, the difference was not clinically relevant. There was no difference in the neonatal composite outcome between the two cohorts. Neonates born to patients who were admitted for inpatient surveillance had lower birthweights (3182 g (2920–3536) vs 3308 g (2980–3724), *p* = 0.004) and lower rates of LGA (13% vs 22%, *p* = 0.022) (Table 2). Fetal weight remained significant even after adjusting for gestational age (*p* = 0.017).

**Conclusion**: Inpatient surveillance was associated with improved glycemic control and reduced rates of LGA infants, however the majority of patients were unable to accept admission. Future studies should focus on increasing accessibility to similar programs.

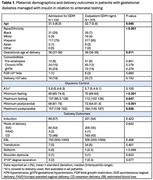

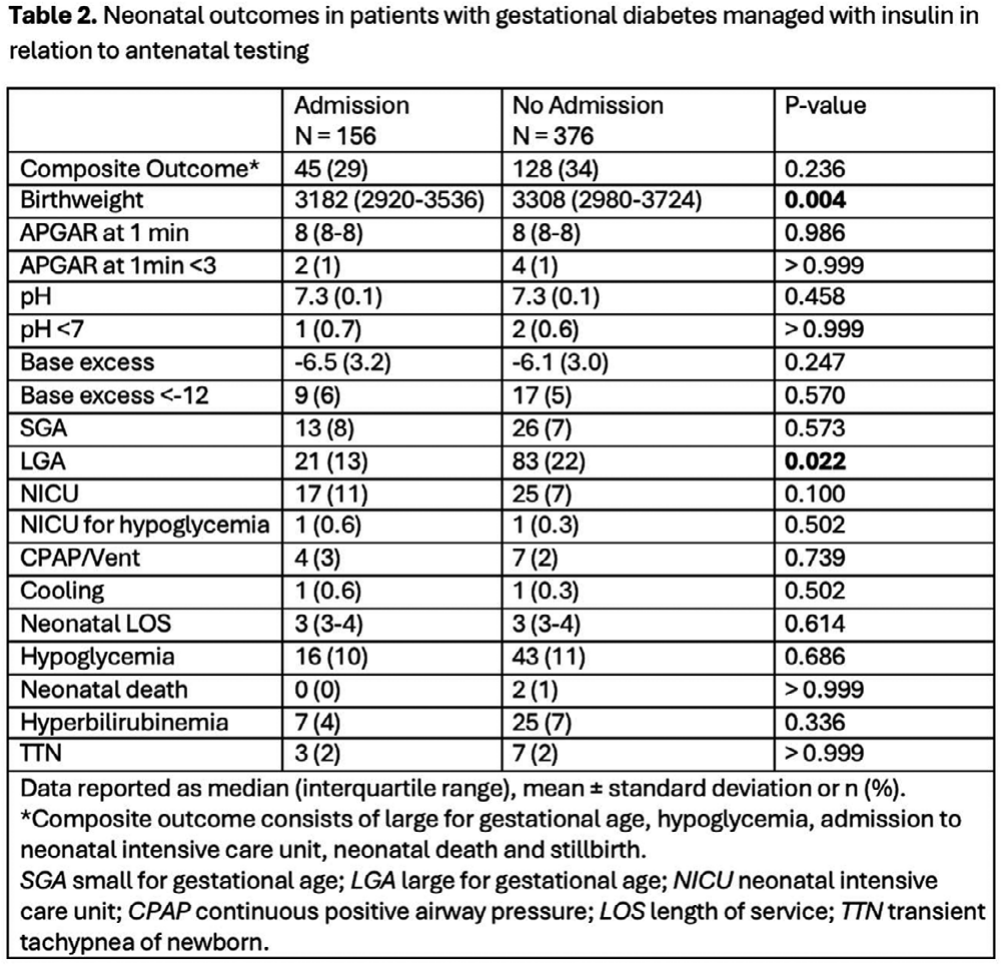

**10:30 AM – 12:00 PM**

## 107 | Under the Microscope: A Closer Look at Placental Histological Findings in Early‐Onset Fetal Growth Restriction

Alex Dakin^1^; Ronan Daly^1^; Denisa Asandei^2^; Zara Molphy^3^; Patrick Dicker^4^; Emma Doyle^5^; Michael Boyle^6^; Etaoin Kent^5^; Fergal D. Malone^7^

^1^Department of Obstetrics and Gynaecology, Royal College of Surgeons in Ireland, Dublin, Dublin; ^2^Department of Obstetrics and Gynaecology, Royal College of Surgeons in Ireland, Royal College of Surgeons in Ireland, Dublin; ^3^Royal College of Surgeons in Ireland, Dublin, Dublin; ^4^Department of Obstetrics and Gynaecology & Department of Public Health and Epidemiology, Royal College of Surgeons in Ireland, Royal College of Surgeons in Ireland, Dublin; ^5^Rotunda Hospital, Dublin, Dublin; ^6^Rotunda Hospital, Rotunda Hospital, Dublin; ^7^Department of Obstetrics and Gynaecology, Royal College of Surgeons in Ireland, Dublin, Ireland., Dublin, Dublin

**Objective**: Early‐onset fetal growth restriction (EOGFR) is associated with histopathological abnormalities on both the maternal and fetal side of the placenta. Our aim was to evaluate placental histological features and correlate with perinatal outcomes.

**Study Design**: A single‐centre retrospective cohort study was performed on EOFGR diagnosed between 18–32 weeks’ gestation at the largest European maternity hospital from July 2016–June 2022. Delphi consensus criteria were used for definition, and genetic, structural or infectious causes were excluded. Placental histological features were compared to antenatal characteristics and pregnancy outcomes using Chi‐square and Wilcoxon rank test. Stepwise multivariate logistic regression was used to determine the most important independent predictors of maternal vascular malperfusion (MVM).

**Results**: There were 341 cases of uteroplacental EOFGR, with a 95% livebirth rate (325/341). 66% (225/341) of placentas were sent for histological analysis. MVM was the most common associated abnormality (60%, 136/225) with fetal vascular malperfusion (FVM) present in 30% (67/225). 14% (31/225) of cases had both MVM & FVM present.

Table 1 correlates MVM and FVM with pregnancy outcomes. Pre‐eclampsia (PET) and umbilical artery Doppler (UAD) abnormality remain strong predictors of MVM after adjustment for the effects of each other (PET, aOR 5.5 (95% CI, 2.2–13.6; *p* < 0.001); Abnormal UAD, aOR 3.0 (95% CI, 3.0–5.9; p 0.001)).

Placental hypoplasia was present in 71% (160/225) and this was significantly associated with MVM (*p* = 0.005) (Figure 1). Mean fetoplacental ratio was 5.7 (SD 1.65) and this was not associated with either MVM (*p* = 0.118) or FVM (*p* = 0.836).

**Conclusion**: MVM is the most frequent placental finding in EOFGR and is more closely associated with preterm delivery, PET and adverse outcome, whereas FVM showed no correlation with these factors, suggesting two distinct pathological processes underlying EOFGR. Given their difference in outcomes, placental histological assessment is critical following any pregnancy affected by EOFGR, to inform monitoring and care of future pregnancies.

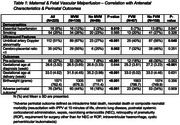

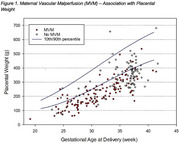

**10:30 AM – 12:00 PM**

## 108 | Impact of Latency Antibiotic Regimens on Neonatal Infectious Morbidity

Alexander Boscia^1^; Madalyn Lutz^2^; Danielle Browning^2^; Christina J. Megli^3^

^1^UPMC Magee‐Womens Hospital, Pittsburgh, PA; ^2^UPMC Children's Hospital of Pittsburgh, UPMC Children's Hospital of Pittsburgh, PA; ^3^University of Pittsburgh Magee Women's Hospital, University of Pittsburgh Magee Women's Hospital, PA

**Objective**: Maternal antibiotic (abx) exposure has been associated with increased rates of necrotizing enterocolitis (NEC) and late‐onset sepsis (LOS). Decreased levels of mammary immunoglobulin A (mIgA) have been implicated in the development of NEC and LOS in murine models. We previously demonstrated an association between decreased mIgA and increased maternal abx exposure in human MBM samples. In the current study, we sought to demonstrate a clinical association between maternal abx exposure and neonatal infectious morbidity in patients with preterm premature rupture of membranes (PPROM). We hypothesized that nontraditional latency regimens would be associated with greater neonatal infectious morbidity.

**Study Design**: We performed a retrospective cohort study of an institutional database comprising PPROM admissions between 2015 and 2020. We included patients with expectantly‐managed PPROM between 24 and 32 weeks without evidence of preterm labor. Patients were grouped into cohorts based on latency regimen: trad (ampicillin/amoxicillin), and alt ‐ cefazolin/cephalexin (ceph) or gentamicin/clindamycin (gent/clinda). The primary outcome was a composite of LOS, NEC, and death. Results were stratified by potential confounders and subgroup analyses performed to evaluate associations between individual groups.

**Results**: Of 227 infants included in the analysis, 212 and 39 were exposed to trad and alt regimens, respectively. The primary outcome occurred in 21% (trad) versus 38% (alt) of patients (*p* = .04). After stratification, the association remained significant for multiple categories (Table 1) including MBM exposure. Ceph regimens demonstrated increased odds of LOS compared to trad regimens (8% vs 29%, *p* < 0.01) that remained significant in similar categories after stratification (Table 2).

**Conclusion**: Non‐traditional latency regimens are associated with increased risks of a composite of neonatal infectious morbidity. Cephalosporin‐based regimens specifically were associated with increased rates of LOS. These effects persisted in infants fed MBM. Larger prospective studies are needed to validate this association.

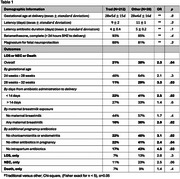

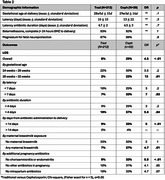

**10:30 AM – 12:00 PM**

## 109 | PREDICT‐GDM: Prospective Evaluation of a Risk‐Based Model for Diabetes Intervention & Care Tailoring

Alexandra Berezowsky^1^; Nir Melamed^2^; Jon F. Barrett^3^; Joel G. Ray^1^; Erin Harrington^1^; Sarah D. McDonald^3^; Michael P. Geary^4^; Beth Murray‐Davis^3^; Vahinie Narang^5^; Howard Berger^1^

^1^University of Toronto, Toronto, ON; ^2^Sunnybrook Health Sciences Center, Toronto, ON; ^3^McMaster University, Hamilton, ON; ^4^Rotunda Hospital Dublin & Royal College of Surgeons in Ireland, Rotunda Hospital Dublin & Royal College of Surgeons in Ireland, Dublin; ^5^St. Michael's Hospital, Toronto, ON

**Objective**: To prospectively assess the validity, safety, feasibility, and clinical impact of a novel risk prediction model to identify women with gestational diabetes mellitus (GDM) at low risk of lifestyle modification (LM) failure who can be redirected to a less resource‐intensive pathway.

**Study Design**: We conducted a single center, parallel‐group, proof‐of‐concept RCT (Aug 2022–Jul 2024), enrolling individuals with singleton GDM pregnancies achieving glycemic targets with LM alone at baseline. Participants were randomized to routine care (RC) in the Diabetes in pregnancy clinic or to a modified care (MC) pathway using a previously developed and validated prediction model. Individuals identified at low risk of LM failure were discharged to routine obstetric care with care provider blinded remote glucose monitoring. Those identified at high risk of LM failure remained in the DIP clinic (Figure 1). The primary outcome was validation of model performance. Exploratory outcomes included glycemic control, maternal/neonatal outcomes, patient satisfaction and resource utilization.

**Results**: Of 582 individuals with GDM, 211 were eligible; 150 consented and were randomized. In the intervention arm, 18 (24%) were identified as low‐risk and 57 as high‐risk. All low‐risk participants maintained glycemic control on LM, yielding NPV of 100%, specificity of 30.2%, and AUC of 0.732 (95% CI: 0.649–0.814). Patient satisfaction (*n* = 67) was high (mean 3.86/5), with no significant group differences. There were lower admission days (2.8 ± 0.86 vs 3.6 ± 1.3, *p* = 0.006) in the low‐risk group and a trend toward lower caesarean rates (6.7% vs 35.8%, *p* = 0.051). Compared with low‐risk controls, low‐risk individuals in the MC pathway differed in the rate of postprandial dinner glucose above target  , post‐partum hemorrhage and mean specialist visits (Table 1).

**Conclusion**: This easily implementable tool accurately identifies nearly a quarter of low‐risk GDM patients who can safely be managed in a lower‐intensity care model thus reducing resource use without compromising outcomes.

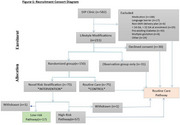

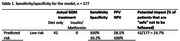

**10:30 AM – 12:00 PM**

## 110 | Predictors of Risk‐Reducing Interventions for High BMI Patients Undergoing Cesarean Delivery

Alexandra C. Gallagher^1^; Elizabeth B. Sherwin^2^; Stephanie A. Leonard^3^; Dipro Chakraborty^1^; Natasha Harrison^1^; Selin Kandemir^4^; Pervez Sultan^5^; Ilona T. Goldfarb^2^

^1^Stanford University, Palo Alto, CA; ^2^Stanford University, Stanford, CA; ^3^Stanford University, Stanford University, CA; ^4^Vanderbilt University, Nashville, TN; ^5^Stanford University Healthcare, Palo Alto, CA

**Objective**: Cesarean delivery poses higher perioperative risks for patients with high BMI. Clinical guidelines suggest several risk‐reducing interventions: prenatal obstetric anesthesia consultation, adjusted prophylactic antibiotic dosing (3 g cefazolin), and pharmacologic venous thromboembolism (VTE) prophylaxis. Our study compared characteristics of patients who received these interventions to those who did not to identify predictors.

**Study Design**: We analyzed electronic health record data in a standardized common data model from a single, high volume academic institution between 2015 to 2023. We included patients with BMI ≥40 and/or maternal weight ≥120 kilograms (kg) delivered by cesarean. As eligibility for 3 g cefazolin is determined by maternal weight, we compared patients with maternal weight ≥120 kg who received 3 g cefazolin to those who did not. As eligibility for prenatal obstetric anesthesia consultation and pharmacologic VTE prophylaxis is determined by high BMI, we compared those with BMI ≥40 who received either or both to those who received neither. To compare groups, Wilcoxon rank sum test was used for continuous variables and Fisher's exact test for categorical variables.

**Results**: 691 patients with BMI ≥40 and 282 with weight ≥120 kg met inclusion criteria. 200 eligible patients (70.9%) received 3 g cefazolin, while 82 (29.1%) did not. Those who received 3 g cefazolin had higher BMI, weight in kg and rate of preexisting diabetes. 491 eligible patients (71%) received either prenatal obstetric anesthesia consultation, pharmacologic VTE prophylaxis or both, while 200 (29%) received neither. Those who received at least one intervention had higher BMI, weight in kg, rates of preexisting diabetes, and obstructive sleep apnea compared to those who did not. No significant racial or insurance differences were observed.

**Conclusion**: Patients with higher BMI and weight were more likely to receive risk‐reducing interventions than their eligible counterparts while many eligible patients received no intervention. Further research across institutions should aim to understand these differences.

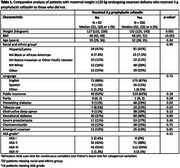

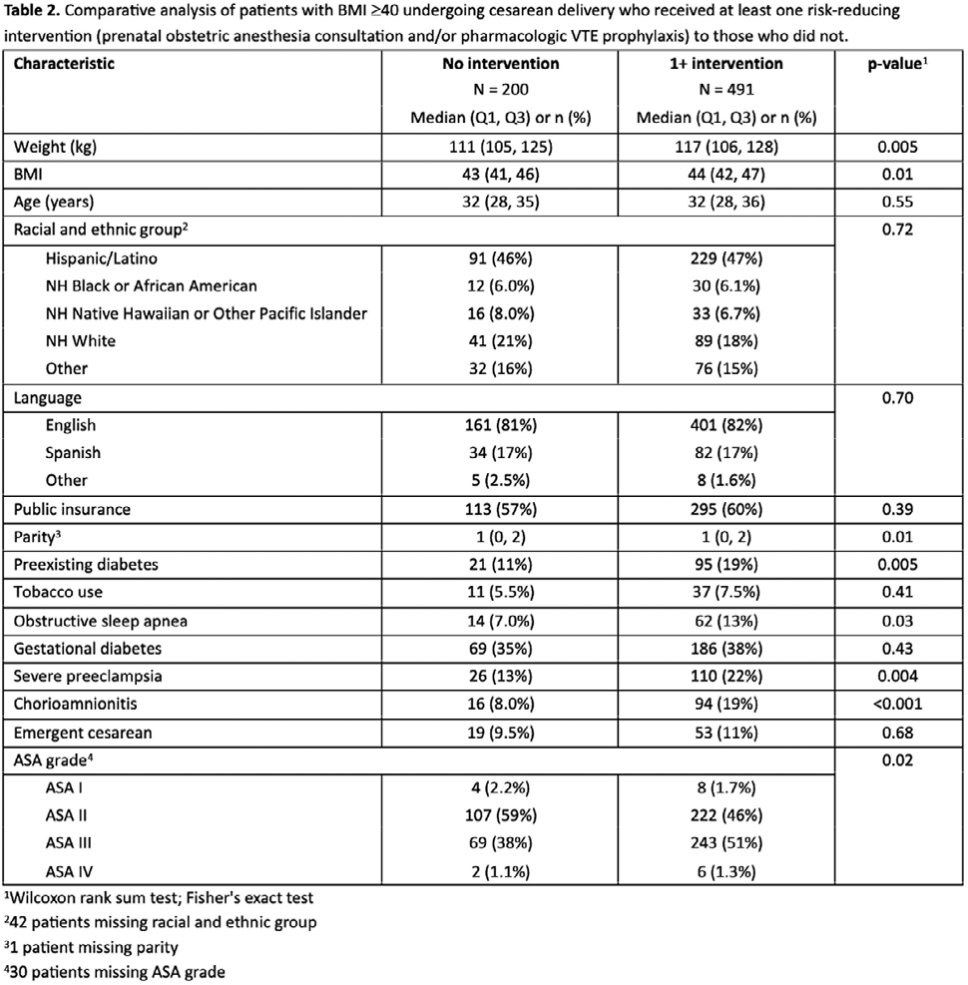

**10:30 AM – 12:00 PM**

## 111 | Scaling the Collaborative Care Model in Obstetrics: Multilevel Determinants to Guide Implementation

Alexandra Turco^1^; Andrea Chu^2^; Emily Feinberg^3^; LG Ward^4^; Emily S. Miller^5^

^1^Warren Alpert Medical School of Brown University, Providence, RI; ^2^Hassenfeld Institute for Child Health Innovation at Brown University, Providence, RI; ^3^Hassenfeld Child Health Innovation Institute at Brown University, Providence, RI; ^4^Center for Behavioral & Preventative Medicine, The Miriam Hospital, Providence, RI; ^5^Women & Infants Hospital of Rhode Island / Warren Alpert Medical School of Brown University, Providence, RI

**Objective**: The Collaborative Care Model (CCM) is an evidence‐based approach to care that improves health outcomes in primary care settings by integrating mental health into routine medical care. While recent adaptations have tailored the CCM for use in obstetric settings to address perinatal mental health (PMH) concerns, real‐world implementation remains limited. Bridging this gap between research and practice requires an understanding of contextual factors influencing adoption and sustainment. Our objective was to identify key factors modifying the implementation of the perinatal Collaborative Care Model (pCCM).

**Study Design**: We conducted a qualitative implementation study guided by the Exploration, Preparation, Implementation, Sustainment (EPIS) framework to evaluate facilitators and barriers of pCCM implementation. Using purposive sampling, we recruited clinical and administrative key informants from six obstetric clinics and one birthing hospital. Semi‐structured interviews were designed to elicit determinants at multiple contextual levels. Data were analyzed using the Rapid Qualitative Analysis process. Emergent themes were mapped to the EPIS domains–Inner Context, Outer Context, Bridging Factors, and Innovation Characteristics.

**Results**: A total of 20 individuals were interviewed prior to thematic saturation. Determinants influencing adoption and sustainability of the pCCM mapped across EPIS domains are summarized in Table 1. While several cross‐cutting barriers emerged, participants also identified multiple facilitators that support integration of the CCM into obstetric settings. The need for site‐level adaptation was an underlying theme, underscoring the opportunity to tailor implementation strategies to local contexts.

**Conclusion**: The CCM is well suited to obstetric care but requires context‐specific adaptation to support adoption and sustainability. Implementation strategies must address multilevel barriers and leverage existing facilitators to bridge the gap between research and practice.

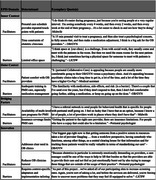

**10:30 AM – 12:00 PM**

## 112 | Feasibility and Outcomes of Early Postpartum 2‐Hour Glucose Tolerance Testing for Patients With Gestational Diabetes

Cheryl Dinglas^1^; Alexis Gerk^2^; Kristen Lentz^2^; Apoorva Rangan^2^

^1^Mount Sinai South Nassau, Rockville Centre, NY; ^2^Mount Sinai South Nassau, Oceanside, NY

**Objective**: To evaluate the acceptance rates and outcomes of the early 2‐hour 75 g oral glucose tolerance test (OGTT) during delivery hospitalization.

**Study Design**: This retrospective cohort analysis included all patients with gestational diabetes (GDM) who delivered between October 2024 and May 2025 after implementation of the inpatient OGTT at our institution. Exclusion criteria included diagnosis of pre‐existing T1DM and T2DM or inability to tolerate the OGTT. The 75 g‐OGTT was performed for patients on postpartum days 2–4 prior to hospital discharge. Electronic medical records were reviewed to collect demographic information, clinical characteristics, results of the OGTT, and delivery outcomes.

**Results**: Of the 1332 patients who delivered during this timeframe, 163 patients had GDM. One hundred and forty‐seven patients were offered screening (90.1%), 99 of whom performed the 2h OGTT (67.3% acceptance rate). Rates of acceptance were higher in the GDMA2 group compared to GDMA1 (55.6% vs. 44.4%; *p* = 0.036). There were no differences in age, body mass index, parity, race, insurance type, gestational age at delivery, mode of delivery, or delivery complications (shoulder dystocia, hemorrhage) or NICU admission between the 2 groups. Of the 99 patients who performed the 2h OGTT, 21 patients with GDMA2 (21.2%) and 13 patients with GDMA1 (13.1%) met criteria for impaired glucose tolerance (IGT), and 2 patients with GDMA2 (2%) and 2 patients with GDMA1 (2%) met criteria for diabetes mellitus (DM).

**Conclusion**: Acceptance of the inpatient GTT is higher amongst patients with GDMA2 compared to GDMA1 yet both groups were found to have IGT and DM. All patients with GDM regardless of treatment type should be encouraged to complete this testing.

**10:30 AM – 12:00 PM**

## 113 | Characterizing Shock Index in Pregnant Patients Undergoing Massive Transfusion for Obstetric Hemorrhage

Alicia Davidson; Hadley Ross; Terri‐Jeanne Liu; Donese Henneke; Elisabeth Halm; Emma Tao; Victoria Epstein; Erika Brigmon; Donald Jenkins; Kayla E. Ireland

UT Health San Antonio, San Antonio, TX

**Objective**: Whole blood (WB) is widely used in trauma resuscitation but remains underutilized in obstetrics. In 2019, our institution incorporated low‐titer O+ whole blood (LTOWB) into its massive transfusion protocol (MTP) for obstetric hemorrhage. Shock index (SI), calculated as heart rate divided by systolic blood pressure, has been proposed as an early marker of hemodynamic compromise, with values >0.9 associated with increased mortality. We aimed to evaluate the physiologic response to LTOWB by assessing time to SI normalization in obstetric patients undergoing massive transfusion.

**Study Design**: We conducted a retrospective observational study of obstetric patients (*n* = 47) who received LTOWB at a single tertiary care center from 2020 to 2024. We assessed time from transfusion initiation to SI normalization (SI < 0.9), along with the proportion of patients normalizing SI at 5, 15, 30, 60, 90, and 120 minutes. Additional analyses included time to peak SI and associations between time to normalization and estimated blood loss, total transfusion volume, hemorrhage etiology, and mode of delivery.

**Results**: Median SI at transfusion initiation was 1.0 (IQR 0.78–1.16), peaking at 1.18 (1.03–1.36) before improving. SI normalization was achieved by 60 minutes (median SI 0.85 [0.72–1.0]) and sustained through 120 minutes (0.80 [0.68–0.94]). Among 27 patients with abnormal SI at initiation, median time to normalization was 76 minutes, with nearly two‐thirds normalizing within 30 minutes. Neither estimated blood loss (HR 1.00, 95% CI 1.00–1.00, *p* > 0.9) nor total transfusion volume (HR 1.01, 95% CI 0.98–1.04, *p* = 0.7) was significantly associated with time to normalization. ICU admission occurred in 63.8%, hysterectomy in 76.6%, and DIC in 14.9%.

**Conclusion**: Among obstetric patients undergoing massive transfusion, LTOWB was associated with rapid improvement in SI, with most patients normalizing within 30 minutes. These findings support the physiologic benefit of early transfusion in obstetric hemorrhage and highlight the need to identify additional predictors of hemodynamic recovery.

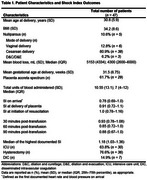

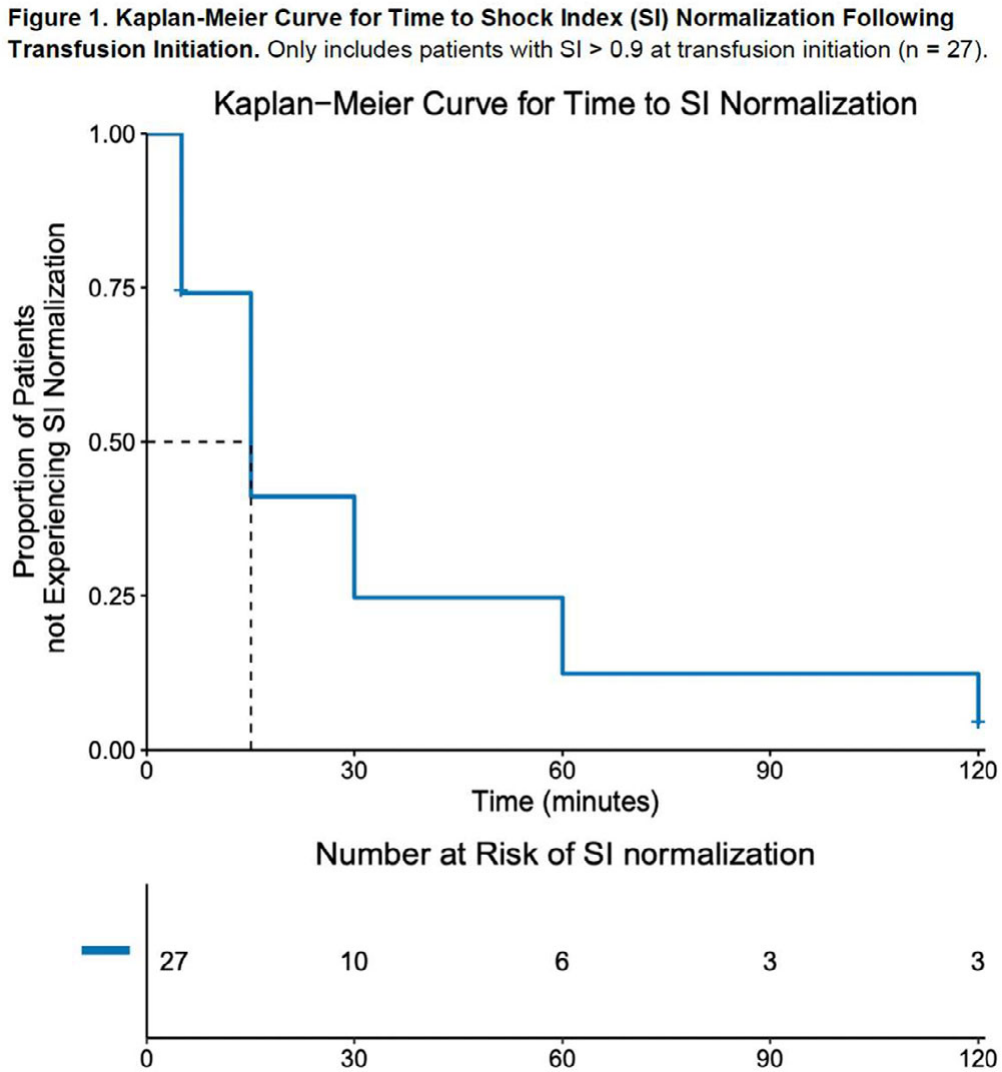

**10:30 AM – 12:00 PM**

## 114 | In‐Utero Ventriculosubgaleal Shunt Placement in Fetal Lamb Hydrocephalus: Phase II Safety and Outcomes

Shohra Qaderi^1^; Weston Northam^2^; Soner Duru^3^; Eyal Krispin^4^; Nikan Zargarzadeh^5^; Ali Javinani^6^; Jose L. Peiro^7^; Hamidreza Forourtan^8^; Ehsan Rojhani^5^; Enaja Sambatur^9^; Faezeh Aghajani^5^; Erdem Fadiloglu^10^; Claudio V. Schenone^4^; Akihiro Hasegawa^6^; Lucas Peiro^11^; Ellen Grant^6^; Ramen H. Chmait^12^; Benjamin C. Warf^6^; Kjersti M. Aagaard^13^; Alireza A. Shamshirsaz^4^

^1^Baylor College of Medicine, Houston, TX; ^2^Boston Children's Hospital, boston, MA; ^3^Cincinnati, Cincinnati, OH; ^4^Boston Children's Hospital, Harvard Medical School, Boston, MA; ^5^Boston Children's Hospital, Boston, MA; ^6^BCH, Boston, MA; ^7^New York University Langone Health Fetal Research Lab, New York, NY; ^8^Laparoscopy Research Center, Shiraz, Fars; ^9^Southeast Health Medical Center, Southeast Health Medical Center, AL; ^10^Hacettepe Uni, Ankara, Ankara; ^11^Cincinnati, Cincinnati, OH; ^12^UC, LA, MA; ^13^HCA, Houston, TX

**Objective**: We evaluated the safety and initial efficacy of intrauterine placement of a ventriculosubgaleal Shunt (VSGS) using a fetal lamb obstructive hydrocephalus model.

**Study Design**: Hydrocephalus was induced in fetuses at 91–93 days of gestation by injecting 1–2 mL of BioGlueⓇ into the cisterna magna under ultrasound (US) guidance. Two weeks later, after confirming the hydrocephalus with US, a paramedian laparotomy and hysterotomy were performed under general anesthesia. Next, the fetal head was exposed through the hysterotomy and the distal end of the VSGS was placed in a lateral ventricle, while the proximal end was tunneled subcutaneously (0.5‐0.8 mm) and fixed with interrupted silk stitches using a transuterine or hysterotomy‐assisted approach. Finally, ewes were euthanized late gestation and fetuses delivered by hysterotomy. Lambs underwent gross inspection and postmortem saline dye testing to assess shunt patency.

**Results**: We included eleven fetuses. Nine underwent successful transuterine hydrocephalus induction, while two required a hysterotomy‐assisted approach due to difficulty accessing the cisterna magna. At two weeks, one ewe containing two fetuses required euthanasia due to intrauterine demise and severe uterine adhesions. Therefore, a total of nine fetuses underwent hysterotomy‐assisted VSGS placement (Table 1). The duration from scalp incision to closure was 5–10 minutes. At cesarean delivery, two out of seven fetuses were demised (22% post‐VSGS loss). Skin was fully healed, and the shunt remained in position in all seven survivors. Two of seven shunts (28%) were confirmed patent during post‐mortem examination (Figure 1), while the others were occluded by fibrin or debris. No cases exhibited sub scalp cerebrospinal fluid (CSF) accumulation or bulging.

**Conclusion**: VSGS placement is technically feasible, with short operative times and high rates of technical success. Post‐procedure loss rates were lower than in the first study phase (22% vs 40%). Shunt patency was achieved in only 28% of successfully inserted catheters.

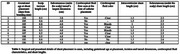

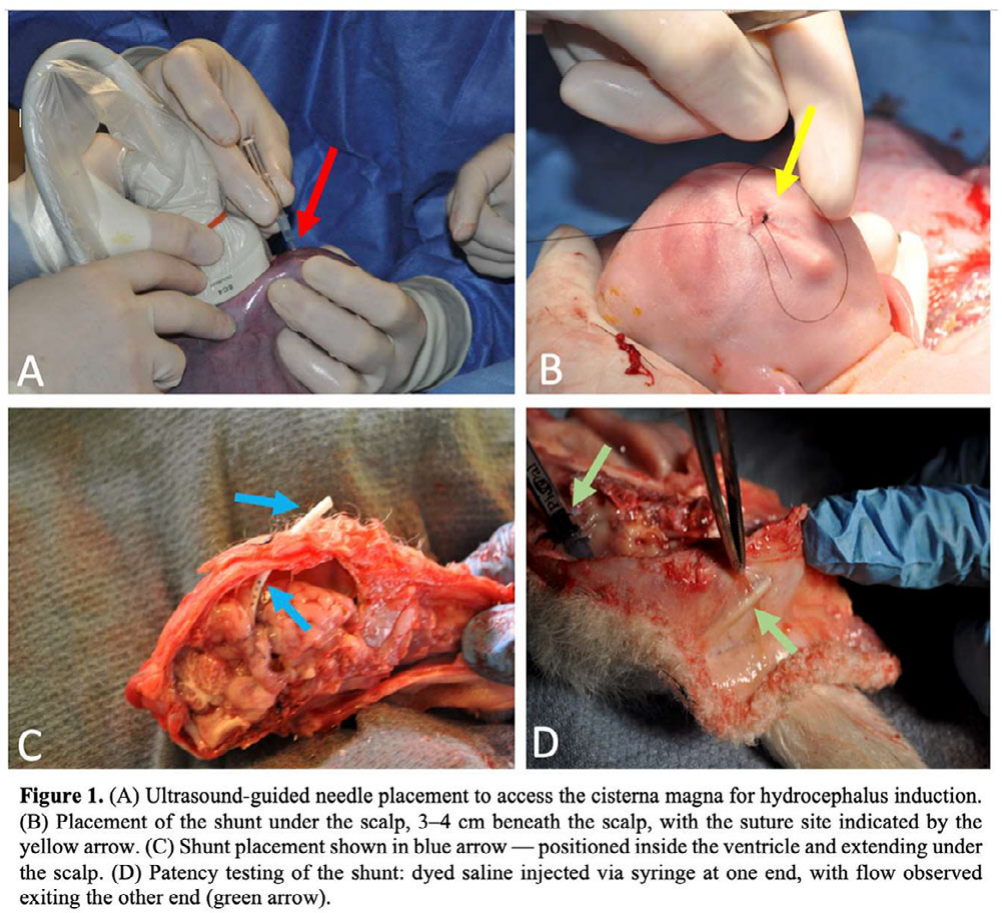

**10:30 AM – 12:00 PM**

## 115 | Factors Associated With Postpartum Depression Symptoms Following Antepartum Hospitalization

Alison N. Goulding^1^; Daniel Palacios^1^; Sukru Aras^1^; Hu Chen^1^; Sasidhar Pasupuleti^1^; Marika Toscano^2^; Nicole Cirino^1^; Israel C. Christie^1^; Zhandong Liu^1^; Emily S. Miller^3^; Terri L. Fletcher^1^

^1^Baylor College of Medicine, Houston, TX; ^2^Johns Hopkins University, Baltimore, MD; ^3^Women & Infants Hospital of Rhode Island / Warren Alpert Medical School of Brown University, Providence, RI

**Objective**: Hospitalized antepartum patients are at increased risk for postpartum depression (PPD), which can contribute to adverse maternal and infant health outcomes. Developing approaches to identify those at highest risk of PPD would enable timely and targeted intervention. We aimed to identify factors associated with PPD symptoms in hospitalized antepartum patients and to assess their predictive utility.

**Study Design**: This retrospective cohort study included pregnant individuals hospitalized due to medical or obstetric complications in a referral center, 2012–2025. Data were extracted from the electronic health record, including demographics, medical history, and postpartum Edinburgh Postnatal Depression Scale (EPDS) score collected within 8 weeks of delivery. Primary outcome was EPDS score ≥ 10, indicating PPD symptoms. We performed bivariate analyses and multivariate logistic regression modeling. Potential predictors were identified based on a priori clinical relevance; the least absolute shrinkage and selection operator (LASSO) was used for final variable selection. Receiver operating characteristic (ROC) curve analyses assessed model discriminatory ability.

**Results**: Among 4161 included individuals, 1291 (31%) reported PPD symptoms. Cohort characteristics stratified by PPD symptoms are presented in Table 1. Multivariate logistic regression modeling showed that certain demographic and clinical factors were associated with PPD symptoms (Figure 1). Discriminatory ability of the multivariate model was modest, with area under the curve (AUC) of 0.70 (95% CI 0.68–0.71).

**Conclusion**: One in three individuals hospitalized during pregnancy reported PPD symptoms. While certain demographic and clinical factors are associated with PPD symptoms, their overall ability to accurately identify those at increased risk is limited, reducing their utility in guiding targeted interventions. These findings support that postpartum mental health services are needed for individuals who experience antenatal hospitalization and highlight the need for universal mental health service provision for this high‐risk population.

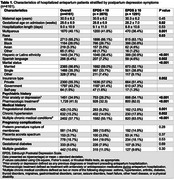

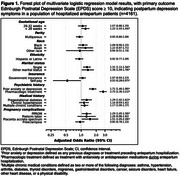

**10:30 AM – 12:00 PM**

## 116 | Poor Sleep Quality in Pregnancy is Associated Adverse Mental Health Outcomes Through 12 Months Postpartum

Alison M. Stuebe, N/A^1^; Danielle Roubinov^1^; Brenda Pearson^1^; Samantha Meltzer‐Brody^1^; Karen Grewen^2^

^1^University of North Carolina School of Medicine, Chapel Hill, NC; ^2^University of North Carolia School of Medicine, Chapel Hill, NC

**Objective**: Prenatal insomnia affects >40% of women in the 3rd trimester. We sought to quantify the association of insomnia symptoms with postpartum mental health and stress.

**Study Design**: We analyzed data from the Mood, Mother, and Infant Study cohort study. Psychiatric history was assessed at enrollment in the 3^rd^ trimester via Structured Clinical Interview, with oversampling of women with a history or current diagnosis of major depressive disorder or anxiety disorders. Sleep quality was assessed with the Pittsburgh Sleep Quality Index at baseline and at 2, 6 and 12 months. Poor sleep was defined by threshold score of >5. Participants completed the Edinburgh Postnatal Depression Scale (EPDS) monthly and the Everyday Stress Index and Parenting Stress Index at 2, 6 and 12 months postpartum. We used repeated measures analysis to measure the association of these outcomes with Poor Sleep in Pregnancy (PSP), adjusting for psychiatric history. We used linear regression to compare 12‐month postpartum measures among women whose poor sleep resolved postpartum, defined as all postpartum PSQI ≤5, compared with those with persistent poor sleep, defined as at least one postpartum PSQI >5, adjusting for psychiatric history. *p* values < 0.05 were considered statistically significant.

**Results**: Among 221 participants, 106 (48.0%) had PSP. PSP was associated with worse sleep quality postpartum (*p* < .0001), higher EPDS scores (*p* < .0001), higher parenting stress (*p* < .0001), and higher everyday stress (*p* = .0003). At 12 months, compared with persistent poor sleep, resolution of poor sleep following delivery was associated with improved EPDS scores (5.7 vs. 2.6, *p* < .01) . Differences in parenting stress (72.8 vs. 67.7, *p* = .16) and everyday stress (13.4 vs 10.2, *p* = .12) among those with resolved versus persistent poor sleep were not statistically significant.

**Conclusion**: PSP is associated with poor postpartum sleep, perinatal depression symptoms, and stress. Resolution of poor sleep is associated with improved mood one year after birth. Treatment of PSP is a promising strategy to improve maternal mental health.

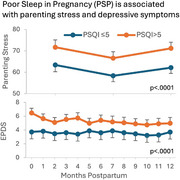

**10:30 AM – 12:00 PM**

## 117 | Large‐Scale Proteomics in Early Pregnancy and Timing of Diagnosis of Hypertensive Disorders of Pregnancy

Alisse Hauspurg^1^; Xiaoning Huang^2^; Philip Greenland^2^; Victoria L. Pemberton^3^; Jasmina Varagic^3^; C. Noel Bairey Merz^4^; George R. Saade^5^; Lisa D. Levine^6^; Lynn M. Yee^7^; Judith H. Chung^8^; Lauren H. Theilen^9^; Brian Kleiboeker^10^; Uma M. Reddy^11^; Janet Catov^10^; Sadiya S. Khan^2^; William A. Grobman^12^

^1^Women & Infants Hospital of Rhode Island / Warren Alpert Medical School of Brown University, Providence, RI; ^2^Northwestern University, Chicago, IL; ^3^National Heart, Lung, and Blood Institute, NIH, Bethesda, MD; ^4^Cedars‐Sinai Medical Center, Los Angeles, CA; ^5^Department of Obstetrics and Gynecology, Eastern Virginia Medical School at Old Dominion University, Norfolk, VA; ^6^Perelman School of Medicine, University of Pennsylvania, Philadelphia, PA; ^7^Northwestern University Feinberg School of Medicine, Chicago, IL; ^8^University of California Irvine Medical Center, Irvine, CA; ^9^University of Utah, Salt Lake City, UT; ^10^University of Pittsburgh, Pittsburgh, PA; ^11^Columbia University Irving Medical Center, New York, NY; ^12^Brown University, providence, RI

**Objective**: Hypertensive disorders of pregnancy (HDP) may first develop antepartum, during labor, or postpartum, and whether these presentations represent a similar disease process remains uncertain. We utilized untargeted large‐scale proteomics to identify pathways associated with HDP based on their timing of onset.

**Study Design**: We used data and first‐trimester plasma samples from the NuMoM2b‐Heart Health Study, a multi‐site prospective cohort that followed nulliparous pregnant individuals from the first trimester through pregnancy. We compared differential protein expression based on timing of onset of HDP (categorized as antepartum, intrapartum, postpartum) versus controls (referent) in a nested case‐control design using SomaScan 7K platform. Associations of individual proteins with timing of onset of HDP, adjusted for co‐variates (age, self‐reported race as a social construct, insurance, body mass index, diabetes, fetal sex), were assessed using logistic regression q value‐based false discovery rates. Pathway enrichment and differential expression analysis was conducted using Ingenuity Pathway Analysis.

**Results**: Of 1628 individuals included, 678 had a HDP, of which 67.3% initially manifested antepartum (AP), 29.5% intrapartum (IP), and 1.4% postpartum (PP). After adjusting for co‐variates, compared to controls, 698 proteins, 39 proteins, and 144 proteins were differentially expressed in those with HDP according to AP, IP, PP onset, respectively. There was little overlap in individual protein expression based on timing of HDP onset, with pathway enrichment analysis suggesting distinct processes. Ingenuity graphical summary noted downregulation of angiogenesis in AP HDP, downregulation of insulin‐like growth factor regulation in IP HDP, and upregulation of fibroblast proliferation in PP HDP.

**Conclusion**: There are differences in protein expression in early pregnancy compared to controls based on whether HDP first manifests in the AP, IP or PP period. This raises the possibility that there may be distinct mechanistic phenotypes that could uniquely inform diagnostic and therapeutic targets for HDP.

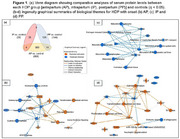

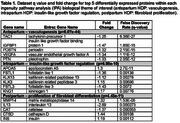

**10:30 AM – 12:00 PM**

## 118 | Tocolytic Use and Altitude Exposure in Air Transport for Threatened Preterm Labor

Alixandria F. Pfeiffer^1^; Karissa Lonon^2^; Katie McBee^2^; Allison Moreno^3^; Hadley Ross^4^; Patrick S. Ramsey^5^; John J. Byrne^6^

^1^University of Texas Health San Antonio, University of Texas health San Antonio, TX; ^2^University of Texas Health San Antonio, San Antonio, TX; ^3^University Health System, San Antonio, TX; ^4^UT Health San Antonio, San Antonio, TX; ^5^Department of Obstetrics and Gynecology University of Texas Health Science Center in San Antonio, San Antonio, TX; ^6^University of Texas Health Science Center in San Antonio, San Antonio, TX

**Objective**: To evaluate whether pre‐transport tocolytic use is associated with controlling uterine contractions during air transport among pregnant patients with threatened preterm labor (PTL), and secondarily to assess whether altitude exposure affects contraction patterns during transport.

**Study Design**: We conducted a retrospective cohort study of pregnant patients ≥20 and <37 weeks’ gestation transported via air to a tertiary maternal care center from 2020–2025. Altitude exposure was categorized as High (>3000 ft), Low (≤3000 ft), or Unknown. The primary outcome was uterine activity pattern during transport (increased, unchanged, or decreased contractions per 10 minutes), focusing on identifying contraction control or reduction. Multivariable logistic regression assessed associations between pre‐transport tocolytic use and contraction control, adjusting for gestational age, flight type (helicopter vs fixed‐wing), insurance, and rurality. Secondary analyses evaluated whether altitude exposure was associated with contraction changes in the full cohort, helicopter‐only subgroup, and cases with known altitude.

**Results**: Among 185 air‐transported patients, 113 were transferred for PTL (60 without tocolytics, 53 with). In adjusted analyses, pre‐transport tocolytic use was not significantly associated with contraction control during transport (OR 0.74, 95% CI 0.36–1.71, *p* = 0.53). Altitude documentation was available for 21 helicopter transports (Low = 17, High = 4). In the helicopter‐only subgroup, altitude category was not significantly associated with contraction changes (Low vs High OR 1.14, *p* = 0.91). In the full cohort model, altitude was also not significantly associated with uterine activity (Low vs High OR 1.11, *p* = 0.93; Unknown vs High OR 0.53, *p* = 0.55).

**Conclusion**: Pre‐transport tocolytic use was not associated with improved control of uterine contractions during air transport for PTL. Altitude exposure, including in helicopter‐only and altitude‐known subgroups, was not associated with contraction changes.

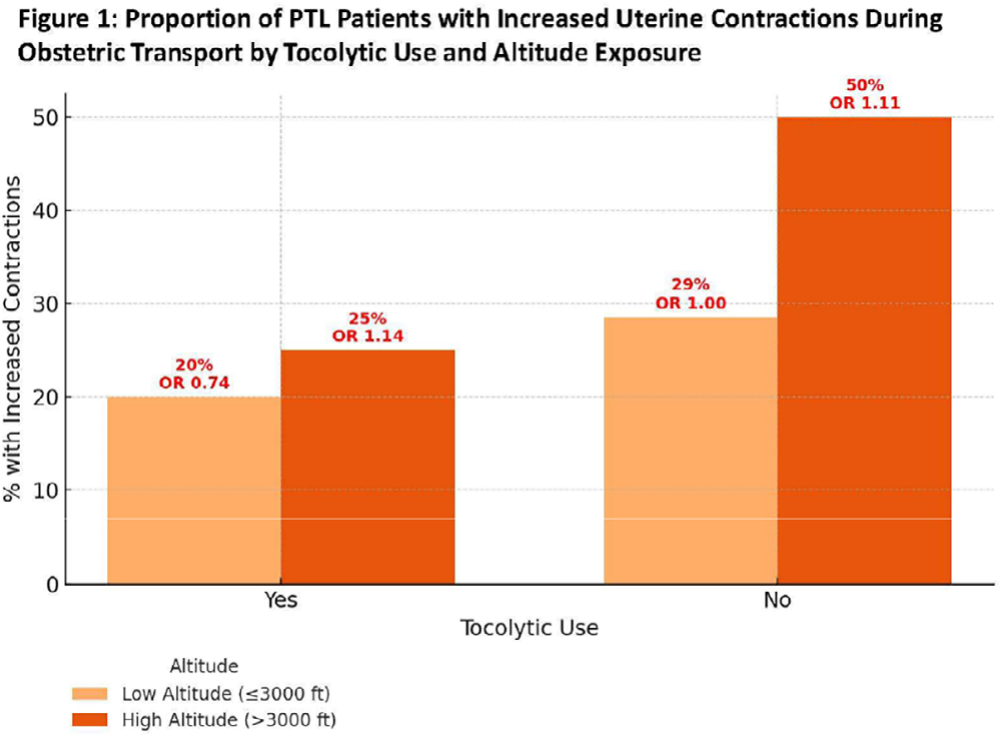

**10:30 AM – 12:00 PM**

## 119 | Risk Factors for Uterine Incision Extension During Emergency Cesarean Deliveries

Alla Saban^1^; Noa Leybovitz Haleluya^2^; Reli Hershkovitz^3^; Adi Y. Y.Weintraub^4^; Tamar Eshkoli^3^

^1^Soroka Medical Center, beer sheva, HaDarom; ^2^Soroka Medical Center, meitar, HaDarom; ^3^Soroka Medical Center, Ben Gurion University of the Negev, beer sheba, HaDarom; ^4^Soroka University Medical Center, Faculty of Health Sciences, Ben‐Gurion University of the Negev, beer sheba, HaDarom

**Objective**: Uterine incision extension during cesarean delivery (CD) can increase maternal morbidity and operative time. This study aimed to identify independent maternal, obstetric, and surgical risk factors associated with uterine incision extension in emergency CDs.

**Study Design**: We conducted a case‐control study of singleton emergency CDs between Feb 2020 and Jan 2022 at a tertiary center. Exclusion criteria included non‐transverse uterine incisions, missing surgical reports, uterine rupture or dehiscence, Müllerian anomalies, placenta accreta, or urgent hysterectomy. Cases were defined as CDs with uterine incision extension, and controls as those without. Clinical, obstetric, and intraoperative variables were compared using bivariate analysis followed by multivariable logistic regression to identify independent risk factors.

**Results**: Of 2676 emergency CDs, 270 (10.1%) were complicated by uterine incision extension. On bivariate analysis (Table 1), factors significantly associated with an increased risk of extensions included younger maternal age (*p* = 0.029), lower gravidity and parity (*p* < 0.001 and *p* = 0.014, respectively), oligohydramnios (*p* = 0.004), higher neonatal birthweight (*p*< 0.001), regional anesthesia (*p* = 0.016), and bladder flap dissection (*p* = 0.001). factors associated with reduced risk included preterm delivery< 37 weeks (*p*< 0.001), hypertensive disorders of pregnancy (*p* = 0.014) and polyhydramnios (*p* = 0.019).

In multivariable logistic regression analysis (Table 2), oligohydramnios (adjusted OR 2.20; 95% CI 1.36–3.55, *p* = 0.001) and bladder flap dissection (adjusted OR 1.54; 95% CI 1.15–2.09, *p* = 0.003) were independently associated with increased odds of uterine incision extension. In contrast, preterm delivery was protective (adjusted OR 0.31; 95% CI 0.18–0.56, *p* < 0.001).

**Conclusion**: Uterine incision extension in emergency CDs is independently associated with bladder flap dissection and oligohydramnios, whereas preterm delivery appears to be protective. Preoperative recognition of these risk factors may assist in optimizing surgical planning and minimizing maternal morbidity.

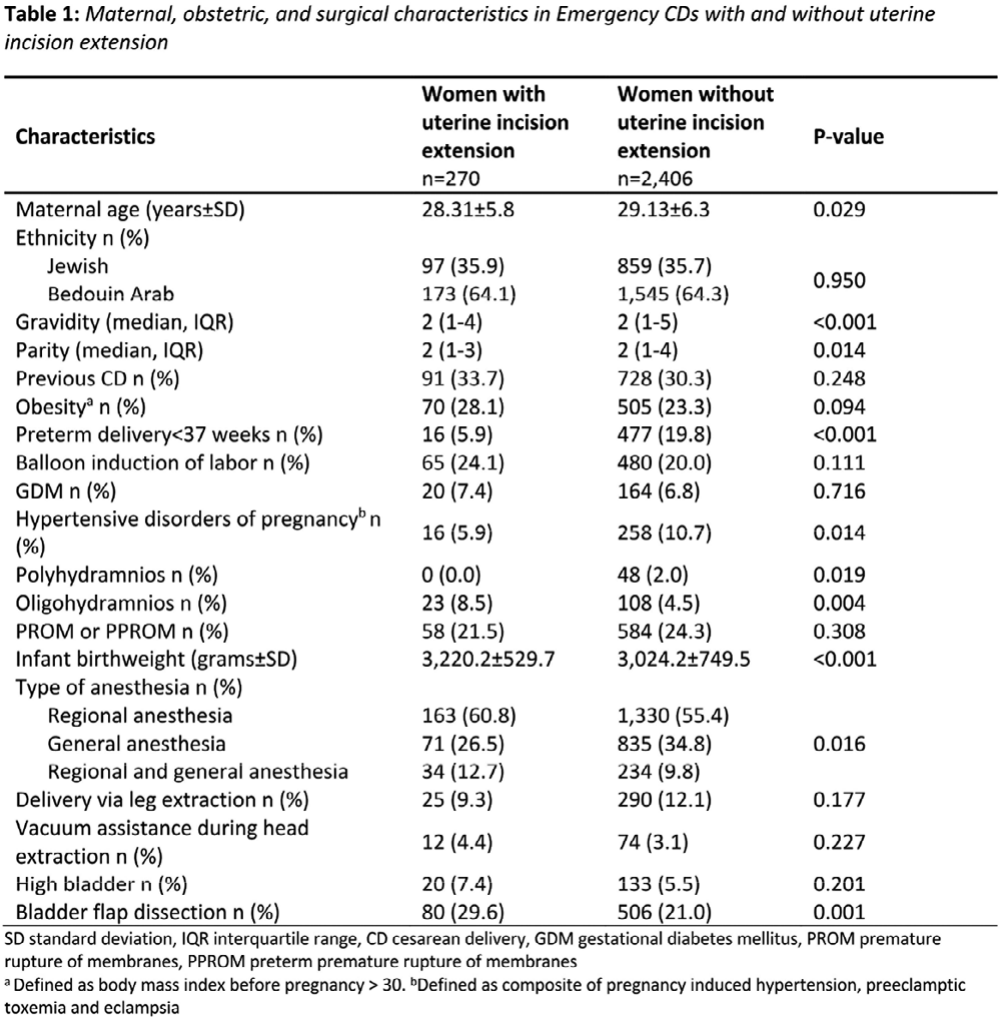

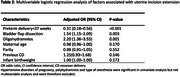

**10:30 AM – 12:00 PM**

## 120 | Infant Birthweight Does Not Differ Following Exposure to Maternal Syphilis After 20 Weeks Gestation

Allison Kurzeja^1^; Kristen A. Warncke^1^; Abigail B. Clark^2^; Shreya Battu^1^; Heath Yancey^1^; Jessica E. Pruszynski^1^; Emily H. Adhikari^1^

^1^University of Texas Southwestern Medical Center, Dallas, TX; ^2^University of Texas Southwestern Medical Center, University of Texas Southwestern Medical Center, TX

**Objective**: Emerging data suggest fetal growth may be impacted by exposure to syphilis after 20 weeks of gestation. We compared birthweight among infants of gravidas with syphilis diagnosed after 20 weeks and unexposed infants.

**Study Design**: This is a retrospective matched cohort study of gravidas with syphilis diagnosed after 20 weeks’ gestation at a large public hospital. We excluded patients with substance use and syphilis treatment prior to pregnancy, unless early syphilis was diagnosed. Cases were matched to controls (1:2) without syphilis based on maternal age ±5 y, race, ethnicity, gestational and pregestational diabetes, chronic or gestational hypertension, and preeclampsia. Comparisons were made using mixed effects models to account for matching.

**Results**: From January 2010 through June 2025, 173 cases of maternal syphilis were diagnosed at a median gestational age of 29 weeks (IQR 23–33) and matched to 346 uninfected controls. Among those with syphilis, 77 (45%) had early‐stage syphilis (primary, secondary, or early latent) and 96 (55%) latent, late or unknown duration. Of 144 (83%) who received intramuscular benzathine penicillin G before delivery, the median gestational age at first dose was 29 weeks (IQR 24.3–32.4). Median gestational age at birth was 39 weeks for both groups (IQR 38–40, *p* = 0.72). Median infant birthweight (g) did not differ between syphilis exposed and control infants (3200 [IQR 2778–3486] vs 3248 [IQR 2912–3600], mean difference ‐60.5 g [95% CI,‐175.0, 54.0]). Infants in the syphilis cohort had lower odds of small for gestational age (SGA) diagnosis compared to controls (13[8%] vs. 50[14%], respectively; OR 0.47 [95% CI, 0.24‐0.89]). Stillbirth occurred in 4(2%) with syphilis compared to 1(0.3%) in controls (*p* = 0.06).

**Conclusion**: Birthweight did not differ among infants exposed to maternal syphilis diagnosed after 20 weeks’ gestation compared to unexposed infants after matching for maternal medical comorbidities and excluding substance misuse.

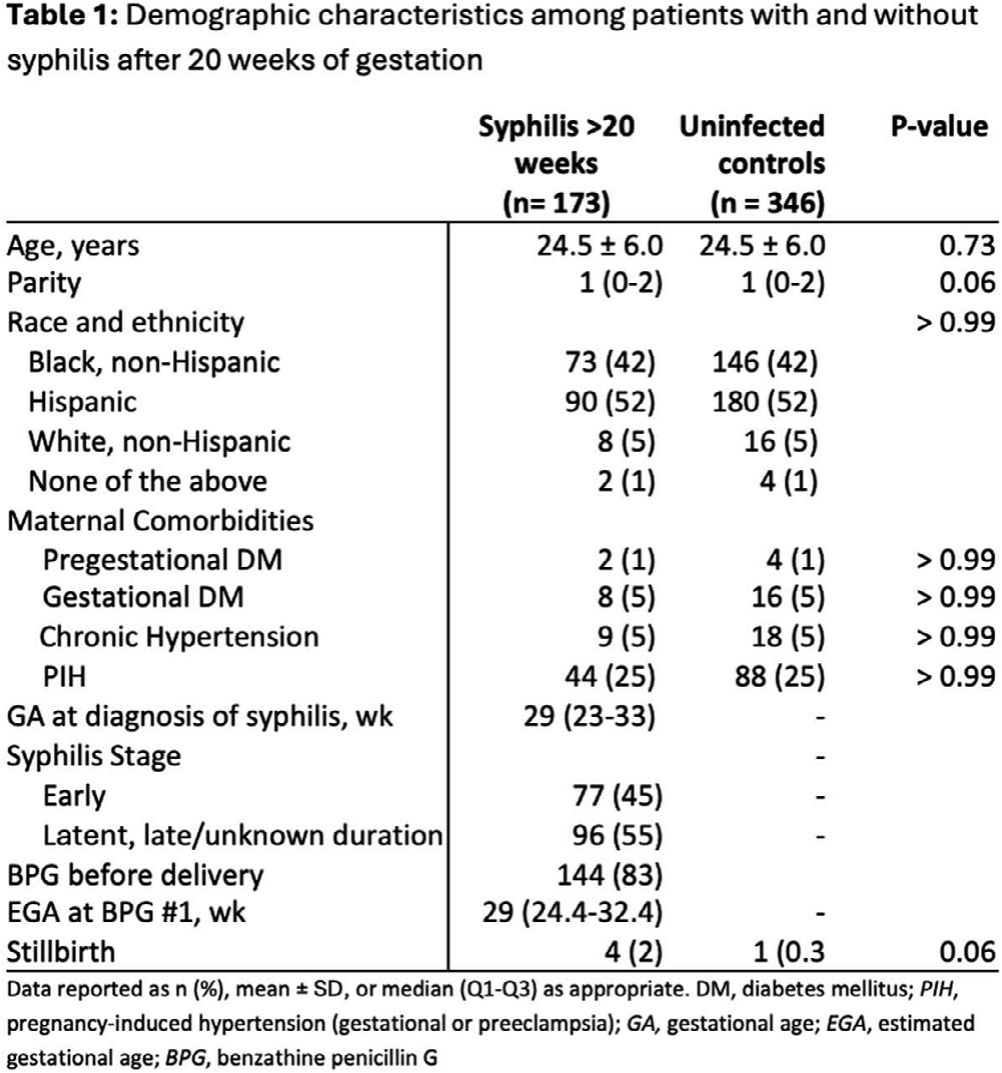

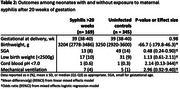

**10:30 AM – 12:00 PM**

## 121 | Outcomes of Radiofrequency Ablation for Multifetal Reduction Before and After 20 Weeks Gestation

Allison D. Perelman^1^; Sara E. Edwards^1^; Nicola F. Tavella^2^; Ariana Mills^3^; Jordan Davis^1^; Manasa G. Rao^4^; Kirema Garcia‐Reyes^3^; Angela T. Bianco^5^; Joanne L. Stone^5^; Chelsea A. DeBolt^1^

^1^Division of Maternal‐Fetal Medicine, Icahn School of Medicine at Mount Sinai, New York, NY; ^2^Division of Maternal‐Fetal Medicine, Icahn School of Medicine, Icahn School of Medicine at Mount Sinai Hospital, New York, NY; ^3^Division of Interventional Radiology, Icahn School of Medicine at Mount Sinai, New York, NY; ^4^Columbia University Irving Medical Center, New York, NY; ^5^Icahn School of Medicine at Mount Sinai, New York, NY

**Objective**: Previous studies have shown that in medically‐complicated monochorionic (MC) pregnancies undergoing radiofrequency ablation (RFA) for multifetal pregnancy reduction (MPR), severity of pregnancy complication rather than gestational age (GA) at procedure time is the main driver of outcomes. RFAs performed at earlier GA have worse outcomes, likely due to early‐onset of severe MC complications. However, previous studies to determine optimal timing of RFA did not include elective RFAs. This study aimed to determine whether live birth and preterm delivery rates differ for MPR via RFA for all indications performed before and after 20 weeks GA.

**Study Design**: This was a retrospective cohort study of MC gestations undergoing MPR via RFA between 2008 and 2025 at a single institution. Chi‐squared and Fisher's exact tests compared proportional differences, and multivariable logistic regression models were used to estimate the likelihood of live birth and preterm delivery.

**Results**: 108 patients underwent RFA with documented GA at time of procedure (Table 1). 88 (81.5%) had RFA performed before 20 weeks GA and 20 (18.5%) after. The later GA group had a significantly higher proportion of MPR for selective indications (defined as a complication of MC or fetal anomaly) compared to the earlier GA group (90% vs 34.1%, *p* < 0.001). The later GA group also had higher rates of preterm delivery (68.8% vs 18.3%, *p* < 0.001) (Table 2). When adjusting for confounders, including the indication for MPR, there was no difference in likelihood of live birth (aOR 0.28, 95% CI 0.02–13.08) or preterm delivery (aOR 0.17, 95% CI 0.01–1.2).

**Conclusion**: In a retrospective cohort, RFAs for MPR of MC pregnancies after 20 weeks GA were more likely to be performed for selective reductions and had higher rates of preterm delivery. However, when adjusting for the indication for MPR (selective vs elective), there was no difference in the rate of preterm delivery or live birth rate. This study supports prior findings that suggest the underlying indication for the reduction is more predictive of poor outcomes than the GA at the time of the procedure.

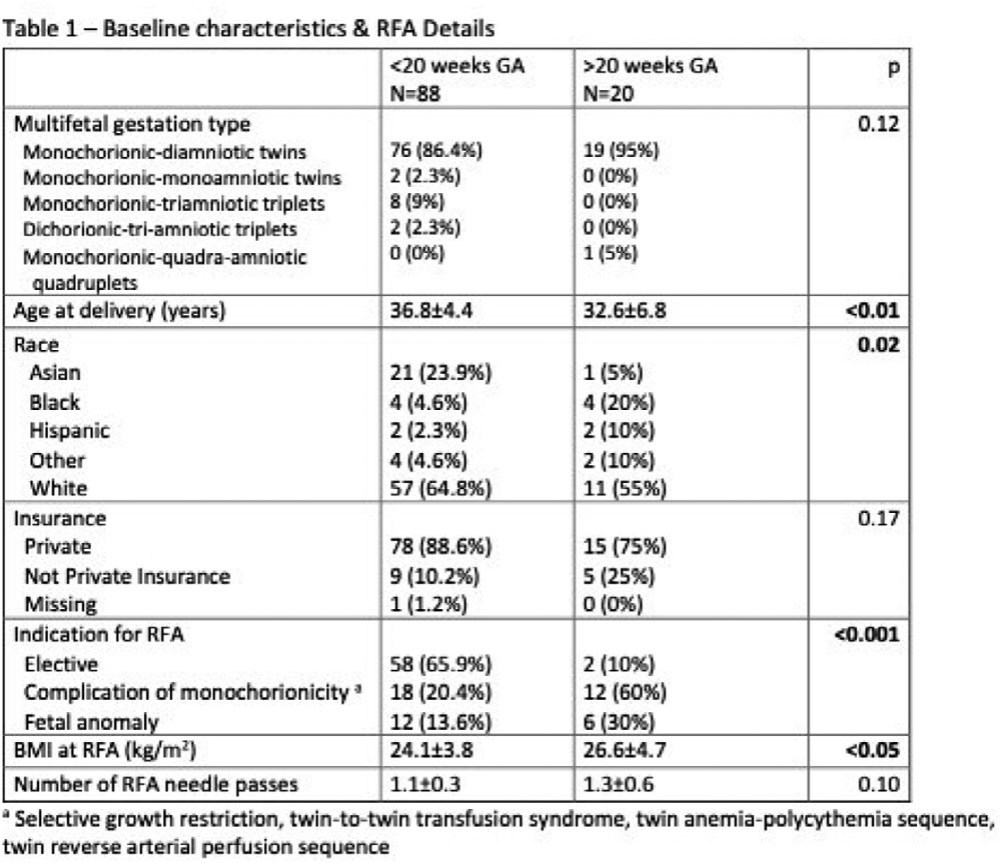

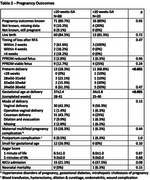

**10:30 AM – 12:00 PM**

## 122 | Trends in Non‐Medically Indicated Induction of Labor at 39 Weeks by Insurance Type

Alyssa R. Hersh^1^; Bharti Garg^2^; Kristin C. Prewitt^1^; Aaron B. Caughey^2^

^1^Oregon Health & Science University, Portland, OR; ^2^Oregon Health & Science University, Oregon Health & Science University, OR

**Objective**: The ARRIVE trial was published in 2018 and quickly led to an increase in utilization of non‐medically indicated induction of labor (IOL) at 39 weeks. Given possible disparities in uptake, we sought to assess whether there was a difference in the rate of IOL compared with expectant management (EM) by insurance type.

**Study Design**: This was a retrospective cohort study of singleton, non‐anomalous births in California between 2017–2020. We only included gestational ages 39–41 weeks and stratified by IOL versus EM. Medical indications for IOL and planned cesarean deliveries were excluded. We examined the utilization of IOL by year and stratified by insurance type to examine the trend in each group.

**Results**: There were 345,597 births included in this analysis, of which 178,054 had private insurance and 167,543 had public insurance. The proportion of births managed with IOL increased over time for both groups but was higher for those with private insurance each year compared to public insurance (Figure). Furthermore, the rate at which IOL increased over time was significantly higher for those with private insurance than public insurance.

**Conclusion**: The utilization over time of non‐medically indicated IOL was significantly higher for those with private insurance than public insurance and increased at a faster rate. Future studies should assess the factors driving these differences and resultant patient outcomes.

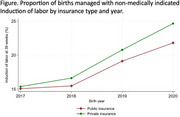

**10:30 AM – 12:00 PM**

## 123 | Mode of Delivery at 22 Weeks Gestational Age: A Cost‐Effectiveness Analysis

Amanda M. Baucom^1^; Robert M. Rossi^2^; Clarice G. Zhou^3^; Aaron B. Caughey^4^

^1^University of Cincinnati, Cincinnati, OH; ^2^Department of Obstetrics and Gynecology Division of Maternal‐Fetal Medicine University of Cincinnati College of Medicine, Cincinnati, OH; ^3^Division of Maternal‐Fetal Medicine, Department of Obstetrics and Gynecology, NYU Grossman School of Medicine, NYU Grossman School of Medicine, NY; ^4^Oregon Health & Science University, Oregon Health & Science University, OR

**Objective**: The American College of Obstetricians and Gynecologists (ACOG) and the Society for Maternal‐Fetal Medicine (SMFM) currently do not recommend emergent cesarean delivery for infants born at 22 weeks’ gestation. However, emerging data suggests potential neonatal survival benefits. Given the established maternal risks associated with emergent cesarean delivery, this study aimed to evaluate the cost‐effectiveness of delivery mode at 22 weeks’ gestation, with particular emphasis on long‐term reproductive outcomes.

**Study Design**: A decision‐analytic model was developed using TreeAge software to compare emergent cesarean versus no emergent cesarean at 22 weeks’ gestation. A theoretical cohort of 1693 pregnant individuals was modeled based on an estimated U.S. annual incidence of 0.05% of deliveries at this gestation. Neonatal survival, neurologic outcomes, and maternal outcomes in subsequent pregnancies were evaluated in terms of costs (2025 USD) and quality‐adjusted life years (QALYs) over a lifetime horizon. Model parameters were derived from literature. Incremental cost‐effectiveness ratios (ICERs) were calculated as cost per QALY gained, employing a willingness‐to‐pay threshold of $100,000/QALY.

**Results**: In this theoretical model, emergent cesarean delivery at 22 weeks’ gestation resulted in 366 additional survivors compared to vaginal delivery, of whom 165 were neurologically intact. In subsequent pregnancies, prior classical cesarean delivery was associated with increased rates of placenta previa and placenta accreta spectrum. The incremental cost of emergent cesarean delivery relative to vaginal delivery was $199,880 per neonate, with an associated gain of 4.73 QALYs, resulting in an ICER of $42,258 per QALY. One‐way sensitivity analysis demonstrated that emergent cesarean delivery remained cost‐effective if infant survival exceeded 25.0%.

**Conclusion**: These findings suggest that, under current neonatal outcome data, emergent cesarean delivery at 22 weeks’ gestation may be a cost‐effective strategy compared to vaginal delivery, even when long‐term implications for subsequent pregnancies are considered.

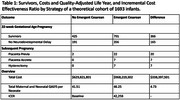

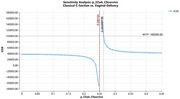

**10:30 AM – 12:00 PM**

## 124 | Effect of Aspirin Exposure on Placental Pathology in Fetal Growth Restriction

Amberly Lao^1^; Julia Kim^2^; Justin Gimoto^2^; Maria Gomez^2^; Annie Rozenblyum^2^; Soo Young Kim^2^; Poonam Khullar^3^; Patricia Rekawek^2^; Martin Chavez^2^; Lakha Prasannan^4^

^1^NYU Langone Hospital‐ Long Island, NYU Langone Hospital‐ Long Island, NY; ^2^NYU Grossman Long Island School of Medicine, Mineola, NY; ^3^Department of Pathology, NYU Grossman School of Medicine, Mineola, NY; ^4^NYU Long Island, Long Island City, NY

**Objective**: Evaluation of placental pathology in fetal growth restriction (FGR) has been limited. Patients with FGR are often exposed to low‐dose aspirin (LDA) for preeclampsia (PEC) prevention due to shared mechanisms of placental insufficiency. However, data on placental findings in this context are scarce. The objective of this study was to evaluate the placental pathology in pregnancies complicated by FGR with and without LDA exposure.

**Study Design**: This was a retrospective cohort study of singleton pregnancies complicated by FGR with confirmed small for gestational age (SGA) neonates at a single institution from 2020 to 2024. FGR was defined as an estimated fetal weight (EFW) and/or abdominal circumference (AC) <10th percentile. Chromosomal abnormalities, major fetal anomalies, multiple gestations, delivery prior to 24 weeks, or discarded placentas were excluded. LDA was defined as 81mg or 162mg daily starting at 11 weeks. ANOVA and Pearson's X^2^ tests with *p* < 0.05 were used and multivariate regression was applied to adjust for confounders.

**Results**: Among 111 pregnancies, 70 were not exposed and 41 were exposed to aspirin.

LDA exposed patients were more likely to have chronic hypertension (7.3% vs 0%, *p* = 0.007) and autoimmune disease (7.3% vs 0%, *p* = 0.02). Persistent muscularization was more frequent with aspirin (9.8% vs 1.4%, *p* = 0.04), and segmental avascular villi were less frequent (0% vs 12.9%, *p* = 0.02). Fetal vascular malperfusion was lower with aspirin (9.8% vs 20%), though not statistically significant. On multivariable regression, persistent muscularization lost significance; regression was not performed for segmental avascular villi due to low frequency.

**Conclusion**: Aspirin exposure may reduce placental findings associated with fetal vascular malperfusion in FGR, particularly segmental avascular villi. Larger studies are needed to evaluate aspirin's histopathologic impact in this high‐risk cohort, including those who were eligible but did not receive aspirin.

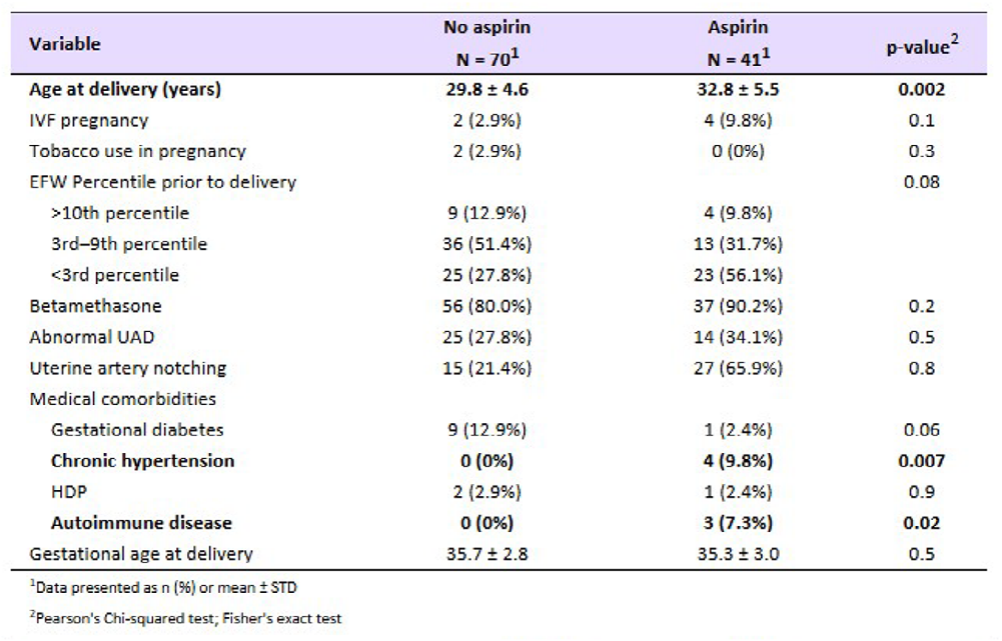

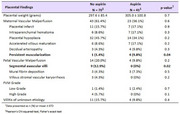

**10:30 AM – 12:00 PM**

## 125 | Optimal Timing of Delivery of Placenta Accreta With Previa: A Decision Analysis

Amelia H. Gagliuso^1^; Carolyn C. Green^2^; Lily Ben‐Avi^3^; Angie‐Mariana Bustos^2^; Aaron B. Caughey^3^

^1^Oregon Health and Science University, Portland, OR; ^2^Oregon Health & Science University, Portland, OR; ^3^Oregon Health & Science University, Oregon Health & Science University, OR

**Objective**: Placenta accreta spectrum (PAS) with placenta previa poses a significant risk of maternal morbidity and mortality. While scheduled delivery between 34 and 36 weeks is standard, the optimal gestational age for delivery remains uncertain. This study aimed to evaluate maternal and neonatal outcomes associated with different planned delivery timings for individuals with PAS and previa.

**Study Design**: A decision‐analytic model was constructed using TreeAge software to compare planned delivery at 33 + 0, 34 + 0, 35 + 0, 36 + 0, and 37 + 0 weeks gestation in those with PAS and placenta previa. The theoretical cohort included 2565 individuals, the approximate number of pregnant individuals affected by PAS and placenta previa in the U.S. Maternal outcomes included maternal death, ICU admission, and uncomplicated delivery resulting in healthy infants, while neonatal outcomes included neonatal death and neurodevelopmental delay. Probabilities, utilities, and costs were derived from literature. QALYs were discounted at a rate of 3%.

**Results**: Delivery at 36 weeks resulted in the highest number of healthy infants. When compared to delivery at 33 weeks, scheduled delivery at 36 weeks prevented 35 neonatal deaths and 22 cases of neurodevelopmental delay. Rates of maternal death and maternal ICU admission were comparable across gestational age of delivery. Overall, planned delivery at 36 weeks yielded the greatest benefit, resulting in 144,026 additional QALYS — the highest among all gestational ages evaluated. Compared specifically to planned delivery at 34 and 35 weeks, delivery at 36 weeks was associated with 875 and 289 more QALYs, respectively. An example of the condensed decision tree can be found in Figure 1.

**Conclusion**: Optimal timing of delivery for PAS with previa involves balancing maternal risks with neonatal complications. This decision analysis suggests that delivery at 36 weeks may minimize maternal and neonatal overall morbidity. Additional studies are needed to validate these findings and inform individualized delivery planning.

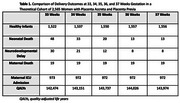

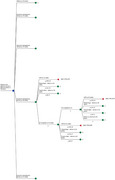

**10:30 AM – 12:00 PM**

## 126 | Term Delivery Following Antenatal Corticosteroid Exposure: Implications for Long‐Term Neurological Health

Amir Snir^1^; Omri Zamstein^2^; Tamar Wainstock^3^; Eyal Sheiner^4^

^1^Soroka University Medical Center, omer, HaDarom; ^2^Soroka University Medical Center, Soroka University Medical Center, HaDarom; ^3^Department of Epidemiology, Biostatistics and Community Health Sciences, Faculty of Health Sciences, Ben‐Gurion University of the Negev, Beer Sheva, HaDarom; ^4^Soroka University Medical Center, Faculty of Health Sciences, Ben‐Gurion University of the Negev, beer sheva, HaDarom

**Objective**: Antenatal corticosteroids (ACS) are routinely given to pregnancies at risk for preterm birth due to their known benefits in reducing neonatal morbidity and mortality. However, predicting preterm birth remains difficult, and many treated pregnancies ultimately deliver at term. We assessed the risk of childhood neurological morbidity among term‐born infants following ACS exposure.

**Study Design**: A population‐based cohort study was conducted at a tertiary referral center, including singleton term births. Infants were classified by exposure to ACS administered before 34 weeks’ gestation for suspected preterm labor. Neurological conditions during childhood were identified through community and hospital medical records. Cumulative incidence was estimated using Kaplan–Meier analysis, and Cox proportional hazards models were used to adjust for confounders.

**Results**: Among 182,626 term births, 2093 neonates (1.1%) were exposed to ACS before 34 weeks’ gestation. These pregnancies had higher rates of diabetes mellitus, hypertensive disorders, cesarean delivery, and earlier gestational age at birth (Table). During follow‐up, 30,616 children were diagnosed with neurological morbidity. The incidence rate was significantly higher in the exposed group (29.8 vs. 20.0 per 1000 person‐years; Kaplan–Meier log‐rank *p* < 0.001; Figure). In a multivariable Cox model, adjusting for maternal age, ethnicity, mode of delivery, gestational complications, and gestational age, ACS exposure remained independently associated with increased neurological morbidity (aHR = 1.11; 95% CI 1.05–1.17; *p* < 0.001). This association was also evident in a subgroup analysis restricted to early‐term births (37 + 0 to 38 + 6 weeks), with an aHR of 1.17 (95% CI 1.09–1.23; *p* < 0.001).

**Conclusion**: ACS exposure before 34 weeks was associated with increased neurological morbidity in children born at term. While essential in managing threatened preterm birth, these findings raise concern for long‐term effects when delivery occurs at term, supporting the need for further research to guide clinical decision‐making.

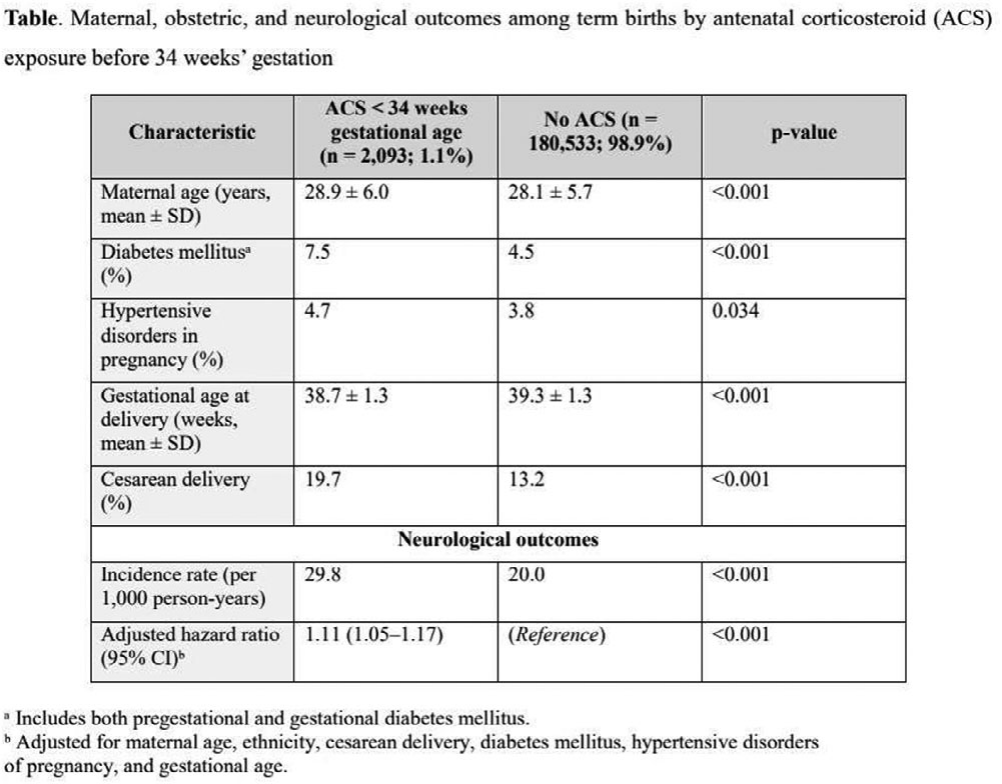

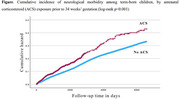

**10:30 AM – 12:00 PM**

## 127 | The Relationship Between Neonatal Hypoglycemia and Maternal Glucose Control During Labor of Individuals With T1D

Amna Jabarin^1^; Tali Cukierman‐Yaffe^1^; Shali Mazaki‐Tovi^2^; Keren Levi^1^; Ronen Fluss^1^; Nirit Agay^1^; Arnona Ziv^1^; Keren Ofir^3^; Hadel Watad^1^; Rakeft Yoeli Ullman^4^

^1^Sheba Medical Center, Ramat Gan, Tel Aviv; ^2^Department of Obstetrics and Gynecology, Sheba Medical Center, Ramat Gan, Tel Aviv; ^3^Sheba Medical Center, Sheba Medical Center, HaMerkaz; ^4^The Sheba Medical Center, The Sheba Medical Center, HaMerkaz

**Objective**: Neonates born to individuals with type 1 diabetes (T1D) are at increased risk for neonatal hypoglycemia. It is hypothesized that avoiding intrapartum maternal hyperglycemia and maintaining glucose levels within 70–110 mg/dL (ACOG) or 63–140 mg/dL (ADA) may reduce fetal hyperglycemia and neonatal hypoglycemia. This study determined the association between clinically significant neonatal hypoglycemia and intrapartum glucose control in individuals with T1D.

**Study Design**: This prospective cohort study included individuals with T1D followed at a multidisciplinary high‐risk pregnancy clinic at a single tertiary center. All used insulin pumps and continuous glucose monitoring. Maternal clinical variables, intrapartum glucose indices, and neonatal glucose levels were recorded. Clinically significant neonatal hypoglycemia was defined as requiring IV glucose. The association with intrapartum glucose control was analyzed.

**Results**: 76 individuals and their newborns were included; twin pregnancies were excluded. Compared to those whose neonates experienced hypoglycemia, individuals whose neonates did not, had a higher rate of time in range (TIR) and lower time above range (TAR) (63–140 mg/dL), both 1 hour (80% vs. 53%, *p* = 0.002; 16% vs. 42%, *p* = 0.01) and 24 hours (77% vs. 64%, *p* = 0.004; 15% vs. 27%, *p* = 0.01) before delivery. Time below range (TBR, < 63 mg/dL) did not differ significantly. Using the 70–110 mg/dL range, no significant differences in TIR, TAR, or TBR were observed between groups (Table 1). A higher rate of TIR (63–140 mg/dL) remained independently associated with lower odds of significant neonatal hypoglycemia at both time periods after adjusting for confounders including delivery mode, gestational age, large for gestational age (LGA), and first‐/third‐trimester TIR. The association at 24 hours lost statistical significance after adjusting for second‐trimester TIR. A similar trend was seen in multivariable analysis (Figure 1).

**Conclusion**: This study supports the association between improved intrapartum glucose control within 63–140 mg/dL range in T1D and reduced risk of significant neonatal hypoglycemia.

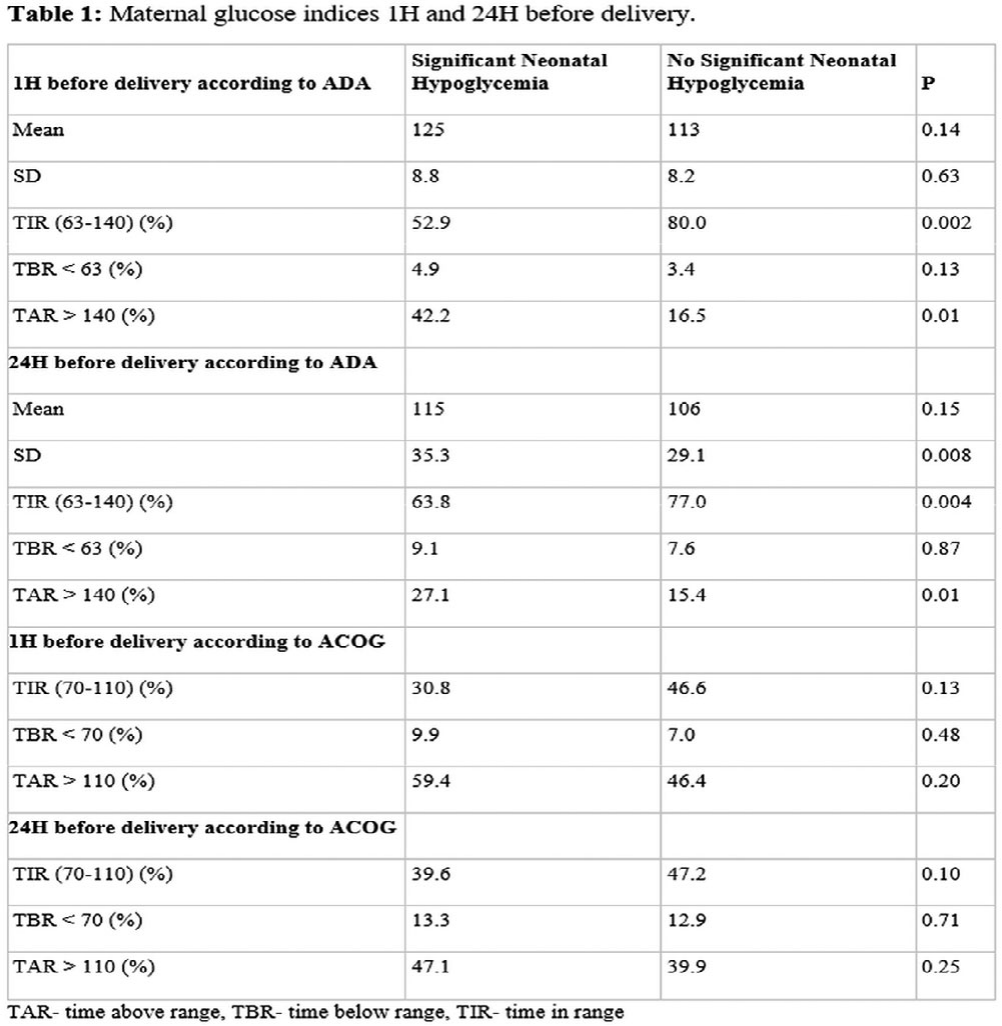

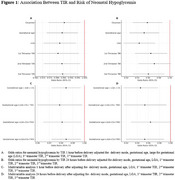

**10:30 AM – 12:00 PM**

## 128 | Use of Hydralazine as an Acute Antihypertensive in Pregnancy

Amy Tao^1^; Albert Tang^2^; Jason Bunn^1^; Donald D. McIntire^3^; Catherine Y. Spong^3^; C. Edward Wells^3^; Josiah S. Hawkins^3^; F. Gary Cunningham^3^

^1^University of Texas Southwestern Medical Center, University of Texas Southwestern Medical Center, TX; ^2^University of Texas Southwestern Medical Center, U, TX; ^3^University of Texas Southwestern Medical Center, Dallas, TX

**Objective**: In 2020, ACOG published algorithms for acute antihypertensive management, which recommend no more than 20 mg of hydralazine before switching to labetalol or seeking expert consultation. Our hospital has historically used a protocol with no upper limit for intravenous hydralazine. We sought to study the effect of the ACOG algorithm before (2017) and after (2021) the algorithm's publication.

**Study Design**: We obtained pharmacy data for acute antihypertensive therapy (intravenous hydralazine and labetalol, as well as oral immediate release nifedipine) for delivery admissions in 2017 and 2021. If a patient was readmitted in relation to the delivery admission, doses of acute antihypertensive therapy were included. Charts were retrospectively reviewed for timing of doses in relation to delivery and maternal and neonatal outcomes, as well as with respect to oral antihypertensive therapy.

**Results**: In 2017 there were 12,270 deliveries, and in 2021 there were 11,410 deliveries. A total of 572 (4.7%) and 623 (5.5%) of these patients for 2017 and 2021, respectively, received acute antihypertensive therapy associated with delivery admissions. The maximum individual doses of such intravenous hydralazine in 2017 and 2021 were 265 mg and 195 mg for delivery admissions and readmissions. Given that delivery admissions varied in length, we compared maximum doses of intravenous hydralazine in any 24‐hour period, which were 65 mg and 70 mg in 2017 and 2021, respectively (Table). No cardiopulmonary events or maternal ICU admissions were attributed to antihypertensive therapy.

**Conclusion**: The increased national attention to acute treatment of hypertension was associated with a higher proportion of patients acutely treated in 2021, with most patients requiring 20 mg or less of hydralazine in any 24‐hour period. Importantly, about 1‐in‐5 patients required doses exceeding 20 mg in any 24‐hour period. This study provides additional guidance for the use of hydralazine exceeding 20 mg per 24 hours, which is safe when indicated and used as part of a defined algorithm.

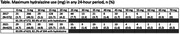

**10:30 AM – 12:00 PM**

## 129 | Basal Plate Myometrial Fibers and Risk of Placenta Accreta Spectrum in Subsequent Pregnancies: A Meta‐analysis

Kamran Hessami^1^; Anika C. Valery^1^; Madeline West^2^; Hendrik A. Lombaard^1^; Jessian L. Munoz^3^; Onur Turkoglu^1^; Alexander M. Saucedo^1^; Charlotte Kim^1^; Heather Keir^4^; Ahmed A. Nassr^5^; Michael A. Belfort^1^; Amir A. Shamshirsaz^6^

^1^Baylor College of Medicine, Houston, TX; ^2^Texas Tech University Health Sciences Center El Paso, El Paso, TX; ^3^Texas Children's Hospital, Texas Children's Hospital, TX; ^4^Baylor, Houston, TX; ^5^Baylor College of Medicine, Baylor College of Medicine, TX; ^6^Division of Maternal‐Fetal Medicine, Department of Obstetrics and Gynecology, Baylor College of Medicine, Houston, TX

**Objective**: Basal plate myometrial fibers (BPMF) refer to the histopathologic finding of myometrial fibers attached to the basal plate of the placenta, with or without intervening decidua. This systematic review was undertaken to determine whether the presence of BPMF on placental pathology is associated with increased risk of Placenta Accreta Spectrum (PAS) and need for peripartum hysterectomy in subsequent pregnancies.

**Study Design**: A systematic review of several databases was performed to identify studies reporting BPMF on placental pathology with a subsequent livebirth that also had placental pathology report. The study protocol was registered in PROSPERO (CRD420251103171). Statistical analysis was conducted using STATA, with pooled prevalence of PAS and peripartum hysterectomy as primary outcomes.

**Results**: Of 167 articles retrieved, three met inclusion criteria including 278 patients who had BPMF during their previous pregnancy. The pooled prevalence of PAS in subsequent pregnancies for this cohort of patients was 8% (95% CI: 0.00–0.17, I^2^ 83%). The prevalence of peripartum hysterectomy was 10% (95% CI: 0.08–0.29, I^2^ 79%).

**Conclusion**: These findings suggest that, following a prior diagnosis of BPMF in the index pregnancy, the prevalence of PAS and the need for peripartum hysterectomy in a subsequent pregnancy are approximately 8% and 10%, respectively.

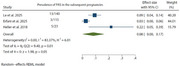

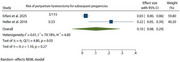

**10:30 AM – 12:00 PM**

## 130 | Maternal Hypoalbuminemia is Associated With Increased Postpartum Edema Among Patients With Hypertensive Disorders of Pregnancy

Anna K. Daoud^1^; Julia Rubin^2^; Mirely Garcia^3^; Ester Sanchez^4^; Kristin M. Voegtline^5^; Moeun Son^6^

^1^New York Presbyterian/ Weill Cornell Medicine, New York, NY; ^2^Weill Cornell Medical College, Weill Cornell Medical College, NY; ^3^NewYork‐Presbyterian/Weill Cornell Medicine, NewYork‐Presbyterian/Weill Cornell Medical Center, NY; ^4^NewYork‐Presbyterian/Weill Cornell Medicine, NewYork‐Presbyterian/Weill Cornell Medicine, NY; ^5^NewYork‐Presbyterian/ Weill Cornell Medicine, New York, NY; ^6^Weill Cornell Medical College, New York, NY

**Objective**: Serum albumin is physiologically decreased during pregnancy but can further decrease in those with hypertensive disorders of pregnancy (HDP). We aimed to assess if hypoalbuminemia at delivery admission in patients with hypertensive disorders of pregnancy (HDP) is associated with increased postpartum edema and diuretic use.

**Study Design**: This is a retrospective cohort study of pregnant persons with singleton gestations diagnosed with HDP who delivered at term at two hospitals from 2020 to 2024. Patients with chronic hypertension, kidney or liver disease, and those missing albumin values were excluded. Eligible individuals were identified via a research database but data were manually extracted from the medical record. Hypoalbuminemia was defined as < = 2.3 g/dL based on prior literature. Study outcomes included postpartum peripheral, pulmonary, and cerebral edema, and diuretic use. Patients with low serum albumin were compared to those with values >2.3 g/dL using Fisher's exact and Chi‐square tests with Bonferroni correction for multiple comparisons. Logistic regression was performed.

**Results**: Baseline characteristics of 572 included patients are shown in Table 1. Patients with hypoalbuminemia were more likely to have preeclampsia with severe features (OR 3.91 95% CI 2.58–5.94), postpartum peripheral edema documented (OR 1.44 95% CI 1.01–2.07), and be discharged on oral diuretics postpartum (OR 5.47 95% CI 3.77–7.93) (Table 2). After adjusting for delivery mode, hypoalbuminemia remained significantly associated with being discharged home with diuretics (*p* < .001), but not peripheral edema. Those who had a cesarean section were more likely to be discharged on diuretics (OR 1.92 95% CI 1.31–2.84) and have peripheral edema (OR 2.29 95% CI 1.57–3.34).

**Conclusion**: Patients with HDP and severe hypoalbuminemia are more likely to have documented postpartum peripheral edema and diuretic use. Albumin levels are readily available in labs collected for patients with HDP. In those with severe hypoalbuminemia, one can consider earlier diuretic use to prevent postpartum edema, but prospective data are needed.

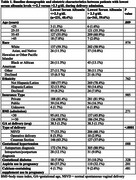

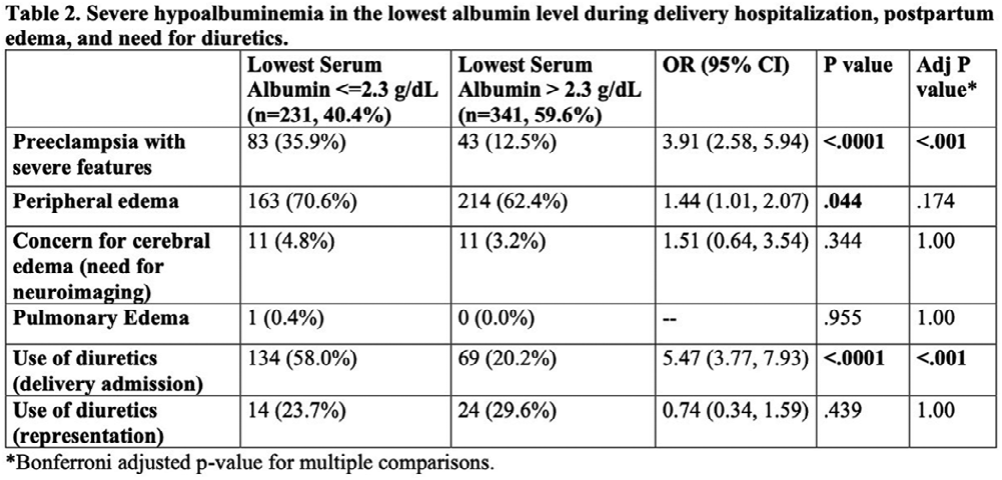

**10:30 AM – 12:00 PM**

## 131 | Postpartum BP Home Monitoring Highlights Risk in Chronic Hypertension vs. Pregnancy‐Induced Disorders

Anna Madden‐Rusnak^1^; Miriam J. Alvarez^1^; Alejandro Guevara^2^; Nandini Raghuraman^3^; George A. A. Macones^1^; Lorie M. Harper^1^; Alison G. G. Cahill^4^

^1^Department of Women's Health, Dell Medical School at the University of Texas at Austin, Austin, TX; ^2^Texas Tech University Health Sciences Center Paul L. Foster School of Medicine, El Paso, TX; ^3^Washington University School of Medicine, St. Louis, MO; ^4^Department of Women's Health, Dell Medical School at the University of Texas at Austin, Department of Women's Health, Dell Medical School at the University of Texas at Austin, TX

**Objective**: Postpartum blood pressure (BP) trends among patients with hypertensive disorders are not well characterized. This study aimed to characterize early postpartum BP trends during the first two weeks after delivery among underserved, Spanish‐speaking individuals with hypertensive disorders.

**Study Design**: This prospective observational study included postpartum individuals with gestational hypertension (gHTN), preeclampsia (Pre‐E), or chronic hypertension (cHTN) for remote BP monitoring. Participants self‐monitored BP twice daily for 2‐weeks in response to automated text message prompts, which provided real‐time feedback. Daily mean BP values were analyzed using a linear mixed‐effects model to assess BP control over time. Logistic regression was used to compare the proportion of participants in each diagnosis group with any elevated BP values (SBP ≥140 mmHg or DBP ≥90 mmHg).

**Results**: Seventy‐five individuals were enrolled, the majority of whom were Hispanic (87%), Spanish‐speaking (72%), and publicly insured (88%). Diagnoses included postpartum gHTN (51%), Pre‐E (40%), and cHTN (9%). Participants submitted a mean of 12 ± 8.4 BP readings. BP trends across the 14‐day study period differed significantly by diagnosis for systolic BP (*p* < 0.001) and diastolic BP (*p* < 0.01). BP increased daily in cHTN patients but decreased similarly for gHTN and Pre‐E. Elevated BP rates were highest in cHTN (30%), then Pre‐E (27%), and gHTN (23%), though differences were not statistically significant.

**Conclusion**: This pilot study found that postpartum individuals with cHTN had progressively rising BP values and higher rates of elevated BP, compared to those with hypertensive disorders of pregnancy. Unlike hypertensive disorders of pregnancy, which generally stabilize within the first week postpartum, cHTN may require extended monitoring periods beyond this period. Implementing home BP monitoring with real‐time feedback could help address care gaps, particularly in high‐risk Hispanic populations, as shown in other at‐risk populations.

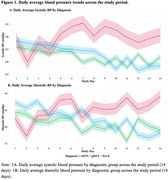

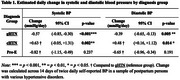

**10:30 AM – 12:00 PM**

## 132 | A Randomized Controlled Trial of Postprandial Ambulation Among Individuals With Gestational Diabetes Mellitus

Anna R. Whelan^1^; Margaret M. Thorsen^2^; Brock E. Polnaszek^3^; Richard Tucker^4^; Allison Payne^5^; Martha Kole‐White^2^

^1^UMass Chan Medical School, Worcester, MA; ^2^Women & Infants Hospital of Rhode Island / Warren Alpert Medical School of Brown University, Providence, RI; ^3^Medical College of Wisconsin, Milwaukee, WI; ^4^Women & Infants Hospital of Rhode Island, Providence, RI; ^5^Women & Infants Hospital of Rhode Island, Alpert Medical School of Brown University, Providence, RI

**Objective**: While low to moderate intensity exercise is recommended for the management of Gestational Diabetes Mellitus (GDM), the optimal timing of exercise in relation to eating remains debated. This study aimed to investigate glycemic, obstetric and neonatal outcomes of timed ambulation in the 2‐hour postprandial period compared to unscheduled ambulation.

**Study Design**: A single‐center RCT was conducted from Oct 2022 to Oct 2024. Individuals with newly diagnosed GDM were recruited from a tertiary maternal center. After written informed consent was obtained, participants were randomized to guided counseling for either 1) postprandial ambulation (walk for 15–20 minutes within 2 hours of 2 meals a day) or 2) unscheduled 30–40 minutes once a day (routine counseling). Participants were instructed to wear a study‐provided Fitbit Inspire activity monitor during all waking hours until delivery. Demographic characteristics, medical history, and pregnancy management and outcomes were abstracted from the medical chart. We estimated that 46 participants will provide 80% power to detect a 15% difference in the primary outcome of neonatal birthweight percentile.

**Results**: A total of 46 individuals were enrolled with 23 randomized to postprandial ambulation. Characteristics of the study sample are described in Table 1. Compliance with Fitbit monitor usage was similar between groups. There was no significant difference in the primary outcome of neonatal birthweight percentile with routine counseling compared to postprandial ambulation counseling (49.7% vs 53.7%, *p* = 0.69). Similarly, there were no differences in other measures of glycemic control, obstetric or neonatal outcomes (Table 2).

**Conclusion**: In this pilot, receiving counseling to ambulate within 2 hours after meals compared to unscheduled ambulation did not significantly affect glycemic, obstetric, and neonatal outcomes among individuals with GDM. Further study is needed to determine whether the lack of observed effects reflects limited adherence or a true absence of benefit from postprandial ambulation timing.

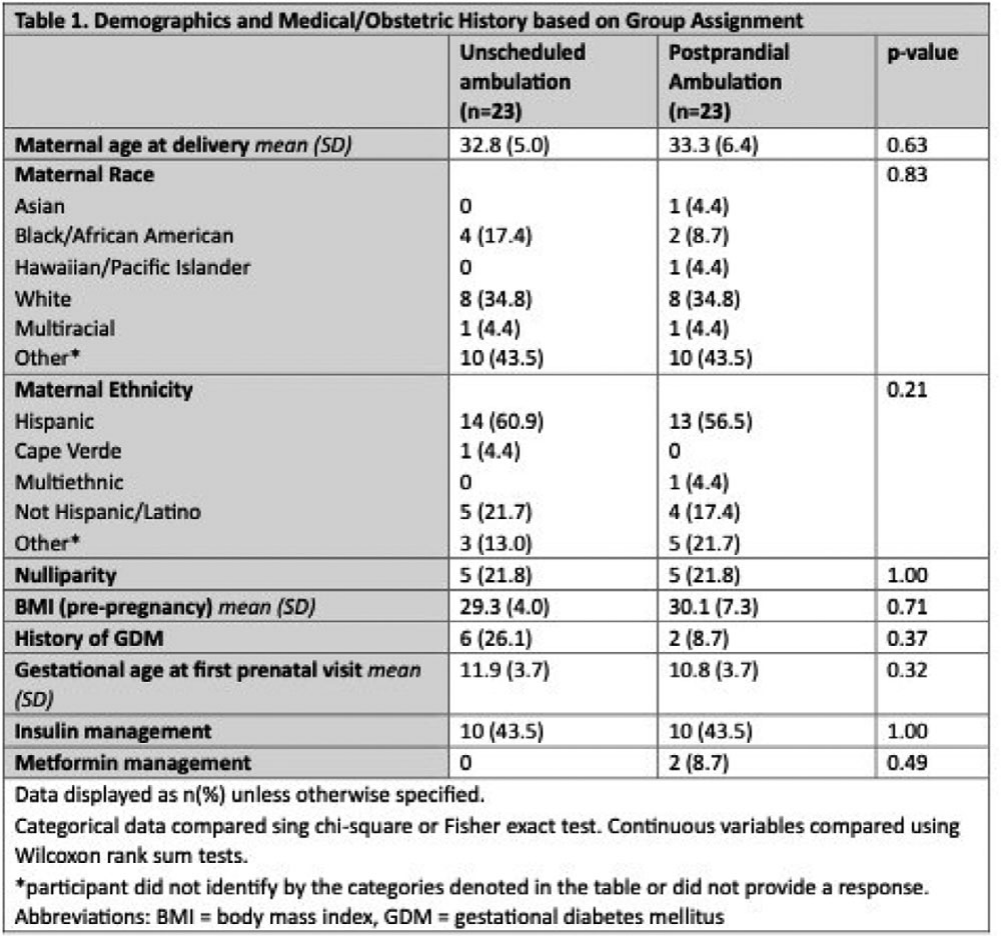

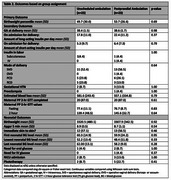

**10:30 AM – 12:00 PM**

## 133 | Individual Bile Acid Trends in Cholestasis of Pregnancy

Anne M. Ambia^1^; Jeffrey McDonald^2^; Yevgenia Y. Fomina^3^; Tamia Harris‐Tryon^2^; Andrea Rizkallah^2^; C. Edward Wells^1^; Catherine Y. Spong^1^; Donald D. McIntire^1^; David B. Nelson^1^

^1^University of Texas Southwestern Medical Center, Dallas, TX; ^2^University of Texas Southwestern, Dallas, TX; ^3^University of Texas Southwestern, university of Texas Southwestern, TX

**Objective**: Intrahepatic cholestasis of pregnancy (ICP) is characterized by elevated total bile acids (BA) as a disease unique to pregnancy with adverse perinatal effect. We have previously established that glycol and tauro conjugated BA levels were increased in ICP. There is a paucity of information on changes in these individual BA levels over time. We aimed to measure individual BAs serially in those with ICP to observe potential treatment effect of ursodiol.

**Study Design**: This was a prospective, observational cohort study of pregnant individuals with ICP(total BA were ≥10 µmol/L using a quantitative enzymatic assay) from Feb 2022 to Aug 2023 in a single academic center. At the time of initial evaluation, a blood sample was collected for analysis of individual BAs. A second sampling was performed at 35–38 weeks at least 2 weeks after the initial analysis. Extraction of BAs were performed through liquid chromatography mass spectrometry methods. Estimations are handled through the model with linear contrasts. *p* values and confidence intervals are adjusted for multiple comparisons through the Tukey‐Kramer method.

**Results**: There were 29 individuals with ICP. The mean gestational age at initial and subsequent sampling was 35.2 ± 3.8 and 36.5 ± 1.2 weeks. The mean total BA was 40 ± 41.6 at initial sampling and decreased to 26.2 ± 29.5 at subsequent sampling following treatment. When comparing serial samples in those with ICP, a statistically significant decrease in levels of taurocholic (*p* < 0.01), taurochenodeoxycholic acid (*p* < 0.001), and taurolambdamuricholic acid (*p* < 0.001) were noted. Glycolithocholic acid was noted to significantly increase from the initial to the subsequent sampling (*p* < 0.001) Table.

**Conclusion**: Data on sequentially measured individual BA profiles in those with ICP demonstrates overall decrease in total BAs. Importantly, significant decreases in several tauro‐conjugated BAs with ursodiol treatment were identified whereas glycolithocholic acid was noted to significantly increase. Understanding the specific BA profile of ICP patients receiving treatment may inform future management strategies.

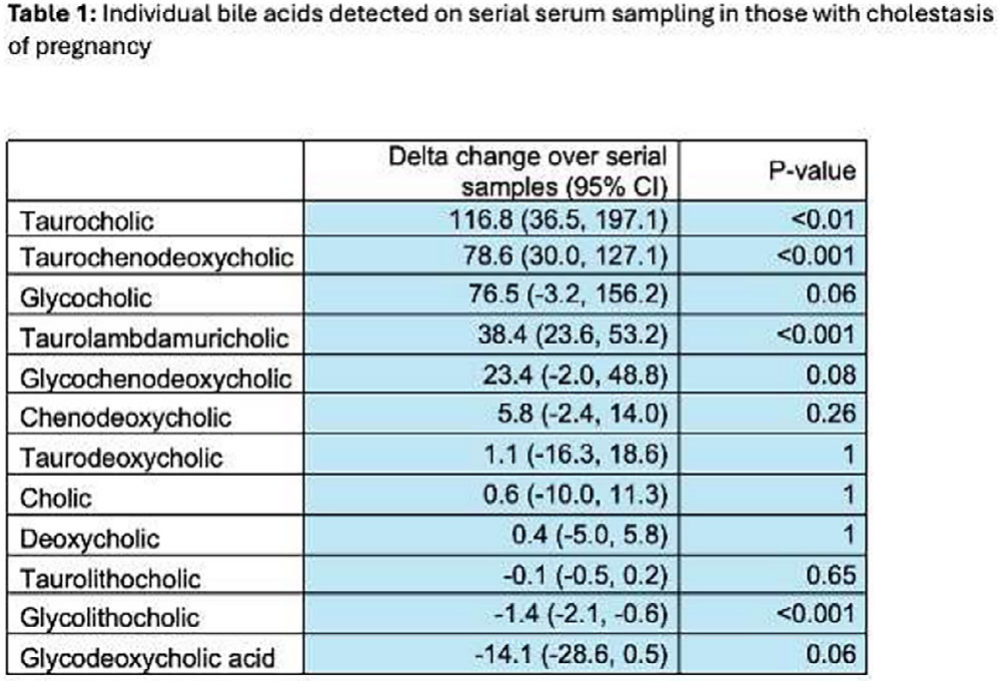

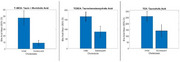

**10:30 AM – 12:00 PM**

## 134 | Benzodiazepine Prescription During Pregnancy and Factors Associated With Sedative Use Disorder

Anthony M. Kendle^1^; Alexandra Hoeman^2^; Chaitanya Chaphalkar^3^; Tanner G. Wright^4^

^1^Department of Obstetrics and Gynecology, University of South Florida, Tampa, FL; ^2^The University of South Florida Morsani College of Medicine, Tampa, FL; ^3^Office of Research, University of South Florida, Tampa, FL; ^4^Department of Pediatrics, The University of South Florida, Tampa, FL

**Objective**: Benzodiazepines (BZD) are frequently prescribed during pregnancy despite lacking efficacy for treating mental health disorders and unfavorable safety profile. Since they are increasingly involved in overdose‐related deaths, we aimed to characterize BZD prescribing patterns and risks for sedative use disorder.

**Study Design**: We conducted a retrospective cohort study of all patients who received prescription BZD during pregnancy and delivered >20 weeks’ gestation at a tertiary care center from January 2014 to June 2024. Patients were excluded if they received BZD only in the context of peri‐procedural anesthesia. Medical records were extracted for sociodemographic and delivery characteristics. International Disease Classification codes identified patients with a coded diagnosis of sedative use disorder and conditions such as seizure, mental health, and substance use disorders. Groups were compared using chi‐square and Fisher's Exact test. Log binomial regression was used to generate risk ratios (RR) and 95% confidence intervals (CI). A *p* < 0.05 was considered significant.

**Results**: During the study period, 568 deliveries occurred to 537 patients. Alprazolam (36.1%), diazepam (28.9%), and clonazepam (25.4%) were the most prescribed BZD. Sedative use disorder was identified in 23 (4.0%) of deliveries. Those with sedative use disorder were more likely to have a prescription for alprazolam (56.5% vs 34.3%, *p* = 0.03), diagnosis of other substance use disorder (82.6% vs 19.4%, *p* < 0.001), and diagnosis of any mental health condition (95.7% vs 74.7%, *p* = 0.02). There was no difference in diagnosis of seizure disorder. Patients with opioid use disorder who are prescribed BZD are more likely to have a diagnosis of sedative use disorder (RR 16.1; 95% CI: 6.5, 39.5). There remained 9‐fold increased risk after adjusting for other substance use and mental health disorders (aRR 9.1; 95% CI: 3.4, 24.6).

**Conclusion**: It is important to evaluate for sedative use disorder among patients prescribed BZDs during pregnancy, particularly those prescribed alprazolam or with co‐existing opioid use disorder.

**10:30 AM – 12:00 PM**

## 135 | Cervical Softening Differs by Maternal BMI and Weight Gain: A Longitudinal Elastography Study

Antonina I. Frolova^1^; Methodius G. Tuuli^2^; Adam K. Lewkowitz^3^; Julie Tumbarello^4^; Emily Diveley, BSN^5^; Crystal F. Ware^3^; Madeline Felske^4^; Peinan Zhao^5^; Molly J. Stout^4^

^1^Washington University School of Medicine, St. Louis, MO; ^2^Department of Obstetrics and Gynecology, Warren Alpert Medical School at Brown University, and Women & Infants Hospital of Rhode Island, Providence, RI; ^3^Women & Infants Hospital of Rhode Island / Warren Alpert Medical School of Brown University, Providence, RI; ^4^University of Michigan, Ann Arbor, MI; ^5^Washington University School of Medicine, Washington University School of Medicine, MO

**Objective**: High BMI is associated with labor outcomes including delayed onset of labor and higher risk for cesarean. It is unclear whether these observations are physiologic, due to clinician behaviors, or both. Using a device that fully quantifies numeric cervical tissue stiffness (FQ‐CES), we tested the association between BMI and cervical tissue stiffness across gestation.

**Study Design**: This is a prospective cohort study of singleton pregnancies at 3 academic centers. Cervical tissue stiffness was measured using FQ‐CES in all three trimesters. Linear mixed effects models tested the association between BMI and cervical softening patterns (log‐scaled Young's Modulus). BMI (kg/m^2^) characteristics examined were: BMI at start of prenatal care, BMI at the end of pregnancy, and BMI change over pregnancy. High BMI was dichotomized as >33 in early pregnancy, >38 at end of pregnancy (either delivery or last prenatal visit), and high BMI change >7.5 based on threshold testing of continuous data. Analyses were stratified by parity.

**Results**: Of 372 individuals, median BMI was 27.4 in early and 31.8 in late pregnancy, with a median change of 4.1. High BMI in early pregnancy (*p* = 0.03), at the end of pregnancy (*p* = 0.002), and higher BMI change over pregnancy (*p* = 0.001) were all associated with lower cervical stiffness (softer cervix). In a multivariable model including all three BMI metrics and parity, only higher BMI change remained significantly associated with reduced cervical stiffness (38.1% lower Youngs Modulus, *p*‐value = 0.005; Table and Figure). In multivariable analysis stratified by parity, change in BMI over pregnancy remains associated with cervical softening in multiparous patients (38.7% lower Youngs Modulus, *p* = 0.01; Table).

**Conclusion**: Higher BMI change over pregnancy is associated with softer cervixes. Delayed cervical remodeling processes may not explain previously documented associations between obesity and labor outcomes. These findings also support individualized approaches to labor induction that may account for clinical characteristics measurable with FQ‐CES.

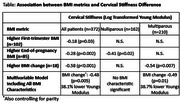

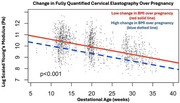

**10:30 AM – 12:00 PM**

## 136 | Does Equilibrio Therapy Improve ECV Success? Insights From a CHAID Decision Tree Model

Ariel Gimple^1^; Itay Shavit^1^; Sharon Maslovitz^1^; Itamar Gilboa^2^; Emmanuel Attali^2^; Yariv Yogev^3^; Daniel Gabbai^2^

^1^Lis Hospital for Women's Health, Tel Aviv Sourasky Medical Center, Tel Aviv, Tel Aviv; ^2^Lis Hospital for Women's Health, Tel Aviv Sourasky Medical Center, Lis Hospital for Women's Health, Tel Aviv Sourasky Medical Center, Tel Aviv; ^3^Lis Hospital for Women's Health, Tel Aviv Sourasky Medical Center Gray Faculty of Medicine, Tel Aviv University, Israel, Lis Hospital for Women's Health, Tel Aviv Sourasky Medical Center, Tel Aviv

**Objective**: To evaluate whether equilibrio therapy, a structured therapeutic massage aimed at promoting musculoskeletal and pelvic balance, is associated with increased success rates of external cephalic version (ECV).

**Study Design**: This retrospective case‐control study was conducted in 2025 at a single university‐affiliated tertiary medical center. All women with breech presentation who underwent ECV were included. All ECV procedures were performed by a single experienced obstetrician. Immediately following the procedure, participants were asked whether they had received equilibrio therapy within the preceding week. Maternal and obstetric characteristics were collected and compared between women with successful and unsuccessful ECV outcomes. Univariate analysis and CHAID (Chi‐squared Automatic Interaction Detector) decision tree modeling were used to explore predictors of success.

**Results**: 1. A total of 87 women underwent ECV during the study period. Among them, 51 (58.6%) had a successful procedure, while 36 (41.4%) were unsuccessful.

2. In univariate analysis, equilibrio therapy was not associated with ECV success in the overall cohort. However, nulliparity was significantly associated with ECV failure (75% vs. 38%, *p* < 0.001), as was higher birthweight (3322 g vs. 2987 g, *p* < 0.05). No other maternal or obstetric characteristics, including age, gestational age, BMI, or history of cesarean (SPCS), were significantly associated with ECV outcomes.

3. In the CHAID decision tree model, nulliparity was the most significant predictor of ECV failure (adjusted *p* = 0.002). Among multiparous women, equilibrio therapy was associated with a higher ECV success rate (80.0% vs. 64.0%, adjusted *p* = 0.007).

**Conclusion**: Equilibrio therapy was associated with increased success rates of ECV among multiparous women. These findings suggest a potential benefit of equilibrio treatment in selected populations; however, further studies with larger sample sizes are needed to validate these results and assess clinical applicability.

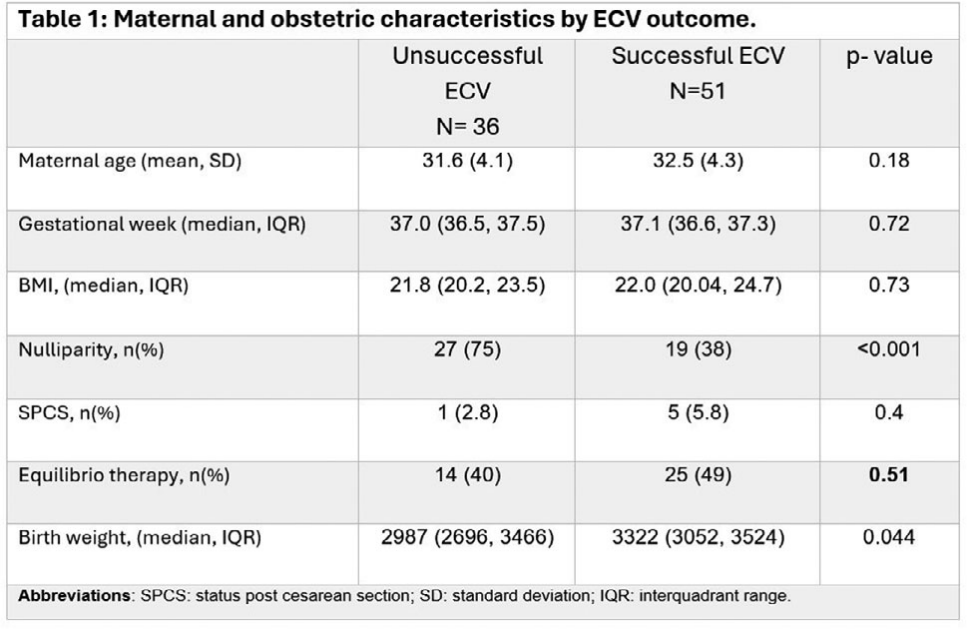

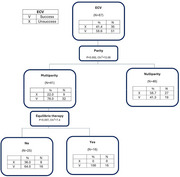

**10:30 AM – 12:00 PM**

## 137 | Discharge on Nifedipine Associated With Lower Self‐Reported Systolic Blood Pressure Two Weeks Postpartum

Audra C. Fain^1^; Jenny Y. Mei^2^; Maral Demirjian^3^; Lorna Kwan^3^; Rashmi Rao^4^; Thalia Mok^5^

^1^Department of Obstetrics and Gynecology, David Geffen School of Medicine at University of California, Los Angeles (UCLA), Los Angeles, CA; ^2^Stanford University, Stanford University, CA; ^3^Department of Urology, David Geffen School of Medicine at University of California, Los Angeles (UCLA), Los Angeles, CA; ^4^Division of Maternal Fetal Medicine, Department of Obstetrics and Gynecology, David Geffen School of Medicine at University of California, Los Angeles (UCLA), Los Angeles, CA; ^5^David Geffen School of Medicine at University of California, Los Angeles (UCLA), Los Angeles, CA

**Objective**: Enhanced postpartum (PP) blood pressure (BP) control after hypertensive disorders of pregnancy (HDP) may lower long‐term cardiovascular risk, but the most effective antihypertensive remains unclear. We compared self‐reported PP BP among patients with HDP or chronic hypertension (CHTN) by antihypertensive medication.

**Study Design**: Retrospective cohort study of patients with HDP or CHTN delivered at a quaternary care center between 2022 to 2025 and enrolled in a remote BP monitoring program upon discharge. Patients were included if discharged on either nifedipine or labetalol and logged at least one BP value. Those discharged on two or more medications were excluded. Primary outcome was mean self‐reported systolic BP, diastolic BP, and mean arterial pressure (MAP) over the first 14 days PP. Secondary outcomes included self‐reported 6‐week PP systolic and diastolic BP. Primary and secondary outcomes were compared by antihypertensive agent with Chi‐Square or Fisher's exact and Wilcoxon rank‐sum tests.

**Results**: 785 pregnancies with HDP or CHTN delivered and were discharged on a single antihypertensive agent: 650 on nifedipine, 135 on labetalol. The labetalol cohort was more likely to be obese, multiparous, and have CHTN (Table 1, *p* < 0.05). Self‐reported systolic BP over the first 14 days PP were significantly lower in the nifedipine cohort (123.8 ± 7.2 vs. 126.8 ± 9.5, *p* = 0.003). There was no difference in self‐reported diastolic BP or MAP over the first 14 days PP, or in self‐reported 6‐week BP values between groups (Table 2, *p* > 0.05).

**Conclusion**: Discharge on nifedipine was associated with a modest but statistically significant reduction in self‐reported systolic BP the first two weeks PP compared to labetalol. However, there were no significant differences in other BP measurements by antihypertensive agent, suggesting comparable overall PP BP control. Further studies are needed to determine if reduction of systolic BP in the immediate PP period reduces long‐term cardiovascular risks. Current findings support individualized antihypertensive selection based on patient‐specific factors.

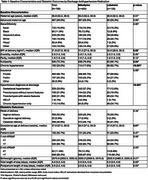

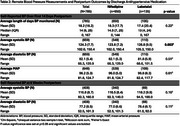

**10:30 AM – 12:00 PM**

## 138 | Association Between Placenta Accreta Spectrum and Postpartum Depression: A Retrospective Case‐Control Study

Austin Oberlin^1^; Sara Giuliano^2^; Lisbet S. Lundsberg^2^; Jennifer F. Culhane^2^; Caitlin Partridge^2^; Colleen Sinnott^3^; Rachel D. Seaman^4^; Yang Yang‐Hartwich^2^; Sonya S. Abdel‐Razeq^5^; Olga Grechukhina^3^

^1^Yale University, New Haven, CT; ^2^Department of Obstetrics, Gynecology, and Reproductive Sciences, Yale School of Medicine, New Haven, CT; ^3^Division of Maternal Fetal Medicine, Yale School of Medicine, New Haven, CT; ^4^Yale School of Medicine, New Haven, CT; ^5^Division of Maternal‐Fetal Medicine, Department of Obstetrics, Gynecology, and Reproductive Sciences, Yale University, New Haven, CT

**Objective**: Placenta accreta spectrum (PAS) is a known risk factor for adverse mental health outcomes, though a more robust understanding of the relationship is needed. Here we aim to assess if: 1) PAS is associated with increased risk of postpartum depression (PPD) and if 2) the timing of the diagnosis and peripartum events further affect this risk.

**Study Design**: This retrospective case‐control study utilized deliveries at a single hospital system between 2013–2025. PAS cases were identified by ICD‐10 codes and verified by manual review. Controls were matched 2:1 for age, parity, and mode of delivery. Cases were manually reviewed to determine timing of PAS diagnosis (antenatal or intrapartum). The primary outcome was PPD defined as either an ICD‐10 code for depression or an Edinburgh Postnatal Depression Scale (EPDS) ≥10 up to one year postpartum. Bivariate analysis was performed followed by multivariate analyses using statistically significant co‐variates. A sub‐analysis of PAS patients, stratified by timing of the diagnosis and hysterectomy, was performed.

**Results**: A total of 232 PAS cases and 464 matched controls were included (Table 1). In addition to PAS, non‐married status, public insurance, chronic hypertension, hypertensive disorder of pregnancy (HDP), NICU admission, and pre‐existing depression/anxiety were associated with increased odds of PPD. PAS remained an independent risk factor for PPD (aOR 3.10, 95% CI 1.56–6.16) along with preexisting diagnosis of anxiety/depression, language and HDP in a multivariate analysis (Table 2). Among PAS patients, neither hysterectomy nor timing of PAS diagnosis were associated with PPD.

**Conclusion**: PAS is independently associated with 3‐fold higher odds of PPD, even after controlling for pre‐existing mental health disorders. This seems to be unrelated to hysterectomy status or timing of PAS diagnosis. Patients treated for PAS should be offered referral to mental health services and monitored closely for development of PPD.

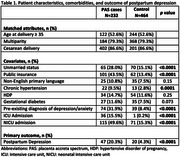

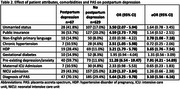

**10:30 AM – 12:00 PM**

## 139 | From Documentation to Communication: Leveraging Generative Artificial Intelligence to Empower High‐Risk Pregnant Individuals

Avihu Krieger^1^; Michal Axelrod^2^; Uri Shemesh^3^; Liron Seidman^4^; Roni Plaschkes^5^; Daphna Komem^1^; Shlomi Toussia‐Cohen^6^; Keren Ofir^2^; Boaz Weisz^1^; Ronen Fluss^6^; Shalom Mazaki‐Tovi^7^; Michal Fishel Bartal^8^; Rakefet Yoeli‐Ullman^1^; Abraham Tsur^6^

^1^The Department of Obstetrics and Gynecology, The Chaim Sheba Medical Center, Ramat‐Gan, Israel, Ramat Gan, Tel Aviv; ^2^Sheba Medical Center, Sheba Medical Center, HaMerkaz; ^3^The Department of Obstetrics and Gynecology, The Chaim Sheba Medical Center, Ramat‐Gan, Israel, Tel Aviv, Tel Aviv; ^4^Lis Hospital for Women's Health, Tel Aviv Sourasky Medical Center Gray Faculty of Medicine, Tel Aviv University, Israel, Ramat Gan, HaMerkaz; ^5^Sheba Medical Center, Ramat Gan, HaMerkaz; ^6^The Sheba Medical Center, The Sheba Medical Center, HaMerkaz; ^7^The Department of Obstetrics and Gynecology, The Chaim Sheba Medical Center, Ramat‐Gan, Israel, Ramat Gan, HaMerkaz; ^8^Division of Maternal‐Fetal Medicine, Department of Obstetrics, Gynecology and Reproductive Sciences, Sheba Medical Center, Tel Aviv University, Israel, Ramat Gan, HaMerkaz

**Objective**: Electronic health records (EHRs) of individuals in high‐risk antepartum units contain valuable clinical data. Large language models (LLMs) offer a promising tool for transforming these notes into patient‐facing summaries. Our aim was to identify the optimal prompt for this task via a structured, clinician‐led evaluation, and to conduct an initial real‐world assessment of patients’ interest in such summaries.

**Study Design**: A prospective two‐phase study. In phase one, 10 clinicians evaluated EHR summaries generated for 10 patients using a HIPAA‐compliant ChatGPT‐4.0 platform. Summaries were created using three prompts (Figure 1): Prompt A–minimal instructions; Prompt B–detailed summarization guidance; and Prompt C–Prompt B plus integration of verified external medical knowledge. Summaries were rated using a validated questionnaire assessing correctness, completeness, conciseness, and safety. We analyzed prompt ratings using linear regression, adjusting for physician, patient, and presentation order. In phase two, 11 summaries were generated with the selected prompt, reviewed them for safety, and shared with patients, who rated their interest and provided feedback.

**Results**: Prompt A, carrying minimal instructions, received the highest overall score, ranking significantly higher than Prompt B (*p* = 0.024) and trending higher than Prompt C (*p* = 0.053) (Figure 2), and was selected to generate 11 summaries for the next step.

In phase two, we generated summaries for 11 patients based on their EHRs, excluding one for safety concerns after incorrectly implying completion of a genetic workup that was only partial. The remaining 10 summaries (90.9%) were delivered in patients’ native languages (9 Hebrew, 1 Arabic). Overall, 80% of patients were interested in additional summaries, but only 40% reported improved understanding.

**Conclusion**: Most high‐risk antepartum patients expressed interest in continuing to receive LLM‐generated summaries of their EHRs after initial exposure. While this approach shows promise for empowering and engaging our patients, clinician review remains essential to ensure safety.

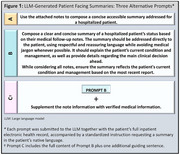

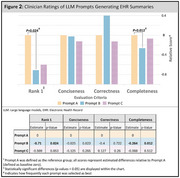

**10:30 AM – 12:00 PM**

## 140 | An Agentic AI Virtual Assistant for Gestational Diabetes: Patient Engagement in the First Two Months

Avish Arora^1^; Pe'er Dar^2^; Georgios Doulaveris^2^

^1^Montefiore/Albert Einstein College of Med., New York, NY; ^2^Montefiore Medical Center/Albert Einstein College of Medicine, Montefiore Medical Center/Albert Einstein College of Medicine, NY

**Objective**: To quantify early patient engagement with an agentic AI virtual assistant for GDM.

**Study Design**: Gestational diabetes mellitus (GDM) demands daily glucose checks, dietary adjustment, and timely nutritional guidance, but adherence often declines and educator capacity may not meet demand. We developed a patient‐facing, agentic AI tool for GDM that received FDA approval as an investigational device to support self‐management. The WhatsApp‐based AI assistant coordinates six specialized agents (Glucose Logging, Glucose Analysis, Nutritional Advice, Daily Check‐in, Vision, Physician Handoff). Functions include structured capture of patient‐entered values, trend‐based meal guidance, symptom triage, and automated physician handoffs. A secure dashboard allows clinicians to review real‐time logs and AI‐flagged alerts at‐a‐glance. Daily operations include a 09:00 am glucose summary and a supportive nudge after 22 h of disengagement (quiet hour rules enforced). Ten pregnant individuals with diet‐controlled GDM enrolled 1 June–30 July 2025. Engagement metrics were extracted from every patient message; glucose targets were <90 mg/dL fasting and <120 mg/dL post meal.

**Results**: Over 60 days, the assistant exchanged 2170 total messages; 710 (33 %) originated from patients. Participants texted the coach on a median 17 separate days (range 1–59) and sent a median 3 messages per engaged day. At least one glucose value was logged on a median 24 days per patient, and 67% of those days contained ≥2 readings. Across 411 captured values, 71% met pregnancy targets. Two symptom texts (“nauseous”, “dizzy”) triggered clinician callbacks within 15 min; no hypoglycemia alerts were missed and no manual overrides were required.

**Conclusion**: The agentic AI GDM Assistant sustained meaningful patient interaction and regular glucose logging during the critical first two months after GDM diagnosis, while safely triaging symptoms without added clinician workload. A randomized controlled trial comparing traditional nutritional counseling with agentic AI–based diabetes management is underway.

**10:30 AM – 12:00 PM**

## 141 | Streaming Labor: What 28,436 YouTube Videos Reveal About Childbirth Content

Ayala Hirsch^1^; Chen Mordehai^2^; Mika Rabinovich^2^; Adi Y. Y.Weintraub^3^; Reut Rotem^4^

^1^Shaare Zedek medical hospital, Jerusalem, Yerushalayim; ^2^Ben‐Gurion University of the Negev, Beer Sheva, HaDarom; ^3^Soroka University Medical Center, Faculty of Health Sciences, Ben‐Gurion University of the Negev, beer sheba, HaDarom; ^4^Cork University Maternity Hospital, Cork, Cork

**Objective**: This study aimed to evaluate childbirth‐related YouTube videos at scale using a Big Data approach, analyzing their emotional tone, classifying them according to content type (e.g., medical recommendations, personal experiences), and thematic content.

**Study Design**: Using 446 unique childbirth‐related search queries, we scraped 28,436 publicly available YouTube videos. We downloaded the audio track for each video, then transcribed it automatically. Metadata, transcripts, and thumbnails were analyzed by integrating multiple natural language processing (NLP) classifiers, topic modeling approaches, and image‐captioning pipelines. This framework enabled sentiment analysis (TextBlob), content classification and medical recommendation assessment (T5 model), topic modeling (LDA), thumbnail image analysis (gpt2), and medication extraction (Med7). Descriptive statistics (frequencies, percentages, medians, interquartile ranges) were used to summarize video characteristics, sentiment distribution, and classification outputs.

**Results**: Short‐form videos (<10 minutes) made up 63.3% of the dataset. Of 5402 videos containing medical recommendations, the vast majority (96%) were classified as balanced. Sentiment analysis revealed that 88% of transcripts were positive, 7.7% negative, and 4.3% neutral. Thumbnails showed a shift toward neutral‐to‐negative sentiment. The most frequently mentioned medications were opioids (e.g., fentanyl, morphine), oxytocin, and antibiotics. “Can you refuse a C‐section” was the most viewed hashtag, reflecting high public interest in maternal autonomy.

**Conclusion**: Childbirth‐related YouTube content serves both emotional and educational roles. While much clinical content is balanced in presenting both positive and negative aspects, its framing is shaped by storytelling and platform aesthetics. Engaging with digital platforms offers healthcare providers an opportunity to improve online health literacy and ensure access to trustworthy, relatable information.

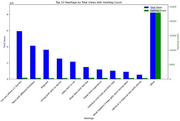

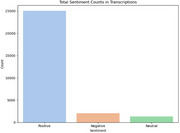

**10:30 AM – 12:00 PM**

## 142 | Effects of Obesity on Surgical Outcomes in Cases of Placenta Accreta Spectrum

Benjamin Gastevich^1^; Rodney A. McLaren, Jr^2^; Tirtza Spiegel Strauss^2^; Emily Schlussel Markovic^2^; Joselle O'Brien^2^; Henri M. Rosenberg^3^; Alexa L. Cohen^4^; Georgios Doulaveris^5^; Rebecca H. Jessel^6^; Meghana A. Limaye^7^; Lois Brustman^8^; Pe'er Dar^5^; Angela T. Bianco^9^; Shoshana Haberman^2^; Scott Chudnoff^2^; Itamar Futterman^1^

^1^Maimonides Medical Center, Maimonides Health, NY; ^2^Maimonides Medical Center, Brooklyn, NY; ^3^Icahn School of Medicine at Mount Sinai, Icahn School of Medicine at Mount Sinai, NY; ^4^University of Miami, Miami, FL; ^5^Montefiore Medical Center/Albert Einstein College of Medicine, Montefiore Medical Center/Albert Einstein College of Medicine, NY; ^6^Division of Maternal‐Fetal Medicine, Department of Obstetrics and Gynecology, NYU Grossman School of Medicine, New York, NY; ^7^Division of Maternal‐Fetal Medicine, Department of Obstetrics and Gynecology, NYU Grossman School of Medicine, NYU Langone, NY; ^8^Icahn School of Medicine, Mount Sinai West, New York, NY; ^9^Icahn School of Medicine at Mount Sinai, New York, NY

**Objective**: We set to evaluate the effects of maternal obesity on surgical morbidity outcomes in cases of placenta accreta spectrum (PAS).

**Study Design**: This was a multicenter retrospective cohort study of histopathologically confirmed PAS cases 1/1/2013–6/30/2022 at five New York academic institutions. Maternal obstetrical and demographic variables were collected. The cohort was divided into two groups, obese (body mass index (BMI) ≥30 kg/m^2^) and non‐obese (BMI < 30 kg/m^2^). The primary outcome was a composite of PAS related surgical outcomes, including intensive care unit (ICU) admission, vasopressors use, intubation, blood loss, organ injury, and transfusion. Univariable analysis compared variables using chi square test for categorical and Mann Whitney U/student t for continuous variables. A logistic regression analysis was then performed adjusting for statistically significant variables between the two groups.

**Results**: Of the 401, 377 cases had documented maternal pre‐pregnancy BMI. There were 159 obese and 218 non‐obese patients. Obese patients were more likely to have a vertical skin incision, less likely to have undergone artificial reproductive technology, present later in the pregnancy for prenatal care and more likely to have been diagnosed with PAS antenatally (55.6% vs. 36.8%, *p* < 0.001, 20.2% vs. 5.1%, *p* < 0.001, 12 (9, 16) vs. 10 (8, 13) *p* = 0.004, 98.7% vs.91.2%, *p* = 0.002). After adjusting for significant variables, aside from an increased risk of intubation in the obese group (aOR = 1.82 with 95% CI 1.09–3.03), surgical morbidity composite did not differ between obese and non‐obese patients (aOR 0.39, 95% CI 0.11–1.37).

**Conclusion**: Obese and non‐obese patients had similar odds of surgical morbidity for most outcomes. Our findings suggest the impact of obesity on PAS surgical morbidity may be more nuanced and that the interplay between obesity and PAS outcomes deserves further investigation.

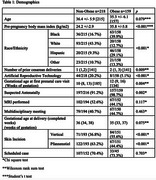

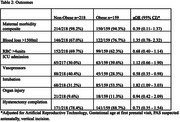

**10:30 AM – 12:00 PM**

## 143 | Maternal Sepsis And Timely Antibiotic Administration: A Quality Improvement Initiative

Beverly C. Tse; Andrea D. Shields; Kathleen Zacherl

University of Connecticut Health, Farmington, CT

**Objective**: Maternal sepsis remains a major cause of pregnancy‐related morbidity and mortality, with timely diagnosis and evidence‐based treatment critical for improved outcomes. The California Maternal Quality Care Collaborative (CMQCC) has developed a validated maternal sepsis screen. Following a positive screen, antibiotic administration within one hour is recommended, even while awaiting confirmatory evaluation. This quality improvement (QI) initiative assessed baseline adherence to the one‐hour antibiotic goal and identified system‐level gaps, with a target of 80% compliance.

**Study Design**: We conducted a retrospective chart review of pregnant and postpartum patients who screened positive for maternal sepsis with suspected infection at an academic medical center. The primary measure was the proportion of patients receiving antibiotics within one hour of screening positive. Outcome measures included ICU admission and confirmed sepsis cases. QI tools, including a Pareto chart and a key driver diagram, were used to assess root causes and guide future interventions.

**Results**: Among 84 patients who screened positive, 56 (66%) patients had suspected infections and received antibiotics. Of those treated with antibiotics, only 32 (57%) received them within the recommended one‐hour timeframe. Compliance fluctuated between 33–86% during the study period, with an initial decline followed by improvement after an education intervention in 7/2024 (Figure 1). Adjusting for five cases in which providers waited for a second fever to diagnose intraamniotic infection, the adjusted one‐hour compliance rate was 52%. Only 32% of patients with confirmed sepsis and 28% of those requiring ICU admission received antibiotics within one hour. Figure 2 demonstrates the causes of delays.

**Conclusion**: Our findings highlight suboptimal adherence to the one‐hour antibiotic target, with only 57% compliance overall and low compliance in patients with confirmed sepsis or ICU admission. Provider education alone was insufficient; future efforts will target systemic interventions and support to improve timely antibiotic delivery for maternal sepsis.

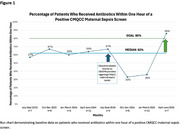

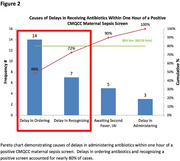

**10:30 AM – 12:00 PM**

## 144 | Prenatal Vaccination Rates Post‐Pandemic in Pregnant Persons With and Without Medical Co‐Morbidities

Bhavana Janga^1^; Judy Jiang^2^; Hannah Hill^3^; Hansika Kunduru^4^; Kelly S. Gibson^5^

^1^MetroHealth Medical Center / Case Western Reserve University, Cleveland, OH; ^2^Case Western Reserve University/MetroHealth Medical Center, Cleveland, OH; ^3^MetroHealth Medical Center, The MetroHealth System, OH; ^4^Case Western Reserve University, Cleveland, OH; ^5^MetroHealth Medical Center/ Case Western Reserve University, Cleveland, OH

**Objective**: To evaluate how the coronavirus disease 2019 (COVID‐19) pandemic affected influenza, tetanus toxoid, reduced diphtheria toxoid and acellular pertussis (TDaP), and COVID‐19 vaccination rates in a pregnant population with medical co‐morbidities.

**Study Design**: Retrospective cohort study utilizing the EPIC COSMOS database to evaluate influenza, TDaP, and COVID‐19 vaccination rates in pregnant patients who had a hypertensive disorder of pregnancy, asthma, or pregestational/gestational diabetes mellitus (DM) from 2016 to 2024. Multivariable logistic regression modeling was conducted to evaluate the effect of time and factors of interest on vaccine rates.

**Results**: There were 23,118,213 patients eligible for vaccination during pregnancy, 5,852,492 of whom had hypertensive disorders of pregnancy, asthma, or pregestational/gestational DM. The high‐risk group had significantly higher vaccination rates across all three vaccines compared to the general population (*p* < 0.001). There was an increase in influenza and TDaP vaccination rates until 2020, after which there was a decrease in vaccination rates almost to pre‐pandemic levels (*p* < 0.001). Peak COVID‐19 vaccination rates in the high‐risk group occurred in 2021 at 16.5% followed by a decline down to 0.05% by 2024. These were significantly higher than rates in the general pregnant population of 14.4% and 0.04%, respectively (*p* < 0.001). TDaP vaccination rates were significantly higher than influenza vaccination rates at peak uptake, 52.2% vs 29% respectively (*p* < 0.001).

**Conclusion**: Pregnant patients with medical co‐morbidities had higher vaccination rates than low‐risk pregnancies. Influenza and TDaP vaccination rates increased until the COVID‐19 pandemic began in 2020 and then declined. COVID‐19 vaccination rates peaked in 2021 followed by a significant decline. This decline in all three vaccination rates highlights the importance of clear, evidence‐based guidance, particularly for pregnant patients at high risk for disease.

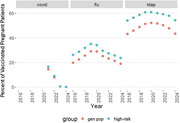

**10:30 AM – 12:00 PM**

## 145 | Carrier Screening as a Pathway to In‐Utero Therapy

Billie R. Lianoglou^1^; Elizabeth Repass^2^; Eugenia Martinez Jaime^2^; Ashley Cantu‐Weinstein^2^; Vivienne Souter^2^; Amber Shamburger^2^; Jeffrey Dungan^2^; Irene J. Chang^3^; Priyanka Arya^2^; Tippi C. MacKenzie^3^

^1^University of California, San Francisco, San Francisco, CA; ^2^Natera, Inc., Austin, TX; ^3^University of California San Francisco, San Francisco, CA

**Objective**: Prenatal interventions for genetic conditions are rapidly expanding (such as in‐utero transfusions for anemias or in‐utero enzyme replacement therapy for lysosomal storage disorders). This study aims to evaluate how reproductive carrier screening can identify pregnancies at risk for genetic conditions in which in‐utero (IU) therapy may be an option.

**Study Design**: We conducted a retrospective study of carrier screening results from women aged 18–55 years at a US‐based commercial lab (Jan 2021‐Aug 2024). Thirteen genes were selected based on their association with conditions for which IU therapies are in clinical use or actively enrolling trials (*n* = 10), or those described in published case reports or investigational therapies (*n* = 3). We calculated screening rates and carrier frequencies for women tested for all 13 conditions of interest. Frequencies were also stratified by self‐reported race and ethnicity.

**Results**: Of the screened population (*N* = 1,077,487), 97% were screened for at least one gene of interest, most frequently *SMN1, CFTR*, and *HBA1/2*. Among those tested for all 13 conditions (*N* = 117,870, 10.9% of screened population), the aggregate carrier frequency for conditions with established IU therapy or trials was 1 in 24 (4.3%); for investigational therapies, 1 in 16 (6.5%). While individual gene frequencies vary by race, the aggregate probability of carrying any of the conditions with the potential for IU therapy is consistently high across racial and ethnic groups.

**Conclusion**: Carrier screening can identify pregnancies at risk for conditions with available or emerging IU therapies. Many of the conditions are rare individually but relatively common in aggregate. Incorporating clinical actionability into screening frameworks, beyond population prevalence alone, may expand equitable access to IU therapy and optimize fetal outcomes. Patient education regarding options for IU therapy may also be valuable in decision‐making to pursue carrier screening.

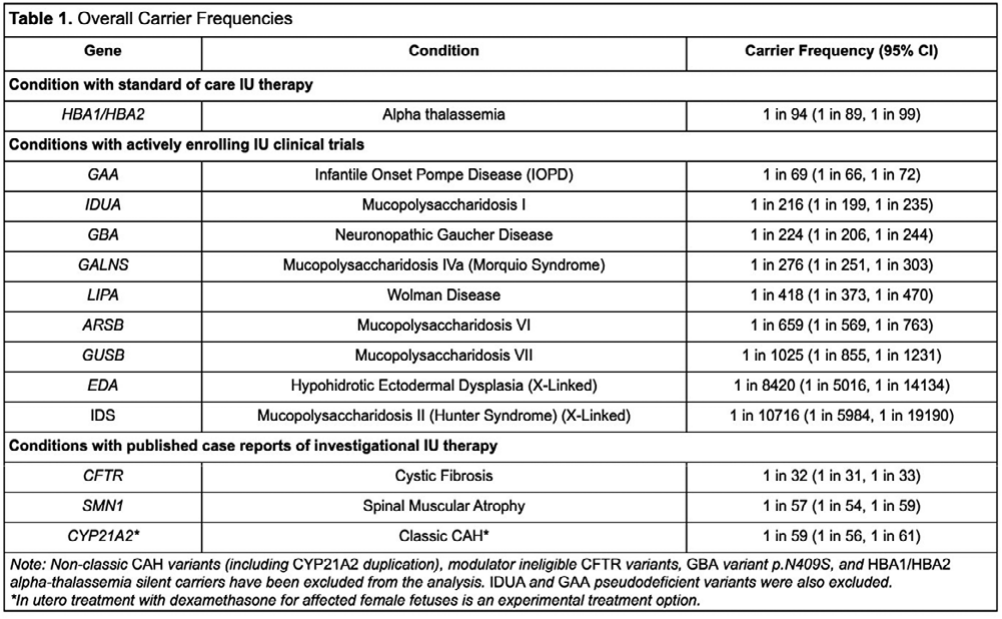

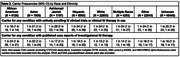

**10:30 AM – 12:00 PM**

## 146 | Maternal and Neonatal Outcomes After Fetoscopic Prenatal Versus Postnatal Repair of Open Neural Tube Defects

Bree A. Goodman^1^; Jagruti Anadkat^2^; Jennifer M. Strahle^1^; Jesse Vrecenak^2^; Sarah Smith^3^; Nandini Raghuraman^1^; Jeannie C. Kelly^3^; Katherine H. Bligard^2^

^1^Washington University School of Medicine, St. Louis, MO; ^2^Washington University School of Medicine, Saint Louis, MO; ^3^Washington University School of Medicine, Washington University School of Medicine, MO

**Objective**: Prenatal repair of open neural tube defects (ONTDs) via hysterotomy offers significant fetal benefits but is highly invasive and poses maternal risks, influencing the choice between prenatal and postnatal repair. Fetoscopic repair is a promising alternative to open prenatal surgery, aiming to provide similar fetal neurological benefits with reduced maternal morbidity and the potential for vaginal delivery (VD). We evaluated maternal and neonatal outcomes for fetoscopic vs. postnatal repair.

**Study Design**: This is a retrospective cohort study of fetal ONTDs referred to a single fetal center from 2020 to 2024. Demographic, surgical, and outcome data were collected and compared between patients who chose fetoscopic prenatal repair versus postnatal repair. Primary outcomes included VD rate and need for CSF diversion in the first year. Secondary outcomes included postpartum blood loss, hospital encounters, surgery length for cesarean delivery (CD), severe maternal morbidity (SMM), preterm delivery rates, and NICU length of stay (LOS).

**Results**: During the study period fetoscopic surgery was performed in 12 patients and 29 opted for postnatal repair. Maternal and fetal characteristics were similar, with the exception of a higher rate of myeloschisis in the fetoscopic repair group (Table 1). Vaginal delivery (VD) rates were similar between groups (66.7% for fetoscopic repair vs. 35.7% for postnatal repair, *p* = .09). For those who ultimately underwent cesarean delivery (CD), the length of procedure was significantly longer in patients who underwent fetoscopic prenatal repair (*p* = 0.02), however there was no difference in other measures of maternal morbidity. With regards to neonatal outcomes, there was a lower rate of CSF diversion in the prenatal repair group (40.0% vs. 78.3%, *p* = 0.03). These patients also delivered at an earlier gestational age (35.7 vs. 39.0 weeks, *p* = 0.001), however NICU LOS was similar between groups (Table 2).

**Conclusion**: Fetoscopic repair of ONTDs is associated with improved fetal neurologic outcomes compared to postnatal repair and high rates of vaginal delivery.

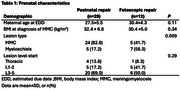

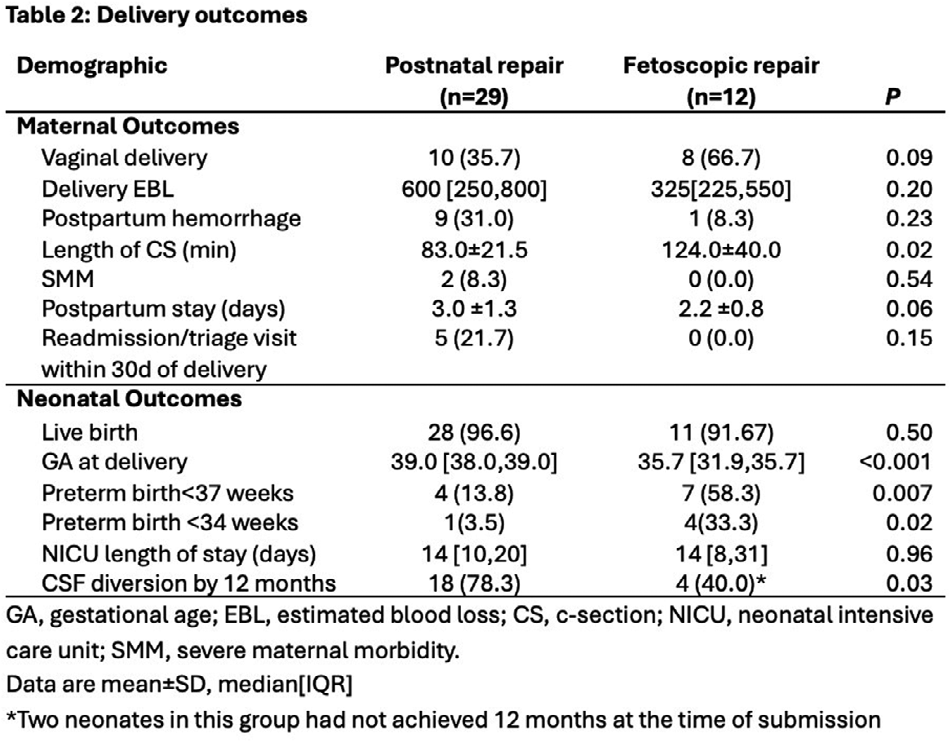

**10:30 AM – 12:00 PM**

## 147 | Laser Energy and Perinatal Outcomes Following Fetoscopic Laser Photocoagulation for Twin‐to‐Twin Transfusion Syndrome

Brian A. Burnett^1^; Kamran Hessami^1^; Rebecca M. Johnson^1^; Allie M. Sidwell^2^; Jessian L. Munoz^3^; Cara Buskmiller^4^; Roopali V. Donepudi^5^; Magdalena Sanz Cortes^5^; Michael A. Belfort^1^; Ahmed A. Nassr^5^

^1^Baylor College of Medicine, Houston, TX; ^2^University of Illinois College of Medicine, Chicago, IL; ^3^Texas Children's Hospital, Texas Children's Hospital, TX; ^4^Baylor College of Medicine, Austin, TX; ^5^Baylor College of Medicine, Baylor College of Medicine, TX

**Objective**: Twin‐to‐twin transfusion syndrome (TTTS) affects monochorionic twin pregnancies and is treated with fetoscopic laser photocoagulation (FLP), the standard of care. However, FLP may be associated with adverse perinatal outcomes. We aimed to evaluate the association between the amount of laser energy used during FLP and perinatal outcomes in TTTS.

**Study Design**: This retrospective cohort study included monochorionic twin pregnancies undergoing FLP for TTTS at a single tertiary center between 2012 and 2024. Total laser energy was analyzed as a continuous variable and dichotomized at 5100 J based on optimal cut‐point via Youden's index. Outcomes included placental abruption, chorioamniotic membrane separation (CAS), preterm premature rupture of membranes (pPROM), and preterm delivery ≤32 and ≤34 weeks. Bivariate analyses compared outcomes by energy groups. Multivariable logistic regression evaluated the association between increasing laser energy (per 100 J increments) and preterm delivery ≤34 weeks while adjusting for preoperative cervical length, gestational age at surgery, postoperative CAS, pPROM, and septostomy.

**Results**: Of 496 patients undergoing laser surgery, 245 received <5100 J and 251 received ≥ 5100 J of laser energy. The higher energy group had a later gestational age at surgery (20.9 vs. 19.4 weeks, *p* < 0.001) and earlier delivery (30.4 vs. 31.3 weeks, *p* = 0.02) (Table 1). Deliveries ≤32 and ≤34 weeks were significantly associated with higher energy use (Table 2). No significant differences were observed between energy groups in rates of CAS, pPROM, or placental abruption. In adjusted multivariate analysis, each 100 J increase in energy was associated with 1% increased odds of delivery ≤ 34 weeks (aOR 1.01, 95% CI 1.00–1.02; *p* = 0.02).

**Conclusion**: Higher laser energy use during TTTS surgery was associated with later gestational age at surgery and with earlier delivery. Each 100 J increase in laser energy independently increased the risk of preterm delivery at ≤34 weeks by 1%. Procedural energy use may represent a modifiable factor influencing latency and warrants further investigation.

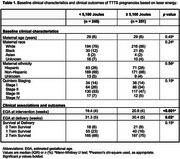

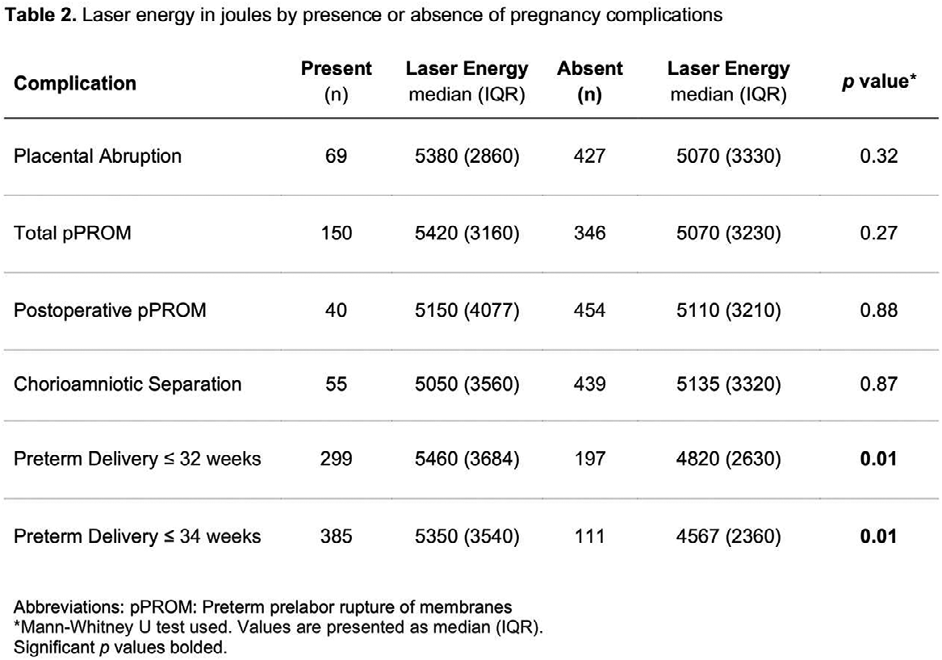

**10:30 AM – 12:00 PM**

## 148 | Delivery Outcomes Among Patients With Neurofibromatosis in the United States 2016–2022

Brittany Arditi^1^; Caitlin D. Baptiste^2^; Marie‐Julie Trahan^1^; Timothy Wen^3^

^1^Columbia University Irving Medical Center, New York, NY; ^2^Division of Maternal‐Fetal Medicine, Department of Obstetrics and Gynecology, Columbia University Irving Medical Center, New York, NY; ^3^UCSD, Irvine, CA

**Objective**: Much of the recent literature regarding neurofibromatosis in pregnancy is limited to case reports and small series. Our objective was to characterize current maternal and obstetric outcomes in pregnancies complicated by neurofibromatosis type 1 (NF1) using a national database.

**Study Design**: We conducted a cross‐sectional study using the U.S. National Inpatient Sample to identify delivery hospitalizations among patients with NF1 between 2016 and 2022. Maternal and obstetric outcomes were compared between patients with and without NF1 using adjusted logistic regression models accounting for demographic, clinical, and hospital characteristics. Associations were reported as adjusted odds ratios (aORs) with 95% confidence intervals (CIs).

**Results**: Among more than 25 million delivery hospitalizations, 3515 involved patients with NF1 at a rate of 13.99 per 100,00 deliveries. The prevalence of NF1‐affected deliveries remained stable over the study period [Figure 1]. Compared to patients without NF1, those with NF1 had significantly higher odds of severe maternal morbidity (aOR 1.91, 95% CI 1.05–3.47), hypertensive disorders of pregnancy (aOR 1.49, 95% CI 1.23–1.81), cesarean delivery (aOR 2.01, 95% CI 1.72–2.33), fetal growth restriction (aOR 2.87, 95% CI 2.26–3.65), and preterm birth (aOR 1.96, 95% CI 1.60–2.39). The greatest risk was seen for very preterm birth before 28 weeks (aOR 2.81, 95% CI 1.68–4.72). There was no significant increase in the risk of intrauterine fetal demise (aOR 1.64, 95% CI 0.74–3.63) or cerebrovascular complications [Table 1].

**Conclusion**: The rate of deliveries affected by NF1 did not change significantly over time. Patients with NF1 are at increased risk for a broad range of adverse obstetric outcomes. Further research is needed to determine what clinical factors potentiate this risk.

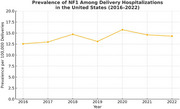

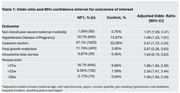

**10:30 AM – 12:00 PM**

## 149 | Shifting Power Dynamics: Can Enhanced Prenatal Care Models Reduce Experiences of Race‐ and Ethnic‐Based Discrimination?

Brittany D. Chambers Butcher^1^; Jessica Johnston^1^; Patience A. Afulani^2^; Bridgette Blebu^3^; Kristin Carraway^4^; Kimberly Coleman‐Phox^5^; Jennifer N. Felder^6^; Mary A. Garza^4^; Deborah Karasek^7^; Daisy León‐Martínez^8^; Charles E. McCulloch^9^; Venise Curry^4^; Miriam Kuppermann^10^

^1^Department of Human Ecology, College of Agricultural and Environmental Sciences, University of California, Davis, Davis, CA; ^2^Department of Obstetrics, Gynecology and Reproductive Sciences, University of California, San Francisco, Universiy of California San Francisco, CA; ^3^Lundquist Institute for Biomedical Innovation ‐ Harbor‐UCLA, Los Angeles, CA; ^4^Central Valley Health Policy Institute at California State University, Fresno, Fresno, CA; ^5^Department of Obstetrics, Gynecology and Reproductive Sciences, University of California, San Francisco, San Francisco, CA; ^6^Department of Psychiatry and Behavioral Sciences, University of California, San Francisco, San Francisco, CA; ^7^Oregon Health & Science University‐Portland State University School of Public Health, Portland, OR; ^8^Division of Maternal‐Fetal Medicine at the University of California San Francisco, University of California San Francisco, CA; ^9^University of California, San Francisco, University of California San Francisco, CA; ^10^Department of Obstetrics, Gynecology and Reproductive Sciences, University of California, San Francisco, University of California San Francisco, CA

**Objective**: To determine whether participation in enhanced group prenatal care (eGPC) reduces experiences of discrimination.

**Study Design**: The EMBRACE (Engaging Mothers & Babies; Reimagining Antenatal Care for Everyone) Study is a pragmatic two‐armed randomized comparative effectiveness trial of the effect of eGPC versus enhanced Individual prenatal care (eIPC) on depression, patient care experiences, and preterm birth, among Medi‐Cal eligible pregnant people in California. The primary outcome of this analysis is self‐reported experiences of discrimination based on race/ethnicity/color as measured by the 9‐item Discrimination in Medical Settings (DMS) scale (scores ranging 0–4). We employed intent‐to‐treat principles (ITT) for the primary analysis and secondary analyses based on eGPC and eIPC attendance (>1 visit;4 visits) and Black and Latine racial/ethnic identity (*n* = 554). Qualitatively trained staff and community members gathered and analyzed qualitative data to support ITT findings among a subsample of 52 Black and Latine participants through audio‐recorded and professionally transcribed interviews.

**Results**: Over 84% of participants identified as Latine. Participants assigned to eGPC (0.97/4) vs. eIPC (1.1 /4) reported similar DMS scores; however, Black participants (2.1/4) reported higher DMS scores than Latine participants (0.92/4) during prenatal care. Adjusting for language, education, prior pregnancy complications, and prior experiences of DMS, there were no significant differences in occurrences of DMS during prenatal care between eGPC vs. eIPC [aMR: 0.01; CI: −0.37,0.39]. Subgroup analysis suggests eGPC maybe protective for Latine participants. Qualitative data provide insights on potential drivers for experiences of DMS, for example: *Patient's autonomy being overshadowed by provider's judgement and inattentiveness*.

**Conclusion**: Findings underscore the lingering effects of institutional racism impacting the quality‐of‐care Black birthing people receive. Given the limited sample of Black birthing people, this study should be replicated with Black‐centered eGPC models.

**10:30 AM – 12:00 PM**

## 150 | Drinking the Kool‐Aid: Misinformation About #Glucola on TikTok

Brooke Schroeder^1^; Sally Kuehn^1^; Joelle Sills^1^; Sarah Dotters‐Katz^1^; Jenny Wu^2^

^1^Duke University School of Medicine, Durham, NC; ^2^Columbia University Medical Center, New York City, NY

**Objective**: TikTok is a popular source of medical information—and misinformation—for pregnant patients. Concerns about artificial dyes and sweeteners have fueled online debate surrounding Glucola, the test for gestational diabetes. We aimed to characterize TikTok content on Glucola.

**Study Design**: After IRB exemption, we evaluated the 100 most‐liked videos tagged #glucola, #glucolatest, #gestationaldiabetestest, and #gdm. Data were extracted via the Apify platform based on validated methods and ranked by number of likes. Videos were excluded if they lacked Glucola content, were non‐English, or had missing audio/video. Two reviewers coded content; discrepancies were resolved by a third. Primary outcome was tone toward Glucola. Secondary outcomes included: misinformation, promotion of alternative testing, and validated scores to assess video reliability(DISCERN) and video understandability and actionality(PEMAT).

**Results**: Of 1017 videos, 444 were excluded due to irrelevance or non‐English language before reaching the top 100 videos. Among the top 100, 98% were by female‐identifying users. Most common content included personal experience with Glucola(*n* = 58), medical education(*n* = 20), and opinion(*n* = 11).

Negative tone toward Glucola appeared in 42% of videos; only 25% were positive. Among patient‐created posts, 48% expressed anxiety about drinking Glucola, 32% complained about taste, and 15% encouraged anxiety in others.

Approximately 30% of videos included partially or completely inaccurate information about Glucola. Of this, 7% of videos claimed the drink contained harmful ingredients (Table 1). Approximately 18% of videos promoted unvalidated alternatives.

Based on the standardized scales, reliability was low, actionability was poor, and understandability was moderate (Table 2).

**Conclusion**: TikTok videos about Glucola frequently include misinformation and promote unvalidated alternatives, fueling concerns and refusal of essential testing. Obstetric providers should be aware of these online narratives and offer anticipatory guidance to address misconceptions and promote evidence‐based care.

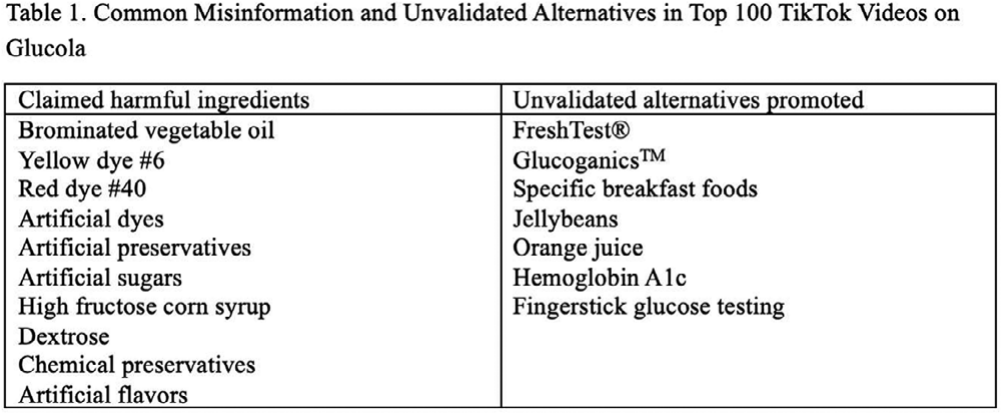

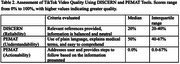

**10:30 AM – 12:00 PM**

## 151 | QAPI Review of MTP Utilization Improves Compliance and Maternal Outcomes

Bryce T. Munter^1^; Ghamar Bitar^1^; Aaron W. Roberts^2^

^1^Division of Maternal‐Fetal Medicine, Department of Obstetrics, Gynecology, and Reproductive Sciences, McGovern Medical School at UT Health Houston, Houston, TX; ^2^Department of Obstetrics, Gynecology and Reproductive Sciences, McGovern Medical School at UTHealth Houston, Houston, TX

**Objective**: Early mobilization of resources via use of a massive postpartum hemorrhage (PPH) protocol has been shown to improve maternal morbidity and mortality. However, early mobilization can be challenging in cases of unanticipated PPH, leading to unexpectedly high blood loss and delays in resuscitation. Our objective was to identify if focused QAPI‐led interventions affected 1) mobilization of resources in settings of massive hemorrhage by using MTP utilization as a marker, and 2) incidence of maternal morbidity, using hemorrhage‐related ICU admission as a marker.

**Study Design**: Our level IV maternal care center massive hemorrhage protocol dictates that MTP should be activated in all patients who have blood loss >1500mL AND either 1) have concern for ongoing bleeding, 2) are en route to the OR, or 3) have hemodynamic instability. Our QAPI team, a multidisciplinary collaborative group comprised of physician and nursing leadership, meets once per month to review variances and pre‐specified obstetric metrics. Activation of MTP protocol was added as a review metric in 10/2024. After QAPI variance review, focused education and feedback was provided using closed‐loop communication to all teams involved in patient care. Compliance rate and hemorrhage‐related ICU admission rate were calculated from 10/2024 to 5/2025. Patients with known placenta accreta spectrum disorders were excluded, given our goal to focus on unanticipated massive hemorrhage.

**Results**: Our QAPI team met 8 times during our study period. Since MTP utilization was added as a metric for review, 82 patients met protocol criteria and MTP was performed for 17 patients (21%). Month‐by‐month focused feedback and education by the QAPI team increased rate of compliance by 17% (95% CI 13.9–36.1%) over 8 months. In the same period, hemorrhage‐related ICU admissions decreased by 22% (95% CI 19.4–46.2%).

**Conclusion**: QAPI‐led interventions increased early resuscitation efforts by 17% and decreased ICU admissions by 22% over 8 months. These findings highlight the importance of maternal quality programs as part of an overall strategy to reduce maternal morbidity.

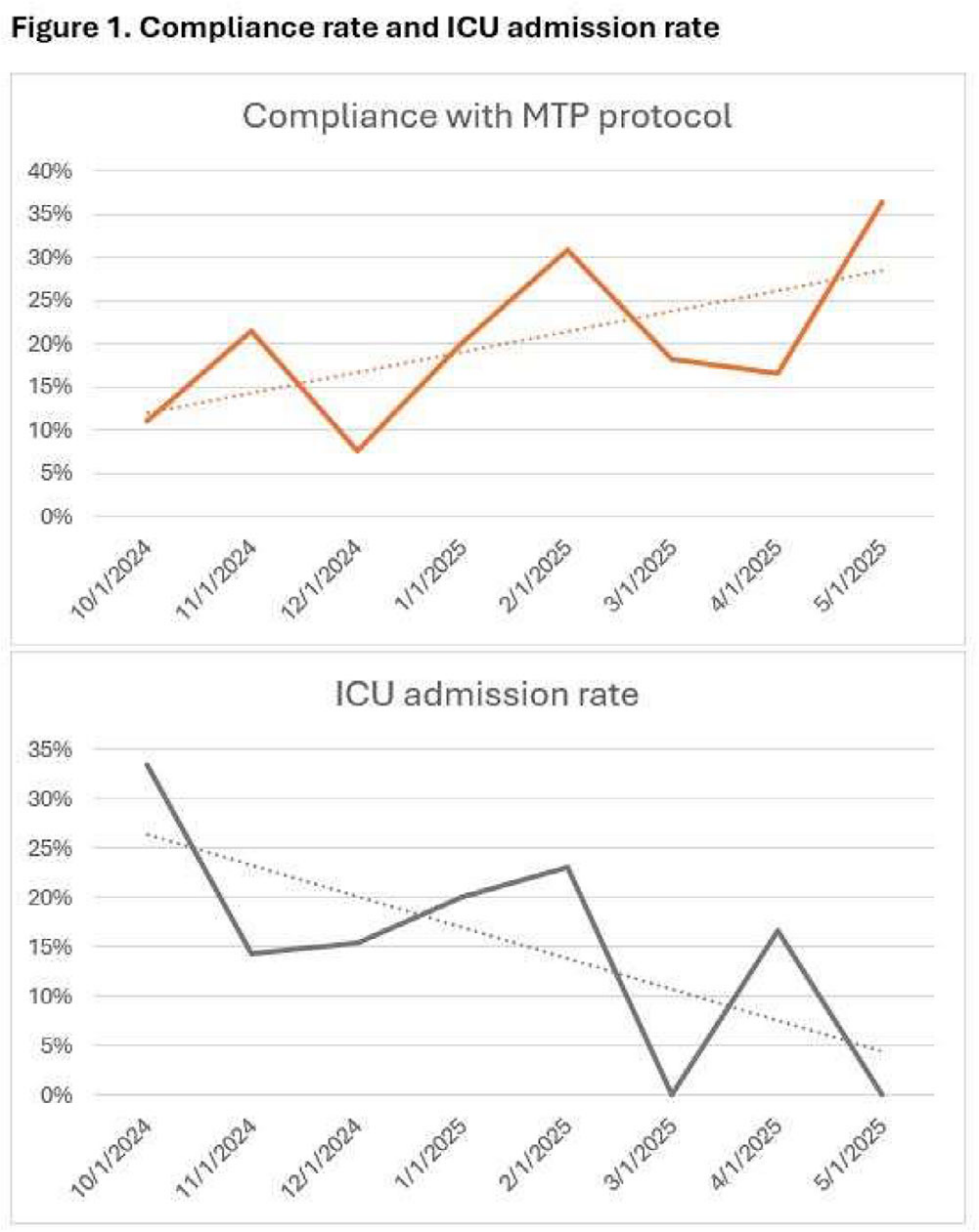

**10:30 AM – 12:00 PM**

## 152 | Prophylactic Selective Embolization of Pelvic Arteries for Invasive Placenta: A Prospective Cohort Study

Jordan A. McKinney^1^; Caitlin Rogers^1^; Erica Smith^2^; Callie F. Reeder^3^; Michael Lazarowicz^1^; Mehmet Genc^1^

^1^University of Florida College of Medicine, Gainesville, FL; ^2^Intermountain Health, Billings, MT; ^3^UTHSC UTGSM Knoxville, Knoxville, TN

**Objective**: Invasive placenta accreta spectrum (PAS) is associated with high maternal morbidity, particularly from hemorrhage during cesarean hysterectomy. This study aimed to evaluate whether prophylactic selective embolization of pelvic arteries (SEPA) reduces intraoperative blood loss and transfusion requirements in patients with invasive PAS.

**Study Design**: We conducted a prospective cohort study of 42 patients with histologically confirmed invasive PAS (grades 2–3) who underwent cesarean hysterectomy at a tertiary referral center from January 2017 to December 2023. Seventeen patients received prophylactic SEPA following cesarean delivery and prior to hysterectomy; 25 did not. Demographic, clinical, and surgical characteristics, as well as maternal and neonatal outcomes, were compared. The primary outcome was intraoperative blood loss. Continuous variables were compared using unpaired t‐tests or Mann‐Whitney U tests, and categorical variables with chi‐square or Fisher's exact tests. A planned multiple linear regression model adjusted for baseline differences.

**Results**: The SEPA and non‐SEPA groups were similar in most baseline characteristics, although SEPA recipients had lower parity and more frequent MRI use; neither variable was significantly associated with blood loss in adjusted models. SEPA was associated with a 48% reduction in the risk of blood component replacement (95% CI, 0.26–0.90; *p* = 0.02), lower median intraoperative blood loss (1,500 mL vs. 2000 mL; *p* = 0.02), and fewer transfused packed red blood cell units (0 vs. 2; *p* = 0.04). Other maternal and neonatal outcomes were similar.

**Conclusion**: Prophylactic SEPA significantly reduced intraoperative blood loss and transfusion needs during cesarean hysterectomy for invasive PAS, supporting its use as an adjunct in surgical management at experienced centers.

**10:30 AM – 12:00 PM**

## 153 | Utility of Expanded Carrier Screening for Identifying Risk of Inherited Genetic Disease in Anomalous Fetuses

Carmen M.A. Santoli^1^; Billie R. Lianoglou^2^; Nuriye Sahin‐Hodoglugil^2^; Katie Tick^2^; Rozlyn C.T Boutin^2^; Kate Swanson^2^; Patrick Devine^2^; Jessica Van Ziffle^2^; Mary E. Norton^2^; Teresa N. Sparks^2^

^1^University of California, San Francisco, University of California, San Francisco, CA; ^2^University of California, San Francisco, San Francisco, CA

**Objective**: Expanded carrier screening (ECS) panels are designed to identify reproductive risk for inherited autosomal recessive (AR), and in some cases, X‐linked (XL) conditions. However, many fetal genetic diseases are sporadic or inherited through other mechanisms. We aimed to determine the proportion of all fetal genetic diseases, as well as the proportion of inherited AR and XL conditions, detectable by ECS.

**Study Design**: Retrospective cohort study of pregnancies that underwent genomic sequencing for ultrasound anomalies (2017–2024) and had at least one pathogenic or likely pathogenic variant indicating a fetal genetic disease. We calculated the proportion of pregnancies with inherited fetal AR and XL genetic diseases and evaluated the actual carrier screening pursued. Further, we calculated the theoretical diagnostic yield of commercially available ECS panels for predicting fetuses at risk for the inherited conditions observed in our cohort.

**Results**: Of 153 pregnancies with a fetal genetic diagnosis identified by genomic sequencing, 65 had inherited diseases and 88 occurred sporadically. Twenty‐nine (19%) had inherited AR or XL conditions (25 and 4, respectively). Twenty‐one of these (72%) underwent carrier screening and only one at‐risk couple was identified for a genetic condition unrelated to the fetal anomaly. The theoretical diagnostic yield of ECS for identifying the 29 AR and XL inherited conditions in our cohort ranged from 0–52% due to variant classification and number of genes evaluated (Figure 1). At most, 10% of the 153 fetuses with a monogenic disease in our cohort could have been identified through ECS (Figure 2).

**Conclusion**: Only 19% of our cohort had an inherited AR or XL genetic disease, and at most half of these could theoretically be detected based on current ECS panels. There are risks and benefits to ECS, and a negative result does not eliminate risk of inherited or sporadic fetal genetic disease. Clinical counseling should emphasize the limitations of ECS and include the option for fetal genomic sequencing to address the breadth of potential etiologies.

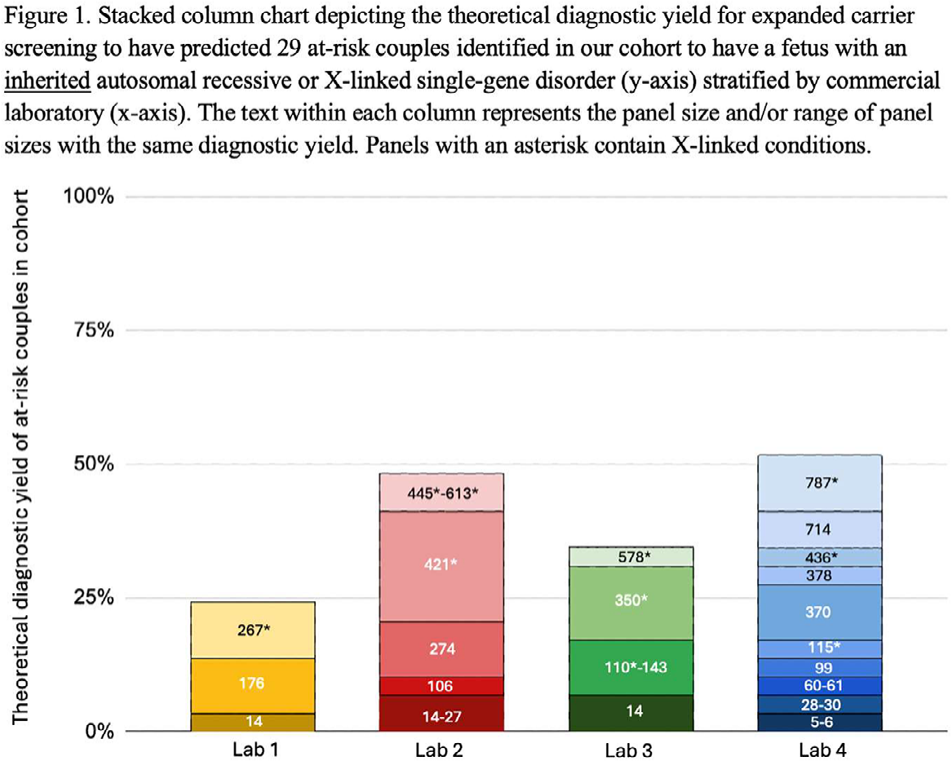

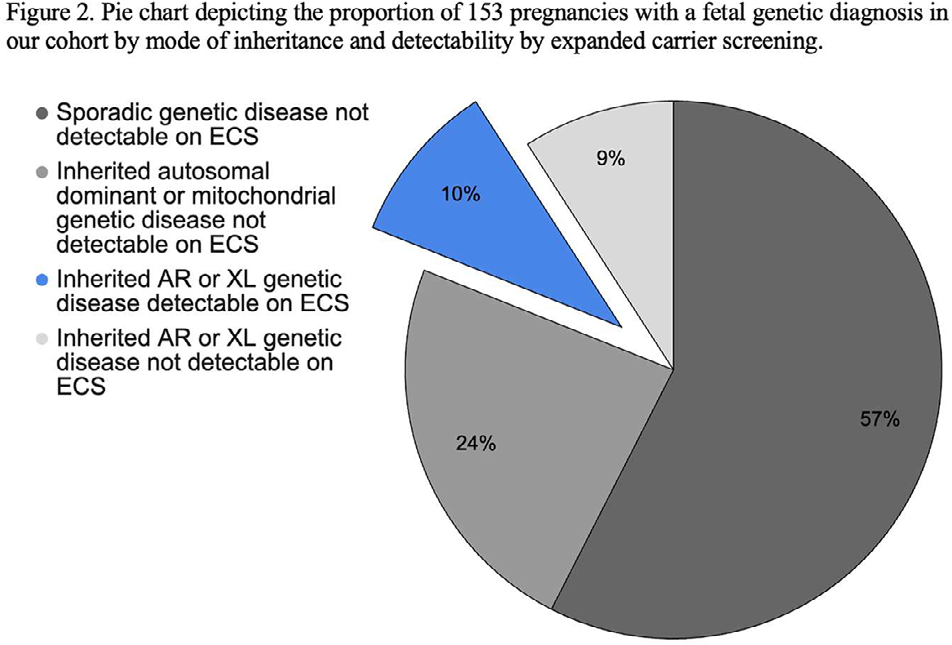

**10:30 AM – 12:00 PM**

## 155 | Tight Versus Standard Postpartum Blood Pressure Control: A Randomized Controlled Feasibility Trial

Carrie Bennett^1^; Rosemary Shay^2^; Sanjana Ghosh^3^; Sila Yavan^1^; Hyagriv Simhan^1^; Malamo Countouris^1^; Janet Catov^3^; Alisse Hauspurg^4^

^1^University of Pittsburgh Medical Center, Pittsburgh, PA; ^2^University of Massachusetts, Worcester, MA; ^3^University of Pittsburgh, Pittsburgh, PA; ^4^Brown University, Providence, RI

**Objective**: There are few data about goal postpartum blood pressure (BP) after a hypertensive disorder of pregnancy (HDP). We assessed the feasibility of a pilot RCT and impact of tight versus standard BP control in the first 6 weeks postpartum (PP) in individuals with HDP.

**Study Design**: We performed a single‐blind feasibility RCT from 11/2023–4/2024 within our institution's remote BP monitoring program. Participants with HDP and no history of chronic HTN were randomized during the delivery hospitalization to one of two arms for initiation and titration of antihypertensives; (1) tight control aligned with American Heart Association guidelines: inpatient BP ≥140/90 mmHg and/or outpatient ≥135/85 mmHg or (2) standard control: BP ≥150/100 mmHg. Participants underwent study visits at 6 weeks and 6 months PP. The primary outcome was feasibility, defined as the proportion of participants enrolled and retained to 6 months PP. Secondary outcomes included home and study visit BP, use of antihypertensives, unplanned health utilization, and persistent HTN at 6 weeks and 6 months PP.

**Results**: Of 143 individuals approached, 61 were randomized (43% enrollment) over a 5‐month time course. There was 80% retention to 6 months PP. Compared to standard control, the tight control arm had lower mean/maximum outpatient systolic BP and study visit systolic BP at 6 weeks PP. Compared to standard control, there were lower rates of Stage 2 HTN (BP ≥140/90 mmHg) at 6 months PP in the tight control arm (4.8% vs 28.6%, *p* = 0.03). There were no differences in unplanned health utilization, medication discontinuation due to symptoms, or lactation continuation between arms.

**Conclusion**: We show that randomization to tight BP control is feasible in the early PP period and may result in lower rates of Stage 2 HTN at 6 months PP. If confirmed in larger trials, this suggests that the immediate PP period may be a critical time for optimizing BP and supports development of interventions to improve long‐term maternal health after HDP.

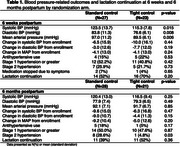

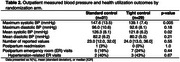

**10:30 AM – 12:00 PM**

## 156 | Fear of Childbirth Among Pregnant Women: Sociodemographic and Discrimination Influences in a Community Sample

Cassandra Heiselman^1^; Clare Whitney^2^; Giselle A. Gerardi^3^; Emily Farkas^4^; Alessa Ramos Vargas^5^; Nicole Lis^5^; Heidi Preis^6^

^1^Stony Brook University Hospital, Stony Brook, NY; ^2^School of Nursing Stony Brook University, Stony Brook, NY; ^3^School of Nursing Stony Brook University, Stony Brook University, NY; ^4^Stony Brook Medicine, Stony Brook, NY; ^5^Renaissance School of Medicine at Stony Brook University, Stony Brook, NY; ^6^Stony Brook University, Stony Brook, NY

**Objective**: Tokophobia, high levels of fear of childbirth, is associated with worse maternal and perinatal outcomes. Emerging data suggest that women from marginalized sociodemographic groups are at higher risk for tokophobia. In the current study we examined the role of discrimination in the occurrence of tokophobia which has not been documented in the U.S.

**Study Design**: The study included 206 women in the first trimester that were recruited 9/2023–1/2025 at outpatient clinics affiliated with a New York academic medical center. Participants completed a survey including the Everyday Experiences Scale, Discrimination in Medical Settings Scale, and the Fear of Birth Scale. A cutoff score of ≥54 was used to identify tokophobia. Analysis included t tests, Chi‐square, and multivariable logistic regression using SPSS 29.0.

**Results**: Participants included *n* = 92 (44.7%) nulliparous patients and *n* = 58 (29.9%) reported fear of birth levels indication tokophobia. Additional sample characteristics are in Table 1. Higher rates of tokophobia were observed among nulliparous compared to multiparous participants (45.9% vs. 17.4%) (X^2^(1) = 18.5, *p*< 0.001). Tokophobia was not associated with any of the sociodemographic factors (Table 1) however, it was associated with significantly higher frequency of everyday discrimination experiences in the past year (*p* = 0.04) and higher frequency of experiences of discrimination in medical settings (*p* = 0.01) (Figure 1). After controlling for parity, frequency of experiencing discrimination in medical settings was associated with double the risk of tokophobia (aOR 2.1; 95% CI 1.2–3.7). General discrimination was not associated with tokophobia after controlling for parity.

**Conclusion**: Past experience of discrimination, particularly in medical settings, increases the risk of tokophobia highlighting the importance of patient‐centered, culturally sensitive care Future research in diverse samples should examine whether higher rates of tokophobia among Black pregnant women that were observed in other studies is attributable to experiences of discrimination in medical settings.

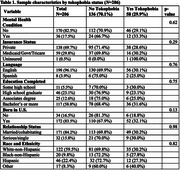

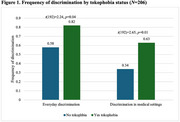

**10:30 AM – 12:00 PM**

## 157 | Bleeding Risk at Birth: The Hidden Cost of Low‐Dose Aspirin in Pregnancy

Cassidy A. O'Sullivan^1^; Jessica C. Fields^2^; Suneet P. Chauhan^3^; Matthew K. Hoffman^4^

^1^Christiana Care Health Services, Newark, DE; ^2^Delaware Center for Maternal Fetal Medicine, Newark, DE; ^3^Delaware Center for Maternal‐Fetal Medicine of Christiana Care, Newark, DE; ^4^Christiana Care Health System, Newark, DE

**Objective**: Low‐dose aspirin is widely prescribed during pregnancy to reduce the risk of pre‐eclampsia. However, its impact on maternal bleeding and transfusion risk at delivery remains unclear. We aimed to evaluate whether aspirin use is associated with increased blood loss or transfusion rates during childbirth.

**Study Design**: This was a retrospective cohort study of 46,796 births at a single tertiary care center. We compared patients who received low‐dose aspirin during pregnancy (*n* = 8628) to those who did not (*n* = 38,168). Primary outcomes included quantitative blood loss (QBL) and need for blood transfusion. Recognizing that the populations of pregnancies prescribed aspirin would differ, we used directed acrylic graphs to define variables to be included in multivariable modeling.

**Results**: Pregnancies prescribed aspirin had significantly higher mean QBL (583 mL vs. 479 mL, *p* < 0.001) and were more likely to require transfusion (5.97% vs. 3.67%, *p* < 0.001), with an odds ratio of 1.67 (95% CI 1.48–1.89). Additionally, aspirin use was associated with a higher likelihood of receiving ≥ 4 units of blood (*p* < 0.001) with an odds ratio of 2.22 (95% CI 1.7–2.89) . Individuals taking aspirin were 2.3 times more likely to receive a transfusion than those not taking aspirin. After controlling for confounding factors identified in a directed acyclic graph including multiple gestation, cesarean delivery, and maternal age, the adjusted odds ratio for transfusion was 1.42 (95% CI 1.28–1.59).

**Conclusion**: Low‐dose aspirin use in pregnancy is independently associated with increased peripartum blood loss and transfusion risk. Given these findings, discontinuing aspirin at 37 weeks' gestation may reduce maternal morbidity and warrants further prospective evaluation.

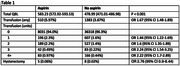

**10:30 AM – 12:00 PM**

## 158 | Placental Growth Factor Concentrations Predict Genetic Testing Yield in Isolated Severe Early‐Onset Fetal Growth Restriction

Catherine M. Windrim^1^; Nicole Jarosz^1^; Nicole Gojska^2^; John W. Snelgrove^1^; Rachel Gladstone^1^; Kelsey McLaughlin^1^; Sebastian R. Hobson^1^; David Chitayat^2^; Homero Flores Mendoza^1^; Rory Windrim^3^; John C. Kingdom, MD^1^

^1^Maternal Fetal Medicine Division, Department of Obstetrics and Gynaecology, Mount Sinai Hospital, University of Toronto, Toronto, Ontario, Canada, Toronto, ON; ^2^Prenatal Diagnosis and Medical Genetics Program, Mount Sinai Hospital, University of Toronto, Toronto, Ontario, Canada, Toronto, ON; ^3^Maternal Fetal Medicine Division, Ontario Fetal Center, Department of Obstetrics and Gynaecology, Mount Sinai Hospital, University of Toronto, Toronto, ON, Canada, Toronto, ON

**Objective**: To determine whether circulating placental growth factor (PlGF) concentrations can predict genetic testing yield in pregnancies with isolated severe early‐onset fetal growth restriction (eoFGR).

**Study Design**: Retrospective cohort study of singleton pregnancies with severe eoFGR (<3rd percentile) at 20–28 weeks gestation between March 2017 and December 2024. Exclusion criteria: stillbirth at evaluation, major fetal structural abnormalities, and major aneuploidy by NIPT. Primary outcome was genetic abnormality detection from amniocentesis or cord blood sampling, stratified by PlGF concentrations using 100 pg/mL threshold.

**Results**: Of 291 pregnancies, 91 (31.3%) underwent genetic evaluation. PlGF <100 pg/mL occurred in 223 cases (76.6%) versus 68 cases (23.4%) with PlGF ≥100 pg/mL. PlGF demonstrated striking inverse relationship with genetic abnormalities: PlGF ≥100 pg/mL yielded 36.8% (14/38) genetic abnormalities versus 5.7% (3/53) in PlGF <100 pg/mL group (OR 9.72, 95% CI 2.5–37.1, *p* < 0.001). Low PlGF (<100 pg/mL) indicated placenta‐mediated disease with 94.3% negative predictive value for genetic abnormalities. Genetic testing rates were higher in PlGF ≥100 pg/mL group (55.9% vs 23.8%, *p* < 0.001). All genetic abnormalities had normal qfPCR results. PlGF <100 pg/mL group showed predominantly placenta‐mediated phenotype with higher maternal vascular malperfusion (75.8% vs 32.4%, *p* < 0.001), preeclampsia/HELLP syndrome (19.3% vs 7.4%, *p* = 0.02), earlier delivery (29.1 vs 33.9 weeks, *p* < 0.001), lower birth weights (823 g vs 1500 g, *p* < 0.001), and reduced live births (70.9% vs 85.3%, *p* = 0.02).

**Conclusion**: In severe eoFGR, low PlGF (<100 pg/mL) indicates placenta‐mediated disease with excellent negative predictive value (94.3%) for genetic abnormalities, allowing prioritization of maternal health surveillance. High PlGF (≥100 pg/mL) indicates 36.8% genetic abnormality risk not captured by routine screening, supporting amniocentesis consideration. PlGF provides rapid, objective biomarker guidance for distinct clinical pathways in severe eoFGR.

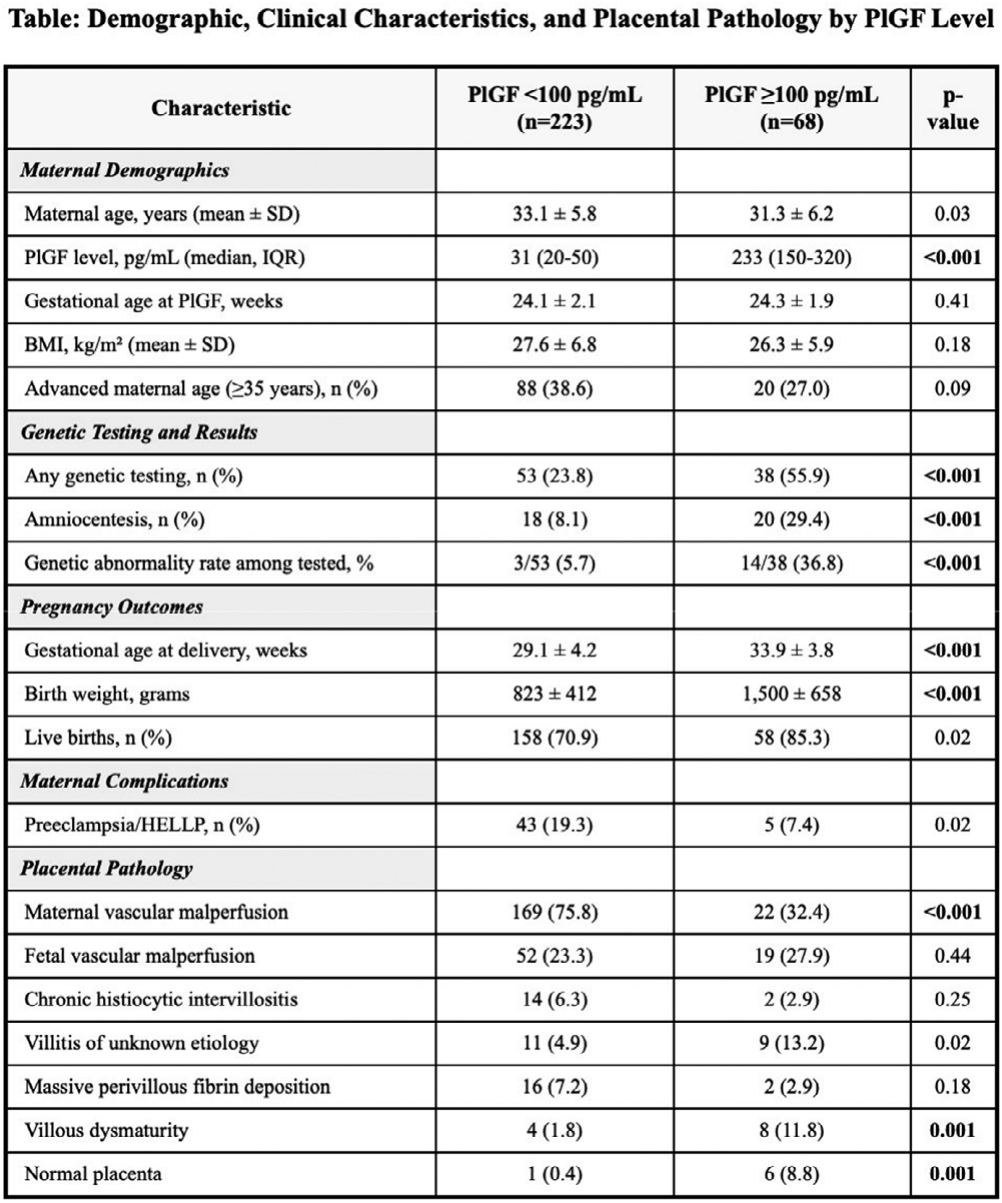

**10:30 AM – 12:00 PM**

## 159 | Effectiveness of a Novel Digital Portable Obstetric Bedside Ultrasound Curriculum

Cecilia Leggett^1^; Saumaya Sao^2^; Alexandra C. Gallagher^3^; Jane Chueh^4^; Elizabeth B. Sherwin^5^; Irogue I. Igbinosa^5^; Erica Wu^4^; Emi Komatsu^4^; Amy Judy^4^

^1^Stanford University School of Medicine, Palo Alto, CA; ^2^Stanford University School of Medicine, Stanford, CA; ^3^Stanford University, Palo Alto, CA; ^4^Stanford University, Stanford University, CA; ^5^Stanford University, Stanford, CA

**Objective**: Ultrasound curriculum implementation is disparate among obstetrics and gynecology (OBGYN) training programs due to difficulty integrating learning goals into clinical environments. This study evaluates whether providing trainees with handheld ultrasound devices (pocket probes) improves education outcomes compared to traditional ultrasound machines.

**Study Design**: A prospective, randomized controlled trial was conducted at an urban academic center from January‐June 2025. OBGYN trainees on a labor and delivery rotation were randomized to either a pocket probe (Butterfly iQ3) that connected to personal devices for image display or traditional ultrasound machines (GE Voluson P8) available on the unit. All participants received a digital obstetric ultrasound curriculum.

The primary outcome was curriculum completion measured by number and type of scans performed. 7 participants per group were needed to detect a mean difference of 18% (alpha 0.05).

Secondary outcomes included knowledge assessment scores, image quality, and perceived workload assessed by a validated survey (NASA Task Load Index). Mann‐Whitney, paired T‐tests, and chi‐square tests were used for analysis.

**Results**: 23 trainees were enrolled (13 pocket, 10 traditional). Groups were similar in age, training year, pre‐curriculum scores, and shift schedules.

The pocket group had significantly higher curriculum completion (median 67% vs 8.3%, *p* = 0.0016, Figure 1). Image quality scores were significantly higher in the pocket group (4.0 vs 3.0, *p* = 0.0003). Post‐curriculum knowledge scores were not significantly different between groups.

The pocket group more often rated workload as low/moderate, while controls rated workload high/very high (*p* = 0.046).Lower mental, physical, temporal demand, and lower frustration were seen among those randomized to pocket probes (Figure 2).

**Conclusion**: Use of pocket‐sized ultrasound probes was associated with significantly greater curriculum engagement, improved image quality, and lower perceived workload among OBGYN trainees in clinical training environments.

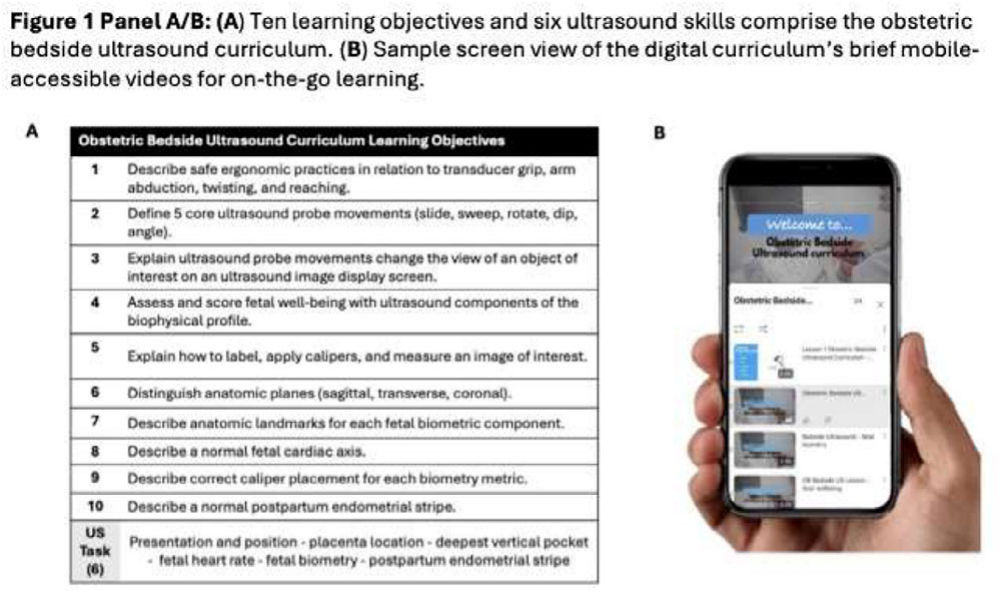

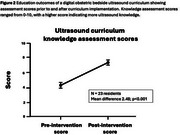

**10:30 AM – 12:00 PM**

## 160 | Planned C‐Section or Attempted Vaginal Delivery: Which is the More Environmentally Friendly?

Sherazade Jaafar^1^; Sophie Cordier^2^; Sayed Gillani^2^; Charles Garabedian^1^

^1^CHU Lille, Lille, Nord‐Pas‐de‐Calais; ^2^Primum Non Nocere, Béziers, Languedoc‐Roussillon

**Objective**: To compare the environmental impact of attempted vaginal birth and planned cesarean section and to identify key levers to improve our impact.

**Study Design**: This single‐center study (Lille, France) used Life Cycle Assessment (LCA) methodology to evaluate the environmental impact of childbirth from labor admission to discharge. Two delivery scenarios—attempted vaginal delivery and planned c‐section—were compared, including associated complications and anesthesia. Environmental impacts covering 80% of total effects were analyzed.

**Results**: Depending on the environmental indicator assessed, attempted vaginal delivery results in an impact between 4% and 84% lower than planned c‐section—even when accounting for potential complications, various modes of analgesia, and all possible delivery outcomes. For exemple, it reduces the impact by 46% in terms of climate change, 44% of fossil ressources use, 30% of mineral and metal ressource use, and 26% of water consumption. In our center in France, the estimated carbon footprint of attempted vaginal delivery is 23.2 kg CO_2_e, compared to 42.9 kg CO_2_e for planned c‐section.

**Conclusion**: Choosing vaginal delivery over scheduled c‐section, when clinically appropriate, could reduce environnemental impact. Beyond mode of delivery, healthcare professionals can reduce environmental impact by addressing other modifiable aspects of obstetric care, without compromising safety or quality. These include reducing the use of medical devices and consumables, as well as acting directly on the devices themselves—particularly through improvements in raw material sourcing and local manufacturing.

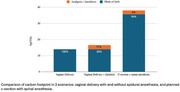

**10:30 AM – 12:00 PM**

## 161 | Metformin Prevents Miscarriage by Reducing Placental Inflammation in a Mouse Model of Preterm Birth

Chiara Tersigni^1^; Marianna Onori^2^; Giuliana Beneduce^2^; Fabio Sannino^2^; Nicoletta Di Simone^3^; Tullio Ghi^4^

^1^Fondazione Policlinico Gemelli IRCCS, Fondazione Policlinico Gemelli IRCCS, Lazio; ^2^Catholic University of Sacred Heart of Rome, Catholic University of Sacred Heart of Rome, Lazio; ^3^IRCCS Humanitas Research Hospital/Humanitas University, IRCCS Humanitas Research Hospital/Humanitas University, Lombardia; ^4^Fondazione Policlinico Gemelli IRCCS/Catholic University of Sacred Heart of Rome, Fondazione Policlinico Gemelli IRCCS/Catholic University of Sacred Heart of Rome, Lazio

**Objective**: Intra‐amniotic infection/inflammation is a trigger mechanism leading to preterm birth (PTB). Abnormal glucose tolerance may contribute to its etiopathogenesis by amplifying tissue inflammation and increasing susceptibility to infections. Metformin is largely used in glucose metabolism disorders of pregnancy for its insulin‐sensitizing effect, but its anti‐inflammatory properties have been poorly investigated. The aims of this study were to evaluate the effects of metformin in a mouse model of PTB in terms of: a) amniochorionic membranes, placental and serum inflammation; b) prevention of miscarriage and PTB.

**Study Design**: Swiss CD1 pregnant mice were treated with intra‐peritoneally injection of lipopolysaccharide (LPS) (14.5 days post coitum‐dpc) with (group 1; *n* = 15) or without (group 2: *n* = 15) 200mg/Kg of metformin administered by oral gavages (8.5/10.5/12.5/14.5 dpc). Control groups were treated with PBS (*n* = 15) or metformin (*n* = 15) only. The expression of the inflammasome NLRP‐3, IL‐1β, IL‐6, IFN‐γ, TNF‐α and insulin receptor (ISR) were quantified by ELISA and Western blot in amniochorionic membranes, placentas and maternal sera.

**Results**: Reduced miscarriage rate (**b**) and higher pups’ weight (**d**) were observed in group 1 compared to group 2 (*p* < 0.05 and *p* < 0.0001, respectively). Decreased placental expression of NLRP‐3 (**g**, *p* < 0.05), IFN‐γ (**h**, *p* < 0.0001) and TNF‐α (**i**, *p* < 0.05) and reduced expression of NLRP‐3 (**l**, *p* < 0.05), IL‐1β (**m**, *p* < 0.05), IL‐6 (**n**, *p* < 0.001), and TNF‐α (**o**, *p* < 0.0001) in amniochorionic membranes were observed in group 1 compared to group 2. Metformin significantly increased ISR levels in amniotic membranes (**p**) and sera (**k**) (*p* < 0.0001 and *p* < 0.001, respectively) and decreased serum IL‐6 levels (**j**, *p* < 0.01).

**Conclusion**: Oral administration of metformin in a mouse model of PTB is able to: a) decrease systemic, amniochorionic and placental inflammation; b) reduce miscarriage rate and increase pups’ weight. This study highlights the anti‐inflammatory properties of metformin and its potential clinical application in the prevention of miscarriage and PTB.

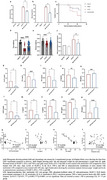

**10:30 AM – 12:00 PM**

## 162 | Rates and Indications for Cesarean Section in Obstetrical Patients With Spinal Cord Injury

Christina Antinora^1^; Anne Harris^2^; Claire J. Mazzia^3^; Anne Berndl^4^

^1^Department of Obstetrics and Gynecology, University of Toronto, Department of Obstetrics and Gynecology, University of Toronto, ON; ^2^Dalla Lana School of Public Health, University of Toronto, Dalla Lana School of Public Health, University of Toronto, ON; ^3^University of Toronto, University of Toronto, ON; ^4^Department of Obstetrics and Gynecology, Division of Maternal Fetal Medicine, University of Toronto, Toronto, ON, Toronto, ON

**Objective**: Spinal cord injury (SCI) in young adults is on the rise globally. Little is known about pregnancy in patients with SCI, including optimal mode of delivery. People with SCI are more likely to deliver via cesarean section (CS), however the reason for this remains unclear. People with SCI may require CS for common obstetrical reasons and for SCI‐specific indications including prior surgery, concerns with neuraxial analgesia or autonomic dysreflexia, and provider preference. Our study aims to determine rates and indications for CS among pregnant patients with and without SCI.

**Study Design**: Data was obtained from a large online cross‐sectional observational questionnaire examining reproductive health outcomes of people with SCI between 2019–2021. We compared rates and indications for CS in respondents with SCI (*n* = 259) and without (*n* = 301) SCI using descriptive statistics and chi‐square analysis.

**Results**: 38% of patients with SCI delivered via CS compared to 23% without (*p* < 0.01). Approximately half of CS occurred in labour in both SCI (46%) and non‐SCI patients (55%) (*p* > 0.05). Indications among SCI patients included safety concerns secondary to SCI (30%), malpresentation (24%), and maternal choice (17%). Among patients without SCI, most common indications were arrest of dilation (25%), cephalopelvic disproportion (22%), and previous CS (19%). 41% of patients with SCI underwent CS under general anesthesia, compared to 19% for those without SCI (*p* < 0.01). Specific indications for CS which differed significantly (*p* < 0.01) between patients with and without SCI were maternal choice, cephalopelvic disproportion, and arrest of dilation.

**Conclusion**: Rates of CS among SCI patients is significantly higher than those without. Maternal request and perceived risks secondary to SCI were among the most common indications, compared to more traditional obstetrical indications in non‐SCI patients. This significantly different pattern suggests the need for tailored guidelines that consider the medical complexity of people with SCI, and shared decision‐making to support evidence‐based delivery planning in this population.

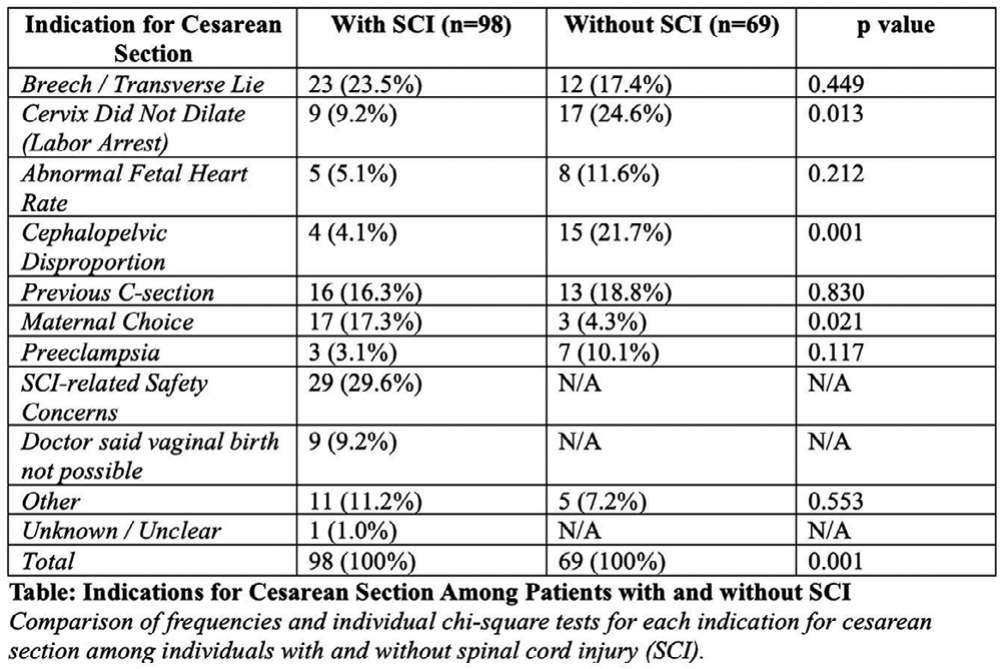

**10:30 AM – 12:00 PM**

## 163 | Risk Factors for Stillbirth in Pregnancies Complicated by Diabetes

Christina M. Boulineaux^1^; Claire A. McIlwraith^2^; Amanda Assad^3^; Christina T. Blanchard^2^; Ashley N. Battarbee^2^

^1^Department of Obstetrics and Gynecology, University of Alabama at Birmingham, University of Alabama at Birmingham, AL; ^2^Center for Research in Women's Health, University of Alabama at Birmingham, Birmingham, AL; ^3^Department of Obstetrics and Gynecology, University of Alabama at Birmingham, Department of Obstetrics and Gynecology, University of Alabama at Birmingham, AL

**Objective**: To identify risk factors for stillbirth among pregnant individuals with diabetes mellitus (DM).

**Study Design**: Retrospective cohort study of pregnant patients with gestational or pregestational DM who received prenatal care and delivered at a tertiary care center (2012–2025). The primary outcome was stillbirth. Secondary outcomes included gestational age (GA) at stillbirth and composite severe neonatal morbidity (Table 2). Descriptive statistics were used to summarize the study cohort, and multivariable logistic regression with backward selection identified the factors that were independently associated with stillbirth and secondary outcomes.

**Results**: Among 5737 included patients, 121 (2.1%) stillbirths occurred at an average GA of 27.9 ± 8.0 weeks. The cohort included 68.2% patients with gestational DM, 22.4% type 2 DM, and 9.4% type 1 DM. Mean maternal age was 30.5 ± 5.9 years, with 41.9% patients identifying as black race (Table 1). In multivariable analysis, third trimester HbA1c was the only factor significantly associated with stillbirth (aOR 1.41, 95% CI 1.01–1.97). For GA at stillbirth, maternal age, self‐identifying as black or white race (compared with other race), gestational DM (compared with type 2), higher HbA1c in early pregnancy and preeclampsia were associated with later GA at stillbirth (Table 2). Higher HbA1c in the third trimester was associated with earlier GA at stillbirth (Table 2). For severe neonatal morbidity, type 1 DM (compared to type 2) and chronic hypertension were significant risk factors (Table 2). In sensitivity analyses stratified by DM type, among patients with type 2 DM, higher third trimester HbA1c (aOR 1.66, 95% CI 1.07–2.58) and multiple gestation (aOR 6.80, 95% CI 1.30–35.53) were associated with increased stillbirth risk. No independent risk factors were identified among patients with gestational or type 1 DM.

**Conclusion**: Third trimester HbA1c emerges as the primary modifiable risk factor for stillbirth in patients with DM. Racial disparities in stillbirth timing and DM type‐specific risk patterns warrant targeted surveillance strategies for high‐risk populations.

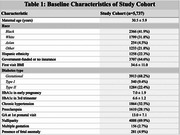

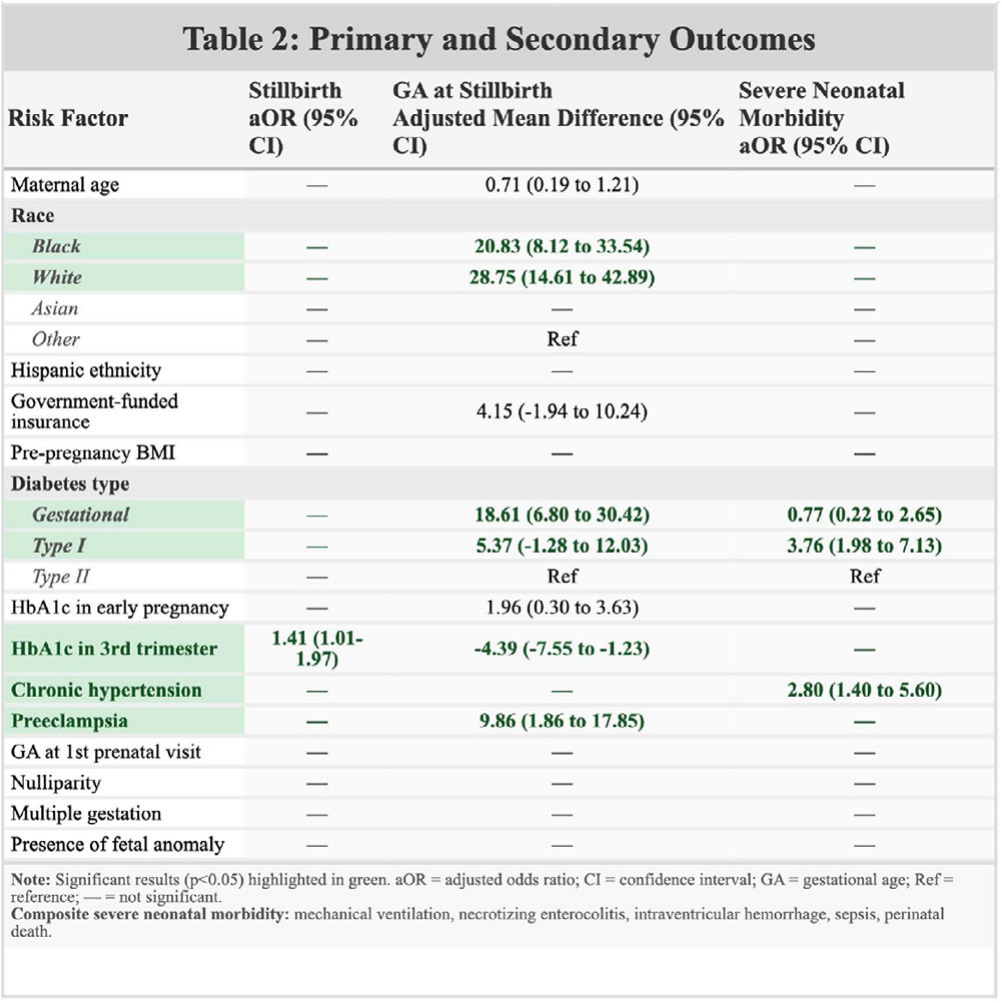

**10:30 AM – 12:00 PM**

## 164 | Fetal Weight Estimation in Gastroschisis: A Formula Comparison

Christina Cortes^1^; Sami Backley^2^; Anthony Chartier^3^; Felicia V. LeMoine^2^; Ashley Salazar^4^; Ramesha Papanna^5^; Jimmy Espinoza^4^; Anthony Johnson^4^; Edgar A. Hernandez‐Andrade^6^

^1^University of Texas ‐ Houston, Houston, TX; ^2^Division of Fetal Intervention, Department of Obstetrics, Gynecology and Reproductive Sciences, McGovern Medical School at UTHealth Houston, Houston, TX; ^3^Department of Obstetrics, Gynecology and Reproductive Sciences, McGovern Medical School at UTHealth Houston, Houston, TX; ^4^UTHealth Houston Fetal Institute; Division of Fetal Intervention, Department of Obstetrics, Gynecology and Reproductive Sciences, McGovern Medical School at UTHealth Houston, Houston, TX; ^5^University of Texas Health Science Center in Houston, McGovern Medical School, Houston, TX; ^6^Division of Maternal‐Fetal Medicine, Department of Obstetrics, Gynecology and Reproductive Sciences, McGovern Medical School at UTHealth Houston, Houston, TX

**Objective**: Estimating fetal weight in fetuses with gastroschisis is challenging due to altered abdominal circumference (AC) resulting from the abdominal wall defect. The Hadlock formula is widely used for estimating fetal weight (EFW) but may be less accurate in this population. The Siemer formula has been proposed as an alternative, aiming to improve EFW accuracy and growth classification. This study compared the accuracy of the Hadlock and Siemer formulas in predicting actual birthweight (BW) when ultrasound was performed within two weeks of delivery.

**Study Design**: Fetuses with gastroschisis who underwent EFW assessment within two weeks of delivery were retrospectively analyzed at a single tertiary referral center between 2016–2024. EFW was calculated using the same biometric parameters for both formulas. Actual BW was recorded, and the absolute percent difference between EFW and BW was calculated. The formula with the smaller absolute difference from BW was considered more accurate.

**Results**: A total of 45 fetuses were included. The median gestational age at the last ultrasound was 34w1d (29 + 6 to 36 + 6), and the median BW was 2340 g (1360–3210 g). The Siemer formula was closer to the actual BW in 26 cases (57.8%), while the Hadlock formula was more accurate in 19 cases (42.2%), though this difference was not statistically significant (*p* = 0.2). The mean interval between ultrasound and delivery was 0.9 weeks. Among fetuses delivered within one week of ultrasound, the mean percent difference between Hadlock EFW and BW was −15.8% (SD 7.9%), compared to −14.1% (SD 10.2%) for Siemer. For those delivered 1–2 weeks later, mean differences were +20.8% (SD 11.4%) for Hadlock and +21.5% (SD 11.3%) for Siemer.

**Conclusion**: In fetuses with gastroschisis, no significant difference in accuracy was found between the Hadlock and Siemer formulas. However, EFW predictions were more accurate when delivery occurred within one week of ultrasound.

**10:30 AM – 12:00 PM**

## 165 | Hofbauer Cells Exhibit Distinct Transcriptional Signatures in Past‐Chronic Placental Malaria

Christine A. Blauvelt^1^; Nida Ozarslan^1^; John Ategeka^2^; Megan Chidboy^1^; Sirirak Buarpung^1^; Jimmy Kizza^2^; Abel Kakuru^2^; Moses R. Kamya^2^; Grant Dorsey^1^; Phillip J. Rosenthal^1^; Joshua F. Robinson^1^; Stephanie L. Gaw^1^

^1^University of California, San Francisco, San Francisco, CA; ^2^Infectious Diseases Research Collaboration, Kampala, Kampala

**Objective**: Placental malaria (PM) is due to sequestration of *Plasmodium falciparum*‐infected red blood cells in placental intervillous spaces and subsequent inflammatory responses. The impact of PM on Hofbauer cells (HBCs) and consequences for placental function and fetal immune development remain poorly understood. This study aimed to define transcriptional alterations in HBCs in past‐chronic PM.

**Study Design**: We conducted a nested case‐control study within a randomized controlled phase III trial in Uganda (NCT04336189), investigating intermittent preventative treatment for malaria in pregnancy. PM status was determined by Rogerson criteria. Formalin‐fixed paraffin‐embedded placental tissue samples from pregnancies with past‐chronic PM (*n* = 4) and healthy controls (*n* = 7) were profiled using the NanoString GeoMx Digital Spatial Profiler. Regions of interest from placental villi were segmented to enrich for HBCs using CD68 immunofluorescence. Spatial RNA sequencing was performed on segmented HBCs, followed by differential gene expression and pathway enrichment analyses.

**Results**: Patient characteristics were similar between groups, including maternal age, gestational age at delivery, and fetal sex. Transcriptomic profiling identified 463 differentially expressed genes in HBCs from PM‐exposed placentas vs controls (Figure 1; unadj *p* < 0.05 and abs FC >1.5). Past‐chronic PM samples exhibited upregulation of genes associated with interferon‐driven antipathogen responses (*MX1, IFI27)*, and downregulation of immunoregulatory signaling *(IL27)*, angiogenic remodeling *(PDGFD)*, and cellular stress responsiveness *(TP73*). GSEA pathway enrichment analysis revealed significant enrichment in interferon signaling, antiviral response, antigen processing and presentation, and cellular metabolism pathways (Figure 2; adj *p* < 0.01).

**Conclusion**: Our findings suggest that past‐chronic PM exposure is associated with persistent transcriptional reprogramming of fetal HBCs. Further studies are needed to elucidate how these changes affect placental vascular remodeling, fetal immune programming, and risk of adverse obstetric and neonatal outcomes.

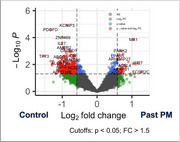

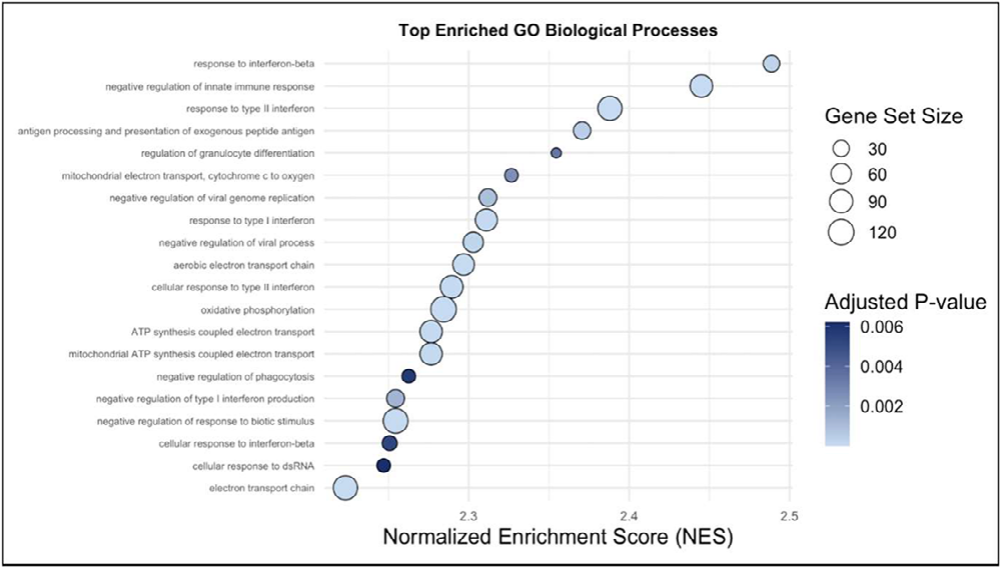

**10:30 AM – 12:00 PM**

## 166 | Long‐Term Progression to Overt Hypothyroidism Following Subclinical Thyroid Disease Identified During Pregnancy

Christine E. Henricks^1^; Sophi Farid^1^; Victoria Starnes^2^; Donald D. McIntire^1^; Morris Bryant^3^; David B. Nelson^1^; F. Gary Cunningham^1^

^1^University of Texas Southwestern Medical Center, Dallas, TX; ^2^UT Southwestern Medical Center, UT Southwestern Medical Center, TX; ^3^UT Southwestern Medical Center, Dallas, TX

**Objective**: To determine the incidence and timing of progression to overt hypothyroidism among patients with subclinical hypothyroidism (SCH), thyroid peroxidase (TPO) antibodies identified during pregnancy.

**Study Design**: This was a longitudinal cohort study of pregnant patients who underwent thyroid screening before 20 weeks gestation at a large, academic center between 2000 and 2003. Serum thyroid stimulating hormone (TSH) levels were measured, and abnormal results were reflexively tested for free thyroxine (T4); those with both abnormal TSH and free T4 were excluded. SCH was defined as TSH ≥97.5th percentile with free T4 >0.680 ng/dL. TPO antibody positivity was ≥50 IU/mL. Of the 1186 eligible patients from 2000–2003, 718 had follow‐up data. The primary outcome was the development of overt hypothyroidism following the index pregnancy, defined by initiation of thyroid hormone therapy or a clinical diagnosis.

**Results**: Over the 25‐year study period, 238 (33.1%) patients with subclinical thyroid disease in pregnancy progressed to overt hypothyroidism. The risk was highest in those with both SCH and TPO positivity (52.9%), followed by TPO positivity alone (28.4%) and SCH alone (25.0%, *p* < 0.001). While most diagnoses occurred more than 5 years postpartum, a notable proportion developed hypothyroidism within the first five years. Higher baseline TSH was associated with earlier progression of overt hypothyroidism (Figure 1).

**Conclusion**: One in three individuals with SCH or TPO antibody positivity in pregnancy progressed to overt hypothyroidism within 25 years. The combination of elevated TSH and TPO positivity conferred the greatest risk. These findings underscore the need for long‐term follow‐up and suggest a potential need to reevaluate current recommendations regarding universal thyroid screening in pregnancy.

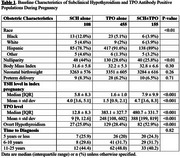

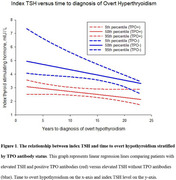

**10:30 AM – 12:00 PM**

## 167 | Simple and Sensitive Recovery of Trophoblasts for Cell‐Based NIPT

Martin Cienciala; Christopher Kan; Daniel Georgiev

Sampling Human Inc., Berkeley, CA

**Objective**: Cell‐free DNA (cfDNA) based non‐invasive prenatal testing (NIPT) revolutionized screening for large chromosomal abnormalities. However, cfDNA's fragmented nature and fetal fraction variation limit the detection of sub‐chromosomal and single‐gene defects. Cell‐based methods, in contrast, isolate high‐quality fetal genomes, but have historically been impractical and unreliable. The Spatial Docking platform (Sampling Human, Inc.) introduces a novel molecular targeting mechanism that simplifies genomic analysis of circulating trophoblast cells and, critically increases the yield and purity of the isolated fractions.

**Study Design**: The Spatial Docking platform was benchmarked on peripheral blood samples combined with a fixed number of trophoblast cells (HTR‐8/SVneo) at concentrations 1 in 3 x 10^4^ cells to 1 in 3 x 10^6^ cells. Samples were processed in one hour, and the isolated fraction was evaluated by immunofluorescence and genomic analysis. Trophoblast recovery and leukocyte contamination rates were calculated. The platform was further tested on clinical samples comprised of whole blood from three pregnant research participants 8–15 WG.

**Results**: In benchmark experiments, trophoblast recovery exceeded 90% with leukocyte contamination less than 10 cells per mL of whole blood (Figure 1). The workflow was scaled to 10mL whole blood samples. For clinical samples, an average of 0.8 trophoblasts per mL of blood were recovered with similar leukocyte contamination of 2.2 nucleated cells/mL.

**Conclusion**: Spatial docking is shown to be a viable method for fetal cell isolation, with performance metrics exceeding reported values for flow cytometry. Furthermore, the one‐step protocol presents a simple solution that minimizes sample transfer and promises to exceed yields of more complex platforms integrating enrichment with imaging and single cell sorting.

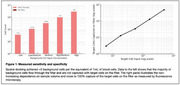

**10:30 AM – 12:00 PM**

## 168 | Sub‐Clinical Inflammatory Trajectories in Mid‐Pregnancy From Vaginal and Blood‐Derived Cytokines and Risk of Early PTB

Claire E. Jensen^1^; Emily Gascoigne^1^; Sarah Heerboth^2^; Hadley Hartwell^1^; Mandy Bush^1^; Annie Dude^1^; William H. Goodnight^1^; Matthew K. Hoffman^3^; Carmen Beamon^4^; Rebecca Fry^1^; Tracy A. Manuck^1^

^1^University of North Carolina, Chapel Hill, NC; ^2^University of North Carolina ‐ Chapel Hill, UNC Chapel Hill, Chapel Hill, NC; ^3^Christiana Care Health System, Newark, DE; ^4^WakeMed, Raleigh, NC

**Objective**: To evaluate associations between changes in mid‐pregnancy vaginal‐ & blood‐derived maternal cytokines, PTB, and GA at delivery.

**Study Design**: Secondary analysis of a prospective cohort enriched for SPTB risk. Asymptomatic multiparas with singletons enrolled <22 wks. Inclusion: 2 vag ± 2 blood samples ≥2 wks apart, obtained 12–28 wks. 12 cytokines/sample were quantified via Luminex. Data were normalized using those at low‐risk for PTB (hx ≥1 term, no PTB, *n* = 85), and Z‐scores (+1Z = 1SD, +2Z = 2SD above low‐risk reference, etc.) calculated. For each cytokine, we calculated:

- **z‐change** = sample2 z‐score–sample1 z‐score;
- t**otal‐z‐change** = sum of z‐change for all 12 cytokines;
- **high‐risk‐z‐change =  **sum of z‐change for those cytokines associated with PTB <35 weeks.

Primary outcome: PTB <35 wks. Secondary outcomes: PTB <37, <32 wks, sample2 time‐to‐delivery. Cox models assessed factors associated with time‐to‐delivery (z‐change score availability to delivery).

**Results**: Of 293 participants, 31/267 (21%) with 2 vag had PTB <35w, 23/134 (17%) with 2 blood had PTB <35w, and 16/105 (15%) with 2 vag & 2 blood samples had PTB <35w. Sample1 was collected at median 16.4 (IQR 15.7–18.6) wks; sample2 a median 5 (IQR 4–6.8) wks later. Total‐z‐change‐vag (mean −0.46 ± 6.7) and total‐z‐change‐blood (mean −0.87 ± 11.5) scores were similar, *p* = 0.76. Six individual vag cytokines (IL‐1β, IL‐4, IL‐6, IL‐8, TNF‐α, and GCSF), the total‐z‐change‐vag score (composite of all 12), and the high‐risk‐z‐change subset (composite of the 6 individual scores IL‐1β, IL‐4, IL‐6, IL‐8, TNF‐α, and GCSF) all increased in hose with PTB <35 weeks vs. later delivery, Figure 1. No blood cytokine z‐changes varied by PTB status. In Cox models, cytokine change scores in vag, but not blood samples remained associated with time‐to‐delivery, Table.

**Conclusion**: Increasing subclinical inflammation among asymptomatic patients in mid‐pregnancy, reflected by relative increases in vaginal‐derived cytokine values over time, is associated with eventual PTB. Evaluating an individual's inflammatory trajectory may provide novel insights into PTB risk and delivery timing.

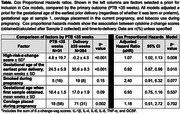

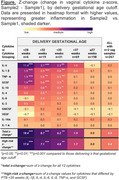

**10:30 AM – 12:00 PM**

## 169 | Timing of Initiation of Continuous Glucose Monitoring in Pregnancy and Adverse Outcomes

Claire A. McIlwraith^1^; Yumo Xue^1^; Madeline Evans^2^; Ashley N. Battarbee^1^

^1^Center for Research in Women's Health, University of Alabama at Birmingham, Birmingham, AL; ^2^Heersink School of Medicine, University of Alabama at Birmingham, Birmingham, AL

**Objective**: Continuous glucose monitoring (CGM) improves pregnancy outcomes in patients with type 1 diabetes and is increasingly being used in patients with type 2 diabetes, but many do not start CGM until after conception. We sought to understand if timing of CGM initiation during pregnancy was associated with adverse outcomes.

**Study Design**: This was a retrospective cohort study of pregnant patients with type 1 and type 2 diabetes with a singleton gestation who started using CGM during pregnancy and delivered at a single tertiary care center from 2018–2024. We excluded patients with fetal anomalies and pregnancy loss <23 weeks. Data were abstracted by electronic medical record review by trained personnel to ensure accuracy. Patients were compared based on timing of CGM initiation: early at <14 weeks versus late at ≥ 14 weeks’ gestation. Our primary outcome was large for gestational age (LGA) infant based on Fenton curves. Multivariable logistic regression estimated the association between early CGM initiation and outcomes. Additionally, we tested for effect modification by diabetes type (1 vs 2) and evaluated GA of CGM initiation as a continuous variable.

**Results**: Of 232 patients included, 93 (40%) initiated CGM early (mean 7.3 ± 4.8 weeks) and 139 (60%) initiated CGM late (22.3 ± 5.3 weeks). Groups differed by many baseline characteristics (Table 1). LGA did not differ between groups (24.7% vs 20.1%; aOR 0.97, 95% CI 0.45–2.10). Secondary maternal and neonatal outcomes were also similar (Table 2). Findings were consistent in analyses stratified by diabetes type and when evaluating GA at CGM initiation as a continuous variable.

**Conclusion**: In this cohort, the timing of CGM initiation was not associated with a significant difference in maternal or neonatal outcomes. Even when early CGM initiation is not possible due to barriers like delayed prenatal care or insurance approval, CGM should still be considered later in pregnancy, as it may offer comparable benefits.

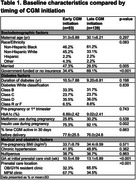

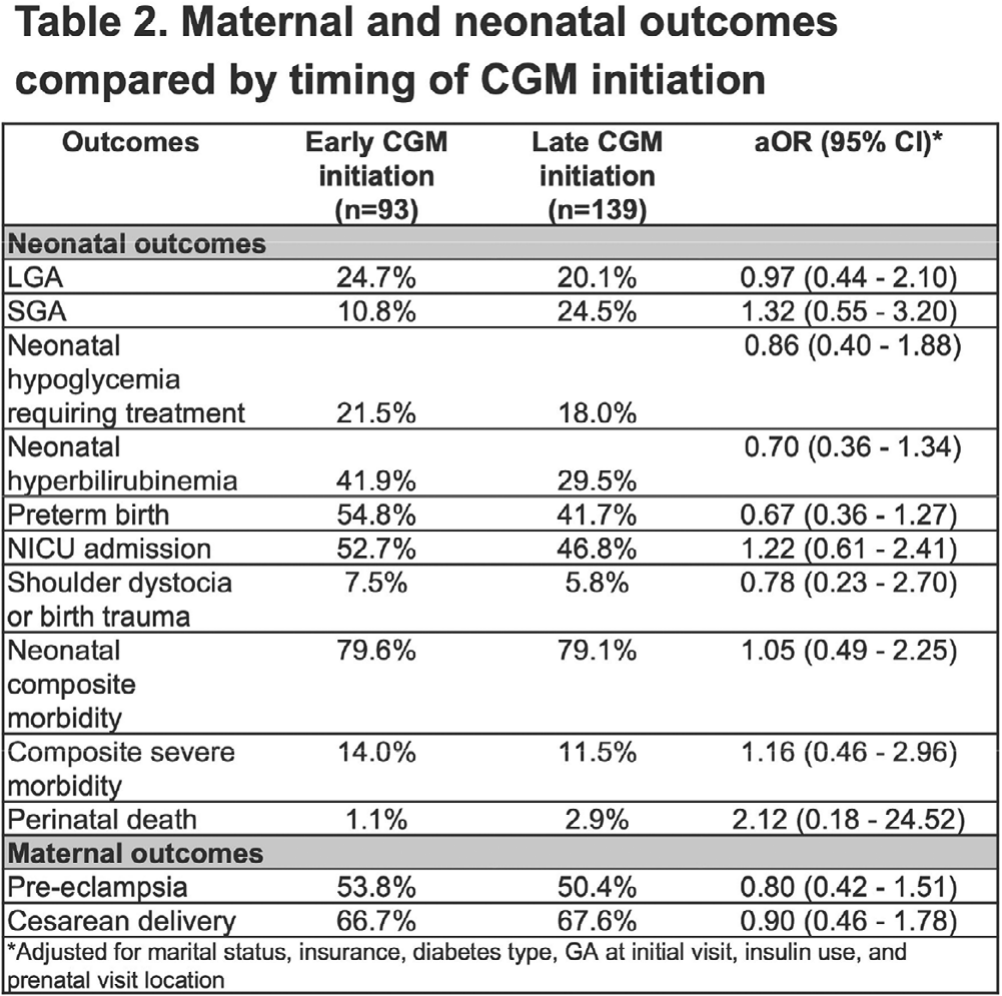

**10:30 AM – 12:00 PM**

## 170 | Very Tight (<130/80 mmHg) Postpartum Blood Pressure Control for Hypertensive Disorders of Pregnancy: A Cost‐Effectiveness Analysis

Clarice G. Zhou^1^; Amelia H. Gagliuso^2^; Lily Ben‐Avi^3^; Aaron B. Caughey^3^; Justin S. Brandt^4^; R. Scott Braithwaite^5^; Christina A. Penfield^6^

^1^Division of Maternal‐Fetal Medicine, Department of Obstetrics and Gynecology, NYU Grossman School of Medicine, NYU Grossman School of Medicine, NY; ^2^Oregon Health and Science University, Portland, OR; ^3^Oregon Health & Science University, Oregon Health & Science University, OR; ^4^Division of Maternal‐Fetal Medicine, Department of Obstetrics and Gynecology, NYU Grossman School of Medicine, New York, NY; ^5^NYU Grossman School of Medicine, NYU Grossman School of Medicine, NY; ^6^Division of Maternal‐Fetal Medicine, Department of Obstetrics and Gynecology, NYU Grossman School of Medicine, New York City, NY

**Objective**: Very tight blood pressure (BP) control <130/80 mmHg with postpartum remote patient monitoring (RPM) decreases emergency department (ED) visits and improves hypertensive disorders of pregnancy (HDP) complications. However, whether benefits outweigh the increased visits and costs to the healthcare system is less clear. Our objective was to determine whether a policy of very tight postpartum BP control using RPM for HDP was cost‐effective compared to standard care.

**Study Design**: A cost‐effectiveness model was constructed to compare very tight BP <130/80 mmHg with RPM to standard care for patients with HDP until 6 weeks postpartum. The model considered number of postpartum and primary care visits, ED visits, readmission, neurological complications, acute kidney injury (AKI), and maternal death. The primary outcomes were total cost and quality‐adjusted life years (QALYs). Probabilities, utilities, and costs were derived from published literature. Costs were expressed in 2025 US dollars with a willingness to pay threshold (WTP) set at $100,000/QALY. Sensitivity analyses were performed to interrogate model assumptions.

**Results**: In our theoretical cohort of 472,000 patients with HDP, very tight BP led to 23,467 more postpartum and 11,946 more primary care visits but resulted in 22,507 fewer ED visits and 1599 fewer hospital readmissions. Tight BP control also led to 17 fewer neurological complications and AKI in the theoretical cohort. A policy of very tight postpartum BP control and RPM led to a net benefit of 37 QALYs for the cohort compared to standard care, but increased healthcare costs by $67 million, suggesting an ICER of $1.8 million, which exceeds the WTP threshold. With sensitivity analysis, when program cost per patient for RPM exceeded $82, standard care remains cost‐effective.

**Conclusion**: While a policy of very tight BP <130/80 mmHg and RPM reduced the rate of hospital readmissions and HDP‐related complications, it is not cost‐effective. Before widespread implementation, strategies to minimize the economic impact of this policy, while maintaining patient‐centered outcomes, may be beneficial.

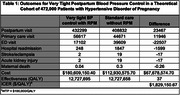

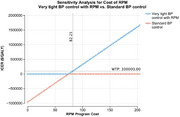

**10:30 AM – 12:00 PM**

## 171 | The Relationship Between Postpartum Mental Health and Sleep Quality

Courtney M. Bisson^1^; Penelope Lialios^2^; Lavisha Singh^3^; Lauren Keenan‐Devlin^4^; Ann E. borders^2^; Sunitha Suresh^5^

^1^The Perinatal Center, The Perinatal Center, OK; ^2^Endeavor Health, Endeavor Health, IL; ^3^Endeavor Health, Endeavor Health Evanston Hospital, IL; ^4^Endeavor Health, Evanston, IL; ^5^Endeavor Health, Endeavor Health System, IL

**Objective**: 60% of maternal deaths occur in the postpartum (PP) period, with postpartum depression (PPD) a major risk factor. While the PP period is six to twelve weeks, nearly 40% of patients are diagnosed with PPD between six months and one year. Poor quality sleep is associated with the development of PP stress and PPD, however has not been assessed in a longitudinal period. The purpose of this study was to examine the relationship between PPD, stress and sleep quality.

**Study Design**: This study was a prospective survey‐based study at a single health care system, recruited in the delivery admission. Starting immediately PP patients received the “Perceived Stress Scale” (PSS) and “Patient Health Questionnaire‐9” (PHQ‐9) monthly until 6 months postpartum. Baseline sleep quality and sleep quality at six months was assessed via the “Pittsburgh Sleep Quality Index” (PSQI). Delivery and sociodemographic information were collected through the electronic medical record. Association between mild PPD (PHQ‐9 > 5), stress (PSS >14) and sleep quality was examined using chi2 analysis. Spearman rank correlation analysis was completed to analyze the association with sleep quality at 0 and 6 months and monthly depression PHQ‐9 and PSS scores.

**Results**: 36 patients were included in this pilot study. Most patients identified as white (72.2%). 88.9% of patients had private insurance, 83.3% were married, and 45% were multiparous. At baseline, the prevalence of mild‐severe PPD and high stress was higher among those with poor sleep compared to good sleep (PPD, 52.4% vs 14.3%; PSS 90.5% vs 57.1%). Similar trends were seen at 6 months with PPD (53.9% vs 0%) and PSS (92.3% vs 33.3%). All p‐values were >0.05. Examining across 6 months, there was a moderate to strong correlation between poor sleep quality at 6 months and PHQ‐9 score peaking at three months PP (Spearman correlation coefficient 0.84, *p* < 0.01).

**Conclusion**: There is a strong correlation between PPD score, PP stress score and poor sleep quality. This represents another possible avenue for screening and intervention such as increasing support longitudinally in the PP period.

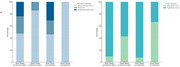

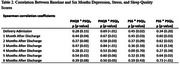

**10:30 AM – 12:00 PM**

## 172 | Adverse Obstetric Outcomes Following Uterine Artery Embolization: A Propensity‐Matched Retrospective Cohort Study

Courtney T. Connolly^1^; Marika Toscano^2^

^1^Johns Hopkins, Baltimore, MD; ^2^Johns Hopkins University, Baltimore, MD

**Objective**: Our aim was to assess the risk of placenta accreta spectrum (PAS), adverse obstetric outcomes, and maternal morbidity in subsequent pregnancies following uterine artery embolization (UAE).

**Study Design**: This was a descriptive study conducted using the TriNetX, a health research network with data from 105 unique health care organizations from 1/2010 to 7/2025. Patients between 12–55 years old with a unique pregnancy were included and divided into two 1:1 propensity score‐matched cohorts: (1) pregnancy following a prior UAE and (2) pregnancy without a history of UAE. Pregnancy and UAE were verified using specific ICD10 and CPT codes. The primary outcome was the rate of PAS diagnosed during pregnancy. Secondary outcomes included rates of postpartum hemorrhage (PPH), placental abnormalities, and severe maternal morbidity (SMM) by Center for Disease Control (CDC) indicators. Statistical analyses were performed in the TriNetX platform.

**Results**: 1378 pregnancies following a prior UAE and 4,084,738 pregnancies without a prior UAE were identified. The UAE‐exposed cohort had significantly higher rates of obesity, diabetes, and hypertensive diseases, and were more likely to be non‐white. After propensity score matching, a total of 1378 patients were included in each cohort. UAE exposed patients had significantly higher odds of PAS (OR 7.21, 95% CI 3.70–4.06), PPH (OR 8.76, 95% CI 6.36–12.05), and retained placenta (OR 5.90 3.00–11.61) despite overall lower rates of Cesarean delivery (3.2% vs 8.4%). They also had a significantly higher odds of experiencing disseminated intravascular coagulation (6.55, 95% CI 3.35–12.83) and composite SMM (3.62, 95% CI 2.72–4.8). There were no differences in rates of placental abruption, placenta previa, or placental malformations.

**Conclusion**: Pregnancies following UAE have significantly higher odds of PAS, PPH, and retained placenta. These results suggest an association between UAE and abnormal placentation in subsequent pregnancies, which should inform prenatal surveillance and delivery planning in UAE‐exposed patients.

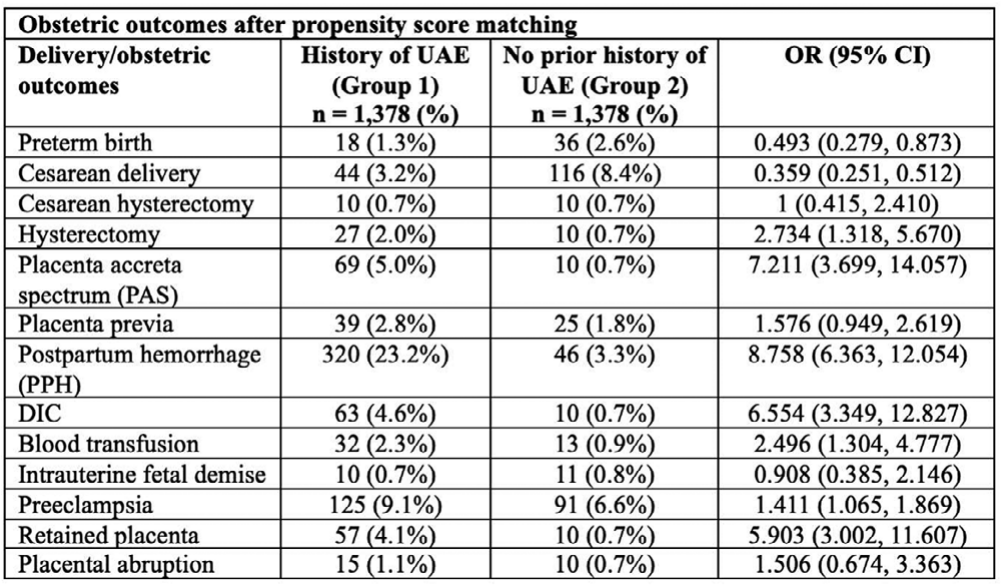

**10:30 AM – 12:00 PM**

## 173 | Mental Health Outcomes Following Cesarean Hysterectomy

Dana Baraki^1^; Marisa R. Imbroane^2^; Ahmed Ahmed^3^; Amol Malshe^3^

^1^Cleveland Clinic, Cleveland Clinic/Cleveland, OH; ^2^Case Western Reserve University School of Medicine, Case Western Reserve University School of Medicine/Cleveland, OH; ^3^Cleveland Clinic Foundation, Cleveland Clinic Foundation, OH

**Objective**: To determine rates of postpartum depression (PPD) and post‐traumatic stress disorder (PTSD) following cesarean hysterectomy (c‐hyst). While PTSD risk is known to increase after emergent c‐hyst, it remains unclear whether this is due to the urgency of the procedure, cesarean delivery itself, or the psychological effects of hysterectomy and loss of fertility. This study compares rates of PPD and PTSD among patients undergoing cesarean—with and without postpartum hemorrhage (PPH)—and c‐hyst—with and without PPH—to assess the independent contribution of hysterectomy to adverse mental health outcomes.

**Study Design**: This retrospective cohort study used the TriNetX research network. Patients who underwent cesarean or c‐hyst between 2015 and June 2025 were identified. A total of 556,584 patients were included: 529,254 cesarean without PPH, 24,480 cesarean with PPH, 1821 c‐hyst without PPH, and 1029 c‐hyst with PPH. Patients were 1:1 matched by age, race, preterm delivery, intrauterine fetal demise, and mental health history. SSRI initiation within 6 months postpartum was also examined.

**Results**: PPD and PTSD rates were 3.8% and 1.1% after cesarean without PPH and 4.7% and 1.6% with PPH (PPD RR 1.24, [1.14–1.35], PTSD RR 1.38, [1.18–1.61]. C‐hyst without PPH was associated with PPD and PTSD rates of 4.3% and 1.7% (PPD RR 1.15, [0.78–1.69], PTSD RR 3.09, [1.58–6.07]). With PPH, c‐hyst was associated with PPD and PTSD rates of 5.7% and 5.0% (PPD RR 1.04, [0.81–1.35], PTSD RR 1.92, [1.45–2.55]; *p* < 0.0001). SSRI initiation was 11.8% after cesarean without PPH vs. 12.9% with PPH (RR 1.10, [1.05–1.16]). C‐hyst without PPH had higher SSRI use (14.2%) vs. cesarean without PPH (RR 1.25, CI 1.04–1.49). No difference in SSRI use was found between hysterectomy groups.

**Conclusion**: Cesarean hysterectomy, particularly with PPH, is associated with increased PTSD risk. While PPH modestly increases PPD and SSRI use, hysterectomy appears to independently elevate PTSD risk. These findings highlight the psychological burden of hysterectomy and support the need for targeted mental health screening in this population.

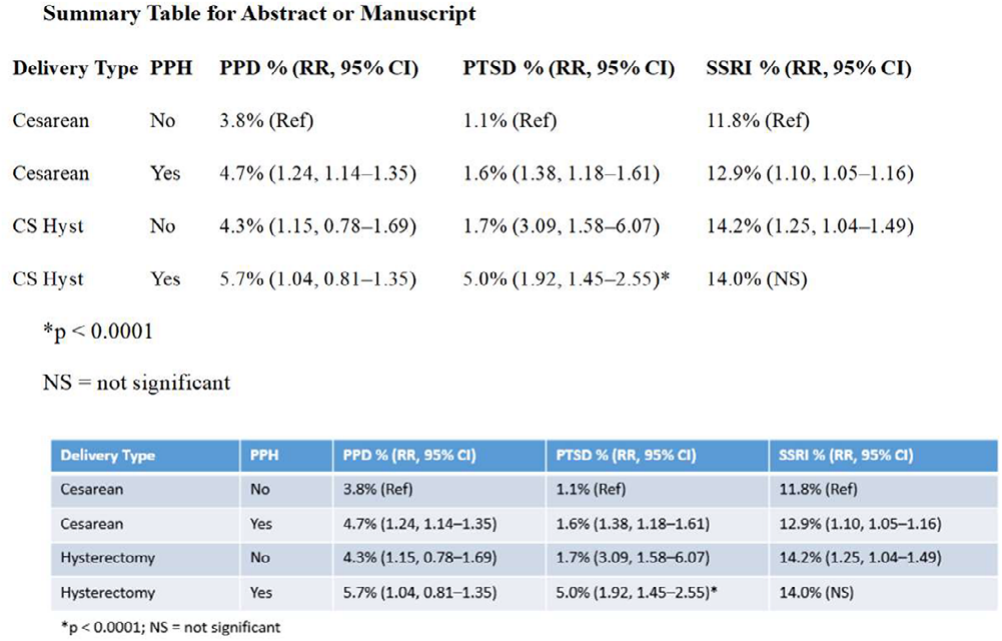

**10:30 AM – 12:00 PM**

## 174 | Comparing Preeclampsia characteristics in Singleton and Twin Pregnancies Using Machine Learning

Daniel Gabbai^1^; Itamar Gilboa^1^; Lee Reicher^2^; Yariv Yogev^3^; Emmanuel Attali^1^

^1^Lis Hospital for Women's Health, Tel Aviv Sourasky Medical Center, Lis Hospital for Women's Health, Tel Aviv Sourasky Medical Center, Tel Aviv; ^2^Lis Hospital for Women's Health, Tel Aviv Sourasky Medical Center, Tel Aviv, HaMerkaz; ^3^Lis Hospital for Women's Health, Tel Aviv Sourasky Medical Center Gray Faculty of Medicine, Tel Aviv University, Israel, Lis Hospital for Women's Health, Tel Aviv Sourasky Medical Center, Tel Aviv

**Objective**: To compare characteristics of preeclampsia (PET) in singleton and twin pregnancies using machine learning, and to determine differences in their relative importance.

**Study Design**: A retrospective cohort study at a university‐affiliated tertiary medical center (2012–2024), including all singleton and twin pregnancies. The primary outcome was PET, defined according to ACOG criteria. Characteristics included demographic, clinical, obstetric, and routine laboratory data available prior to delivery. Missing values were imputed using the median. Data were randomly split into training (70%) and testing (30%) sets. Separate XGBoost models were developed for each group, Feature importance was compared between groups using both model gain and SHAP (SHapley Additive exPlanations) value.

**Results**:

1. A total of 141,425 singleton pregnancies and 5574 twin pregnancies were included. PET occurred in 2453 (1.7%) singleton pregnancies and 522 (9.4%) twin pregnancies.
2. The models demonstrated high discrimination in both groups (AUC = 0.92 for singletons, 0.95 for twins).
3. Among twins, the most powerful predictors of PET (by model gain) were albumin/creatinine ratio (31%), pre‐pregnancy BMI (8%), platelet count (8%), neutrophil‐to‐lymphocyte ratio (7%), maternal age (7%), white blood cell count (6%), hemoglobin (5%), AST (5%), gestational weight gain (5%), and gestational age at delivery (4%).
4. While the same predictors were relevant in singletons, SHAP analysis revealed distinct differences in their effect distributions between groups.

**Conclusion**: Although singleton and twin pregnancies share similar predictors for PET, the magnitude and direction of their influence differ. These findings support the development of group‐specific risk assessment tools to enhance preeclampsia prediction and prevention strategies

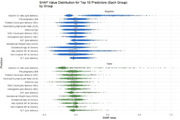

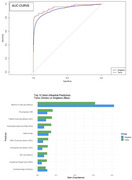

**10:30 AM – 12:00 PM**

## 175 | Fetal/Neonatal Alloimmune Thrombocytopenia and Childhood Neuropsychiatric Disorders: Role of Sex, Race/Ethnicity, and Gestational Age

Darios Getahun^1^; Morgan R. Peltier^2^; Theresa M. Im^1^; Vicki Y. Chiu^1^; Michael J. Fassett^3^

^1^Department of Research & Evaluation, Kaiser Permanente Southern California, Pasadena, CA; ^2^Psychiatry and Behavioral Health, Jersey Shore University Medical Center, Neptune City, NJ; ^3^Department of Obstetrics and Gynecology, Kaiser Permanente West Los Angeles Medical Center, Los Angeles, CA

**Objective**: Fetal and neonatal alloimmune thrombocytopenia (FNAIT), caused by a mismatch in human platelet antigens between mother and fetus, can lead to bleeding and bruising. Prompt treatment is essential to prevent life‐threatening intracranial hemorrhage and neuronal injury. We examined the association between FNAIT and mental, emotional, behavioral, or developmental (MEBD) disorders, and whether these vary by child's sex, race/ethnicity, and gestational age at birth in a large healthcare system.

**Study Design**: This retrospective cohort study included pregnant women and their children delivered at Kaiser Permanente Southern California (2011–2021). FNAIT cases were identified using ICD‐9/10 codes from inpatient and outpatient records for singleton births at ≥20 weeks gestation. MEBD outcomes, including attention‐deficit/hyperactivity (ADHD), autism spectrum (ASD), major depressive (MDD), generalized anxiety (GAD), and disruptive behavior (DBD) disorders, were identified using validated diagnostic codes. Modifying effects of child's sex, race/ethnicity, and gestational age were examined. Adjusted hazard ratios (aHRs) quantified associations.

**Results**: Among 281,628 pregnancies, 2755 FNAIT cases (9.8 per 1000) were identified. Compared to non‐FNAIT births, these cases were more likely to be preterm (52% vs. 22%, *p* < 0.001) and have any MEBD diagnosis (23.8% vs. 15.1%, *p* < 0.001). FNAIT was associated with increased risks of ASD (aHR 2.10, 95% confidence intervals [CI] 1.89–2.33), ADHD (aHR 1.23, 95% CI 1.04–1.44), and DBD (aHR 1.23, 95% CI 1.07–1.41), but not GAD or MDD. Stratified analyses showed significant associations for ASD (aHR 1.96, 95% CI 1.74–2.21) and ADHD (aHR 1.21, 95% CI 1.01–1.45) in boys, and ASD (aHR 2.68, 95% CI 2.16–3.33) and DBD (aHR 1.47, 95% CI 1.14–1.90) in girls. Risk varied by race/ethnicity and gestational age.

**Conclusion**: FNAIT exposure is associated with a higher burden of MEBDs in children, with risk differing by sex, race/ethnicity, and gestational age, underscoring the need for early surveillance and intervention.

**10:30 AM – 12:00 PM**

## 176 | Explainable AI–Enabled Prediction of Intrapartum Fetal Asphyxia: A Multicentre Study

Debjyoti Karmakar^1^; Lochana Mendis^2^; Brett Manley^3^; Jeanie Cheong^2^; Emerson Keenan^2^; Jim Holberton^4^; Enes Makalic^5^; Fiona Brownfoot^6^

^1^University of Melbourne/Mercy Hospital for Women, Mercy Hospital for Women/Heidelberg, Victoria; ^2^University of Melbourne, University of Melbourne, Victoria; ^3^University of Melbourne/Mercy Hospital for Women, University of Melbourne/Mercy Hospital for Women, Victoria; ^4^Mercy Hospital for Women, Mercy Hospital for Women, Victoria; ^5^Monash University, Monash University, Victoria; ^6^University of Melbourne/Mercy Hospital for Women, Mercy Hospital for Women/University of Melbourne, Victoria

**Objective**: To evaluate performance and generalizability of an interpretable AI model incorporating clinical heuristics and game theory–based feature selection, using minimally pre‐processed CTG signals to predict intrapartum fetal asphyxia.

**Study Design**: Diagnostic model development and external validation study. The model was trained on 36,792 labors from an Australian tertiary hospital (2010–2021) and validated on two independent cohorts: an Australian community hospital (*n* = 15,413; 2010–2021) and a European university hospital (*n* = 552; 2010–2012). Analysis followed a pre‐specified protocol.

< ![if !supportLineBreakNewLine] >

< ![endif] >

Singleton labors ≥36 weeks’ gestation with ≥15 minutes of interpretable CTG in the final hour. Exclusions included major congenital anomalies and >90% signal dropout.

Fetal heart rate and uterine activity signals processed via a Python pipeline to derive physiological metrics, artifact measures, and deep learning embeddings. Waveform features were engineered using clinician expertise. Shapley Additive Explanations guided feature selection. The final model was a gradient boosting classifier integrating the features.

The primary outcome was intrapartum fetal asphyxia, defined by a Delphi‐derived composite including hypoxic–ischemic encephalopathy, stillbirth, therapeutic hypothermia, low Apgar scores, prolonged resuscitation, or cord pH <7.00. The secondary outcome was cord pH <7.15.

**Results**: Asphyxia prevalence was 3.3%, 3.2%, and 7.1% across cohorts. In the development cohort, AUROC was 0.76 (95% CI, 0.72–0.80), with 68.5% sensitivity and 74.2% specificity. At matched clinician specificity (78.5%), model sensitivity improved from 35.8% to 60.2%. External AUROCs ranged between 0.73 and 0.83. The model achieved sensitivity for pH <7.15 of 83.9%.

**Conclusion**: This interpretable AI model significantly and consistently outperformed clinician judgment and generalized across diverse clinical settings. Its integration of domain knowledge and signal integrity supports further evaluation as a real‐time decision‐support tool in labor.

**10:30 AM – 12:00 PM**

## 177 | Novel Electrohysterography Simulation Model and Non‐Invasive Detection Method for Patients With High Postpartum Hemorrhage Risk

Deneb Zavala^1^; Kelly W. Yang^1^; Catalin S. Buhimschi^2^

^1^University of Illinois Chicago College of Medicine, Chicago, IL; ^2^University of Illinois Chicago, University of Illinois Chicago, IL

**Objective**: Postpartum hemorrhage (PPH) is a leading cause of maternal mortality, with uterine atony implicated in 70–80% of cases. Current detection methods—visual estimation, blood loss quantification, and manual palpation—are imprecise and delay treatment. We developed an in vitro non‐invasive electrohysterography (EHG) method to assess postpartum uterine contraction strength from the maternal abdominal surface, to potentially treat PPH sooner and ultimately prevent it.

**Study Design**: Our in vitro saline‐filled uterine model was designed to simulate and record EHG signals non‐invasively. Due to the lack of postpartum datasets, the Term‐Preterm Electrohysterogram Database (TPEHG), which contains well‐characterized laboring myoelectrical signals, was used for sensor calibration and validation. One hundred EHG signals spanning mild to strong contractions (∼0.7–2.3 µV peak‐to‐peak) were transmitted. A 0.3–3 Hz noise filter was applied, and fidelity was verified using Welch's method. Non‐contracting signals defined the noise floor; alert threshold was set at 3x baseline. Sensor outputs were compared to inputs to calculate linearity, gain, and signal‐to‐noise ratio (SNR). A Kruskal‐Wallis test evaluated the sensors’ ability to differentiate resting tone from active contractions.

**Results**: Linearity analysis showed a strong correlation between detected signals and input EHG signals across uterine contraction strengths (R^2^ > 0.98). Sensor gain was 3.60 counts/uV while noise baseline was 3.95 counts. Weakest contractions yielded an SNR of 7.5, confirming reliable low‐amplitude detection. The 11.9‐count alert threshold triggered warnings within 1 second, achieving 91.3% sensitivity, 8.7% false positives, and 8.9% specificity. There was a significant signal energy (RMS) difference between baseline and all contraction strengths (*p* < 0.01).

**Conclusion**: Our novel in vitro proof‐of‐concept EHG uterine model supports the feasibility of continuous, objective monitoring of ineffective uterine contractions and lays the groundwork for future in vivo validation to ultimately prevent PPH.

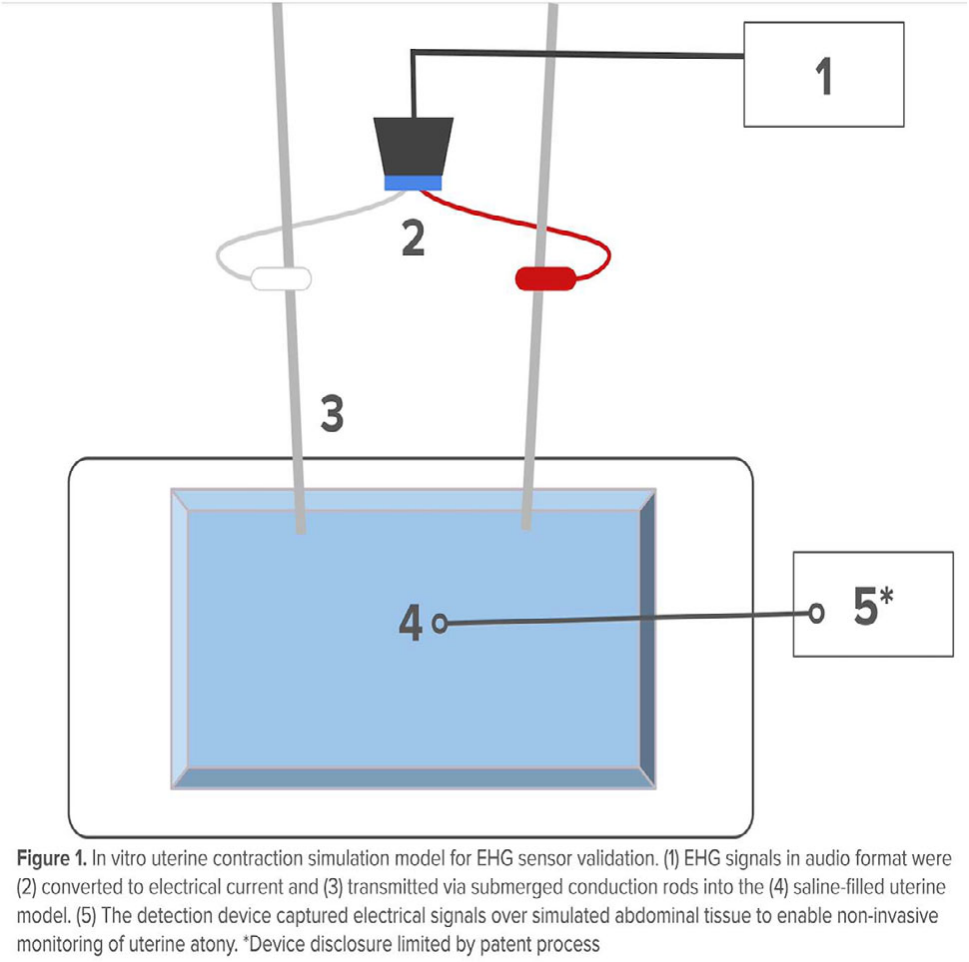

**10:30 AM – 12:00 PM**

## 178 | Cardiac Diastolic Dysfunction Predates Hypertensive Disorders of Pregnancy

Devangi D. Mahtani^1^; Harnoor Mann^2^; Laura Diab^3^; Poyee Tung^4^; Carlos El‐Tallawi^5^; Farah H. Amro^3^; Baha M. Sibai^3^

^1^Division of Maternal‐Fetal Medicine, Department of Obstetrics, Gynecology and Reproductive Sciences, McGovern Medical School at UTHealth Houston, UT Health Houston, TX; ^2^Department of Cardiology, McGovern Medical School at UTHealth Houston, UT Health Houston, TX; ^3^Division of Maternal‐Fetal Medicine, Department of Obstetrics, Gynecology and Reproductive Sciences, McGovern Medical School at UTHealth Houston, Houston, TX; ^4^Department of Cardiology, McGovern Medical School at UTHealth Houston, Houston, TX; ^5^Houston Methodist DeBakey Cardiology Associates, Houston Methodist DeBakey Cardiology Associates, TX

**Objective**: Hypertensive disorders of pregnancy (HDP), including preeclampsia are associated with long‐term cardiovascular risk. Prior studies suggest that left ventricular (LV) diastolic function may be impaired in affected individuals. However, it is unknown whether such cardiac changes are detectable before clinical onset of HDP. We aimed to determine whether echocardiographic markers of impaired diastolic function precede a diagnosis of HDP.

**Study Design**: We conducted a retrospective case‐control study of pregnant individuals who underwent transthoracic echocardiography. Participants were categorized into no‐HDP (*n* = 35) and hypertensive disorders of pregnancy (*n* = 25). Echocardiograms were obtained at an average of 34.7 days (range 4–113 days) prior to HDP diagnosis for other comorbidities. Diastolic function was assessed using mitral inflow velocities and tissue Doppler imaging of the septal and lateral mitral annulus. Multinomial logistic regression was performed to assess associations between echocardiographic parameters, adjusting for relevant clinical covariates such as age, body mass index, parity, and chronic hypertension.

**Results**: Despite patients having echocardiographic values in the normal range, the HDP group showed early signs of trend towards diastolic dysfunction. The lateral E’ was significantly lower in the HDP group (12.6 ± 3.0 cm/s) compared to the no‐HDP group (15.3 ± 7.2 cm/s, *p* = 0.05). Additionally, Grade I diastolic dysfunction was more frequent in HDP cases (48% vs. 23%, *p* = 0.04). Other diastolic indices, such as septal E’ and E/A ratio, showed a non‐significant trend towards impairment. Systolic function as evidenced by left ventricular ejection fraction remained preserved in both groups.

**Conclusion**: Subclinical diastolic dysfunction, as evidenced by reduced lateral E’ velocities and higher frequency of Grade I diastolic dysfunction is present prior to the onset of HDP. These subtle changes occur weeks before the clinical diagnoses suggesting that cardiac remodeling predates preeclampsia.

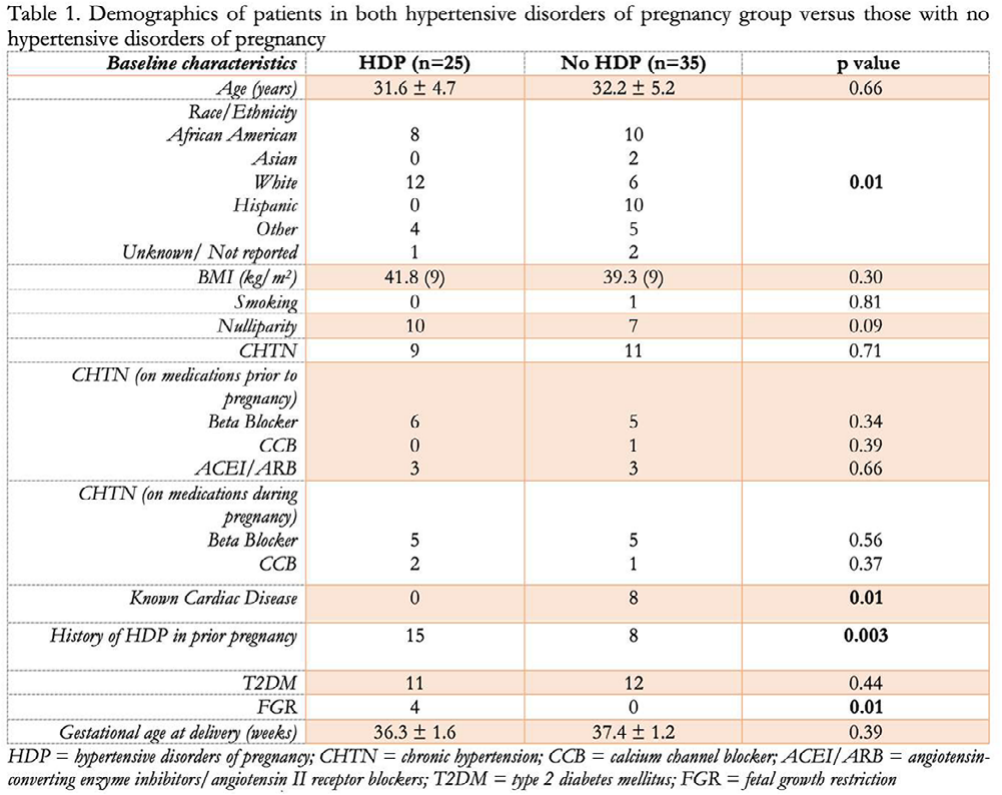

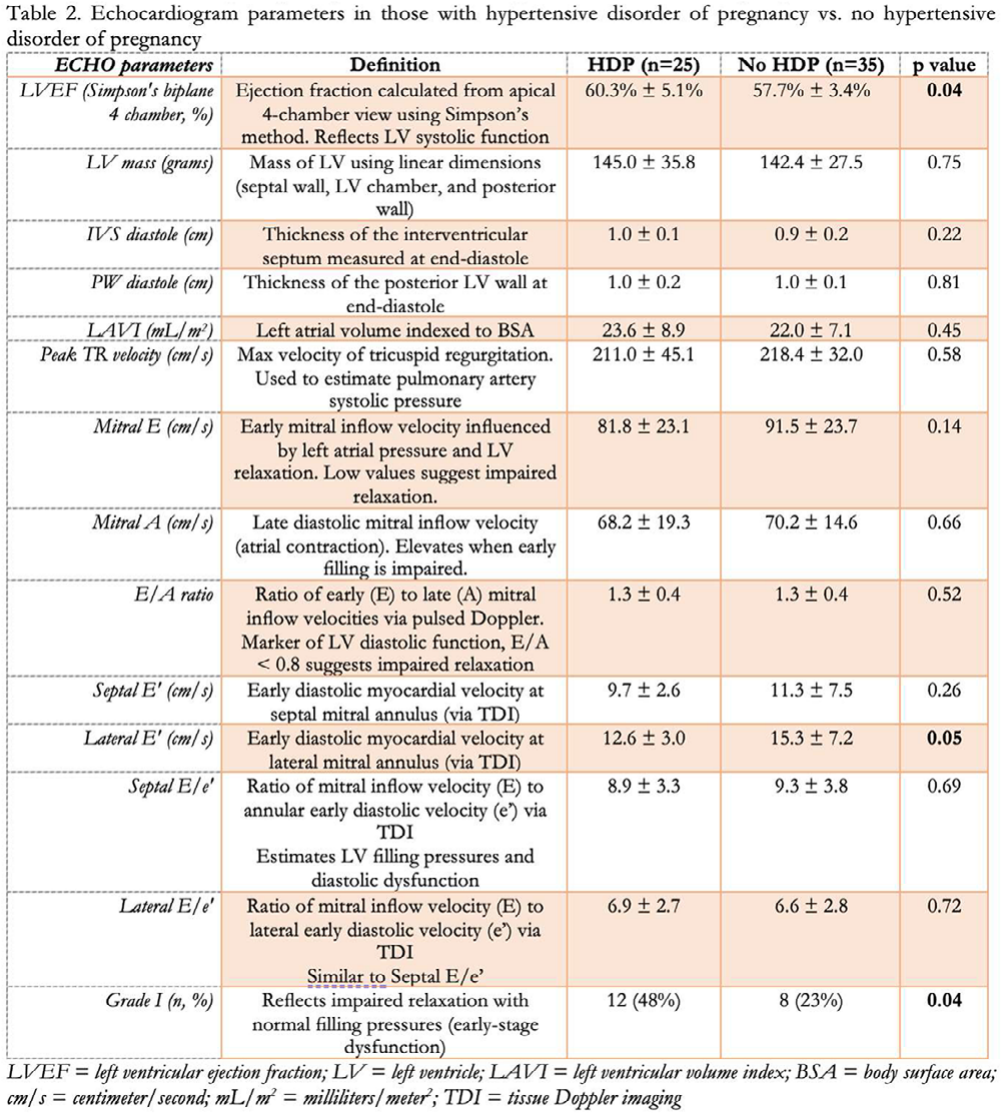

**10:30 AM – 12:00 PM**

## 179 | Determining the Optimal Pre‐Operative Hemoglobin to Prevent Transfusion During Cesarean Hysterectomy for Placenta Accreta Spectrum

Devesh Naidoo^1^; Vineet Shrivastava^2^; Megan C. Oakes^3^

^1^MemorialCare Miller's Children's and Women's Hospital, Long Beach, CA; ^2^MemorialCare Miller Children's and Women's Hospital Long Beach, Long Beach, CA; ^3^MemorialCare Miller Children's and Women's Hospital, Long Beach, CA

**Objective**: Placenta accreta spectrum (PAS) is associated with high rates of severe maternal morbidity (SMM), largely due to hemorrhage requiring transfusion. While there is consensus regarding aggressive correction of anemia, there is a lack of data regarding specific preoperative hemoglobin (Hgb) targets. We aimed to quantify the risk of transfusion based on preoperative anemia status and identify the optimal pre‐operative Hgb level to avoid transfusion.

**Study Design**: A retrospective cohort study of patients with antenatally diagnosed PAS undergoing cesarean hysterectomy (c‐hyst) at >20 weeks’ gestation from 1/2010–9/2024 at a single, tertiary care center. The exposure was pre‐operative anemia (<11 g/dL or <10.5 g/dL for the 3rd and 2nd trimesters, respectively). The primary outcome was red blood cell (RBC) transfusion. Secondary outcomes included transfusion of >4 units RBCs, ICU admission, disseminated intravascular coagulopathy (DIC), and post‐operative length of stay. The Youden index was used to identify the ideal Hgb value to avoid RBC transfusion and the area under receiver operator curve (AUROC) was compared to the standard anemia definition (<11 g/dL).

**Results**: Among 77 patients who underwent c‐hyst for PAS, 38 (49%) had pre‐operative anemia and 39 (51%) did not. Average gestational age at delivery between groups was similar at ∼32 weeks. RBC transfusion was significantly higher among those with pre‐operative anemia (97.37 vs 71.97%; RR 1.35, 95% CI 1.11–1.66) (Table 1). There were no significant differences between the groups for secondary outcomes. The “optimal” pre‐operative Hgb concentration to avoid a transfusion was 11.35 g/dL, which performed significantly better than the standard anemia definition (AUC 0.82, 95% CI 0.72‐0.91 vs AUC 0.75, 95% CI 0.65‐0.85; *p* = 0.001) (Figure 1).

**Conclusion**: Pre‐operative anemia increases the risk of RBC transfusion at time of cesarean hysterectomy for PAS patients. Utilizing an even higher Hgb value to target when optimizing patients with PAS prior to surgery may avoid intra‐ or post‐operative transfusion‐related SMM.

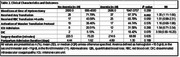

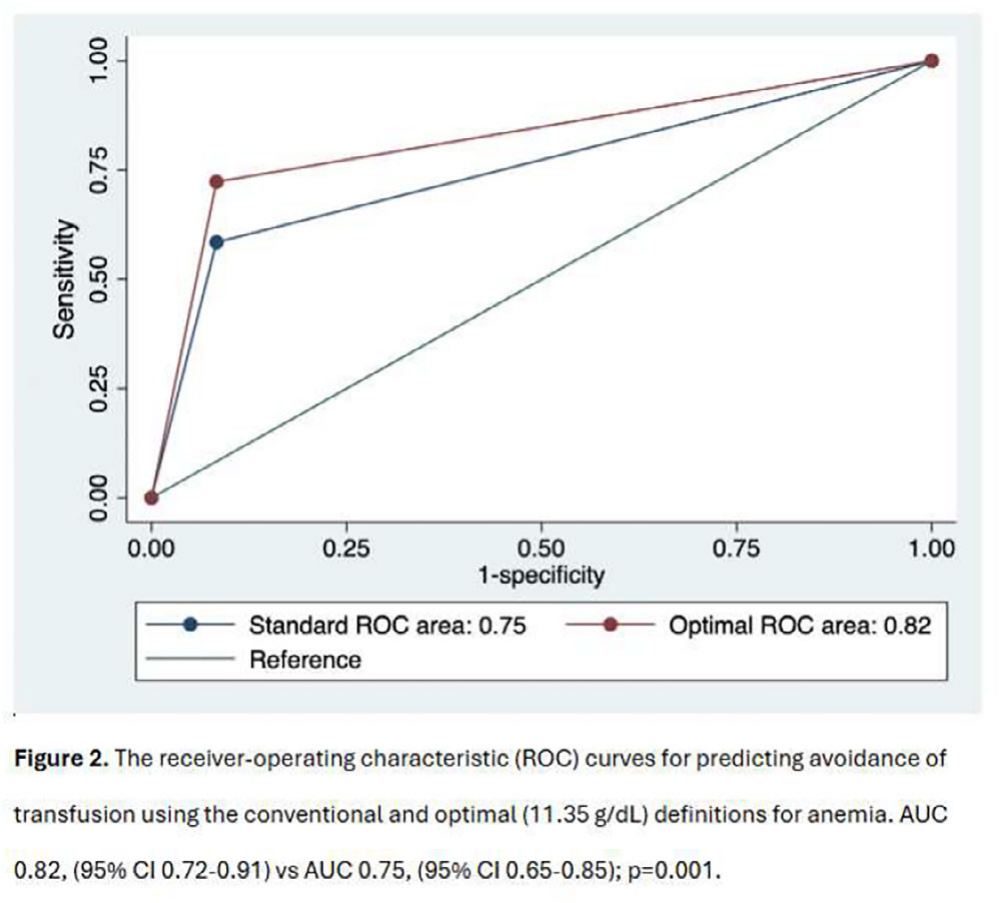

**10:30 AM – 12:00 PM**

## 180 | Maternal Preeclampsia and Offspring Neurological Morbidity: A Sibling‐Matched Cohort Study

Dor Marciano^1^; Tamar Wainstock^2^; Eyal Sheiner^3^

^1^Soroka university medical center, Soroka, HaDarom; ^2^Department of Epidemiology, Biostatistics and Community Health Sciences, Faculty of Health Sciences, Ben‐Gurion University of the Negev, Beer Sheva, HaDarom; ^3^Soroka University Medical Center, Faculty of Health Sciences, Ben‐Gurion University of the Negev, beer sheva, HaDarom

**Objective**: Emerging evidence links maternal preeclampsia to offspring neurological disorders; however, many studies are limited by residual confounding. This study used a sibling‐matched design to control for unmeasured familial and environmental factors and assess whether preeclampsia is independently associated with long‐term neurological morbidity, including autism.

**Study Design**: We conducted a retrospective population‐based cohort study of singleton siblings born to the same mother, where one sibling was exposed to preeclampsia and the other was not. Neurological diagnoses during childhood—including autism spectrum disorder (ASD), epilepsy, and ADHD—were assessed using Kaplan‐Meier survival analysis and generalized estimating equation (GEE) models adjusted for maternal age and preterm delivery.

**Results**: Among 8544 siblings (4,272 exposed to preeclampsia), there was no significant difference in cumulative neurological‐related hospitalizations between groups (log‐rank *p* = 0.217). Crude hospitalization rates were 4.0% in the exposed group vs. 3.4% in the unexposed (OR = 0.86; 95% CI, 0.70–1.09; *p* = 0.237). Adjusted analysis confirmed no significant association between preeclampsia exposure and long‐term neurological morbidity (aHR = 0.94; 95% CI, 0.76–1.17; *p* = 0.305). Specifically, no increased risk for autism was observed.

**Conclusion**: In this sibling‐matched cohort study, maternal preeclampsia was not associated with increased long‐term neurological morbidity in offspring. These findings challenge prior associations and suggest earlier studies may have been influenced by unmeasured confounding. The absence of increased autism risk is particularly reassuring. Sibling‐matched analyses offer a valuable approach to strengthen causal inference in perinatal epidemiology.

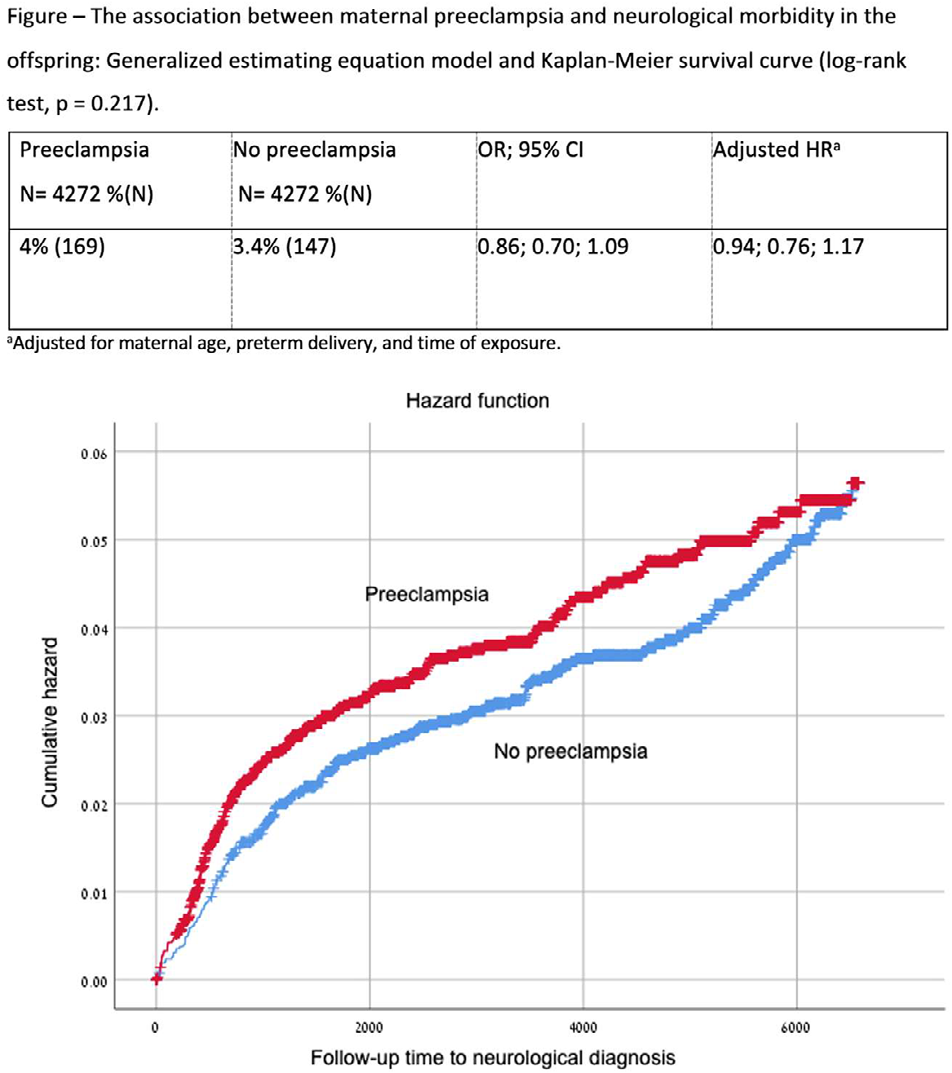

**10:30 AM – 12:00 PM**

## 181 | Antenatal Corticosteroid Exposure and Risk of Childhood Allergy

Sivan Farladansky Gershnabel^1^; Yael Wolf^2^; Matat Koren^3^; Tal Biron Shental^4^; Dorit Ravid^1^; Idit Lachover‐Roth^3^

^1^Department of Obstetrics and Gynecology, Meir Medical Center, Kfar Saba, HaMerkaz; ^2^Faculty of Medical and Health Sciences, Tel Aviv University, Kfar Saba, HaMerkaz; ^3^Meir Medical Center, Kfar Saba, HaMerkaz; ^4^Meir Medical Center, Meir Medical Center, HaMerkaz

**Objective**: Antenatal corticosteroids (ACS) are routinely used to enhance fetal lung maturity in threatened preterm birth. However, their immunomodulatory effects may impact fetal immune development and increase susceptibility to allergic disease. As ACS use expands to borderline indications, understanding long‐term safety is critical. This study evaluated whether ACS exposure is associated with increased risk of allergic disease in early childhood and whether this association persists through age 3.

**Study Design**: Secondary analysis of a prospectively followed cohort of 2252 mother–infant dyads delivered between 2015–2022. ACS exposure was defined as intramuscular corticosteroid administration during pregnancy. Maternal and neonatal data were extracted from electronic records. Allergic outcomes—atopic dermatitis, food allergy, and hyperreactive airway disease (HRAD)—were assessed through structured pediatric follow‐up at ages 1, 2, and 3. Associations were evaluated using chi‐square and multivariable logistic regression adjusting for maternal age, parity, gestational age, birthweight, gender, delivery mode, hypertensive disorders, ethnicity, and residence. Due to attrition, 2251 infants were followed at year 1, 1901 at year 2, and 1726 at year 3.

**Results**: Among 1241 ACS‐exposed and 1011 unexposed infants, the exposed group had lower gestational age (37.6 vs. 39.1 weeks), lower birthweight (3091 g vs. 3298 g), and higher rates of cesarean and preterm birth. At age 1, atopic comorbidity was more prevalent in ACS‐exposed infants (5.3% vs. 3.1%, *p* = 0.015), remaining significant after adjustment (aOR 1.89; 95% CI 1.22–2.92; *p* = 0.004). No significant difference was observed at age 2, but by age 3, prevalence was again higher (7.1% vs. 1.4%, *p* = 0.002; aOR 1.55; 95% CI 1.06–2.28).

**Conclusion**: ACS exposure was independently associated with increased risk of allergic disease in early childhood. While ACS remains vital for preterm neonatal outcomes, its long‐term immunologic impact warrants further research and careful risk‐benefit consideration.

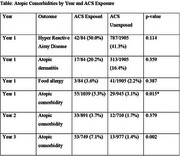

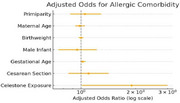

**10:30 AM – 12:00 PM**

## 182 | Postpartum Diabetes Medication Titration Protocol: Effect on Inpatient Efficiency and Glycemic Management Outcomes

Drew M. Hensel^1^; Katherine Massa^2^; Kelley Williams^3^; Cynthia Herrick^3^; Amanda C. Zofkie^2^; Sydney M. Thayer^4^; Nandini Raghuraman^2^; Megan L. Lawlor^2^

^1^Washington University School of Medicine, SAINT LOUIS, MO; ^2^Washington University School of Medicine, St. Louis, MO; ^3^Washington University School of Medicine, Saint Louis, MO; ^4^Washington University School of Medicine, Washington University School of Medicine, MO

**Objective**: Insulin resistance declines postpartum, necessitating timely medication adjustments in those with diabetes. On our Labor & Delivery (L&D) unit, medication regimen decision‐making was delaying transfer to postpartum (PP). In response, we implemented a protocol to calculate postpartum medication regimens among patients with pre‐existing diabetes during the antepartum period. Our objective was to evaluate if the protocol improved efficient PP patient transfer and improved inpatient and outpatient diabetes outcomes.

**Study Design**: This was a pre‐post quality improvement study at a tertiary‐care academic hospital. The study included all delivering patients with pre‐existing diabetes separated into two cohorts pre‐ (Feb 2023–Feb 2024) and post‐ (Apr 2024–April 2025) protocol implementation. The primary outcome was delayed PP transfer, defined as post‐delivery time on L&D >1 standard deviation above the mean (>3.66 hours based on institution‐specific transfer time of 2.9 +/− 0.76 hours). Outcomes were compared between the cohorts using bivariate analyses and logistic regression.

**Results**: There were 124 patients in each cohort and 31% had type 1 diabetes. The post‐implementation cohort had decreased odds of delayed PP transfer compared to pre‐ implementation after adjusting for insulin pump use, hypertensive disorders of pregnancy, and postpartum hemorrhage (aOR 0.50 [0.26–0.95]). This decrease in delayed PP transfer was not seen when stratified by diabetes type (Table 1). There was a higher rate of documented PP medication plans and outpatient postpartum glycemic control review in the post‐implementation cohort (Table 2). There were no differences in inpatient postpartum hyper‐ or hypoglycemia or postpartum endocrinology or primary care follow up.

**Conclusion**: Enacting a postpartum diabetes medication protocol facilitated more efficient PP transfers and improved PP glycemic control follow up, while not increasing rates of inpatient postpartum hyper‐ or hypoglycemia. This serves as a model to improve L&D efficiency at other high acuity tertiary care centers.

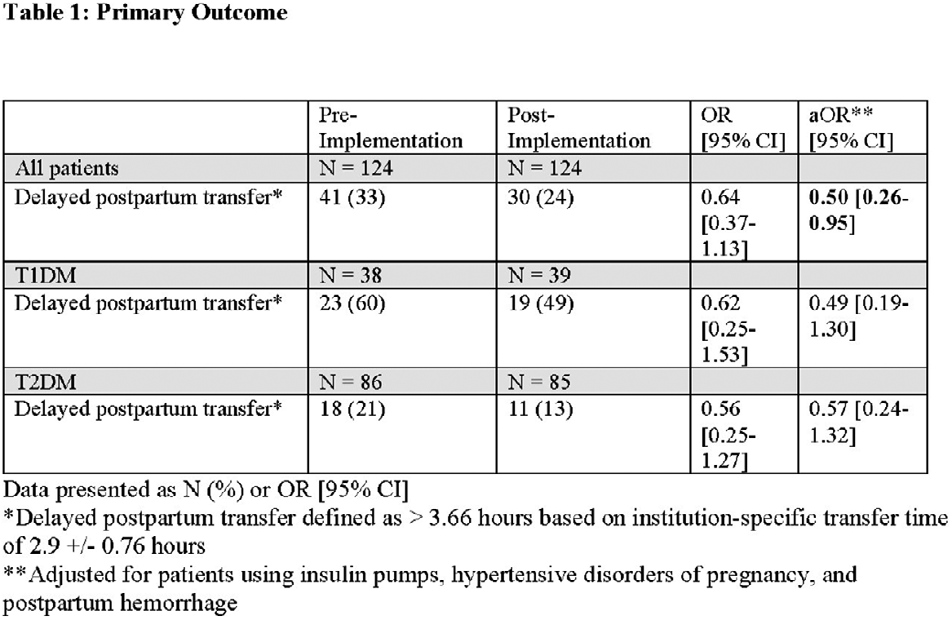

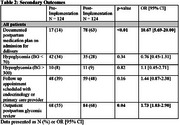

**10:30 AM – 12:00 PM**

## 183 | The Impact of E‐Cigarettes on Pregnancy & Childhood Health Outcomes–The ECHO Study

Eibhlín F. Healy^1^; Abigail O'Connell^2^; Marah Shaikh Yousef^3^; Adele Reddin^4^; Lydia Healy^5^; Gohit Tankala^6^; Anne Doolan^7^; Patricia Fitzpatrick^8^; Cecily Kelleher^8^; Tim Coleman^9^; Kate Frazer^8^; Jennifer Walsh^10^; Shane Higgins^8^; Fergal D. Malone^11^; Eoin O'Curráin^12^; Michael Boyle^13^; Michelle Downes^8^; Desmond Cox^14^; Carmen Regan^15^

^1^Royal College of Surgeons in Ireland, Royal College of Surgeons in Ireland, Dublin; ^2^UCD, UCD, Dublin; ^3^RSCI, Royal College of Surgeons, Dublin; ^4^The Rotunda Hospital, Dublin, Dublin; ^5^The Coombe Hospital, The Coombe Hospital/Dublin, Dublin; ^6^University College Dublin, Dublin, Dublin; ^7^The Coombe Hopsital, Dublin, Dublin; ^8^UCD, Dublin, Dublin; ^9^University of Nottingham, Nottingham, England; ^10^National Maternity Hospital, National Maternity Hospital, Dublin; ^11^Royal College of Surgeons in Ireland, Dublin, Dublin; ^12^Children's Health Ireland, Dublin, Dublin; ^13^Rotunda Hospital, Rotunda Hospital, Dublin; ^14^UCD/Children's Health Ireland, Children's Health Ireland, Dublin, Dublin; ^15^RCSI/The Coombe Hospital, Dublin, Dublin

**Objective**: E‐cigarette use is increasingly prevalent in pregnancy, yet maternal and infant outcomes remain poorly understood with few longitudinal studies examining outcomes. The ECHO Study (The impact of E‐Cigarettes on pregnancy and Childhood Health Outcomes–NCT: 06297005) ‐ is a prospective, multicentre, observational cohort study of women and fetuses exposed to E‐cigarettes [1].

**Study Design**: Three groups of pregnant women were recruited; smokers, e‐cigarette users and non‐users. Their exposure status was objectively determined using specific biomarkers (exhaled breath carbon monoxide and urine cotinine analysis). Obstetric outcomes examined included birthweight, small for gestational age and gestation at delivery.

**Results**: Outcomes for 684 women are presented with 307 controls, 200 smokers, 143 vapers and 34 vaper/smokers. Chi‐squared tests were used for comparison of proportions, and Wilcoxan rank‐sum tests were used for non‐parametric data. Demographic and obstetric outcomes are described in Tables 1 and 2.

Vaping mothers had infants of similar birthweight to controls (*p*‐value 0.8) and their babies were significantly heavier than those of smoking mothers (*p* = 0.05). Smokers had significantly more babies <10^th^ percentile for gestational age than vapers (*p*‐value <0.0001). No significant difference in median gestational age  , rate of preterm birth or admission to NICU was found (Table 2).

**Conclusion**: Unlike cigarette smoking, E‐cigarette usage in pregnancy is not associated with an increase in SGA babies. This is a clinically relevant finding and contrasts with earlier studies which interpret large datasets. The strength of our study lies in its prospective design and use of objective biomarker determination of exposure groups providing reliable confirmation of cigarette consumption and E‐cigarette usage.

Reference:

1. Healy EF, O'Connell A, Yousef MS, Reddin A, Boyle M, Coleman T, et al. The impact of electronic cigarettes on pregnancy and childhood health outcomes: the ECHO study—a protocol for a multicentre, prospective, observational, cohort. Archives of Gynecology and Obstetrics. 2025:1–9.

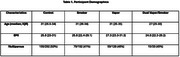

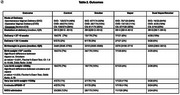

**10:30 AM – 12:00 PM**

## 184 | Association Between Indication for Previous Cesarean Delivery and the Risk for Uterine Rupture in TOLAC

Einat Tako^1^; Itai Atar^1^; Itamar Gilboa^2^; Daniel Gabbai^2^; Yariv Yogev^3^; Emmanuel Attali^2^

^1^Lis Hospital for Women's Health, Tel Aviv Sourasky Medical Center, Tel Aviv, Israel, Tel Aviv, Tel Aviv; ^2^Lis Hospital for Women's Health, Tel Aviv Sourasky Medical Center, Lis Hospital for Women's Health, Tel Aviv Sourasky Medical Center, Tel Aviv; ^3^Lis Hospital for Women's Health, Tel Aviv Sourasky Medical Center Gray Faculty of Medicine, Tel Aviv University, Israel, Lis Hospital for Women's Health, Tel Aviv Sourasky Medical Center, Tel Aviv

**Objective**: To determine whether the indication for previous cesarean delivery (CD) is associated with the risk of uterine rupture in women undergoing trial of labor after cesarean (TOLAC).

**Study Design**: A retrospective cohort study was conducted at a single university‐affiliated tertiary center. The study included all women with one previous CD who attempted a TOLAC at our institution between 2012–2024, regardless of whether the trial ended in vaginal birth or intrapartum CD. Among all women who underwent TOLAC after one prior CD, only those with a clearly documented indication for the prior CD were eligible for inclusion. Women with a nonviable fetus or contraindications to TOLAC were excluded.

Indications for previous CD were categorized into four groups:

1. Antepartum indications–mal‐presentation, maternal request and others non‐ labor indications
2. Suspected Fetal distress–non‐reassuring fetal heart rate, cord prolapse, reduced fetal movements
3. First‐stage dystocia–dysfunctional labor, failed induction
4. Second stage dystocia–arrest of descent, failed vacuum

Risk factors were assessed using multivariable logistic regression analysis.

**Results**:

1. During the study period, 147,045 women delivered at our center. Of these, 1806 women met inclusion criteria; 91 women (1.6%) were diagnosed with uterine rupture.
2. Prior CD due to second‐stage dystocia was significantly more common in the rupture group (12.1% vs. 3.2%, *p* < 0.001)
3. In multivariate analysis, second‐stage dystocia was an independent predictor for uterine rupture (OR 3.9, 95% CI 1.14–13.37, *p* = 0.03)
4. No other indications were significantly associated with uterine rupture. First‐stage dystocia was excluded from multivariable analysis due to limited number of events (*n* = 4). Time interval between prior CD and TOLAC did not differ significantly between groups (2.7 vs. 2.6 years, *p* = 0.31)

**Conclusion**: Second‐stage dystocia as an indication for previous CD was independently associated with a nearly fourfold increased risk of uterine rupture during TOLAC. This indication‐specific risk warrants explicit consideration in delivery planning and counseling

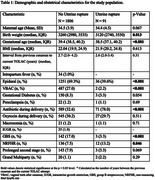

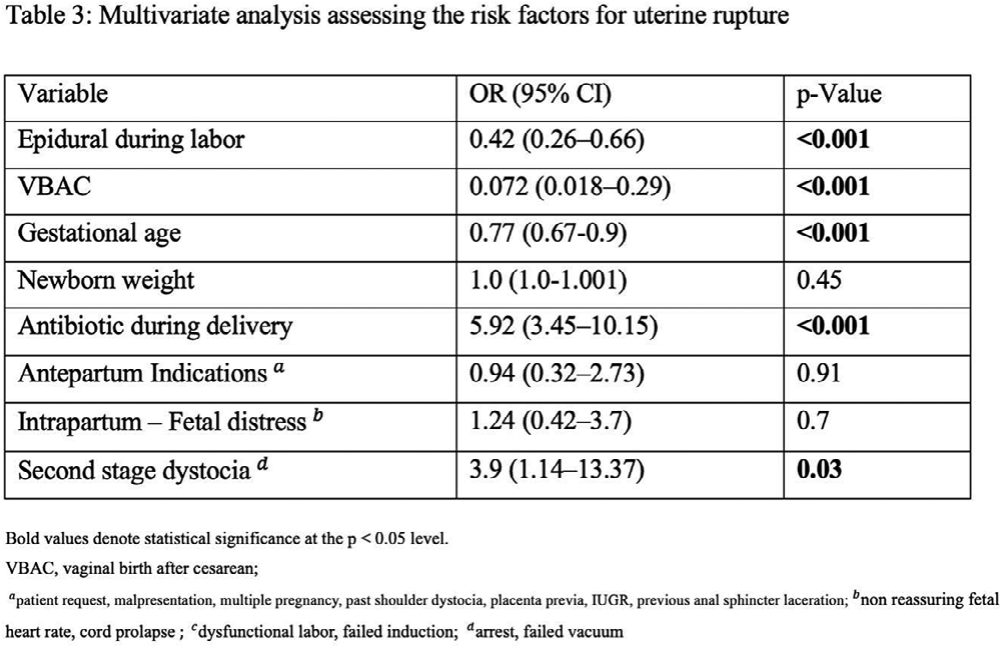

**10:30 AM – 12:00 PM**

## 185 | Obstetric Emergency Response: Predictability and Long‐Term Impact

Elena Lands^1^; Carrie Bennett^2^; Jes Eskridge^2^; Anna Binstock^2^; Jacob Larkin^2^

^1^University of Pittsbugh Medical Center Magee‐Womens Hospital, Pittsburgh, PA; ^2^UPMC Magee‐Womens Hospital, Pittsburgh, PA

**Objective**: Development of an obstetric emergency response (OER) team is promoted by ACOG as an important patient safety initiative. Despite the prominent role of OER teams in modern practice, optimal operational protocols and criteria for activation remain unknown. Moreover, the impact of the OER on an individual's long‐term mental health is not well understood. We aimed to: 1) identify characteristics associated with OER activation and 2) gauge its impact on postpartum mood disorders (PPMD).

**Study Design**: Retrospective cohort study of 36,649 births from January 2021 through April 2024 at one tertiary academic medical center. Multivariate logistic regression was used to develop a predictive model for OER activation. In the subset delivered between May 2022 and April 2023, rates of PPMD and referral for mental health care were compared in births with and without OER.

**Results**: OER rates significantly increased annually with 1 in 25 births affected in 2024 (Figure 1, *p* < 0.001). OER was significantly more likely for African‐American patients (aOR 1.5, 95% CI 1.3–1.73), those with spontaneous labor (aOR 1.36, 95% CI 1.17–1.59), and preterm births (aOR 2.98, 95% CI 2.57–3.47). OER was not associated with insurance status, hypertension, diabetes, or smoking. The developed model was a poor predictor for activation of OER with an AUC of 0.65 (Figure 2). Those with OER were significantly more likely to be diagnosed with new mood disorders within 8 weeks postpartum (OR 2.65, 95% CI 1.72–4.09), to be referred to therapy (OR 12.41, 95% CI 6.67–23.11), and to attend therapy (OR 2.82, 95% CI 1.3–6.13).

**Conclusion**: While at least 4% of delivering patients experience an OER, activation is difficult to predict and is often associated with new mood disorders and utilization of mental health services. This underscores a crucial need for robust evidence not only to optimize OER but also to develop strategies to prepare patients for its possibility and to reduce associated trauma.

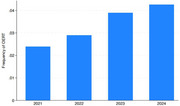

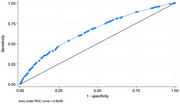

**10:30 AM – 12:00 PM**

## 186 | Geographic and Demographic Risk Factors for Stillbirth in Hawai'i

Elizabeth Mercer^1^; Kyle M. Ishikawa^1^; April Binari^1^; Ashley Graham^2^; Corrie Miller^1^; Hyeong J. Ahn^3^; Men‐Jean Lee^1^

^1^University of Hawaii, Honolulu, HI; ^2^Pacific Basin Telehealth Resource Center, Honolulu, HI; ^3^University of Hawaii John A Burns School of Medicine, Honolulu, HI

**Objective**: Stillbirth disproportionately affects Native Hawaiian and Pacific Islander (NHPI) populations in Hawaiʻi. While statewide rates are known, maternal risk factors and geographic variability remain unclear. We aimed to identify demographic and geographic predictors of stillbirth in Hawaiʻi, including maternal age, ethnicity, English proficiency, and travel burden to the delivery location.

**Study Design**: We conducted a retrospective cohort study using Hawaiʻi birth registry data from 2014 to 2024, including all hospital deliveries. Each birth was linked to the home ZIP code, maternal demographics, and delivery location. Stillbirth rates per 1000 births were calculated overall and by ZIP code. Univariate and multivariable logistic regression were used to identify independent predictors. Stillbirths were also mapped by ZIP code to visualize geographic disparities.

**Results**: Among 116,125 deliveries, 808 were stillbirths (6.96 per 1000). In the multivariable analysis shown in Table 1, advanced maternal age (OR: 1.49, *p*‐value: < 0.001), NHPI ethnicity (OR: 1.40, 2.09, *p*‐value: 0.004, <0.001 respectively), and interisland delivery (OR: 4.38, *p*‐value: <0.001) were significantly associated with stillbirth. The heat map in Figure 1 revealed clusters of high stillbirth rates in rural ZIP codes on Hawaiʻi Island, including 96725, 96737, and 96783. These areas shared characteristics such as higher NHPI populations, lower population density, limited OB care access, and longer travel times to delivery hospitals.

**Conclusion**: Stillbirth in Hawaiʻi is strongly influenced by both maternal and geographic factors. Disparities are most pronounced in rural communities with reduced healthcare infrastructure and high NHPI populations. In response, we are launching a pilot remote non‐stress testing (NST) program in these high risk areas to expand access to antenatal surveillance to reduce the risk of stillbirth across the state.

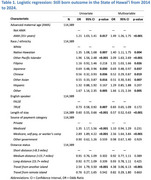

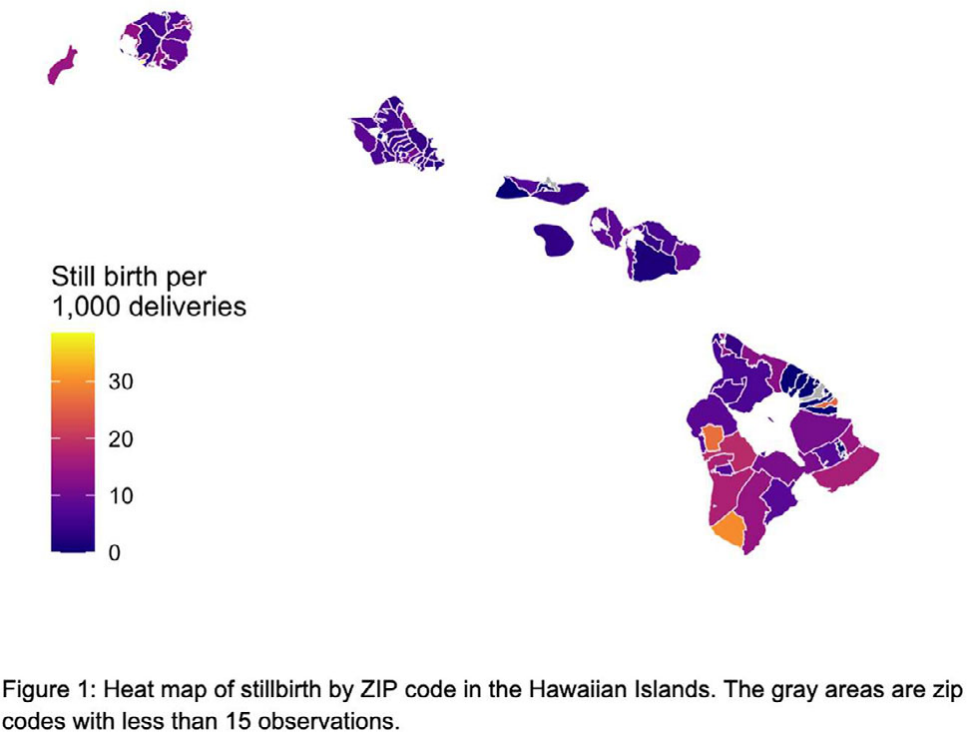

**10:30 AM – 12:00 PM**

## 187 | Risk of Adverse Perinatal Outcomes After Low‐Risk NIPT and Elevated Nuchal Translucency >95%

Ellie Marlor^1^; Genevieve Mazza^1^; Femitan Ajayi^1^; Jenny Chang^2^; Jonathan Steller^1^

^1^UC Irvine Medical Center, Orange, CA; ^2^University of California, Irvine, Irvine, CA

**Objective**: Fetal nuchal translucency (NT) ultrasound and maternal serum noninvasive prenatal testing (NIPT) are important components of early pregnancy risk assessment. It is widely accepted that an abnormal nuchal translucency (NT) is associated with increased risk for major fetal structural anomalies, genetic defects, and fetal loss. However, there is a paucity of evidence to guide counseling regarding perinatal outcomes in the setting of low‐risk NIPT. Thus, the purpose of this study is to evaluate the association between an elevated NT and the risk for adverse perinatal outcomes after low‐risk NIPT.

**Study Design**: This was a retrospective cohort study of patients who received an NT ultrasound at our tertiary care center between 1/2021–12/2024. Those with an NT greater than the 95th %ile and a low risk NIPT were included. Data on delivery timing, mode of delivery, and maternal and fetal complications were collected. The cohort was divided into two groups: NT 95‐98th %ile and NT >99th %ile. Variables were compared using chi‐square, Fisher's exact, or Student's T‐tests as appropriate.

**Results**: 96 patients met inclusion criteria. 54 patients had an NT between 95–98th %ile and 42 had an NT greater than the 99th%ile. The overall rate of preterm birth in the cohort was 17.6% (11.3% in those with NT 95–98th %ile; 26.3% in those with NT >99th %ile; *p* = 0.0639). The overall rate of Cesarean delivery was 39.8% (38.5% in those with NT 95–98%ile; 41.7% in those with NT >99%ile; *p* = 0.7926). 22.9% of the cohort had at least one fetal complication (13% in those with NT between 95–98%ile, 35.7% in those with NT >99%ile; *p* = 0.0085).

**Conclusion**: Despite a low‐risk NIPT, an elevated NT >95%ile is associated with increased risk for preterm birth, Cesarean delivery, and fetal complications at rates higher than national average. These rates are even more striking for patients with an NT >99%ile. These findings underscore that patient‐centered counseling should include a thorough discussion of the increased perinatal and delivery‐related risks after an abnormal NT ultrasound.

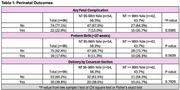

**10:30 AM – 12:00 PM**

## 188 | Confronting Disparities: Piloting an Individualized Clinician Equity Dashboard for Labor Outcomes

Emily Gleason^1^; Sindhu K. Srinivas^2^; Abigail Wolf^3^; Rebecca F. Hamm^4^

^1^Department of Obstetrics and Gynecology, Northwestern Medicine, Chicago, IL; ^2^Department of Obstetrics and Gynecology, Perelman School of Medicine, Maternal and Child Health Research Center, Philadelphia, PA; ^3^Pennsylvania Hospital, Philadelphia, PA; ^4^Perelman School of Medicine, University of Pennsylvania, Philadelphia, PA

**Objective**: An Equity in Labor Outcomes Dashboard can directly provide clinicians with individual performance data stratified by patient race with the goal of reducing disparities. Building on prior participatory mixed methods work, we assessed the feasibility, acceptability, and perceived clinical impact of implementing this Dashboard in a pilot cohort.

**Study Design**: This prospective study enrolled ten obstetric clinicians (attending and trainee physicians; CNMs) at 2 units to receive bimonthly Dashboards via email from 9/2024–2/2025. Reports demonstrated individual outcomes (cesarean, hemorrhage, transfusion, ICU, and composite maternal morbidity) by patient race, benchmarked against unit aggregates (Figure). Outcomes were attributed to clinicians who wrote ≥1 labor note and/or attended delivery. Coaching sessions with quality leads were offered bimonthly. Participants completed the validated Feasibility and Acceptability of Intervention Measures [FIM/AIM; Likert 4–20] and qualitative interviews guided by the Consolidated Framework for Implementation Research 2.0.

**Results**: All 30 planned individualized Dashboards (3/participant) were viewed at least once. Dashboards were considered feasible (16/20 IQR[16–17]) and acceptable (16.5/20 IQR[15–20]). Qualitative data (Table) revealed high acceptability, with participants expressing motivation for equity and appreciating opportunities for self‐reflection. Most had no prior sense of individual performance by race. However, some reported negative feelings (“defensive,” “embarrassed”), and questioned utility, citing small sample sizes and attribution concerns, limiting perceived ability to alter care. Suggestions included making the Dashboard cumulative and interactive. Departmental expansion was viewed favorably, while national rollout raised concerns about punitive action and legal risk.

**Conclusion**: This pilot demonstrates the feasibility and acceptability of an individualized Equity in Labor Outcomes Dashboard. These data underscore opportunities for refinement and the need for a larger‐scale implementation study powered to examine impact on racial disparities.

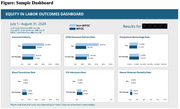

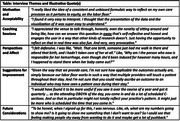

**10:30 AM – 12:00 PM**

## 189 | Social Risks‐Focused Lifestyle Intervention to Reduce Preeclampsia in Nulliparous Black Women: A Pilot RCT

Erica Marion^1^; Amandla Gales^1^; Abbey Kruper^1^; Alyssa M. Hernandez^2^; Melodee Liegl^1^; Amy Y. Pan^2^; Anna Palatnik^3^

^1^Medical College of Wisconsin, Milwaukee, WI; ^2^Medical College of Wisconsin, Medical College of Wisconsin, WI; ^3^Medical College of Wisconsin, MILWAUKEE, WI

**Objective**: To evaluate the feasibility and preliminary efficacy of social risks‐focused lifestyle intervention on preeclampsia risk in pregnant Black nulliparas.

**Study Design**: In this randomized controlled trial, pregnant nulliparous non‐Hispanic Black women aged ≥18 years and ≤16 weeks’ gestation were randomized 2:1 to receive either the SAIL (Social and Lifestyle) intervention plus routine prenatal care or routine prenatal care (SOC) alone. The SAIL intervention consisted of six monthly in‐person group sessions led by a maternal‐fetal medicine physician, study nurse, social worker, and perinatal psychologist. Sessions addressed preeclampsia education, stress management, problem‐solving skills, and navigation of social resources. Participants completed surveys on preeclampsia knowledge, social needs, perceived stress, and resilience at baseline and in the third trimester. The primary outcome was intervention feasibility. Secondary outcomes included incidence of preeclampsia, live birth, preterm birth, postpartum hemorrhage, SGA, NICU admission, Stress assessed with perceived stress scale, and preeclampsia knowledge assessed with preeclampsia awareness survey.

**Results**: A total of 100 participants were enrolled and randomized. Baseline demographics and clinical characteristics were similar between groups (Table 1). Among those assigned to the SAIL intervention, 29 (48%) did not attend any group sessions, including 4 who delivered extremely preterm. Median session attendance was 17% (IQR 0–67%). Five participants were excluded from the analysis due to missing delivery data (*n* = 58 SAIL, *n* = 37 SOC). No significant differences were observed between groups in clinical outcomes (Table 2). However, in the third trimester, the SAIL group demonstrated significantly higher preeclampsia awareness (67% correct responses) compared to SOC (59% correct; *p* = 0.003).

**Conclusion**: Although in person group social risks‐focused lifestyle intervention was not feasible due to low attendance, it showed potential to improve preeclampsia awareness during pregnancy.

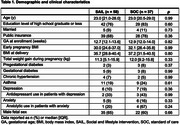

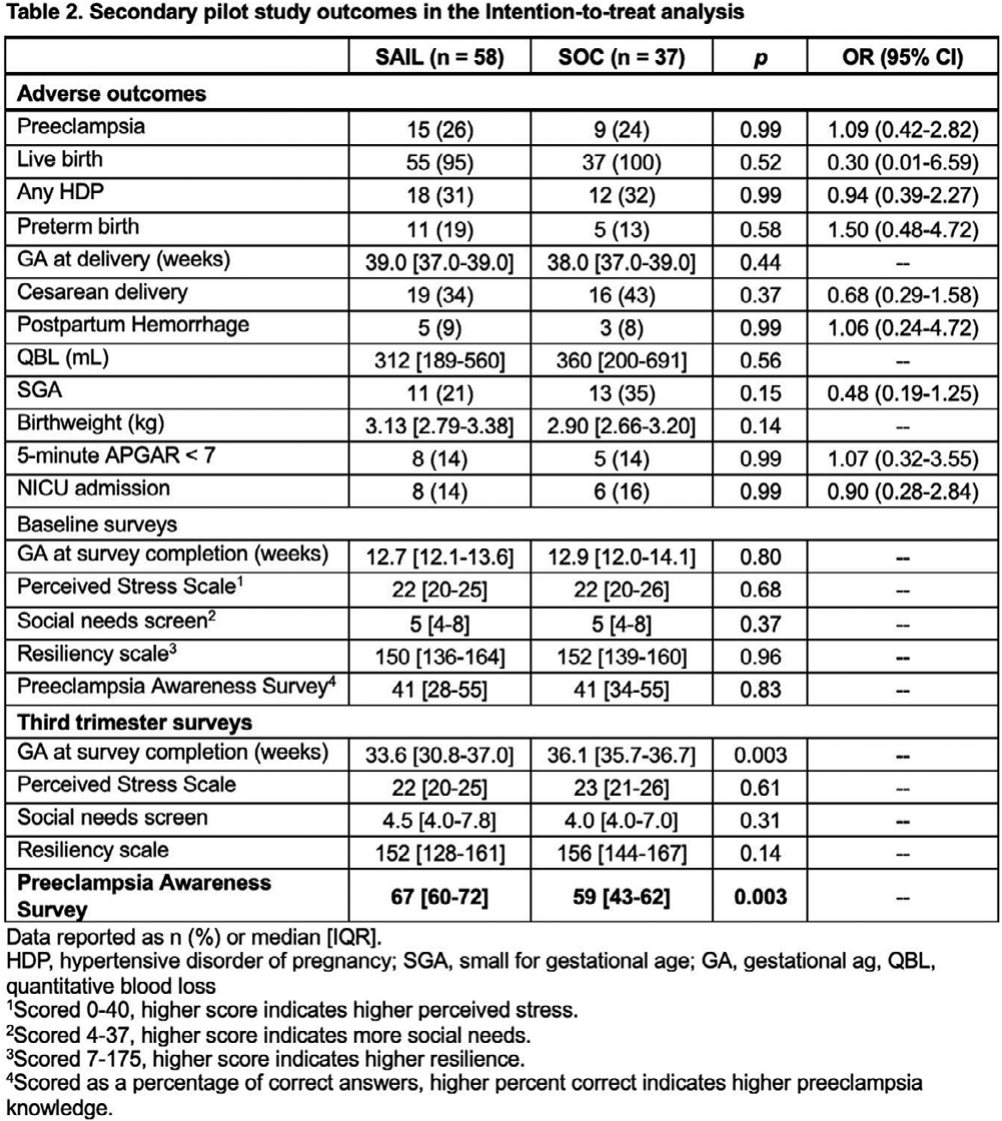

**10:30 AM – 12:00 PM**

## 190 | Adopting Technology for Glucose Optimization and Lifestyle in Pregnancy (AT GOAL): A Randomized Control Trial

Erin Bailey^1^; Janine S. Rhoades^2^; April Eddy^3^; Ken Croes^4^; Scott Hetzel^5^; Jacquelyn H. Adams^6^

^1^University of Wisconsin School of Medicine and Public Health, Department of Obstetrics and Gynecology, Madison, WI; ^2^University of Wisconsin Madison School of Medicine and Public Health, Madison, WI; ^3^Unity Point Health, Madison, WI; ^4^University of Wisconsin Madison, Madison, WI; ^5^University of Wisconsin‐Madison, Madison, WI; ^6^University of Wisconsin School of Medicine and Public Health, Madison, WI

**Objective**: This study aimed to assess the effect of use of continuous glucose monitors (CGMs) as well as patient satisfaction associated with their use among pregnant patients with type 2 diabetes (T2D).

**Study Design**: In this randomized control trial, pregnant patients with T2D were randomized <20 weeks’ gestation to standard finger stick blood glucose meters or CGMs from 8/2022–10/2024. Controls wore a blinded CGM on study entry and between 28–32 weeks for comparison. All participants answered quality of life surveys at set time points in the study. Qualitative, semi‐structured interviews were conducted postpartum. Interviews were transcribed and analyzed using a descriptive inductive qualitative methodology. Statistical analysis included t‐tests, Fisher's exact tests, and mixed effects longitudinal data analysis models.

**Results**: 16 patients enrolled in the study with similar baseline demographic data. Although not statistically significant, a clinically significant increase in time in range (TIR) occurred among the CGM group (8%) with a decrease in controls (‐9%). In interviews, emergent themes were identified and a conceptual framework was developed. There was significant dissatisfaction with checking finger sticks multiple times per day with a negative impact on patient experience. Concerns included forgetting to check, discomfort, amount of time and lack of control over blood sugars. CGM alleviated some of this burden; patients found it reduced the stress associated with having diabetes in pregnancy. Additionally, CGM use allowed for identification of dietary patterns that influenced blood sugar levels, increasing feelings of control over their disease. Neither cost nor device accuracy were of concern.

**Conclusion**: The burden of managing T2D in pregnancy is significant, and CGM appears to improve TIR, reduce burden on patients, and help patients feel in greater control. As CGM becomes more ubiquitous, these data support increased use of CGM in pregnancy to allow for greater individualized knowledge of blood sugar control with decreased stress of managing T2D. A larger randomized control trial is needed.

**10:30 AM – 12:00 PM**

## 191 | **Comparative Latency Following Mid‐Trimester Cerclage in Combination With Vaginal Progesterone**

Ethan Litman; Ai‐ris Y. Collier

Beth Israel Deaconess Medical Center, Boston, MA

**Objective**: There are no consensus guidelines for the use of combined therapy (cerclage and vaginal progesterone) for ultrasound‐indicated or physical exam‐indicated cerclage performed in mid‐gestation of pregnancy. Our objective is to define pregnancy latency following cerclage in combination with vaginal progesterone use before and/or after cerclage placement in comparison to cerclage alone.

**Study Design**: We utilized the Epic cosmos database to identify a cohort of pregnant individuals with cerclage procedure at 18–24 weeks gestational age from Jan 2015‐ Dec 2024 with singleton pregnancies who gave birth after 24 weeks. Patients with multifetal gestations, prescription for intramuscular progesterone, or missing birth records were excluded. Progesterone exposure was defined by presence of micronized progesterone prescription prior to and/or after cerclage procedure to generate 4 exposure groups: 1) No progesterone before nor after cerclage; 2) Progesterone before cerclage only; 3) Progesterone after cerclage only; and 4) Progesterone before and after cerclage. Data are presented as percent survival (undelivered) by group using Kaplan‐Meier curve. Pairwise comparisons of survival estimates at 28, 32, 34, and 37 weeks were reported with 95% confidence intervals (CI).

**Results**: 19,509 pregnancies were identified with cerclage placement at 18–24 weeks, including 10,666 in group 1; 4544 group 2; 2853 in group 3; and 1446 in group 4. 50% of pregnancies survived to at least 36 weeks gestation for all groups (Figure 1). Progesterone initiation after cerclage was associated with the lowest survival at all timepoints; whereas progesterone both pre‐ and post‐cerclage was associated with highest survival (Table 1). Progesterone added after cerclage conferred no additional survival benefit.

**Conclusion**: Data from this large cohort suggests that continuing progesterone after cerclage has highest benefit for pregnancy latency, but initiating progesterone after cerclage is associated with worse latency.

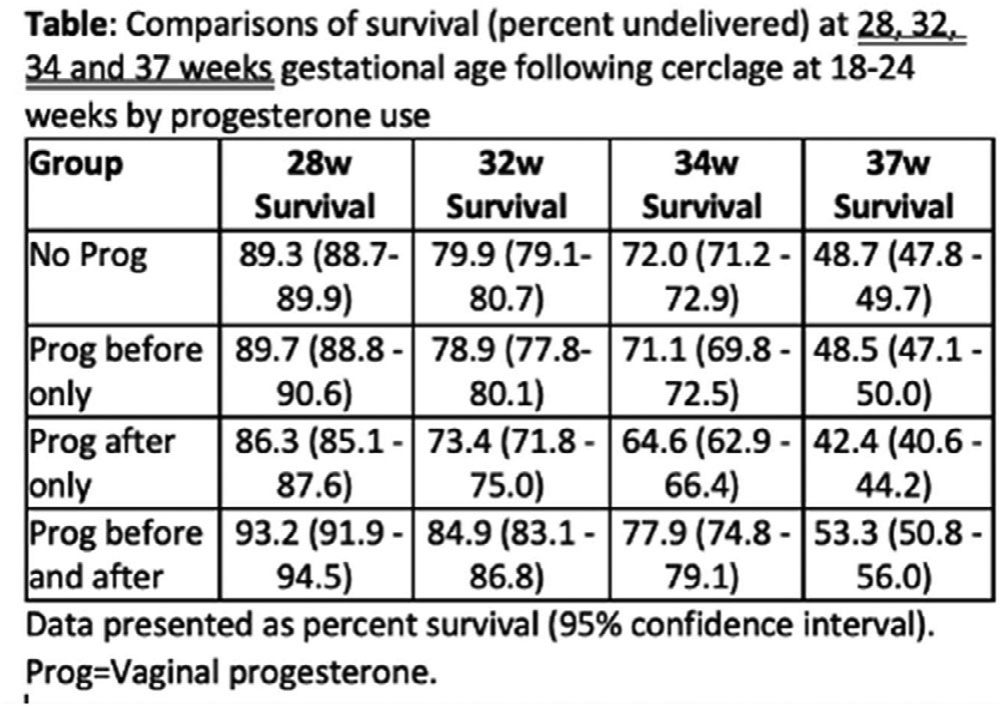

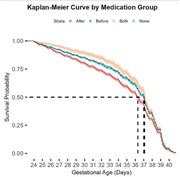

**10:30 AM – 12:00 PM**

## 192 | Maternal Comorbidity Burden and Maternal‐Newborn Dyadic Birth Outcomes: A Desirability of Outcome Ranking Analysis

Frank I. Jackson; Sarah H. Abelman; Oladunni Ogundipe; Fernando Suarez; Adriann Combs; Cara S. Wetcher; Dawnette Lewis; Burton Rochelson; Matthew J. Blitz

Northwell, New Hyde Park, NY

**Objective**: To evaluate the association between obstetric comorbidity index (OB‐CMI) and maternal‐newborn dyadic outcomes using the desirability of outcome ranking (DOOR) methodology, which orders outcomes by clinical desirability. OB‐CMI quantifies maternal comorbidity burden.

**Study Design**: This retrospective cross‐sectional study included all deliveries eligible for the Joint Commission's PC‐06 metric (term newborns without preexisting conditions) across seven hospitals in a New York health system (2019–2024). Only each patient's first delivery was included. Anemia was categorized as none (Hb ≥11.0 g/dL), mild (10.0–10.9), or moderate‐to‐severe (<10.0). Outcomes were classified into four DOOR categories: (1) uncomplicated vaginal delivery, (2) uncomplicated cesarean, (3) vaginal delivery with complications, and (4) cesarean with complications. Maternal and neonatal complications were defined by CDC severe maternal morbidity and PC‐06 criteria. Multinomial logistic regression assessed associations between anemia and DOOR, adjusting for obstetric comorbidity index (OB‐CMI) score, race and ethnicity, multiparity, insurance, and language

**Results**: A total of 105,156 deliveries were included. Birth outcomes by anemia severity are shown in the Figure; regression results are presented in the Table. Worsening anemia severity was strongly associated with less desirable outcomes. Compared to no anemia, mild anemia was associated with higher odds of vaginal delivery with complications (aOR 1.49; 95% CI, 1.34–1.65) and cesarean with complications (aOR 2.13; 95% CI, 1.93–2.34). Risks were greater for moderate‐to‐severe anemia: 3.49 times higher odds of vaginal delivery with complications (95% CI, 3.10–3.92) and 5.86 times higher odds of cesarean with complications (95% CI, 5.30–6.49). A dose‐response pattern was observed across all categories.

**Conclusion**: Maternal anemia is a strong, independent predictor of adverse maternal‐newborn outcomes. Increasing severity is linked to significantly worse DOOR outcomes, underscoring the need for early detection and intervention.

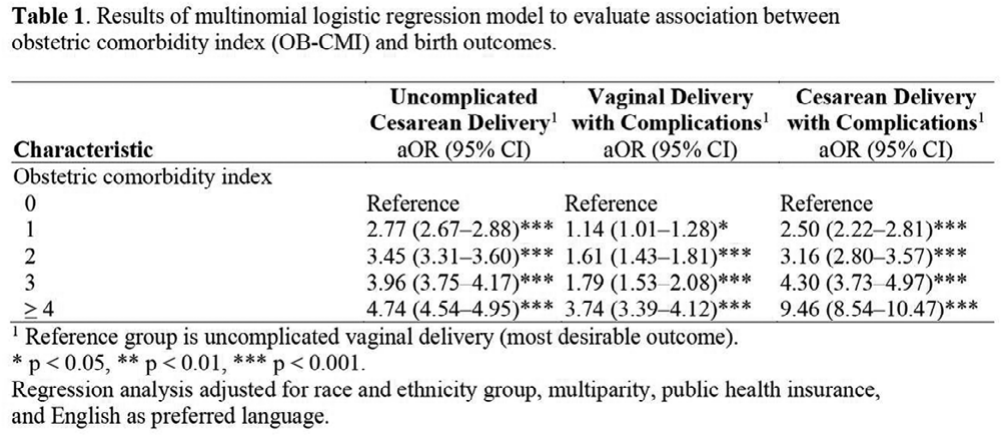

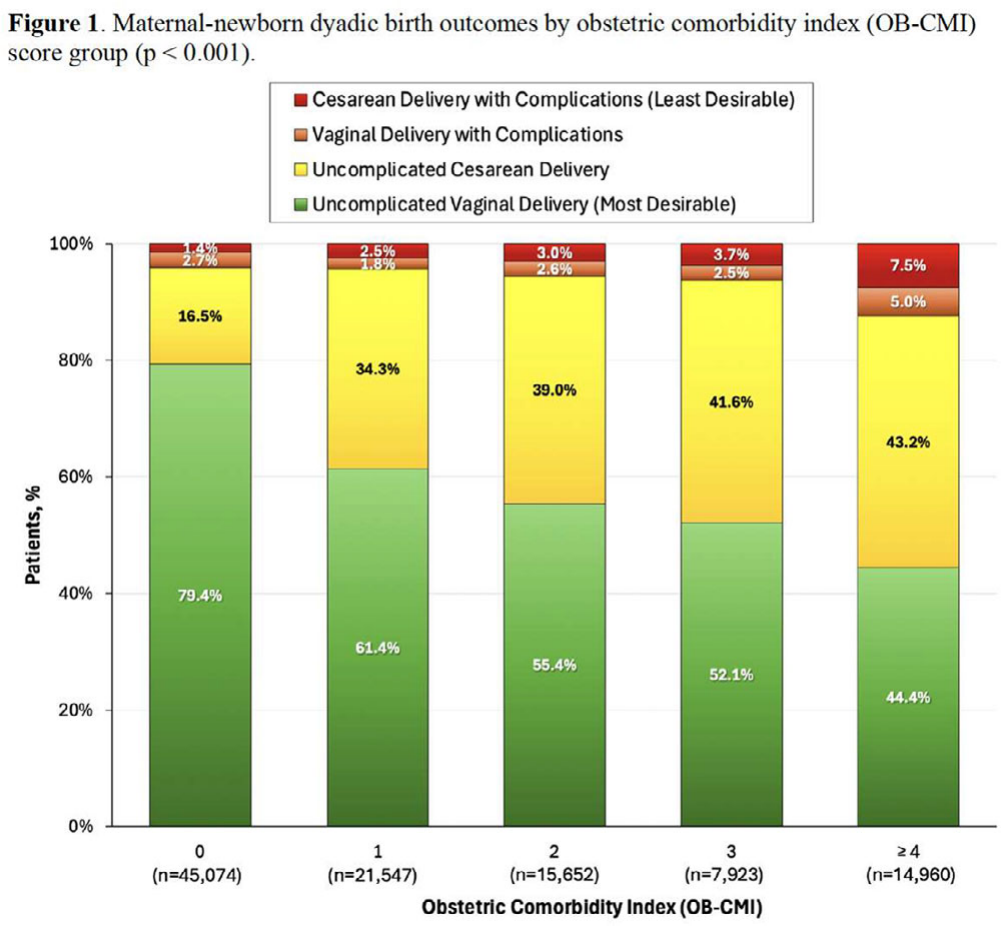

**10:30 AM – 12:00 PM**

## 193 | Prenatal Deep Phenotyping Using Ultrasound and MRI Improves the Diagnostic Yield of Exome Sequencing

Gabriela McMahon^1^; Samantha Doyle^2^

^1^National Maternity Hospital, National Maternity Hospital, Dublin; ^2^National Maternity Hospital, National Maternity Hosiptal, Dublin

**Objective**: To evaluate whether comprehensive prenatal deep phenotyping increases the diagnostic yield of exome sequencing in fetuses with anomalies where QF‐PCR and chromosomal microarray testing were non‐diagnostic.

**Study Design**: We conducted a prospective cohort study of cases referred to our department over a four‐year period for consideration of further genetic testing due to a suspected fetal anomaly. All cases had previously undergone QF‐PCR and chromosomal microarray with no molecular diagnosis identified. Selected cases underwent prenatal deep phenotyping. This was defined as detailed, multidisciplinary evaluation of the fetal phenotype using ultrasound, MRI or both to guide further genetic testing.

**Results**: A total of 109 patients met the inclusion criteria. The phenotypes seen were multisystem anomalies in 45% (*n* = 49), musculoskeletal in 22% (*n* = 24), neurological in 11.9% (*n* = 13), fetal hydrops in 6.4% (*n* = 7), cardiac in 4.6% (*n* = 5), cystic hygroma and gastrointestinal in 3.7% (*n* = 4), renal in 1.8% (*n* = 2) and face in 0.9% (*n* = 1).

An overall molecular diagnosis was achieved in 51.4% of cases (*n* = 56). Of the 109 cases, 34 underwent deep phenotyping by ultrasound, MRI or a combination of the two. Among the 34 cases who underwent deep phenotyping, 74% (*n* = 25) received a molecular diagnosis, compared to 41% (*n* = 31) of the 75 cases who did not undergo deep phenotyping. This difference was statistically significant (*p* = 0.002), indicating that deep phenotyping was associated with an increased diagnostic yield in this cohort.

**Conclusion**: Prenatal deep phenotyping using ultrasound and/or MRI is significantly associated with a higher rate of molecular diagnosis. This supports its value in the evaluation of fetal anomalies with inconclusive preliminary genetic testing.

**10:30 AM – 12:00 PM**

## 194 | **Assessing Applicant Attributes Valued by Maternal‐Fetal Medicine Fellowship Program Directors**

Rayyan Khan^1^; Gabrielle Hernandez^2^; Cameron Jackson^1^; Claudia Szlek^1^; Amalia Brawley^1^; Padmashree Woodham^1^

^1^Medical College of Georgia, Augusta, GA; ^2^Medical College of Georgia, Johns Creek, GA

**Objective**: Despite an increase in OB/GYN residents applying to Maternal‐Fetal Medicine (MFM) fellowship programs in recent years, the proportion of applicants matching at their top‐ranked program has declined significantly, from 34% in 2022 to 15% in 2023. This trend may reflect decreased transparency in the MFM fellowship application process, leaving applicants uncertain about how to optimize their chances of a successful match. The objective of this study was to identify which applicant characteristics are most valued by MFM fellowship program directors, in order to provide evidence‐based guidance for future applicants.

**Study Design**: A cross‐sectional electronic survey was distributed via institutional Qualtrics to all accredited MFM fellowship program directors. Responses were tabulated and ranked by median importance scores. Statistical analysis was conducted using the Friedman test in SPSS. This study received IRB Exempt Review under Category 2a.

**Results**: The Friedman test demonstrated a statistically significant difference among ranked factors (*p* < 0.001). Among the 52 responses received, the most highly ranked factor was an applicant's apparent commitment to the MFM specialty, followed by exposure to high‐risk obstetrics, letters of recommendation from MFM faculty, apparent fit with the program, and overall impression during interview. Factors rated as least important included elective rotations outside the home institution, international work, family planning training, and applying from within the same institution.

**Conclusion**: Apparent commitment to MFM, supported by relevant field exposure and strong specialty‐specific recommendations, appears to be the most influential factor in fellowship selection. These findings underscore the importance of intentional career development during residency and may help applicants better align their experiences and materials with fellowship program expectations.

**10:30 AM – 12:00 PM**

## 195 | Episiotomy and Obstetric Anal Sphincter Injury in Nulliparous Women With Prolonged Second Stage

Gal Bachar^1^; Shahar Rosenthal^2^; Nira Gridish^2^; Naphtali Justman^2^; Nizar Khatib^1^; Ron Beloosesky^3^; Yaniv Zipori^4^

^1^Rambam Health Care Campus, Haifa, HaZafon; ^2^Rambam Medical Health Center, Haifa, Hefa; ^3^Rambam Health Care Campus, Rambam Health Care Campus, Hefa; ^4^Rambam Health Care campus, Haifa, HaZafon

**Objective**: Allowing a prolonged second stage of labor in nulliparous women remains controversial. A significant concern is the increased risk of obstetric anal sphincter injuries (OASIS). This study assesses whether routine episiotomy can effectively reduce OASIS occurrence in nulliparous women undergoing vaginal delivery following a prolonged second stage.

**Study Design**: This retrospective study examined nulliparous women with singleton pregnancies who had a second stage of labor lasting≥3 hours and ultimately achieved spontaneous, non‐operative, vaginal delivery between 2014–2024. Participants were grouped by episiotomy status: with and without episiotomy. The primary outcome was the occurrence of OASIS, namely third‐ and fourth‐degree perineal lacerations. The main secondary outcomes comprised maternal and neonatal measures.

**Results**: Compared to the no‐episiotomy group, women in the episiotomy group were younger (27.79 ± 4.3 vs. 28.47 ± 4.5 years, *p* < 0.001), had slightly longer second stage (3.62 ± 0.4 vs. 3.53 ± 0.4 hours, *p* < 0.001), and higher birthweight (3366 ± 390 vs. 3284 ± 376 grams, *p* < 0.001). As shown in Figure 1, OASIS rates were comparable between the groups (1.9% vs. 2.2%, *p* = 0.82), consistent across all subtypes, and in a subanalysis of women with a second stage of ≥4 hours (2.9% vs. 2.5%, *p* = 0.588). Table 1 presents the secondary clinical outcomes: women who underwent episiotomy reported significantly greater postpartum pain during hospitalization (VAS score: 4.06 ± 3.2 vs. 3.61 ± 3.1, *p* = 0.003). Notably, despite this increased discomfort, they demonstrated a significantly higher rate of breastfeeding initiation (65.1% vs. 50.7%; *p* = = 0.01). Other maternal and neonatal outcomes did not differ significantly.

**Conclusion**: In nulliparous women with a prolonged second stage, routine episiotomy did not reduce the risk of OASIS. Although associated with increased postpartum pain, it was linked to higher breastfeeding initiation rates. These findings support existing guidelines advocating against routine episiotomy in this population. Further research is warranted to validate our findings and refine management strategies.

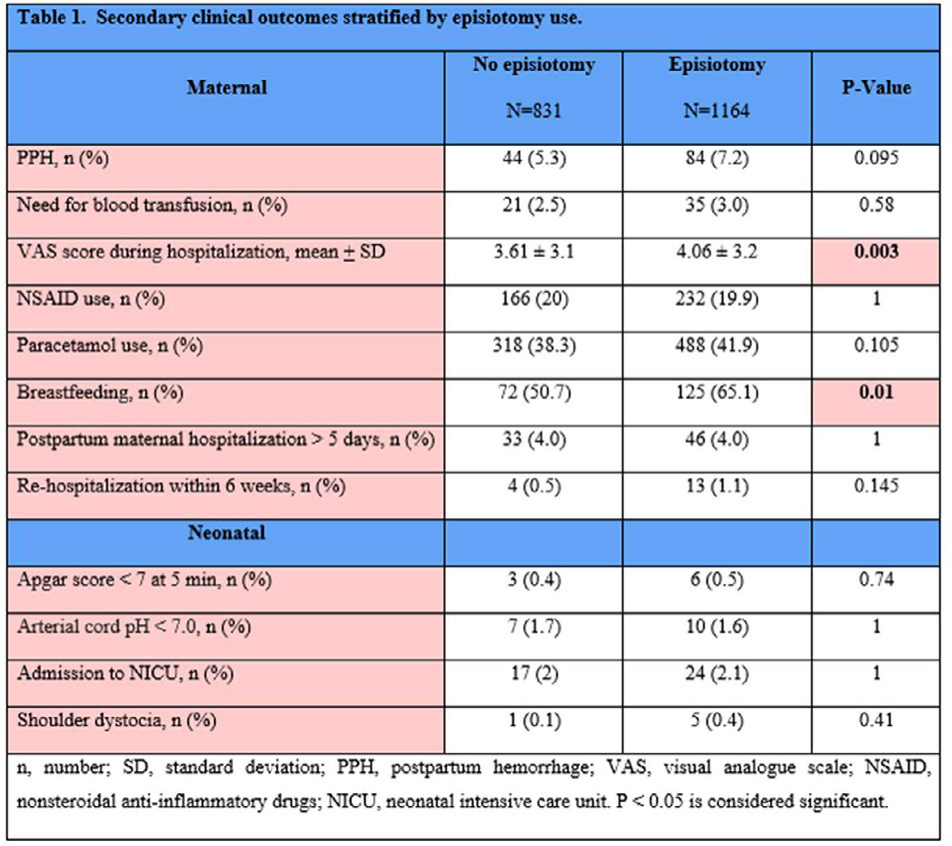

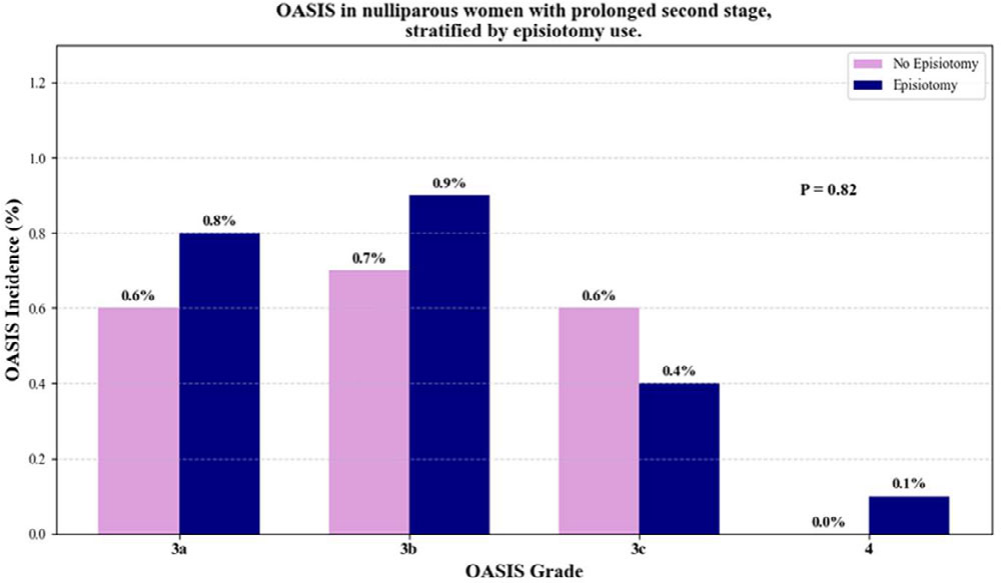

**10:30 AM – 12:00 PM**

## 196 | Association Between Breastfeeding and Risk of Postpartum Mental Health Disorders: A Nationwide Cohort Study

Geum Joon Cho^1^; Seo‐yeon KIM^2^; Sung Hun Na^3^; Ho Yeon Kim^4^; Jin‐Gon Bae^5^; Dong Wook Kwak^6^; Hyun‐Joo Seol^4^; Min‐Jeong Oh^4^; Young‐Han Kim^7^

^1^Department of Obstetrics and Gynecology, Korea University College of Medicine, Korea, Department of Obstetrics and Gynecology, Korea University College of Medicine, Korea, Seoul‐t'ukpyolsi; ^2^Department of Obstetrics and Gynecology, Kangbuk Samsung Hospital, Department of Obstetrics and Gynecology, Kangbuk Samsung Hospital, Korea, Seoul‐t'ukpyolsi; ^3^Department of Obstetrics and Gynecology, Kangwon National University, School of Medicine, Department of Obstetrics and Gynecology, Kangwon National University, School of Medicine, Kangwon‐do; ^4^Department of Obstetrics and Gynecology, Korea University College of Medicine, Department of Obstetrics and Gynecology, Korea University College of Medicine, Seoul‐t'ukpyolsi; ^5^Department of Obstetrics and Gynecology, Keimyung University, School of Medicine, Department of Obstetrics and Gynecology, Keimyung University, School of Medicine, Taegu‐jikhalsi; ^6^Department of Obstetrics and Gynecology, Ajou University School of Medicine, Suwon, Kyonggi‐do; ^7^Department of Obstetrics and Gynecology, Yonsei University College of Medicine, Department of Obstetrics and Gynecology, Yonsei University College of Medicine, Seoul‐t'ukpyolsi

**Objective**: This study aims to evaluate whether breastfeeding is associated with a reduced risk of postpartum mental health disorders within six months after delivery, using a nationwide cohort.

**Study Design**: We conducted a retrospective cohort study utilizing data from the Korean National Health Insurance Claims Database, encompassing all women who delivered between 2017 and 2020. Postpartum mental health disorders were defined as any new diagnosis of depression, anxiety, or stress‐related conditions within six months postpartum, identified using relevant ICD‐10 codes. Breastfeeding status was determined based on responses to the national infant health screening survey, which is routinely administered during early childhood health checkups. Participants were categorized into three groups according to infant feeding method: exclusive breastfeeding, mixed feeding, and formula feeding.

**Results**: A total of 190,992 postpartum women with available data on infant feeding methods were included in the analysis. Among them, 47,448 (24.8%) practiced exclusive breastfeeding, 91,443 (47.9%) used formula feeding, and 52,101 (27.3%) reported mixed feeding. On logistic regression analysis, the risk of postpartum mental health disorders was significantly lower in the exclusive breastfeeding groups (OR 0.640, 95% CI 0.576–0.711) and in the mixed feeding group (OR 0.799, 95% CI 0.727–0.878) compared to the formula feeding group after adjusting for potential confounding variables.

**Conclusion**: This nationwide cohort study demonstrated that breastfeeding, including mixed feeding, was associated with a significantly reduced risk of postpartum mental health disorders within six months after delivery. These findings suggest that promoting breastfeeding, even when not exclusively practiced, may have beneficial effects on maternal mental health. Future efforts should focus on developing and implementing strategies to support and encourage breastfeeding among postpartum women as a potential means to improve maternal psychological outcomes.

**10:30 AM – 12:00 PM**

## 197 | **Post‐Maturity and Offspring Neurological Outcomes: Uncovering Unexpected Benefits**

Gil Gutvirtz^1^; Gali Pariente^2^; Tamar Wainstock^3^; Eyal Sheiner^4^

^1^Department of Obstetrics and Gynecology, Soroka University Medical Center, Ben‐Gurion University, Metar, HaDarom; ^2^Department of Obstetrics and Gynecology, Soroka University Medical Center, Ben‐Gurion University, beer sheva, HaDarom; ^3^Department of Epidemiology, Biostatistics and Community Health Sciences, Faculty of Health Sciences, Ben‐Gurion University of the Negev, Beer Sheva, HaDarom; ^4^Soroka University Medical Center, Faculty of Health Sciences, Ben‐Gurion University of the Negev, beer sheva, HaDarom

**Objective**: Prematurity poses a significant risk factor for offspring adverse neurological outcomes as the fetal brain is still evolving, which requires great efforts to prevent preterm births. Guidelines are now indicating the full‐term period (39–41 weeks) as the optimal timing of delivery. However, post‐maturity (≥42 weeks) is rarely investigated. We sought to investigate whether birth beyond full‐term poses additional neurological risks for the offspring.

**Study Design**: We conducted a population‐based retrospective study including singleton births occurring between 1991–2021 at a tertiary medical center. The cohort was stratified by gestational age into full‐term (39–41 weeks), late‐term (41–42 weeks) and post‐term (≥42 weeks) deliveries. Offspring neurological health was investigated through data extracted from community and hospitalization records. Kaplan‐Meier survival curves were used to compare cumulative incidence of neurological morbidity and Cox regression models were used to estimate the long‐term risk of the offspring to develop neurological disorders, controlling for relevant confounders.

**Results**: A total of 232,476 births were included. The overall neurological morbidity was lower for children born beyond full‐term (Table), and the survival curves also demonstrated lower cumulative incidence of neurological morbidity among these children (Figure). The Cox regression models, controlling for maternal and gestational age, diabetes mellitus, hypertensive disorders and cesarean delivery, and comparing to full‐term birth, found a decreased risk of neurological morbidity in the offspring (Table).

**Conclusion**: In this large population‐based cohort, offspring born at late‐ and post‐term gestations demonstrated a lower risk of long‐term neurological morbidity compared with those born at full term. These findings may suggest enhanced neurological maturity with advancing gestational age. Nevertheless, the clinical decision to prolong pregnancy should be balanced against the established maternal and perinatal risks associated with post‐term gestation, underscoring the need for individualized timing of delivery.

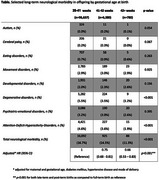

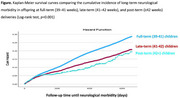

**10:30 AM – 12:00 PM**

## 198 | Long‐Term Neurological Morbidity of Offspring Born to Women With Uterine Rupture: A Population‐Based Cohort Study

Gil Gutvirtz^1^; Moran Shahar^2^; Gali Pariente^3^; Tamar Wainstock^4^; Eyal Sheiner^5^

^1^Department of Obstetrics and Gynecology, Soroka University Medical Center, Ben‐Gurion University, Metar, HaDarom; ^2^soroka medical center, soroka medical center, HaDarom; ^3^Department of Obstetrics and Gynecology, Soroka University Medical Center, Ben‐Gurion University, beer sheva, HaDarom; ^4^Department of Epidemiology, Biostatistics and Community Health Sciences, Faculty of Health Sciences, Ben‐Gurion University of the Negev, Beer Sheva, HaDarom; ^5^Soroka University Medical Center, Faculty of Health Sciences, Ben‐Gurion University of the Negev, beer sheva, HaDarom

**Objective**: Uterine rupture is a rare but serious obstetric complication, often resulting in adverse perinatal outcomes including fetal hypoxia, acidosis, and even death. While the short‐term neonatal risks associated with uterine rupture are well‐documented, data regarding long‐term consequences in surviving offspring are scarce. We aimed to assess whether being born to a mother who experienced uterine rupture is associated with increased long‐term neurological morbidity in the offspring.

**Study Design**: A population‐based retrospective study was conducted, including singleton deliveries at a tertiary hospital between 1991–2014. The incidence of pediatric neurological‐related hospitalizations (up to age 18) was compared between children born to mothers with and without uterine rupture. Neurological morbidity was obtained from hospitalization records. We excluded multiple gestations and children with congenital or chromosomal abnormalities. Cases of perinatal death were excluded from the long‐ term analysis. Kaplan‐Meier survival curves assessed cumulative incidence, and multivariable Cox regression models adjusted for potential confounders.

**Results**: Among 238,953 singleton deliveries, 139 (0.05%) were complicated by uterine rupture. Compared to those without rupture, mothers with rupture had higher rates of hypertensive disorders (9.4% vs. 5.1%, *p* = 0.021), placental abruption (5.8% vs. 0.5%, *p* < 0.001), and cesarean delivery (84.9% vs. 13.7%, *p* < 0.001). Perinatal mortality was significantly higher in the uterine rupture group (8.6% vs. 0.1%, *p* < 0.001). After excluding perinatal deaths, the final cohort included 127 offspring born after uterine rupture and 238,495 unexposed controls. Rates of long‐term neurological hospitalizations were similar between groups (3.1% vs. 1.6%, *p* = 0.314), with no significant difference in cumulative incidence (Figure). In adjusted analysis, uterine rupture was not associated with increased neurological morbidity (aHR 0.4; 95% CI 0.11–1.86; *p* = 0.279).

**Conclusion**: Uterine rupture during labor was not independently associated with increased long‐term neurological morbidity in the offspring.

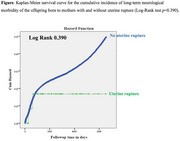

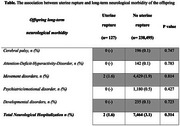

**10:30 AM – 12:00 PM**

## 199 | Fetal Heart Rate Measurement Using the Oxitone 1000M Wrist Device Compared to Standard Doppler Monitoring

Gil Shechter Maor^1^; Or Eliner^2^; Omer Weitzner^1^; Meital Golts^3^; Reuven Gladshtein^3^; Leon Eisen^3^; Tal Biron Shental^1^

^1^Meir Medical Center, Kfar Saba, HaMerkaz; ^2^Department of Obstetrics and Gynecology, Meir Medical Center, Ra'anana, HaMerkaz; ^3^Oxitone Medical LTD, Kfar Saba, HaMerkaz

**Objective**: The Oxitone 1000M is a wearable medical device designed for remote monitoring of heart rate using trans‐illumination photoplethysmography technology from peripheral sites, specifically the maternal wrist

This study aimed to evaluate the accuracy of fetal heart rate (FHR) measurement using the Oxitone 1000M device in comparison with the standard Doppler fetal heart rate monitor.

**Study Design**: A prospective observational study comparing two methods of FHR assessment simultaneously. The Oxitone 1000M device and the hospital's external Doppler monitor, the Philips Avalon FM30, as the reference.

Patients were recruited in the second half of a low‐risk, singleton pregnancy. Monitoring was conducted as part of routine prenatal follow‐up.

The Oxitone algorithm's performance was assessed using statistical methods, including correlation analysis and Bland‐Altman plots, to evaluate agreement with the reference device.

**Results**: We enrolled 20 participants who underwent NST. For each participant, FHR measurements obtained using the Oxitone algorithm and compared to those acquired from the reference device

The average gestational week was −40.6534.6 (  25. weeks) with an average BMI of 29.3 (18.1–39). Eight patients (40%) were primiparous, and 4 (20%) had contractions.

The average recording duration was 58 minutes (40‐ 100 minutes). In an average one‐hour recording for each patient, signal data were obtained continuously for 54.9% of the time.

The majority of the averaged measurements obtained using the Oxitone algorithm fell within a ±10% error margin relative to the reference device.

We evaluated the agreement and differences between the fetal heart rate measurements derived from the Oxitone device and the reference device for all participants. A total of 89.9% of the calculated data points were within a ±10% error range. Among the remaining values, 8.57% exceeded the upper limit, while 1.53% fell below the lower limit, with an average error of 6.8767 bpm (4.31%).

**Conclusion**: The Oxitone 1000M is a pioneering wearable device that enables non‐invasive, passive remote FHR monitoring from the maternal wrist.

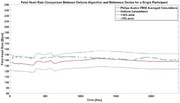

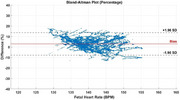

**10:30 AM – 12:00 PM**

## 200 | Statewide Maternal Morbidity and Mortality of Previa‐Associated Placenta Accreta Spectrum With Hysterectomy in California, 2007–2020

Graciela Caraballo^1^; Elizabeth B. Sherwin^2^; Stephanie A. Leonard^3^; Suzan L. Carmichael^3^; Christine H. Morton^4^; Katherine Bianco^5^; Daniela A. Carusi^6^; Deirdre J. Lyell^7^

^1^Stanford Healthcare, Palo Alto, CA; ^2^Stanford University, Stanford, CA; ^3^Stanford University, Stanford University, CA; ^4^Stanford University School of Medicine, Palo Alto, CA; ^5^Stanford University Healthcare, Palo Alto, CA; ^6^Department of Obstetrics and Gynecology, MassGeneral Brigham; Division of Maternal‐Fetal Medicine; Harvard Medical School, Boston, MA; ^7^Division of Maternal‐Fetal Medicine, Department of Obstetrics and Gynecology, Stanford School of Medicine, Stanford, CA

**Objective**: Placenta accreta spectrum (PAS) is a leading cause of obstetric hemorrhage and death from hemorrhage, yet few population‐level studies have reported its morbidity and mortality burden in an era of specialized care. Counseling based on outdated data may misinform patients with suspected PAS with previa, and compound distress. We aimed to quantify maternal death and severe maternal morbidity (SMM) in cases of previa‐associated PAS with hysterectomy in California.

**Study Design**: We conducted a retrospective cohort study using California linked vital record and maternal discharge data for births from 2007 to 2020. PAS diagnosis was identified using validated ICD‐9 methodology (Creanga et al., 2015) and ICD‐10 diagnosis codes for placenta accreta, increta, or percreta. Inclusion required a diagnosis of PAS, concurrent placenta previa, and cesarean hysterectomy during birth hospitalization, excluding uterine rupture. Outcomes were death and CDC‐defined SMM (excluding hysterectomy) during birth hospitalization. Modified Poisson regression modeling was used to estimate the risk of SMM and SMM indicators among PAS cases compared with all other deliveries during the same period.

**Results**: Among 2283 individuals with previa‐associated PAS with hysterectomy, mortality occurred in 0.53% (*n* = 12), 58.7% experienced SMM, and 19.7% experienced non‐transfusion SMM. Key contributors to SMM were blood transfusion (54.0%), disseminated intravascular coagulation (11.3%), acute respiratory distress syndrome (7.8%), ventilation (6.8%), and shock (5.8%). Nearly all SMM risks were substantially greater in PAS compared to all other deliveries (Table 1).

**Conclusion**: In this large population‐based cohort of previa‐associated PAS with hysterectomy, maternal death during delivery hospitalization occurred in 0.53%, lower than rates reported in older datasets, while rates of SMM and non‐transfusion SMM were substantial. These findings reflect recent management paradigms, inform patient counseling, and underscore the importance of risk‐appropriate regionalized care planning for patients with suspected previa‐associated PAS.

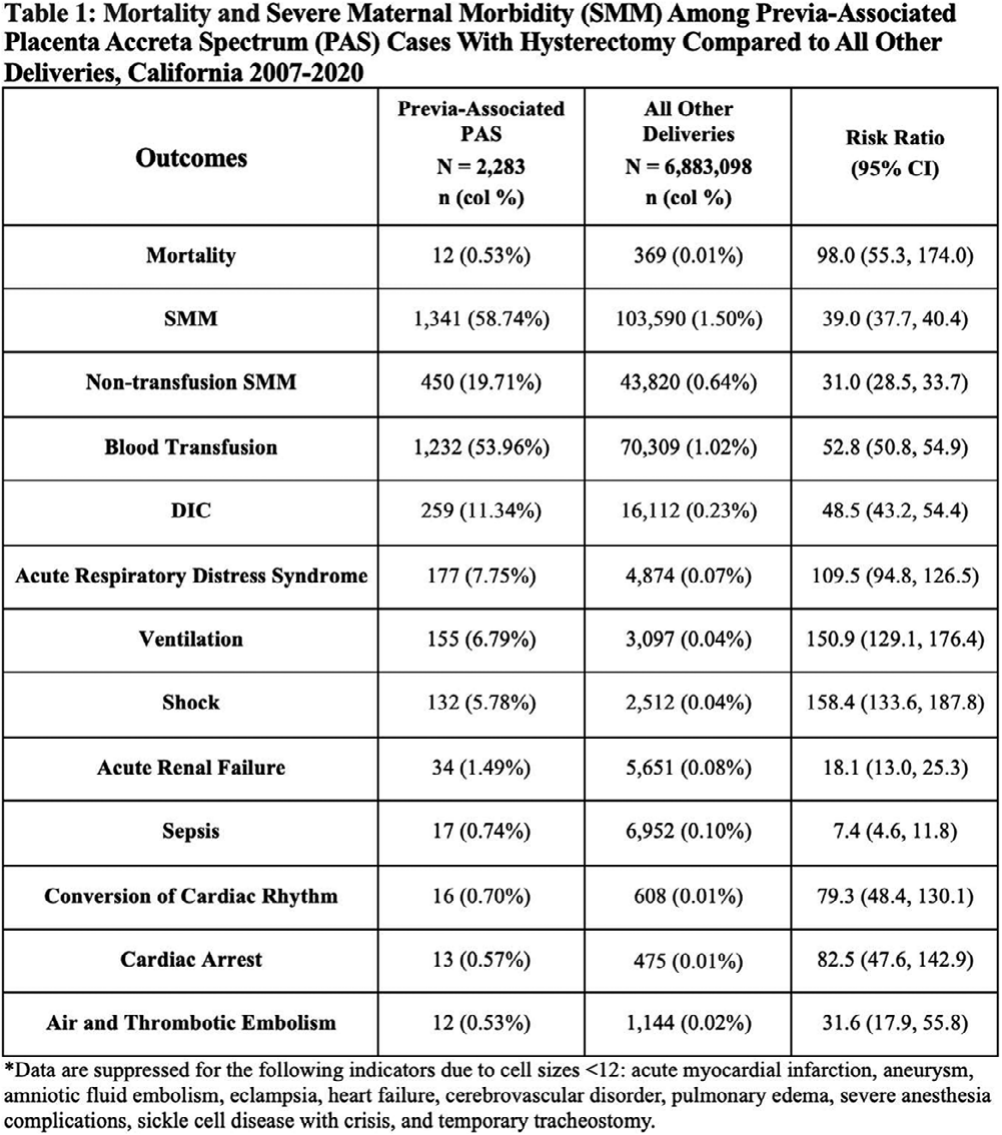

**10:30 AM – 12:00 PM**

## 201 | Maternal Asthma and Offspring Immunity: A Link to Long‐Term Infectious Morbidity?

Hagar Brami^1^; Gil Gutvirtz^2^; Tamar Wainstock^3^; Eyal Sheiner^4^

^1^Soroka, 0, HaDarom; ^2^Department of Obstetrics and Gynecology, Soroka University Medical Center, Ben‐Gurion University, Metar, HaDarom; ^3^Department of Epidemiology, Biostatistics and Community Health Sciences, Faculty of Health Sciences, Ben‐Gurion University of the Negev, Beer Sheva, HaDarom; ^4^Soroka University Medical Center, Faculty of Health Sciences, Ben‐Gurion University of the Negev, beer sheva, HaDarom

**Objective**: Asthma is a common chronic respiratory condition among reproductive age women, driven by immune system responses. In pregnancy, asthma is known to impact short‐term maternal and perinatal outcomes. However, the long‐term consequences of asthma on the offspring remains largely unexplored. We sought to evaluate whether maternal asthma during pregnancy is associated with increased long‐term infectious morbidity in the offspring, using data from a large population‐based birth cohort.

**Study Design**: A population‐based cohort analysis was conducted, including all singleton deliveries at a tertiary hospital that took place between 1991 and 2021. We compared the long‐term infectious morbidity of offspring born to mothers with and without asthma. A Kaplan‐Meier survival curve was used to assess offspring cumulative infectious morbidity. A Cox proportional hazards model was used to control for confounders.

**Results**: Out of the 232,476 births included in the study, 2875 (1.4%) were to mothers with asthma. Compared to offspring of non‐asthmatic women, those of asthmatic mothers had significantly higher rates of viral and bacterial infections as well as respiratory, gastrointestinal and ear‐nose‐throat infections, accumulating to a higher rate of total infectious morbidity (Table). The cumulative incidence of infectious morbidity over time was also significantly higher in children of asthmatic mothers (Figure). A Cox regression analysis, controlling for maternal and gestational age, diabetes mellitus, hypertensive disorders and cesarean delivery, found that maternal asthma was an independent risk factor for long‐term infectious morbidity in the offspring (aHR 1.16, 95% CI 1.11–1.21, *p* < 0.001).

**Conclusion**: Maternal asthma was independently associated with increased long‐term infectious morbidity in offspring, suggesting a possible alteration in immune development due to in‐utero exposure to a chronic inflammatory environment. These findings underscore the need to further explore the impact of maternal asthma on offspring health to elucidate the underlying mechanism.

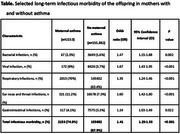

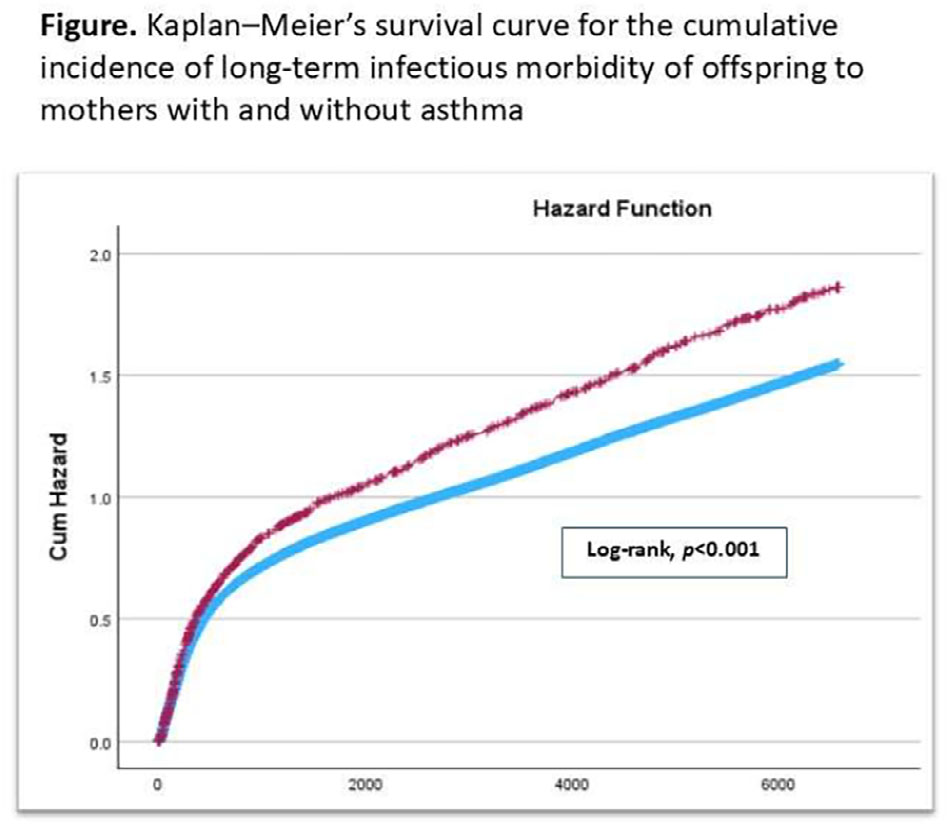

**10:30 AM – 12:00 PM**

## 202 | Predicting Periviable Delivery: Identifying Risk Factors Among Patients Admitted Between 21–24 Weeks

Hannah Caldwell; Alison Asirwatham; Edesiri Igbuya; Jessica Kloppenburg; Yiming Zhang; Jessica Moszkowicz; Katherine Leung; Katherine Johnson

UMass Chan Medical School, Worcester, MA

**Objective**: To identify factors associated with delivery <25 weeks in patients with periviable admissions.

**Study Design**: We conducted a retrospective cohort study of patients admitted between 21w0d–24 w6d at a single tertiary care center from 2018–2024. Patients were stratified by gestational age at delivery (<25 vs ≥25 weeks). Maternal demographics, admission characteristics, counseling, interventions, and outcomes were compared using appropriate statistical tests. A Kaplan‐Meier analysis was used to estimate gestational age at delivery.

**Results**: Among 255 patients included with periviable admissions, 72 (28.2%) delivered <25 weeks. Significant predictors of delivery <25 vs ≥25 weeks included higher BMI (32.1 vs 29.7, *p* = 0.019), Black race (32.6% vs 10.0%, *p* = 0.003), multiple gestations (16.7% vs 5.5%, *p* = 0.011), and admission for spontaneous preterm labor (45.8% vs 11.5%, *p* < 0.001) or PPROM (43.1% vs 13.7%, *p* < 0.001). There were no significant differences in maternal age or ethnicity. Kaplan‐Meier survival analysis showed 50% of the patients who received periviability counseling delivered before 27 weeks versus 37 weeks for the group without counseling.

Antenatal interventions such as corticosteroids, magnesium sulfate, and tocolysis were similarly planned for patients who ultimately delivered <25 vs ≥ 25 weeks, with no significant differences.

**Conclusion**: These findings may inform prospective identification strategies to guide early counseling and targeted periviable management. The differences in delivery timing between the counseled (50% by 27 weeks) and non‐counseled (50% by 37 weeks) groups suggest clinicians are already identifying high‐risk cases, though future predictive tools could enhance risk stratification at the time of admission.

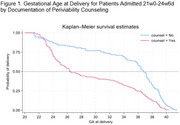

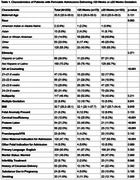

**10:30 AM – 12:00 PM**

## 203 | Induction of Labor After Cesarean: Factors Associated With Repeat Cesarean Delivery

Hannah S. Foster^1^; Holly Cummings^2^; Markolline Forpka^3^; Lisa D. Levine^4^

^1^Hospital of the University of Pennsylvania, Philadelphia, PA; ^2^Perelman School of Medicine at the University of Pennsylvania, Philadelphia, PA; ^3^Penn Medicine Women's Health Clinical Research Center, Philadelphia, PA; ^4^Perelman School of Medicine, University of Pennsylvania, Philadelphia, PA

**Objective**: Prior vaginal delivery (VD), more favorable Bishop score, and non‐recurring indication for primary Cesarean Delivery (pCD), e.g. fetal malpresentation are associated with successful Vaginal Birth After Cesarean (VBAC). However this research is in patients in spontaneous labor. Less is known about factors influencing successful Trial of Labor after Cesarean (TOLAC) among patients undergoing an induction of labor (IOL). Therefore, we aimed to evaluate risk factors for failed IOL after Cesarean (IOLAC).

**Study Design**: This retrospective cohort study included patients at 2 hospitals who underwent IOLAC in 2024. Term patients with a history of 1 pCD were included. The primary outcome was failed IOLAC (defined as repeat CD, rCD). Association between mode of delivery and patient characteristics were calculated using Fisher's Exact, Student's t‐, Chi‐Square, and Wilcoxon Rank Sum tests where appropriate. Multivariable stepwise regression analyses with statistically significant associations with mode of delivery were utilized to predict rCD.

**Results**: Out of the 14,205 patients who delivered in 2024, 485 were TOLACs, 232 (50.7%) underwent IOL, and 201 were term. 133 patients (66.2%) had a VBAC, and 68 (33.8%) had an rCD. Table 1 compares characteristics between those who had a VBAC vs. rCD. In univariate analysis, patients with a prior VD before their CD, prior VBAC, and higher Bishop were less likely to have an rCD. Indication for pCD and IOL method were also found to be significantly associated with mode of delivery. In multivariable regression modeling, only higher Bishop, history of prior VBAC, and indication for pCD remained in the final regression model. Among the indications for pCD, a prior history of failed induction had the highest risk of rCD (RR: 4.68, CI: 1.54–14.29) when controlling for prior VBAC and Bishop.

**Conclusion**: In this diverse cohort of 201 IOLAC patients, more than 65% were successful. Having a lower Bishop, no prior VBAC, and having a pCS for failed induction are factors predictive of risk of rCD. Future research regarding specific methods of IOLAC and associated outcomes is warranted.

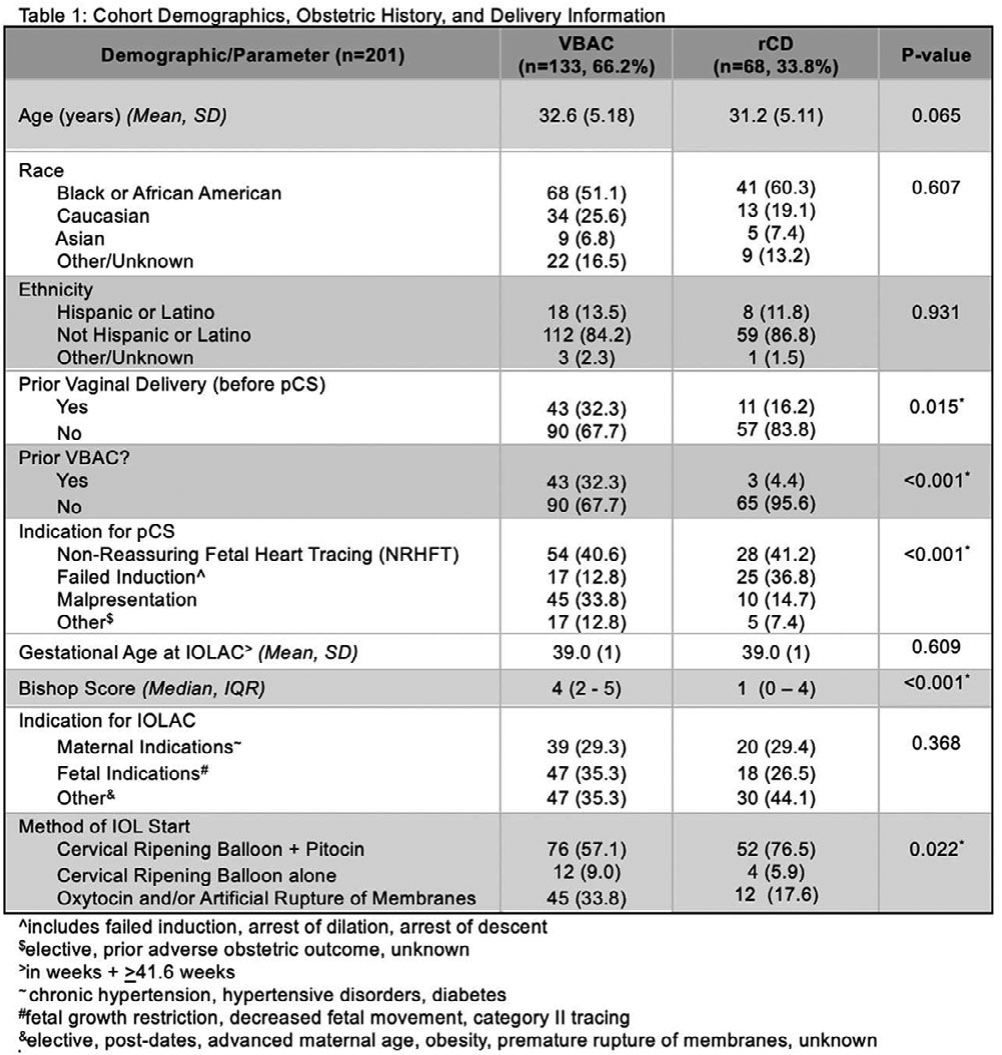

**10:30 AM – 12:00 PM**

## 204 | Maternal Outcomes Differ by Preeclampsia With Severe Features Subtype

Hannaneh Mirmozaffari^1^; Madison Calvert^1^; Maya Patel^1^; Jaye Boissiere^2^; Sally Kuehn^2^; Brooke Schroeder^2^; Matt Fuller^2^; Marie‐Louise Meng^2^; Johanna Quist‐Nelson^1^; Kim Boggess^3^

^1^University of North Carolina, Chapel Hill, Chapel Hill, NC; ^2^Duke University School of Medicine, Durham, NC; ^3^University of North Carolina at Chapel Hill, Chapel Hill, NC

**Objective**: Preeclampsia (PEC) is associated with major maternal morbidity. Up to 43% with PEC have >1 qualifying criteria for severe features. Although ACOG defines criteria for PEC with severe features (PEC‐SF), the frequency of each severe criterion and the association of different criteria on adverse maternal outcomes is unknown. We aimed to estimate the association between defining criteria of PEC‐SF with immediate severe maternal morbidity (SMM).

**Study Design**: Retrospective cohort study of patients with PEC‐SF who delivered at two centers from 12/2015 to 12/2017. Patients were identified via ICD‐10 codes and chart abstraction was done. PEC‐SF was defined per ACOG guidelines. Our primary outcome was CDC‐defined SMM (MI, aneurysm, renal failure, ARDS, AFE, VFib, cardioversion, DIC, transfusion, eclampsia, CHF, CVA, anesthetic complications, sepsis, shock, sickle crisis, pulmonary edema, air/thrombotic embolism, hysterectomy, tracheostomy, and/or ventilation) during the delivery hospitalization. Our secondary outcome was SMM at 1 year postpartum (PP). Using multiple regression, adjusting for maternal BMI >30 kg/m^2^, preexisting CHTN, diabetes, and/or renal insufficiency, we report aOR (95% CI) to estimate the association between each criterion and SMM at the 2 time points.

**Results**: 774 patients were analyzed. Severe HTN (85.4%) and headache (34.9%) were the most common criteria (Figure 1). SMM occurred at delivery hospitalization in 95 (12.3%) and within 1 year PP in 30 (3.9%). Blood pressure and headache were least likely to be present in patients with SMM at delivery (Figure 2). Thrombocytopenia [aOR 1.3 (1.2–1.4)], renal dysfunction [aOR 1.4 (1.3–1.6)], and pulmonary edema [aOR 1.9 (1.6–2.1)] were associated with SMM at delivery, and liver dysfunction [aOR 0.9 (0.9–1.0)] was protective. There were no significant associations between criteria and SMM within 1 year PP.

**Conclusion**: More than 10% of patients with PEC‐SF have an SMM at delivery hospitalization and those with thrombocytopenia, kidney dysfunction, or pulmonary edema as severe criteria should be delivered at hospitals equipped to manage SMM.

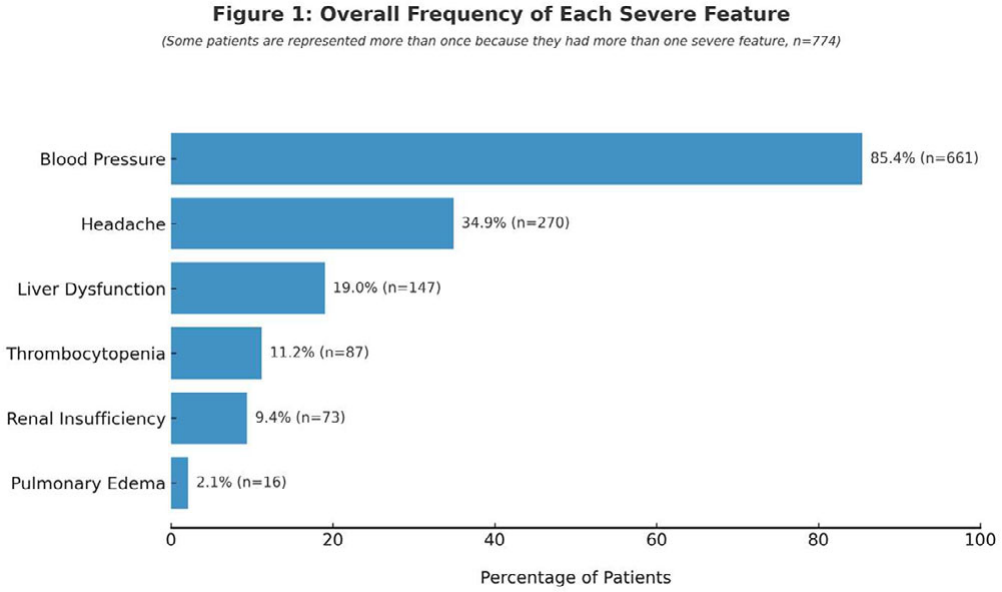

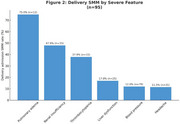

**10:30 AM – 12:00 PM**

## 205 | Postpartum Weight Retention Trajectories in Women With Prior Gestational Diabetes

Harumi Kanzawa^1^; Ichiro Yasuhi^2^; Hiroshi Yamashita^2^

^1^Nagasaki University Graduate School of Biomedical Sciences, Omura, Nagasaki; ^2^NHO Nagasaki Medical Center, Omura, Nagasaki

**Objective**: Postpartum weight retention (PPWR) is a known risk factor for type 2 diabetes among women with prior gestational diabetes (GDM). Although breastfeeding may support postpartum metabolic recovery, its influence on weight trajectories in this population remains unclear. This study evaluated 5‐year PPWR trajectories and the impact of early postpartum breastfeeding intensity.

**Study Design**: This longitudinal study included 521 women with prior GDM who delivered singleton pregnancies at a Japanese tertiary perinatal center. PPWR was calculated as postpartum weight minus prepregnancy weight and assessed at early postpartum and at 1, 2, 3, and 5 years. Women were categorized by early PPWR level (high: > = 75th percentile vs. low: <75th) and by breastfeeding intensity (high‐intensity breastfeeding [HIB] vs. non‐HIB). Multivariable linear models adjusted for prepregnancy BMI, gestational weight gain (GWG), maternal age, time of assessment, and delivery mode were used to compare mean PPWR trajectories.

**Results**: Women with high early PPWR (> = 75th percentile) had persistently higher PPWR at all time points through 5 years (*p* < 0.001). The gap in weight retention between high and low early PPWR groups widened over time. In contrast, breastfeeding intensity showed no significant association with PPWR at any point, including at 5 years (Figure). Among covariates, early PPWR and GWG were the most robust predictors of long‐term weight retention.

**Conclusion**: Early postpartum weight retention strongly predicts long‐term maternal weight retention in women with prior GDM. Despite its other benefits, breastfeeding intensity did not significantly influence PPWR trajectories. Interventions targeting women with high early PPWR, regardless of breastfeeding status, may be key to preventing prolonged weight retention and its cardiometabolic consequences.

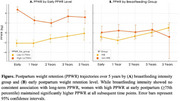

**10:30 AM – 12:00 PM**

## 206 | Pre‐Pregnancy Body Mass Index and its Association With Severe Maternal Morbidity

Havilah Reimche‐Vu^1^; Bo Park^2^; Ashra Tugung^3^; Nida Ali^3^; Neville Tritch^1^; Kriti N. Vedhanayagam^4^; Kevin H. Hu^4^; Sergio A. Karageuzian^5^; Ilish Gedestad^5^; Ruofan H. Yao^4^

^1^Loma Linda University School of Medicine, Loma Linda, CA; ^2^California State University, Fullerton, Fullerton, CA; ^3^Loma Linda University Children's Health | Perinatal Institute, Loma Linda, CA; ^4^Loma Linda University, Loma Linda, CA; ^5^Loma Linda University Health, Loma Linda, CA

**Objective**: To evaluate the association between pre‐pregnancy body mass index and the risk of severe maternal morbidity (SMM) as defined by the Centers for Disease Control and Prevention.

**Study Design**: This is a population‐based, retrospective cohort study using the California Office of Statewide Health Planning and Development Linked Birth File with hospital discharge International Classification of Diseases, 9th Revision, Clinical Modification (ICD‐9‐CM) diagnoses between 2007 and 2012. Chi‐square tests were used to determine the association between pre‐pregnancy BMI and SMM. Cox proportional hazard analysis was used to determine the risk of SMM associated with each BMI category, adjusting for confounding variables such as delivery method, number of cesarian sections, and maternal age. A cumulative hazard graph was generated to show the timing of SMM events during pregnancy with each associated BMI category.

**Results**: This analysis included 3,076,580 births. Among individuals with a normal pre‐pregnancy BMI (*n* = 1,404,674), 1.41% experienced at least one SMM outcome. Underweight and morbidly obese groups showed the highest risk of SMM, with the underweight group (*n* = 114,529) showing a SMM rate of 1.56% and a hazard ratio (HR) of 1.28 [95% CI: 1.22–1.34] and the morbidly obese group (*n* = 328,652) having an SMM rate of 1.73% and a HR of 1.14 [95% CI: 1.10–1.17]. Conversely, overweight and obese groups are associated with decreased SMM risk, with overweight individuals (*n* = 729,967) having a SMM rate of 1.46% and a HR of 0.96 [CI: 0.94‐0.98] and obese individuals (*n* = 498,758) having a SMM rate of 1.47% and a HR of 0.90 [95% CI: 0.87‐0.92].

**Conclusion**: Underweight and morbidly obese groups are associated with an increased risk of SMM, whereas overweight and obese groups are associated with a lower risk of SMM.

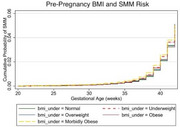

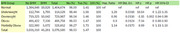

**10:30 AM – 12:00 PM**

## 207 | Microplastic‐Exposed Endometrial Stromal Cells Exhibit Mitochondrial Dysfunction During Decidualization, Potentially Impairing Pregnancy Outcomes

Hayan Kwon^1^; Yong‐Sun Maeng^1^; Yeji Lee^1^; Suhra Kim^1^; Ju‐Hee Yoon^1^; Yun Ji Jung^1^; JoonHo Lee^1^; Young‐Han Kim^2^; Ja‐Young Kwon^1^

^1^Department of Obstetrics and Gynecology, Yonsei University College of Medicine, Seoul, Seoul‐t'ukpyolsi; ^2^Department of Obstetrics and Gynecology, Yonsei University College of Medicine, Department of Obstetrics and Gynecology, Yonsei University College of Medicine, Seoul‐t'ukpyolsi

**Objective**: The widespread use of plastics has led to increasing exposure to microplastics (MP), raising concerns about their potential impact on reproductive health. Decidualization, the differentiation of human endometrial stromal cells (hESCs), is essential for establishing a receptive endometrium and maintaining a healthy maternal‐fetal interface. Disruption of this process by MP exposure could impair implantation and lead to adverse pregnancy outcomes. Mitochondrial dysfunction may be a key mediator of this effect, but the underlying mechanisms remain poorly understood. This study investigates the impact of MP exposure on decidualization of hESCs, focusing on mitochondrial dysfunction as a possible mechanism.

**Study Design**: MP (polyvinyl chloride  :PVC), in micro (<25 µm) were used to investigate the toxic effects and potential mechanisms of MP on decidualization of hESCs. Morphological and functional changes were assessed in vitro using immunofluorescence staining, electron microscopy, quantitative real‐time polymerase chain reaction, Western blotting, and microarray analysis.

**Results**: The results showed that exposure to PVC had significant biological effects, inhibiting the decidualization of hESCs in vitro, both morphologically and functionally. Electron microscopy revealed mitochondrial abnormality and immunofluorescence staining showed functional impairement of mitochondria. In addition, Microarray analysis exhibited that PVC‐exposed decidualized stromal cells (DSCs) showed a reduction in the expression of genes related with Ca2+ fluxes between ER and mitochondria in ESC.

**Conclusion**: This study demonstrated that PVC inhibits the decidualization of hESCs and induces the mitochondrial dysfunction in DSCs. It is suggested that mitochondrial dysfunction in decidualization of hESCs caused by PVC may be one of the mechanisms potentially compromising the maternal‐fetal interface and leading to adverse pregnancy outcomes (Grant Number  : NRF‐2022R1I1A1A01064011 and RS‐2024‐00342487)

**10:30 AM – 12:00 PM**

## 208 | **Pilot Implementation of Nurse‐Physician Phone Support Intervention for Maternity Health Workers: Global Health Translational Research**

Henna Budhwani^1^; Comfort Enah^2^; Lily B. Cooper^1^; Gregory Halle‐Ekane^3^; Eric Wallace^4^; Victoria R. Jauk^5^; Jeff M. Szychowski^6^; Waldemar A. Carlo^7^; Mary Glory Ngong^8^; Rahel M. Kyeng^8^; Pius M. Tih^8^; Alan TN Tita^5^

^1^Florida State University College of Nursing, Tallahassee, FL; ^2^University of Massachusetts Lowell School of Nursing, Lowell, MA; ^3^University of Buea Department of Obstetrics and Gynecology, Buea, Sud‐Ouest; ^4^University of Alabama at Birmingham Heersink School of Medicine, Birmingham, AL; ^5^Center for Research in Women's Health, University of Alabama at Birmingham, Birmingham, AL; ^6^Department of Biostatistics, University of Alabama at Birmingham, Birmingham, AL; ^7^Department of Pediatrics, University of Alabama at Birmingham, Birmingham, AL; ^8^Cameroon Baptist Convention Health Services, Bamenda, Nord‐Ouest

**Objective**: Pilot test a maternal health systems provider‐to‐provider phone‐intervention adapted for local contexts and evaluate its acceptability in the Ndop health district of Cameroon.

**Study Design**: Translational adaptation of 24/7 provider support system Medical Information Service via Telephone (MIST) into a mobile system (mMIST). Prior to full‐scale stepped wedge cluster randomized implementation, we conducted a pilot assessment in one health district, (1) capturing call volume over a 12‐month period, and (2) evaluating maternal outcomes pre‐ and during the intervention period by analyzing administrative data from the regional health authority. From the administrative data, a preliminary effectiveness outcomes included maternal deaths.

**Results**: From 03/2022‐02/2023, 106 providers were trained to provide mMIST consults (*n* = 30 nurses, midwives, OB/GYNs, and pediatricians) and utilize mMIST (*n* = 76 peripheral providers, including nurses and lay practitioners). Average monthly volume was 14 calls into the mMIST system; ∼30% required expert consultation, indicating adequate demand and acceptability. Most frequent reasons for maternal calls were abnormal labor (23%), hemorrhage (12%), infections (7%), hypertensive disorders (7%), and fetal malpresentation (6%). There were 8 maternal deaths in the pre‐intervention period compared to 1 during the intervention period. Select secondary outcomes increased during the pilot. For example, prolonged/obstructed labor increased to 3.8% vs. 2.5% and uterine rupture was 0.6% vs. 0.1%.

**Conclusion**: During the pilot, civil unrest increased in the region, increasing the need for maternal health support for remote providers and communities. Even so, the mMIST intervention was feasible and acceptable demonstrating potential for translating a multilevel, provider‐focused maternal health intervention for high‐need resource‐limited settings. Ongoing assessment will guide scalable deployment strategies to strengthen maternal care systems in Cameroon.

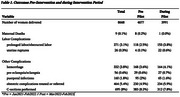

**10:30 AM – 12:00 PM**

## 209 | Preconception Prediction of Placenta Accreta Spectrum Using Machine Learning and EMR Data

Henri M. Rosenberg^1^; Tess EK Cersonsky^2^; Isabelle Band^3^; Eugenia Alleva^4^; Angela T. Bianco^4^

^1^Icahn School of Medicine at Mount Sinai, Icahn School of Medicine at Mount Sinai, NY; ^2^Icahn School of Medicine at Mount Sinai, NY; ^3^Mount Sinai Hospital, New York, NY; ^4^Icahn School of Medicine at Mount Sinai, New York, NY

**Objective**: To develop a machine learning (ML) model using only preconception electronic medical record (EMR) data to identify patients at risk for Placenta Accreta Spectrum (PAS) and evaluate its predictive performance.

**Study Design**: We conducted a retrospective case‐control study using EMR data from 118,890 deliveries (2013–2023) within a single academic health system. PAS was confirmed in 274 cases (0.23%) via manual review of delivery and pathology records. Pre‐pregnancy data included demographics, obstetric and surgical history, vitals, labs, billing codes, and provider notes. Natural language processing (NLP) extracted structured features from free‐text notes. ML models tested included logistic regression, decision trees, random forest, XGBoost, and multilayer perceptron. Model performance was evaluated using AUC, sensitivity, specificity, and SHAP (Shapley Additive Explanations) for interpretability.

**Results**: XGBoost achieved the highest area under the receiver operating curve (AUROC) (0.86) and outperformed logistic regression, AUROC 0.76 [Figure 1]. Random forest demonstrated the highest sensitivity (91%), while logistic regression had the highest specificity (91%) [Figure 1]. SHAP analysis revealed that traditional risk factors—including advanced maternal age, prior cesarean delivery, dilation and curettage (D&C), gynecologic surgery, and history of obstetric complications were among the top predictors. Pre‐pregnancy anemia emerged as a previously unrecognized risk factor [Figure 2].

**Conclusion**: Preconception ML models using EMR data can identify patients at elevated risk for PAS with high sensitivity and specificity. This early screening approach may facilitate risk counseling, referral, and personalized care planning before conception—especially in patients without access to expert imaging. The identification of anemia as a novel predictor highlights the potential role of modifiable risk factors and warrants further investigation.

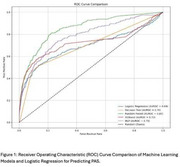

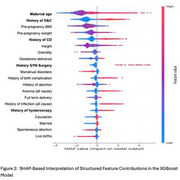

**10:30 AM – 12:00 PM**

## 210 | Mode of Delivery and Survival in Extremely Preterm Twins by Gestational Age and Neonatal Intervention

Hiba J. Mustafa^1^; Jana Karam^2^; Kevin Moss^3^; Cynthia Gyamfi‐Bannerman^4^; George R. Saade^5^; Alireza A. Shamshirsaz^6^

^1^Division of Maternal‐Fetal Medicine, Indiana University School of Medicine, Indianapolis, IN; ^2^Lebanese American University School of Medicine, Beirut, Beyrouth; ^3^Indiana University, Indianapolis, IN; ^4^Division of Maternal Fetal Medicine, Department of Obstetrics, Gynecology and Reproductive Sciences, University of California San Diego, San Diego, CA; ^5^Eastern Virginia Medical School at Old Dominion University, Norfolk, VA; ^6^Boston Children's Hospital, Harvard Medical School, Boston, MA

**Objective**: To investigate the association between mode of delivery and neonatal outcomes in twin pregnancies delivered at 22–27 + 6 weeks’ gestation, focusing on survival by gestational age and neonatal active intervention

**Study Design**: A cross‐sectional, population‐based cohort study was conducted using U.S. natality matched multiple birth and fetal death data from 2016–2020. Live‐born twin pregnancies between 22 and 27 + 6 weeks were included. The primary exposure was mode of delivery (cesarean or vaginal); the primary outcome was in‐hospital neonatal death. Secondary outcomes included need for ventilation, surfactant or antibiotic use, seizures, and NICU admission. Analyses were stratified by gestational age and conducted for the entire cohort and the subgroup receiving active postnatal interventions. Adjusted odds ratios (aOR) were estimated using multivariable logistic regression.

**Results**: Among 9036 twin pregnancies, cesarean delivery was associated with reduced odds of neonatal death compared to vaginal delivery at 22–24 + 6 weeks (aOR 0.35; 95% CI 0.19–0.69). When analysis was restricted to neonates who received active postnatal interventions (such as mechanical ventilation or surfactant), the overall mortality difference between modes of delivery was attenuated and not statistically significant (aOR 0.89; 95% CI 0.71–1.11). Importantly, within these actively treated neonates, a significant survival benefit for cesarean delivery remained only in those delivered at 24 to 24 + 6 weeks, with no significant difference observed at other gestational ages or beyond 25 weeks.

**Conclusion**: Cesarean delivery is associated with significantly lower neonatal mortality for extremely preterm twins between 22 and 24 weeks, with a persistent benefit among actively treated infants only at 24 to 24 + 6 weeks. No mortality difference was observed for cesarean delivery beyond 25 weeks or in other actively treated gestational age groups. Counseling for periviable twin pregnancies should balance these nuanced findings with maternal risk and institutional context.

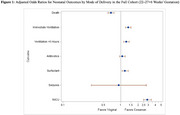

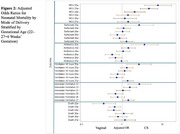

**10:30 AM – 12:00 PM**

## 211 | Maternal Disability and Risk of Neurodevelopmental Disorders in Offspring: A Nationwide Population‐Based Cohort Study

Hyungyu Park^1^; Dong Wook Shin^2^; Hea Lim Choi^2^; Jio Kim^3^; Ji Hoi Kim^1^; So Hee Kim^1^; Chan‐Wook Park^4^; Joong Shin Park^4^; Kyung Do Han^5^; Seung Mi Lee^6^

^1^Seoul National University Hospital, Seoul, Seoul‐t'ukpyolsi; ^2^Department of Clinical Research Design and Evaluation, Samsung Advanced Institute for Health Sciences & Technology (SAIHST), Sungkyunkwan University, Seoul, South Korea, Seoul, Seoul‐t'ukpyolsi; ^3^Department of Obstetrics and Gynecology, Seoul National University, Seoul, Korea, Seoul, Seoul‐t'ukpyolsi; ^4^Department of Obstetrics and Gynecology, Seoul National University Hospital, Seoul National University College of Medicine, Seoul, Republic of Korea, Seoul, Seoul‐t'ukpyolsi; ^5^Department of Statistics and Actuarial Science, Soongsil University, Seoul, Korea, Seoul, Seoul‐t'ukpyolsi; ^6^Department of Obstetrics and Gynecology, Seoul National University College of Medicine, Seoul, Seoul‐t'ukpyolsi

**Objective**: Women with disabilities are vulnerable to adverse perinatal outcomes, yet little is known about the long‐term neurodevelopmental outcomes in their children. This study aimed to assess the association between maternal disability and neurodevelopmental disorders (NDDs) in offspring, using a comprehensive national birth cohort.

**Study Design**: A retrospective cohort study was performed using data from the Korean National Health Insurance Service database linked to the Korea National Disability Registration System, including 3,515,478 births from 2009 to 2021. Maternal disability was categorized into five types: physical, visual, hearing, intellectual, and others. NDDs, including intellectual disability, developmental delay, and autism spectrum disorder, were identified via ICD‐10 codes. The primary outcome was NDD incidence after birth. Cox proportional hazards modeling was used to calculate adjusted hazard ratios (aHRs) and 95% confidence intervals (CIs), adjusting for maternal age, infant sex, sociodemographic factors, disability status, parity, delivery mode, multifetal gestation, pre‐pregnancy comorbidities, miscarriage history, and pregnancy complications.

**Results**: Among 18,983 children born to mothers with disabilities, the incidence rate of NDDs was 4.70 per 1000 person‐years versus 1.03 per 1000 in children of mothers without disabilities. Fully adjusted models showed significantly higher NDD risk (aHR 3.26; 95% CI: 3.03–3.51) in the disability group. Stratified analysis revealed the highest risk in offspring of mothers with intellectual disability (aHR 14.71; 95% CI: 13.30–16.27), followed by hearing (aHR 2.09), other disabilities (aHR 2.14), visual (aHR 1.65), and physical disability (aHR 1.27). All associations were statistically significant, with CIs excluding the null. (Figure)

**Conclusion**: Maternal disability is an independent risk factor for NDDs in offspring, with the greatest risk for maternal intellectual disability. These findings highlight the need for early developmental screening, targeted interventions, and comprehensive perinatal care for women with disabilities and their children.

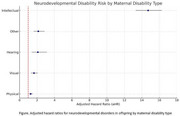

**10:30 AM – 12:00 PM**

## 212 | Rate of Mosaicism on Diagnostic Testing for Sex Chromosome Abnormalities on NIPT

Ifeoma M. Ogamba‐Alphonso^1^; Annie Rozenblyum^2^; Teresa Dunn^3^; Xiwei Yang^1^; Nicole Cacace^1^; Martin Chavez^2^; Anju Suhag^4^

^1^NYU Langone Hospital ‐ Long Island, Mineola, NY; ^2^NYU Grossman Long Island School of Medicine, Mineola, NY; ^3^CytoGenX Medical Genetic Laboratories, Stony Brook, NY; ^4^NYU Langone Long Island, Mineola, NY

**Objective**: Noninvasive prenatal testing (NIPT) is commonly used for aneuploidy screening, but its positive predictive value is variable for sex chromosome aneuploidies (SCA). Mosaicism may contribute to discordant results. This study aimed to assess the rate of fetal and placental mosaicism among cases with high‐risk NIPT results for SCA, including those with atypical sex chromosome findings.

**Study Design**: This retrospective review included patients with positive NIPT for SCA who underwent diagnostic testing between March 2020 and June 2025. Patients with incomplete records, contraindications to NIPT (e.g., organ/bone marrow transplant, hematologic malignancy) and normal NIPT results were excluded. Medical records were reviewed for demographics and karyotype results. The primary outcome was the rate of fetal & placental mosaicism. Fetal mosaicism was defined as mosaicism confirmed on amniocentesis karyotype, while confined placental mosaicism (CPM) was defined as mosaicism on chorionic villus sampling (CVS) with a subsequent normal amniocentesis karyotype. Descriptive analysis were used.

**Results**: Ninety‐one patients met inclusion criteria and, all underwent diagnostic testing. Of these, 38 cases (41.8%) were confirmed. Among confirmed cases, 26 (68.4%) were non‐mosaic and 12 (31.6%) were mosaic. Of the mosaic cases, 7 were fetal mosaic, 4 were CPM, and 1 showed mosaicism on CVS but was not confirmed on amniocentesis. The types of mosaic SCAs included 10 cases of mosaic monosomy X, and 2 cases of mosaic triple X syndrome. CVS was the most common diagnostic method, and 13% of patients who had CVS required follow‐up amniocentesis to clarify mosaicism type.

**Conclusion**: Mosaicism accounted for nearly one‐third of confirmed SCA cases, with both fetal and placental mosaicism observed. Mosaic monosomy X was the most frequently identified subtype. These findings underscore the critical role of diagnostic testing, including follow‐up procedures to differentiate true fetal SCA from confined placental mosaicism and guide appropriate counseling and shared clinical decision‐making.

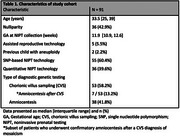

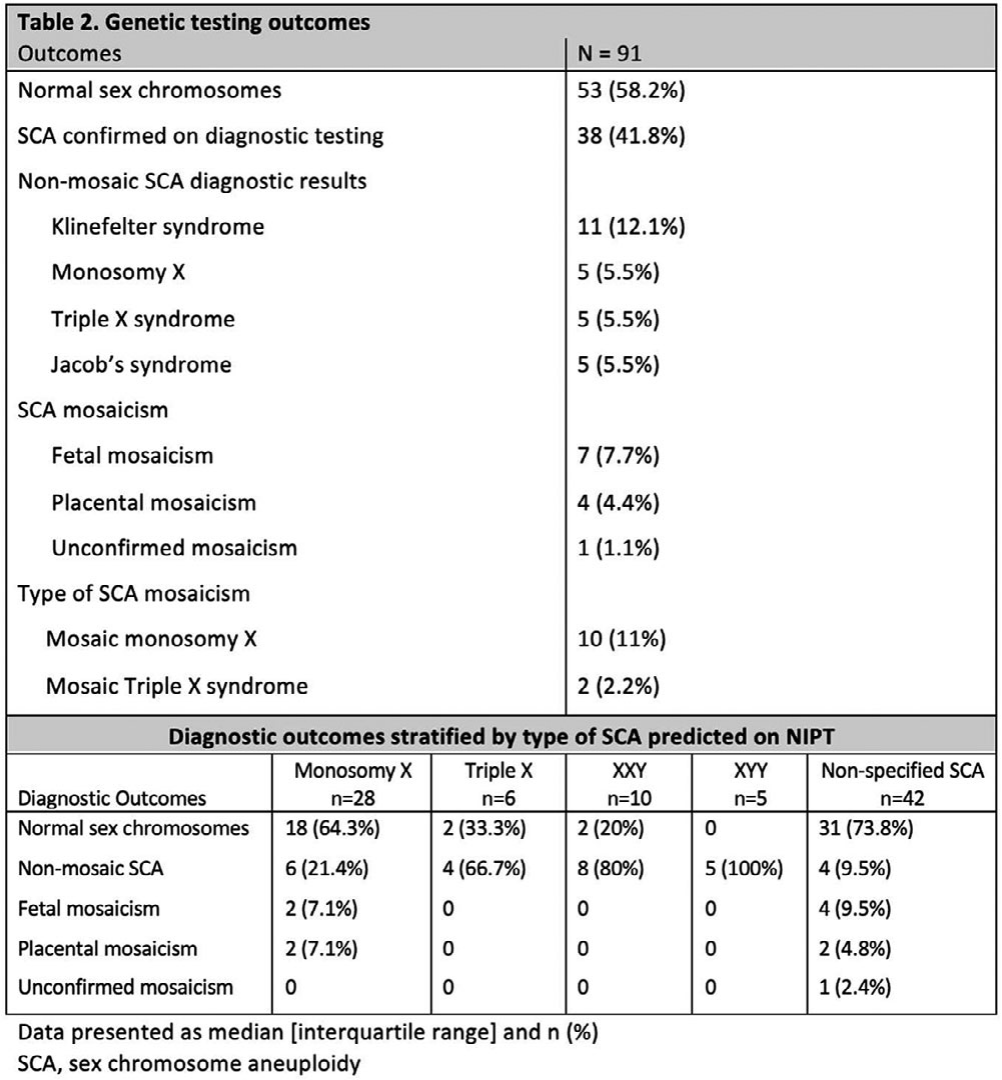

**10:30 AM – 12:00 PM**

## 213 | Migraine and Adverse Pregnancy Outcomes: A Large‐Scale Propensity Score Matched Cohort Study

Inbal Akavian^1^; Roie Alter^1^; Snir Tamari^2^; Hadar Rosen^1^; Doron Kabiri^1^

^1^Hadassah Ein‐karem hospital, Obstetrics and gynecology department, Jerusalem, Jerusalem, Yerushalayim; ^2^Hadassah Medical Center and Hebrew University of Jerusalem, Jerusalem, Israel, Hadassah Medical Center and Hebrew University of Jerusalem, Jerusalem, Israel, Yerushalayim

**Objective**: Migraine affects 15–20% of women of reproductive age, with peak incidence during the childbearing years. While previous studies have suggested an association between migraine and adverse pregnancy outcomes, findings have been inconsistent due to methodological limitations, such as small sample sizes and inadequate confounder control. This study aimed to evaluate the relationship between migraine and a comprehensive range of obstetric complications.

**Study Design**: We conducted a retrospective cohort study using the TriNetX Collaborative Network, which includes de‐identified electronic medical records from 70 healthcare organizations between March 2005, and January 2024.

Pregnant women aged 18–40 with a migraine diagnosis and no pre‐pregnancy hypertension diagnosis were matched 1:1 to pregnant women without migraine (*n* = 210,742 per group) using propensity scores based on age, race, BMI, tobacco use, and prior Cesarean delivery. Outcomes were defined using ICD‐10 codes, procedures, or laboratory values.

**Results**: Women with migraine demonstrated significantly increased risks across multiple adverse pregnancy outcomes compared to matched controls. The strongest associations were observed for hypertensive disorders: eclampsia (OR 1.87, 95% CI 1.66–2.10; 0.4% vs 0.2%), preeclampsia (OR 1.49, 95% CI 1.45–1.53; 7.3% vs 5.0%), and pregnancy‐induced hypertension (OR 1.50, 95% CI 1.47–1.54; 9.8% vs 6.7%). Migraine was also associated with increased risk of preterm labor (OR 1.53, 95% CI 1.50–1.57; 8.9% vs 6.0%), abortion (OR 1.31, 95% CI 1.29–1.34; 8.9% vs 6.9%), gestational diabetes mellitus (OR 1.23, 95% CI 1.20–1.26; 8.7% vs 7.2%), placental abruption (OR 1.28, 95% CI 1.21–1.35; 1.3% vs 1.0%), and intrauterine fetal death (OR 1.25, 95% CI 1.17–1.35; 0.8% vs 0.6%).

**Conclusion**: This large, matched propensity score cohort study demonstrates that migraine independently increases the risk of multiple adverse pregnancy outcomes. These findings indicate that migraine history should be considered an important risk marker in pregnancy, warranting enhanced surveillance and potentially preventive interventions for affected women.

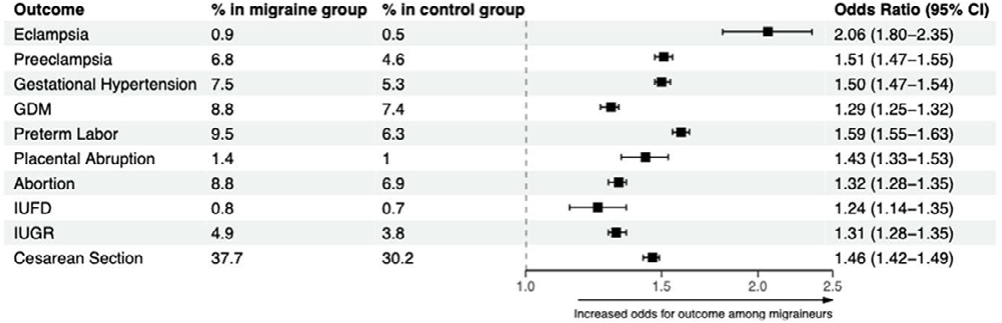

**10:30 AM – 12:00 PM**

## 214 | The Impact of Stepwise Interventions on NTSV Cesarean Rates and on Racial Inequities

India Eaford^1^; Mercy M. Shobiye^2^; Elizabeth West^1^; Allison S. Bryant^3^; Christina M. Duzyj^4^

^1^Massachusetts General Hospital, Massachusetts General Hospital/Boston, MA; ^2^Department of Obstetrics and Gynecology, MassGeneral Brigham; Division of Maternal‐Fetal Medicine; Harvard Medical School, Massachusetts General Hospital/Boston, MA; ^3^Massachusetts General Hospital, Boston, MA; ^4^Department of Obstetrics and Gynecology, MassGeneral Brigham; Division of Maternal‐Fetal Medicine; Harvard Medical School, Boston, MA

**Objective**: Nulliparous, term, singleton, vertex (NTSV) cesarean (CS) rate has emerged as a key quality indicator; however, strategies for rate reduction, as well as addressing racial inequities have been lacking. We evaluated whether our interventions improved our institutional rate.

**Study Design**: This retrospective review examined NTSV CS data over four years at a large tertiary institution, stratified by race. A stepwise series of interventions were implemented to improve patient outcomes (Table 1). The impact of the interventions was assessed using chi‐square tests. A sub‐analysis focused on rates among self‐identified Black individuals (Table 2).

**Results**: In the primary analysis, there was no statistically significant change in NTSV CS rate across the intervention series. Amongst all individuals, the pre‐intervention NTSV CS rate (three months prior) was 22.2% compared to 26.9% three months after the final intervention. In the sub‐analysis of Black individuals, two interventions showed statistically significant differences: development of the hospital working group (*p* = 0.014) and patient identification in safety rounds (*p* = 0.004). However, for both, the rates increased post‐intervention, indicating more cesarean deliveries. Amongst Black individuals, the pre‐intervention NTSV rate (three months prior to interventions) was 6.7%, rising to 54.5% after the last intervention.

**Conclusion**: Despite implementing a stepwise series of interventions aimed at reducing NTSV cesarean rates overall, and particularly among Black individuals, rates remained essentially unchanged. These findings suggest that no single intervention sufficiently lowers the NTSV CS rate, underscoring the need for continued innovation to address this important quality discrepancy.

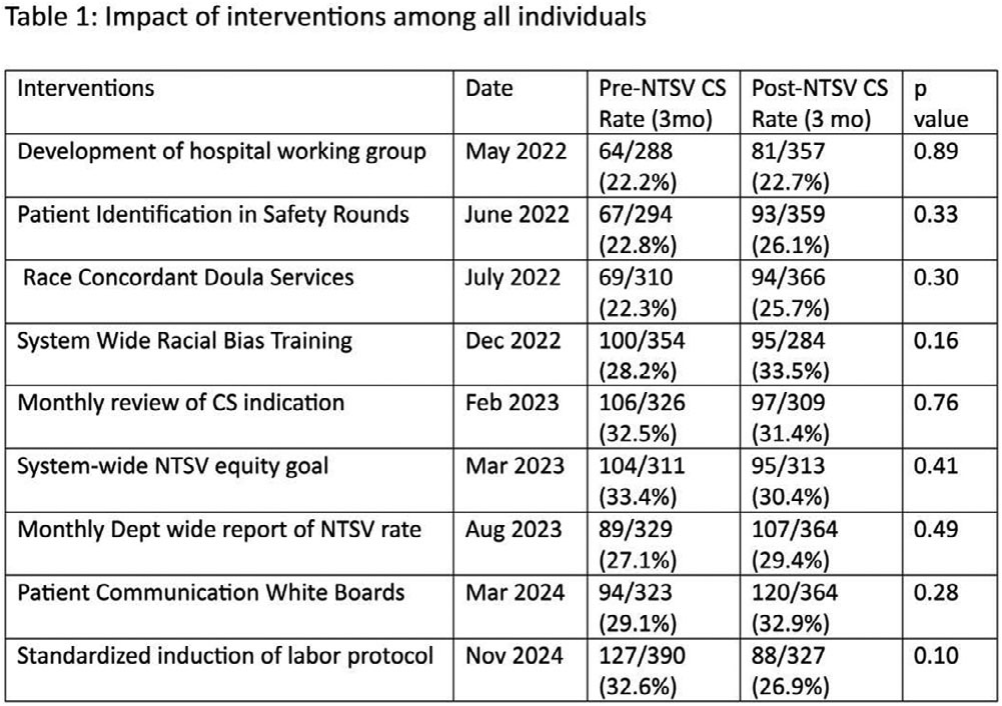

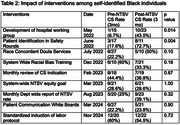

**10:30 AM – 12:00 PM**

## 215 | Updated Ultrasonographic Fetal Indicators of Congenital Syphilis

Irene A. Stafford; Donatella Gerulewicz; Sabrina C. DaCosta; Analuisa C. Mosqueda; Sean C. Blackwell; Edgar Hernandez‐Andrade

Division of Maternal‐Fetal Medicine, Department of Obstetrics, Gynecology and Reproductive Sciences, McGovern Medical School at UTHealth Houston, Houston, TX

**Objective**: This study aims to identify updated ultrasonographic (US) markers of congenital syphilis (CS) in exposed fetuses and examine their association with neonatal CS classification.

**Study Design**: Following institutional approval, a prospective study of pregnant women with syphilis and their neonates was performed between 05/23‐06/25 at the University of Texas in Houston. All consented participants >16 weeks gestational age (GA) underwent serial (US) evaluation within 7 days of maternal treatment and monthly thereafter. We evaluated several traditional US findings of CS (i.e., placentomegaly (>2SD for GA) and hepatomegaly (>90% for GA) versus longitudinal estimated fetal weight (EFW) and femur length (FL) measurement percentiles as predictors for neonatal CS categories. All measurements were compared to established nomograms. Logistic regression was used to evaluate associations between sonographic measurements and neonatal CS and linear mixed‐effects models for trends in fetal growth parameters and CS categories.

**Results**: Of 84 enrolled pregnant women, eleven (13%) were diagnosed with early syphilis and 47 (56%) were diagnosed <20 weeks GA. No fetal deaths were reported. Among 84 neonates, 16 (19%) were classified as confirmed/highly probable CS, 8 (10%) as possible CS, and 60 (71%) as less‐likely CS. Sixty‐four newborns (76%) had antenatal serial growth data. Placentomegaly was common across all CS groups (Table 1) and hepatomegaly was significantly associated with confirmed/highly probable CS (*p* = 0.002). In addition to these traditional CS findings, FL percentile decreased by 2.5 points (95% CI:−0.63,−1.39) and EFW percentile by 1.7 points (95% CI:−2.94,−0.46) at each successive scan. Neonates with FL ≤10th percentile had significantly higher odds of confirmed/highly probable CS (OR 3.63, 95% CI:1.02–12.9, *p* = 0.046) (Figure 1).

**Conclusion**: In addition to traditional US stigmata of CS, most fetuses exposed to syphilis experienced significant growth abnormalities, especially in FL. US growth assessment might be considered as part of an antenatal US evaluation in the context of syphilitic infection.

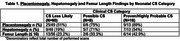

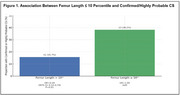

**10:30 AM – 12:00 PM**

## 216 | Patterns of Food Security and Iron Deficiency Anemia Among Black Pregnant Women

Jonathan Altamirano^1^; Ijeoma I. Iwekaogwu^2^; Tayler J. Hughes^1^; Kendra L. Smith^3^; Irogue I. Igbinosa^4^

^1^Stanford, Stanford, CA; ^2^California, Stanford, CA; ^3^Smith Research & Consulting, LLC, Houston, TX; ^4^Stanford University, Stanford, CA

**Objective**: Black pregnant people experience up to 5 times the risk of food insecurity when compared to other pregnant people. Among other social determinants of health and racism, food insecurity can lead to iron deficiency and iron deficiency anemia (IDA). Our study aimed to quantify nutrition knowledge, food security, and dietary behaviors among Black pregnant individuals and examine differences between those with and without IDA.

**Study Design**: We conducted structured interviews with 30 Black pregnant and postpartum women aged ≥18 from 2024–2025, living in California or Texas. Participants self‐reported IDA status, eligibility for the Women, Infants, and Children (WIC) program, and completed the United States Department of Agriculture's Household Food Security Survey Module. Scores were categorized as food secure (≤1) or food insecure (2–6), stratified into low (2–4) and very low (5–6) security. Differences were assessed using Fisher's exact test (*p* < 0.05).

**Results**: Of 30 participants, 15 (50%) reported IDA, 15 (50%) were WIC‐eligible, and 8 (27%) were both WIC‐eligible with IDA (Table 1). Overall food insecurity was comparable between IDA (46.7%) and non‐IDA (40.0%) groups. Very low food security was more common in IDA vs non‐IDA participants, although the difference was not statistically significant (20.0% vs. 6.7%, *p* = 0.76) (Figure 1). Food security was lower in WIC‐eligible participants. Comparing WIC‐eligible vs. ineligible participants, 46.7% vs. 66.7% were food secure, 26.7% vs. 33.3% had low food security, and 26.7% vs. 0% had very low food security (*p* = 0.13). WIC eligible participants with IDA were at the most risk for very low food security (*p* = 0.26).

**Conclusion**: Very low food security was more frequent among participants with IDA, WIC eligibility, or both. Though not statistically significant, this pattern highlights vulnerabilities potentially contributing to food insecurity. Our findings underscore the need for routine food insecurity screening in prenatal care and studies on culturally responsive interventions to reduce nutritional disparities among Black pregnant individuals.

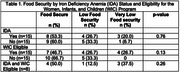

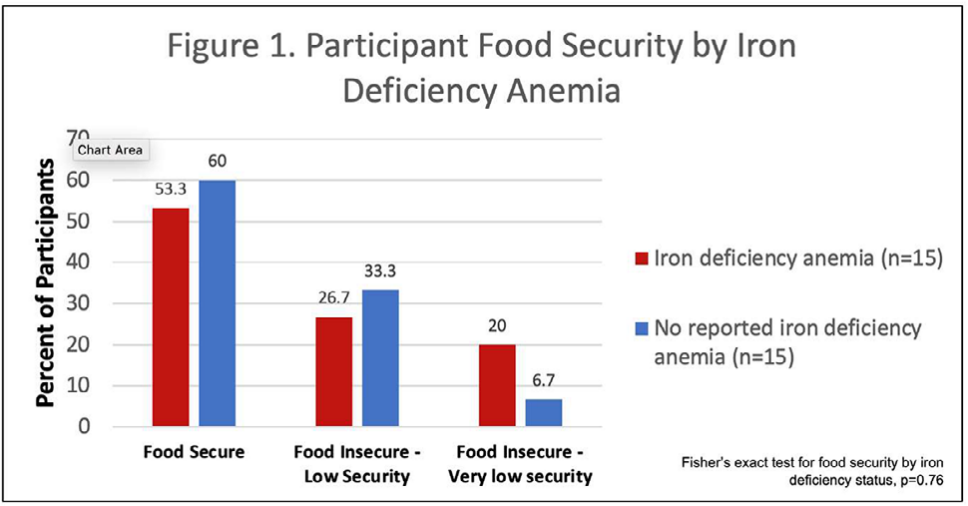

**10:30 AM – 12:00 PM**

## 217 | Child Opportunity Index (COI) and Maternal‐Newborn Outcomes: A Desirability of Outcome Ranking (DOOR) Analysis

Isabella A. Molina; Sarah H. Abelman; Frank I. Jackson; Oladunni Ogundipe; Matthew J. Blitz

Northwell, New Hyde Park, NY

**Objective**: To evaluate the association between child opportunity index (COI) and maternal‐newborn dyadic outcomes using the desirability of outcome ranking (DOOR) methodology. COI quantifies neighborhood‐level resources and conditions that support child development. We assessed how birth outcomes, ranked from most to least desirable, differed based on census tract‐linked COI.

**Study Design**: This retrospective cross‐sectional study included all deliveries eligible for the Joint Commission's PC‐06 quality metric (term newborns without preexisting conditions) at seven hospitals within a large New York health system from 2019–2024. Patient home addresses were matched to census tracts and linked to their corresponding COI scores. Patients with missing COI scores were excluded. Birth outcomes were classified using DOOR methodology into four groups based on delivery mode and maternal or neonatal complications: (1) uncomplicated vaginal delivery, (2) uncomplicated cesarean delivery, (3) vaginal delivery with complications, and (4) cesarean delivery with complications. Maternal complications were defined using CDC severe maternal morbidity indicators, and neonatal complications by PC‐06 criteria. Multinomial logistic regression assessed associations between COI and birth outcomes, adjusting for relevant covariates.

**Results**: A total of 113,451 deliveries were included. Birth outcomes by COI group are shown in the Figure; regression results are in the Table. Compared to very high COI neighborhoods, high and moderate COI were associated with lower odds of uncomplicated cesarean delivery and higher odds of vaginal delivery with complications. Low and very low COI were associated with higher odds of vaginal delivery with complications. COI was not significantly associated with cesarean delivery with complications.

**Conclusion**: Neighborhood COI is associated with the quality and desirability of birth outcomes, suggesting that community‐level disparities impact perinatal care.

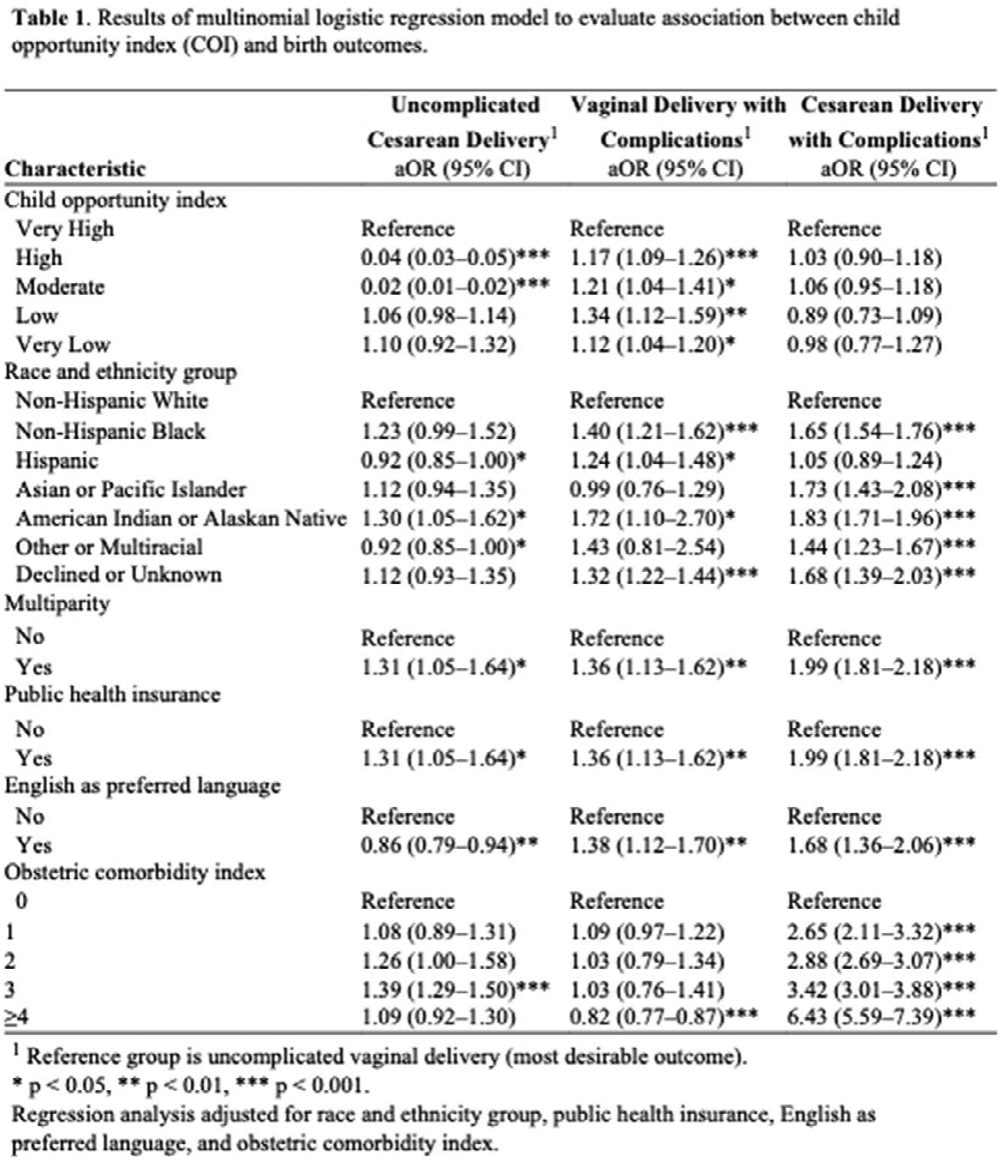

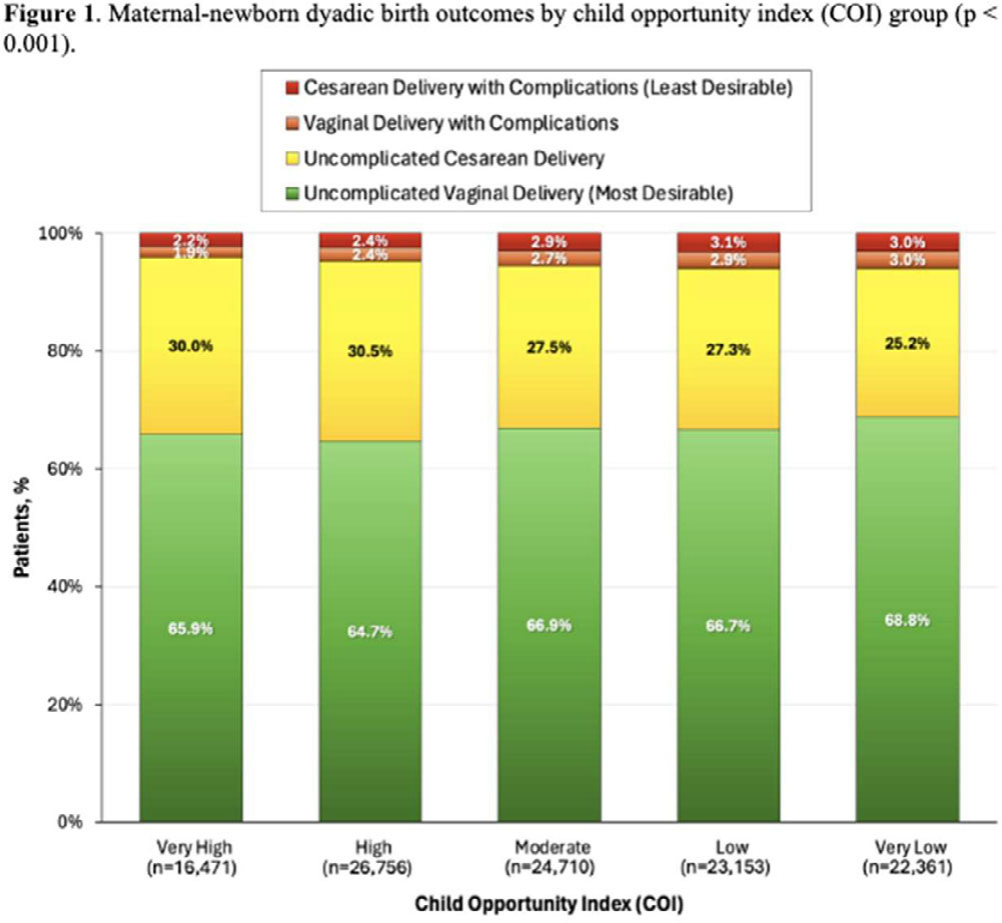

**10:30 AM – 12:00 PM**

## 218 | Consequences of Preconception Bariatric Surgery for Pathological Growth Restriction: A Linked Population Registry Study

Isabelle S. Hardy^1^; Jessica Liauw^1^; Olof Stephansson^2^; Martin Neovius^2^; Kari Johansson^2^; Jennifer Hutcheon^1^

^1^Division of Maternal Fetal Medicine, Department of Obstetrics and Gynaecology, University of British Columbia., Vancouver, BC; ^2^Clinical Epidemiology Division, Department of Medicine Solna, Karolinska Institutet, Stockholm, Stockholms Lan

**Objective**: To estimate the association between preconception bariatric surgery and fetal growth restriction (FGR) in Swedish women with obesity.

**Study Design**: We linked nationwide bariatric surgery, birth, and other health registries from Sweden. Births (2013–2021) after maternal bariatric surgery were paired to control births with 1:1 matching for parity, pre‐surgery body mass index, diabetes, and a propensity score of sociodemographic characteristics and maternal comorbidities. Births were classified as FGR using two definitions: 1) a postnatal Delphi consensus definition of FGR (Beune et al., 2018), and 2) a latent class model‐based definition that combined neonatal anthropometry, adverse neonatal outcomes, and maternal risk factors. We also examined a measure of small fetal size that does not necessarily imply pathology, small‐for‐gestational‐age birth (SGA, <10th percentile).

For each definition, conditional logistic regression was used to estimate the association between bariatric surgery and the odds of FGR with 95% confidence intervals. Proportions of adverse neonatal outcomes amongst SGA births were compared between births after bariatric surgery and matched controls.

**Results**: We included 5258 post bariatric surgery births and 5258 matched control births. Bariatric surgery was associated with increased odds of SGA birth (13.3% vs. 7.7%, respectively, odds ratio (OR) 1.85 [95% CI: 1.62, 2.11]). However, SGA births after bariatric surgery experienced fewer adverse neonatal outcomes than SGA births in controls (Table 1). When using the more rigorous definitions of FGR, the association between bariatric surgery and FGR was less pronounced (6.1% vs. 4.6% using Delphi definition, OR 1.35 [95% CI: 1.14, 1.61]) or no longer present (5.5% vs. 5.1% using latent class definition, OR 1.09 [95% CI: 0.92, 1.30]).

**Conclusion**: Preconception bariatric surgery was associated with increased odds of SGA birth, but these births had lower risks of adverse outcomes. The use of more rigorous definitions of FGR suggests that preconception bariatric surgery may not be associated with pathological FGR.

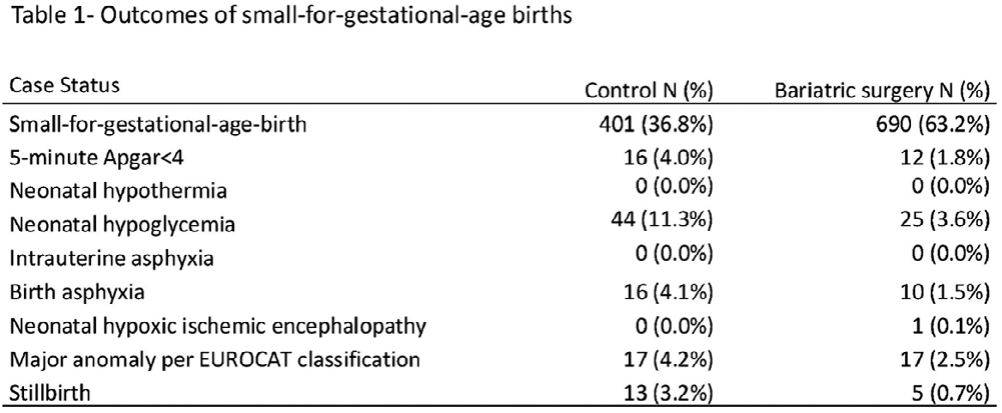

**10:30 AM – 12:00 PM**

## 219 | Association Between Maternal 50‐Gram Glucose Challenge Levels Within Normal Range and Long Term Offspring Morbidities

Israel Yoles^1^; Dana Shmuel^1^; Tuvia Baevsky^1^; Micha Barchana^2^

^1^Clalit Health Services, The Central District, Rishon Le Tzion, HaMerkaz; ^2^Bar Ilan University, Ramat GAn, HaMerkaz

**Objective**: The 50‐gram Glucose Challenge Test (GCT) is currently used only to determine whether a 100‐gram Oral Glucose Tolerance Test is required, while the absolute GCT value is often considered negligible. We have previously shown that high‐normal GCT results (126–139 mg/dL) are associated with increased risk of maternal cardiometabolic diseases later in life, compared with low‐normal values. This study evaluated the association between high versus low‐normal 50‐gram GCT results in pregnancy and long‐term pediatric health outcomes.

**Study Design**: This population‐based retrospective cohort included 35,478 children born to 21,304 mothers with documented GCT results and complete follow‐up in a large HMO between 2000–2024. Infants with birth weight <2000 g, gestational age <35 weeks, and multiple births were excluded. Maternal GCT results were grouped as ≤125, 126–139, and ≥140 mg/dL. Pediatric morbidities were assessed over 10 years using Kaplan–Meier time‐to‐event analysis and log‐rank tests. Multivariable Cox models estimated adjusted hazard ratios, controlling for maternal age, socioeconomic status, birth weight, gestational age, and offspring sex.

**Results**: High‐normal maternal GCT (126–139 mg/dL) was associated with higher 10‐year cumulative incidence of childhood obesity (10.9% vs 12.6% vs 12.9%, *p* = 0.0011), autism (*p*  = 0.0012), dermatitis (*p*  = 0.0216), congenital anomalies (*p*  = 0.0172), and psychiatric disorders (*p*  = 0.0326). Cox models confirmed that high maternal glucose, male sex, and advanced maternal age increased risk, whereas favorable socioeconomic status was protective.

**Conclusion**: Maternal high‐normal GCT results, although within the non‐diabetic range, are associated with increased long‐term pediatric morbidities, particularly obesity and neurodevelopmental disorders. These findings indicate that women in the high‐normal GCT group, despite being classified as non‐diabetic, merit specific guidance and follow‐up during pregnancy and underscore the need for more attentive long‐term pediatric care for their children.

**10:30 AM – 12:00 PM**

## 220 | **Clinical Predictors and Surgical Intervention in Postpartum Vaginal and Vulvar Hematomas: A Matched Case‐Control Study**

Itai Atar^1^; Einat Tako^1^; Daniel Gabbai^2^; Itamar Gilboa^2^; Yariv Yogev^3^; Emmanuel Attali^2^

^1^Lis Hospital for Women's Health, Tel Aviv Sourasky Medical Center, Tel Aviv, Israel, Tel Aviv, Tel Aviv; ^2^Lis Hospital for Women's Health, Tel Aviv Sourasky Medical Center, Lis Hospital for Women's Health, Tel Aviv Sourasky Medical Center, Tel Aviv; ^3^Lis Hospital for Women's Health, Tel Aviv Sourasky Medical Center Gray Faculty of Medicine, Tel Aviv University, Israel, Lis Hospital for Women's Health, Tel Aviv Sourasky Medical Center, Tel Aviv

**Objective**: To identify risk factors for postpartum vaginal or vulvar hematoma and early clinical predictors of surgical intervention.

**Study Design**: A retrospective matched case‐control study was conducted at a tertiary, university‐affiliated center (2012–2024). Women diagnosed with vaginal or vulvar hematoma (cases) during the postpartum hospitalization were matched 1:3 by maternal and gestational age to controls. Independent risk factors were identified using conditional logistic regression with a Cox regression model. A risk score was derived from the multivariable model, and ROC analysis assessed performance. A subgroup analysis evaluated predictors of surgery.

**Results**: Among 147,045 deliveries, 57 women (0.039%) had postpartum hematoma; 170 matched controls were included. Diagnosis occurred 1.0 ± 1.6 days after delivery. Hematoma was associated with vacuum‐assisted delivery (21.1% vs 4.7%, *p* < 0.001), episiotomy (24.6% vs 2.4%, *p* < 0.001), second‐degree laceration (38.6% vs 15.3%, *p* < 0.001), nulliparity, and lower body mass index (BMI; *p* < 0.001). In multivariable analysis, episiotomy (OR 35.9, 95% CI 5.6–232.4), second‐degree laceration (OR 10.3, 95% CI 3.2–32.9), and vacuum delivery (OR 5.0, 95% CI 1.0–24.5) independently increased risk, whereas higher BMI (OR 0.69/unit, 95% CI 0.55–0.85) and epidural analgesia (OR 0.33, 95% CI 0.12–0.93) were protective. The model's AUC was 0.83; a score ≥3 yielded 68% sensitivity and 79% specificity. Among women with hematoma, 26.3% required surgery. Time to surgery averaged 6.5 ± 3.8 hours. Prolonged second stage (20% vs 2.4%, *p* < 0.05) was significantly associated with surgery, with trends toward more vacuum deliveries, no epidural, and greater hemoglobin decline. Postpartum hemorrhage and blood transfusion rates did not differ.

**Conclusion**: Postpartum hematoma, though rare, follows a recognizable pattern. Vacuum‐assisted delivery, episiotomy, second‐degree laceration, and low BMI increase its risk, whereas epidural analgesia is protective. Early recognition may aid risk stratification, monitoring, and timely management.

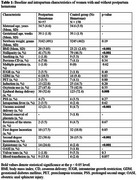

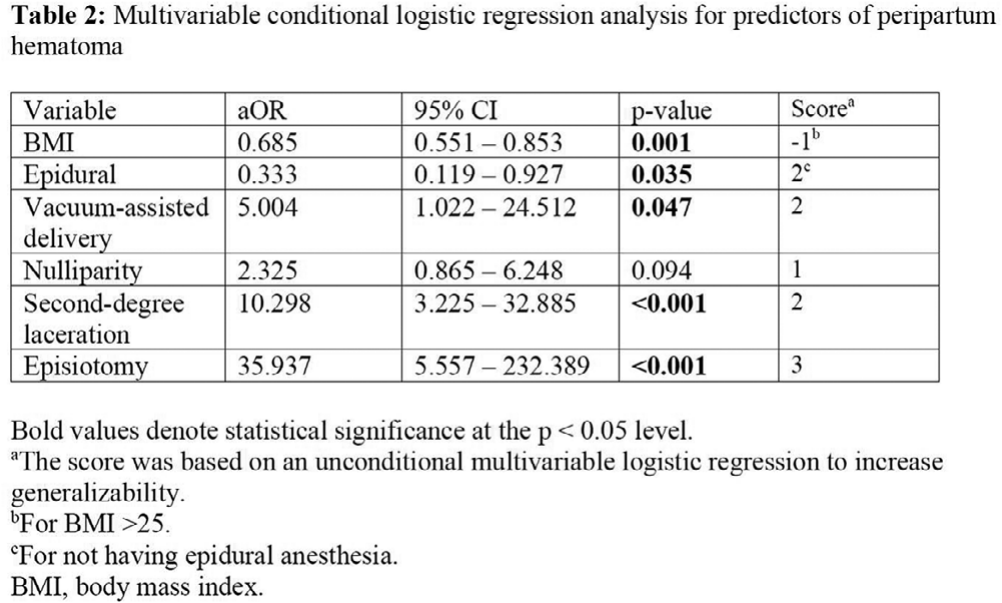

**10:30 AM – 12:00 PM**

## 221 | Vacuum‐Induced Uterine Tamponade for Atonic Postpartum Hemorrhage: A First‐in‐Human Descriptive Pilot Study

Vajdana Tomić^1^; Dejan Tirić^1^; Nikolina Penava^1^; Ivona Šutalo Alilović^1^; Vedrana Mandrapa^1^; Ivona Margeta^1^; Ana Damjanović^1^; Mario Tomić^1^; Osoti Alfred^2^; Gwako John^2^; Michael Stark^3^; Itamar Futterman^4^; Scott Chudnoff^5^; Agnes J. Karongo^2^; Wachira Kanyiri^6^; Omondi Ogutu^2^

^1^University of Mostar, University of Mostar, Federation of Bosnia and Herzegovina; ^2^The University of Nairobi, The University of Nairobi, Nairobi Area; ^3^New European Surgical Academy, New European Surgical Academy, Berlin; ^4^Maimonides Medical Center, Maimonides Health, NY; ^5^Maimonides Medical Center, Brooklyn, NY; ^6^The University of Nairobi, The University of Nairobi, North‐Eastern

**Objective**: To evaluate the safety and performance of a novel vacuum‐induced uterine tamponade device (Alma) for the treatment of postpartum hemorrhage (PPH) following failure of first‐line medical treatment.

**Study Design**: A prospective, open‐label pilot study was conducted in two tertiary care centers between 04/2024‐03/2025. Participants included patients with term pregnancies who were recruited upon arrival for delivery and who's birthing process was complicated by PPH secondary to atony. Variables collected included time to bleeding control, blood loss, transfusion requirements, incidence of adverse events as well as usability ratings (scoring each of the following: setup, insertion, inflation, vacuum and deflation).

**Results**: Sixteen (16) women with PPH following vaginal (n = 13) or cesarean (n = 3) delivery, who did not respond to standard first‐line interventions, were treated using the Alma device. All 16 patients achieved prompt bleeding control, with restoration of uterine tone occurring within an average of 2.6 ± 0.8 minutes. The estimated blood loss prior to treatment averaged 878.1± 222.8 mL. The measured post‐treatment blood loss averaged 115.5 ± 71.6 mL. No device‐related complications or subsequent re‐interventions were reported during 6‐week follow‐up. Red blood cell transfusions were administered in 7/16 (43.7%) of cases, while fresh frozen plasma was required in 4/16 (25.0%). Usability ratings were consistently high, with a mean score of 4.5/5 ±0.34 (Table 1).

**Conclusion**: This study suggests that vacuum‐induced uterine tamponade as applied using the Alma device, may offer a safe, rapid, and effective approach for controlling atonic PPH. Further, well designed, research is needed to assure product safety and effectiveness.

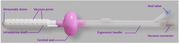

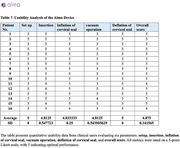

**10:30 AM – 12:00 PM**

## 222 | Impact of a Real‐Time Intrapartum Monitoring Dashboard on Delivery Outcomes

Itamar Gilboa^1^; Daniel Gabbai^1^; Emmanuel Attali^1^; Yariv Yogev^2^; Anat Lavie^1^

^1^Lis Hospital for Women's Health, Tel Aviv Sourasky Medical Center, Lis Hospital for Women's Health, Tel Aviv Sourasky Medical Center, Tel Aviv; ^2^Lis Hospital for Women's Health, Tel Aviv Sourasky Medical Center Gray Faculty of Medicine, Tel Aviv University, Israel, Lis Hospital for Women's Health, Tel Aviv Sourasky Medical Center, Tel Aviv

**Objective**: In January 2023, our center introduced a real‐time intrapartum digital monitoring dashboard, allowing clinicians in each of the 16 delivery rooms to view continuous fetal heart rate and contraction tracings from all laboring patients. We aimed to determine whether this intervention influenced delivery outcomes.

**Study Design**: A retrospective cohort study at a single university‐affiliated tertiary medical center with approximately 13,000 deliveries annually. All women ≥24 weeks’ gestation who underwent a trial of labor between January 2019 and December 2024 were included. Cesarean deliveries (CD) without labor and non‐viable fetuses were excluded. Patients were grouped into pre‐implementation (January 2019–December 2022) and post‐implementation (January 2023–December 2024) cohorts. The primary outcome was operative delivery (cesarean delivery or vacuum‐assisted delivery [VAD]). Secondary outcomes included prolonged second stage (PSS), third‐ or fourth‐degree perineal injury, and 5‐minute Apgar <7. Multivariable logistic regression adjusted for key clinical covariates.

**Results**:

1. A total of 59,046 deliveries were analyzed—33,780 (57.2%) pre‐implementation and 25,266 (42.8%) post‐implementation.
2. Cesarean delivery occurred in 5147 women (11.5%) in the pre‐implementation group and 1820 (9.2%) in the post‐implementation group (*p* < 0.001).
3. Vacuum‐assisted delivery increased from 4606 (10.3%) to 2236 (11.2%) (*p* < 0.001).
4. No significant differences were observed in the rates of prolonged second stage (6.0% vs. 5.8%), third‐ or fourth‐degree perineal injury (0.5% vs. 0.5%), or 5‐minute Apgar <7 (0.9% vs. 1.1%).
5. In multivariable analysis, dashboard implementation was independently associated with reduced odds of intrapartum CD (aOR 0.84, 95% CI 0.79–0.89, *p* < 0.001), without increased odds of VAD (aOR 1.04, 95% CI 0.98–1.10, *p* = 0.186).

**Conclusion**: Implementation of a continuous intrapartum digital dashboard was associated with a significant reduction in intrapartum cesarean rates, without adverse maternal or neonatal effects. This tool may enhance clinical awareness and support proactive labor management.

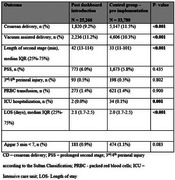

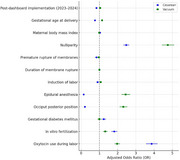

**10:30 AM – 12:00 PM**

## 223 | Continuous Glucose Monitoring (CGM) During 1‐Hour Oral Glucose Tolerance Testing (OGTT)

Ivonne Verduzco^1^; Allen M. Zhou^2^; Nathaniel E. Santoso^2^; Michael A. Kohn^3^; Nasim C. Sobhani^3^

^1^UCSF, San Francisco, CA; ^2^UC Berkeley, UC Berkeley/Berkeley, CA; ^3^UCSF, UCSF/San Francisco, CA

**Objective**: Although it is the standard approach to the diagnosis of gestational diabetes (GD), the oral glucose tolerance test (OGTT) has many disadvantages. Continuous glucose monitors (CGMs) may circumvent those disadvantages. A study was designed to examine the diagnostic utility of 7–10 days of CGM wear prior to the OGTT. The main objective of this secondary analysis was to explore what CGMs reveal about glucose dynamics during the OGTT.

**Study Design**: Gravidas without pre‐existing diabetes at 24–28 weeks wore a blinded Dexcom G6 CGM prior to and during the 1‐hour OGTT. For this analysis of CGM readings during the OGTT, the outcomes were 1) screen positive OGTT (1‐hour blood glucose [BG] ≥ 130 mg/dL) and 2) subsequent diagnosis of GD. CGM glucose and venous BG at 60 minutes were also compared.

**Results**: Of 75 study participants, 27 (36%) had a positive OGTT and 6 (8%) were diagnosed with GD. Mean glycemic excursion on CGM during the 1‐hour OGTT was 11.5 mg/dL higher in the screen‐positive group vs the screen‐negative group (*p* = 0.02) and 13.6 mg/dL higher in those with GD vs those without GD (*p* = 0.06). Five participants (*n* = 2 screen‐positive, *n* = 3 screen‐negative) were excluded from the 60‐minute glucose comparison due to early CGM removal. Compared to venous BG, CGM glucose was 20.3 mg/dL higher on average. CGM correctly identified elevated BG (≥ 130 mg/dL) in 23 out of 25 (92%) screen‐positive cases but was incorrectly elevated in 21 out of 45 (47%) of screen‐negative cases (*p* < 0.001).

**Conclusion**: Mean glycemic excursion on CGM worn during OGTT is significantly higher among those who screen positive compared to those who screen negative and substantially higher among those with GD compared to those without GD. Importantly, CGM readings at 60 minutes were significantly higher than venous BG. If CGM is used to screen for or diagnose GD, a higher cutoff value should be utilized to avoid false positives, since CGM readings lag venous BG when BG is decreasing.

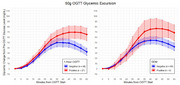

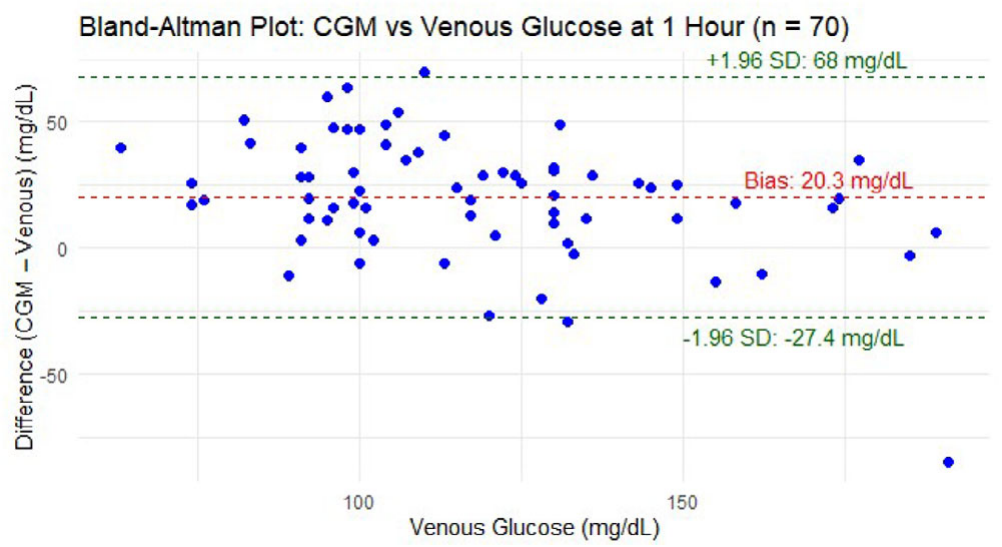

**10:30 AM – 12:00 PM**

## 224 | Racial Differences in Maternal Perceptions of Respectful Care During Childbirth

Jada Lee^1^; Shammah Kodjo‐Soroh^2^; Ri'enna Boyd^3^; Anthony J. Nixon, Jr.^4^; Taylor D. Powell^5^; Harry A. Obeng^3^; Shondra Clay^6^; Bridget C. Huysman^7^; Jeannie C. Kelly^7^; Nandini Raghuraman^8^; Candice L. Woolfolk^7^

^1^Washington University in St. Louis; ^2^Maimonides Medical Center, Washington University, MO; ^3^School of Public Health, Washington University in St. Louis, Washington University in St. Louis, MO; ^4^School of Public Health, Washington University in St. Louis, St. Louis, MO; ^5^Williams and Associates, Inc., St. Louis, MO; ^6^Northern Illinois University, DeKalb, IL; ^7^Washington University School of Medicine, Washington University School of Medicine, MO; ^8^Washington University School of Medicine, St. Louis, MO

**Objective**: Racial disparities in respectful maternity care are prevalent in the United States; BIPOC birthing people are more likely to report disrespect, leading to medical mistrust and exacerbating inequities in perinatal outcomes. Maternal perceptions of respectful care in patients experiencing labor interventions are not well known. We sought to examine differences in patient‐reported measures of respect by race in patients experiencing an intervention in labor.

**Study Design**: We conducted a prospective study of 200 patients presenting for delivery with a labor induction or unplanned cesarean from September 2024 to April 2025. Patients completed the Mothers on Respect Index (MORi) survey; responses were categorized by high/less than high respect (score ≥/<67). Survey scores were compared by race using chi‐squared and Mann Whitney U tests. Multivariable logistic regression was used to estimate adjusted odds ratios (aOR) and 95% confidence intervals (CI), adjusting for relevant confounders.

**Results**: Of the 200 patients, 118 (59.0%) were BIPOC and 82 (41.0%) were White. BIPOC patients had significantly lower total MORi scores than White patients (77.28 ± 9.81 vs. 80.02 ± 7.69, *p* = 0.03). BIPOC patients had almost three times the odds of reporting less than high respect, after adjusting for delivery mode (aOR: 2.93, 95% CI: 1.04, 8.21). Among the individual survey components, BIPOC patients reported poorer treatment due to race/ethnicity (*p* < 0.01), sexual orientation (*p* < 0.01), insurance (*p* < 0.01), and differences in opinion regarding care (*p* = 0.03).

**Conclusion**: BIPOC patients reported lower total MORI scores compared to White patients, indicating less than ideal respectful maternity care. This disparity was driven by lower scores related to perceived poor treatment due to race, sexual orientation, insurance status, and differences in opinions regarding care. These findings highlight inequities in patient‐reported quality of maternity care and the need for targeted interventions to improve culturally responsive communication and promote equitable care for all birthing people.

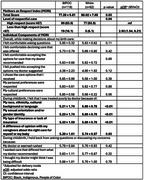

**10:30 AM – 12:00 PM**

## 225 | A Polysocial Risk and Primary Cesarean Delivery

Jameaka L. Hamilton^1^; William A. Grobman^2^; Jiqiang Wu^3^; Sadiya S. Khan^4^; Lynn Yee^5^; David M. Haas^6^; Rebecca B. McNeil^7^; Jessica Pippen^8^; Hyagriv Simhan^9^; Uma M. Reddy^10^; Robert M. Silver^11^; Lisa D. Levine^12^; George R. Saade^13^; Jun Wu^14^; Courtney C. Lynch^15^; Eric Jelovsek^16^; Kartik K. Venkatesh^17^

^1^The Ohio State University, Columbus, OH; ^2^Warren Alpert Medical School of Brown University, Providence, RI; ^3^The Ohio State University Wexner Medical Center, Columbus, OH; ^4^Northwestern University, Chicago, IL; ^5^Northwestern University Feinberg School of Medicine, Chicago, IL; ^6^Indiana University School of Medicine, Indianapolis, IN; ^7^RTI International, Triangle Park, NC; ^8^MetroHealth Medical Center, Cleveland, OH; ^9^University of Pittsburgh Medical Center, Pittsburgh, PA; ^10^Columbia University Irving Medical Center, New York, NY; ^11^Department of Obstetrics and Gynecology, University of Utah Health, Salt Lake City, UT; ^12^Perelman School of Medicine, University of Pennsylvania, Philadelphia, PA; ^13^Eastern Virginia Medical School at Old Dominion University, Norfolk, VA; ^14^University of California, Irvine, Orange, CA; ^15^Ohio State University Wexner Medical Center, COLUMBUS, OH; ^16^Duke University, Raleigh, NC; ^17^The Ohio State University Wexner Medical Center, The Ohio State University Wexner Medical Center, OH

**Objective**: While social determinants of health (SDOH) have been assessed as individual exposures in pregnant populations, a polysocial risk score that concurrently integrates multiple SDOH factors remains to be investigated. We developed a polysocial risk score and assessed whether it was associated with cesarean delivery (CD) among nulliparous individuals who delivered a term singleton in the vertex presentation.

**Study Design**: A secondary analysis of data from the prospective Nulliparous Pregnancy Outcomes Study: Monitoring Mothers‐To‐Be observational cohort. A total of 19 individual and neighborhood‐level SDOH factors conceptualized per 2030 Healthy People Domains were measured in early pregnancy. The outcome was CD. Sparse Principal Components Analysis was used to develop a polysocial risk score. Modified Poisson regression was used to determine the association between this polysocial risk score and CD as both a continuous measure and categorized into tertiles after adjustment for age, body mass index, chronic hypertension, and pregestational diabetes. Model discrimination per the c‐statistic was done to assess the capability of the polysocial risk score to predict CD.

**Results**: Among 9012 eligible individuals, 24% experienced a CD. Those who had a CD were generally more likely to experience adverse SDOH factors. Among 19 candidate SDOH factors (6 neighborhood‐ and 13 individual‐level), the polysocial risk score included a total of 11 variables using Sparse Principal Components Analysis for variable selection (Figure). In adjusted analyses, a higher polysocial risk score, whether evaluated as a continuous measure (aOR: 1.63; 95% CI: 1.35, 1.98) or after categorization in tertiles (T2 vs. T1: 40.9% vs. 26.1%; aRR: 1.35; 95% CI: 1.19, 1.53; and T3 vs. T1: 33.1% vs. 26.1%; aRR: 1.44; 95% CI: 1.25, 1.66) was associated with CD. The polysocial risk score provided satisfactory discrimination with a c‐statistic of 0.68 (95% CI: 0.662, 0.688).

**Conclusion**: A polysocial risk score was associated with an increased risk of CD among individuals who delivered term singletons in the vertex presentation.

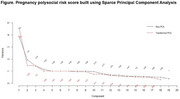

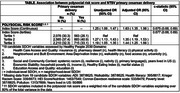

**10:30 AM – 12:00 PM**

## 226 | COVID‐19 Pandemic Impact on Perinatal Vaccination Trends

James D. Toppin^1^; Talia Suner^1^; Brian W. James^1^; Joseph R. Biggio, Jr.^2^; Frank B. Williams^3^

^1^Ochsner Clinic Foundation, New Orleans, LA; ^2^Ochsner Health, New Orleans, LA; ^3^Ochsner Clinic Foundation, Ochsner Clinic Foundation, LA

**Objective**: During the COVID‐19 pandemic, vaccinations during pregnancy became a contentious topic. We hypothesized that the pandemic has been associated with decreased uptake of maternal and neonatal vaccination rates using interrupted time series analysis.

**Study Design**: We performed a retrospective cohort study of 103,549 births from 2016 to 2024 from a large regional health system with 12 delivery hospitals in the American South. An interrupted time series with segmented regression was used to assess trends in maternal Tdap, neonatal hepatitis B, and coordinated vaccination rates. The COVID‐19 interruption point was defined as March 1, 2020. Age‐stratified analysis was performed to identify vulnerable populations.

**Results**: Pre‐COVID vaccination rates increased monthly for maternal Tdap (+0.34 percentage points per month), neonatal hepatitis B (+0.13 percentage points per month), and coordinated vaccination (+0.51 percentage points per month, all *p* < 0.001). COVID‐19 produced non‐significant immediate declines but significant sustained trend reversals for maternal Tdap (‐0.81 percentage points per month), neonatal hepatitis B (‐0.30 percentage points per month), and coordinated vaccination (‐1.04 percentage points per month, all *p* < 0.001, Figure 1). Age stratification revealed heterogeneous immediate COVID impacts with mothers aged 40 years and older experiencing the largest decline (‐7.6 percentage points), while mothers aged 25–29 years showed immediate improvement (+1.7 percentage points). All age groups demonstrated post‐COVID declining trends.

**Conclusion**: At a large regional health system in the South, COVID‐19 reversed favorable pre‐pandemic maternal and neonatal vaccination trends, with age‐dependent immediate impacts but universally negative sustained effects. Targeted interventions addressing age‐specific vulnerabilities are essential for restoring perinatal vaccination coordination.

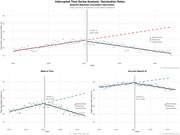

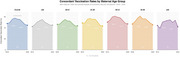

**10:30 AM – 12:00 PM**

## 227 | Diagnostic Accuracy of Point‐of‐Care Echocardiography for Detecting Cardiac Remodeling in Pregnant Patients With Chronic Hypertension

Jameson A. Mitchell^1^; Edward Chien^2^; Margaret Fuchs^1^; Nancy Obuchowski^1^; Karlee Hoffman^1^; Justin R. Lappen^1^

^1^Cleveland Clinic Foundation, Cleveland, OH; ^2^Cleveland Clinic Foundation, Cleveland Clinic Foundation, OH

**Objective**: Pathologic cardiac remodeling is common in pregnancies complicated by stage 2 chronic hypertension (cHTN) and is associated with adverse outcomes. This study evaluated the diagnostic accuracy of point‐of‐care echocardiography (POC‐E) compared to transthoracic echocardiography (TTE) in detecting left ventricular (LV) remodeling in pregnant patients with cHTN.

**Study Design**: We conducted an IRB‐approved prospective cohort study of pregnant patients aged 18–45 with stage 2 cHTN. Exclusion criteria included multiple gestation, abnormal aneuploidy screening, major fetal anomalies, congenital or acquired heart disease, and connective tissue disorders. All participants underwent POC‐E at enrollment, followed by TTE within 4 weeks. A parasternal long‐axis image was obtained at end‐diastole to measure LVID, IVS, and PWT to calculate left ventricular mass index (LVMI) and Relative Wall thickness (RWT) (Figure 1). Remodeling was defined as RWT >0.42 or LVMI >0.95. The primary outcome was the diagnostic accuracy of POC‐E for detecting cardiac remodeling by receiver operating characteristic analysis compared to TTE. Secondary outcomes included associations between remodeling and adverse maternal or perinatal outcomes. Assuming a remodeling prevalence of 51%, we calculated that 68 participants would provide 80% power (α = 0.05) to detect an AUC >0.7.

**Results**: 190 patients were screened and 83 were enrolled. Of these, 61 (73%) completed both POC‐E and TTE and were included in the primary analysis. The median interval between the POC‐E and TTE was 4 weeks (IQR 3, 7). The estimated AUC of POC‐E for detecting remodeling was 0.56 (95% CI: 0.41–0.70) (Figure 2). Sensitivity and specificity were 51.7% and 59.4%, respectively. No significant associations were found between TTE‐assessed LV remodeling and adverse maternal or perinatal outcomes.

**Conclusion**: In pregnant patients with cHTN, POC‐E demonstrated limited accuracy in diagnosing LV remodeling. While POC‐E can be utilized for qualitative cardiac evaluation, further studies are needed to assess if quantitative applications can be validated in an obstetric population.

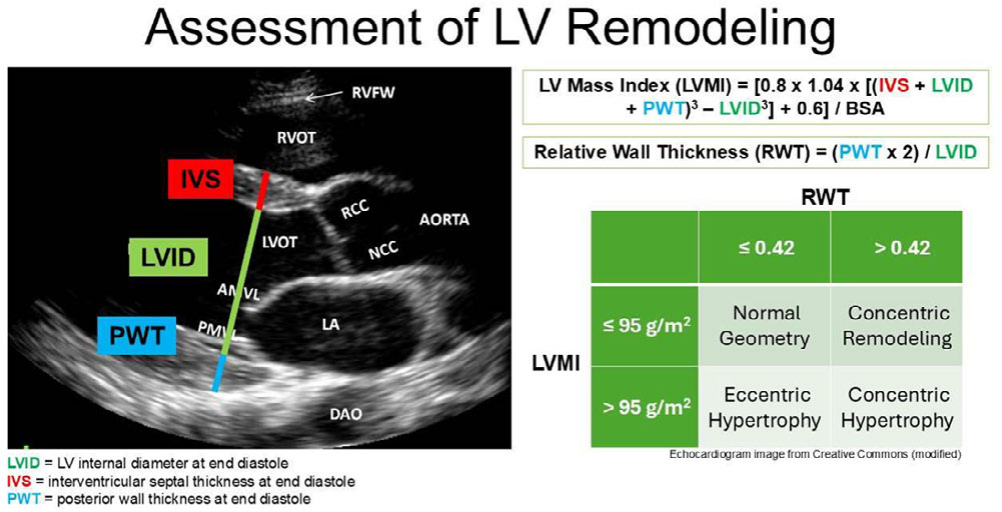

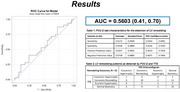

**10:30 AM – 12:00 PM**

## 228 | Maternal Mortality and Severe Maternal Morbidity Among Jehovah's Witnesses and Others Declining Blood Products

Jamie Green^1^; Sarah H. Abelman^1^; Frank I. Jackson^1^; Oladunni Ogundipe^1^; Luis A. Bracero^1^; Jeanne Woods Ludwig^2^; Fernando Suarez^1^; Adriann Combs^1^; Burton Rochelson^1^; Matthew J. Blitz^1^

^1^Northwell, New Hyde Park, NY; ^2^Northwell, Northwell / Manhasset, NY

**Objective**: To evaluate the incidence of maternal mortality and non‐transfusion severe maternal morbidity (SMM) among Jehovah's Witnesses and other patients who decline blood products, compared to the general obstetric population.

**Study Design**: This retrospective cross‐sectional study included all deliveries at seven hospitals within a large New York health system from 2019–2024. There were no exclusion criteria. The primary exposure was blood product refusal, documented at admission via a standardized intake question for all patients. Outcomes were inpatient maternal mortality and non‐transfusion SMM, with maternal mortality rates (MMR) calculated per 100,000 live births. Multivariable logistic regression modeled the association between blood product refusal and non‐transfusion SMM, adjusting for race and ethnicity group, health insurance, preferred language, multiparity, and obstetric comorbidity index (OB‐CMI) score, which is calculated based on 24 weighted comorbidity indicators identified through ICD‐10 codes and clinical documentation.

**Results**: A total of 160,516 pregnancies were included. Blood product refusal was documented in 1107 cases, of which 373 identified as Jehovah's Witnesses. There were 8 maternal deaths overall, with 1 occurring among Jehovah's Witnesses. The MMR was 90.3 per 100,000 live births for patients declining blood products and 4.4 per 100,000 for others (risk ratio 20.6; 95% CI 2.5–169). Among Jehovah's Witnesses specifically, the MMR was 268.1 per 100,000 (risk ratio 61.3; 95% CI 7.5–498). Non‐transfusion SMM occurred in 2.4% of patients declining blood products versus 1.6% in others (aOR 1.43; 95% CI 0.95–2.07). Among Jehovah's Witnesses, the non‐transfusion SMM rate was 3.5% (aOR 1.82; 95% CI 0.98–3.07).

**Conclusion**: Patients who declined blood products had over 20‐fold higher maternal mortality, and Jehovah's Witnesses had more than 60‐fold higher mortality compared to the general obstetric population. Non‐transfusion SMM was also higher among these groups, though not statistically significant.

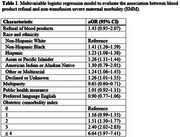

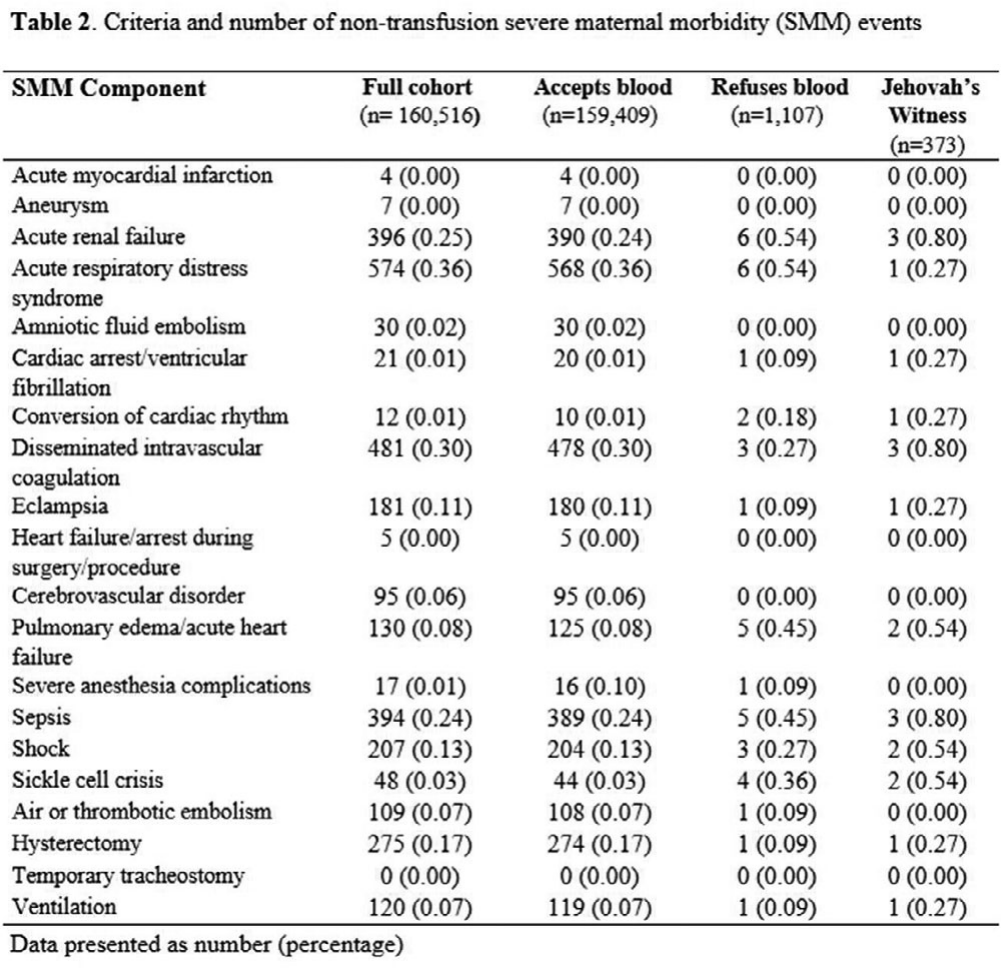

**10:30 AM – 12:00 PM**

## 229 | Evaluation of Severe Maternal Morbidity in Pregnancies Complicated by Maternal Seizure Disorder

Janki Patel^1^; Stephanie Schreiber‐Gonzalez^2^; Rachel K. Harrison^3^; Calla Holmgren^4^

^1^Advocate, Chicago, IL; ^2^Ascension, Chicago, IL; ^3^Department of Maternal‐Fetal Medicine, Advocate Aurora Health, Chicago, IL; ^4^Advocate Medical Group, Chicago, IL

**Objective**: To identify prevalence of seizure disorders in pregnancy in a modern cohort and assess rates of severe maternal morbidity (SMM) among those with seizure disorders.

**Study Design**: Retrospective cohort study using the National Inpatient Sample (NIS) database from 2017–2021 through HCUP/AHRQ encompassing antepartum, delivery and postpartum admissions. Pregnant patients with seizure disorder were identified by ICD‐10 codes for epilepsy and were compared to pregnant patients without. We assessed prevalence of seizure disorders in pregnancy from 2017 to 2021 using weighted sampling. We compared baseline characteristics of those who experienced seizures to those who did not. Our primary outcome was a composite of SMM as per the 21 indicators defined by the CDC. Secondary outcomes were hemorrhage, cesarean delivery, maternal death, and maternal length of stay. Statistical analysis was performed using Stata version 17.0 via student's T test, Chi2 analysis, and linear and logistic regression to control for confounders.

**Results**: Over a 5‐year period, more than 16 million subjects were included of which a total of 835,443 pregnant patients had a seizure disorder. The incidence of epilepsy increased from 2017 to 2021 (0.184% to 0.210%, *p* < 0.001). Patients with a seizure disorder were more likely to be overweight, use tobacco and have diabetes mellitus and hypertension. They were more likely to experience the composite SMM, hemorrhage, cesarean delivery, and maternal death with longer length of stay than those without a seizure disorder. These findings persisted in the adjusted analysis with rates of SMM and maternal death much more likely among those with seizure disorder (aOR 2.05, 95% CI 1.92–2.18 and aOR 5.94, 95% CI 2.89–12.24, respectively). Hemorrhage, cesarean, and increased length of stay also remained associated.

**Conclusion**: Seizure disorder in pregnancy increased in frequency from 2017 to 2021 and is associated with identifiable risk factors including obesity, tobacco use, DM and hypertension. These patients are more likely to experience SMM and death than pregnant patients without a seizure disorder.

**10:30 AM – 12:00 PM**

## 230 | Latency Antibiotic Use and Outcomes in Previable Prelabor Rupture of Membranes

Jasmin S. Abdeldayem^1^; Emily Fahl^2^; Julie Gutierrez^3^; Sean C. Blackwell^1^; Baha M. Sibai^1^; Irene A. Stafford^1^

^1^Division of Maternal‐Fetal Medicine, Department of Obstetrics, Gynecology and Reproductive Sciences, McGovern Medical School at UTHealth Houston, Houston, TX; ^2^Department of Obstetrics, Gynecology and Reproductive Sciences, McGovern Medical School at UTHealth Houston, Houston, TX; ^3^Houston Methodist, Houston, TX

**Objective**: There is limited evidence supporting the use of latency antibiotics <22 weeks gestation age (WGA). Our study aimed to evaluate whether latency antibiotic administration <22 WGA in patients with previable prelabor rupture of membranes (pPROM) is associated with a difference in maternal outcomes and prolonged latency.

**Study Design**: After Institutional approval, we conducted a retrospective cohort study of pregnant individuals presenting with pPROM <22 WGA at three tertiary care hospitals in Texas between 01/2018‐03/2023. ICD‐10 codes were used for chart reviews. Exclusion criteria included presentation in preterm labor, fetal demise, higher‐order multiples (≥3), elective termination, or missing delivery data. Antibiotic administration, including type and duration, was abstracted from the medical record. The primary outcome was a composite of maternal morbidity, including sepsis, ICU admission and blood transfusion.The secondary outcome was latency to delivery. Baseline characteristics were compared using *t*‐tests or Wilcoxon rank‐sum for continuous, and chi‐square or Fisher's exact tests for categorical variables. Adjusted relative risks (aRR) with 95% confidence intervals (CI) were estimated using Poisson regression.

**Results**: During the study period, 221 individuals met inclusion criteria, 76 (34%) received latency antibiotics. Baseline characteristics were largely similar between groups, though patients receiving antibiotics had later GA at rupture (*p* = 0.02). There were no significant differences in the primary composite maternal outcome between the antibiotic and no‐antibiotic groups (24% vs 26%; aRR 0.93, 95% CI 0.56–1.52). Antibiotic use was associated with longer latency (  12 vs 5, *p* < 0.001), higher GA at delivery (median 147 vs. 131 days, *p*< 0.001), and greater live neonatal discharge (9.9% vs 1.4%, *p* = 0.007; aRR 5.55, 95% CI 1.30–23.71).

**Conclusion**: In pregnancies complicated by pPROM, latency antibiotics administration <22 WGA was not associated with a reduction in composite maternal morbidity. However, it was linked to prolonged latency, higher GA at delivery, and increased neonatal survival.

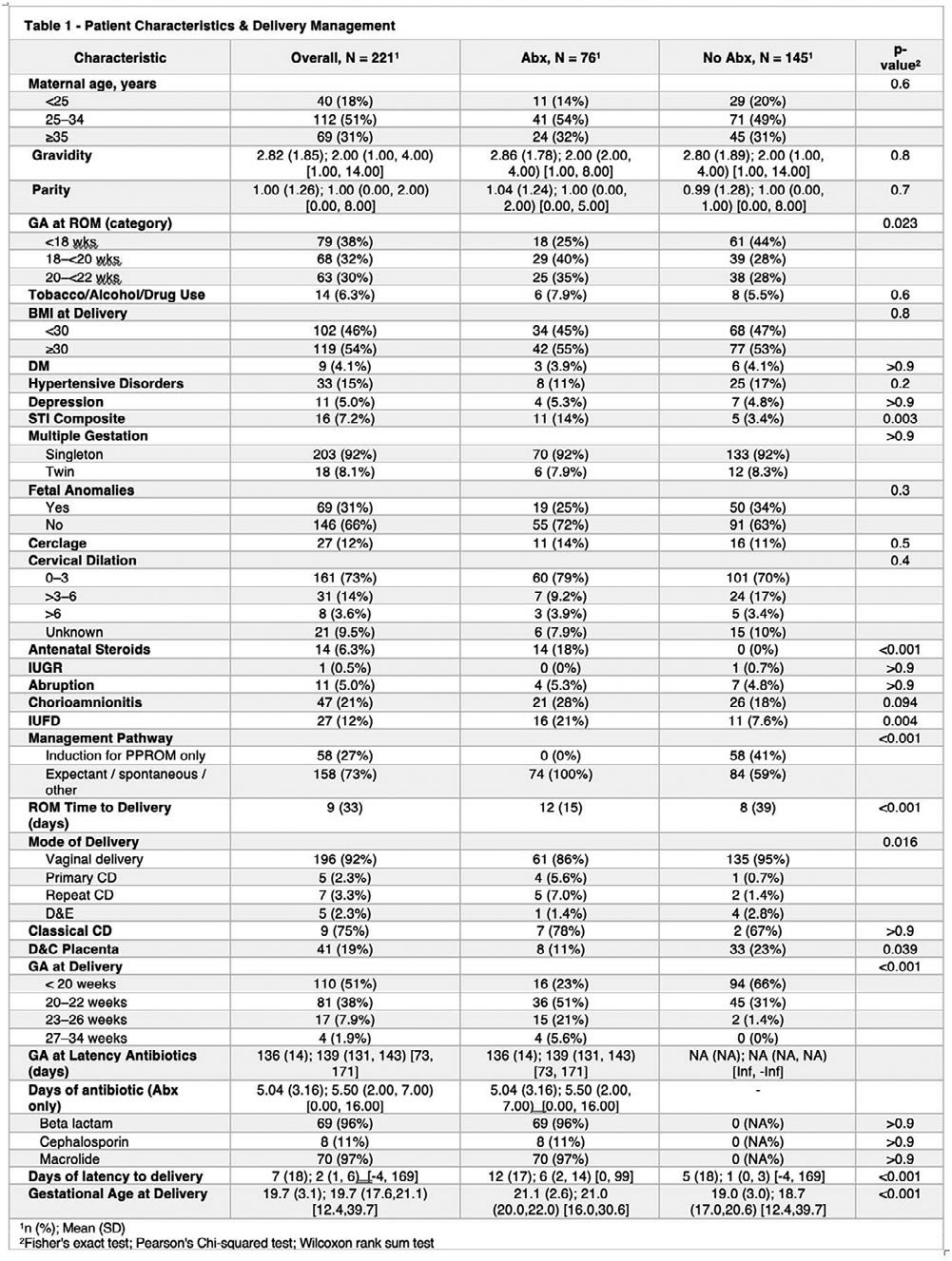

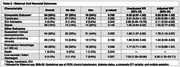

**10:30 AM – 12:00 PM**

## 231 | Third Trimester Ultrasound Fetal Abdominal Circumference Measurement and Risk of Neonatal Shoulder Dystocia at Birth

Jasmine Hitt; Maria Som; Cassandra Seifert; Katherine Goetzinger; May Hsieh Blanchard

University of Maryland Medical Center, Baltimore, MD

**Objective**: Fetal macrosomia is a well‐established risk factor for neonatal shoulder dystocia (ShD). There is data that EFW >4500 g and abdominal circumference (AC)/head circumference (HC) ratio >90% is associated with shoulder dystocia, but risk of shoulder dystocia by AC parameter alone is scarce. We evaluated whether the risk of shoulder dystocia is associated with third trimester ultrasound measurement of AC >99%.

**Study Design**: We investigated 208 mothers who delivered infants with ShD and 720 randomly selected controls who delivered vaginally from a cohort of women who received antenatal care at an urban, tertiary medical center from 2012–2023 for association of AC >99% and shoulder dystocia at delivery. Our secondary aim was to evaluate if other common risk factors of ShD are generalizable to our diverse, urban patient population. We used adjusted logistic regression to estimate the risk of ShD in those with AC >99%.

**Results**: Pregnant women who had infants with ShD were more likely to be younger (26.8 vs 28.4; *p* < 0.001), nulliparous (1.1 vs 1.5; *p* = 0.001), demonstrate greater weight gain in pregnancy (29.5 vs 24.7; *p* = 0.02), report a history of prior shoulder dystocia (6.2% vs 2.2%; *p* = 0.001), have diabetes (23.9% vs 14.6%; *p* = 0.003) and undergo operative vaginal delivery (3.8% vs 1.7%; *p* = 0.04). Patients with third trimester fetal AC >99% did not have a difference in their risk of shoulder dystocia compared to those with third trimester fetal AC <99% (32.6% vs 28.6%; OR 1.2; *p* = 0.29). Patients with EFW >4500 g on third trimester ultrasound were more likely to have shoulder dystocia compared to those with EFW <4500 g (2.6% vs 0.7%; OR 3.9; *p* = 0.02).

**Conclusion**: Fetal ultrasound measurements of AC >99% in the third trimester are not associated with increased risk of shoulder dystocia. Other identified risk factors of shoulder dystocia such as maternal age, parity, weight gain in pregnancy, history of shoulder dystocia, diabetic status, EFW >4500 g, and operative vaginal delivery are similar in this patient population.

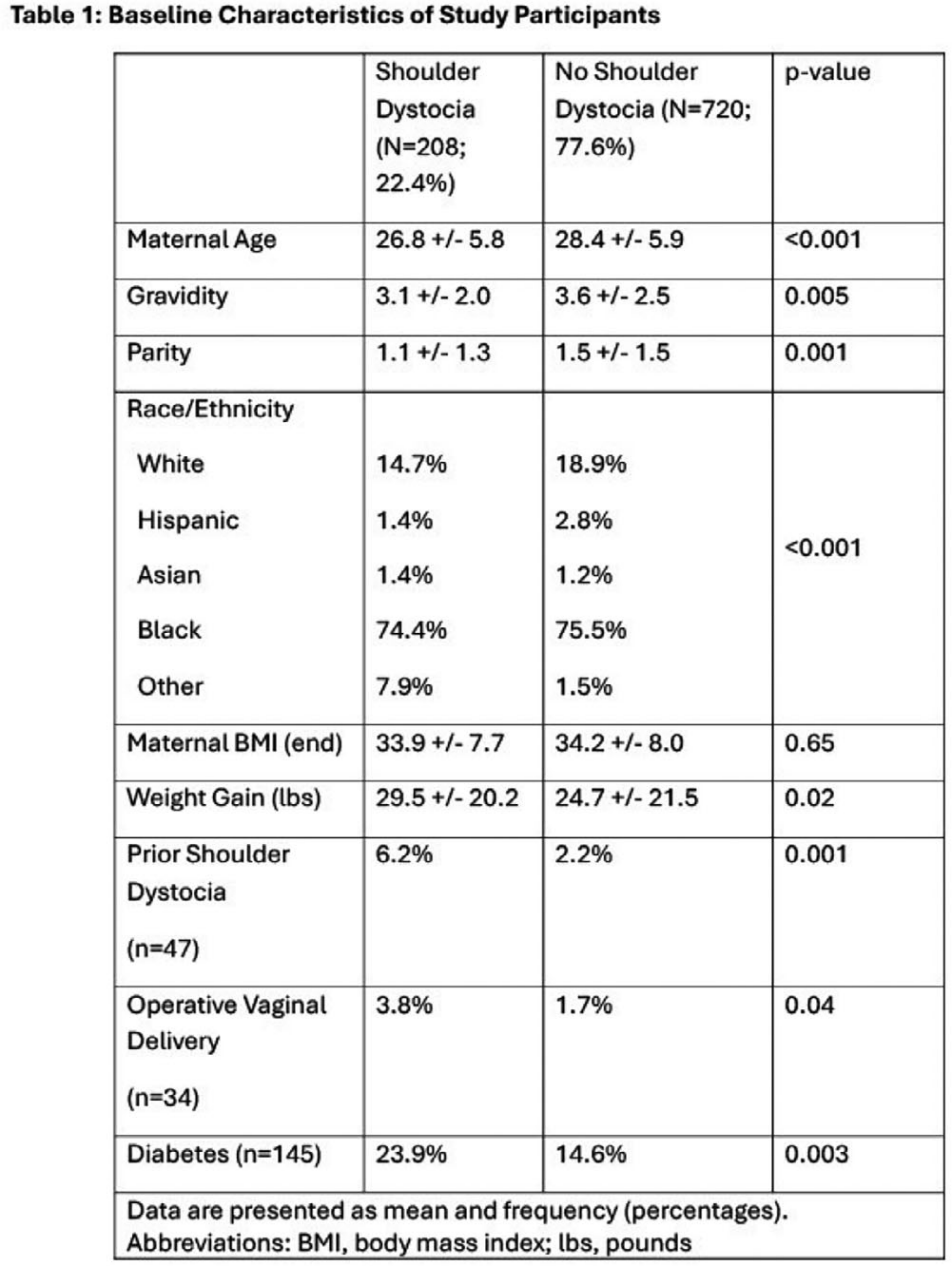

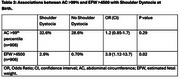

**10:30 AM – 12:00 PM**

## 232 | Fetal Electroencephalography as a Predictor of Distress in Labor: A Retrospective Analysis of 140 Cases

Jasmine W. Jiang^1^; Juan Urrutia‐Gandolfo^2^; Molly Lucas^3^; Jose Cortes‐Briones^2^; Emily Lee^4^

^1^Yale School of Medicine, Yale School of Medicine, CT; ^2^Department of Psychiatry, Yale School of Medicine, New Haven, CT; ^3^Wavelet Medical Inc., Brooklyn, NY; ^4^Department of Obstetrics, Gynecology, and Reproductive Sciences, Yale School of Medicine, New Haven, CT

**Objective**: Fetal heart rate (FHR) monitoring is the standard for assessing fetal status during labor, but it is often equivocal and has low positive predictive value (PPV) for fetal distress. Despite its widespread use, rates of neurologic injury from birth have not declined, while cesarean deliveries have increased. Fetal electroencephalography (EEG) has been proposed as a complementary tool to better assess neurologic risk.

**Study Design**: We analyzed data from 140 laboring individuals (31 weeks to term) with fetal EEG recorded via a fetal scalp electrode (FSE). Data included FHR tracings, fetal scalp pH, cord gases, and 1‐minute Apgar scores. EEG was recorded using a suction‐mounted silver chloride electrode. Fetal distress was defined as pH ≤ 7.20 and/or Apgar <7. Abnormal EEG was defined as reduced amplitude/frequency, absent sleep cycling, or near‐isoelectric tracing. We calculated sensitivity, specificity, PPV, and negative predictive value (NPV) of EEG and compared it to Category II/III FHR tracings.

**Results**: Among 33 cases of fetal distress, 26 had abnormal EEGs and 7 had normal EEGs. Among 107 without distress, 103 had normal EEGs and 4 had abnormal EEGs (Table 1). EEG showed sensitivity of 79%, specificity of 96%, PPV of 87%, and NPV of 94%. These values were higher than those observed with FHR monitoring and consistent with published data (Table 2).

**Conclusion**: Fetal EEG during labor correlates with indicators of distress, including low pH and Apgar scores. Compared to FHR monitoring, EEG demonstrated superior specificity, PPV, and NPV. EEG may serve as a valuable adjunct to FHR monitoring to improve fetal neurologic assessment and perinatal outcomes.

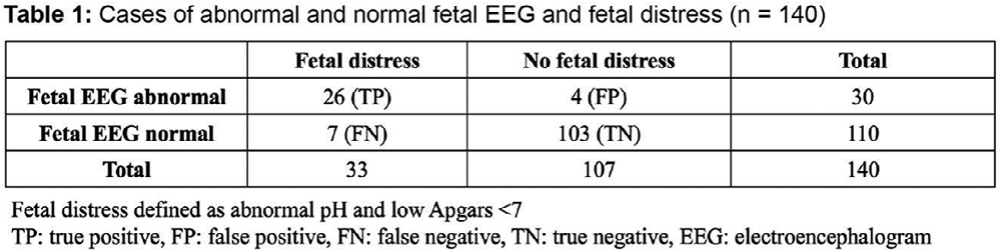

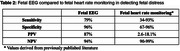

**10:30 AM – 12:00 PM**

## 233 | Food Insecurity, Systemic and Placental Inflammation, and Adverse Perinatal Outcomes

Jasmine T. Rios^1^; Chuhan Wu^2^; Lauren Keenan‐Devlin^2^; Linda M. Ernst^2^; Alexa A. Freedman^3^; Gregory E. Miller^4^; Ann E Borders^5^

^1^University of Chicago Pritzker School of Medicine, Chicago, IL; ^2^Endeavor Health, Evanston, IL; ^3^Northwestern University, Chicago, IL; ^4^Northwestern University, Evanston, IL; ^5^Endeavor Health, Evanston Hospital, Evanston, IL

**Objective**: Food insecurity during pregnancy has been consistently linked to adverse birth outcomes, potentially due to increased inflammation. This study compared systemic and placental inflammation among pregnant patients experiencing food insecurity and those who were food secure to investigate inflammation patterns associated with food insecurity in pregnancy.

**Study Design**: 605 participants were enrolled in the prospective Stress, Pregnancy, and Health (SPAH) study 2018–2022. Food insecurity was assessed during the 3^rd^ trimester using the validated 2‐Item Hunger Vital Sign screener. Serum cytokines were measured via 2^nd^ trimester blood draw and placentas were collected at delivery, with biopsies reviewed for inflammatory and vascular lesions. Multivariable analyses were adjusted for maternal age, gestational age at sample collection, and race/ethnicity.

**Results**: Among 524 participants, 57 (10.9%) screened positive for food insecurity. Food‐insecure participants were more likely to be enrolled in Medicaid, have lower household education, higher household unemployment, and higher BMI (all *p* < 0.05). Participants with food insecurity were more likely to experience adverse neonatal outcomes (aOR = 3.00, *p* = 0.004), including preterm birth (aOR = 2.36, *p* = 0.044) and neonatal intensive care admission (aOR = 3.48, *p* = 0.006). However, no significant associations were found between food insecurity and elevated pro‐inflammatory cytokines or placental inflammatory and vascular pathology (all *p* > 0.2).

**Conclusion**: Food insecurity during pregnancy is a significant risk factor for adverse perinatal outcomes. However, this risk does not appear to operate through systemic inflammation or placental pathology. Addressing food insecurity in pregnancy is an opportunity to reduce perinatal risk. Other biological or psychosocial mechanisms may drive the relationship between food insecurity and poor birth outcomes, warranting further investigation.

**10:30 AM – 12:00 PM**

## 234 | **Postpartum Hypertension and Readmission Among Patients With Preeclampsia With Severe Features**

Jaye Boissiere^1^; Marie‐Louise Meng^1^; Madison Calvert^2^; Sally Kuehn^1^; Hannaneh Mirmozaffari^2^; Maya Patel^2^; Ashley Ruhashya^2^; Brooke Schroeder^1^; Matt Fuller^1^; Jerome J. Federspiel^1^; Johanna Quist‐Nelson^2^; Kim Boggess^3^

^1^Duke University School of Medicine, Durham, NC; ^2^University of North Carolina, Chapel Hill, Chapel Hill, NC; ^3^University of North Carolina at Chapel Hill, Chapel Hill, NC

**Objective**: Patients with preeclampsia with severe features (PEC‐SF) experience more morbidity postpartum (PP) than those without severe features. Morbidity risk by specific severe feature is unknown. We evaluated the relationship between specific PEC severe feature and number of severe features, and the risk of need for postpartum hypertension care (readmission or outpatient medication management).

**Study Design**: This retrospective cohort study included adult patients diagnosed with PEC‐SF in two tertiary care centers between 12/2015 and 12/2017. PEC and SF diagnoses were based on ACOG guidelines. The primary outcome was readmission for inpatient HTN management within 0–7, 7–90, or 90–365 days PP. The secondary outcome was need for outpatient HTN therapy adjustment within the same timeframes. Descriptive statistics and multivariate logistic regression models were used to examine associations between severe feature type or number of severe features and outcomes, controlling for age, chronic HTN, diabetes, renal disease, and obesity (BMI >30).

**Results**: Of the 774 patients with PEC‐SF, 48 (6.2%), 33 (4.3%), and 14 (1.8%) were readmitted in the 0–7, 7–90, and 90–365 days PP timeframes, respectively. Severe range BP was associated with increased risk of readmission within 7 days PP (Table). No other severe criteria were associated with increased risk of readmission at any timeframe. There was no association between individual severe criteria and outpatient PP HTN medication adjustment (Table). There was no association between the number of SFs and the risk of readmission or outpatient medication adjustment (Table).

**Conclusion**: Readmission within 1 week PP was more common in patients with a diagnosis of PEC‐SF by blood pressure than by other severe features of preeclampsia. The specific criteria and number of criteria used to diagnose PEC‐SF did not influence the development of worsening HTN requiring outpatient management in the PP period. Our findings emphasize the importance of close follow‐up of patients with PEC‐SF during the first week PP.

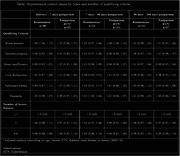

**10:30 AM – 12:00 PM**

## 235 | Association of Unplanned Postpartum Hospital Use With Patient‐Reported Outcomes

Jecca R. Steinberg^1^; Sydney L. Raucher^2^; Ying Cheung^2^; Brittney R. Williams^2^; Joseph M. Feinglass^2^; Michelle A. Kominiarek^2^; William A. Grobman^3^; Lynn M. Yee^2^

^1^UCSF, San Francisco, CA; ^2^Northwestern University Feinberg School of Medicine, Chicago, IL; ^3^Brown University, Providence, RI

**Objective**: Postpartum hospital readmission or emergency department use has significant medical, social, and financial consequences, yet little is known about how unplanned hospital use may influence patient‐reported outcomes (PROs). We aimed to evaluate whether postpartum hospital use is associated with PROs in the first year postpartum.

**Study Design**: This was a secondary analysis of data from a randomized trial of postpartum patient navigation which included patients who were ≥16 years, had public insurance, and were English‐ or Spanish‐speaking. Postpartum hospital use was defined as emergency department visits or hospital readmissions after delivery discharge within 12 weeks postpartum. All postpartum hospital use occurred prior to study visits. Participants completed study visits that included PROs assessing perceived health status at 4–12 weeks and 11–13 months postpartum. Differences in PRO scores by postpartum hospital use were evaluated using bivariable tests.

**Results**: Of 405 randomized participants, 18% (*n* = 73) had postpartum hospital use, including 15 readmissions. Participants with and without postpartum hospital use were similar with respect to demographic characteristics (Table 1). At 4–12 weeks postpartum, postpartum hospital use was associated with lower perceived physical health, lower self‐efficacy, and greater perceived stress; these findings did not persist at 11–13 months (Table 2). At 11–13 months, participants with postpartum hospital use reported lower perceived informational support (i.e. availability of helpful information). No differences were seen at either time in perceived mental health, patient activation, knowledge of postpartum risks, postpartum care satisfaction, breastfeeding self‐efficacy, postpartum preparedness, or depressive symptoms.

**Conclusion**: Unplanned postpartum hospital use was associated with poorer perceived health, confidence in managing health, and stress at 4–12 weeks postpartum. Low‐income patients who experience unplanned postpartum hospital use may benefit from increased support and resources to optimize postpartum health in the early postpartum period.

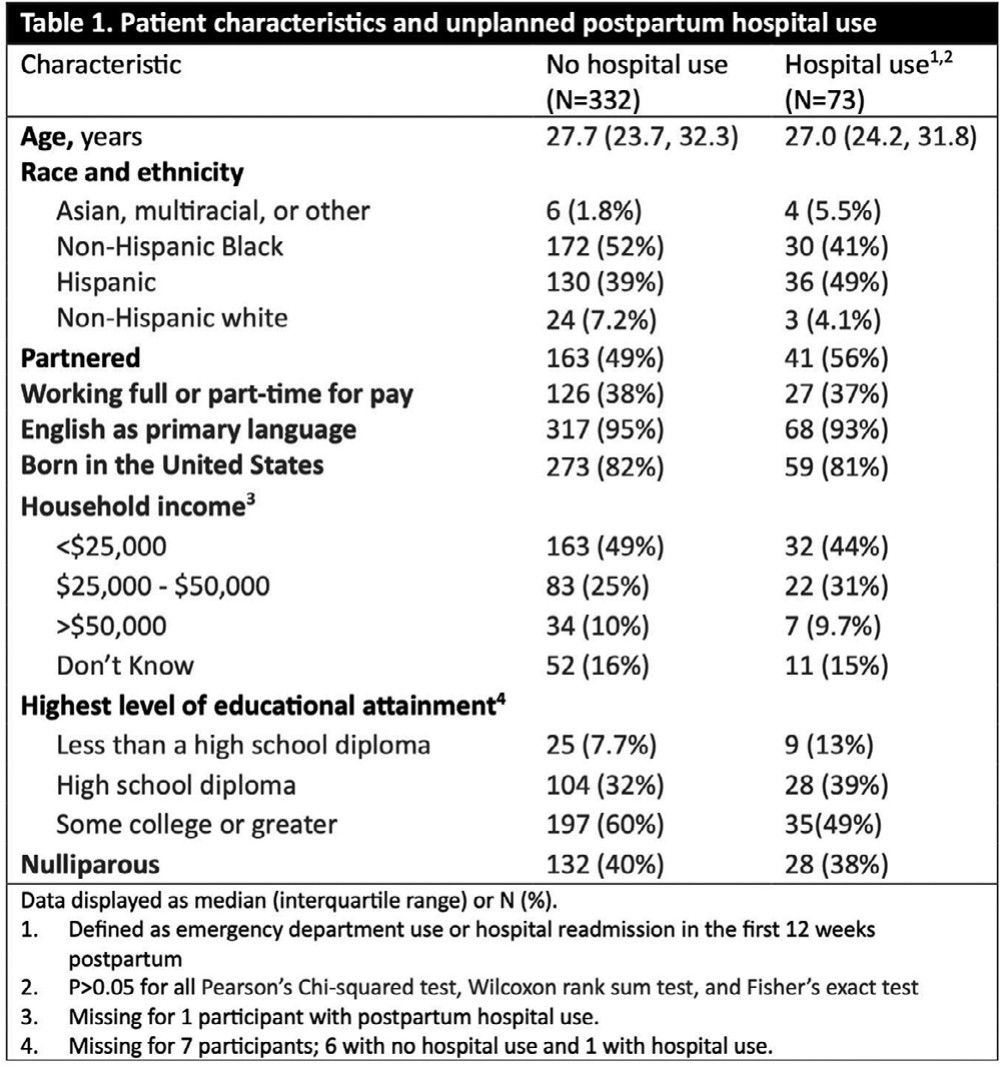

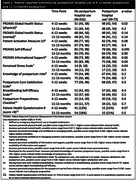

**10:30 AM – 12:00 PM**

## 236 | Maternal and Perinatal Course in Antenatally Diagnosed Vein of Galen Malformation (VOGM)

Jennifer M. Watchmaker^1^; Brandon Philbrick^1^; Alex Devarajan^1^; Alejandro Berenstein^1^; Miwa Geiger^2^; Angela T. Bianco^3^; Tomoyoshi Shigematsu^1^; Johanna Fifi^1^

^1^Icahn School of Medicine at Mount Sinai, Icahn School of Medicine at Mount Sinai, NY; ^2^Mount Sinai Medical Center, New york, NY; ^3^Icahn School of Medicine at Mount Sinai, New York, NY

**Objective**: VOGM is increasingly diagnosed in utero, allowing for prenatal counseling and delivery planning. However, data on the timing and nature of perinatal care is limited. This study aims to describe the perinatal care timeline and outcomes for pregnancies affected by VOGM, with the goal of informing multidisciplinary care pathways.

**Study Design**: We conducted a retrospective review of all pregnancies with antenatal VOGM diagnosis born at our referral center between 2014 and 2025. Data collected included maternal demographics, gestational age (GA) at diagnosis, GA at fetal MRI and neurointerventional consultation, antenatal digoxin use, GA at delivery, mode of delivery, and neonatal outcomes.

**Results**: Of 33 reviewed neonatal VOGM cases, 25 pregnancies with an antenatal diagnosis of VOGM were included in the analysis. The median maternal age was 32.9 years, and VOGM was detected at a median GA of 32.1 weeks. Fetal MRI was performed in 22 cases, and consultation with neurointerventional surgery was performed at a median GA of 33.3 weeks. Antenatal digoxin was initiated in 22.7% of cases for management of high‐output cardiac failure detected on fetal echocardiogram. Cesarean delivery was the predominant mode of delivery (83.3%), with median GA at delivery of 37.6 weeks and median birthweight of 3.3 kg. Among neonates, 64% underwent endovascular embolization during the birth hospitalization (median age at first embolization was 1.4 days). There were 3 neonatal mortalities, one in which embolization was not offered due to extensive brain injury on postnatal MRI, and two mortalities following embolization. The median length of birth admission was 16.5 days for survivors (7.0 days for patients not requiring neonatal embolization, and 28 days for patients who underwent neonatal embolization).

**Conclusion**: In cases requiring neonatal intervention, embolization was frequently performed shortly after birth. Given the complexity of VOGM, timely coordination with specialized centers with neurointerventional and neonatal expertise following antenatal diagnosis may be critical to optimizing outcomes.

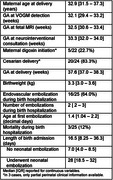

**10:30 AM – 12:00 PM**

## 237 | Predicting Fetal Acidemia Using Convolutional Neural Networks Trained on Intrapartum Electronic Fetal Monitoring Data

Jess Breda; Darshan Joshi; Benison Pang; Matthew Wells; Melissa S. Wong

Cedars‐Sinai Health Sciences University, Los Angeles, CA

**Objective**: Continuous electronic fetal monitoring (EFM) is widely used to assess fetal well‐being during labor. However, its interpretation is subjective, inconsistent, and poorly correlated with neonatal outcome. We sought to develop and evaluate a convolutional neural network (CNN) to predict fetal acidemia using intrapartum fetal heart rate data.

**Study Design**: We conducted a retrospective study of singleton, >37 0/7 weeks, liveborn deliveries with EFM data at a tertiary care hospital from 2011–2023 for which pH or APGAR data were available. Only births with high‐quality FHR signal in the 30 minutes prior to time of birth (defined as non‐zero signal in 70% of samples) were included for training which restricted our dataset to vaginal births (Figure 1). We applied a convolutional neural network to predict a composite marker of neonatal distress, defined as pH <7.2 or APGAR at 1 minute <7. We evaluated performance on 50 sample held‐out test‐set using the area under the receiver operating characteristic curve (AUC). Both training and test sets were balanced with positive and negative samples. We assessed model performance on various input types and architectures of relevance to the field.

**Results**: 62,299 deliveries met inclusion criteria. Of these, 6379 had pH test obtained and the remainder had APGAR available. Of these, 5423 met criteria for our composite marker of neonatal distress (of which 3391 had pH <7.2). The model achieved an AUC of 0.83 + 0.01 in predicting acidemia on the holdout dataset. We found a single‐channel model with only fetal heart rate performed better than a two‐channel model that also included uterine activity and that the model performed best with filter‐sizes of 2‐minutes in length (Figure 2).

**Conclusion**: A deep learning model can accurately predict fetal acidemia using short segments of FHR data in the final minutes of labor. We have further demonstrated operational proof of concept of EFM streaming, integrated this into our existing data pipeline. Future work will focus on prospective validation, integration into clinical decision making, and evaluation of impact on outcomes.

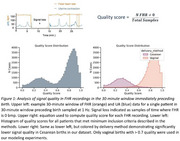

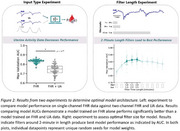

**10:30 AM – 12:00 PM**

## 238 | Alabama Womb2Heart Pilot Randomized Trial for Postpartum Individuals With Adverse Pregnancy Outcomes

Jesse E. Rattan^1^; Lynda G. Ugwu^2^; Kayla Torres‐Benson^1^; Vivek V. Shukla^3^; Colm P. Travers^4^; Rosylen R. Quinney^5^; Molly B. Richardson^6^; Aduke A. A. Toluhi^7^; Lynetta West^8^; Trinita Ashford^9^; Victoria R. Jauk^2^; Nancy Saxon^1^; Nickel Cofield^10^; Alysia N. Campbell^11^; Donna E. Dunn^2^; Eleni Z. Tsigas^12^; Justin M. Leach^2^; Henna Budhwani^13^; Janet M. Turan^11^; Waldemar A. Carlo^3^; Alan TN Tita^2^; Rachel G. Sinkey^14^

^1^Department of Obstetrics and Gynecology, University of Alabama at Birmingham, Birmingham, AL; ^2^Center for Research in Women's Health, University of Alabama at Birmingham, Birmingham, AL; ^3^Department of Pediatrics, University of Alabama at Birmingham, Birmingham, AL; ^4^Department of Pediatrics, University of Alabama at Birmingham Heersink School of Medicine, Birmingham, AL; ^5^University of Alabama at Birmingham, Department of Obstetrics and Gynecology, University of Alabama at Birmingham, AL; ^6^University of Alabama at Birmingham, University of Alabama at Birmingham, AL; ^7^Department of Obstetrics and Gynecology, University of Alabama at Birmingham, Department of Obstetrics and Gynecology, University of Alabama at Birmingham, AL; ^8^ConnectionHealth, Birmingham, AL; ^9^ConnectionHealth, ConnectionHealth, AL; ^10^Department of Obstetrics and Gynecology, University of Alabama at Birmingham, Center for Research in Women's Health, University of Alabama at Birmingham, AL; ^11^University of Alabama at Birmingham, Birmingham, AL; ^12^Preeclampsia Foundation, Preeclampsia Foundation, FL; ^13^Florida State University College of Nursing, Tallahassee, FL; ^14^Center for Women's Reproductive Health Department of Obstetrics and Gynecology University of Alabama at Birmingham, Birmingham, AL

**Objective**: Cardiovascular disease (CVD) is the #1 killer of women. Adverse pregnancy outcomes (APOs) are associated with future CVD, yet few interventions have targeted the postpartum (PP) period. We evaluated the acceptability of a community health worker (CHW) intervention that aimed to improve PP blood pressure (BP) control and reduce CVD risk among Black women who are disproportionately affected by APOs.

**Study Design**: We conducted a single‐center, unblinded, parallel‐group randomized controlled trial. Eligible participants self‐identified as Black and experienced an APO (Table 1). Participants were randomized postpartum to usual care (UC) vs UC + CHW support for 12 weeks. Both groups received a Cuff Kit^®^ home BP monitor and education on APO‐related CVD risks and postpartum hypertension. The CHW intervention included weekly contact to provide psychosocial support, coach BP monitoring, assist with connecting to a primary care provider (PCP), and link participants to community resources. The primary outcome was satisfaction, defined by satisfaction scores (1–10 scale) and validated healthcare acceptability scale scores. Secondary outcomes included BP monitoring frequency, mean BP, and PCP appointment scheduling.

**Results**: Of 64 approached patients, 95% (61) consented and were randomized (UC *n* = 31; UC + CHW *n* = 30) between September 2024 and April 2025. Median age was 29 years, 61% were parous, 53% had less than ≤ high school diploma, and 64% were publicly insured. Among UC+ CHW participants, 84% (21/25) were extremely satisfied, and 100% (19/19) rated CHW support as highly acceptable (5/5). Over 90% of participants in both groups monitored BP at home. Mean BP obtained clinically at the PP visit was 134/87 mmHg in the UC group vs 127/84 mmHg in the CHW group. PCP appointment scheduling was reported by 65% in the UC group (11/17) and 80% (12/15) in the UC + CHW group (Table 2).

**Conclusion**: A CHW‐led intervention for PP individuals at elevated CVD risk was feasible and highly acceptable among Black participants. Findings support and our next steps include a larger clinical trial powered to evaluate clinical outcomes.

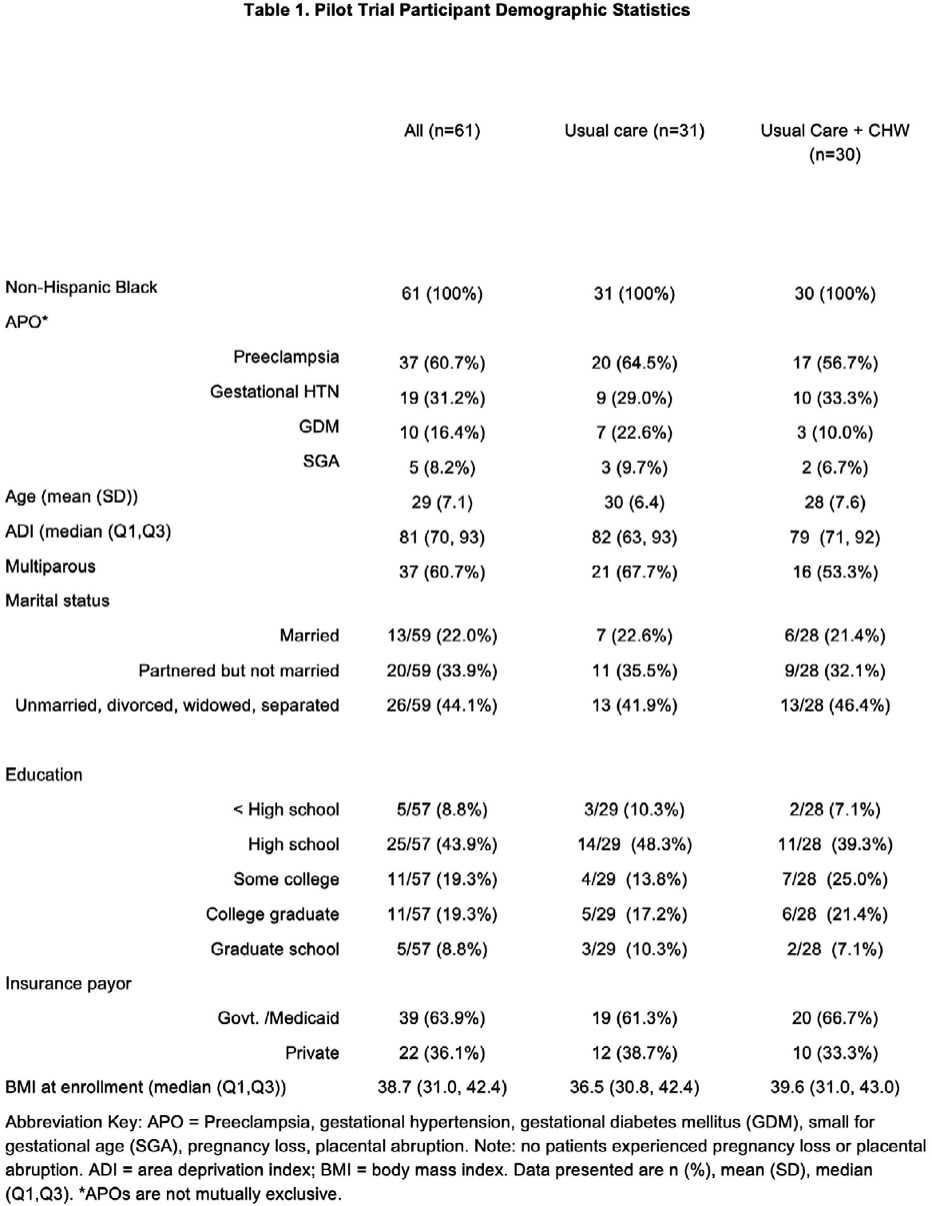

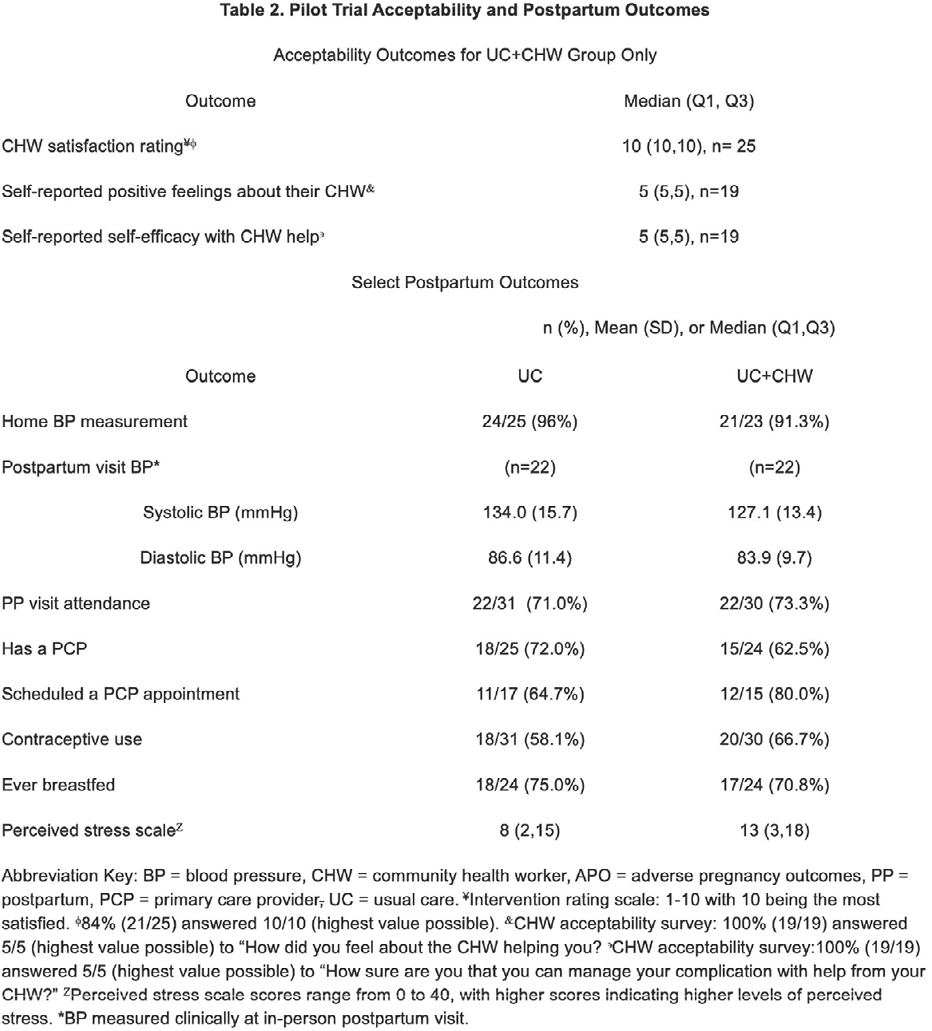

**10:30 AM – 12:00 PM**

## 239 | Effect of Glucose Monitoring Frequency on Outcomes in Gestational Diabetes: A Systematic Review and Meta‐Analysis

Jessica Montgomery; Miriam J. Alvarez; Saniya Gayake; Kobina Ghartey; Anne Mardy; Lorie M. Harper

Department of Women's Health, Dell Medical School at the University of Texas at Austin, Austin, TX

**Objective**: Frequent glucose monitoring for gestational diabetes (GDM) is a source of patient dissatisfaction. Reduced testing frequency may offer a more acceptable yet effective approach to glycemic control in pregnancy. This study aimed to evaluate outcomes with less frequent glucose monitoring compared to standard four‐times‐daily testing in patients with GDM.

**Study Design**: Electronic databases were searched Jan 1990 ‐ Apr 2025 for randomized controlled trials (RCT) or cohort studies comparing four‐times‐daily to less frequent testing in GDM. Exclusion criteria were other interventions, non‐English, unpublished studies, and other study designs. The primary outcome was birth weight. Secondary outcomes were macrosomia, cesarean delivery, neonatal hypoglycemia, and need for medication. Pooled risk ratios (RRs) or mean differences (MDs) were calculated using Mantel‐Haenszel random‐effects models as appropriate; heterogeneity was assessed with I^2^ and τ^2^.

**Results**: Of 1346 titles and abstracts reviewed, two RCTs and two retrospective cohort studies were included. Study quality was moderate. Less frequent testing was not associated with increased birth weight (MD = 131.1 g; 95% CI: −8.7–270.9, *p* = 0.07) but was associated with a significantly higher risk of macrosomia (RR = 1.4; 95% CI: 1.1–1.7, *p* = 0.01). Cesarean delivery and neonatal hypoglycemia were similar between groups (RR = 1.1; 95% CI: 0.9–1.3, *p* = 0.56; and RR = 0.96; 95% CI:0.7–1.4, *p* = 0.84). Pharmacologic treatment was more common with less frequent testing (RR = 1.4; 95% CI: 1.1–1.7, *p* = 0.01). Heterogeneity was high: RCTs compared daily to every‐other‐day testing, while cohort studies compared daily to weekly testing. Stratified analysis showed that every‐other‐day testing resembled daily testing, whereas weekly testing worsened outcomes.

**Conclusion**: Reduced glucose testing in GDM is associated with increased risks of macrosomia and pharmacologic treatment. These findings support continued use of standard testing protocols, though further studies should focus on every‐other‐day testing as a possible strategy to reduce patient burden.

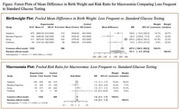

**10:30 AM – 12:00 PM**

## 240 | Evaluating the Real‐World Impact of the CHAP Trial on Blood Pressure Control and Perinatal Outcomes

Jessica L. Rizzuto^1^; Emily Stone^2^; Jinyuan Liu^1^; Amir Javid^1^; Marc Robinson^1^; Zainab Shonibare^3^; Kathryn J. Lindley^4^; Soha S. Patel^1^; Sarah O. Osmundson^5^

^1^Vanderbilt University Medical Center, Nashville, TN; ^2^University of Alabama, Tuscaloosa, Tuscaloosa, AL; ^3^Vanderbilt University School of Medicine, Nashville, TN; ^4^Department of Medicine, Division of Cardiology, Vanderbilt University Medical Center, Nashville, TN; ^5^Vanderbilt University Medical Center, Vanderbilt University Medical Center, TN

**Objective**: The Chronic Hypertension and Pregnancy (CHAP) trial shifted paradigms around blood pressure (BP) control in pregnant women with chronic hypertension (CHTN). We assessed whether national and local recommendations to target BPs in pregnancy to <140/90 mmHg resulted in lower blood pressure and improved perinatal outcomes using both cohort and time series analyses.

**Study Design**: We conducted a retrospective cohort study of all pregnant women with CHTN who initiated prenatal care with at least 2 prenatal visits prior to 20 weeks’ gestation and delivered at a single medical center between 1/1/2018 and 7/1/2024. The primary outcome was visit systolic blood pressure (SBP). Individual patient records were reviewed to assess composite outcomes from the CHAP trial. The pre‐Chap period spanned visits from 1/1/2018 through the CHAP trial publication (4/2/2022) and the post‐CHAP period spanned 6‐weeks after rollout of local guidelines for CHTN management (7/1/2022) through the end of the study period (7/1/2024). We conducted an interrupted time series analysis using mixed effects models to analyze the effects of guideline changes.

**Results**: Of 12,979 pregnancies meeting inclusion criteria, 556 (4.3%) had CHTN (346 deliveries pre‐CHAP, 210 post‐CHAP). On average, SBP decreased 0.020 mmHg (95% CI −0.027, −0.013) weekly during the study period. Guideline changes were associated with a 2.25 mmHg lower SBP (95% CI −4.19, −0.32) but no additional SBP decrease was noted after guidelines. Guideline changes were not associated with a decrease in the CHAP trial outcomes (Table 2). However, during the overall time period, each 1‐unit decrease in SBP was associated with a 1.2% decreased risk for the CHAP trial outcomes (aRR −0.012, 95% CI −0.021, −0.002, *p* = 0.014).

**Conclusion**: In a real‐world situation, average visit SBP decreased over a 6‐year period and was impacted by national and local implementations of guidelines from the CHAP trial. The overall decrease in SBP was associated with a reduction in the composite outcome reported in the CHAP trial.

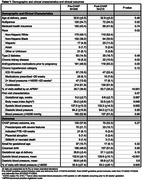

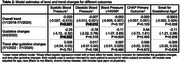

**10:30 AM – 12:00 PM**

## 241 | Influence of Area Deprivation on Patient‐Reported Community Needs: A Prospective Analysis of Underserved Urban Region

Jessica D. Sisco; Kristie R. Wilburn‐Wren; Donald D. McIntire; Robert B. Martin; Elaine L. Duryea; Catherine Y. Spong; David B. Nelson

University of Texas Southwestern Medical Center, Dallas, TX

**Objective**: Because the majority of modifiable contributors that affect maternal health are driven by nonmedical factors, the purpose of this study was to assess individual, patient‐reported community needs among postpartum women in underserved areas of an urban community.

**Study Design**: This was a prospective analysis of individual, patient‐reported community needs among postpartum women enrolled in the extending Maternal Care After Pregnancy (eMCAP) program. This program provides postpartum care through mobile and telehealth services for up to 12 months for residents in a high‐risk underserved region. Upon enrollment, patient‐reported community needs were collected by a dedicated team using a standardized instrument modeled after Healthy People 2030. Area Deprivation Indices (ADI) were assigned according to patients’ home residence and dichotomized according to decile of ADI. Patient‐reported community needs were examined for association with ADI using the chi‐square test. Demographics, pregnancy characteristics, and perinatal outcomes were examined through univariate analysis followed by logistic regression including interaction terms to test the association of ADI and selected patient responses with *p* < 0.05 considered significant.

**Results**: Between 1 Oct 2020–30 Sept 2024 4419 patients were enrolled in eMCAP. Because the eMCAP program targets an underserved region, ADI 70 was identified as the dichotomized cut‐point (<70 = lower area deprivation, >70 = greater area deprivation). In reporting what community improvement would most improve their quality of life, patients within the ADI >70 were more likely to report community safety (*p* < 0.001) and less likely to report housing and public transportation (*p* < 0.001). These findings persisted with logistic regression for factors associated with ADI (Table 1) wherein community safety was the most important factor for patients living in areas of greatest deprivation (Figure 1, *p* < 0.001).

**Conclusion**: Among postpartum women living in underserved areas, community safety was the most important patient‐reported feature for those living in areas with greatest deprivation.

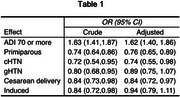

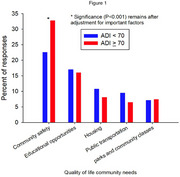

**10:30 AM – 12:00 PM**

## 242 | Prediction of Preterm Birth and Its Determinants Using Machine Learning Based on Korean Health Insurance

Elisa Gi Soo Um^1^; Hyun Sun Ko^2^; Seon‐Kwon Jang^3^; Min‐Ho Lee^3^

^1^Department of Obstetrics and Gynecology, Seoul St. Mary's Hospital, College of Medicine, The Catholic University of Korea, Seoul, Korea, Seoul, Seoul‐t'ukpyolsi; ^2^Department of Obstetrics and Gynecology, Seoul St. Mary's Hospital, College of Medicine, The Catholic University of Korea, Seoul, Seoul‐t'ukpyolsi; ^3^Department of Life Science, Dongguk University, Seoul, Seoul‐t'ukpyolsi

**Objective**: Preterm birth (PTB), defined as delivery between 20 + 0 and 36 + 6 weeks of gestation, is a major cause of neonatal morbidity and mortality worldwide. In South Korea, the PTB rate has increased from 3.8% in 2000 to 9.2% in 2021 despite declining fertility rates. This study aimed to develop a machine learning (ML)‐based model for PTB prediction using national health and screening data and to identify key determinants of PTB risk, at the national level.

**Study Design**: We conducted a retrospective cohort study using linked data from the Korean National Health Insurance Service, national health screenings, and infant records. Eligible participants were women with deliveries (2017–2020) who underwent a health screening within 1.5 years prior to delivery and had matched infant data. After merging seven datasets, 41 predictive features were retained following statistical filtering. To address class imbalance, fixed‐size stratified splits were used: training (*n* = 120,000; 20,000 PTB), validation (*n* = 60,000; 10,000 PTB), and test (*n* = 566,344; 10,686 PTB). ML models (Random Forest, XGBoost, LightGBM) were trained and evaluated by AUC and accuracy.

**Results**: Among 705,658 women with deliveries, 40,656 (5.8%) resulted in PTB. All three models demonstrated high performance, with an AUC of 0.72 and an accuracy of 98.1%. Feature importance analysis identified fetal number and maternal weight as the top predictors across all models, followed by socioeconomic factors such as occupation and insurance status. Among the pre‐pregnancy medication histories, use of sleeping pill and antianxiety medication were identified as important features.

**Conclusion**: ML models trained on large‐scale health data showed effective performance in PTB prediction. These models provide interpretable risk stratification tools that may support early detection and enable targeted perinatal interventions to reduce PTB rates in South Korea. Additionally, efforts to lower the rate of multiple pregnancies—one of the major contributors to PTB—should also be considered as part of a broader prevention strategy.

**10:30 AM – 12:00 PM**

## 243 | **Increased Opioid Use in Patients With NSAID Contraindications After Cesarean Hysterectomy**

Jocelyn Spizman^1^; Megan Gauger^1^; Jennifer Cate^2^; Aimee Rolston^2^; Jourdan E. Triebwasser^3^; David E. Arnolds^4^; Shitanshu Uppal^2^; Molly J. Stout^3^; AnneMarie E. Opipari^3^

^1^University of Michigan Medical School, Ann Arbor, MI; ^2^Michigan Medicine, Department of Obstetrics and Gynecology, Ann Arbor, MI; ^3^University of Michigan, Ann Arbor, MI; ^4^Michigan Medicine, Department of Anesthesia, Ann Arbor, MI

**Objective**: To investigate whether patients undergoing cesarean hysterectomies with contraindications (CI) to nonsteroidal anti‐inflammatory drugs (NSAIDs) utilize more opioids via patient‐controlled analgesia (PCA) during postoperative admission.

**Study Design**: A retrospective cohort study of all patients who underwent cesarean hysterectomy at a single academic center between 2014 and 2025. Patient characteristics and postoperative PCA administration data were collected through electronic health records. Primary exposure was presence of one or more CI to NSAIDs including ketorolac and ibuprofen; secondary exposure was use of postoperative neuraxial anesthesia (NA). Statistical analysis was completed with unpaired t‐test and chi square tests. Primary outcome was mean PCA postoperative morphine equivalents (POME) administered prior to discharge. Secondary outcomes include length of postoperative admission and count of patients with PCA use.

**Results**: 215 patients were included in the study. 12 (5.6%) patients had CI to NSAIDs, 5 (42%) of which received postoperative NA. 203 (94%) patients did not have a CI, of which 46 (23%) received NA. Patients with CI utilized significantly more POME and had longer postoperative admissions (Table 1; *p* < 0.0001 for both), despite similar PCA use (*p* = 0.1192). When comparing patients with CI who used NA, and patients without CI who did not use NA, total mean POME and hospital length of stay were significantly higher in patients with CI (Table 2, *p* < 0.0001 for both).

**Conclusion**: Increased opioid exposure is associated with a higher risk of prolonged opioid use, dependence, and opioid use disorder. Our results suggest increased exposure to opioids during longer postoperative admissions in patients with CI to NSAIDs, which was not normalized with NA use. To minimize exposure to opioids, multimodality pain control strategies are warranted in patients with NSAID CIs including interrogation of the etiology of the CI, allergy testing when indicated, and consideration for adjunctive nonpharmacologic modalities.

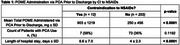

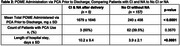

**10:30 AM – 12:00 PM**

## 244 | Impact of Social Determinants of Health on Expectant Management of Preeclampsia With Severe Features

Joe Haydamous^1^; Yossi Bart^2^; Zakaria Doughan^3^; Ahmed Zaki Moustafa^4^; Baha M. Sibai^2^

^1^Department of Obstetrics, Gynecology and Reproductive Sciences, McGovern Medical School at UTHealth Houston, Department of Obstetrics and Gynecology, McGovern Medical School at UT Health, Houston, TX; ^2^Division of Maternal‐Fetal Medicine, Department of Obstetrics, Gynecology and Reproductive Sciences, McGovern Medical School at UTHealth Houston, Houston, TX; ^3^Department of Obstetrics and Gynecology, McGovern Medical School at UT Health, Houston, Houston, TX; ^4^Division of Maternal‐Fetal Medicine, Department of Obstetrics, Gynecology and Reproductive Sciences, McGovern Medical School at UTHealth Houston, University of Texas ‐ Houston, TX

**Objective**: To evaluate the association between individual‐ and neighborhood‐level social determinants of health (SDOH) and latency (time from diagnosis to delivery) in patients with preeclampsia with severe features managed expectantly.

**Study Design**: A retrospective cohort study of patients diagnosed with preeclampsia with severe features between 23w0d and 33w3d from 2016–2025 at a single quaternary care center. Patients were excluded if they delivered within one day of admission or had contraindications for expectant management on presentation (eclampsia, HELLP syndrome, stroke, acute kidney injury, pulmonary edema, intrauterine fetal demise, or placental abruption). Individual‐level variables included maternal age, race/ethnicity, insurance type, marital status, and substance use. Neighborhood‐level variables were derived from the CDC Social Vulnerability Index (SVI) and dichotomized at the 90th percentile. The primary outcome was latency ≥3 days from diagnosis to delivery.

**Results**: Among 405 eligible patients, 269 (66.4%) had a latency ≥3 days, while 136 (33.6%) delivered within 3 days. Demographic and behavioral characteristics were comparable between groups. Both groups demonstrated high neighborhood vulnerability, particularly in the minority status/language domain (median SVI = 0.85). In unadjusted analyses, no individual‐ or neighborhood‐level factors differed significantly between latency groups. Multivariable regression adjusting for demographic, behavioral, and neighborhood characteristics similarly showed no statistically significant associations.

**Conclusion**: For patients with expectantly managed preeclampsia with severe features before 34 weeks, SDOH were not associated with latency from diagnosis to delivery. The consistency of this finding across both individual‐ and neighborhood‐level factors suggests that in the setting of severe disease, delivery timing is primarily driven by clinical acuity and obstetric criteria.

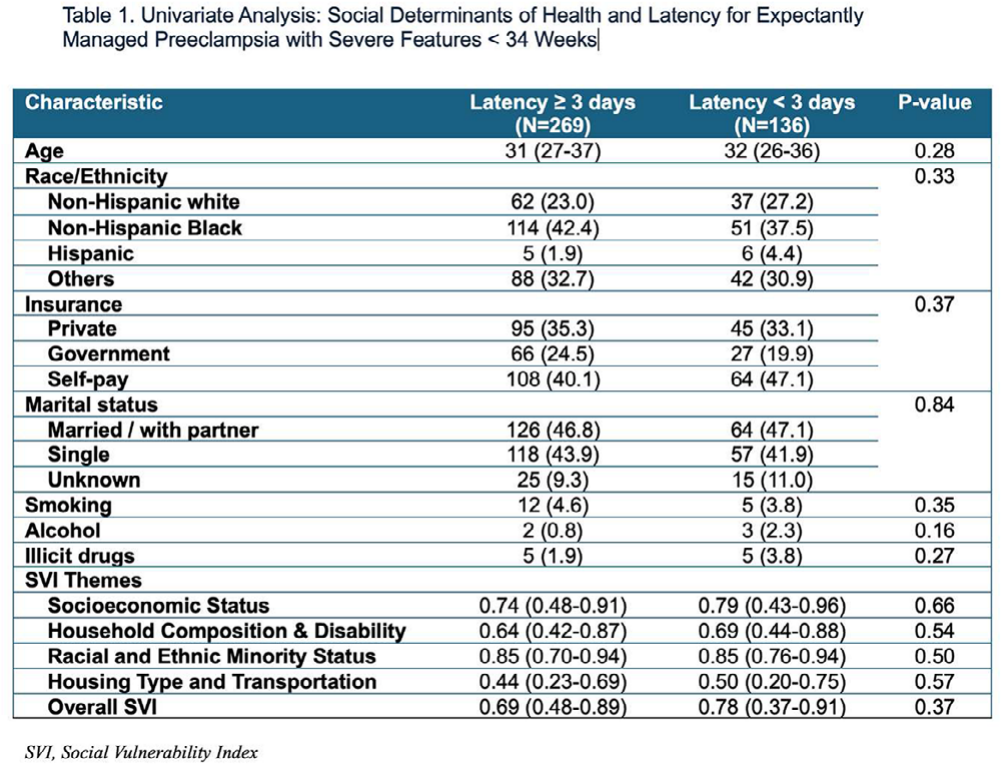

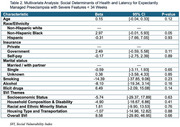

**10:30 AM – 12:00 PM**

## 245 | The Relationship Between Agentry During Antepartum Hospitalization and Postpartum Depression or Anxiety Symptoms

John Soehl^1^; Agata Atayde^2^; Melissa A. Clark^3^; Emily S. Miller^4^; Nina Ayala^4^

^1^University of Wisconsin‐Madison, Madison, WI; ^2^Warren Alpert Medical School of Brown University, Providence, RI; ^3^Brown University School of Public Health, Providence, RI; ^4^Women & Infants Hospital of Rhode Island / Warren Alpert Medical School of Brown University, Providence, RI

**Objective**: Psychological well‐being during pregnancy predicts postpartum mental health, yet few tools exist to identify modifiable factors in high‐risk populations. The Antepartum Agentry Scale (AAS), adapted from the Labor Agentry Scale, measures perceived control among individuals hospitalized during pregnancy. We aimed to evaluate whether AAS scores during antepartum hospitalization are associated with symptoms of depression and anxiety at six weeks postpartum.

**Study Design**: In this prospective cohort study, the AAS, Patient Health Questionnaire (PHQ‐9), and Generalized Anxiety Disorder (GAD‐7) were administered to individuals hospitalized due to pregnancy complications. Follow‐up PHQ‐9 and GAD‐7 surveys were emailed six weeks postpartum. Participants were categorized by AAS scores as “Low” (<1 standard deviation [SD] below mean) “Average” (±1 SD) or “High” (>1 SD above mean). Cuzick's test for trend evaluated associations between AAS category and postpartum mental health symptoms. Mediation analyses examined the extent to which the relationship between antepartum and postpartum mental health symptoms was partially explained by the AAS.

**Results**: Of the 60 enrolled participants, 43 (72%) completed the postpartum survey. Characteristics of the analyzable sample are shown in Table 1. Higher AAS categories were associated with significantly lower PHQ‐9 and GAD‐7 scores (Table 2). Mediation analysis indicated that AAS score mediated 26% (95% CI–23% to 76%) of the relationship between antepartum and postpartum PHQ‐9 scores, and 21% (95% CI–33% to 75%) of the relationship between antepartum and postpartum GAD‐7 scores, although these did not reach statistical significance.

**Conclusion**: Higher perceived control during antenatal hospitalization was associated with lower symptoms of postpartum depression and anxiety. Although mediation results were nonsignificant, these findings suggest that antepartum agentry is a potential target for intervention to improve postpartum mental health outcomes in high‐risk populations.

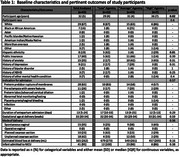

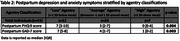

**10:30 AM – 12:00 PM**

## 246 | The Utility of Hemoglobin Electrophoresis in the Setting of Expanded Carrier Screening

Joselle O'Brien^1^; Eliana Wachstock^2^; Emily Schlussel Markovic^1^; Itamar Futterman^3^; Robyn Weiss^1^; Shoshana Haberman^1^; Rodney A. McLaren, Jr^1^; Tirtza Spiegel Strauss^1^

^1^Maimonides Medical Center, Brooklyn, NY; ^2^Yeshiva University, New York, NY; ^3^Maimonides Medical Center, Maimonides Health, NY

**Objective**: ACOG recommends universal hemoglobinopathy screening for prenatal patients. Historically, this was performed via hemoglobin electrophoresis. However, expanded carrier screening (ECS) is increasingly used in the prenatal setting, and includes screening for hemoglobinopathies. We compared the concordance in detection rates between hemoglobin electrophoresis and ECS, with regards to identifying hemoglobinopathies.

**Study Design**: This was an IRB approved retrospective cohort study of 444 patients who underwent ECS at our academic hospital clinic. Hemoglobin electrophoresis results were available for 362 patients. We compared the detection rates between the hemoglobin electrophoresis and ECS, and their concordance, using Chi‐square test.

**Results**: Patient demographics are in Table 1. Among the 362 patients, 19/362 (5.2%) had abnormal electrophoresis findings. Of these, 18/19 (94.7%) had concordant hemoglobinopathy‐related findings on ECS; the one discordant case involved a clinically benign variant (Hb Korle Bu). ECS detected hemoglobinopathy‐related findings in 43/362 patients (11.9%, *p* >0.01). Of these 43 patients, 18 (41.9%) had concordant abnormal electrophoresis findings; the other 25 (58.1%) were all silent alpha thalassemia carriers.

**Conclusion**: ECS demonstrates high concordance with hemoglobin electrophoresis to detect clinically relevant hemoglobinopathies, but can also detect silent carriers of alpha thalassemia, which may be a missed opportunity for partner screening in setting of hemoglobin electrophoresis only, especially in populations with high prevalence of cis alpha thalassemia carriers. In a prenatal care setting where ECS is routinely used, additional utility of the hemoglobin electrophoresis may be limited.

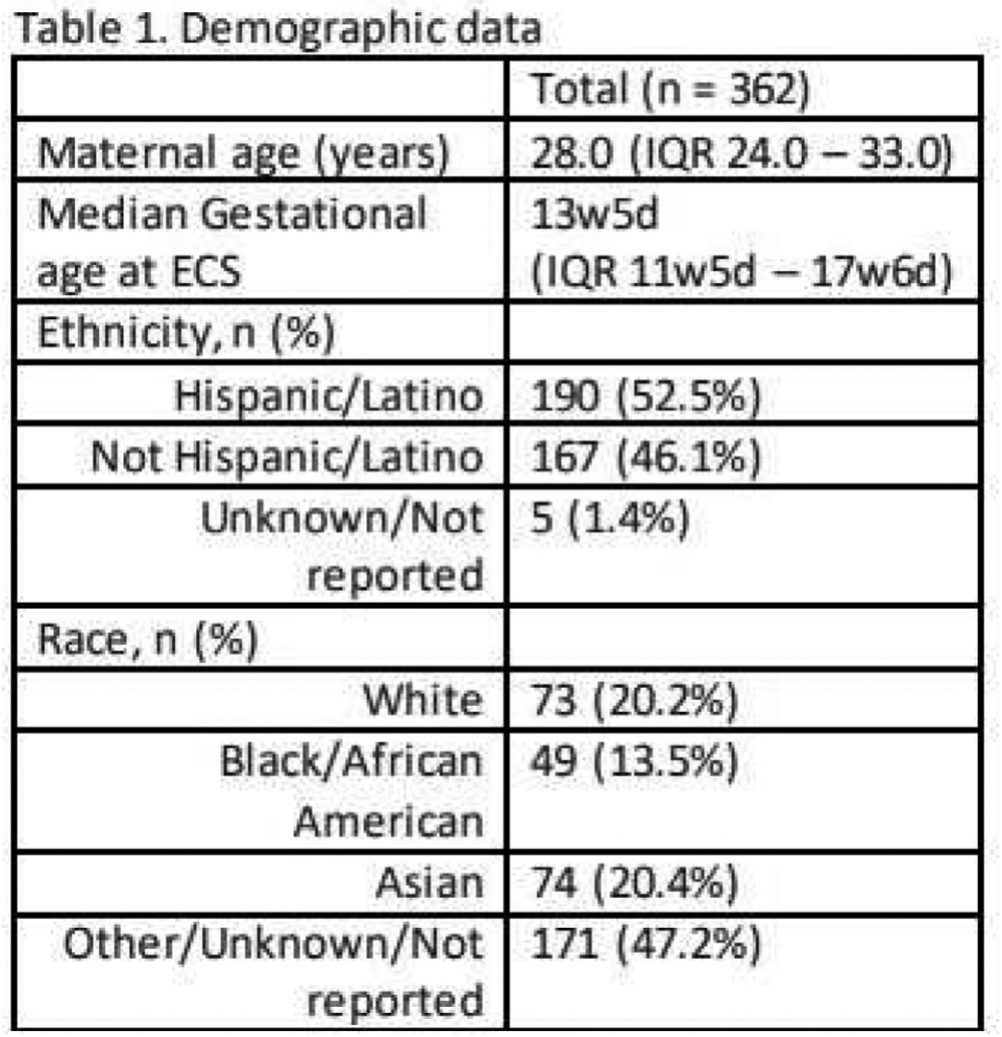

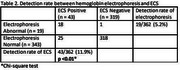

**10:30 AM – 12:00 PM**

## 247 | Decreased Growth Velocity is Associated With Adverse Outcomes in Normally Grown and Growth Restricted Fetuses

Joseph Levy^1^; Avery Day^2^; Mason Sifford^2^; Chloe Ramirez Biermann^2^; Zachary Alholm^2^; Anthony O. Odibo^3^

^1^University of Missouri‐Kansas City, University of Missouri‐Kansas City, MO; ^2^University of Missouri‐Kansas City, Kansas City, MO; ^3^University of Missouri Kansas City, Kansas City, MO

**Objective**: The current Society for Maternal Fetal Medicine (SMFM) diagnosis for fetal growth restriction is based on the estimated fetal weight (EFW) or the abdominal circumference, whereas the International Society of Ultrasound in Obstetrics and Gynecology (ISUOG) recommends evaluation of growth velocity as a possible risk factor for growth restriction. We sought to estimate the association between falling fetal growth velocity and adverse pregnancy outcomes (APO).

**Study Design**: A retrospective cohort study of all singleton gestations with at least two growth ultrasounds during their pregnancy with known pregnancy outcomes between 2019 and 2025. Growth velocity was calculated as the difference between the last and first ultrasound EFW Z‐Score, based on the Hadlock growth curve. The APO was defined as a composite outcome including at least one of the following: 5‐minute APGAR score ≤7, umbilical cord arterial pH <7.1, cesarean delivery for non‐reassuring fetal heart rate, stillbirth, and infant hospital length of stay (Above 90%ile = >4.5 days). Analysis includes descriptive and multivariant logistic regression.

**Results**: Among the 6469 included deliveries, the mean gestational age (GA) at the first and last ultrasound were 21.8 and 33.9 weeks, respectively. The APO was observed in 1190 patients (18.4%). The association between the change in EFW Z‐Score and the APO is shown in the Table. A fall in the Z‐score greater than 2.0 had the highest associated with APO (aOR 1.84 95% CI 1.21–2.78){adjusted for GA at delivery}. The final EFW, when less than 10%ile, was also associated with the APO (aOR 1.56, 95% CI 1.26–1.93), but was less predictive than a Z‐Score change greater than 2. A Z‐score greater than 2.0 was associated with all the individual components of the composite APO except for 5‐minute APGAR scores (see Table).

**Conclusion**: Although both EFW and Z‐Score are associated with APO, the fall in fetal growth velocity appears to be a better predictor of APO.

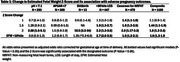

**10:30 AM – 12:00 PM**

## 248 | Association Between Resident Participation in Cesarean Delivery and Complications and Operative Times

Joseph R. Mims, Jr.; Amanda A. Allshouse; Torri D. Metz

University of Utah, Salt Lake City, UT

**Objective**: To evaluate whether resident physician involvement in cesarean delivery was associated with increased maternal or neonatal morbidity and increased operative times.

**Study Design**: Secondary analysis of the MFMU APEX study, a multicenter observational cohort of deliveries occurring from 2008–2011. Participants with a non‐anomalous, singleton term cesarean were included. The exposure was resident involvement in cesarean. The primary outcome was serious maternal operative morbidity, defined as bowel or genitourinary injury; peripartum hysterectomy for uterine trauma; reoperation for bleeding or uterine trauma; wound dehiscence requiring reoperation; or maternal death. Secondary outcomes were minor maternal operative morbidity; composite neonatal morbidity; operative time from skin incision to delivery; and total operative time from incision to completion. Propensity scores were calculated using maternal demographics, comorbidities, and labor characteristics. Risk ratios reported from ATT weighted analysis.

**Results**: Among 28,087 included deliveries, 73.8% (*n* = 20,728) had resident involvement. In propensity weighted models, resident involvement was not associated with serious maternal operative morbidity (0.6% vs 0.4%, aRR 1.24, 95% CI 0.71–2.14). However, resident involvement was associated with higher risk of minor maternal operative morbidity, longer operative times, and lower risk of neonatal morbidity (Table 2). In exploratory analyses examining the number of residents involved, results were similar in magnitude and direction to primary analysis regardless of whether comparing 2 or 3 residents to none, or 1 resident to none (data not shown).

**Conclusion**: Resident involvement in cesarean deliveries was not associated with severe maternal morbidity, such as organ injury, reoperation, or death. However, trainee involvement was associated with increased minor maternal morbidity, driven primarily by postpartum hemorrhage, and longer operative times. While resident involvement was associated with lower neonatal morbidity, differences were not clinically significant.

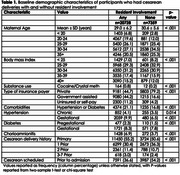

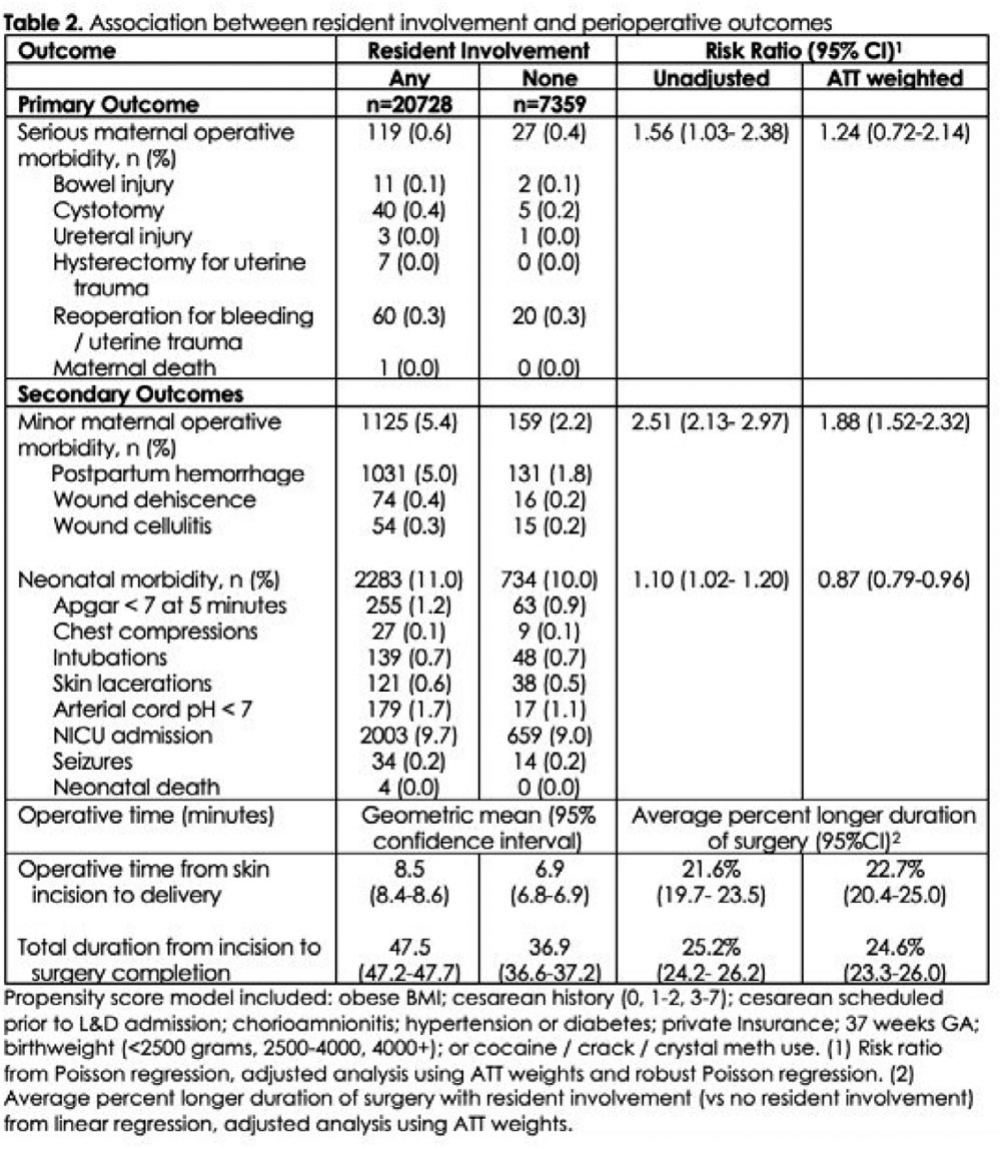

**10:30 AM – 12:00 PM**

## 249 | Evaluation of Antenatal Corticosteroid Administration in Fetal Growth Restriction

Julia Kim^1^; Amberly Lao^2^; Justin Gimoto^1^; Maria Gomez^1^; Annie Rozenblyum^1^; Soo Young Kim^1^; Hye J. Heo^1^; Wendy Kinzler^1^; Martin Chavez^1^; Lakha Prasannan^3^

^1^NYU Grossman Long Island School of Medicine, Mineola, NY; ^2^NYU Langone Hospital‐ Long Island, NYU Langone Hospital‐ Long Island, NY; ^3^NYU Long Island, Long Island City, NY

**Objective**: Antenatal corticosteroids (ACS) are administered for pregnancies at risk for preterm birth, such as those with fetal growth restriction (FGR). The optimal therapeutic window is often defined as latency from ACS to delivery of 2–7 days. Yet, timely administration of ACS can often be difficult to predict. The objective was to assess real‐world ACS administration practices and evaluate associated factors of timely administration of ACS when given for FGR. The primary outcome was the percentage of patients who received ACS within the therapeutic window. The secondary outcome was characteristic differences amongst the groups.

**Study Design**: This was a retrospective cohort study of singleton pregnancies who received ACS for FGR indications which included abnormal umbilical artery Dopplers, severe FGR and new onset FGR at a single institution from 2020 to 2024. Patients with chromosomal abnormalities, fetal anomalies, multiple gestations, or delivery prior to 24 weeks gestation were excluded. ANOVA test and Pearson's Chi‐squared test were used with *p* < 0.05 to compare characteristics amongst the groups.

**Results**: Of 154 patients with FGR and ACS exposure, 7.1% of patients were delivered within the therapeutic window. When incorporating 36 patients who received rescue ACS, 11.7% delivered within the window. Patients who delivered within the therapeutic window from initial ACS exposure were more likely to be multiparous, have history of FGR, given ACS at a later gestational age and for abnormal Dopplers. A subanalysis of those that received rescue ACS was not significant, though limited by sample size.

**Conclusion**: We observed low rates of timely administration of ACS for FGR. Yet characteristics such as multiparous, prior history of FGR, later gestational age, and abnormal Dopplers can assist in optimizing timing of ACS. Further studies should evaluate the impact of timing of ACS administration for FGR on neonatal outcomes.

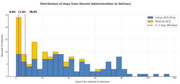

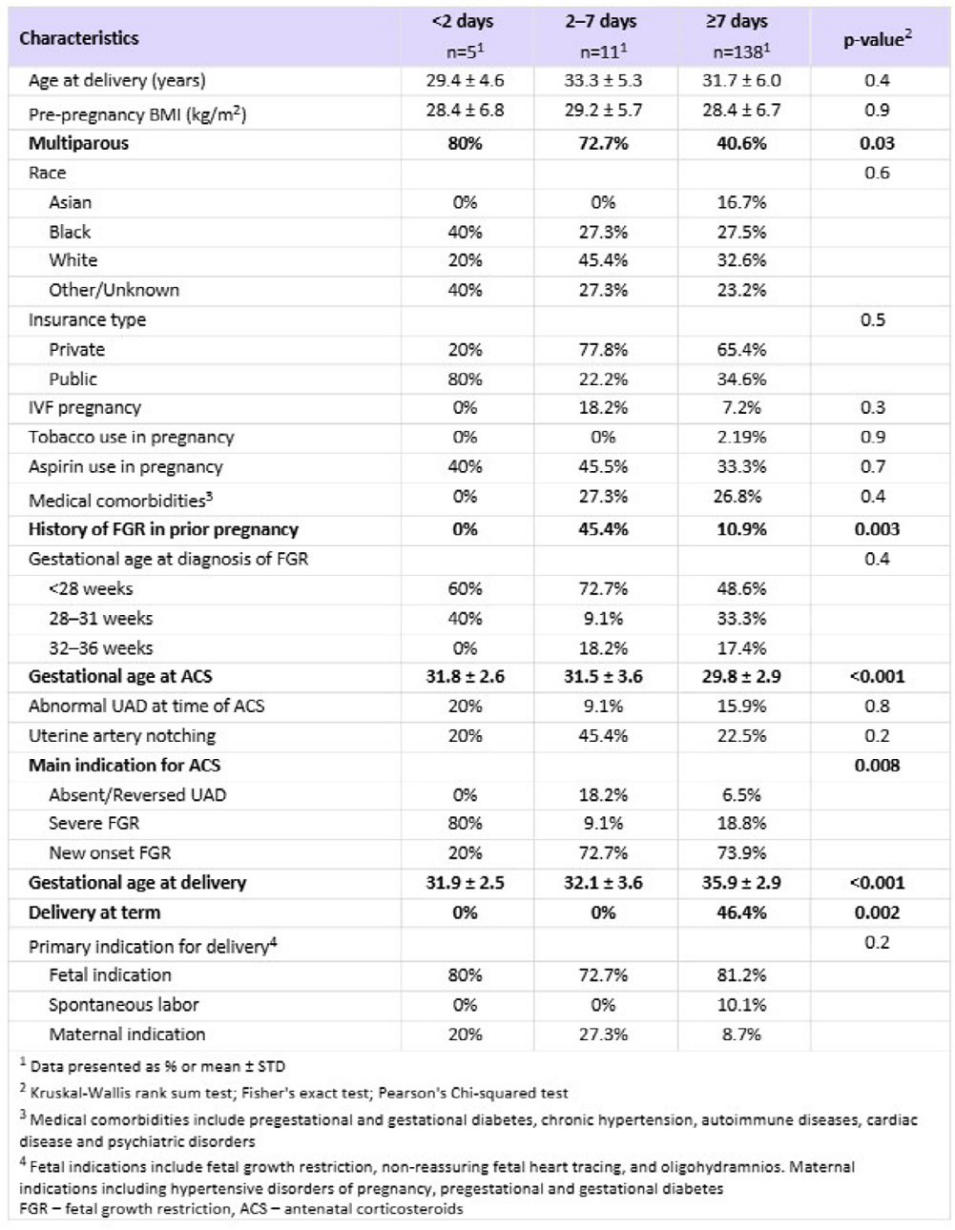

**10:30 AM – 12:00 PM**

## 250 | Hyponatremia and Associated Severity of Hypertensive Disorders of Pregnancy and Peripartum Management

Julia Rubin^1^; Anna K. Daoud^2^; Mirely Garcia^3^; Ester Sanchez^4^; Kristin M. Voegtline^5^; Moeun Son^6^

^1^Weill Cornell Medical College, Weill Cornell Medical College, NY; ^2^New York Presbyterian/ Weill Cornell Medicine, New York, NY; ^3^NewYork‐Presbyterian/Weill Cornell Medicine, NewYork‐Presbyterian/Weill Cornell Medical Center, NY; ^4^NewYork‐Presbyterian/Weill Cornell Medicine, NewYork‐Presbyterian/Weill Cornell Medicine, NY; ^5^NewYork‐Presbyterian/ Weill Cornell Medicine, New York, NY; ^6^Weill Cornell Medical College, New York, NY

**Objective**: Among patients with hypertensive disorders of pregnancy (HDP), we aimed to assess if hyponatremia is a marker of disease severity and influences fluid and medication decisions.

**Study Design**: We conducted a retrospective cohort study of pregnant patients with term singleton gestations who had a diagnosis of HDP at 2 NYC hospitals from 2020 to 2025. Patients were identified using an institutional research data repository and data were extracted via manual chart review. Patients with chronic hypertension, multiple gestations, and those missing serum sodium (Na^+^) during delivery admission (exposure) were excluded. The threshold for a low Na^+^ level was determined by calculating the lowest 10^th^ percentile (equivalent to 132 mEq/L) of values among our cohort. Those with Na^+^ ≤132 mEq/L were compared to those with Na^+^ > 132 mEq/L. The main outcomes were (1) receipt of immediate‐release (IR) antihypertensives, (2) receipt of magnesium sulfate infusion, and (3) prescribed oral antihypertensives at discharge. Other peripartum complications were also examined. Bivariate and multivariate analyses were performed.

**Results**: 572 patients met eligibility criteria. A significantly greater proportion of patients in the lowest 10th percentile of Na^+^ received IR antihypertensives (41% vs. 10%, adjusted *p* = 0.001), received magnesium sulfate infusion (56% vs. 13%, adjusted *p* = 0.001), and were discharged on oral antihypertensives (59% vs. 40%, adjusted *p* = 0.042) (Table 2). In models adjusted for preeclampsia with severe features, the lowest 10th percentile of Na^+^ was associated with 2.2 times greater odds of requiring IR antihypertensives (OR 2.2, 95% CI 1.00–4.83) and 5.1 times greater odds of receiving magnesium sulfate infusion (OR 5.1, 95% CI 1.93–13.88).

**Conclusion**: Hyponatremia is associated with severity of HDP and disease severity appears to drive the associated risks of medication use and other outcomes. Hyponatremia is independently associated with HDP‐related adverse outcomes, including need for IR antihypertensives and magnesium sulfate infusion.

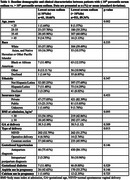

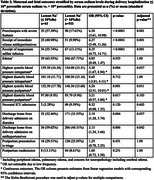

**10:30 AM – 12:00 PM**

## 251 | Implementation of an Inpatient 2‐hour Glucose Tolerance Test Improves Racial Disparities in Postpartum Screening

Julia K. Smeal^1^; Elizabeth Quigley^2^; Antoilyn Nguyen^1^; Celeste Durnwald^3^; Matthew K. Janssen^1^; Rebecca F. Hamm^1^

^1^Perelman School of Medicine, University of Pennsylvania, Philadelphia, PA; ^2^Pennsylvania Hospital, University of Pennsylvania, Philadelphia, PA; ^3^Perelman School of Medicine at the University of Pennsylvania, Philadelphia, PA

**Objective**: Growing evidence supports that implementation of a 2‐hour glucose tolerance test (2hGTT) during postpartum (PP) admission in gestational diabetes (GDM) increases adherence without decreasing efficacy. This study aims to determine if implementation of a PP inpatient 2hGTT impacts racial disparities in GTT adherence.

**Study Design**: This single‐site prospective cohort study compared 1 year before (PRE 2/2023–1/2024) to 1 year after (POST 1/2024–2/2025) implementation of an inpatient 2hGTT performed on PP day 2 for patients with GDM in place of a 6–12 week screen. Patients with GDM were included if they received prenatal care and delivered a live fetus at ≥24 weeks’ gestation in our health system. Self‐reported race was divided into Black and non‐Black. Primary outcome was rate of GTT adherence within 12 weeks of delivery. PP visit attendance was used as a balancing metric. Multivariable logistic regression assessed for variables associated with exposure (race in PRE/POST groups) and outcome (GTT adherence) as confounders. Interaction terms evaluated for impact of inpatient 2hGTT on disparities in GTT completion by race.

**Results**: 380 patients were included (PRE = 183; POST = 197). In the PRE group, there was a statistically significant disparity in rate of 2hGTT adherence, with only 5.4% Black patients completing a GTT vs 23.3% of non‐Black patients (*p* = 0.02), which persisted in adjusted analyses (aRR 0.21, 95% CI[0.05‐0.93]). No such disparity was seen POST (Black 80%, non‐Black 74.5%; *p* = 0.47; aRR 1.37, 95% CI[0.58–3.21]). 2hrGTT implementation was associated with a significant reduction in disparities in GTT completion by race (interaction *p* = 0.02). There was no difference in PP attendance in either group by race (PRE aRR 2.67, 95% CI[0.87–8.08]; POST aRR 0.45 95% CI[0.14–1.41]; interaction *p* = 0.08).

**Conclusion**: There were significant racial disparities in 2hGTT adherence among GDM patients at baseline that were effectively eliminated after implementation of an inpatient PP 2hGTT. This highlights the importance of care access interventions in advancing health equity.

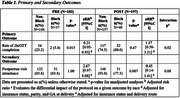

**10:30 AM – 12:00 PM**

## 252 | Implementation of sFlt‐1/PlGF Testing Among Hospitalized Patients With Hypertensive Disorders: Impact on Healthcare Utilization

Julia Whitley^1^; Dong Wang^1^; Rachel Paul^2^; Michael Dombrowski^1^; Roxane Rampersad^1^; Amanda C. Zofkie^1^; Megan L. Lawlor^1^; Jeannie C. Kelly^3^; Antonina I. Frolova^1^; Nandini Raghuraman^1^

^1^Washington University School of Medicine, St. Louis, MO; ^2^Washington University School of Medicine, Washinton University in St. Louis School of Medicine, MO; ^3^Washington University School of Medicine, Washington University School of Medicine, MO

**Objective**: In 2023, the FDA approved a sFlt‐1/PlGF test for risk assessment of preeclampsia with severe features (PS). We implemented this test on our antepartum unit in March 2024. Due to the high negative predictive value of this test, we tested the hypothesis that availability of sFlt‐1/PlGF testing reduces avoidable healthcare utilization.

**Study Design**: This is a pre‐post cohort study of all patients admitted to a single center to rule out PS. Patients on therapeutic anticoagulation, younger than 18, multifetal gestations, and without delivery information were excluded. The primary outcome was gestational latency (duration from HDP admission to delivery). Secondary outcomes included number and duration of antepartum hospitalizations as well as readmission within 6 weeks postpartum. Outcomes were compared between the pre‐implementation cohort (2/1/2023–2/29/2024) and post‐implementation cohort (3/1/2024–4/1/2025). Segmented regression‐based approaches were used to assess association between pre‐ and post‐cohorts with each outcome, adjusting for potentially unbalanced confounders. For the number of antepartum hospitalizations, a non‐parametric bootstrap resampling method was applied.

**Results**: 98 patients were admitted to rule out PS in the pre‐cohort and 73 in the post‐cohort. In the post‐cohort, 21 (28%) had sFlt‐1/PlGF testing. The incidence of PS or eclampsia in the pre‐ and post‐cohorts was 76.0% and 73.6%, respectively (*p* = 0.72). sFlt‐1/PlGF testing was more frequently ordered among patients with chronic hypertension (*p* = 0.015). The availability of sFlt‐1/PlGF testing was not associated with a difference in gestational latency, even when the analysis was restricted to those who had sFlt‐1/PlGF testing sent. There was no difference in number or duration of antepartum admissions or postpartum readmission.

**Conclusion**: The availability of sFlt‐1/PlGF testing was not associated with a difference in gestational latency or hospital utilization in this cohort. This may be related to low uptake of the test (<30%) and calls for future work studying barriers to uptake and management plans based on results.

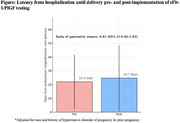

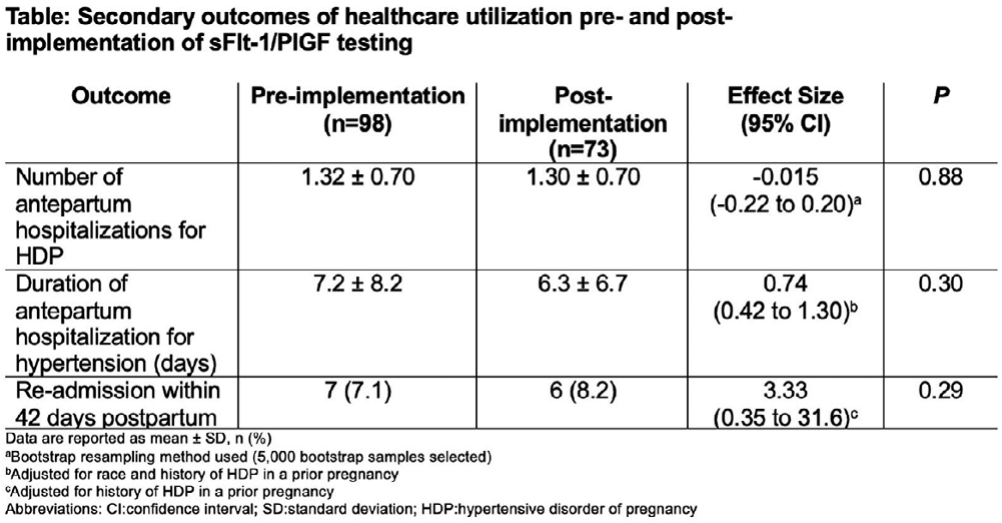

**10:30 AM – 12:00 PM**

## 253 | Association of Placenta Accreta Index With Delivery Timing in Setting of Placenta Accreta Spectrum

Kamran Hessami^1^; Sarah Tounsi^1^; Michael D. Jochum, Jr.^1^; Christina C. Reed^1^; Sanmay Sarada^2^; Yamely H. Mendez^1^; Martha Rac^1^; Jessian L. Munoz^3^; Hendrik A. Lombaard^1^; Onur Turkoglu^1^; Amir A. Shamshirsaz^4^; Michael A. Belfort^1^; Alexander M. Saucedo^1^

^1^Baylor College of Medicine, Houston, TX; ^2^Baylor College of Medicine, Baylor College of Medicine, TX; ^3^Texas Children's Hospital, Texas Children's Hospital, TX; ^4^Division of Maternal‐Fetal Medicine, Department of Obstetrics and Gynecology, Baylor College of Medicine, Houston, TX

**Objective**: We aimed to assess the Placenta accreta index (PAI) score and its correlation with gestational age (GA) at delivery and risk of unscheduled deliveries for patients with suspected PAS.

**Study Design**: Single center retrospective cohort of patients referred for suspected PAS who were evaluated and delivered at a large academic center between January 2023–July 2025. Patients were included if they had ≥ 1 prior cesarean delivery and low‐lying/ previa placentation. Sonographic images obtained in the third trimester were reviewed by 2 investigators. PAS was diagnosed if clinical evidence of invasion was seen at time of delivery or if any placental invasion was identified histologically. The primary outcome was the association between the PAI score and gestational age at delivery using spearman test, secondarily, we also evaluated the GA at the time of delivery and risk of unscheduled deliveries based on different PAI score groups using Kruskal‐Wallis test and chi‐square test, respectively.

**Results**: Ninety patients with antenatal suspicion for PAS were identified. Seventy of them had pathologic confirmation of PAS. There was a significant negative association between PAI score and GA at delivery (Figure 1) (Spearman's ρ = −0.221, *p* = 0.036). Unscheduled deliveries were significantly more frequent in higher PAI groups (*p* = 0.015) (Table 1). There was no significant difference in frequency of different reasons for unscheduled delivery among different PAI groups (*p* = 0.595).

**Conclusion**: Higher PAI scores were associated with earlier gestational age at delivery and an increased risk of unscheduled deliveries in patients with suspected PAS. These findings suggest that the PAI can be a useful tool for anticipating potential complications surrounding timing of delivery that may lead to modified management of those at highest risk of PAS to ensure optimal outcomes.

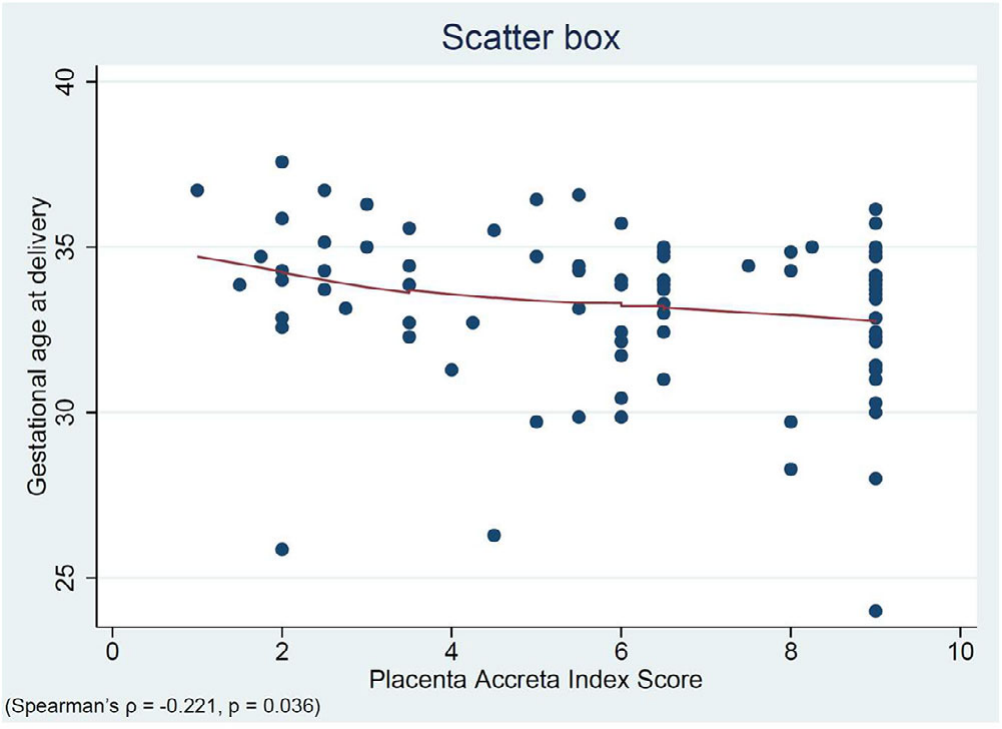

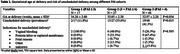

**10:30 AM – 12:00 PM**

## 254 | PDGF‐BB as a Potential Biomarker for Early Diagnosis of Postpartum Hemorrhage

Karthikeyan Thirugnanam^1^; Brock E. Polnaszek^1^; Amy Y. Pan^2^; Ramani Ramchandran^1^

^1^Medical College of Wisconsin, Milwaukee, WI; ^2^Medical College of Wisconsin, Medical College of Wisconsin, WI

**Objective**: Postpartum hemorrhage (PPH) remains a leading cause of maternal mortality globally. Current risk stratification models lack specificity and sensitivity. Platelet‐derived growth factor‐BB (PDGF‐BB), a vascular stability cytokine was hypothesized to serve as a diagnosis biomarker for PPH, which this study investigated.

**Study Design**: We conducted a retrospective analysis of 80 plasma samples collected after 21 weeks 0 days gestation but prior to delivery. Samples were selected based on diagnosis in tissue bank at delivery of PPH (*n* = 23), PPH with preeclampsia (PPH+PE; *n* = 6), and PE alone (*n* = 41). Controls (*n* = 10) were samples that had no diagnosis of PPH or PE (Table 1). PDGF‐BB protein levels were assessed by ELISA and Western blot methods. Logistic regression analysis was used to evaluate the association between PDGF‐BB and PPH.

**Results**: Among the 80 plasma samples, body mass index was statistically significantly different among PPH, PPH+PE, PE and controls, *p* = 0.047 (Table 1). ELISA assay results showed that PDGF‐BB levels were significantly lower in PPH (median and interquartile range [7.5 (6.6, 9.3) pg/mL] and PPH+PE [6.7 (4.8, 10.8) pg/mL] compared to controls [22.9 (21.7, 24.5) pg/mL], adjusted *p* < 0.0001 and *p* = 0.0015, respectively. PE alone group (22.3 pg/mL) showed no significant difference from controls. Western blot method confirmed ∼1‐fold reduction in PDGF‐BB in PPH groups. Higher PDGF‐BB levels were inversely associated with PPH risk [OR 0.62, 95% CI: 0.44–0.88; *p* = 0.0068]. ROC analysis identified a PDGF‐BB cutoff of 11.5 pg/mL with 100% sensitivity and specificity (AUC = 1.0) (Figure 1).

**Conclusion**: Low antepartum plasma PDGF‐BB levels were significantly associated with PPH and demonstrated high diagnostic performance at a defined threshold. These findings support PDGF‐BB as a promising early biomarker for PPH risk stratification. Prospective validation in larger, diverse cohorts is warranted to assess clinical applicability and potential integration into management of obstetric hemorrhage.

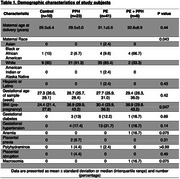

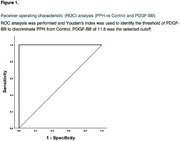

**10:30 AM – 12:00 PM**

## 255 | Assessing Aspirin Recommendation for Pre‐Eclampsia

Katherine B. Kaak^1^; Jill M. Maples^2^; Rebecca Purvis^3^; Megan L. Young^1^; Alicia M. Mastronardi^4^; Brittany Bohrer^5^; Lynlee M. Wolfe^4^; Kimberly B. Fortner^6^; Callie F. Reeder^7^

^1^University of Tennessee Medical Center, Knoxville, TN; ^2^University of Tennessee Health Science Center COM Knoxville, Knoxville, TN; ^3^University of Tennessee Health Science Center College of Medicine‐ Knoxville, Knoxville, TN; ^4^University of Tennessee Graduate School of Medicine, Knoxville, TN; ^5^University of Tennessee Knoxville, Knoxville, TN; ^6^University of Tennessee College of Medicine‐ Knoxville, Knoxville, TN; ^7^UTHSC UTGSM Knoxville, Knoxville, TN

**Objective**: USPSTF provides guidance on clinical risk factors warranting daily low‐dose aspirin (ASA) to prevent preeclampsia (PreE). However, adherence to these recommendations remains challenging. We aimed to assess provider ASA recommendations among patients with PreE, stratified by USPSTF‐defined risk level and self‐reported race.

**Study Design**: This retrospective study examined patients diagnosed with PreE delivering at a regional academic center between Jan 2022‐Mar 2023. Data were extracted from electronic records. Patients were stratified by race and by USPSTF definitions of low, moderate, or high risk. Prenatal and delivery records were reviewed by one investigator for presence of patient risk factors and documentation of provider ASA recommendation. Low risk patients were excluded because black race is a moderate risk factor. A 4‐level categorical variable was created: no ASA, moderate risk; yes ASA, moderate risk; no ASA, high risk; and yes ASA, high risk. Chi‐square tests were used to evaluate differences by race and other risk factors.

**Results**: Among 5527 patients who delivered, 440 (7.96%) patients had PreE, with 363 (6.57%) of those in the moderate or high‐risk groups. Among the 363, 56.5% (*n* = 205) were moderate risk and 43.5% (*n* = 158) were high risk. Black patients were less likely to have a documented ASA recommendation compared to non‐black patients, in both moderate (12.8% vs 19%) and high‐risk groups (25.5% vs 37.7%) (*p* = 0.021) (Fig 1). Other than self‐reported race, the only risk factor associated with ASA recommendation was pregestational diabetes, which was significantly associated with a higher likelihood of documented ASA recommendation (*p* = 0.049, data not shown).

**Conclusion**: Gaps remain in ASA prophylaxis, with notable racial disparities. Interventions to improve risk identification, documentation, and equity are needed. The association between pregestational diabetes and ASA may reflect earlier access to MFM care. Given persistent inequities and underutilization, universal ASA prophylaxis may be a population‐level strategy to improve maternal outcomes.

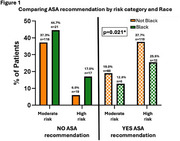

**10:30 AM – 12:00 PM**

## 256 | Impact of 2016 ACOG Postpartum Care Guidelines on Postpartum Readmissions

Katherine Pressman^1^; Jason L. Salemi^2^; Jean Paul P. Tanner^2^; Daniela R. Crousillat^3^; Mary A. Cain^1^

^1^Department of Obstetrics and Gynecology, University of South Florida, Tampa, Florida, Tampa, FL; ^2^Department of Epidemiology, College of Public Health, University of South Florida, Tampa, Florida, Tampa, FL; ^3^Division of Cardiovascular Sciences, Department of Medicine and Obstetrics and Gynecology, University of South Florida Morsani College of Medicine, Tampa, Florida, Tampa, FL

**Objective**: In 2016, ACOG published guidelines to optimize postpartum care, including earlier follow‐up and surveillance for hypertensive disorders of pregnancy (HDP). We evaluated the association of these guidelines with changes in postpartum readmissions among HDP‐affected deliveries.

**Study Design**: We conducted an interrupted time series analysis using the 2011–2021 National Readmissions Database. Deliveries to individuals aged 12–55 were included; those in November or December were excluded to ensure complete 42‐day follow‐up. Deliveries were classified as pre‐guideline (2011–2015) or post‐guideline (2016–2021) and stratified by HDP status using ICD‐9/10 codes. Postpartum readmissions were defined as any inpatient encounter within 42 days of delivery and categorized as all‐cause or HDP‐related based on diagnosis codes. Severe readmissions were defined using All Patient Refined Diagnosis Related Groups (APRDRG) severity metrics with severity of illness or risk of mortality scores of 3 (major) or 4 (extreme), based on 3M Health Information Systems Classifications.

**Results**: Across the study period, there were 4.2 million HDP deliveries and 28.9 million non‐HDP deliveries. Crude 42‐day all‐cause readmission rates were substantially higher among HDP deliveries compared to non‐HDP (35.5 vs. 11.9 per 1000 deliveries). Severe readmission rates among HDP and non‐HDP groups were 9.5 and 2.8 per 1000, respectively. Following the 2016 guidelines, all‐cause readmissions among HDP deliveries increased significantly in trend relative to non‐HDP (trend difference +0.095 per 1000 deliveries per month, *p* < 0.001). HDP‐specific readmissions increased in both level and trend (level difference +1.86 per 1000, *p* = 0.002; trend difference +0.11 per 1000 per month, *p* < 0.001). Severe postpartum readmissions among HDP deliveries declined significantly, with a relative level decrease of 2.18 per 1000 deliveries (*p* < 0.001) and a downward trend (*p* = 0.019).

**Conclusion**: The 2016 ACOG guidelines were associated with higher overall and HDP‐specific readmissions but fewer severe readmissions.

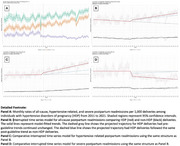

**10:30 AM – 12:00 PM**

## 257 | Impact of the Dobbs Decision on Racial and Socioeconomic Disparities in Congenital Anomaly‐Affected Births

Kavisha Khanuja^1^; Laura Uricoechea Stoker^2^; Kathryn M. Schoenauer^3^; Hadley A. Morgan^3^; Kavita Vinekar^2^; Huda B. Al‐Kouatly^4^; Rodney A. McLaren, Jr^5^

^1^Sidney Kimmel Medical College Thomas Jefferson University, Philadelphia, PA; ^2^Thomas Jefferson University, Thomas Jefferson University, PA; ^3^Sidney Kimmel Medical College at Thomas Jefferson University, Philadelphia, PA; ^4^Division of Maternal‐Fetal Medicine, Department of Obstetrics and Gynecology, Sidney Kimmel Medical College at Thomas Jefferson University, Philadelphia, PA, USA, Philadelphia, PA; ^5^Maimonides Medical Center, Brooklyn, NY

**Objective**: Since its passing in 1979, the Hyde Amendment has demonstrated to disproportionately affect women of color and low‐income individuals. The recent Dobbs decision has raised concerns of worsening these existing disparities through either outright banning abortion or implementing early gestational limits. Therefore, the objective of this study is to assess the impact of Dobbs decision on various socioeconomic and racial groups by examining the incidence of live births with congenital anomalies or chromosomal disorders.

**Study Design**: This was a population‐based cohort study of all live births complicated by congenital anomalies or chromosomal disorders using data from the U.S. Natality Vital Statistics Database from 2019–2023. Differences between specific socioeconomic and racial cohorts in the incidence of live births with congenital anomalies or chromosomal disorders before and after Dobbs were examined using univariate analysis.

**Results**: Among 46,490 total anomaly‐affected live births, 35,279 (0.34%) occurred pre‐Dobbs and 11,211 (0.33%) post‐Dobbs. Overall incidence remained stable across insurance type (private vs. WIC) and education level. However, significant racial variation emerged. The proportion of Hispanic individuals among anomaly‐affected births increased significantly post‐Dobbs (21.8% vs. 24.0%, *p* < 0.001), while representation remained stable among White and Black populations. There was a modest decline in anomaly‐affected births among those with some college education (19.3% vs. 18.1%, *p* = 0.012), but no change was noted for those with WIC coverage (31.8% both pre‐ and post‐Dobbs, *p* = 0.950).

**Conclusion**: While overall rates remained stable, the increased incidence of anomaly‐affected births among Hispanic individuals post‐Dobbs suggests emerging disparities tied to abortion restrictions. These findings underscore the need for policy and clinical strategies that address inequities in reproductive healthcare access.

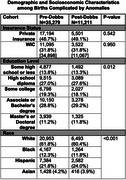

**10:30 AM – 12:00 PM**

## 258 | Postpartum Visit Attendance Among Patients at Elevated Cardiovascular Risk in a Large Urban Cohort

Rachel Nordlicht^1^; Jessica Atrio^2^; Kavita Vani^3^

^1^Albert Einstein College of Medicine, The Bronx, NY; ^2^Alice L. Walton School of Medicine, Bentonville, AR; ^3^Albert Einstein College of Medicine/Montefiore Medical Center, Bronx, NY

**Objective**: To evaluate postpartum visit (PPV) attendance of those at elevated risk for long‐term cardiovascular disease (CVD), defined by adverse pregnancy outcomes known to be associated with increased risk of CVD, in a large urban cohort.

**Study Design**: We conducted a retrospective cohort study of 13,874 patients who received prenatal care and delivered at Montefiore Medical Center between June 2018‐May 2022. Elevated CVD risk was defined as having >1 of the following: preterm delivery (<37 weeks), low birthweight (<2500 grams), intrauterine fetal demise, hypertensive disorder of pregnancy, or gestational diabetes, based on ICD‐9/10 coding. Bivariate analyses compared demographics, clinical characteristics and outcomes by CVD risk status. Logistic regression assessed the association between CVD risk and the primary outcome of PPV attendance within 12 weeks, adjusting for age, prenatal care use, race/ethnicity, parity, delivery mode, COVID‐19 period, obesity, mental health, NICU admission and insurance. Secondary outcomes included emergency room (ER) visitation and readmission within 12 weeks.

**Results**: 5854 deliveries (42.2%) met criteria for elevated CVD risk. Compared to those without CVD risk, at‐risk patients were older (34.4 +/− 6.2 vs. 33.1 +/− 5.9 years), more likely to be Black (33.3% vs. 27.9%), and had higher rates of cesarean delivery (42.4% vs. 31.1%), obesity (37.4% vs. 24.3%), NICU admission (18.2% vs. 4.7%) and mental health conditions (11.0% vs. 8.7%), all *p* < 0.01. PPV attendance was higher among those with CVD risk (61.8% vs. 49.7%), across all time intervals (0–3 weeks: 44.4% vs. 28.6%; 3–8 weeks: 27.0% vs. 23.5%; 9–12 weeks: 20.0% vs. 17.7%), all *p* < 0.01. After adjustment, CVD risk remained associated with higher odds of PPV attendance (aOR 1.30, 95% CI 1.20–1.40, *p* < 0.01), ER visits (aOR 1.13, 95% CI 1.02–1.25, *p* = 0.02) and readmission (aOR 1.2, 95% CI 1.02–1.44, *p* = 0.04).

**Conclusion**: Patients at elevated CVD risk had greater odds of postpartum care engagement, yet 40% still missed follow‐up. Early postpartum visits offer a key opportunity for targeted prevention in this high‐risk group.

**10:30 AM – 12:00 PM**

## 259 | Preterm Birth in a Heterogeneous Cohort of Patients With Acquired and Inherited Heart Disease

Kelecia Brown; Leah Carnick Ledford; Sequoia Finney; Tasha L. Gill; Julio Mateus Nino

Wake Forest School of Medicine, Charlotte Campus, Charlotte, NC

**Objective**: Women with heart disease are at risk for adverse pregnancy outcomes, including preterm birth (PTB). This study aimed to determine whether the New York Heart Association (NYHA) Functional Classification of heart failure can help to estimate the risk for PTB and to identify the most common indications for PTB in this high‐risk population.

**Study Design**: This was a retrospective cohort study of patients with inherited and acquired heart disease and singleton pregnancies who received care in a cardio‐obstetrics clinic from August 1, 2018, to November 15, 2023. Patients were evaluated by a cardiologist, Maternal‐Fetal Medicine specialist, and nurse practitioner. NYHA classification (I, II, III, IV, or unknown) was assigned at the first visit. PTB was defined as delivery <37 0/7 weeks. Indication for PTB were analyzed. Multivariate logistic regression was used to assess the association between NYHA classification and PTB.

**Results**: Among 119 singleton pregnancies, 25 (21%) had PTB. There were no significant differences in heart disease diagnosis, age, race, BMI, history of PTB and parity between the PTB and term birth groups. The most common indications for PTB included maternal cardiac complications 9 (36%), PPROM 7 (28%), and preeclampsia 4 (16%). Among PTB cases, 11 (31%) had Class I, 9 (25%) had Class II, and 5 (14%) had Class III/IV. Compared with Class I, the odds for PTB were 3.8 times higher with Class II (OR = 3.84, 95% CI:1.37–10.78) and 11 times higher with Class III/IV: OR = 11.36, 95% CI: 2.38–54.35). After adjusting for pre‐pregnancy BMI and history of PTB, the odds of PTB for Class II increased, while the odds of Class III/IV attenuated (Class II: OR = 4.77, 95% CI: 1.56–14.60; Class III/IV: OR = 7.56, 95% CI: 1.25–45.73).

**Conclusion**: The NYHA classification appears to be a useful clinical tool to estimate the risk for PTB. The increasing rates of PTB by NYHA class underscores the importance of preventive measures such as optimization of NYHA during pregnancy and low‐dose aspirin.

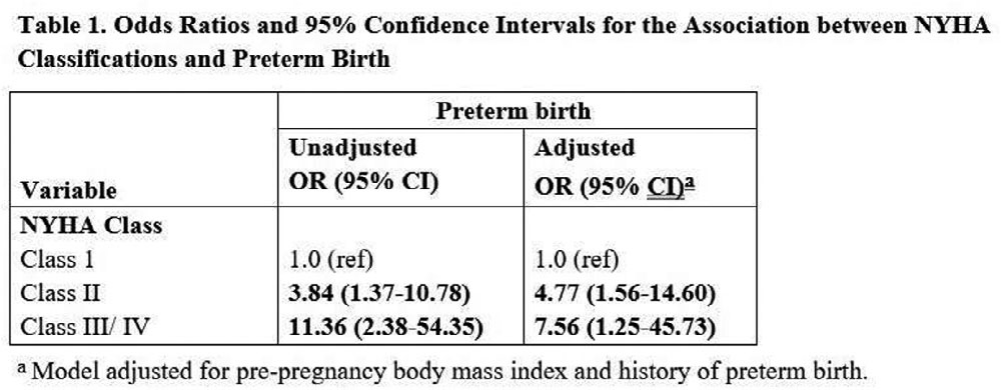

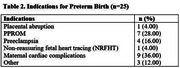

**10:30 AM – 12:00 PM**

## 260 | Reasons for Hospitalization at Periviable Gestation, Interventions, and Subsequent Delivery

Matthew A. Rysavy^1^; Kelly S. Gibson^2^

On behalf of the Eunice Kennedy Shriver National Institute of Child Health and Human Development (NICHD) Maternal‐Fetal Medicine Units (MFMU) Network and Neonatal Research Network (NRN), Bethesda, MD

^1^McGovern Medical School at University of Texas Health Science Center Houston, McGovern Medical School at University of Texas, Houston, TX; ^2^MetroHealth Medical Center/ Case Western Reserve University, Cleveland, OH

**Objective**: Management at periviable gestation (20 + 0 ‐ 25 + 6 wk) has changed during the past decade. Prior studies have focused on birth outcomes, yet there has been little insight into characteristics and management of all individuals who are hospitalized in this gestational age range. This study aimed to describe the reasons, interventions, and probability of delivery among pregnant patients hospitalized during periviable gestational ages.

**Study Design**: This was a prospective multicenter cohort study of inpatient hospitalizations at periviable gestation May 2024 ‐ April 2025 at 12 hospitals. All patients with hospitalizations with a live fetus at admission were included except when admission was for termination for reasons other than rupture of membranes, preeclampsia, maternal infection, or vaginal bleeding. Key measures included reasons for hospitalization, antenatal steroids, and antenatal counseling from a neonatal clinician. We calculated the probability of delivery after admission for each reason and for each intervention.

**Results**: Among 1738 pregnant patients, the median (interquartile range) gestation at first hospitalization was 22.9 (21.4–24.3) weeks and median duration of hospitalization was 2 (1–5) days; 197 patients (11.3%) had multiple hospitalizations and 520 (29.9%) delivered at a periviable gestational age. The reasons for hospitalization with greatest probability of periviable delivery were (Table 1) preterm labor (*n* = 222, 74.8% delivered) and premature rupture of membranes (267, 62.9% delivered). Of the 655 patients who received antenatal steroids, 418 (63.8%) delivered during hospitalization. Of the 554 patients who received counseling from a neonatal clinician, 375 (67.7%) delivered during hospitalization.

**Conclusion**: The probability of delivery, antenatal steroids, and antenatal counseling varied substantially by reason for admission. One‐third of patients hospitalized at periviable gestation who received antenatal steroids or counseling from a neonatal clinician did not deliver during hospitalization.

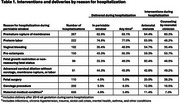

**10:30 AM – 12:00 PM**

## 261 | Access to Home Blood Pressure Monitor on Outcomes of Chronic Hypertension in Pregnancy: CHAP Secondary

Kevin S. Shrestha

On behalf of the CHAP Consortium

Center for Research in Women's Health, University of Alabama at Birmingham, Birmingham, AL

**Objective**: Treatment of mild chronic hypertension (CHTN) to a blood pressure (BP) goal of less than 140/90mmHg improves pregnancy outcomes (CHAP Trial). Home BP monitoring is a practical method of monitoring BP. Thus, we aimed to assess whether having a home blood pressure monitor at home or not modified pregnancy outcomes in the original CHAP trial.

**Study Design**: Secondary analysis of the CHAP trial, an RCT of antihypertensive treatment versus no treatment of pregnant patients with mild chronic hypertension. Ownership of a home BP monitor was by patient report at time of enrollment. The primary outcome was the CHAP composite of pregnancy outcomes. Secondary outcomes included individual components of the composite, severe HTN and others analyzed (Table). We analyzed whether the effect of treatment the outcomes differed by whether the patient had a home BP monitor using linear or logistic regression model as appropriate, with *p* < 0.05 as significant for interaction.

**Results**: Of 2325 patients who reported on home BP monitor, 1298 (55.8%) had a monitor and 1027 did not. Of the patients with a home BP monitor, 50.2%, were in the active treatment arm as were 50.4% of those without home BP monitor. For baseline characteristics, there were no significant interactions between treatment assignment and having a home BP monitor, except healthcare insurance (*p* = 0.0093). Overall, having a home BP monitor did not change the effect of treatment of CHTN on the primary outcome (*p* = 0.625) or any secondary outcome (Table).

**Conclusion**: The effect of treatment of mild CHTN on the CHAP primary composite outcome and other outcomes including severe hypertension and mean BP did not appear to differ by whether pregnant patients had a home BP monitor. Further studies should investigate whether using a home BP influences treatment outcomes.

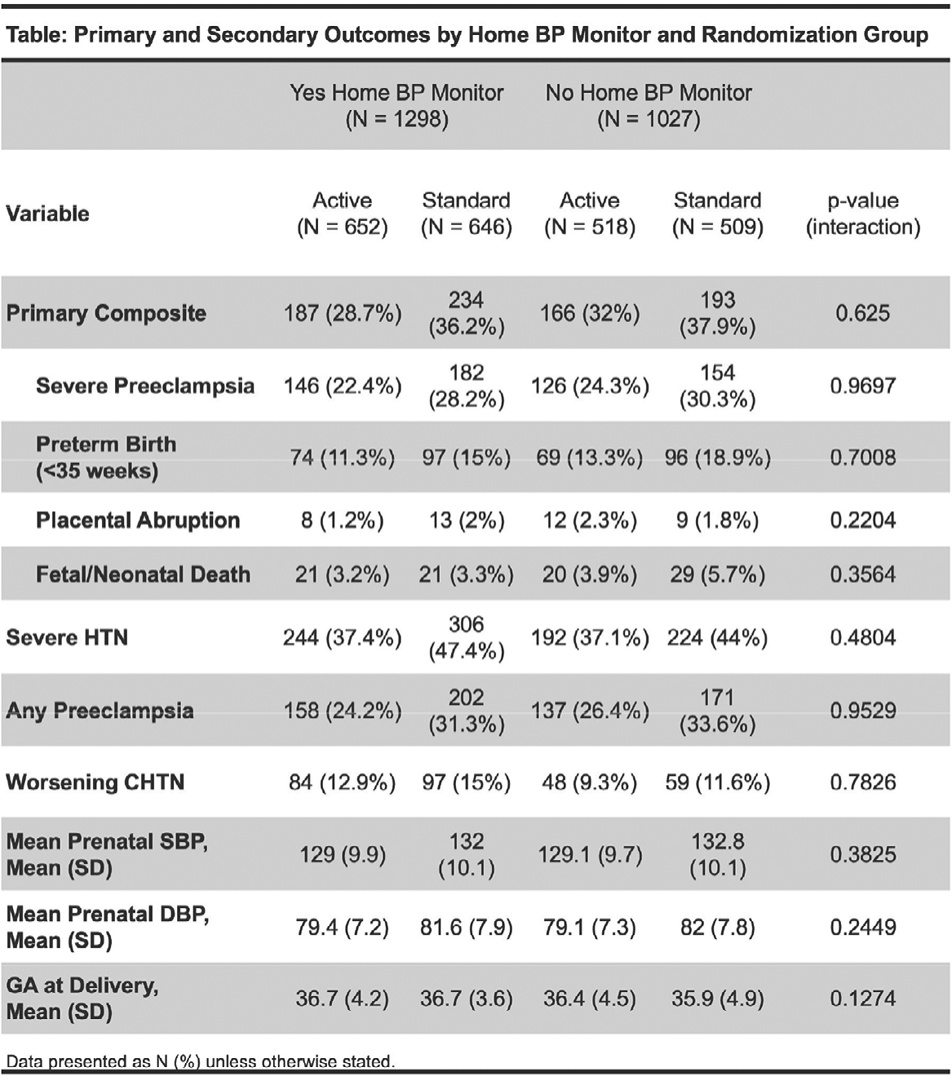

**10:30 AM – 12:00 PM**

## 262 | Evaluation of the Association Between Primary and Secondary Syphilis Incidence and Social Vulnerability Index

Aashri I. Aggarwal^1^; Khushie Matharoo^1^; Misa Hayasaka^1^; Kaitlin Hufstetler^1^; Grace Spencer^2^; Tetsuya Kawakita^3^

^1^EVMS Department of Obstetrics and Gynecology, Macon & Joan Brock Virginia Health Sciences at Old Dominion University, Norfolk, VA; ^2^Eastern Virginia Medical School at Old Dominion University, EVMS OBGYN, Virginia Health Sciences at Old Dominion University, VA; ^3^Eastern Virginia Medical School Department of Obstetrics and Gynecology, Macon & Joan Brock Virginia Health Sciences at Old Dominion University, Norfolk, VA

**Objective**: To examine the association between Social Vulnerability Index (SVI) and primary and secondary (P&S) syphilis incidence in pregnant individuals across the United States (US) over time.

**Study Design**: This was a cross‐sectional study using the Centers for Disease Control and Prevention Natality dataset. Subjects were categorized into quartiles (Qs) based on county‐level SVI, with Q4 being the most vulnerable. The primary outcome was P&S syphilis incidence per 100,000 births. Mixed‐effect generalized linear models with negative binomial distribution were used to quantify disparities in syphilis incidence. Spatial and temporal patterns across quartiles and years were mapped to US counties and visualized according to SVI. A difference‐in‐differences (DID) analysis was used to compare trends in syphilis incidence across different SVI quartiles and time periods.

**Results**: The incidence of P&S syphilis in the US increased by approximately 25,000 from 2016 to 2021. Rates of syphilis increase from 2016 to 2021 at the county‐level are plotted on a U.S. map, showing clusters of spread, with the highest rates being in the southwest US (Figure 1). The estimated incidence of syphilis across all years was graphed for each SVI quartile, showing both the increasing disparity of syphilis rates between SVI quartiles across 2016–2021, and the consistently higher rates of syphilis in more socially vulnerable areas (Figure 2).

The most significant widening disparity was observed between Q4 and Q1, with a difference of incidence increase by 85 cases per 100,000 births in Q4 (95% CI [50, 120]; *p* = 0.003). Similarly, the incidence rate in Q3 had a widening disparity compared to Q2 (DID estimate 35 cases per 100,000 births; 95% CI [6, 67]; *p* = 0.0028). There was no statistically significant difference between SVI Q2 and Q1.

**Conclusion**: P&S syphilis incidence rates increased disproportionately amongst counties with higher social vulnerability, emphasizing the need for targeted interventions geared towards these communities.

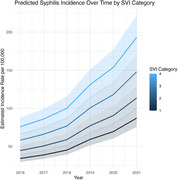

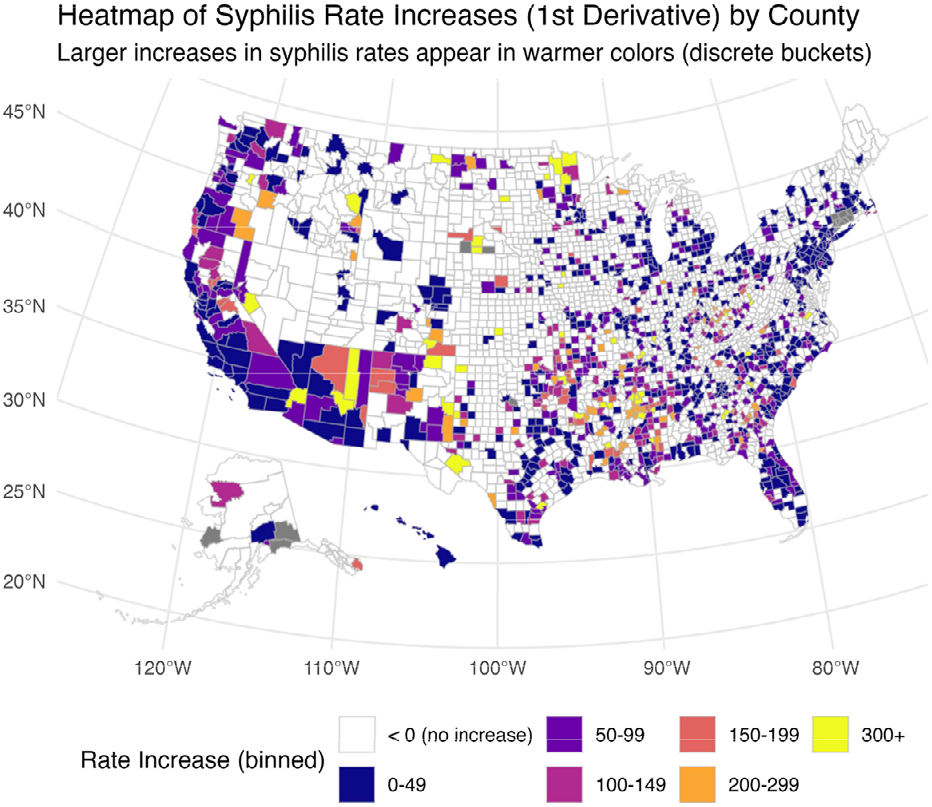

**10:30 AM – 12:00 PM**

## 263 | Aspirin Prescribing and Adherence Patterns for Pregnant Patients in a Safety‐Net System

Kimberly Z. Huynh^1^; Bridgette Blebu^2^; Manasa Kavasery^3^; Erin Saleeby^3^

^1^David Geffen School of Medicine at University of California, Los Angeles (UCLA), Los Angeles, CA; ^2^Lundquist Institute for Biomedical Innovation at Harbor‐UCLA, Torrance, CA; ^3^Harbor‐UCLA Medical Center, Torrance, CA

**Objective**: To review gestational ages at which aspirin (ASA) was prescribed and adherence patterns for patients at moderate and high risk for preeclampsia in a safety‐net system, to inform future implementation strategies where this patient population is often at increased risk for preeclampsia.

**Study Design**: We conducted a retrospective cohort study of 921 deliveries at‐risk for preeclampsia from January to November 2024 of three safety‐net hospitals in California. Among the 537 at‐risk patients who received ASA during pregnancy, maternal demographics, clinical and delivery data, medication reconciliation data of self‐reported adherence for ASA, and adherence through pregnancy to delivery were extracted. Descriptive statistics were used to compare medical and social characteristics with preeclampsia outcomes. A multinomial logistic regression model was used to assess the association between ASA adherence (low, moderate, high), timing of prescription, and preeclampsia risk. An interaction term was included to analyze if preeclampsia risk varied by prescription timing in each adherence group.

**Results**: 59% of those prescribed ASA were high risk with multiple moderate risk factors: Hispanic (64%) or Black (23%), publicly insured (93%) and BMI >30 (76%). There was a significant interaction between timing and adherence, *p* = 0.03. High adherence was more common when aspirin was prescribed <12 weeks (68.7%) than at 12–16 weeks (41.5%). Compared to high adherence, preeclampsia risk was higher in the low (aOR = 4.53, 95% CI: 2.61–7.93) and moderate (aOR = 3.32, 95% CI: 1.86–5.95) adherence groups.

**Conclusion**: In safety‐net hospitals where most patients are high risk for preeclampsia, high adherence to ASA (≥ 75% of self‐reported adherence) during pregnancy significantly reduces preeclampsia risk. Adherence improves when patients are prescribed ASA between 12–16 weeks compared to >16 weeks, with the best adherence seen when prescribed <12 weeks. Implementation strategies such as early prescription and early tailored counseling should be prioritized in safety‐net settings to improve adherence and lower prescribing barriers.

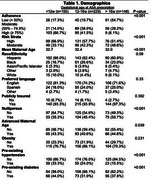

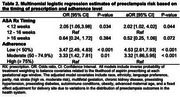

**10:30 AM – 12:00 PM**

## 264 | How to Best Utilize a Urine Protein Creatinine Ratio to Rule Out Preeclampsia

Krishna Majmundar^1^; Fatima Ali^2^; Malek Ghandour^1^; Bernard Gonik^3^

^1^Department of Obstetrics and Gynecology, Wayne State University School of Medicine, Detroit, MI; ^2^C.S Mott Center for Human Growth and Development, Wayne State University, Detroit, MI; ^3^Division of Maternal Fetal Medicine, Department of Obstetrics and Gynecology, Wayne State University, Detroit, MI

**Objective**: 24‐hour urine protein (24UP) collection remains the gold standard for detecting proteinuria in the setting of preeclampsia. This method is time‐consuming and error‐prone, while urine protein creatinine ratio (UPCR) offers a faster, more convenient alternative. The objective of this study is to reevaluate the utility of UPCR in detecting significant proteinuria.

**Study Design**: Inclusion criteria for this retrospective cross‐sectional study included singleton pregnancies with a 24UP collection between 2019–2022 for baseline or diagnostic evaluation. Patients with chronic kidney disease or nephropathy were excluded. Data including 24UP, comorbidities, delivery information and demographics was collected. UPCR was stratified into low (<0.15), intermediate (0.15‐0.29) and elevated (≥ 0.30) categories. Significant proteinuria was defined as 24UP of ≥ 300 mg/day. Concordance of the UPCR calculated from the same sample of 24‐hour urine collection was determined and assessed using the area under the curve (AUC) of the receiver operating characteristic (ROC) curve. Sensitivity, specificity, and positive and negative predictive values (PPV, NPV) of each of the three categories were calculated.

**Results**: A total of 150 patients were included. The ROC revealed an AUC of 0.938. Using a UPCR cutoff of <0.15 yielded sensitivity of 75% with a NPV of 96% for excluding significant proteinuria. Surprisingly, UPCR ≥ 0.30 had a sensitivity of only 50% though PPV was 97% for detecting significant proteinuria. For UPCR values between 0.15–0.29, 28 of 52 patients had significant proteinuria on 24UP yielding a NPV of 27.5% for ruling out significant proteinuria.

**Conclusion**: Our study questions the clinical utility of the standard UPCR cutoff of ≥ 0.30. While this threshold has a high PPV, it misses approximately half of patients with significant proteinuria. Conversely, a UPCR cutoff of <0.15 reliably ruled out proteinuria suggesting that patients with intermediate values (0.15‐0.29) may benefit most from a 24UP collection. Further research should assess how demographics and comorbidities influence the accuracy of these tests.

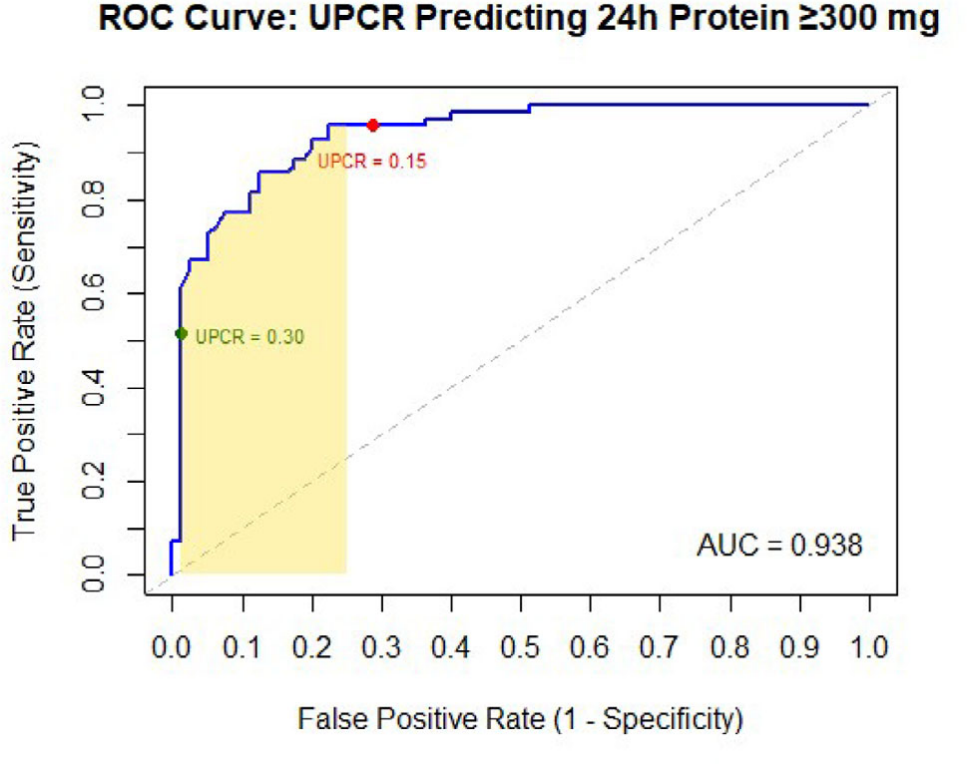

**10:30 AM – 12:00 PM**

## 265 | Postpartum Healthcare Receipt After Spontaneous and Medically Indicated Preterm Birth

Kristan A. Scott^1^; Rachel F. Ledyard^1^; Taneisha R. Sinclair^2^; Anne Mullin^3^; Timothy D. Nelin^4^; Heather H. Burris^1^

^1^Children's Hospital of Philadelphia, Philadelphia, PA; ^2^Children's Hospital of Philadelphia, Children's Hospital of Philadelphia, PA; ^3^Maine Medical Center, Maine Medical Center, ME; ^4^Children's Hospital of Philadelphia, Children, PA

**Objective**: ACOG reports that up to 40% of patients do not attend a postpartum visit. Parents of preterm infants are at higher risk for not receiving postpartum care than those of term infants, but are often sicker with conditions such as hypertensive disorders and postpartum depression. Preterm birth (PTB) can be classified as spontaneous PTB (sPTB) (i.e., premature rupture of membranes or preterm labor) and medically indicated PTB (mPTB) (i.e., clinician‐initiated due to maternal or fetal conditions). We compared postpartum visit attendance after sPTB and mPTB to term birth.

**Study Design**: Retrospective cohort study of births from 2010–2019 in *GeoBirth*, a curated dataset of all births in two hospitals in Philadelphia, PA. Each PTB was manually classified by two blinded reviewers as sPTB or mPTB. We performed bivariate analyses and multivariable, modified Poisson regression models to calculate risk ratios (RR) of no postpartum care receipt after sPTB and mPTB compared to term birth. Models adjusted for age, parity, hospital, body mass index, insurance, education, race and ethnicity, cesarean birth. A linear combination test was used to compare RRs between PTB subtypes.

**Results**: Of the 69,493 included births, 3489 (5.0%) were sPTB, 2770 (4.0%) were mPTB. 696 unclassified PTBs were excluded. Overall, 33.5% had no postpartum visit within 6 weeks, with higher proportions among sPTB (42.8%) and mPTB (38.8%) than term birth (32.8%) (*p* < 0.001) (Table 1). Compared to term birth, the unadjusted risk of no postpartum care was higher after sPTB (RR 1.30, 95% CI: 1.24–1.37) than mPTB (RR 1.18, 1.11–1.26) (p value comparing RRs = 0.015) (Table 2). In model including all covariates, risk was similarly elevated for both sPTB (aRR 1.16, 1.10–1.22) and mPTB (aRR, 1.10, 1.03–1.17) (p value comparing RRs = 0.206).

**Conclusion**: Compared to term births, individuals with both sPTB and mPTB are at higher risk of not attending postpartum visits, despite elevated comorbidity prevalence. Innovative, collaborative, multidisciplinary efforts are essential to meet the needs of this vulnerable population, especially within the NICU.

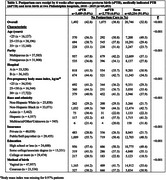

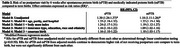

**10:30 AM – 12:00 PM**

## 266 | Possible Syphilis in Pregnancy After Prior Treatment: Low Neonatal Morbidity but a Clinical Conundrum

Kristen A. Warncke^1^; Isabela Anawate^1^; Shreya Battu^1^; Heath Yancey^1^; Kimberly Sladek^1^; Madeline Gallagher^2^; Abigail Clark^1^; Brenda Correra^3^; Nixie Bailey^2^; Jessica E. Pruszynski^1^; Emily H. Adhikari^1^

^1^University of Texas Southwestern Medical Center, Dallas, TX; ^2^Parkland Health, Dallas, TX; ^3^Dallas County Health and Human Services, Dallas, TX

**Objective**: Examine syphilis management and neonatal outcomes in pregnant patients with prior syphilis treatment and possible infection, versus clinically active infection

**Study Design**: This is a retrospective cohort study of pregnant individuals with reactive syphilis serologies at a large public hospital. Treatment history, if available, was confirmed with local public health officials. We excluded individuals considered serofast (serologic response, adequate prior treatment, and no concern for reinfection) and those with documented reinfection (new symptoms, fourfold titer increase or newly reactive RPR). We compared demographics, comorbidities, syphilis characteristics and neonatal outcomes in the remainder considered to have possible infection (+/− serologic response, uncertain adequacy of treatment, or persistent positive titers) vs no reported treatment (active syphilis) prior to the current pregnancy.

**Results**: From January 1, 2010, through June 30, 2025, 898 patients delivered with reactive serologies, including 576(64.1%) reporting no prior treatment (active syphilis) and 322(35.9%) reporting prior treatment. Of the latter, excluded were 149(16 .6%) serofast and 50(5.6%) with documented reinfection. The 123(13.7%) with prior treatment (possible syphilis) had lower RPR titers and were more likely staged as latent, late or unknown duration stage than those with active syphilis. Of these with possible infection, 109 (89%) were given at least 2.4M units IM BPG despite 52(42%) having confirmed previous adequate treatment. Stillbirths from congenital syphilis occurred in 0 previously treated and 8(1%) with active syphilis. Congenital syphilis was less frequent among previously treated (4[3%] vs 86[15%], *p* < 0.001). Nonetheless, 30% of neonates in both groups were treated for possible congenital syphilis with 10 days of IV penicillin (*p* = 0.99).

**Conclusion**: Distinguishing active syphilis infection in seroreactive, previously treated individuals remains a challenge, although neonatal morbidity is low. Improved diagnostics would impact both maternal and neonatal management in this population.

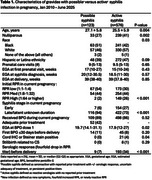

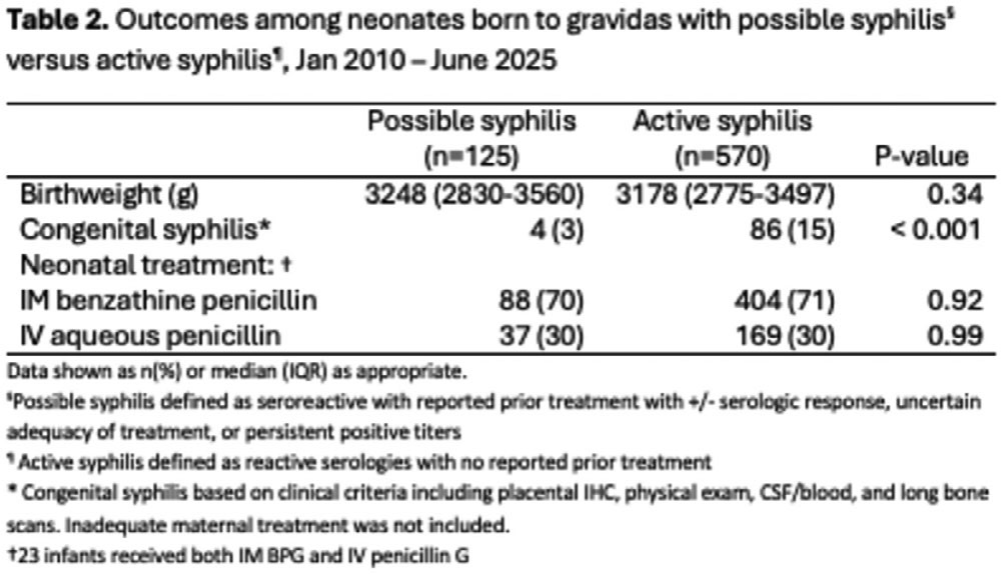

**10:30 AM – 12:00 PM**

## 267 | Prenatal Genetic Diagnosis Post‐Dobbs in an Abortion‐Banned State: An 18‐Month Retrospective Cohort Study

Kristina M. Carter^1^; William L. Riley^2^; Alix Hunzicker^1^; Alicia Mastronardi^1^; Alissa Paudel^3^; Rebecca Purvis^4^; Kimberly B. Fortner^5^; Jordan E. Lewis^1^; Nikki B. Zite^1^; Jill M. Maples^6^

^1^University of Tennessee Graduate School of Medicine, Knoxville, TN; ^2^Georgia Perinatal Consultants, Milton, GA; ^3^University of Tennessee Medical Center, Knoxville, TN; ^4^University of Tennessee Health Science Center College of Medicine‐ Knoxville, Knoxville, TN; ^5^University of Tennessee College of Medicine‐ Knoxville, Knoxville, TN; ^6^University of Tennessee Health Science Center COM Knoxville, Knoxville, TN

**Objective**: Abortion was banned with few exceptions in Tennessee following the decision in Dobbs v. Jackson Women's Health Organization (Dobbs) in 2022. Impacts of abortion restrictions or bans on rates of prenatal diagnostic genetic testing (PDGT) is uncertain. This study aims to evaluate the rate of PGDT in a period prior to and post the Dobbs decision to further assess whether the changing abortion laws had an impact on rates of PDGT.

**Study Design**: This retrospective cohort study was performed evaluating women undergoing PDGT via chorionic villus sampling (CVS) or amniocentesis at a single tertiary referral center. The comparison groups included procedures performed pre‐Dobbs (July 2018–Dec 2019) and post‐Dobbs (July 2022‐Dec 2023). The primary outcomes were rates of CVS and amniocentesis during these periods. Demographic information, ultrasound findings, prior screening results, and details of the procedure were collected. All pregnancies electing PDGT were included, however, procedures performed due to fetal demise or purposes other than genetic testing (fetal lung maturity, amnioreduction, fetal therapy, or amnio dye testing) were excluded.

**Results**: During the study period, 240 women underwent PDGT, and after exclusions, 219 procedures remained for analysis (92 pre‐Dobbs vs.127 post‐Dobbs). Pre‐Dobbs, CVS accounted for 18.5% of procedures, compared to 26.0% post‐Dobbs, which corresponded to a significant increase in the rate of CVS procedures (*p* = 0.03) (Figure). A higher proportion of PDGT were performed in women without abnormal ultrasound findings or abnormal screening results post‐Dobbs (30% vs 17%, *p* = 0.02) (Table).

**Conclusion**: Rates of prenatal diagnostic genetic testing were higher following the Dobbs decision. In addition, a larger proportion of women underwent first‐trimester genetic testing (CVS), allowing earlier information and increased time for decision‐making. While our study cannot determine the reasons for this shift, further research aimed at understanding the reasons behind the desire for genetic testing may further inform about the impact of abortion bans on patient decision making.

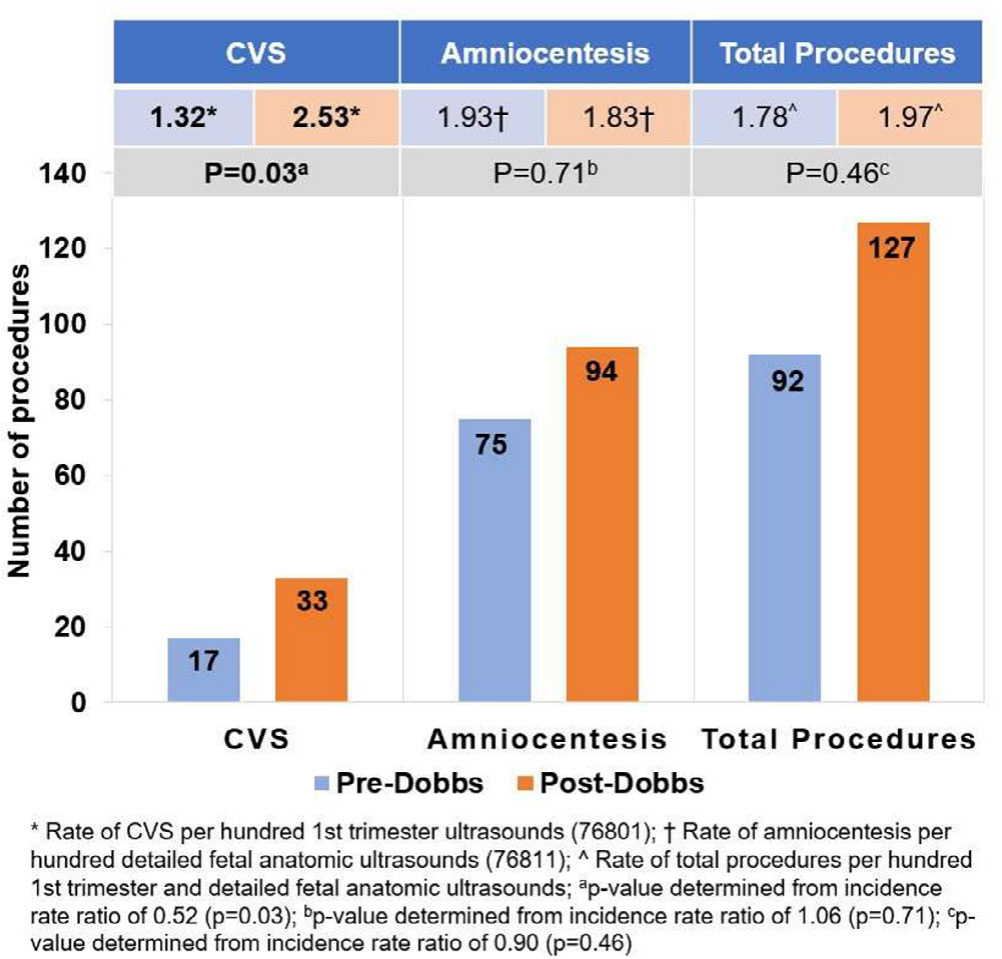

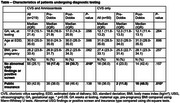

**10:30 AM – 12:00 PM**

## 268 | Hemoglobin A1C Versus 50 g OGTT for Diabetes Screening in Early Pregnancy

Laura C. Colicchia^1^; Patricia Harper^2^; Sandra Castro‐Pearson^3^; Anna K. Schulte^2^; Gretchen Benson^4^; Abbey C. Sidebottom^2^

^1^Allina Health/Minnesota Perinatal Physicians, Minneapolis, MN; ^2^Allina Health, Minneapolis, MN; ^3^Allina Health Clinical Research, Informatics, and Analytics, Minneapolis, MN; ^4^MHIF, Minneapolis, MN

**Objective**: Screening for early GDM or undiagnosed type 2 DM is recommended for pregnant patients meeting high risk criteria, but the ideal screening test is unknown. Traditional oral glucose challenge testing requires glucose load prior to lab draw which requires additional clinic and staff time and may be declined by patients. We hypothesized that screening completion rates would be higher with HgbA1C as the initial DM screen versus GTT.

**Study Design**: Our clinical protocol for early pregnancy DM screening for high risk patients changed in 2022 from 50 g GTT followed by 100 g 3hr GTT if >140, to HgbA1C. Patients with HgbA1C >6.4 were diagnosed with DM. Patients with HgbA1C 5.9–6.4 underwent 3hr GTT. Patients with HgbA1C <5.9 screened negative for early DM. Carpenter‐Coustan criteria were used for 3hr GTT. We performed a retrospective cohort study of all patients meeting criteria for early DM screening for 1 year pre‐ and post‐ protocol implementation. Primary outcomes included completion of DM screening prior to 20 weeks and time between OB intake and screening completion. Secondary outcomes included proportion of each cohort diagnosed by early versus routine GDM screening and obstetric and neonatal outcomes for each screening cohort.

**Results**: 1656 patients were included in this study (840 under GTT screening protocol, 816 under HgbA1C screening protocol. 89.7% of HgbA1C patients completed early DM screening versus 83.6% of GTT patients, *p* < 0.001. Median time to completion was shorter in the HgbA1C group (20 (9,33) vs 25 (13,34) days, *p* < 0.001). Early diabetes was diagnosed more frequently in the GTT cohort (111/840, 13.2% vs 21/816, 2.6%, *p* < 0.001). GDM was diagnosed more frequently at 28 week screening in the A1C cohort (154/771, 20.0% vs 106/702, 15.1%, *p* = 0.046) Obstetric and neonatal outcomes including preeclampsia, miscarriage, C‐section, preterm birth, macrosomia and shoulder dystocia did not differ between cohorts.

**Conclusion**: Using HgbA1C as an initial screen for diabetes in early pregnancy results in higher screening rates and faster completion, but results in lower rates of diagnosis early in pregnancy.

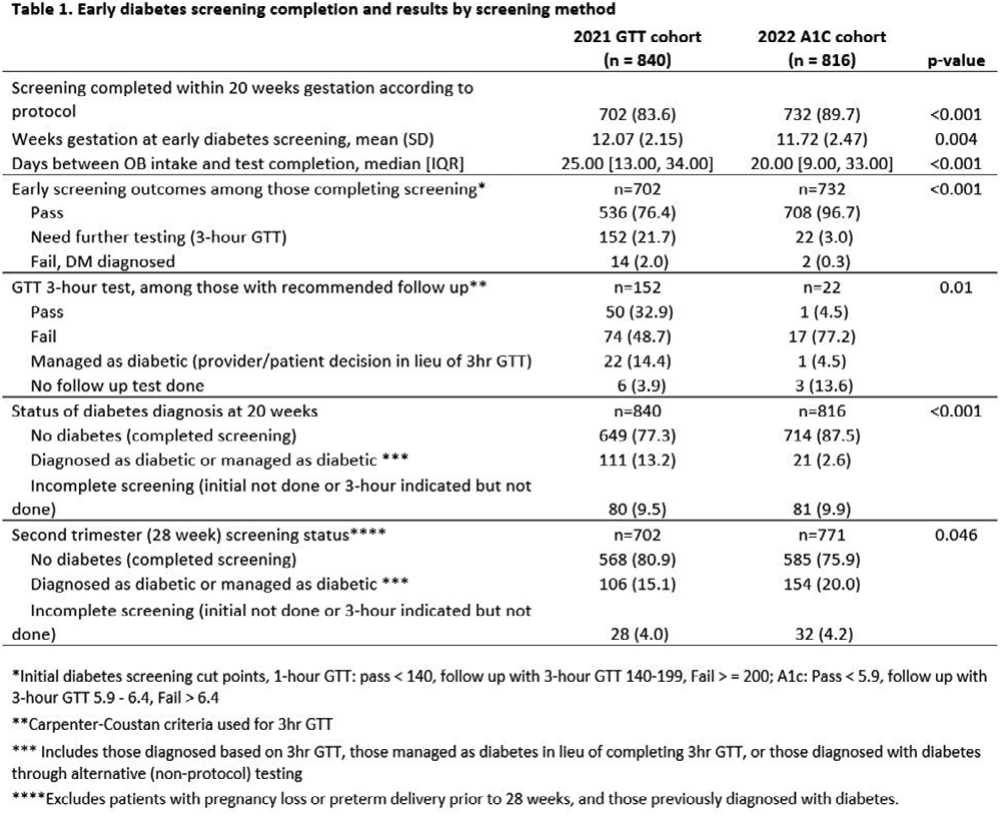

**10:30 AM – 12:00 PM**

## 269 | Hypertensive Disorders of Pregnancy and Long‐Term Cardiomyopathy Risk: A Systematic Review and Network Meta‐Analysis

Laura Queiroz^1^; Maria Lemos^2^; Ayesha Ayesha^3^; Paweł Łajczak^4^; Yami Bhaskar^5^

^1^Santa Casa de Misericórdia de Belo Horizonte, Belo Horizonte, Minas Gerais; ^2^Kurah Women's Health Institute, Montverde, FL; ^3^Shifa College of Medicine, Islamabad, Islamabad; ^4^Medical University of Silesia, Katowice, Slaskie; ^5^Vydehi Institute of Medical Sciences and Research Centre, Bangalore, Karnataka

**Objective**: This study aims to evaluate the long‐term risk of cardiomyopathy, including dilated cardiomyopathy (DCM), associated with hypertensive disorders of pregnancy (HDP), by comparing gestational hypertension and preeclampsia to normotensive pregnancies, as well as to each other, using a network meta‐analysis.

**Study Design**: A comprehensive literature search was conducted across PubMed, Cochrane Library, Embase, and Web of Science to identify studies published up to July 2025. Studies were included if they compared hypertensive disorders of pregnancy in relation to cardiomyopathy outcomes, including dilated cardiomyopathy. Data were analyzed using a random‐effects model, and heterogeneity was assessed using I^2^ statistics in R software.

**Results**: Regarding cardiomyopathy (Figure 1), women with a history of gestational hypertension had a significantly increased risk compared to normotensive pregnancies (OR 2.10; 95% CI 1.56–2.81). A similar increase was observed for preeclampsia versus normotensive pregnancies (OR 1.98; 95% CI 1.62–2.42). No significant difference was found between gestational hypertension and preeclampsia (OR 1.06; 95% CI 0.77–1.46). For DCM (Figure 2), both gestational hypertension (OR 2.70; 95% CI 1.71–4.26) and preeclampsia (OR 2.64; 95% CI 1.80–3.89) were associated with increased long‐term risk compared to normotensive pregnancies. The comparison between gestational hypertension and preeclampsia remained non‐significant (OR 1.02; 95% CI 0.57–1.82).

**Conclusion**: HDP significantly increases the long‐term risk of cardiomyopathy and DCM compared to normotensive pregnancies. These findings emphasize the importance of accurate diagnosis and proper management during pregnancy, as well as continued cardiovascular monitoring and follow‐up after delivery in women with a history of hypertensive disorders during pregnancy.

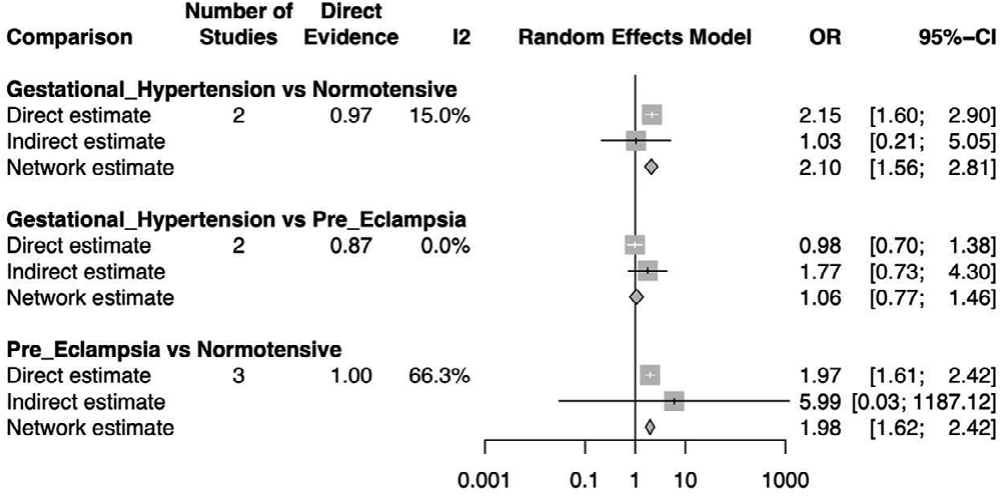

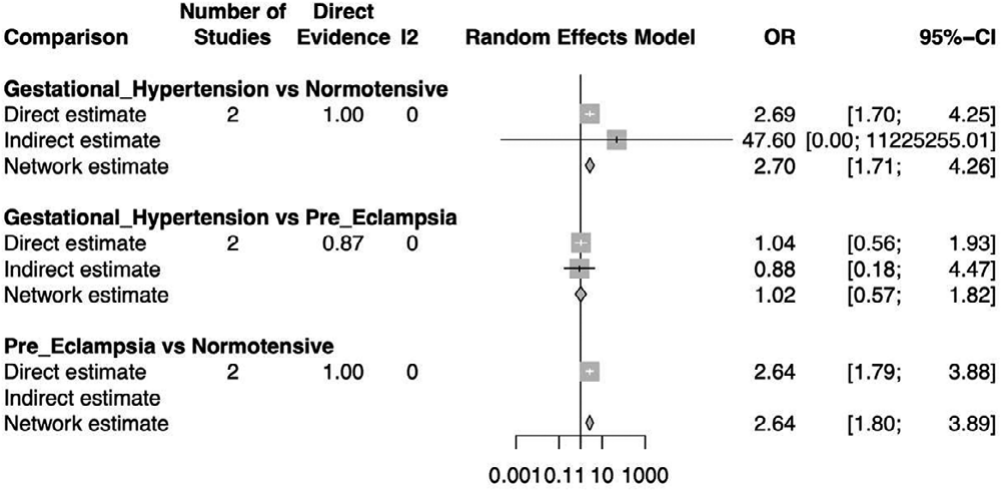

**10:30 AM – 12:00 PM**

## 270 | Under Pressure: Exploring Relationships Between Hypertensive Disorders of Pregnancy and Abnormal Aortic and Valvular Anatomy

Lauren E. Holroyd; Bryan L. Aaron; Levi J. Anderson; Tanuka Piech; Laine Boitos; David E. Hamilton; Melinda B. Davis; Elizabeth S. Langen; Ashley M. Hesson

University of Michigan, Ann Arbor, MI

**Objective**: Abnormal aortic morphology predisposes to hypertensive disorders of pregnancy (HDP). However, bicuspid aortic valve (BAV) with or without aortopathy has not been characterized as a risk for HDPs. We investigate the degree of and change in aortic diameter in pregnant people with BAV and the association of these parameters with HDPs.

**Study Design**: Pregnancies in people with BAV from 2012–2025 in an academic Cardio‐Obstetrics program were identified by ICD codes. Pregnancies were excluded if patients had prior aortic valve surgery or incomplete records. Clinical outcomes and echocardiographic parameters were abstracted. Random forest modeling and bootstrapped logistic regression simulated effect sizes in the setting of rare outcomes. Occurrence of an HDP was modeled with respect to patient age, aortic dilation (< or >4 cm), anatomic location of dilation, and progression of aortic dilation (< or >3mm) over pregnancy.

**Results**: 73 patients and 100 pregnancies were reviewed, 23 experienced an HDP (Table 1). 80 pregnancies had >1 ECHO. 21.3% (*N* = 17) demonstrated aortic dilation >4 cm, with 10.0% (*N* = 8) showing progression through pregnancy. The ascending aorta was the most common location for abnormal dilation (*N* = 39, 48.8%). In predicting an HDP, patient age was the most important factor, followed by the presence of abnormal aortic dilation (Figure 1 top); the location of and change in dilation over pregnancy did not contribute. Both progression and degree of dilation were unevenly distributed by HDP‐type (Figure 1 bottom), with severe forms of disease being disproportionately represented in those with expanding and abnormally dilated aortas. In a bootstrapped regression model predicting severe HDP, abnormal dilation was a significant predictor (absolute risk increase of 0.11, *p* = 0.02). Progressive dilation was not a significant predictor of severe HDP (*p* = 0.08).

**Conclusion**: Having a dilated aorta with BAV is independently associated with severe HDP occurrence. Risk mitigation strategies should be considered in this population even in the absence of classic HDP risk factors.

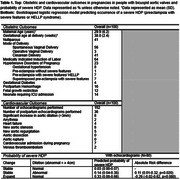

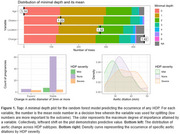

**10:30 AM – 12:00 PM**

## 271 | Breastfeeding Peer Counseling and Breastmilk Feeding Disparities in Very Low Birthweight Infants

Lauren Keenan‐Devlin^1^; Ann E Borders^2^; Andrew Franklin^1^

^1^Endeavor Health, Evanston, IL; ^2^Endeavor Health, Evanston Hospital, Evanston, IL

**Objective**: Breastmilk is medicine for very low birthweight (VLBW) infants <1500 g, but Black VLBW infants are less likely to receive breastmilk feedings than white infants. We examined whether a breastfeeding peer counseling (BPC) intervention reduced racial disparities in breastmilk feeding for VLBW infants.

**Study Design**: BPC was delivered as standard care for all infants hospitalized in the neonatal intensive care unit (NICU) starting January 2024. The BPC counseled NICU families on the benefits of breastfeeding and assisted with breast pumping, milk management, and infant feeding as often parents visited or requested. Feeding flowsheets were abstracted for all infants discharged pre‐intervention 2015–2020 and in the first program year 2024. Race and ethnicity were abstracted from the infant medical record. Breastmilk feeding at discharge was coded if the infant received any breastmilk (mother's own milk or donor milk) in the 24 hours prior to hospital discharge, and proportion of breastmilk feeds was also calculated.

**Results**: Data were available for 623 infants over the study period, 522 pre‐intervention and 101 in the first program year. The average dyad received 2.6 encounters with the BPC, and the range was 1–27 encounters depending on infant length of stay. Percent of breastmilk feedings in the 24 hours prior to discharge for the overall NICU population increased from 27% in the year pre‐intervention to 33% in the first program year (Fig 1). Among VLBW infants, the percent receiving breastmilk prior to discharge increased from 54% to 85% (Fig 2). Pre‐intervention, white infants were more than twice as likely to receive breastmilk at discharge compared to Black infants (61% versus 29%), however after the first program year the gap shrank by 16 percentage points (86% versus 70%).

**Conclusion**: A BPC intervention was associated with an increase in breastmilk feedings for VLBW infants and a reduction in breastfeeding disparities. Future research should evaluate the strategies of BPC associated with disparities reduction to improve lactation care.

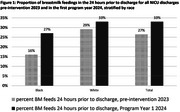

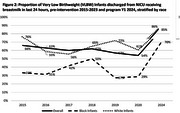

**10:30 AM – 12:00 PM**

## 272 | Accelerations or Variable Decelerations Among Preterm Birth at 22.0–31.6 Weeks: Associated Neonatal Composite Adverse Outcomes

Lauren C. London^1^; Ha L. Tran^2^; Cassidy A. O'Sullivan^3^; Gabrielle Montlouis^4^; Adam Wright^3^; Kelley Kovatis^3^; Rachel L. Wiley^5^; Anthony C. Sciscione^1^; Suneet P. Chauhan^6^

^1^Christiana Care Health System, Newark, DE; ^2^Nemours Children's Health, Newark, DE; ^3^Christiana Care Health Services, Newark, DE; ^4^Rowan‐Virtua, Stratford, NJ; ^5^Division of Maternal Fetal Medicine, Department of Obstetrics, Gynecology and Reproductive Sciences, University of California San Diego, San Diego, CA; ^6^Delaware Center for Maternal‐Fetal Medicine of Christiana Care, Newark, DE

**Objective**: There is a paucity of data on the association of adverse neonatal outcomes with accelerations or variable decelerations in fetal heart rate tracings (FHRT) in the very preterm (22.0–31.6 weeks) period. We sought to address this knowledge gap.

**Study Design**: A retrospective review of all deliveries (Jan to Dec 2023) at a tertiary hospital. Inclusion criteria were non‐anomalous singletons delivered at 22.0–31.6 weeks, where at least 10 minutes of FHRT was available, and neonatal resuscitation was initiated. An obstetrician—blinded to all outcomes—reviewed the FHRT (0–120 min proximal to delivery, at 20 min epochs), and outcome data was obtained from chart review. The primary outcome was short‐term neonatal morbidity or mortality (STNMM); the secondary outcome was long‐term neonatal morbidity or mortality (LTNMM). Descriptive statistics with 95% confidence intervals (CI) were calculated with non‐overlapping CI as significant.

**Results**: Among the 6521 deliveries, 169 (2%) occurred at 22.0–31.6 weeks, of which 99 (58%) met the inclusion criteria. Intrapartum magnesium sulfate was administered in 97% of deliveries and antenatal corticosteroids in 98%. Accelerations of any type were present in 61% (95% CI 51–71%) of FHRT. Accelerations of 10x10 beats per minute (BPM) occurred in 41% (95% CI 31–52%) of FHRT and were associated with a significantly lower likelihood of STNMM (32%; 95% CI 18–48%) than no acceleration (74%; 95% CI 54–89%). Variable decelerations occurred in 59% (95% CI 48–68%), and the likelihood of STNMM was similar for those without deceleration. The LTNMM did not significantly differ among those with or without accelerations, nor those with and without variable decelerations (Table 1).

**Conclusion**: Among singletons delivered at 22.0–31.6 weeks, accelerations and variable decelerations occur in the majority, and while accelerations of at least 10x10 bpm significantly lowered the likelihood of STNMM, they are not associated with LTNMM. The presence of variable decelerations was not associated with STNMM or LTNMM.

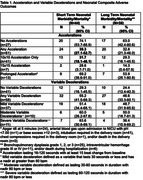

**10:30 AM – 12:00 PM**

## 273 | Use of Prostaglandins for Labor Induction in Pregnancies Complicated by Fetal Growth Restriction

Lauryn Adams^1^; Kai Holder^1^; Alicia Liu^2^; Oluwaseyi Ajayi^3^; Francesca Facco^4^

^1^UPMC Magee‐Womens Hospital, Pittsburgh, PA; ^2^UPMC Magee Womens Hospital, UPMC/Pittsburgh, PA; ^3^UPMC, Pittsburgh, PA; ^4^UPMC, UPMC, PA

**Objective**: Practice patterns for induction of labor (IOL) in pregnancies affected by fetal growth restriction (FGR) often vary by provider due to a lack of evidence‐based guidelines and concerns regarding labor intolerance with prostaglandin (PG) use. Prior studies are limited by restrictive exclusion criteria and omission of key confounding variables. We aimed to evaluate whether PG use during IOL in FGR pregnancies is associated with cesarean delivery (CD).

**Study Design**: This retrospective cohort study included singleton pregnancies complicated by FGR undergoing IOL between January 2021 and December 2023 at a single tertiary care center. FGR was defined as an estimated fetal weight (EFW) or abdominal circumference <10th percentile on the most recent ultrasound (US). Exclusion criteria included multiple gestation, delivery <23 weeks, planned CD, and contraindications to PG use. Maternal demographics, IOL characteristics (including modified Bishop scores [BS]), US findings, and delivery outcomes were abstracted. Categorical variables were compared using chi‐square tests and continuous variables with t‐tests; linear and logistic regression were used to control for confounding variables.

**Results**: Of 1230 pregnancies, 312 met inclusion criteria (168 received PG; 144 did not). PG use was more common in nulliparous patients and those with BS ≤5 (Table). PG use was not associated with higher CD rates (20.1% vs. 22.6%, *p* = 0.595); this finding persisted after excluding favorable BS and adjusting for parity (OR 0.98, 95% CI 0.52–1.83). CD rates did not differ by BS, parity, severity of FGR, or umbilical artery Dopplers. The primary CD indication of non‐reassuring fetal heart rate was similar between groups (18.4% vs. 13.8%, *p* = 0.612). PG use was associated with a modest, non‐significant increase in labor duration (19.8 vs. 17.1 hours, *p* = 0.067), which attenuated after adjusting for parity (*p* = 0.239).

**Conclusion**: PG use in IOL for pregnancies complicated by FGR was not associated with increased rates of CD. These findings support the use of PG for cervical ripening in pregnancies affected by FGR.

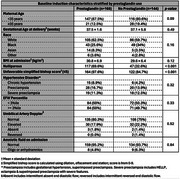

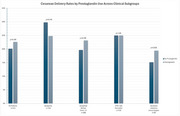

**10:30 AM – 12:00 PM**

## 274 | Comparing the Performance of a Novel Maternal Sepsis Screening Score to Clinician Judgement

Leah A. Chen^1^; Alexa Henderson^2^; Anna B. Binstock^3^; Christina J. Megli^4^

^1^University of Pittsburgh Magee Women's Hospital, Pittsburgh, PA; ^2^UPMC Magee Women's Hospital, University of Pittsburgh Magee Women's Hospital, PA; ^3^UPMC Magee‐Womens Hospital, UPMC, PA; ^4^University of Pittsburgh Magee Women's Hospital, University of Pittsburgh Magee Women's Hospital, PA

**Objective**: The physiologic changes of pregnancy pose challenges to establishing sepsis screening thresholds. To address this, our institution developed a sepsis alert system with a maternal sepsis score (MSS), with the goal of accelerating obstetric sepsis recognition and treatment. A sepsis work‐up order set was also added and used at clinician discretion, including blood cultures, antibiotics, and fluid resuscitation. The MSS uses obstetric adjusted Systemic Inflammatory Response Syndrome (SIRS) criteria in combination with signs of organ dysfunction (Figure 1). We performed a retrospective analysis to compare the performance of MSS to “clinician judgement” (clinician‐initiated sepsis workups) and previously established maternal sepsis scores.

**Study Design**: Inclusion criteria consisted of patients who were pregnant or within 6 weeks postpartum and had an MSS alert or clinician‐initiated sepsis work‐up from February to August 2024. The clinical course, vital signs, and labs were abstracted. Sepsis was defined if patients had sustained vital sign or lab abnormalities and a pathogenic infection with an identified organism. We compared MSS with SIRS, Maternal Early Warning Trigger (MEWT), and Maternal Early Warning Criteria (MEWC).

**Results**: Out of 208 cases, the true sepsis rate was 36%. Most false positive MSS alerts were in patients with normal labor or intra‐operative Cesarean section (28%), or in those with an isolated postpartum hemorrhage (20%). MSS alone had a poor PPV for maternal sepsis (0.25). There were 7 cases where clinician judgement and MSS overlapped. These limited cases had a PPV of 0.57. Clinical judgement alone had a PPV of 0.52. When tested on our cohort, unmodified SIRS, MEWC, and MEWT also had low PPVs of 0.37, 0.32, and 0.39.

**Conclusion**: Our study supports prior work highlighting the complexity of creating clinically useful obstetric sepsis screening protocols, especially when applied universally across pregnancy stages and postpartum. This data also reinforces the need for experienced providers, as clinician judgement had a PPV rivaling established obstetric sepsis screening scores.

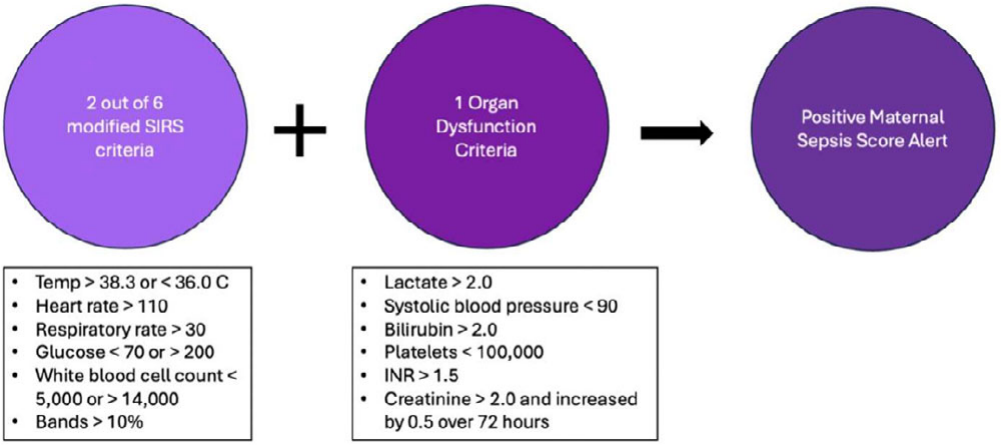

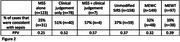

**10:30 AM – 12:00 PM**

## 275 | Automation in Uterine Contraction Recognition: A Preliminary Step Toward Predicting Spontaneous Preterm Birth

Vivian Pae^1^; Wesly Brooks^2^; Haley Shah^1^; Chelsey King^3^; Maya A. Sahtout^3^; Sharon Yuen^1^; Herman Hedriana^4^; Philip Strong^4^; Lihong Mo^4^

^1^School of Medicine, UC Davis, Sacramento, CA; ^2^Data Science and Informatics, UC Davis, Davis, CA; ^3^UC Davis School of Medicine, Sacramento, CA; ^4^Department of Obstetrics and Gynecology, UC Davis, Sacramento, CA

**Objective**: Spontaneous preterm birth (sPTB) remains a major contributor to neonatal morbidity and mortality. Uterine contractions are a known antecedent, but their patterns and physiological significance remain poorly quantified in clinical practice. Tocometry provides a non‐invasive measure of uterine activity that are widely collected but the interpretation is subjective. We aim to develop a preliminary machine learning (ML) framework that characterizes uterine contraction patterns in patients presenting with preterm contractions, as a preliminary step toward predictive modeling of sPTB.

**Study Design**: We conducted a retrospective case‐control study of patients with preterm contractions between 2016 and 2024. A total of 2925 patients were screened to identify three groups: (1) those with spontaneous preterm birth (sPTB), (2) those with preterm contractions without sPTB, and (3) those without preterm contractions. Cases were included if they had intrauterine pressure recordings between 10 minutes and 2.75 hours. In total, 310 cases were included: 114 with sPTB and 196 with resolved preterm contractions. We extracted quantitative features from the tocometer time series, including the median interval between contractions, median amplitude, and proportion of waveform segments showing increasing pressure trends. These features were analyzed using logistic regression with 10‐fold cross‐validation to explore their discriminatory potential between sPTB and non‐sPTB cases.

**Results**: The model correctly identified 57 of 114 sPTB cases and 112 of 196 non‐sPTB cases; however, the overall classification performance was not significant (χ^2^, *p* = 0.27). Representative tracings illustrated in Figure.

**Conclusion**: This study demonstrates the feasibility of algorithmic analysis of uterine contraction patterns using tocometer waveforms. While not predictive in isolation, these waveform features represent a promising step toward developing more robust models for sPTB risk stratification. Future studies leveraging larger multicenter datasets and incorporating multimodal features are warranted.

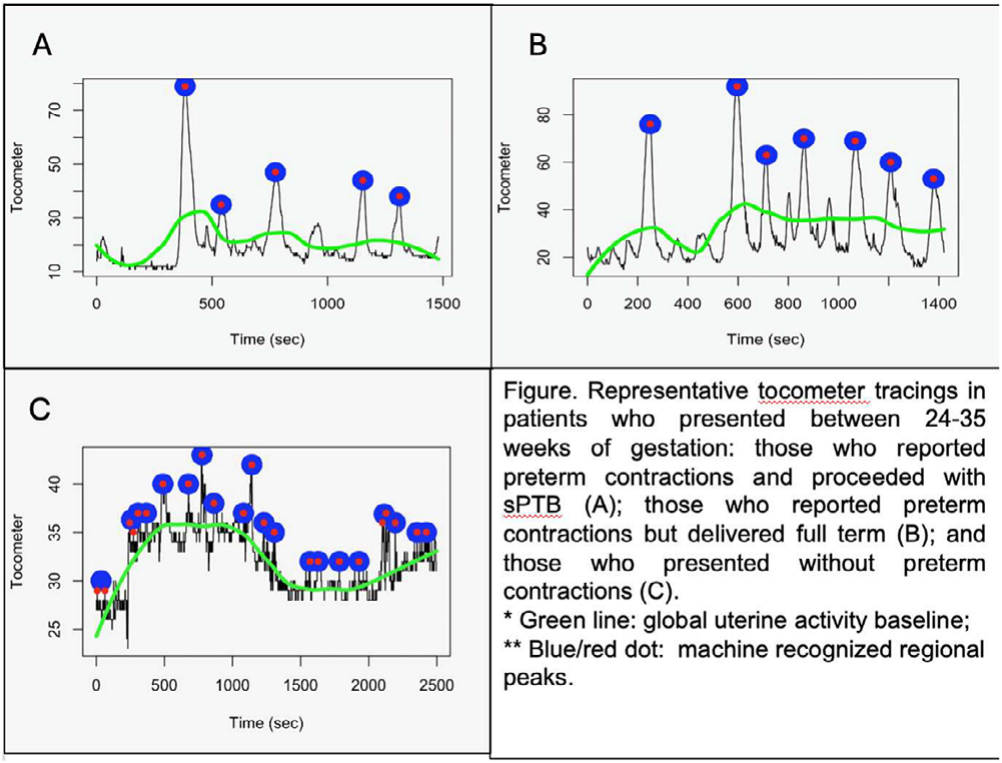

**10:30 AM – 12:00 PM**

## 276 | Food and Housing Insecurity and Breastfeeding in Low‐Come Pregnant Women: The CRADLE Study

Lilly Picklesimer^1^; Veronica A. Mize^2^; Jessica H. Britt^2^; Lu Zhang^3^; Moonseong Heo^4^; Amy H. Crockett^2^

^1^The Ohio State University, The Ohio State University, OH; ^2^Prisma Health/University of South Carolina School of Medicine Greenville, Greenville, SC; ^3^Clemson University, clemson, SC; ^4^Clemson University, Clemson, SC

**Objective**: Food and housing security may promote breastfeeding (BF) by ensuring adequate nutrition, reducing chronic stress, and allowing for safe and private environments. Evidence on the association between food and housing security and BF is inconsistent with small sample sizes. We examined the relationship between food and housing insecurity on BF initiation and continuation in a large, well‐defined cohort of low‐income pregnant women.

**Study Design**: Secondary analysis of the Cradle randomized clinical trial evaluating group prenatal care. Participants completed surveys of demographics, food and housing security, and perceived stress scales. Medical records were abstracted for BF initiation at hospital discharge and at 6 weeks postpartum. Participants were divided between food secure and insecure and housing secure and insecure. Chi square and t‐tests differences between groups. Multivariable logistic regressions adjust for race and age.

**Results**: 2160 participants, all of whom completed food security screens; 1905 completed housing security screens. Food insecurity (555, 25.7%) was more common than housing insecurity (359, 18.9%). Participants with food insecurity were less likely to be married (19.1% vs 25.0%), more likely to be White (42.3% vs 34.7%), to have unintended pregnancy (71.5% vs 65.3%) and smoke before (45.4% vs 33.1%) and during pregnancy (26.3% vs 15.1%), *p* < 0.01 for all. Housing insecure participants had similar characteristics but were older (26.0 vs. 25.0, *p* < 0.01). Higher perceived stress scores were seen for both food (7.0 vs. 4.0) and housing (7.0 vs 5.0) insecurity (*p* < 0.01). BF initiation or continuation was not significantly associated with housing insecurity (aOR: 1.14 [0.87–1.50] and 1.02 [0.76–1.35], respectively) or food insecurity (aOR: 1.17 [0.92–1.48] and 1.26 [0.98–1.62], respectively).

**Conclusion**: Rates of BF were not affected by food or housing insecurity. Further studies should explore contributing factors of relative success in this group since these may provide insight that can be leveraged for improving other health behaviors for patients with food or housing insecurity.

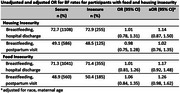

**10:30 AM – 12:00 PM**

## 277 | Altered Cord Blood Metabolomic Profile in Term‐Born Fetuses Following Threatened Preterm Labor

Lina Youssef^1^; Natalia Abadia‐Cuchi^2^; Juan Lerma‐Irureta^2^; Jesus Orduna^3^; Irene Orera^3^; Ana Medel‐Martinez^2^; Marta Fabre^2^; Jesus Gonzalez^2^; Pilar Maestro‐Quibus^2^; Sara Ruiz‐Martinez^2^; Jon Schoorlemmer^4^; Diego Lerma^5^; Daniel Oros^2^; Cristina Paulés^6^

^1^BCNatal–Barcelona Center for Maternal‐Fetal and Neonatal Medicine (Hospital Clínic and Hospital Sant Joan de Deu), IDIBAPS, University of Barcelona, Barcelona, Catalonia; ^2^Instituto de Investigación Sanitaria de Aragón (IIS Aragón), Zaragoza, Aragon; ^3^Departamento de Química Orgánica, Universidad de Zaragoza, Zaragoza, Aragon; ^4^Placental Pathophysiology & Fetal Programming Research Group, GIIS‐028 del IISA, Zaragoza, Aragon; ^5^Obstetrics Department, Hospital Clinico Universitario Lozano Blesa, Zaragoza, Aragon; ^6^Hospital Clinico Universitario Lozano Blesa, Hospital Clinico Universitario Lozano Blesa (Zaragoza), Aragon

**Objective**: To examine the cord blood metabolomic profile of fetuses exposed to threatened preterm labor (TPL), comparing those born preterm with those who ultimately delivered at term.

**Study Design**: This prospective observational study included 42 singleton pregnancies exposed to TPL and 40 unexposed controls. Cord blood was collected at delivery and analyzed using LC‐MS/MS‐based metabolomics. Both multivariate and univariate statistical methods were applied.

**Results**: Among TPL‐exposed pregnancies, 27 (64.3%) delivered preterm, while 15 (35.7%) delivered at term. Of the 507 metabolites analyzed, 506 were detected in at least 70% of samples. Multivariate analysis using principal component analysis (PCA) and partial least squares discriminant analysis (PLS‐DA) showed distinct separation between TPL cases and controls. PCA revealed that the first two components explained 22.2% and 8.4% of the variance, while PLS‐DA explained 60.3% and 39.7%, respectively. Notably, term births following TPL exhibited an intermediate metabolomic profile. Univariate analysis identified 122 metabolites with significant differences across groups, supporting the multivariate findings. Hierarchical clustering of the top 25 metabolites revealed three distinct clusters: one predominantly composed of preterm births, one of controls, and an intermediate cluster containing most term deliveries following TPL.

**Conclusion**: Cord blood metabolomic profile is altered in fetuses exposed to TPL, regardless of whether delivery occurs preterm or at term. These findings suggest that TPL may have lasting metabolic effects, underscoring the need for further research into its impact on fetal metabolic health.

**10:30 AM – 12:00 PM**

## 278 | **Mode of Planned Delivery in Advanced Maternal Age**

Lital Shaham^1^; Shiran Bookstein^2^; Michal Fishel Bartal^3^; Michal Axelrod^4^

^1^Department of Obstetrics and Gynecology, Sheba Medical Center, Sheba Medical Center, HaMerkaz; ^2^Sheba Medical Center, Ramat Gan, Israel, Ramat Gan, HaMerkaz; ^3^Division of Maternal‐Fetal Medicine, Department of Obstetrics, Gynecology and Reproductive Sciences, Sheba Medical Center, Tel Aviv University, Israel, Ramat Gan, HaMerkaz; ^4^Sheba Medical Center, Sheba Medical Center, HaMerkaz

**Objective**: To compare maternal and neonatal outcomes between induction of labor (IOL) and planned cesarean delivery (CD) among nulliparous individuals of advanced maternal age (≥40 years).

**Study Design**: We conducted a retrospective cohort study at a single tertiary center (2011–2025), including nulliparous women aged ≥40 years with singleton pregnancies ≥34 weeks who underwent IOL or planned CD. Individuals were excluded if they had a mandatory indication for CD (e.g., placenta previa, vasa previa, malpresentation) or experienced spontaneous labor onset or rupture of membranes. Primary outcomes included composite maternal adverse outcomes (CMAO: postpartum fever, hemorrhage, blood transfusion, ICU admission, prolonged hospitalization, or readmission) and composite neonatal adverse outcomes (CNAO: low Apgar, cord pH <7.1, neonatal intensive care unit (NICU) admission, RDS, subgaleal hematoma, or TTN).

**Results**: Of 653 eligible individuals, 359 (55.0%) underwent planned CD and 294 (45.0%) underwent IOL. Among those induced, 130 (44.2%) had an unplanned CD. Age >45 years (aOR 2.37, 95% CI 1.04–5.39, *p* = 0.040) and severe preeclampsia (aOR 3.43, 95% CI 1.05–11.28, *p* = 0.042) were independently associated with unplanned CD. CMAO occurred more often with IOL (19.4% vs. 9.5%, *p* < 0.001), including higher rates of postpartum fever (5.8% vs. 0.6%, *p* < 0.001), blood transfusion (5.1% vs. 0.8%, *p* = 0.001), and longer hospitalization (3.5 vs. 0.9 days, *p* < 0.001). CNAO rates were similar after adjusting for gestational age (aOR 1.19, 95% CI 0.63–2.26, *p* = 0.591), although Small for gestational age (31.3% vs. 16.4%, *p* < 0.001) and NICU admission (1.5% vs. 5.2%, *p* = 0.01) differed.

**Conclusion**: Nearly half of nulliparous ≥40 undergoing IOL required unplanned CD. Age >45 and severe preeclampsia were independent predictors. IOL was associated with higher maternal morbidity compared to planned CD. These findings support individualized counseling and a risk‐stratified approach to delivery planning in advanced maternal age.

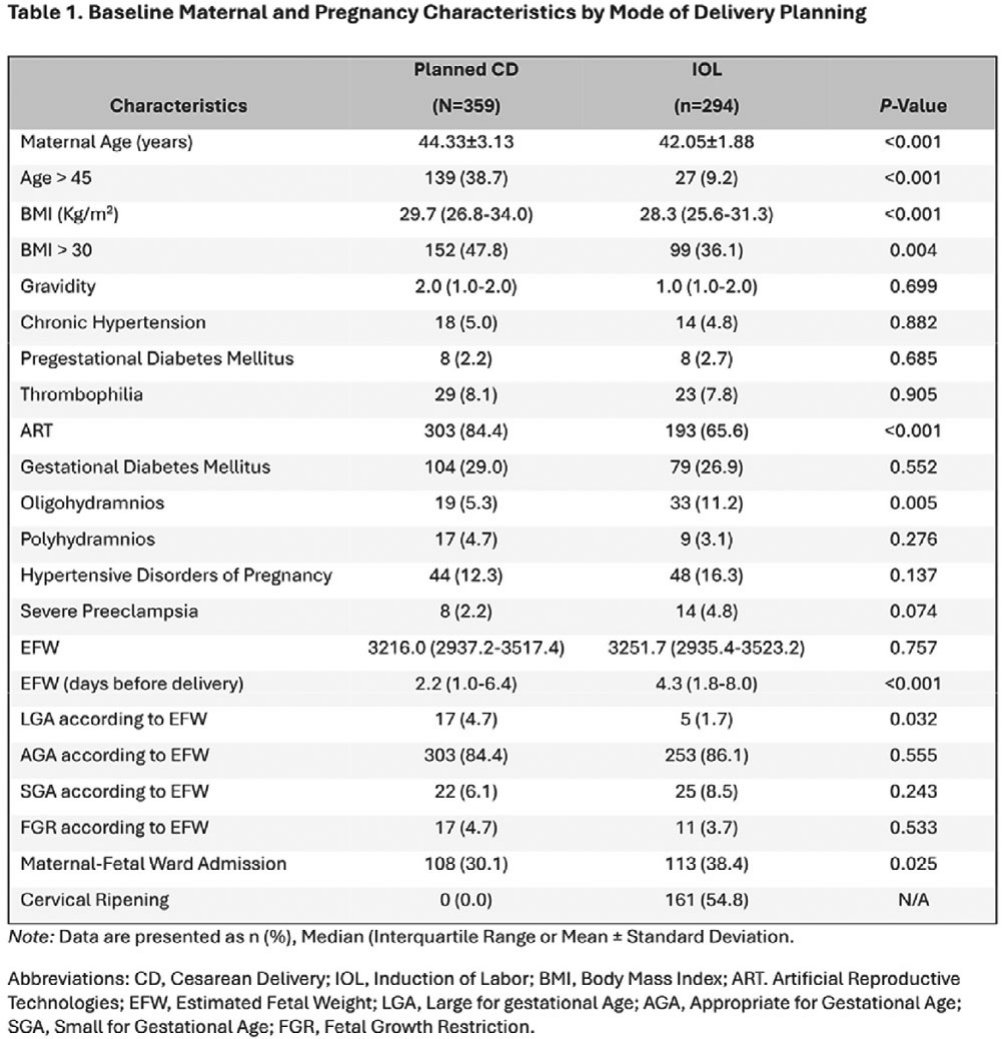

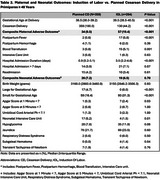

**10:30 AM – 12:00 PM**

## 279 | Nativity and Regional Anesthesia Use in Vaginal Deliveries: Comparing United States and Foreign‐Born Individuals

Lucy Qi^1^; Leshae A. Cenac^2^; Rodney A. McLaren, Jr^3^; Huda B. Al‐Kouatly^4^

^1^Thomas Jefferson University College of Population Health, Philadelphia, PA, USA, Philadephia, PA; ^2^Division of Maternal‐Fetal Medicine, Department of Obstetrics and Gynecology, Sidney Kimmel Medical College at Thomas Jefferson University, Philadelphia, PA, USA, Philadephia, PA; ^3^Maimonides Medical Center, Brooklyn, NY; ^4^Division of Maternal‐Fetal Medicine, Department of Obstetrics and Gynecology, Sidney Kimmel Medical College at Thomas Jefferson University, Philadelphia, PA, USA, Philadelphia, PA

**Objective**: Epidural anesthesia is widely used for labor pain management in the United States. While prior research shows disparities by race and socioeconomic status, there is still a significant gap in knowledge of nativity and epidural use in the United States. Thus, the objective of this study was to evaluate differences in regional anesthesia use during vaginal deliveries between United States‐born and foreign‐born individuals.

**Study Design**: A population‐based retrospective analysis was conducted on births with singleton vaginal deliveries using data from the National Vital Statistics from 2016 to 2023. Births by cesarean delivery, multifetal gestations, <23 or >42 weeks, congenital anomalies, or missing variables were excluded. The primary outcome was regional anesthesia use. Multivariable logistic regression assessed the association between nativity status and regional anesthesia use, adjusting for demographic and clinical factors.

**Results**: Of the 17,653,560 included births, 3,878,084 (22.0%) were from foreign‐born individuals and 13,775,476 (78.0%) were from United States‐born individuals. Births from foreign‐born individuals had significant differences in age, insurance, and education compared to United States‐born individuals, Table 1. After adjusting for differences in multiple sociodemographic and clinical factors, regional anesthesia use was more prevalent among United States‐born compared to foreign‐born individuals (75.0% vs. 68.1%; aOR: 0.86, 95% CI 0.86‐0.87), Table 2.

**Conclusion**: Foreign‐born individuals were significantly less likely to receive regional anesthesia during vaginal delivery in the United States, even after adjusting for multiple sociodemographic and clinical factors. These findings highlight the importance of addressing nativity status as a distinct and under‐recognized factor in maternal pain management disparities. Future research is needed on interventions addressing communication barriers, cultural beliefs, and informed decision‐making on regional anesthesia use.

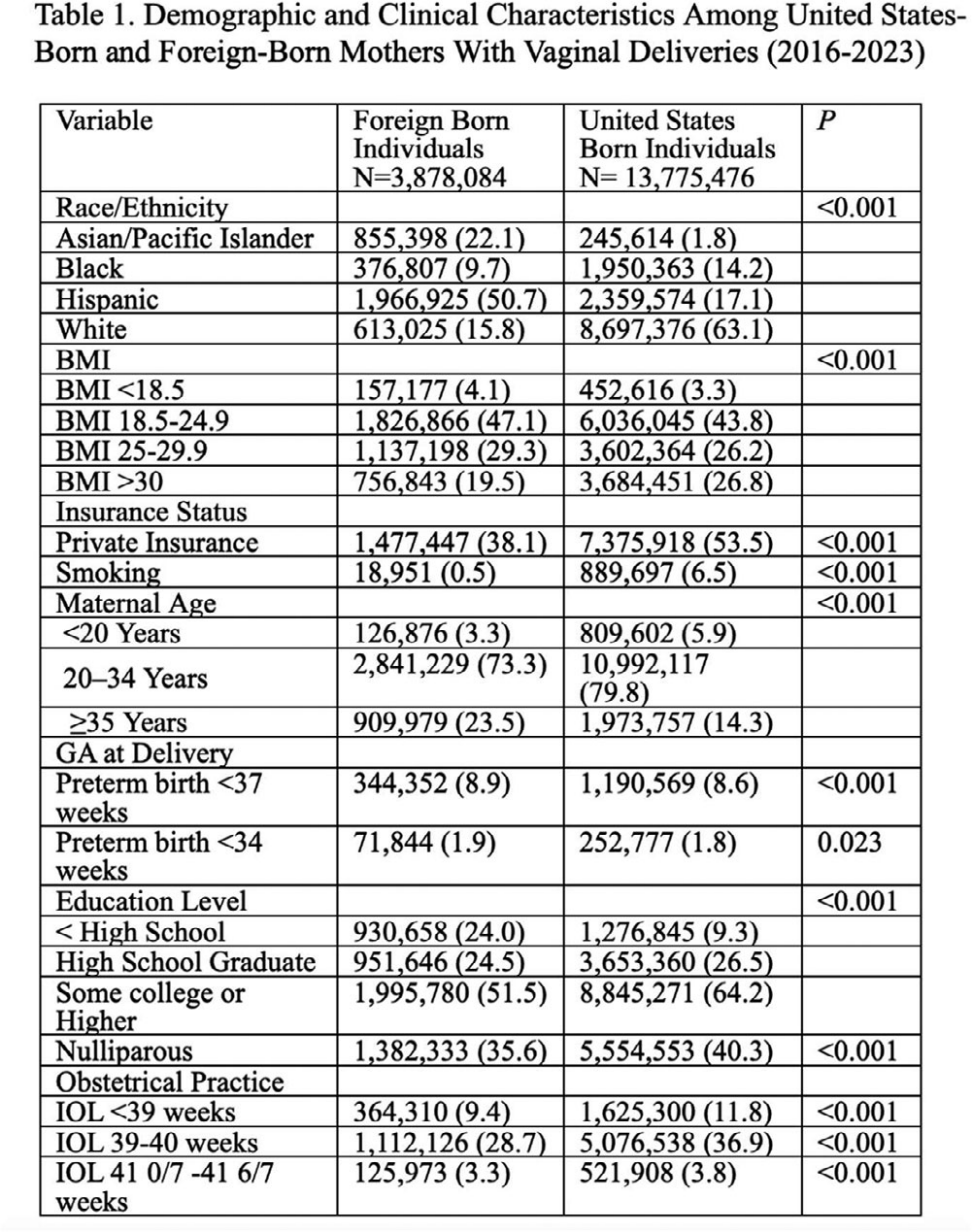

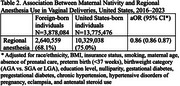

**10:30 AM – 12:00 PM**

## 280 | Association of Health Literacy With Patient‐Reported Outcomes in the Perinatal Period Among Low‐Income Individuals

Lynn M. Yee^1^; Sydney L. Raucher^1^; Ying Cheung^1^; Jecca R. Steinberg^2^; Brittney R. Williams^1^; Joseph M. Feinglass^1^; Ka'Derricka Davis^1^; Michelle A. Kominiarek^1^; William A. Grobman^3^

^1^Northwestern University Feinberg School of Medicine, Chicago, IL; ^2^UCSF, San Francisco, CA; ^3^Brown University, providence, RI

**Objective**: Inadequate health literacy (HL) is associated with suboptimal perinatal health, yet its association with patient experiences is not known. We aimed to evaluate whether HL is associated with patient‐reported outcomes (PROs) assessing perceived health status, healthcare engagement, and well‐being in the third trimester and first year postpartum.

**Study Design**: This is a secondary analysis of data from a randomized trial of postpartum patient navigation, in which pregnant people were eligible if they were ≥16 years, English‐ or Spanish‐speaking, and had public insurance. Upon enrollment (≥30 weeks’ gestation through postpartum hospitalization), participants completed the Newest Vital Sign, a validated measure of HL, which was categorized as inadequate versus adequate based on standard scoring. PRO measures, which reflected multiple aspects of health, were collected at enrollment, 4–12 weeks postpartum, and 11–13 months postpartum. The significance of differences in PRO scores by HL adequacy was determined using bivariable tests; multivariable models adjusted for maternal age and education.

**Results**: Of 405 participants randomized, 59.3% (*N* = 240) had inadequate HL. Those with inadequate HL were younger and less likely to have a college degree than those with adequate HL. At enrollment, in adjusted analysis, those with inadequate HL reported lower global health status (mental health component) and patient activation, and greater perceived stress (Table 1). At 4–12 weeks postpartum, participants with inadequate HL reported lower global health status (physical health component), patient activation, self‐efficacy, and postpartum care satisfaction; they also reported higher perceived stress (Table 2). At 11–13 months postpartum, participants with inadequate HL continued to report lower patient activation (Table 2).

**Conclusion**: Among low‐income pregnant participants in a prospective study, inadequate HL was common and associated with several indicators of suboptimal perinatal health and healthcare engagement. HL may be an understudied area for interventions to optimize maternal health.

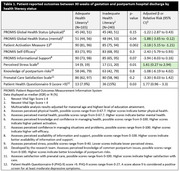

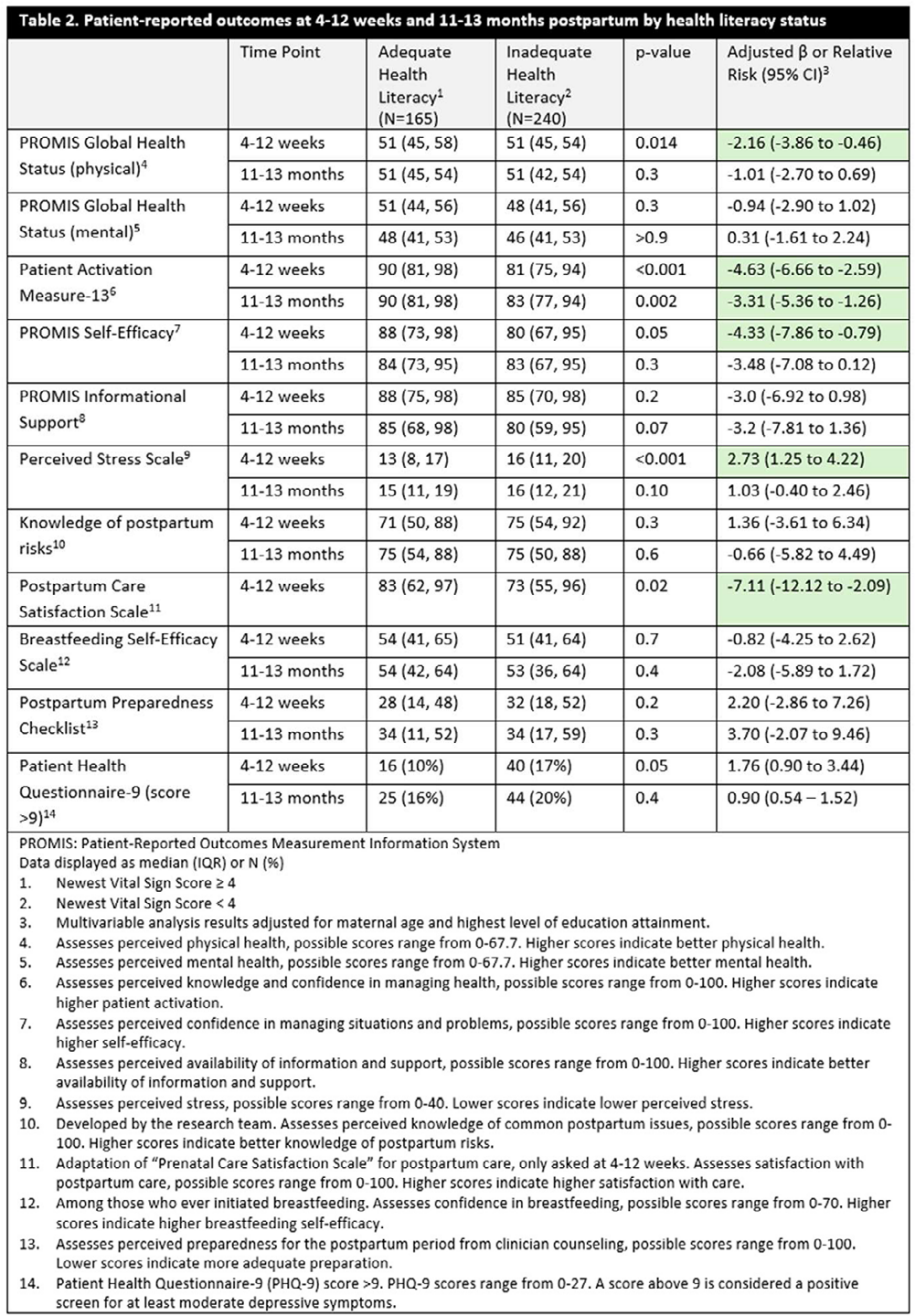

**10:30 AM – 12:00 PM**

## 281 | Use of Multiple Substances in a Pregnant Population With Substance Use Disorder

Macy Afsari^1^; Allison Kurzeja^1^; Mary Ann Faucher^2^; Polly B. Cordova^3^; Jessica McNeil^3^; Amber Fisher^3^; Stephanie Morillos^3^; Joshua Kern^1^; Nancy S. Onisko^1^; Aldo Andino^1^; Kurt Kleinschmidt^1^; April R. Gorman^1^; Emily H. Adhikari^1^

^1^University of Texas Southwestern Medical Center, Dallas, TX; ^2^Parkland Health, Lubbock, TX; ^3^Parkland Health, Dallas, TX

**Objective**: Use of multiple substances significantly increases the risk for overdose‐related death. Data on trends in substance use combinations among pregnant individuals is limited. The objective of this study was to describe patterns of use of multiple substances in a pregnant population in an urban setting.

**Study Design**: This was a retrospective cohort study of pregnant patients with moderate to severe substance use disorder (SUD) who received care at a safety‐net hospital. Substance use patterns were obtained via medical record review, including patient‐reported use patterns and urine toxicology screens. A correlation matrix was generated to describe strength of association for each pair of substances with R‐factor >|0.7| considered strong association.

**Results**: From July 28, 2021 through June 25, 2022, 184 patients with SUD were identified. Eighty‐five (46%) patients identified as Hispanic, 65(35%) identified as Non‐Hispanic White, 32(17%) as Non‐Hispanic Black, and 2(1%) as Asian. Of these, 177(64%) attended prenatal care. Forty‐five (24%) patients reported use of only one substance. Of those with use of only one substance, 17(38%) used opiates, 13(29%) used alcohol, 10(22%) used methamphetamine, 3(7%) used prescription stimulants, and 2(4%) used cocaine. Among the remaining 139(76%) patients with use of multiple substances, there was a strong negative correlation between use of stimulants and cocaine together (R‐factor −0.99) and benzodiazepines and hallucinogens together (R‐factor −0.93). There were moderate positive correlations between use of hallucinogens and cocaine (R‐factor 0.54), stimulants and hallucinogens (R‐factor 0.46), and benzodiazepines and stimulants (R‐factor 0.42).

**Conclusion**: This study provides valuable insight into the complex patterns of substance use in pregnant patients with known SUD, with a majority engaging in use of multiple substances. These findings reflect the need for ongoing comprehensive screening and interventions to address multiple substance use in this population. Further research is essential to validate these patterns and link them to maternal and neonatal outcomes.

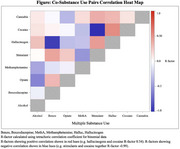

**10:30 AM – 12:00 PM**

## 282 | Adverse Pregnancy Outcomes in a Medicaid Population Lacking Pre‐Pregnancy Claims History

Madeline F. Perry^1^; Lena Bertozzi^2^; Ian J. Hooley^2^; Isabelle Von Kohorn^2^; Eva M. Luo^2^

^1^Hospital of the University of Pennsylvania, Philadelphia, PA; ^2^Pomelo Care, Pomelo Care, NY

**Objective**: Adverse pregnancy outcomes (APOs) are increasing in the US and disparities exist. Management of chronic conditions is critical to reducing the risk of APOs, but people insured by Medicaid experience higher rates of uninsurance and insurance churn, limiting access to care. We used complete maternity episode data to compare outcomes for nulliparous individuals with and without a pre‐pregnancy Medicaid claims history.

**Study Design**: This cross‐sectional analysis used Medicaid claims for nulliparous, Medicaid insured individuals in GA and TX who received care from a virtual medical practice from 2023–2025. We compared patients with no Medicaid claims in the three years prior to pregnancy to those with claims. Outcomes included CS, preterm birth, gestational DM, hypertensive disorders of pregnancy (HDP), severe maternal morbidity, and NICU admission. Rates of chronic conditions before the first prenatal visit (PNV) and at birth were compared. We used multivariate logistic regression to assess the association between a lack of pre‐pregnancy claims and adverse outcomes, controlling for sociodemographic and medical factors.

**Results**: Of the 4219 participants, 60% (*n* = 2490) had no Medicaid claims in the 3 years preceding pregnancy. People with no claims pre‐pregnancy had fewer chronic conditions before the first PNV (claims between start of pregnancy and first PNV), fewer chronic conditions at the time of birth, and higher percent change in chronic conditions identified between the two time points (Table 1). Participants with no pre‐pregnancy claims were 24% more likely to have a CS (aOR 1.24, 95% CI 1.07, 1.43) and 42% more likely to develop HDP (aOR 1.42, 95% CI 1.21, 1.66) than people with claims pre‐pregnancy (Table 2).

**Conclusion**: Even though most participants had no claims pre‐pregnancy, many were diagnosed with chronic conditions that likely pre‐dated pregnancy. We found an association between no pre‐pregnancy claims and certain APOs. Evaluation and intervention for chronic conditions before and early in pregnancy may provide an opportunity to reduce the risk for APOs.

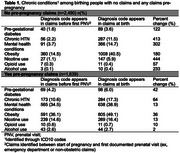

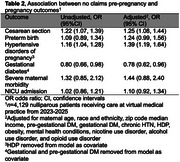

**10:30 AM – 12:00 PM**

## 283 | Pre‐Pregnancy GLP‐1 Exposure and Interval Development of Gestational Diabetes During Pregnancy

Maggie Ross^1^; Harsheen Chawla^2^; Andrea Neira Gesteira^3^; Mariella Gastanaduy^4^; Meena Mishra^5^; Sarah Olsen^6^; Frank B. Williams^1^

^1^Ochsner Clinic Foundation, Ochsner Clinic Foundation, LA; ^2^Ochsner Clinic Foundation, New Olreans, LA; ^3^Ochsner, ochsner/New Orleans, LA; ^4^Ochsner Center for Outcomes Research, New Orleans, LA; ^5^Ochsner Center of Outcomes & Health Services Research, New Orleans, LA; ^6^University of Queensland, University of Queensland, LA

**Objective**: One in 8 US adults have used GLP‐1 receptor agonists, with at least half using specifically for weight loss. Preconception GLP‐1 use and its impact on pregnancy remains to be well described. We aim to investigate whether preconception use of GLP‐1 receptor agonists is associated with an increased risk of developing gestational diabetes (GDM).

**Study Design**: We performed a retrospective cohort study of singleton pregnancies receiving prenatal care and delivering at a large regional health system from 2022–2024. Those with documented pregestational diabetes, prior bariatric surgery and multifetal pregnancies were excluded. Those with documented GLP‐1 use in the 12 months prior to pregnancy were considered exposed, while those without were considered controls. The primary outcome was diagnosis of GDM. Baseline characteristics were compared with chi square or Mann Whitney U tests. Regression analysis controlling for age and baseline body mass index (BMI) generated adjusted odds ratios with 95% confidence intervals.

**Results**: Of 24,403 eligible pregnancies, 165 were exposed to GLP‐1 receptor agonists prior to pregnancy, while 24,238 had no documented exposure. Patients exposed to GLP‐1 receptor agonists had higher age, BMI and chronic hypertension, while controls were more likely to be Black and to use Medicaid (Table). GDM occurred in 22.4% of the GLP‐1 receptor agonists group compared to 9.1% in the control group (aOR 2.6, 95% CI 1.8–3.8, Figure).

**Conclusion**: GLP‐1 receptor agonist exposure prior to pregnancy was associated with an increased odds for GDM when controlling for age and BMI.

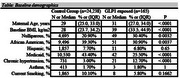

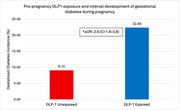

**10:30 AM – 12:00 PM**

## 284 | Angle of Progression Velocity as a Sonographic Marker for Operative Delivery Due to Labor Arrest

Mai Shiber^1^; Yossi Geron^2^; Nati Bor^3^; Miriam Lopian^2^; Yinon Gilboa^2^; Eran Hadar^4^; Natav Hendin^5^

^1^Helen Schneider Hospital for Women, Rabin Medical Center, Rabin Medical Center–Beilinson Hospital, HaMerkaz; ^2^Helen Schneider Hospital for Women, Rabin Medical Center, Petah Tikva, HaMerkaz; ^3^Helen Schneider Hospital for Women, Rabin Medical Center, Petah Tikva, Israel, Petah Tikva, HaMerkaz; ^4^Rabin Medical Center, Petach Tikva, HaMerkaz; ^5^Rabin Medical Center, Petah Tikva, HaMerkaz

**Objective**: To evaluate whether the rate of Angle of Progression (AoP) advancement, measured via serial transperineal ultrasound during the second stage of labor, is associated with mode of delivery–specifically the risk of operative vaginal or cesarean delivery due to prolonged second stage (PSS) or arrest of descent compared to spontaneous vaginal delivery.

**Study Design**: This retrospective cohort study included 154 women who delivered at a single tertiary center (May 2023 ‐ July 2025) and underwent ≥ 2 AoP measurements during the second stage of labor. Patients were categorized by mode of delivery: spontaneous vaginal delivery (*n* = 85) vs. operative delivery (vacuum or cesarean) due to prolonged second stage or arrest of descent (*n* = 69). Cases of operative delivery for non‐reassuring fetal heart rate were excluded. AoP velocity (°/min) was calculated and compared between groups. Multivariate logistic regression assessed associations between sonographic measures and mode of delivery, adjusted for parity, fetal head position, station, and time from full dilation to first AoP.

**Results**: Operative delivery was associated with significantly lower AoP velocity (0.18 ± 0.15 vs. 0.27 ± 0.28 °/min, *p* < 0.05). In adjusted multivariable analysis, faster AoP velocity remained independently associated with reduced odds of operative delivery (adjusted OR 0.08; 95% CI 0.01–0.79; *p* < 0.05). Neonatal outcomes, including NICU admission, cord pH and low Apgar (<7 at 5 minutes), did not differ significantly between groups. The operative delivery group had higher postpartum hemorrhage (22.9% vs. 5.6%; *p* < 0.05) and longer postpartum stay (4.24 vs. 3.65 days; *p* =< 0.05).

**Conclusion**: Slower AoP velocity is a strong marker for operative delivery due to labor arrest. In contrast, faster progression of AoP during second‐stage labor is independently associated with spontaneous vaginal delivery. Serial AoP assessment may provide a real‐time, objective tool to support intrapartum decision‐making and anticipate abnormal labor progression.

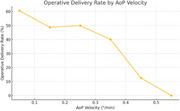

**10:30 AM – 12:00 PM**

## 285 | Preeclampsia in Monochorionic Twins With Selective Fetal Growth Restriction

Amelia Jiang‐Yu^1^; Christina Rivera^2^; Laura S. Tan^3^; Camille Shantz^4^; Jena L. Miller^5^; Michelle Kush^6^; Ahmet A. Baschat^7^; Mara Rosner^5^

^1^Johns Hopkins University, Johns Hopkins University/Baltimore, MD; ^2^Johns Hopkins University, Silver Spring, MD; ^3^Johns Hopkins University, Baltimore, MD; ^4^Boston University, Boston, MA; ^5^Johns Hopkins Center for Fetal Therapy, Johns Hopkins University/Baltimore, MD; ^6^Johns Hopkins Center for Fetal Therapy, Johns Hopkins University/BAltimore, MD; ^7^Johns Hopkins University, Johns Hopkins University, MD

**Objective**: Selective fetal growth restriction (sFGR) in monochorionic twin (MC) pregnancies is considered a disorder of unequal placental share, whereas preeclampsia (PEC) is a disorder of placental dysfunction. We sought to understand the frequency and characteristics of PEC occurring in the setting of MC twins with sFGR.

**Study Design**: Retrospective study of MC pregnancies with sFGR from 6/2014–4/2025. Higher order gestations, Twin to Twin Transfusion at presentation, severe anomalies, and cases that were lost to follow up were excluded. Presentation, course, and outcome characteristics were compared between patients with and without PEC. Data analysis was performed using Pearson Chi‐Squared, Fisher's Exact, or Mann‐Whitney U.

**Results**: 33/197 (16.8%) patients meeting criteria with sFGR developed PEC during their pregnancy. There was no difference in baseline maternal characteristics including nulliparity, overweight or obesity, prior PEC history, or chronic hypertension (cHTN). However, PEC was more common in patients with Type I sFGR (21%) and less common in patients with Type III sFGR (5.8%, *p* = 0.046). There was no difference in aspirin (ASA) utilization or Doppler course in the growth restricted fetus between groups. Unplanned and obstetrically indicated deliveries were more frequent in patients with PEC. Survival outcomes were not different between groups.

**Conclusion**: PEC complicated approximately 1 in 6 MC twins with sFGR. PEC in sFGR appeared to be independent of traditional risk factors including cHTN, PEC history, maternal weight category, parity, and ASA course. Ultimately our findings support that pregnancies complicated by sFGR require close maternal and fetal surveillance. The relationship of PEC with sFGR Type requires further study.

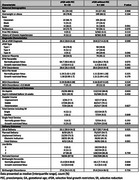

**10:30 AM – 12:00 PM**

## 286 | 1‐Year Neonatal Survival in Extremely Preterm and Extremely Low Birth Weight Infants

Marcia Des Jardin^1^; Tao Chen^2^; Colm P. Travers^3^; Akila Subramaniam^1^; Vivek V. Shukla^4^; Waldemar A. Carlo^4^

^1^Center for Research in Women's Health, University of Alabama at Birmingham, Birmingham, AL; ^2^Department of Pediatrics, University of Alabama at Birmingham, University of Alabama at Birmingham, AL; ^3^Department of Pediatrics, University of Alabama at Birmingham Heersink School of Medicine, Birmingham, AL; ^4^Department of Pediatrics, University of Alabama at Birmingham, Birmingham, AL

**Objective**: Counseling and decision‐making in the periviable period are often based on rates of adverse neonatal outcomes at a given gestational age (GA) and/or birthweight. Viability thresholds have been lowered but remain inconsistent between institutions. We aimed to characterize infant one‐year mortality rates by GA and birthweight among extremely preterm and extremely low birth weight infants, to help provide counseling guidance.

**Study Design**: We performed a retrospective cohort study of all infants born at 22 0/7–27 6/7 weeks GA and birthweight <1000 g who delivered at a single center from 1998 to 2023. We excluded infants who died within 12 hours of birth without active postnatal treatment to reduce bias in mortality rates from restriction of care. Infant one‐year mortality rates were categorized by completed weeks of gestation and by birthweight into predefined 100‐gram (g) ranges (Table). Receiver‐operator curves (ROC) with associated area under the curve (AUC) were used to characterize ability to predict 1‐year survival.

**Results**: 3221 infants met study criteria and were analyzed. One infant resuscitated at ≤300 g (*n* = 1) did not survive. Infants born ≤500 g had ≥50% chance of mortality at all extremely preterm GA categories; mortality decreased with increasing birthweight and gestational age. Among infants at 301–400 g and 401–500 g, the mortality rate was high across all GA (range 67–100% for 301–400 g, 56–77% for 401–500 g), with reduction by GA only noted in the 401–500 g group. At 24 weeks and after, infants >500 grams had ≤50% mortality. Birthweight alone (AUC = 0.75) and GA alone (AUC = 0.72) had similar but only fair prediction for survival at 1 year, but when combined, prediction was excellent (AUC = 0.86).

**Conclusion**: Infants born ≤500 g had ≥50% chance of mortality at all extremely preterm GA categories; whereas mortality decreased as both GA and birthweight increased among these infants. Neither birthweight nor GA alone predict 1‐year survival; but both together resulted in excellent prediction and should be taken into consideration when counseling patients on obstetric and neonatal interventions.

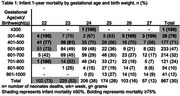

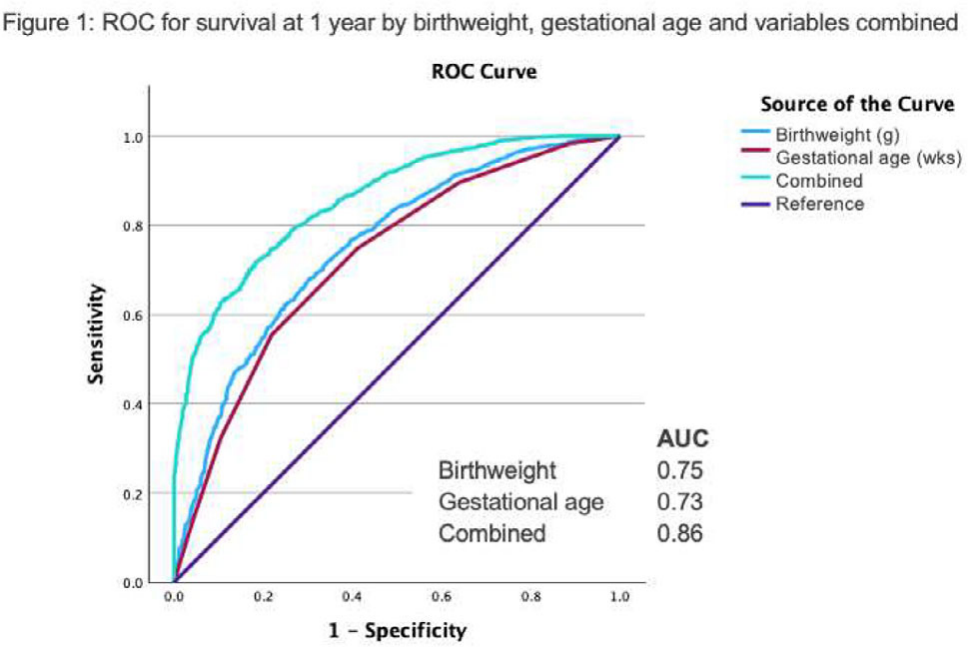

**10:30 AM – 12:00 PM**

## 287 | Hypertensive Disorders of Pregnancy and Long‐Term Risk of Dilated Cardiomyopathy: A Systematic Review and Meta‐Analysis

Maria Lemos^1^; Ayesha Ayesha^2^; Paweł Łajczak^3^; Laura Queiroz^4^; Yami Bhaskar^5^

^1^Kurah Women's Health Institute, Montverde, FL; ^2^Shifa College of Medicine, Islamabad, Islamabad; ^3^Medical University of Silesia, Katowice, Slaskie; ^4^Santa Casa de Misericórdia de Belo Horizonte, Belo Horizonte, Minas Gerais; ^5^Vydehi Institute of Medical Sciences and Research Centre, Bangalore, Karnataka

**Objective**: Hypertensive disorders of pregnancy (HDP), including gestational hypertension and preeclampsia, are associated with increased long‐term cardiovascular disease. While peripartum cardiomyopathy is well‐recognized, the link between HDP and subsequent dilated cardiomyopathy (DCM) beyond the peripartum period remains unclear. This study aims to quantify the long‐term risk of DCM following HDP and compare risks across HDP subtypes.

**Study Design**: A comprehensive literature search was conducted across PubMed, Cochrane Library, Embase and Web of science, to identify studies published up to July 2025. Studies were included if they compared individuals with hypertensive disorders of pregnancy to normotensive controls in the assessment of cardiomyopathy outcomes, including dilated cardiomyopathy. Data was analyzed using a random‐effects model and heterogeneity through I^2^ statistics on R software.

**Results**: The analysis included three studies with a total of 5.119 patients, of whom 564 (11%) had hypertensive disorders of pregnancy. In the pooled analysis, patients with hypertensive disorders showed a significantly higher incidence of dilated cardiomyopathy compared to normotensive patients (OR 2.42; 95% CI 1.9 to 3.06; *p* < 0.001; I^2^ = 0%; Figure 1). When evaluating overall cardiomyopathies, the incidence was also significantly higher among those with hypertensive disorders (OR 1.99; 95% CI 1.65 to 2.41; *p* < 0.001; I^2^ = 72.8%; Figure 2).

**Conclusion**: Hypertensive disorders of pregnancy are significantly associated with long‐term risk of DCM. This underscores the need for postpartum cardiovascular monitoring and targeted prevention strategies. A history of HDP should be routinely incorporated into cardiovascular risk stratification to guide future cardiac care.

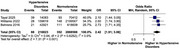

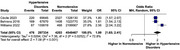

**10:30 AM – 12:00 PM**

## 288 | Continuous Cuffless Blood Pressure Monitoring: A Novel Approach to Blood Pressure Monitoring in Pregnancy

Mariam K. Ayyash^1^; Shai Bejerano^1^; Helen B. Gomez Slagle^1^; Matthew K. Hoffman^2^; Russell S. Miller^3^; Uma M. Reddy^1^

^1^Columbia University Irving Medical Center, New York, NY; ^2^Christiana Care Health System, Newark, DE; ^3^Division of Maternal‐Fetal Medicine, Department of Obstetrics and Gynecology, Columbia University Irving Medical Center, New York, NY

**Objective**: Novel continuous cuffless blood pressure monitoring (CCBPM) devices have recently emerged as an alternative for traditional cuff‐based non‐invasive BP monitoring, but their performance in pregnancy remains unknown. Hilo (Aktiia SA, Neuchâtel, Switzerland) is a CCBPM band that estimates BP using photoplethysmography signals collected with optical sensors at the wrist. This pilot study evaluated the agreement between Hilo band BP readings and standard at‐home BP cuff measurements across varying gestational ages.

**Study Design**: We conducted a prospective observational pilot study of 12 low‐risk pregnant and postpartum patients (3 per trimester and postpartum). This represents off‐label use of the Hilo band, and Aktiia granted authorization for publication. Participants wore the Hilo band for 7 days while also performing 21 standard cuff measurements (3 per day). CCBPM readings within 30–60 minutes of cuff readings were paired. Daily average systolic and diastolic BP values between the two devices were compared using linear mixed‐effects models. Agreement was assessed by calculating the proportion of paired measurements within 5, 10, and 15 mmHg.

**Results**: A total of 74 average daily BP readings were obtained, including 145 paired measurements within 30 minutes and 180 within 60 minutes of cuff readings. The mean age of participants was 31.2 ± 4.1 years, and the mean body mass index (BMI) was 29.2 ± 7.3 kg/m^2^. Fewer than 50% of systolic and diastolic readings fell within 5 mmHg; 60–70% were within 10 mmHg, and 80–85% within 15 mmHg. There was no significant difference in daily average systolic BP between devices (4.09 mmHg difference, *p* = 0.10). Daily average diastolic BP was 5.3 mmHg lower with Hilo (*p* = 0.02). Adjusting for BMI dichotomized at 30 kg/m^2^ did not alter these findings.

**Conclusion**: This limited dataset provides a novel assessment of a CCBPM device in pregnancy. While preliminary findings are encouraging, cuff‐based devices should remain the standard of care until larger studies confirm the accuracy and reliability of cuffless technologies.

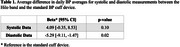

**10:30 AM – 12:00 PM**

## 289 | Implementation of a Standardized Protocol for Intrapartum Diabetes Management

Marissa A. Hand^1^; Adina R. Kern‐Goldberger^2^; Megan R. Ansbro^1^; Hannah E. Bowers^1^; Stacey Ehrenberg^2^; Cara D. Dolin^3^

^1^Cleveland Clinic Foundation, Cleveland Clinic Foundation, Cleveland, OH; ^2^Cleveland Clinic Foundation, Cleveland Clinic Foundation, OH; ^3^Cleveland Clinic, Clevelend, OH

**Objective**: To determine if implementation of a standard protocol for intrapartum diabetes management is associated with improved maternal and neonatal outcomes.

**Study Design**: This retrospective cohort study included patients with diabetes in pregnancy who underwent a trial of labor from 1/1/24–3/31/25. In July 2024, a standardized intrapartum diabetes management protocol was implemented, providing structured guidance for blood glucose monitoring and medical management in patients with pregestational and gestational diabetes, with a target intrapartum glucose of 70–120 mg/dL. Before this, management was individualized. Outcomes were compared before (1/1/24–6/30/24) and after (10/1/24–3/31/25) protocol implementation, excluding a 3‐month transition. The primary outcome was neonatal hypoglycemia (<50 mg/dL) in the first 24 hours. Secondary neonatal outcomes included NICU admission and hyperbilirubinemia. Maternal outcomes included intrapartum hyperglycemia and insulin treatment. Bivariable analysis compared demographic and clinical characteristics, and a negative binomial model assessed changes in the primary outcome using an interrupted time series analysis.

**Results**: There were no significant differences in neonatal outcomes between the pre‐(*n* = 483) and post‐implementation (*N* = 518) cohorts: neonatal hypoglycemia (54.2% vs 53.3%, *p* = 0.76), hyperbilirubinemia (7.2% vs 8.9%, *p* = 0.34), NICU admission (17% vs 14.7%, *p* = 0.32) (Table 1). Patient characteristics and delivery outcomes were similar between cohorts. Rates of pregestational diabetes (21.9% vs 21.6%, *p* = 0.90) and insulin use (53.0% vs 54.8%, *p* = 0.56) were similar, however initial HbA1c was lower post‐implementation (5.7 vs 5.4, *p* < 0.01). After implementation, the number of intrapartum glucose checks per patient increased (IRR 1.17, 95% CI 1.12–1.22, *p* < 0.01) (Figure 1), as did the maximum intrapartum glucose value (115 vs 130, *p* < 0.01).

**Conclusion**: Implementation of a standardized intrapartum diabetes protocol led to more frequent glucose monitoring and higher recorded maternal glucose values, without worsening neonatal outcomes.

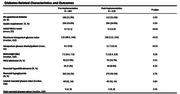

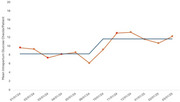

**10:30 AM – 12:00 PM**

## 290 | Use and Out‐Of‐Pocket Costs of Antenatal Fetal Surveillance for Patients With Chronic Conditions

Rebecca A. Gourevitch^1^; Amanda Speller^2^; Anna D. Sinaiko^3^; Mark A. Clapp^4^; Jessica L. Cohen^3^

^1^University of Maryland, College Park, MD; ^2^Harvard University, Boston, MA; ^3^Harvard. T.H. Chan School of Public Health, Boston, MA; ^4^Department of Obstetrics and Gynecology, Mass General Brigham, Massachusetts General Hospital, MA

**Objective**: To measure variation in use and out‐of‐pocket costs for antenatal fetal surveillance (AFS) among commercially insured patients with chronic hypertension or pre‐gestational diabetes.

**Study Design**: This was a cross‐sectional analysis of pregnancies of at least 20‐weeks’ gestation between 2017–2022. We used administrative enrollment and health insurance claims data from the Health Care Cost Institute, which includes commercially insured pregnancies nationwide. Our sample included patients with chronic hypertension or pre‐gestational diabetes. Our outcomes were the number of days with AFS testing during pregnancy, the out‐of‐pocket costs for those tests, and those costs as a proportion of all out‐of‐pocket costs during pregnancy. We use linear regression models to compare variation in these outcomes by health plan type, patient factors (age, clinical characteristics), and geographic factors (rurality, maternity care access, and area‐level income and race distributions).

**Results**: Our sample included over 150,000 pregnancies with chronic hypertension or pre‐gestational diabetes. On average, patients with chronic hypertension received 5.1 AFS tests and those with pre‐gestational diabetes received 6.5. There was significant variation in the number of AFS received, with 15.9% of patients with chronic hypertension and 12.3% of those with pre‐gestational diabetes receiving no AFS tests during pregnancy. Receiving no AFS was most common for patients in areas that were rural, low‐income, or had a higher concentration of Black individuals. One‐quarter of patients with chronic conditions faced out‐of‐pocket costs for AFS of $264 or more, or $301 or more for a quarter of patients with pre‐gestational diabetes. Patients in POS or PPO plans, in rural areas, and in areas with a higher concentration of white people had higher costs.

**Conclusion**: AFS is the primary tool for stillbirth prevention, yet we found wide variation in AFS use and costs in this commercially‐insured population of high‐risk pregnancies–including many patients who receive no AFS.

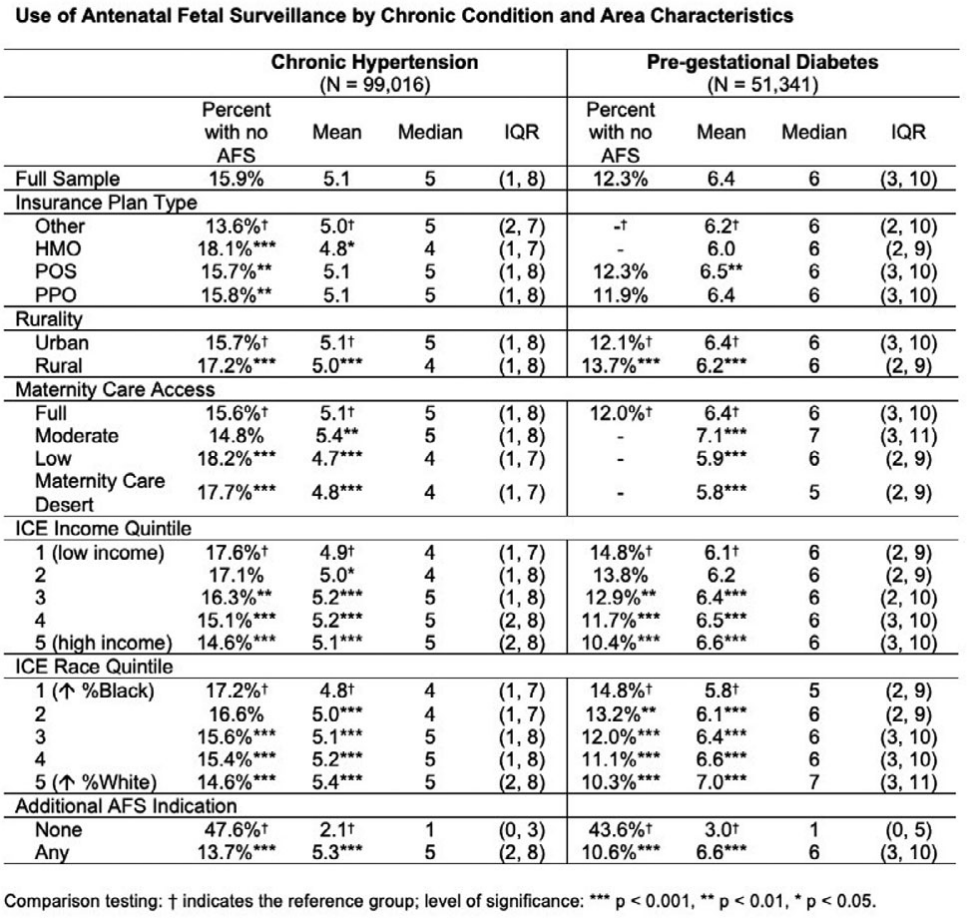

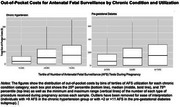

**10:30 AM – 12:00 PM**

## 291 | Late and Post‐Term Birth and Endocrine Morbidity in Offspring: A Population‐Based Study

Mark Volevich^1^; Gil Gutvirtz^2^; Gali Pariente^3^; Tamar Wainstock^4^; Eyal Sheiner^5^

^1^Department of Obstetrics and Gynecology, Soroka University Medical Center, Beer sheva, HaDarom; ^2^Department of Obstetrics and Gynecology, Soroka University Medical Center, Ben‐Gurion University, Metar, HaDarom; ^3^Department of Obstetrics and Gynecology, Soroka University Medical Center, Ben‐Gurion University, beer sheva, HaDarom; ^4^Department of Epidemiology, Biostatistics and Community Health Sciences, Faculty of Health Sciences, Ben‐Gurion University of the Negev, Beer Sheva, HaDarom; ^5^Soroka University Medical Center, Faculty of Health Sciences, Ben‐Gurion University of the Negev, beer sheva, HaDarom

**Objective**: At term, the fetal endocrine system is well‐developed and capable of supporting the transition to neonatal life, as most hormonal axes are active and functionally mature. However, post‐term birth (>42 weeks’ gestation) has been linked to various physiological stressors, and its association with long‐term endocrine outcomes is unclear. We aimed to evaluate a possible association between gestational age at delivery and the risk of offspring long‐term endocrine morbidity.

**Study Design**: We conducted a retrospective, population‐based study including all singleton births between 1991–2021 at a tertiary medical center. Offspring were stratified by gestational age into: full term (39.0–40.6 weeks), late term (41.0–41.6 weeks), and post‐term (≥42.0 weeks). Long‐term endocrine morbidity was identified through both inpatient and outpatient records. Cumulative incidence across gestational age groups was assessed using Kaplan‐Meier survival curves. A multivariable Cox regression model was used to evaluate the independent association between gestational age and endocrine morbidity, adjusting for maternal and gestational age, gestational diabetes, hypertensive disorders, and mode of delivery.

**Results**: A total of 103,797 singleton births beyond 39 weeks were included. The overall rate of endocrine morbidity was comparable between full‐term, late‐term and post‐term offspring (Table). However, the cumulative incidence of endocrine morbidity over time was lower for offspring born late‐ and post‐term (Figure), and the adjusted analysis found that late‐term birth was associated with a decreased risk of endocrine morbidity (HR 0.79; 95% CI 0.69–0.89; *p* < 0.001), while post‐term birth did not differ significantly from full‐term (HR 0.77; 95% CI 0.57–1.05; *p* = 0.101).

**Conclusion**: Late‐term birth appears to confer a modest protective effect against long‐term endocrine morbidity in offspring, but this effect was not apparent for post‐term births. Additional studies are needed to better elucidate whether prolongation of pregnancy offers any significant advantage to the offspring.

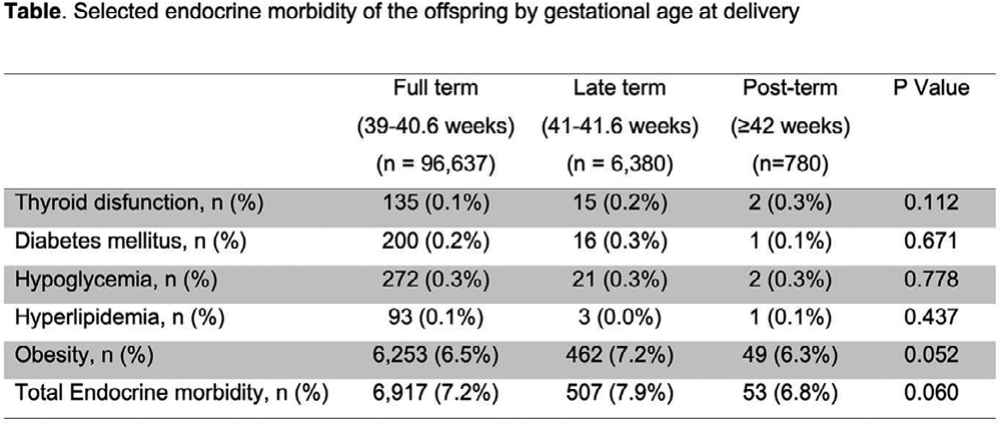

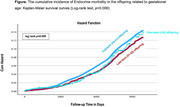

**10:30 AM – 12:00 PM**

## 292 | Baseline Mental Health Risk Stratification in Group Prenatal Care: A Comparative Analysis

Mary E. Peeler^1^; EleVATE Collaborative^2^; Nia Plump^2^; Julia Muller^3^; Traci Johnson^4^; Cheron Phillips^5^; Richelle Smith^5^; Kelly McKay‐Gist^5^; Rachel Paul^6^; Annie Dude^3^; Jeannie C. Kelly^7^; Nandini Raghuraman^8^; Melissa Tepe^9^; Candice L. Woolfolk^7^; Shannon Lenze^10^; Ebony Carter^11^

^1^UNC Chapel Hill, UNC Chapel Hill, NC; ^2^EleVATE Collaborative, St. Louis Integrated Health Network, St. Louis, MO; ^3^University of North Carolina, Chapel Hill, NC; ^4^University of Missouri‐Kansas City, Kansas City, MO; ^5^St. Louis Integrated Health Network, St. Louis, MO; ^6^Washington University School of Medicine, Washinton University in St. Louis School of Medicine, MO; ^7^Washington University School of Medicine, Washington University School of Medicine, MO; ^8^Washington University School of Medicine, St. Louis, MO; ^9^Affinia Healthcare, St. Louis, MO; ^10^Washington University in St. Louis, School of Medicine, Department of Psychiatry, St. Louis, MO; ^11^University of North Carolina, University of North Carolina, NC

**Objective**: Recent trials of group prenatal care (GPC) models show mixed or negative results for key perinatal outcomes, but high‐risk subgroups may preferentially benefit. This study aims to characterize baseline mental health profiles across high‐risk subgroups enrolled in a GPC trial to inform future risk‐stratified analyses of intervention effectiveness.

**Study Design**: Participants enrolled in a randomized trial of a GPC intervention embedded with evidence‐based behavioral health techniques had baseline mental health profiles collected using validated assessment tools: Edinburgh Postnatal Depression Scale, Perceived Stress Scale, PROMIS Anxiety scale, and PTSD Checklist. Adolescents (age <20) and participants with a history of prior preterm birth (PTB) were identified as high‐risk for adverse perinatal outcomes, and their profiles were compared with adults and those without a PTB history using crude odds ratios (ORs) and 95% confidence intervals (CIs).

**Results**: Of 307 enrolled participants, 14.7% were adolescents and 8.0% had a prior PTB. Baseline demographics were similar between adolescents and participants with a prior PTB compared to the rest of the sample. Adolescents demonstrated distinct mental health profiles with significantly higher rates of positive screening for depression (42% adolescents vs. 27% adults; OR 1.99; 95% CI 1.02–3.89) and perceived stress (74% adolescents vs. 57% adults; OR 2.19; 95% CI 1.05–4.56), but no differences were seen in trauma or anxiety between age groups. Women with prior PTB showed similar baseline mental health profiles to those without a history across all screening measures.

**Conclusion**: Adolescents had significantly higher rates of baseline depression and stress compared to adults, while those with PTB showed no mental health differences. Our findings suggest that adolescents may be optimal candidates for behaviorally integrated GPC interventions. Future stratified outcome analyses will determine whether baseline mental health profiles predict differential responses, potentially explaining heterogeneous trial results and informing precision prenatal care approaches.

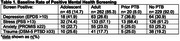

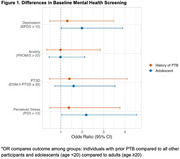

**10:30 AM – 12:00 PM**

## 293 | Development and Validation of a Screening Tool to Predict Severe Neonatal Morbidity

Matthew J. Blitz; Frank I. Jackson; Ji Yoon Lee; Dawnette Lewis; Adriann Combs; Michael Nimaroff; Burton Rochelson; Annemarie Stroustrup

Northwell, New Hyde Park, NY

**Objective**: To develop and validate a clinical screening tool to predict severe neonatal morbidity (SNM) at both labor floor admission and delivery using routinely available maternal, fetal, social, and intrapartum factors.

**Study Design**: This retrospective cohort study included singleton deliveries at ≥32 weeks of gestation at two tertiary hospitals in New York (2019–2023). Patients were randomly assigned to training (70%) and testing (30%) cohorts. SNM was defined using a composite of diagnoses and procedures indicating life‐threatening neonatal complications. Six multivariable logistic regression models were developed using stepwise selection. Maternal variables were derived from obstetric comorbidity index (OB‐CMI) components. Fetal factors included fetal size abnormalities, amniotic fluid abnormalities, major structural anomalies, and abnormal umbilical artery Doppler measurements. Additional models incorporated social and intrapartum variables. Models were compared using AUC and Brier score; final models were validated on the testing cohort.

**Results**: Among 53,159 deliveries, 2554 (4.8%) newborns experienced SNM. Models based on maternal or fetal factors alone had moderate discrimination (AUCs 0.72 and 0.68, respectively). Combining maternal and fetal variables improved discrimination (AUC 0.73); adding social factors yielded the best model at admission (AUC 0.74, Brier 0.057). Gestational age, major fetal anomaly, and polyhydramnios were the strongest predictors available at admission. After delivery, cesarean birth and chorioamnionitis were highly predictive, and a final model incorporating intrapartum variables achieved the best performance (AUC 0.76). All models demonstrated good calibration and consistent validation.

**Conclusion**: We developed and validated a practical tool for SNM prediction at maternal admission and delivery. Incorporating defined fetal characteristics alongside maternal and social factors improved predictive performance and may support early risk stratification and neonatal care planning.

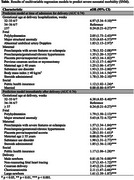

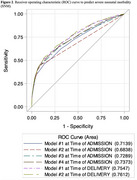

**10:30 AM – 12:00 PM**

## 294 | Association Between Very Elevated BMI and Cesarean Delivery in the United States

Maya Nitecki^1^; Osinakachukwu Mbata^2^; Dana Hazimeh^1^; Lillian Boettcher^3^; Emma Peek^4^; Sara I. Jones^5^; Hannah Kelly^5^; Amanda M. Craig^5^; Jerome J. Federspiel^5^

^1^Duke University Medical Center, Duke University Medical Center, NC; ^2^Duke University Health, Durham, NC; ^3^Department of Obstetrics and Gynecology, Duke University School of Medicine, Duke University School of Medicine/Durham, NC; ^4^Duke University School of Medicine, Duke University Medical Center, NC; ^5^Duke University School of Medicine, Durham, NC

**Objective**: Elevated body mass index (BMI) is an established risk factor for cesarean delivery (CD), but the implications of BMI elevation beyond 40 kg/m^2^ on CD risk are poorly described. Our aim was to describe the proportion of CD in the United States across a broad spectrum of BMI, focusing on people with BMI greater than 40.

**Study Design**: This retrospective cohort study utilized the Epic Cosmos dataset. Pregnancies were included if they occurred between July 2018 and June 2025, had an available delivery summary, pre‐pregnancy BMI was between 18.5 and 80 kg/m2, and were without history of prior CD. BMI was categorized as 18.5–24.9, 25.0–29.9, 30.0–39.9, 40.0–49.9, 50.0–59.9, 60.0–69.9, and 70.0–79.9. The proportion of pregnancies resulting in CD and the 95% confidence interval (CI) were calculated for each BMI category. Results were stratified by parity; and among nulliparous patients, additional stratification by gestational diabetes (GDM), pre‐gestational diabetes (DM), and chronic hypertension (cHTN) according to the International Classification of Diseases codes was performed.

**Results**: A total of 7,324,281 pregnancies were included, of which 19.5% resulted in CD. The probability of CD increased from 15.5% with BMI 18.5–24.9 to 49.2% for BMI 70.0–79.9 in a monotonic fashion (Figure 1A). Parity strongly impacted the probability of CD, with nulliparous patients at greater risk of CD (Figure 1B). Among nulliparous patients, GDM, pregestational DM, and cHTN were all associated with higher rates of CD across the range of BMI (Figures 2A–2B).

**Conclusion**: Among pregnancies without history of CD, there is a monotonic relationship between BMI and CD risk, strongly influenced by parity. Comorbid conditions further increase CD risk. The average nulliparous patient with BMI >50 has >50% probability of CD, a risk that increases further with higher BMI or comorbidities. Studies are warranted to investigate indications for CD among these high‐risk patients and to support individualized counseling regarding CD risk and delivery planning.

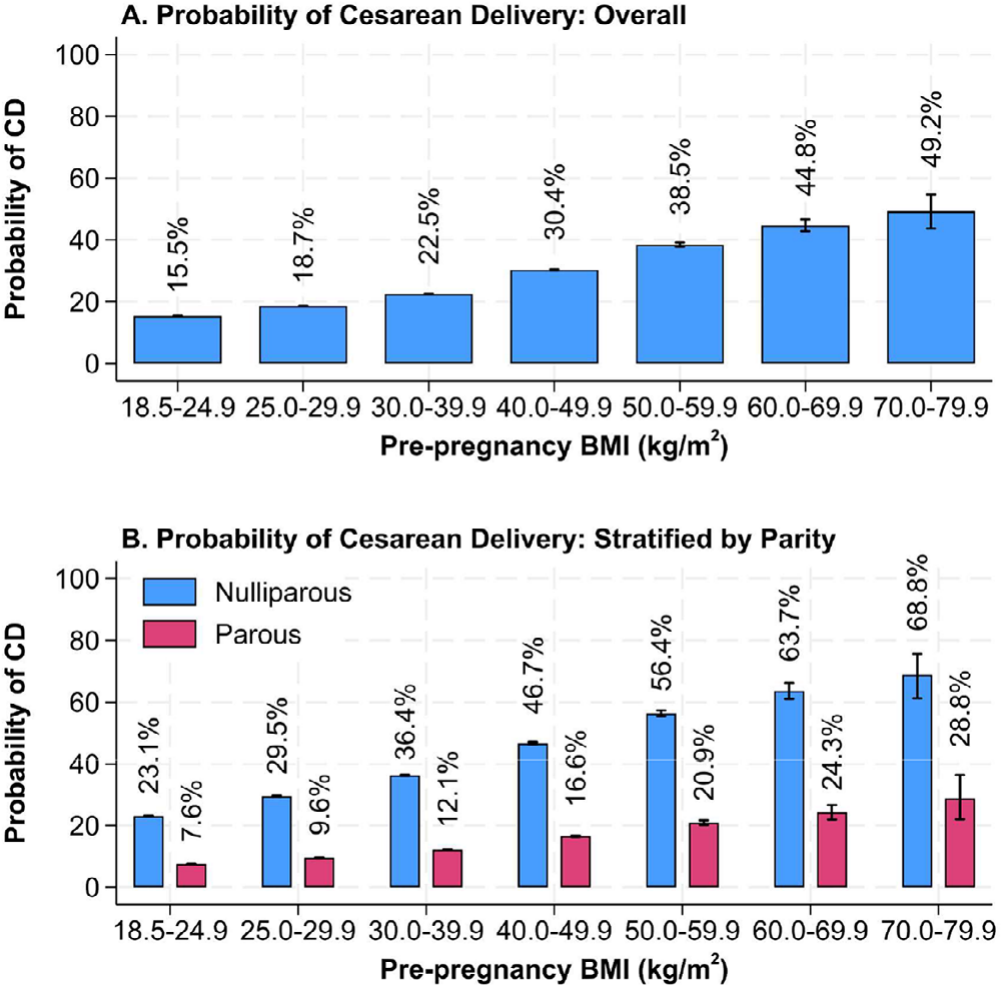

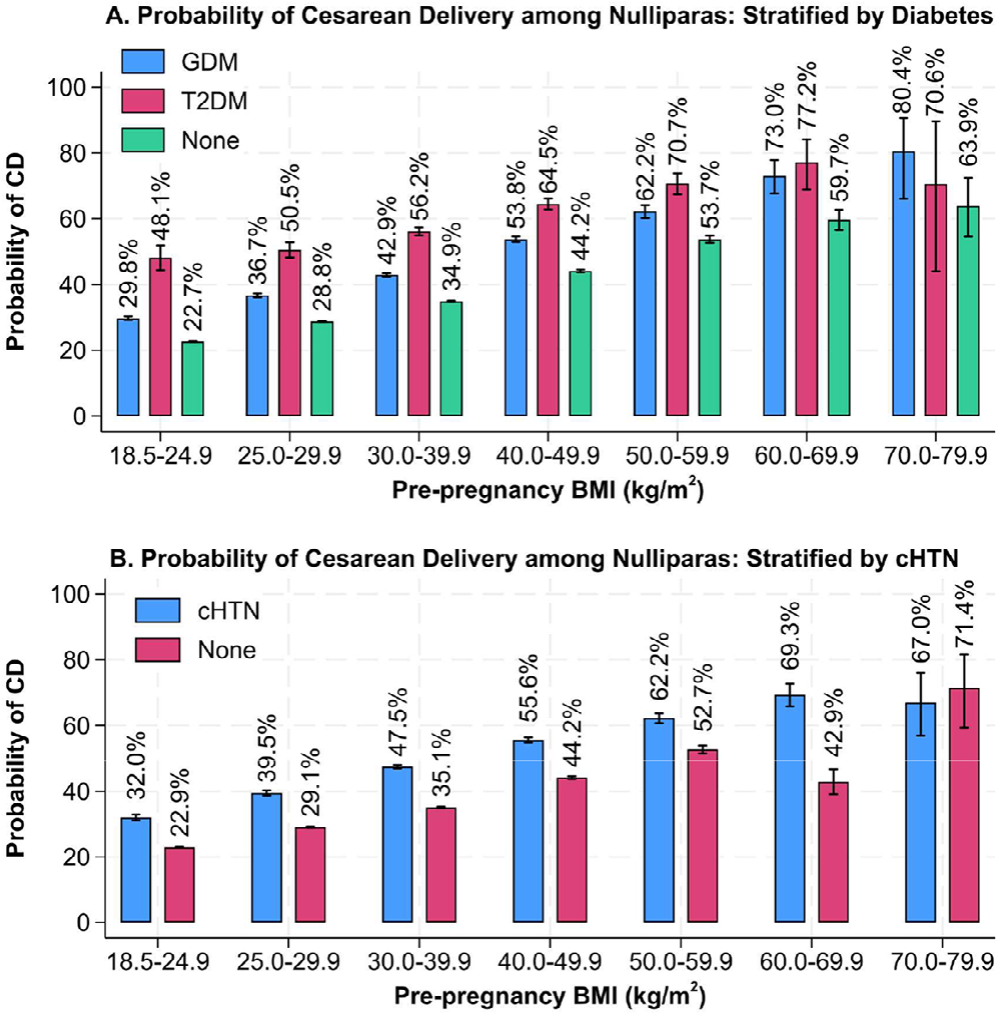

**10:30 AM – 12:00 PM**

## 295 | Quality and Accuracy of Obstetrical Videos Using #Epidural or #Labor on TikTok

McKensie Wall^1^; Minhazur R. Sarker^2^; Isabel Katlaps^1^; Emma Roberts^1^; Rachel L. Wiley^2^; Gladys A. Ramos^3^; Chia‐Ling Nhan‐Chang^3^

^1^Department of Obstetrics, Gynecology and Reproductive Sciences, University of California San Diego, San Diego, CA; ^2^Division of Maternal Fetal Medicine, Department of Obstetrics, Gynecology and Reproductive Sciences, University of California San Diego, San Diego, CA; ^3^Division of Maternal Fetal Medicine, Department of Obstetrics, Gynecology and Reproductive Sciences, San Diego, CA

**Objective**: Reproductive age individuals often turn to social media for health information. We aimed to evaluate the quality and accuracy of TikTok content using #epidural or #labor.

**Study Design**: We conducted a cross‐sectional analysis of TikTok content using #epidural and #labor. The most popular videos for each hashtag were queried using the Apify TikTok Data Extractor scraper tool. Our primary outcome was the number of relevant videos for each hashtag. The secondary outcomes included pre‐specified themes. Each video was assigned a Global Quality Score (GQS) ‐ a validated tool rating medical content from 1 (low, use discouraged) to 5 (high, medically useful). If the content made medical claims, the mDISCERN score for each video was calculated–a validated scale to assess the accuracy of medical information.

**Results**: Among the 68 #epidural and 81 #labor videos, 40 (58.8%) and 43 (53.1%) videos were relevant, respectively. The majority of videos were created by women and by individuals without credentials listed. The majority of content across both hashtags was personal experience with some medical education. The median [interquartile range, IQR] GQS for both #epidural and #labor was 1 [1,1] suggesting that the majority of content was poor quality and not at all medically useful for patients. Of the 9 (22.5%) #epidural videos making medical claims, only 3 (33.3%) were accurate and 6 (66.7%) were inaccurate with a median [IQR] mDISCERN score of 1 [1,1]. No #pitocin videos made medical claims.

**Conclusion**: Our findings suggest that only slightly over half the top videos using #epidural or #labor are relevant to pregnancy. Additionally, the top content for #epidural and #labor on TikTok is over generally low quality and low usefulness to patients.

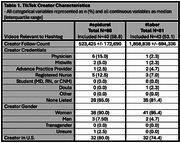

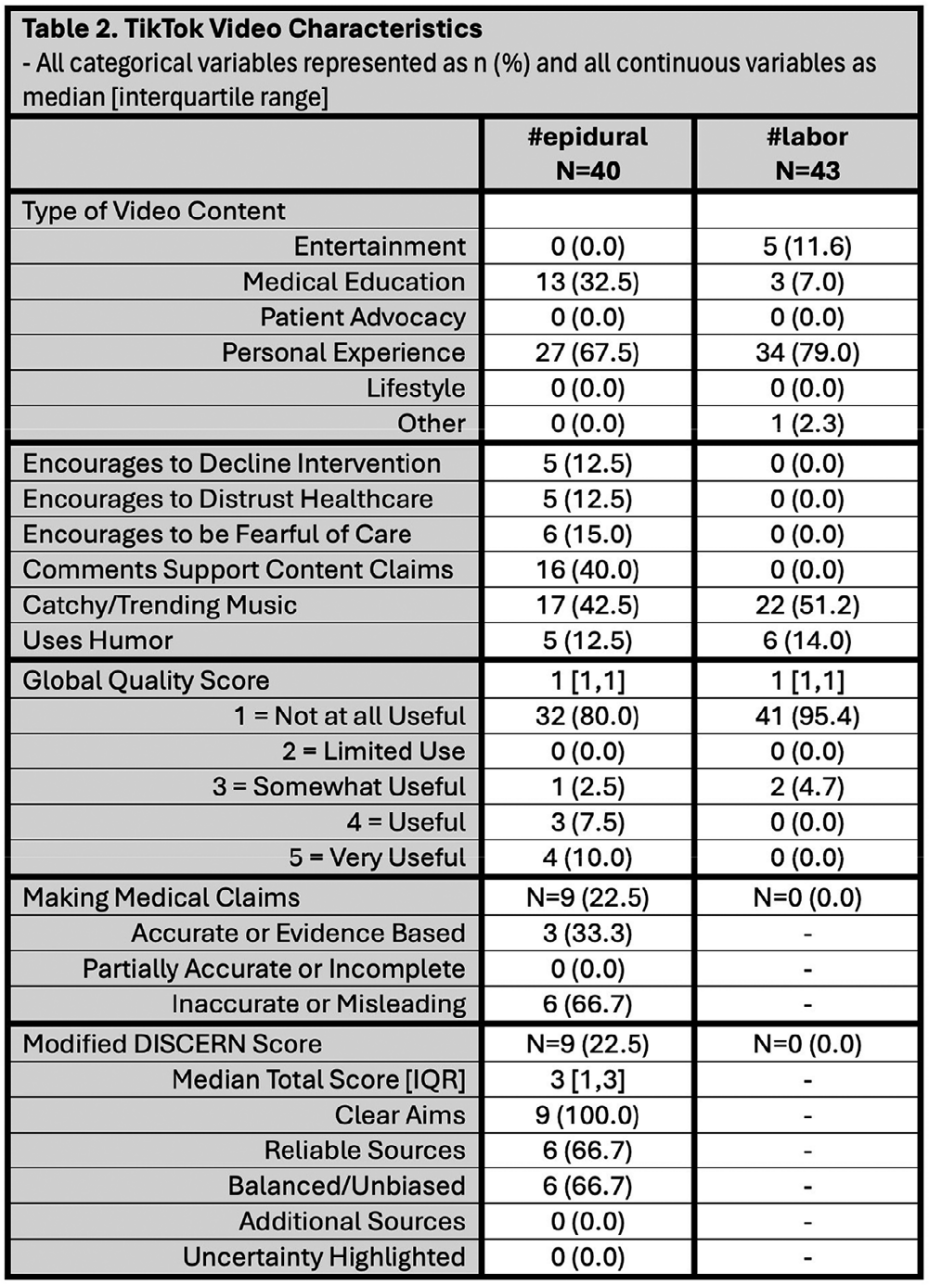

**10:30 AM – 12:00 PM**

## 296 | Missingness as a Social Determinant: Data Gaps Undermine Aspirin Eligibility for Preeclampsia Risk Reduction

Melissa S. Wong^1^; Emily Wong^2^; Tiffani Bright^2^

^1^Cedars‐Sinai Health Sciences University, Los Angeles, CA; ^2^Cedars‐Sinai Medical Center, Los Angeles, CA

**Objective**: Low‐dose aspirin (LDA) is recommended for pregnant persons at elevated risk for preeclampsia. Eligibility depends on availability of clinical and social risk factor data. Income, a moderate risk factor per USPSTF, is frequently missing and may contribute to under identification. We evaluated whether missing income data was associated with differential LDA eligibility across racial and ethnic groups and its impact on preeclampsia outcomes.

**Study Design**: A secondary analysis of the nuMoM2b study, a prospective cohort of nulliparous individuals with singleton pregnancies. We included subjects with available preeclampsia outcome data. USPSTF criteria were applied to determine LDA eligibility, classifying participants eligible if they had ≥1 high‐risk or ≥2 moderate risk factors (all met nulliparity; Black race was excluded to allow subgroup analysis). We quantified how many moderate risk factors were present, whether income data were missing, and how missingness varied by sociodemographic characteristics. Last, we examined how LDA eligibility changed based on the presence of income data.

**Results**: 8775 participants were included. 4729 (54%) met LDA criteria, with 4331 (92%) qualifying through moderate‐risk factors alone. Of these, 3186 (74%) had only one additional moderate‐risk factor beyond nulliparity, most commonly income (Fig 1).

Income data were disproportionately missing among participants who were younger, Black, Hispanic, government‐insured, or had lower education levels and was associated with lower LDA eligibility (Table 1). Among participants who developed preeclampsia but were not identified for prophylaxis, income data were missing in 84% of Black and 67% of Hispanic versus 12% of White individuals (Fig 1).

**Conclusion**: Missingness itself may function as a social determinant of health, undermining equitable identification of patients who qualify for LDA prophylaxis and disproportionately excluding those already at elevated risk. Addressing structural missingness and improving routine collection of social risk factors are essential steps to reduce disparities in preeclampsia prevention.

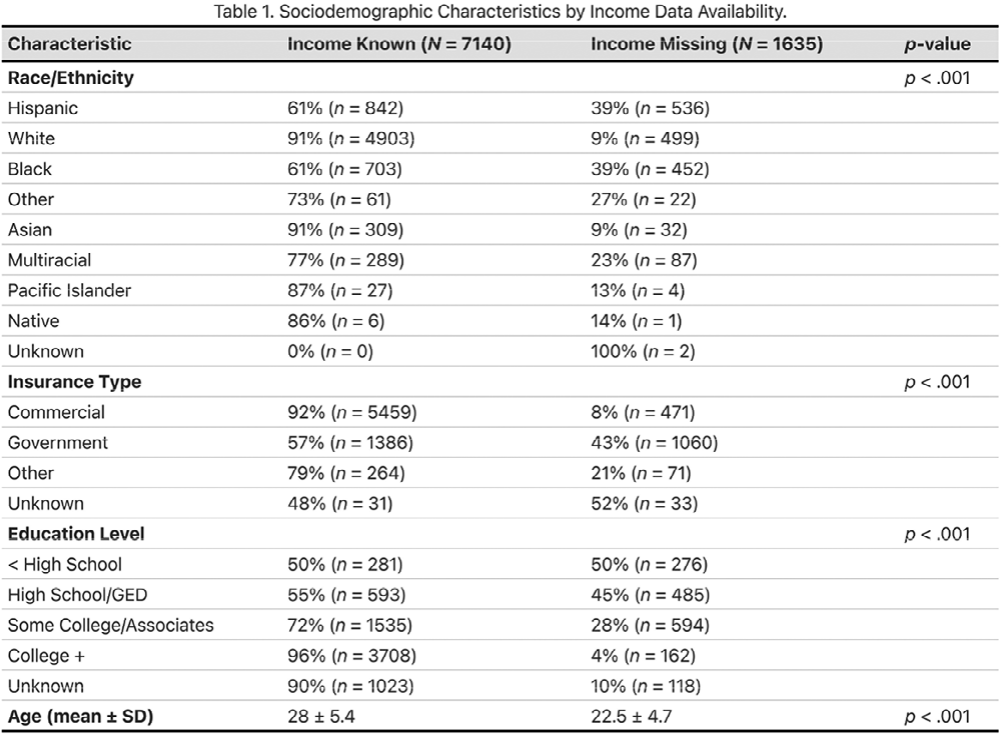

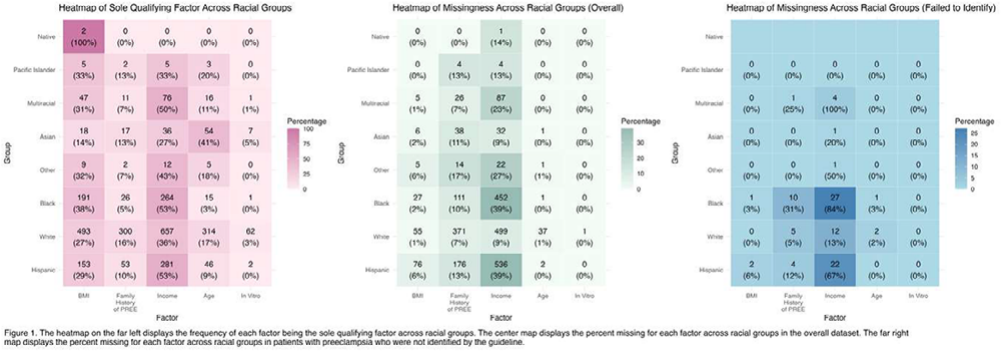

**10:30 AM – 12:00 PM**

## 297 | Levetiracetam Versus Magnesium Sulfate for Seizure Prophylaxis in Pregnancies Complicated by Preeclampsia With Severe Features

Daniel J. Martingano^1^; Melody Boafo^2^; Andrea Rivera^3^; Crystal Awad^4^; Marwah Al‐Dulaimi^5^; Samantha Giler^1^; Jaina Diaz‐Kelly^2^; Kristen Henry^2^; Kelly Mondey^2^; Brenna M. Regan^6^; Kristen Cohen^7^; Iris Gomez‐Brito^8^; Eddie Santana^9^; Angelo Oduro^1^; Jacqueline Marecheau^2^

^1^St. John's Episcopal Hospital‐South Shore / Lake Erie College of Osteopathic Medicine, St. John's Episcopal Hospital‐South Shore / Far Rockaway, NY; ^2^St. John's Episcopal Hospital‐South Shore, St. John's Episcopal Hospital‐South Shore / Far Rockaway, NY; ^3^Wyckoff Heights Medical Center, Wyckoff Heights Medical Center / Brooklyn, NY; ^4^Jamaica Hospital Medical Center, Jamaica Hospital Medical Center / Queens, NY; ^5^St. John's Episcopal Hospital‐South Shore, St. John's Episcopal Hospital‐South Shore, NY; ^6^William Carey University College of Osteopathic Medicine, William Carey University / Hattiesburg, MS; ^7^RWJBarnabas Health Trinitas Regional Medical Center, RWJBarnabas Trinitas Regional Medical Center / Elizabeth, NJ; ^8^RWJBarnabas Trinitas Regional Medical Center, RWJBarnabas Trinitas Regional Medical Center / Elizabeth, NJ; ^9^Good Samaritan University Hospital / NYIT, Good Samaritan University Hospital / West Islip, NY

**Objective**: Intravenous (IV) Levetiracetam (LM) remains a potential alternative to magnesium sulfate (MS) in pregnancies complicated by preeclampsia with severe features (SPEC), yet its comparative efficacy remains undefined. This study sought to evaluate the effectiveness of LM as compared to MS in pregnancies complicated by SPEC.

**Study Design**: We conducted a multi‐center, prospective observational study from 7/2022 to 7/2025 and included all pregnant women diagnosed with SPEC from 34 0/7 to 41 0/7 weeks‐gestation. Patients initially presenting with SPEC in the postpartum period were not included. Both medications were administered IV. Medication choice was based on physician preference. All patients received aspirin 81mg daily. Primary outcomes included progression to eclampsia, events of maternal side‐effects or toxicity (TOX), post‐treatment development of category II fetal heart rate tracings (CatII), as discrete events. Patients with pre‐existing neurologic, renal, hematologic or cardiac disorders, multi‐fetal gestations, or Myasthenia Gravis, were excluded.

**Results**: The study included 444 patients, with 133 receiving LM and 311 receiving MS. Baseline demographic factors were not significantly different. Patients receiving LM had lower rates of TOX (3.7% v. 10.9% *p* < 0.001) and CatII (6.8% v. 19.9%, *p* < 0.001), with an 89% (RR = 0.11, 95% CI 0.66‐0.94, *p* = 0.002) and 79% (RR = 0.29, (95% CI 0.59‐0.87, *p* = 0.003) decreased risk in confounder‐adjusted models, respectively. Both medications did not have significantly different rates or risk of progression to eclampsia

**Conclusion**: Intravenous levetiracetam remains a safe and efficacious alternative to magnesium sulfate for seizure prophylaxis in pregnancies complicated by preeclampsia with severe features, with a potential advantage in preventing maternal and fetal medication‐related side‐effects.

**10:30 AM – 12:00 PM**

## 298 | **Symptom Clusters During Gestation Associated With Risk of Preterm Birth in a Digital Pregnancy Platform**

Mia Charifson^1^; Timothy Wen^2^; Sara M. Sauer^3^; Isabel Fulcher^4^

^1^Delfina Care Inc, Delfina Care Co., CA; ^2^University of California San Diego, San Diego, CA; ^3^Delfina Care Inc, Brooklyn, NY; ^4^Delfina Care Inc, San Francisco, CA

**Objective**: The connection between pregnancy symptoms and adverse pregnancy outcomes has been understudied, largely due to lack of data infrastructure to measure symptoms longitudinally. This research uses longitudinal symptom data from a digital pregnancy care platform to investigate the association between pregnancy symptoms and preterm birth, a common adverse pregnancy outcome.

**Study Design**: Pregnant individuals from four prenatal clinics were enrolled in a mobile application (2022–2025) and were instructed by their care team to track weekly symptoms from a list of 100 symptoms (including ‘No symptoms’). Individuals who tracked symptoms for more than 5 gestational weeks were included in the analysis. Principal component analysis and k‐means clustering identified and classified patients into three distinct clusters of symptom reporting throughout gestation. Multivariate logistic regression was conducted to estimate the association between symptom cluster and preterm birth (delivery <37 weeks gestation), adjusting for primiparity, pre‐pregnancy body mass index, primary language, and prenatal clinic.

**Results**: 718 individuals enrolled in the digital health platform, delivered and reported symptoms for at least 5 gestational weeks. Three symptom clusters were identified (1) few to no symptoms (*n* = 286, 39.8%), (2) common symptoms (e.g. fatigue, back pain) (*n* = 355, 49.4%), (3) neurological/psychological symptoms (e.g. headaches, dizziness, memory changes, stress) (*n* = 77, 10.7%). Adjusting for potential confounders, the neurological/psychological symptom cluster was associated with significantly higher odds of preterm birth (aOR = 2.34 [1.11, 4.81]) compared to the few to no symptoms group. The effect estimate for the common symptom cluster suggested higher odds of preterm birth, albeit with a wide confidence interval (aOR = 1.44 [0.86, 2.48]).

**Conclusion**: This study demonstrated a meaningful association between symptom clusters and preterm birth. Advancements in digital tools to track symptoms throughout pregnancy open new avenues to monitor and potentially identify patients at risk of adverse outcomes.

**10:30 AM – 12:00 PM**

## 299 | **Predicting Success of External Cephalic Version: Machine Learning vs. Traditional Models in a Large Cohort**

Michal Axelrod^1^; Mika Yosef^2^; Maor Sagi^2^; Shlomi Toussia‐Cohen^3^; Shir Koren^2^; Ronit Silber^2^; Chen Berkovitz^2^; Tal Cahan^2^; Orit Moran^2^

^1^Sheba Medical Center, Sheba Medical Center, HaMerkaz; ^2^Sheba Medical Center, Ramat Gan, HaMerkaz; ^3^The Sheba Medical Center, The Sheba Medical Center, HaMerkaz

**Objective**: To develop and compare predictive models for successful external cephalic version (ECV) using traditional statistical methods and modern machine learning (ML) approaches on a large dataset, aiming to improve clinical decision‐making through interpretable artificial intelligence.

**Study Design**: We conducted a retrospective analysis of all ECV procedures performed at a single tertiary center between 2011–2024. The cohort included 1454 cases ‐ one of the largest datasets in this domain ‐ of which 834 (57.3%) were successful. Four clinically relevant predictors were selected: parity, body mass index (BMI), placental location, and amniotic fluid index (AFI). Two predictive models were developed: logistic regression and a tree‐based eXtreme Gradient Boosting (XGBoost) model. The data were randomly divided into training and test sets (80:20). The model performance was assessed using area under the curve (AUC), accuracy, and precision‐recall metrics. SHapley Additive exPlanations (SHAP) were used to evaluate interpretability and to visualize the importance of each feature to individual predictions.

**Results**: The XGBoost model demonstrated superior performance compared to logistic regression. In the test cohort, AUC was 0.72 for XGBoost and 0.69 for logistic regression. It also achieved an accuracy of 0.66 vs. 0.61 and a precision of 0.73 for both models. SHAP analysis revealed that lower BMI and multiparity were associated with a higher likelihood of ECV success. Posterior placenta and higher AFI also contributed favorably to predictions. These findings were consistent with clinical expectations and improved model transparency.

**Conclusion**: Machine learning models, particularly XGBoost, offer improved performance over traditional tools in predicting external cephalic version success. SHAP improved model interpretability and clinicians' understanding and trust. This study, based on one of the largest published ECV cohorts, highlights the value of ML in advancing prediction and personalization in obstetric care.

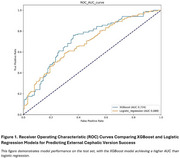

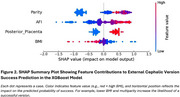

**10:30 AM – 12:00 PM**

## 300 | How do Atherosclerotic Cardiovascular Risk Models Compare When Applied to a High‐Risk Postpartum Population?

Michelle R. Petrich^1^; Andy Chen^2^; Stephanie Shorey^3^; Meredith Meyer^4^; Carly Fesler^5^; Mary Park^5^; Mary McLennan^1^; Niraj R. Chavan^6^

^1^Saint Louis University/SSM Health, Saint Louis University/St. Louis, MO; ^2^SSM Health/St. Louis University School of Medicine, St. Louis, MO; ^3^SSM Health, SSM Health/St. Louis, MO; ^4^St. Mary's Hospital/SSM Health, SSM Health/St. Louis, MO; ^5^Saint Louis University/School of Medicine, Saint Louis University/St. Louis, MO; ^6^Saint Louis University, Saint Louis, MO

**Objective**: We sought to compare performance of atherosclerotic cardiovascular risk prediction models among patients followed at a comprehensive postpartum clinic for cardiometabolic health optimization.

**Study Design**: We conducted a retrospective cohort study of patients who received care at our specialized postpartum center from 9/2024–5/2025. Demographic characteristics, pregnancy and postpartum outcomes, and follow up adherence were abstracted from the electronic records. Cardiometabolic assessments (including BMI, blood pressure, tobacco use, medication history, and lipid and renal profiles) were performed to evaluate and compare 10‐year atherosclerotic cardiovascular risk profile, across 2 distinct prediction models–i.e. American Heart Association's Predicting Risk of Cardiovascular Disease Events (PREVENT) [evaluated with the addition of 2 expansion variables–Social Deprivation Index (SDI) and A1c (in separate models)] and the American College of Cardiology Atherosclerotic Cardiovascular Disease (ASCVD) risk assessment. Two‐sample t‐tests were used to assess differences; significance was set at *p* ≤.05.

**Results**: Of 186 patients followed through our specialized postpartum center, 47 (25%) had a complete metabolic assessment performed. The 10‐year cardiovascular risk profile as evaluated through the PREVENT with Social Deprivation Index (SDI) expansion was 0.59%, PREVENT with A1c expansion was 0.75%, and ASCVD was 1.25% across the subset with completed metabolic assessments. PREVENT with SDI predicted a significantly lower risk than ASCVD for those with hypertensive disorders of pregnancy (*p* = .01), BMI <40 (*p* < .01), and age <40 (*p* = .04) (Table 1). PREVENT with A1c predicted a significantly lower risk than ASCVD for those with hypertensive disorders of pregnancy (*p* < .01), BMI <40 (*p* < .01), and age <40 (*p* < .01) (Table 2).

**Conclusion**: Postpartum cardiometabolic assessments are essential for predicting and appropriately counseling on long term cardiovascular risk. Further research in a larger cohort is critical for developing accurate risk assessment models and optimizing interconception and periconception care.

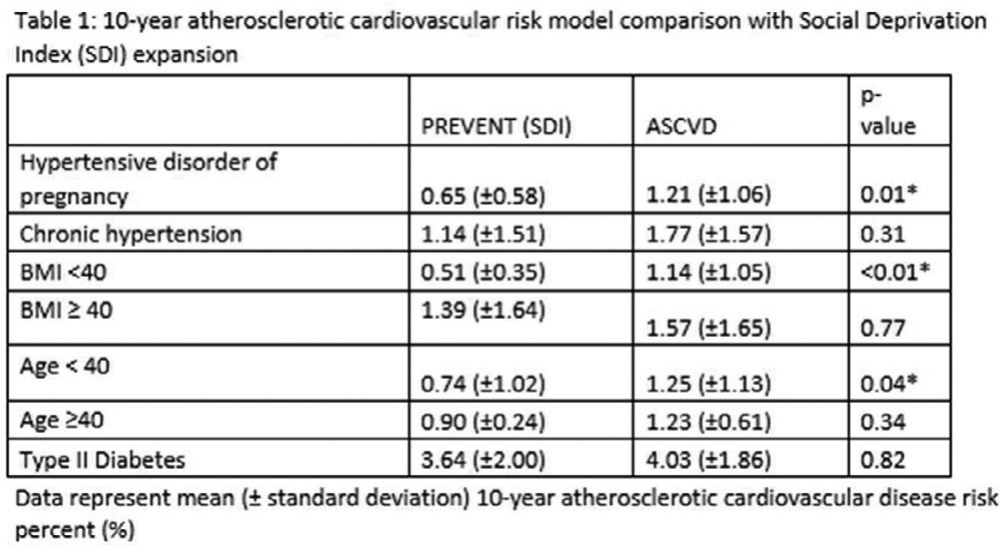

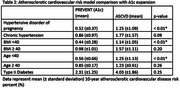

**10:30 AM – 12:00 PM**

## 301 | Forceps‐Assisted Vaginal Delivery Proficiency Among Maternal‐Fetal Medicine Fellow Physicians

Minhazur R. Sarker^1^; Dana R. Canfield^2^; Rachel L. Wiley^1^; Yalda Afshar^3^; Kelsey Rose^3^; Scott Harvey^4^; E. Nicole Nicole Teal^1^

^1^Division of Maternal Fetal Medicine, Department of Obstetrics, Gynecology and Reproductive Sciences, University of California San Diego, San Diego, CA; ^2^Department of Obstetrics & Gynecology  , University of Washington, Seattle, WA, Seattle, WA; ^3^David Geffen School of Medicine at University of California, Los Angeles (UCLA), Los Angeles, CA; ^4^Department of Obstetrics, Gynecology and Reproductive Science, University of California San Diego, San Diego, CA

**Objective**: Obstetricians’ proficiency with operative vaginal delivery (OVD), and particularly forceps‐assisted vaginal delivery (FAVD), has been declining over time. This study aims to characterize FAVD proficiency among maternal‐fetal medicine (MFM) fellow physicians ‐ a group of physicians obtaining additional training in obstetrics.

**Study Design**: We conducted a cross‐sectional survey of MFM fellows from January to June 2025. The survey was distributed primarily by email to fellows, program directors, and program coordinators. The primary outcome was fellow proficiency with FAVD after fellowship. Secondary outcomes included fellow proficiency with vacuum assisted vaginal delivery (VAVD), perceived barriers to OVD training, number of OVD performed, and comfort with not only performing but also teaching OVD. Institutional Review Board exemption was obtained prior to survey distribution.

**Results**: Of the 411 fellows in training at study implementation, 162 (39.4%) responded with no differences in fellowship year or region among responders (Table 1). While 67 (41.4%) entered fellowship with proficiency in FAVD, 110 (67.9%) anticipate comfort with FAVD after training suggesting fellowship continues to train and/or solidify this skillset. During residency, 65.4% of respondents performed 10 or fewer FAVD and only 8.0% performed >20 FAVD while 49.4% of respondents performed 11–20 VAVD and 22.2% performed >20 VAVD (Table 2). In their current level of training, respondents more often felt comfortable teaching VAVD than FAVD (Table 2). The greatest perceived OVD training barriers were low volume, lack of attending proficiency, and too many trainees (Table 1). In the 2^nd^ stage of labor, the preferred mode of delivery among respondents is OVD over cesarean delivery, even in an emergency (Table 2).

**Conclusion**: Less than half of MFM fellows who participated in this study felt comfortable teaching FAVD. While MFM fellowship is expected to improve FAVD proficiency in a quarter of respondents, the declining rate of teaching capacity highlights an impending barrier to maintaining FAVD proficiency in the obstetric community.

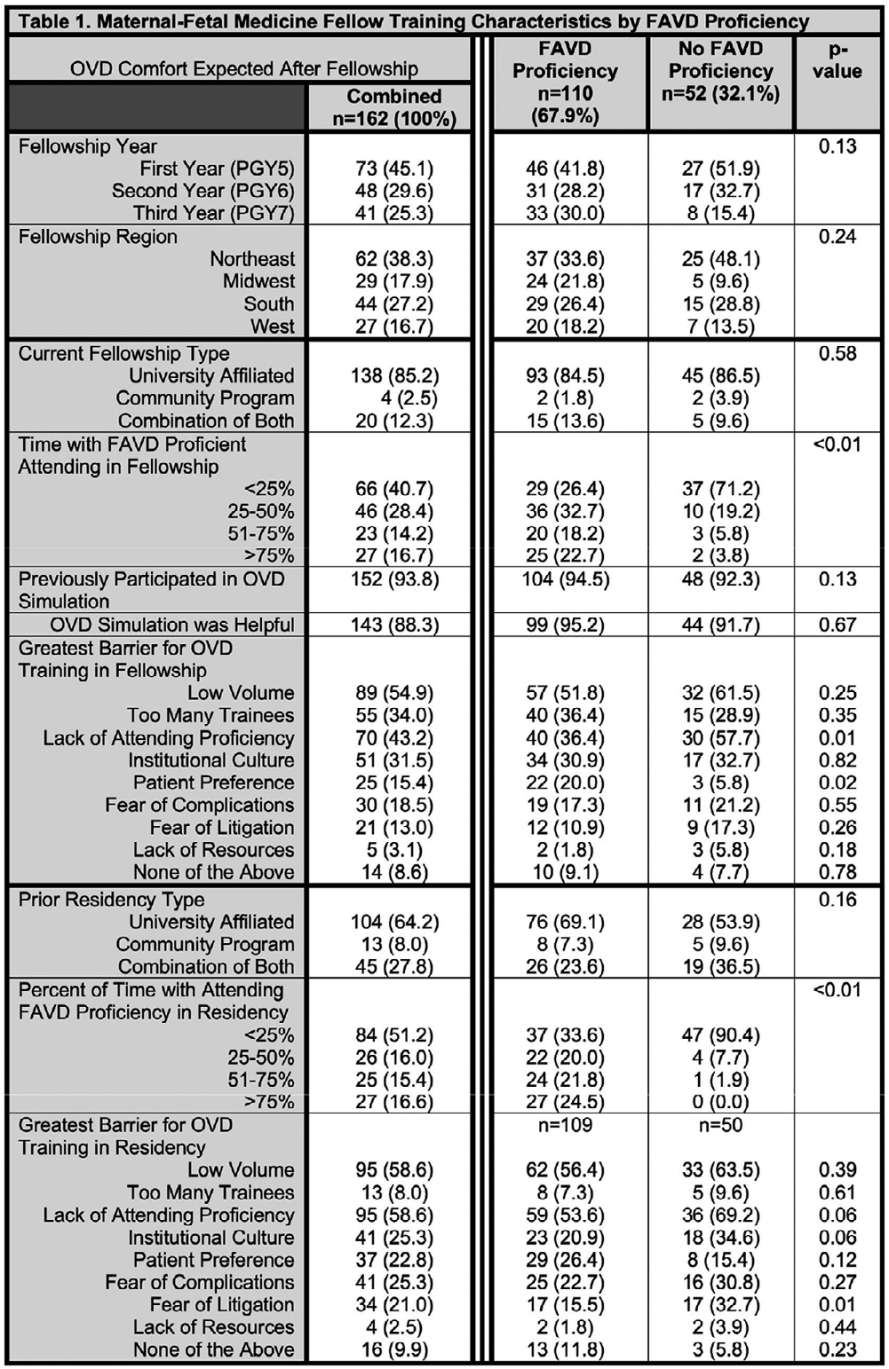

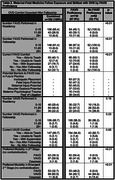

**10:30 AM – 12:00 PM**

## 302 | Perinatal Outcomes Following Intravenous Iron Therapy Among Those With Iron Deficiency Anemia in Pregnancy

Monica Sosa^1^; Brett D. Einerson^2^; Rupam Das^1^; Muyuan Zhang^3^; JJ Horns^1^; Ann M. Bruno^1^

^1^University of Utah, Salt Lake City, UT; ^2^Department of Obstetrics and Gynecology, University of Utah Health, Salt Lake City, UT; ^3^University of Utah, University of Utah, UT

**Objective**: To evaluate the association between intravenous (IV) iron therapy for maternal iron deficiency anemia (IDA) and perinatal outcomes.

**Study Design**: Repeated cross‐sectional analysis of individuals aged 13–50 years with pregnancy and delivery data at >20 weeks’ gestation available in the MarketScan Commercial Insurance Research Database from 2011–2021. Those with IDA, identified by International Classification of Diseases insurance claims codes, were included. We could not control for IDA severity in this dataset. The exposure was receiving IV iron, identified by National Drug Codes. Maternal and neonatal outcomes included severe maternal morbidity, postpartum hemorrhage, postpartum depression, need for blood transfusion, neonatal IDA and NICU admission. Baseline characteristics were compared between those receiving and not receiving IV iron therapy in pregnancy or through 6 weeks postpartum. Multivariable logistic regression models estimated the association between IV iron therapy and maternal and neonatal outcomes. A Cox proportional hazard model estimated the association between IV iron therapy and neonatal IDA.

**Results**: Of 110,719 pregnancies complicated by IDA, 7911 (7.15%) received IV iron therapy. Those receiving IV iron were older and were more likely to have maternal co‐morbid health condition (Table 1). IV iron therapy was associated with higher odds of postpartum depression (5.55% vs 3.77 %, aOR 1.50, 95% CI 1.36–1.66), postpartum hemorrhage (4.68% vs 3.74%, aOR 1.25, 95% CI 1.12–1.40) and severe maternal morbidity (5.47% vs 4.80%, aOR 1.14, 95% CI 1.03–1.26) with no difference in maternal blood transfusion. IV iron therapy was associated with increased risk of neonatal IDA (59.0% vs 41.1 %, HR 1.78, 95% CI 1.73–1.83).

**Conclusion**: Among those with maternal IDA, IV iron therapy was associated with higher risk of some adverse maternal and neonatal complications. Findings may reflect residual confounding with more severe anemia in those receiving IV iron therapy, which could not be evaluated or controlled for in this dataset. Further research is needed to optimize management of maternal IDA.

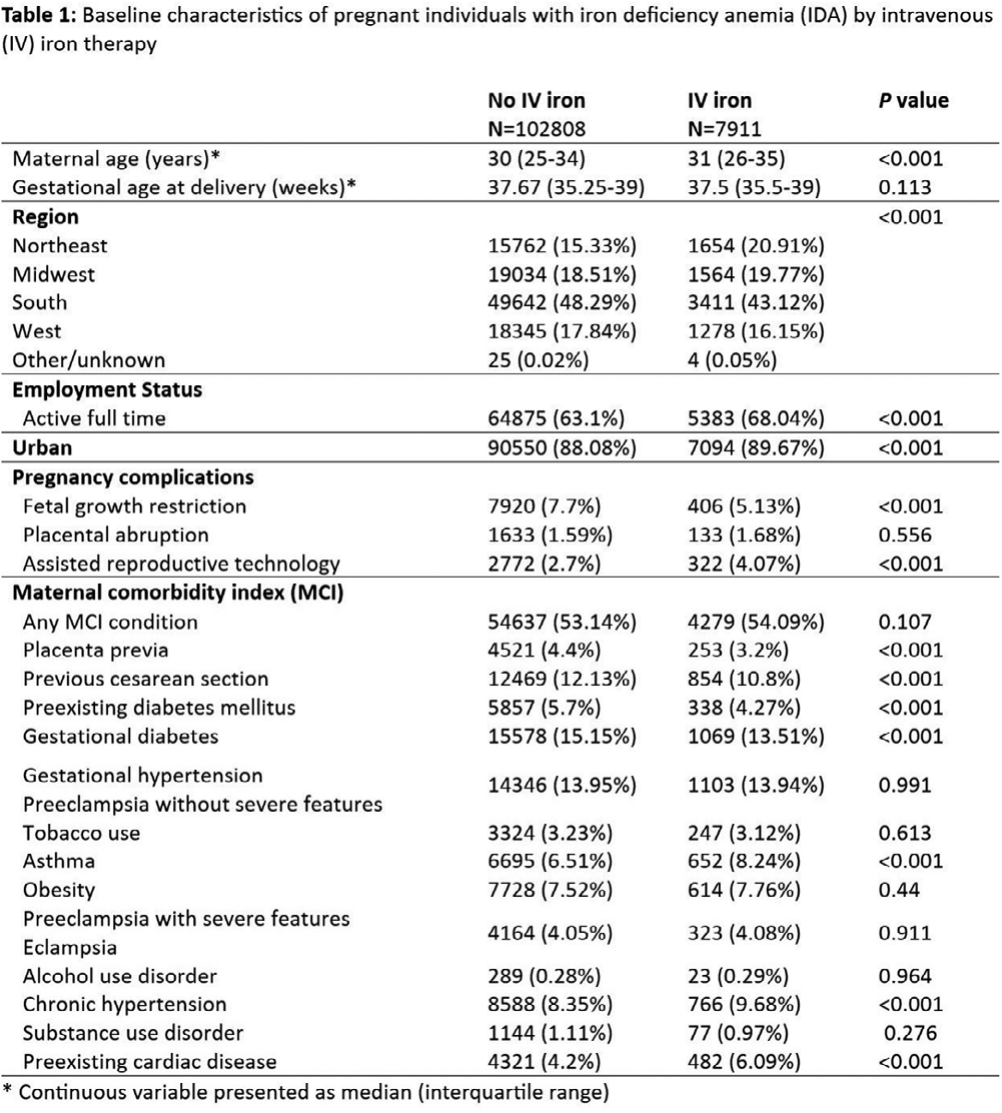

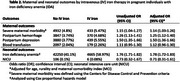

**10:30 AM – 12:00 PM**

## 303 | Fetal Brain Changes in Fetuses Exposed to Threatened Preterm Labor Delivered Near or at Term

Montse Palacio *^1^; Clara Murillo Bravo *^2^; Elisenda Eixarch^2^; Claudia Rueda^1^; Marta Larroya^1^; David Boada^1^; Laia Grau^3^; Júlia Ponce^1^; Victoria Aldecoa^4^; Elena Monterde^5^; Sílvia Ferrero^6^; Vicente Andreu‐Fernández^7^; Gemma Arca^5^; Laura Oleaga^5^; Olga Ros^5^; Maria Pilar Hernández^5^; Eduard Gratacós^1^; Teresa Cobo^8^

^1^Hospital Clínic Barcelona, BARCELONA, Catalonia; ^2^Hospital Clínic Barcelona, HOSPITAL CLINIC BARCELONA / BARCELONA, Catalonia; ^3^Hospital Sant Joan de Deu, BARCELONA, Catalonia; ^4^Hospital Clínic Barcelona, Hospital Clinic (Barcelona), Catalonia; ^5^Hospital Clínic Barcelona, Hospital Clínic Barcelona, Catalonia; ^6^BCNatal–Barcelona Center for Maternal Fetal and Neonatal Medicine Hospital Sant Joan de Déu and Hospital Clínic, University of Barcelona, BARCELONA, Catalonia; ^7^Biosanitary Research Institute, Valencian International University, VALENCIA, Comunidad Valenciana; ^8^Hospital Clinic Barcelona, Hospital Clinic (Barcelona), Catalonia

**Objective**: To evaluate whether fetuses exposed to threatened preterm labor (PTL) without intra‐amniotic infection or inflammation (IAI) and delivered after 34 weeks show brain structural changes, and whether these persist one month later.

**Study Design**: Prospective cohort study including singleton pregnancies with threatened PTL between 23–34 weeks, confirmed absence of IAI via amniocentesis, latency to delivery >4 weeks, and birth beyond 34 weeks. Fetal neurosonography was performed at admission and one month later. A gestational age–matched control group was included. Amniotic fluid levels of neuron‐specific enolase, S100B protein, and glial fibrillary acidic protein (GFAP) were compared to biobank samples.

**Results**: 33 fetuses from the threatened PTL group and 42 controls were analyzed. No differences were found in baseline characteristics, fetal sex, fetal growth or feto‐placental Doppler at admission or one month later. 84% of fetuses in the threatened PTL group were born at term. Total corpus callosum area was significantly smaller in the threatened PTL group at admission, mainly affecting the rostral body and anterior midbody. These differences persisted one month later. No other differences in brain biometry, intracranial structures, or cortical development (Silvian fissure, parieto‐occipital, cingulate and calcarine sulcus) were observed. Higher amniotic fluid concentrations of neuron‐specific enolase, S100B protein, and GFAP supported the neurosonographic findings at admission.

**Conclusion**: Fetuses exposed to threatened PTL without IAI and delivered near or at term exhibit persistent corpus callosum reduction, particularly in central regions. Elevated amniotic fluid neuron‐specific enolase, S100B, and GFAP levels at admission reinforce early neurosonographic findings. These results align with published data on neurodevelopmental risks in term‐born children after PTL and help identify a previously unrecognized high‐risk neonatal subgroup that could benefit from early follow‐up and preventive interventions.

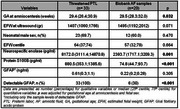

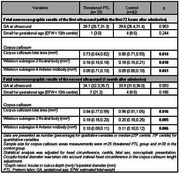

**10:30 AM – 12:00 PM**

## 305 | Area Deprivation Index and Severity of Diabetic Ketoacidosis in Pregnant Individuals With Pre‐Existing Diabetes

Melissa J. Cazzell^1^; Morgan A. Scaglione^1^; Erkan Kalafat^2^; Theresa Corbine^1^; George R. Saade^1^; Grace Spencer^3^; Marwan Ma'ayeh^1^

^1^Eastern Virginia Medical School at Old Dominion University, Norfolk, VA; ^2^Koc University Hospital, Istanbul, Koc University Hospital, Istanbul, Istanbul; ^3^Eastern Virginia Medical School at Old Dominion University, EVMS OBGYN, Virginia Health Sciences at Old Dominion University, VA

**Objective**: To evaluate whether higher levels of socioeconomic deprivation, as measured by the national Area Deprivation Index (ADI), are associated with increased severity of diabetic ketoacidosis (DKA) at presentation among pregnant individuals with pre‐existing diabetes.

**Study Design**: This was a retrospective cohort study of pregnant individuals with Type I or Type II diabetes mellitus. We identified all hospital admissions for DKA. The primary exposure was the national ADI, a composite measure of neighborhood socioeconomic disadvantage. The primary outcome was severe DKA, defined as meeting at least two of the following criteria on admission: serum bicarbonate <10 mEq/L, anion gap >20 mEq/L, or blood glucose >350 mg/dL. We used multivariable mixed‐effect logistic regression to evaluate the association between ADI and severe DKA, accounting for repeated admissions within the same pregnancy and adjusting for diabetes type, maternal age, and gestational age at admission.

**Results**: A total of 158 DKA admissions from 107 unique pregnancies were included. As expected, patients with severe DKA had significantly lower admission bicarbonate (median 9.0 vs. 17.0 mEq/L, *p* < 0.001) and higher admission anion gap (median 25.0 vs. 18.9 mEq/L, *p* < 0.001) compared to the non‐severe group (Table 1). There was no significant difference in the median national ADI between those presenting with severe DKA versus non‐severe DKA (67.0.0 vs. 58.5, *p* = 0.498). In the mixed‐effect logistic regression analysis, there was no statistically significant association between a higher ADI and the odds of presenting with severe DKA for patients with either Type I (OR 1.31, *p* = 0.886) or Type II diabetes (OR 1.11, *p* = 0.977) (Table 2).

**Conclusion**: In this cohort of pregnant individuals with pre‐existing diabetes, neighborhood‐level socioeconomic deprivation as measured by the national ADI was not associated with the clinical severity of DKA at presentation.

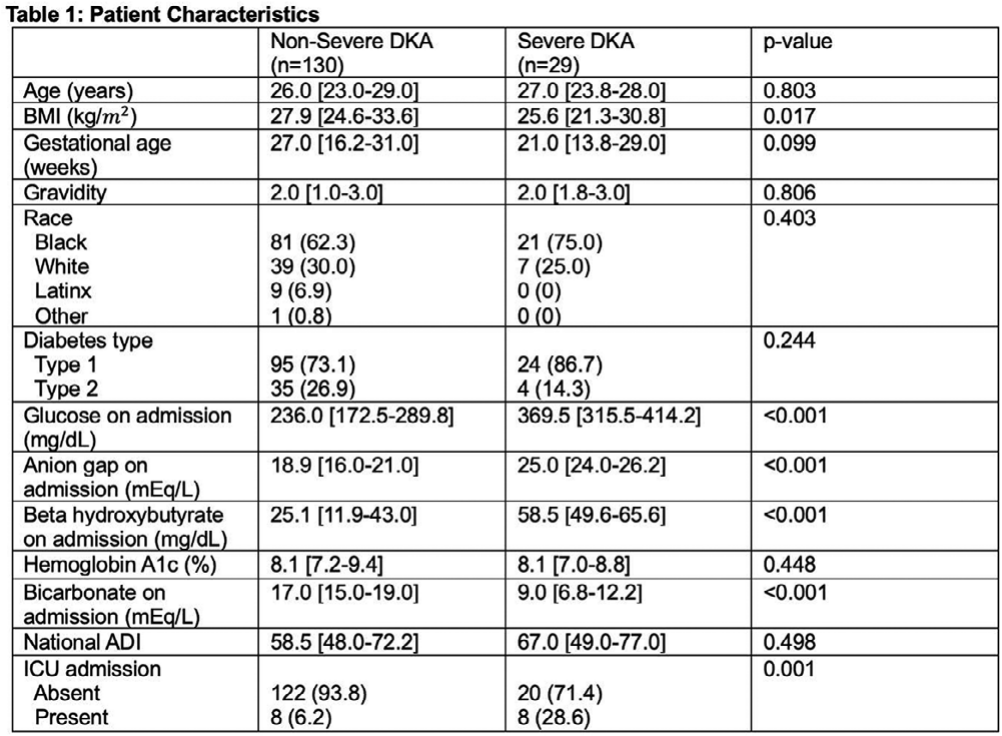

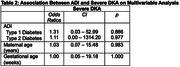

**10:30 AM – 12:00 PM**

## 306 | Planned Cesarean Versus Planned Vaginal Delivery: Maternal and Neonatal Outcomes in Low‐Risk Term Nulliparas

Moti Gulersen^1^; Itai Hazan^2^; Erez Lenchner^3^; Vincenzo Berghella^4^; Eran Bornstein^5^

^1^Thomas Jefferson University, PHILADELPHIA, PA; ^2^Ben‐Gurion University of the Negev, Beer Sheva, HaDarom; ^3^New York University Rory Meyers College of Nursing, New York, NY; ^4^Thomas Jefferson University, Philadelphia, PA; ^5^Northwell, New Hyde Park, NY

**Objective**: To compare maternal and neonatal outcomes among low‐risk nulliparous patients undergoing planned cesarean delivery (PCD) versus planned vaginal delivery (PVD) in a large contemporary United States (US) cohort.

**Study Design**: Retrospective analysis of the Centers for Disease Control and Prevention Natality Live Birth database (2016–2023). This low‐risk cohort included nulliparous, term, singleton, vertex (NTSV), in‐hospital births. Pregnancies with fetal congenital/chromosomal anomalies, pregestational and gestational diabetes, and hypertensive disorders were excluded. The rates of several adverse maternal and neonatal outcomes, as well as demographic variables, were compared between two groups: those with PCD at 39 0/7–39 6/7 weeks and those with PVD at 39 0/7–41 6/7 weeks. Multivariable logistic regression was performed to adjust for potential confounders. Data are presented as adjusted odds ratios (aOR) with 95% confidence intervals (CI).

**Results**: Of the 3,813,708 NTSVs included, 159,557 (4.2%) had a PCD. Demographic variables such as advanced maternal age, obesity, and use of assisted reproductive technology were more common in the PCD group, whereas Non‐Hispanic white race and ethnicity was more common in the PVD group. PCD was associated with higher odds of maternal intensive care unit (ICU) admission and adverse neonatal outcomes such as NICU admission and neonatal death compared to PVD (Table). However, PCD was associated with lower odds of maternal transfusion, neonatal assisted ventilation, and antibiotics for suspected neonatal sepsis (Table).

**Conclusion**: The incidence of PCD among low‐risk NTSVs in the US is low (4.2%). Both PCD and PVD are associated with adverse maternal and neonatal outcomes in NTSV patients. These findings highlight the importance of individualized counseling and shared decision‐making when planning delivery. Data from randomized controlled trials are needed to further inform optimal delivery planning in this patient population.

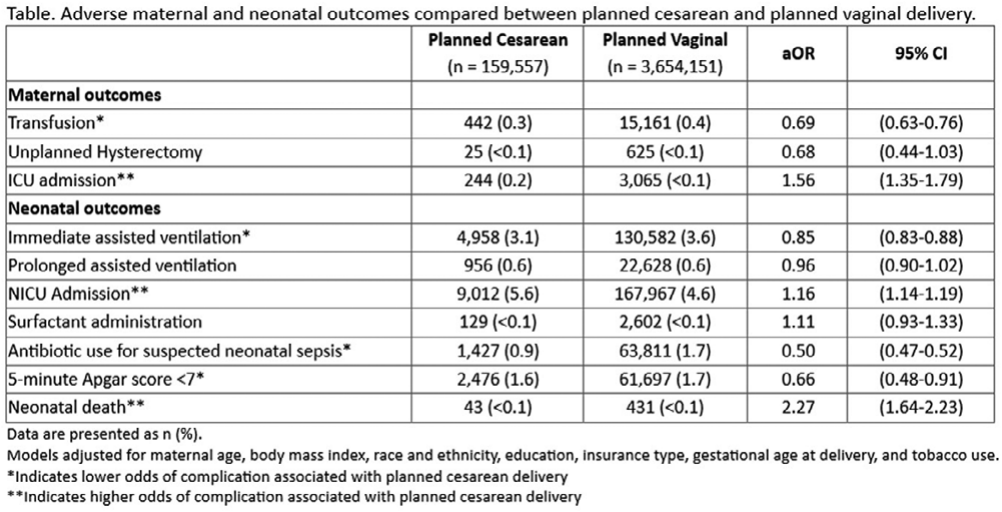

**10:30 AM – 12:00 PM**

## 307 | Beyond the Bump: Trauma's Lasting Impact on Postpartum Weight

Natalie E. Poliektov^1^; Mariana Rocha^2^; Kaitlyn K. Stanhope^3^; Alicia Smith^1^; Abigail Lott^2^; Vasiliki Michopoulos^2^; Suchitra Chandrasekaran^1^

^1^Department of Gynecology and Obstetrics, Emory University School of Medicine, Atlanta, GA; ^2^Department of Psychiatry, Emory University School of Medicine, Atlanta, GA; ^3^Rollins School of Public Health, Rollins School of Public Health, GA

**Objective**: Postpartum weight retention (PPWR), weight retained after delivery compared to pre‐pregnancy weight, is a key predictor of adverse long‐term cardiometabolic outcomes. While prior studies have linked trauma exposure to weight dysregulation in non‐pregnant populations, impacts of trauma exposure on PPWR remain poorly understood. Given the established cardiac risks associated with increased PPWR, we sought to evaluate the association between various trauma exposure domains & PPWR.

**Study Design**: We performed a secondary analysis of a prospective longitudinal cohort study investigating associations between trauma exposures & pregnancy outcomes at an urban safety net hospital. Childhood trauma (CT), Post‐Traumatic Stress (PTSD), Lifetime Stress (LS), Adult Trauma (AT), & Postnatal Depression (PD) were captured using validated trauma exposure questionnaires (Table 1). Maternal weight was recorded in each trimester & at 30 (30d) & 42 (42d) days postpartum. PPWR was calculated at 30d *(30d weight‐initial 1^st^ trimester weight)* and 42d *(42d weight‐initial 1^st^ trimester weight)*. Linear regression models were conducted to investigate associations between trauma exposure scores & PPWR, adjusting for maternal age and body mass index.

**Results**: In our cohort of *N* = 328, CT *(β = 0.33, 95% CI 0.07‐0.60*, *p* = 0.015), PTSD *(β = 0.29, 95% CI 0.03‐0.55*, *p* = 0.031), & LS *(β = 1.93, 95% CI 0.63–3.22*, *p* = 0.004) were associated with PPWR at 30d (Figure 1). There was no association between AT & PPWR (*p* = 0.24). A trend was observed between PD & PPWR *(β = 0.75, 95% CI −0.02–1.5, p = 0.05)*. At 42d, associations between CT *(β = 0.41, 95% CI 0.06‐0.76, p = 0.02) &* LS *(β = 2.05, 95% CI 0.14–3.96, p = 0.03)* & PPWR remained significant. Interestingly, at 42d, PD was significantly associated with PPWR *(β = 1.27, 95% CI 0.11–2.4, p = 0.03)* whereas PTSD (*p* = 0.08) & AT (*p* = 0.29) were not.

**Conclusion**: Our data innovatively demonstrate that trauma exposure, & specific subtypes of trauma, significantly impact PPWR. As the paradigm of postpartum care evolves, incorporating trauma‐informed strategies is essential to reduce long‐term maternal cardiometabolic risk.

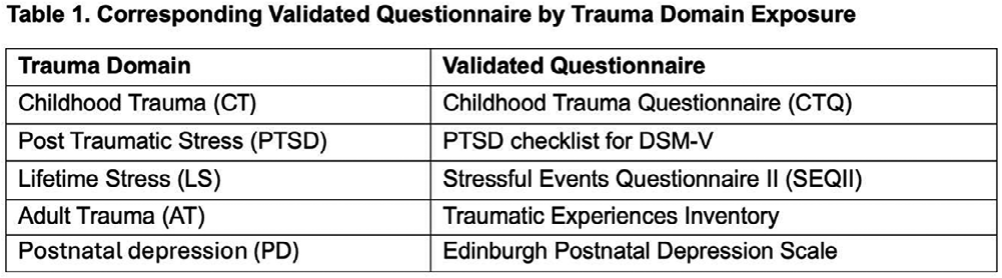

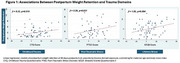

**10:30 AM – 12:00 PM**

## 308 | Adverse Pregnancy Outcomes Associated With Cystic Fibrosis Carriers

Neel K. Rana^1^; Bharti Garg^2^; Alyssa R. Hersh^1^; Marie J. Boller^3^; Aaron B. Caughey^2^

^1^Oregon Health & Science University, Portland, OR; ^2^Oregon Health & Science University, Oregon Health & Science University, OR; ^3^Oregon Health and Science University, Portland, OR

**Objective**: Since 2001, the American College of Obstetrics and Gynecology (ACOG) has recommended cystic fibrosis (CF) screening in pregnancy for individuals at elevated risk. In 2011 ACOG updated their recommendation to universal screening in pregnant individuals. Despite increasing numbers of pregnant individuals learning they are carriers for CF, data regarding the impact of this status on pregnancy outcomes remain limited. This study aimed to investigate the risk of adverse perinatal outcomes in CF carriers compared to those without CF.

**Study Design**: This is a retrospective cohort study using linked vital statistics and hospital discharge data for births in California (2008–2020). We included pregnant individuals with singleton gestation who delivered between 23 and 42 weeks. We compared demographic characteristics and outcomes between pregnant individuals who are CF carriers to those without a CF mutation. ICD‐9 and ICD‐10 codes were used to identify CF carriers (V83.81/Z14.1). Chi‐square tests and multivariable Poisson regression models were used to assess risk of outcomes.

**Results**: A total of 5,395,388 births were included, with 11,851 (0.22%) identified as CF carriers. CF carriers were more likely be non‐Hispanic White (60.0% vs 26.5%,), to have private insurance (75.4% vs 48.4%,), and be nulliparous (46.4% vs 39.4%,) than those without. After adjusting for confounders, CF carriers were more likely to develop hypertensive disease of pregnancy (aRR = 1.25, 95% CI 1.19–1.31), severe maternal morbidity (aRR = 1.30, 95% CI 1.12–1.51), non‐transfusion severe maternal morbidity (aRR 1.30, 95% CI 1.04–1.63), PPROM (aRR = 1.29, 95% CI 1.15–1.44), and neonatal hypoglycemia (aRR = 1.39, 95% CI 1.25–1.54).

**Conclusion**: We found that CF carriers are at increased risk of adverse perinatal outcomes. These findings can be used for patient counseling and delivery planning. Future studies should further investigate these findings and strategies to mitigate these risks.

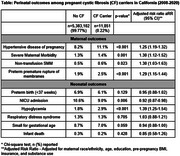

**10:30 AM – 12:00 PM**

## 309 | Labor Management Mediates Racial and Ethnic Disparities in Mode of Delivery Among an NTSV Cohort

Nicola F. Tavella^1^; Katie Larratt^2^; Christina Hope Lefebvre^2^; Hajer Naveed^2^; Donessa Colley^2^; Lily Leibner^2^; Meghna Wagley^3^; Toni Stern^2^; Joanne L. Stone^2^; Angela T. Bianco^2^

^1^Division of Maternal‐Fetal Medicine, Icahn School of Medicine, Icahn School of Medicine at Mount Sinai Hospital, New York, NY; ^2^Icahn School of Medicine at Mount Sinai, New York, NY; ^3^SUNY Downstate Health Sciences University, Brooklyn, NY

**Objective**: Research has previously reported racial and ethnic disparities in labor and delivery management. Despite knowledge of disparate care, these differences persist, and their impacts on maternal and neonatal outcomes are under‐studied. However, further rigorous investigations can potentially help reduce perinatal health inequities. This study examined racial and ethnic disparities in labor management and these disparities’ mediating effects on the risk of cesarean delivery (CD).

**Study Design**: This was a retrospective study of all NTSV deliveries in 2021 at a single urban academic medical center. We included patients who had nulliparous, term, singleton, and vertex gestations and who were admitted for scheduled induction or spontaneous labor. Individual‐level data were abstracted from electronic medical records. Multivariable linear regression models estimated the associations between racial or ethnic identity and labor management while adjusting for individual‐level covariates. Mediation analysis examined how differences in labor management mediate the relationship between racial and ethnic identity and mode of delivery.

**Results**: A sample of 2717 laboring patients was included. Hispanic patients had significantly longer times from 1) admission to delivery, 2) admission to full dilatation, 3) admission to epidural, and 4) second stage of labor to delivery, compared to White patients. Asian patients also had longer time from admission to delivery compared to White patients (Table 1). Two of these variables (admission to delivery and admission to full dilatation) mediated a greater risk of unplanned cesarean delivery among Hispanic patients compared to the rest of the cohort. A shorter length of time from admission to delivery for Black patients mediated a lower risk of unplanned CD for this group compared to the rest of the cohort (Table 2).

**Conclusion**: This suggest that disparate labor management for different NTSV racial and ethnic groups mediates the causal relationship with unplanned CD. Efforts to improve intrapartum health outcomes would therefore benefit from social equity‐focused interventions.

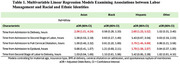

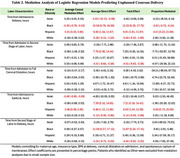

**10:30 AM – 12:00 PM**

## 310 | Impact of the American College of Obstetricians and Gynecologists (ACOG) Pregestational Diabetes 2024 Screening Update

Nicole Loza^1^; Alexis D. Pean^1^; Tina Liu^1^; Navya Kancharla^1^; Rachel L. Wood^2^; Madeline Morello^3^; Sarah K. Dotters‐Katz^4^

^1^Duke University School of Medicine, Duke University/Durham, NC; ^2^Division of Maternal Fetal Medicine, Department of Obstetrics and Gynecology, Duke University, Durham, NC; ^3^Department of Obstetrics and Gynecology, Duke University, Durham, NC; ^4^Division of Maternal Fetal Medicine, Department of Obstetrics and Gynecology, Duke University, Duke University/Durham, NC

**Objective**: In July 2024, ACOG updated gestational diabetes screening guidelines, pivoting from early glucose tolerance test(GTT) to early testing for undiagnosed pre‐existing pre‐gestational diabetes(DM) in high‐risk patients‐ defined as BMI >25 (>23 in Asian Americans) & at least 1 other risk factor. We implemented a protocol following these guidelines Sept 2024. We evaluated implementation accuracy of 2024 ACOG diabetes screening guidelines.

**Study Design**: IRB approved retrospective cohort study of patients presenting for initial OB visit in the 1st trimester within the first 3 months of guideline implementation (10/1–12/31/2024) in our large academic health system. Outcome measure was percent of patients correctly screened for diabetes testing(with A1c). Process measures included: % receiving A1c who should not have, % not receiving A1c who should have, & common screening errors.

**Results**: 962 patients presented for an initial prenatal visit in the 1st trimester in the study period. 766(79.6%) were correctly screened for A1c testing, including 31(3.2%) with known DM. 179(18.6%) patients were incorrectly screened & 17(1.8%) were correctly screened but did not have correct DM test performed. Of 179 incorrectly screened, 112(62.6% of incorrectly screened) had unindicated A1c drawn, including 54(30.2%) who did not meet BMI threshold & 58(32.4%) who met the BMI threshold but had no additional risk factor. Of note, white patients were most likely to be incorrectly tested on basis of BMI alone(*n* = 32). None of the incorrectly tested patients were found to have pre‐existing DM. Of the 84 that should have had A1c drawn, 67(79.7% of those missing indicated A1c) were incorrectly screened, 5(6.0%) screened correctly but A1c not ordered, 8(9.5%) had A1c ordered but not drawn, & 4(4.8%) had GTT ordered (Figure 1).

**Conclusion**: With implementation of the new ACOG screening guidelines, correct application of screening occurred in 79.6% individuals. The majority of missed screening resulted in over testing for DM. Continued education is necessary to increase screening accuracy.

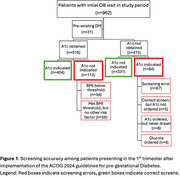

**10:30 AM – 12:00 PM**

## 311 | Psychiatric and Hypertensive Comorbidities as Independent and Interactive Risk Factors for Severe Maternal Morbidity

Nida Ali^1^; Bo Park^2^; Ashra Tugung^1^; Sergio A. Karageuzian^3^; Havilah Reimche‐Vu^4^; Kriti N. Vedhanayagam^5^; Neville Tritch^4^; Kevin H. Hu^5^; Ruofan H. Yao^5^

^1^Loma Linda University Children's Health | Perinatal Institute, Loma Linda, CA; ^2^California State University, Fullerton, Fullerton, CA; ^3^Loma Linda University Health, Loma Linda, CA; ^4^Loma Linda University School of Medicine, Loma Linda, CA; ^5^Loma Linda University, Loma Linda, CA

**Objective**: This study aimed to examine how psychiatric conditions and hypertension (HTN), individually and in combination, are associated with severe maternal morbidity (SMM). While each condition is known to increase risk, the effect of having both is not well understood.

**Study Design**: We conducted a population‐based retrospective cohort study using linked administrative and birth certificate data from over 3 million deliveries in California (2008–2012). Patients were categorized into four groups: no psychiatric or HTN diagnosis (*n* = 3,048,897), psychiatric only (*n* = 12,405), HTN only (*n* = 15,118), and both conditions (*n* = 160). We compared SMM rates using Chi‐square tests and used Cox proportional hazards models to estimate adjusted hazard ratios (HRs) and 95% confidence intervals (CIs), accounting for maternal age, parity, prior cesareans, and year of delivery.

**Results**: Of 3,076,580 total births, 1.47% (*n* = 45,261) involved SMM. SMM occurred in 1.44% of those with neither condition, 2.60% in psychiatric‐only (HR: 1.70; 95% CI: 1.52–1.90), 5.79% in HTN‐only (HR: 5.52; 95% CI: 5.16–5.91), and 8.12% in those with both conditions (HR: 7.13; 95% CI: 4.14–12.28). After adjusting for other factors, psychiatric conditions alone were linked to a 70% higher risk of SMM, HTN alone increased the risk more than five‐fold, and having both conditions resulted in over seven times the risk compared to patients with neither.

**Conclusion**: Psychiatric disorders and hypertension are each associated with increased SMM risk, and the interaction of both leads to a substantially greater risk. These results highlight the importance of integrated prenatal care that addresses both mental and physical health to improve maternal outcomes.

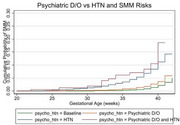

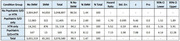

**10:30 AM – 12:00 PM**

## 312 | Hypertensive Disorders in Twins; Impact on Subsequent Singleton Pregnancy

Nimrod Dori‐Dayan^1^; Michal Fishel Bartal^2^; Michal Axelrod^3^; Avihu Krieger^4^; Shali Mazaki‐Tovi^5^; Baha M. Sibai^6^; Keren Ofir^3^

^1^Sheba Medical Center, Ramat Gan, HaMerkaz; ^2^Division of Maternal‐Fetal Medicine, Department of Obstetrics, Gynecology and Reproductive Sciences, Sheba Medical Center, Tel Aviv University, Israel, Ramat Gan, HaMerkaz; ^3^Sheba Medical Center, Sheba Medical Center, HaMerkaz; ^4^The Department of Obstetrics and Gynecology, The Chaim Sheba Medical Center, Ramat‐Gan, Israel, Ramat Gan, Tel Aviv; ^5^Department of Obstetrics and Gynecology, Sheba Medical Center, Ramat Gan, Tel Aviv; ^6^Division of Maternal‐Fetal Medicine, Department of Obstetrics, Gynecology and Reproductive Sciences, McGovern Medical School at UTHealth Houston, Houston, TX

**Objective**: Data on recurrent hypertensive disorders of pregnancy (HDP) after a twin gestation, particularly when stratified by the presence of severe features (SF), are limited. This study aimed to determine whether history and a severity of HDP in a twin gestation are associated with an increased risk of recurrence and fetal and maternal adverse outcomes in a subsequent singleton pregnancy, and to determine the role of severe features (SF) as a predictor for future pregnancy risk and management.

**Study Design**: A retrospective cohort study of patients with a twin pregnancy complicated by HDP followed by a singleton pregnancy from 2011 to 2025. Participants were stratified by the presence or absence of SF during the index twin pregnancy. Maternal and neonatal outcomes in the subsequent singleton gestation were compared between groups.

**Results**: The study population included 5908 twin pregnancies, of them 577 (9.8%) were complicated by HDP and 82 had a subsequent singleton pregnancy. The recurrence rate of HDP was 18.3%. Recurrence was significantly higher in those with prior HDP and SF (36.7%) compared to those without prior SF (7.7%, *p* = 0.001). Additionally, 27% of those with prior HDP and SF developed HDP with SF in the subsequent pregnancy, whereas none of the patients without prior SF did. HDP with SF in the index twin pregnancy was associated with a higher rate of adverse composite outcomes (46.7% vs. 21.2%, *p* = 0.02), including placental abruption, fetal growth restriction, preterm labor, and recurrent HDP (Tables).

**Conclusion**: A history of HDP with severe features in a twin pregnancy is associated with an increased risk of recurrence and adverse outcomes in a subsequent singleton gestation. In contrast, individuals with prior HDP without severe features had recurrence rates similar to the general population. These findings support incorporating HDP severity in twin pregnancies into counseling and risk stratification for future singleton pregnancies

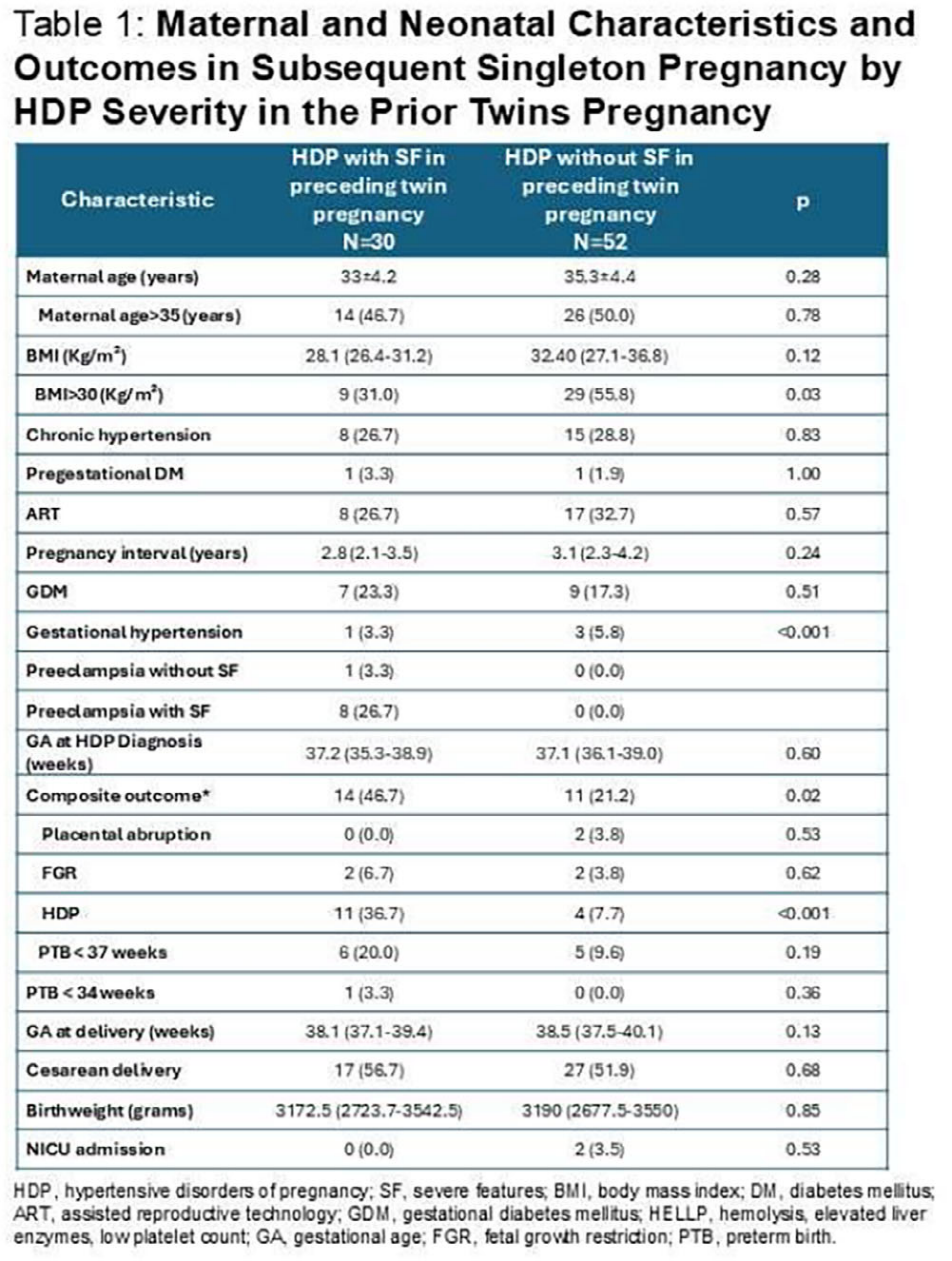

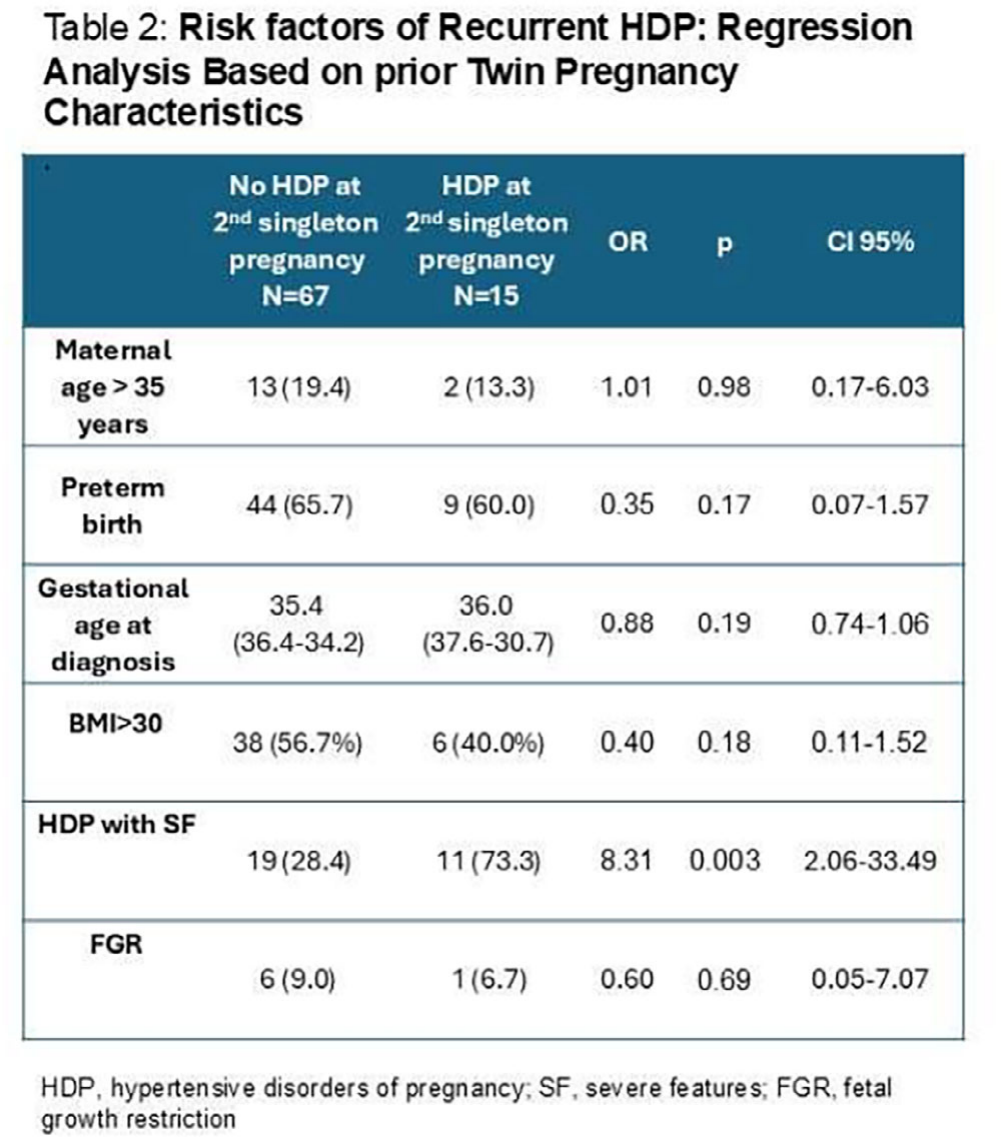

**10:30 AM – 12:00 PM**

## 313 | Risk Factors, Not Late OGTT, Predict Adverse Outcomes After a Normal GCT

Noa Haggiag^1^; Moran Gawie‐Rotman^1^; Milana Gelman^2^; Rawia Hussein‐Aro^1^; Eiman Shalabna^1^; Basel Habib Nasser^3^; Adi Malkoff Rabin^4^; Rinat Gabbay‐Benziv^5^; Amir Naeh^1^

^1^Hillel Yaffe Medical center, Hadera, HaZafon; ^2^hillel yaffe medical center, Hadera, HaZafon; ^3^Hillel Yaffe Medical center,Hadera, Israel  ; Rappaport Faculty of Medicine, Technion ‐ Israel Institute of Technology, Haifa, Israel, Hadera, HaZafon; ^4^Hillel Yaffe Medical center, Binyamina, HaZafon; ^5^Hillel Yaffe Medical Center, Hillel Yaffe Medical Center, HaMerkaz

**Objective**: Large‐for‐gestational‐age (LGA) fetus or polyhydramnios are considered relative indications for third‐trimester oral glucose tolerance test (OGTT) even after a normal glucose challenge test (GCT). The clinical significance of late‐diagnosed gestational diabetes (GDM) remains unclear. We aimed to evaluate the utility of late OGTT in this population and its association with adverse outcomes, considering maternal diabetic risk factors.

**Study Design**: This retrospective cohort included 585 singleton pregnancies (2018–2021) with normal GCT who underwent late OGTT (>28 weeks) in a university‐affiliated center. GDM was diagnosed by the two‐step strategy based on the Carpenter and Coustan criteria (≥2 abnormal values). Women were stratified according to the diagnosis of late GDM, followed by a second analysis into four groups based on OGTT results and presence of diabetic risk factors (BMI >30 kg/m^2^, family history of diabetes, prior GDM or macrosomia). Outcomes included cesarean delivery (CD), LGA, macrosomia, and birthweight. Univariate analysis was followed by a multivariable analysis to adjust for confounders.

**Results**: Among 585 women, 134 (22.9%) were diagnosed with GDM. GDM cases had lower PAPP‐A and higher first‐trimester fasting glucose levels. They delivered earlier (38.9 vs. 39.3 weeks) with lower birthweights (3437 vs. 3536 g) and lower macrosomia rates (7.5% vs. 15.1%), *p* < 0.05 for all. Nevertheless, CDs were more frequent in the GDM group, mainly for suspected macrosomia. In the second analysis, LGA rates were similar between women without GDM and those with GDM plus risk factors (35–39%), but lowest in GDM without risk factors (17.9%, *p* = 0.03). In the multivariate analysis, only diabetic risk factors—not OGTT positivity—predicted CD (aOR 2.09, 95% CI 1.3–2.1, *p* < 0.001).

**Conclusion**: Overall, a late OGTT after a normal GCT did not identify additional risk for macrosomia or LGA. Only for women with diabetic risk factors, late GDM was associated with higher CD rates. These findings support a risk‐based approach to third‐trimester OGTT even in the presence of LGA or polyhydramnios.

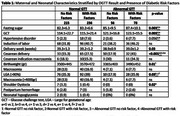

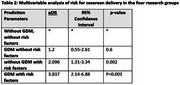

**10:30 AM – 12:00 PM**

## 314 | Twin Pregnancy Following Assisted Reproductive Treatments: Is it a Risk Factor for Adverse Perinatal Outcome?

Noa Leybovitz Haleluya^1^; Tamar Wainstock^2^; Eyal Sheiner^3^

^1^Soroka Medical Center, meitar, HaDarom; ^2^Department of Epidemiology, Biostatistics and Community Health Sciences, Faculty of Health Sciences, Ben‐Gurion University of the Negev, Beer Sheva, HaDarom; ^3^Soroka University Medical Center, Faculty of Health Sciences, Ben‐Gurion University of the Negev, beer sheva, HaDarom

**Objective**: Assisted reproductive techniques (ART), including in vitro fertilization (IVF) and ovulation induction (OI), have become increasingly widespread. While it is well established that singleton pregnancies conceived via ART carry a higher risk for adverse outcomes compared to spontaneously conceived singletons, the evidence regarding twin pregnancies is less consistent. Specifically, it remains unclear whether the increased risks associated with ART persist in multiple gestations. This study aimed to evaluate whether twin pregnancies conceived via ART are independently associated with increased rates of perinatal complications

**Study Design**: A population‐based cohort study including all offspring of twin gestations, born between the years 1991–2021 at a tertiary medical center was conducted. Perinatal morbidity and mortality, as well as obstetrical complications were assessed among twin pregnancies conceived by IVF, OI or spontaneously. Infants with congenital malformations were excluded. A multivariable generalized estimating equation (GEE) model was used to adjust for confounders.

**Results**: The study included 7790 newborns: 1380 (17.7%) were conceived by IVF, 696 (8.9%) by OI and 5714 (73.3%) with no ART. Twins pregnancies conceived by ART were at an increased risk for adverse perinatal outcome (Table): The rates of preterm delivery <37 weeks and preterm delivery <34 weeks were significantly higher in ART groups. In addition, significantly higher rates of low birthweight as well as very low birthweight were noted in ART groups. Higher rates of cesarean delivery were also noted in the ART groups. Using a multivariable GEE model, IVF remained an independent risk factor for preterm delivery <37 weeks (adjusted OR 1.57, 95% CI 1.30–1.89; *p* < 0.001) as well as for cesarean deliveries (adjusted OR 2.05, 95% CI 1.64–2.56; *p* < 0.001). No independent association was found between OI and adverse perinatal outcomes while controlling for potential confounders.

**Conclusion**: Twin pregnancies conceived via IVF are independently associated with an increased risk of preterm delivery (<37 weeks) and cesarean delivery.

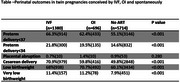

**10:30 AM – 12:00 PM**

## 315 | Trauma‐Informed Maternity Care: Insights From Women's Experiences After perinatal counseling

Hedi Benyamini‐Raischer^1^; Ayelet Oreg^2^; Ayelet Gertner‐Bonfis^3^; Adva Pollak‐Gan^3^; Noah Zafran^3^

^1^Emek Medical Center, Aכוךש, HaZafon; ^2^Bar‐Ilan University, Ramat Gan, HaMerkaz; ^3^Emek Medical Center, Afula, HaZafon

**Objective**: To explore how women with histories of sexual trauma, obstetrical trauma or complex emotional backgrounds experienced peripartum, trauma‐informed care by a midwife and to understand which aspects were perceived as supportive, what challenges remained to be improved or expanded.

**Study Design**: A qualitative, multiple case study design, focusing on in‐depth analysis of eight individual cases. Each case included a semi‐structured interview with the woman after childbirth, along with examination of relevant clinical documentation and narrative accounts of trauma. The data were analyzed using reflexive thematic analysis, with attention to both shared patterns and individual variations across cases.

**Results**: Four central themes emerged:

**Relational Safety through Attuned Communication**: Women described feeling emotionally safe when clinicians listened without judgment, respected boundaries, and offered genuine presence. This trust countered prior experiences of objectification and fostered a reparative relational dynamic.

**Emotional and Somatic Preparation as Anchors for Control**: The structured care model‐including personalized birth plans and preparatory conversations, was seen as empowering. These tools enabled regaining a sense of choice and predictability.

**Limits of Preparation and Re‐encountering Trauma during Birth**: Some women experienced moments of helplessness, fear, or trauma activation during labor, often arising when medical circumstances changed unexpectedly, highlighting the need for flexible, real‐time emotional support.

**Need for Integrated and Continuous Psychosocial Support**: Participants expressed a desire for continuity of care. The emotional and psychological needs postpartum were often unmet, suggesting the necessity of more holistic and long‐term trauma‐informed maternity services.

**Conclusion**: Trauma‐informed approaches may enhance safety, agency, and emotional well‐being during pregnancy and childbirth. However, postpartum care may be essential for truly comprehensive, trauma‐sensitive maternity care.

**10:30 AM – 12:00 PM**

## 316 | **Doppler Progression, Time to Delivery, and Risk of Fetal Demise in Early‐Onset Severe FGR**

Noam Pardo^1^; John C. Kingdom, MD^2^; Nir Melamed^3^

^1^Sunnybrook Health Sciences Centre, Toronto, ON; ^2^Maternal Fetal Medicine Division, Department of Obstetrics and Gynaecology, Mount Sinai Hospital, University of Toronto, Toronto, Ontario, Canada, Toronto, ON; ^3^Sunnybrook Health Sciences Center, Toronto, ON

**Objective**: Understanding the natural history of umbilical artery (UA) Doppler in early‐onset fetal growth restriction (FGR) is critical for optimal management. However, data on UA Doppler progression and time to delivery are limited by heterogeneous study populations and small sample sizes. Our objective was to characterize the rate of progression of UA Doppler abnormalities, time to delivery, and risk of intrauterine fetal demise (IUFD) in a homogeneous cohort of pregnancies with severe early‐onset FGR that ultimately progressed to absent or reversed end‐diastolic flow (AREDF) in the UA

**Study Design**: This was a retrospective cohort study of patients with a singleton or dichorionic twin pregnancy and severe early‐onset FGR that ultimately progressed to AREDF (2014–2024). All cases with genetic or major anatomical anomalies were excluded. UA Doppler findings were categorized as elevated UA‐PI (>95th percentile), intermittent or persistent absent end‐diastolic flow (iAEDF or AEDF), and intermittent or persistent reversed end‐diastolic flow (iREDF or REDF).

**Results**: A total of 287 patients (2,536 ultrasound exams) met the study criteria. There were 16 (5.6%) cases of IUFD. The median (IQR) time to progression was 5 (2–9) days from elevated UA‐PI to iAEDF, 4 (2–9) days from iAEDF to AEDF, 5 (2–12) days from AEDF to iREDF, 2 (1–9) days from iREDF to REDF, and 0 (0–3) days from REDF to late DV changes (Figure 1). The risk of IUFD increased progressively from 1.0% with iAEDF to 14.5% with REDF (Figure 1). The time to delivery was 7 (3–16) days for elevated UA‐PI, 5 (2–12) days for iAEDF, 3 (1–8) days for AEDF, 0 (0–4) days for iREDF, 0 (0–1) days for REDF, 2 (0–11) days for elevated PI in the DV, and 0 (0–4) days for late DV changes (Figure 2).

**Conclusion**: The rates of Doppler progression and time to delivery observed in this homogeneous cohort with severe FGR and AREDF represent a worst‐case clinical scenario and can thus inform recommendations on the minimum interval for fetal surveillance. Importantly, the risk of IUFD remains low in the absence of REDF and abnormal ductus venosus Doppler findings.

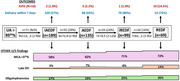

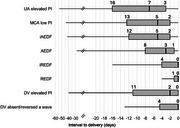

**10:30 AM – 12:00 PM**

## 317 | Impact of Food Deserts on Maternal Behavioral Health During Pregnancy

Noor K. Al‐Shibli^1^; Elizabeth C. Rhodes^2^; Erin Ferranti^3^; Anne L. Dunlop^4^; Suchitra Chandrasekaran^5^

^1^Emory University School of Medicine, Emory University School of Medicine, GA; ^2^Rollins School of Public Health, Emory University, Atlanta, GA; ^3^Nell Hodgson Woodruff School of Nursing, Dept of Gynecology & Obstetrics, Emory University, Nell Hodgson Woodruff School of Nursing, Dept of Gynecology & Obstetrics, Emory University/ Atlanta, GA; ^4^Department of Gynecology and Obstetrics, Division of Research, Emory University School of Medicine, Dept of Gynecology & Obstetrics, Division of Research, Emory University School of Medicine/ Atlanta, GA; ^5^Department of Gynecology and Obstetrics, Emory University School of Medicine, Atlanta, GA

**Objective**: Food deserts (FD), regions with limited access to nutritious and affordable foods, often reflect broader social and geographic inequities. In non‐pregnant populations, FD residence has been linked to poorer mental health outcomes & substance use disorders (SUD). Given that maternal mental health disorders (MMHD) & SUD are leading contributors to maternal mortality in the U.S., we sought to examine associations between living in a FD & the risk of MMHD & SUD.

**Study Design**: Utilizing our institutional obstetric database, we performed a retrospective cohort study of all deliveries from 2016–2023 with documented zip‐code & clinical/demographic data. FD status was assigned by zip‐code of primary residence according to the U.S. Department of Agriculture (USDA) definitions. MMHD & SUD were identified using ICD‐10 codes & clinical data. Kruskal‐Wallis & Chi‐square tests were performed to compare continuous & categorical variables, respectively. Poisson regressions, adjusted for relevant confounders, were used to estimate incidence risk ratios (IRR).

**Results**: Among *N* = 21,065 pregnancies, 7160 (34.0%) lived in a FD. FDs had higher rates of Black/African American individuals *(74% vs 54%, p < 0.001)* and lower rates of White individuals *(11.7% vs 19.3%, p < 0.001)*. Living in a FD was associated with higher rates of MMHD *(11.5% vs 10.2%, p < 0.001)* & SUD *(6.7% vs 4.8%, p < 0.001)*. Controlling for maternal age, SUD & race determined that living in a FD was associated with a 1.1x higher risk for MMHD *(IRR 1.1, 95% CI 1.01–1.20, p = 0.02)*. Controlling for maternal age, MMHD & race, those in a FD had a 1.3‐fold higher risk for SUD *(IRR 1.3, 95% CI 1.2–1.5, p < 0.001)*.

**Conclusion**: Living in a FD is independently associated with an increased risk of MMHD and SUD. Our findings underscore the urgent need for public health strategies to address structural inequities in food access, housing and neighborhood conditions. Interventions targeting social determinants of health are essential to improving MMHD, advancing equity in perinatal care, and ultimately decreasing adverse pregnancy outcomes.

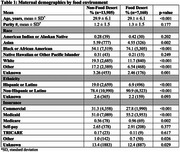

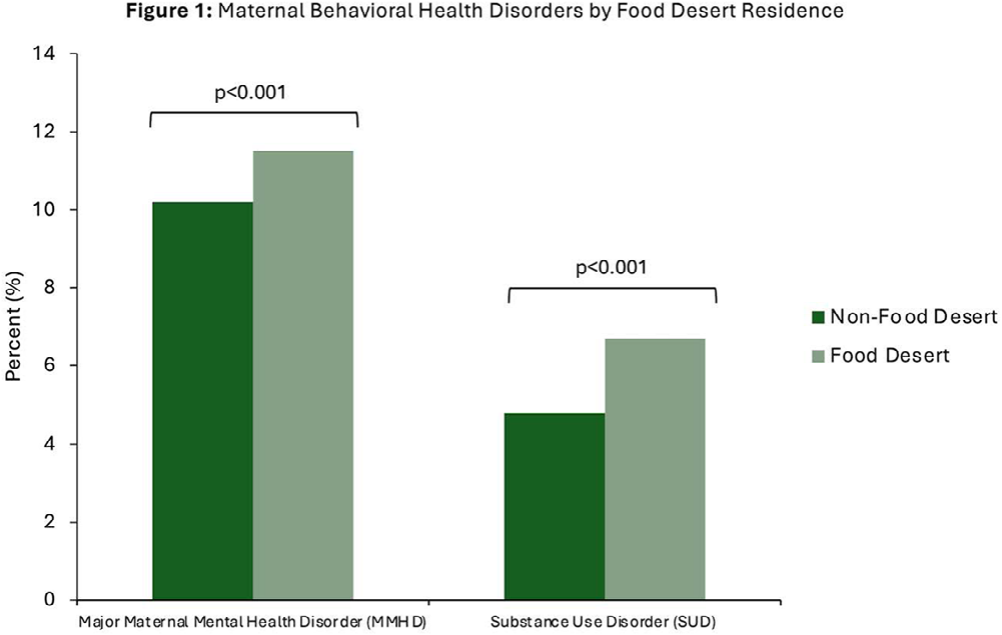

**10:30 AM – 12:00 PM**

## 318 | Factors Associated With Infant Feeding Choice in People Living With HIV

Noor Chadha^1^; Willa Molho^2^; Emily C. Swisher‐Rosa^1^; Ellen Cooper^1^; Cassandra Pierre^1^; Naima T. Joseph^1^

^1^Boston Medical Center, Boston, MA; ^2^Boston University, Boston, MA

**Objective**: In January 2023, the U.S. Department of Health and Human Services revised its clinical guidelines from a recommendation against breastfeeding to an endorsement of patient‐centered, shared decision‐making regarding infant feeding in the context of maternal HIV infection. This study sought to characterize infant feeding decisions among pregnant PLWH following this update.

**Study Design**: We evaluated breastfeeding rates among PLWH between 2023–2024 at a single academic tertiary medical center in the United States. Baseline characteristics and key perinatal outcomes were abstracted from electronic records and compared across patients who elected breastfeeding versus formula feeding. Mann‐Whitney U test and Fisher's Exact Test were used to compare continuous and categorical data, respectively.

**Results**: A total of 37 PLWH delivered between January 2023 and December 2024, of whom 7 elected to breastfeed (18.9%). Median duration of breastfeeding was 182 days (IQR 84.0, 188.5). Compared to PLWH who chose formula, a higher proportion of breastfeeding PLWH had an undetectable viral load at first prenatal visit (86% vs 47%, *p* = 0.10) and delivered vaginally (71% vs 37%, *p* = 0.20). Among the 30 PLWH who formula fed, 13 (43%) were advised against breastfeeding due to clinical concerns, including suboptimal antiretroviral adherence (*n* = 1) and detectable viral load during pregnancy (*n* = 12). Other reasons for not breastfeeding included parental concerns about transmission (*n* = 6), desire for shorter duration of infant post exposure prophylaxis (*n* = 1), no interest (*n* = 5), or infant given formula in first hour of life (*n* = 1). There was no perinatal HIV transmission in this cohort.

**Conclusion**: In this single institution study, approximately 1 in 6 PLWH chose to breastfeed. The predominant reason for formula feeding was parental and provider concern regarding HIV transmission. These findings suggest an urgent need for national data on lactation‐associated HIV transmission among those with sustained viral suppression, as well as a need for evidenced‐based tools to support shared‐decision making regarding infant feeding among PLWH.

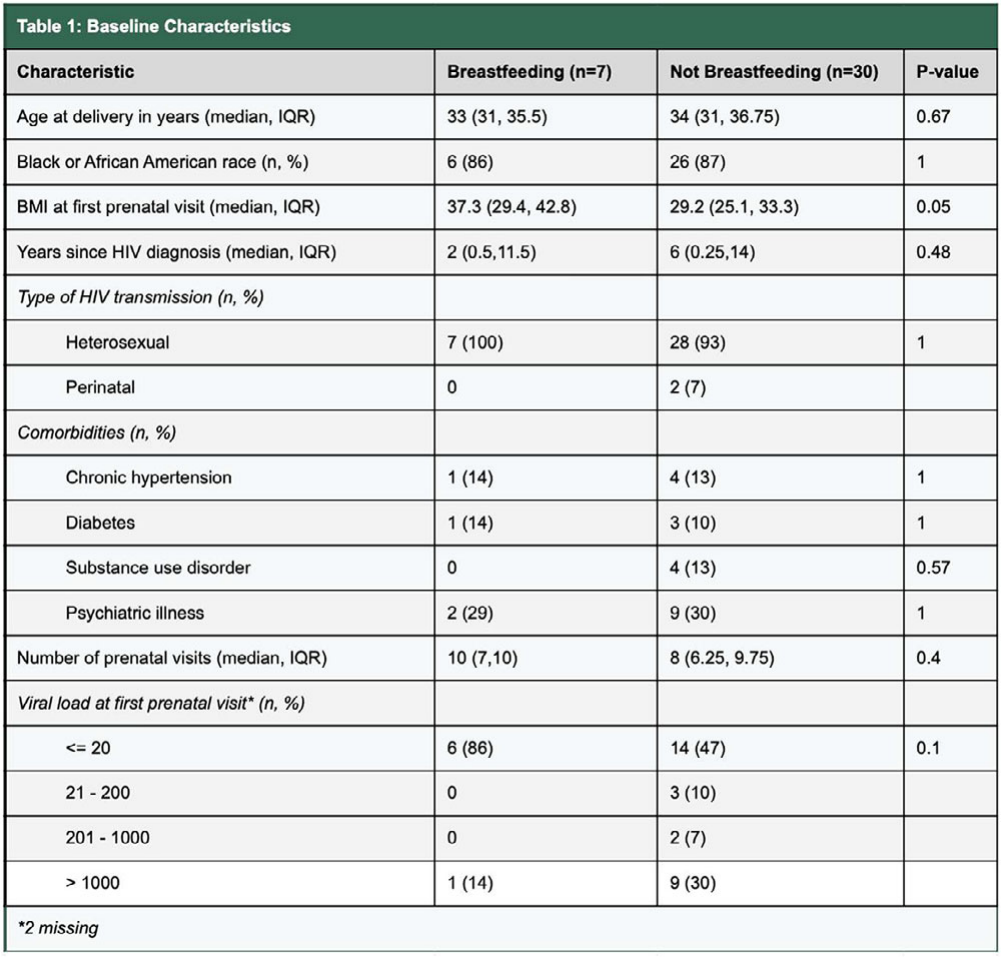

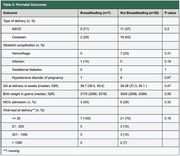

**10:30 AM – 12:00 PM**

## 319 | Impact of Healthcare Access on External Cephalic Version and Breech Delivery in a U.S. Cohort

Oladunni Ogundipe; Frank I. Jackson; Sarah H. Abelman; Luis A. Bracero; Matthew J. Blitz

Northwell, New Hyde Park, NY

**Objective**: External cephalic version (ECV) reduces rates of cesarean delivery for malpresentation. However, access to providers skilled in ECV technique may be limited in underserved areas, leading to unnecessary cesarean deliveries. This study evaluated national ECV attempt rates in term breech pregnancies, stratified by access to maternity care.

**Study Design**: This cross‐sectional study used 2023 U.S. National Vital Statistics data and included singleton deliveries ≥37 weeks with breech presentation. Patients were included if they underwent an ECV attempt (successful or failed) or delivered a breech fetus (via cesarean or vaginal delivery). Healthcare access was defined using March of Dimes classifications (full, moderate, or low access to maternity care, or maternity care desert), incorporating availability of obstetric hospitals, obstetric clinicians, and insurance coverage. Logistic regression was used to assess associations between healthcare access and mode of delivery. *p* < 0.05 was considered statistically significant. Analyses were performed in R version 4.4.1.

**Results**: Among 109,217 eligible patients, those in areas with moderate access to care were most likely to undergo ECV compared to those in full‐access regions (aOR 2.82, 95% CI 2.50–3.17). In contrast, patients in low‐access areas were significantly less likely to have an ECV (aOR 0.76, 95% CI 0.64–0.91) or a vaginal breech delivery (aOR 0.76, 95% CI 0.64–0.91). Living in a maternity care desert was not significantly associated with ECV rates. Hispanic (aOR 0.68, 95% CI 0.63–0.73) and Asian (aOR 0.57, 95% CI 0.49–0.65) patients were also less likely to undergo ECV.

**Conclusion**: Lower ECV and vaginal breech delivery rates in low‐access areas highlight potential missed opportunities to reduce cesarean deliveries. Surprisingly, ECV was more frequent in moderate‐access areas than in full‐access regions. Persistent disparities by race and ethnicity further emphasize the need for equitable access to ECV and skilled breech management.

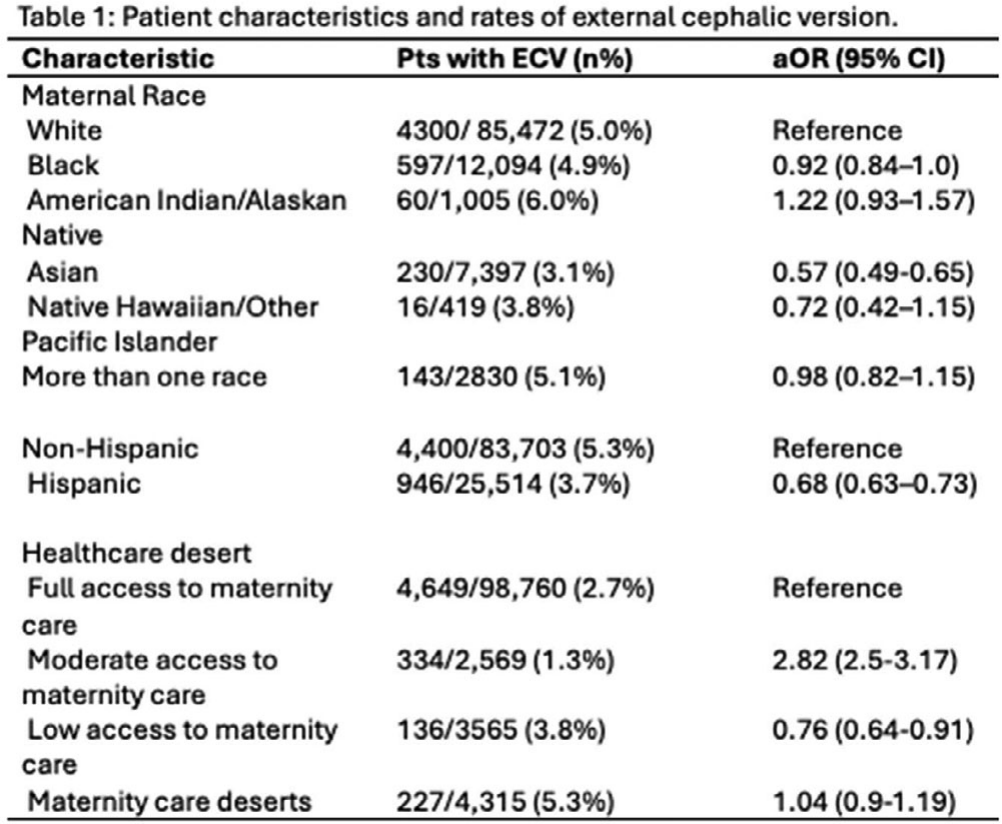

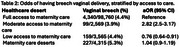

**10:30 AM – 12:00 PM**

## 320 | Twins After‐Hours: Does Timing Tip the Scale on Outcomes?

Or Eliner^1^; Gil Shechter Maor^2^; Or Touval^3^; Tal Biron Shental^4^

^1^Department of Obstetrics and Gynecology, Meir Medical Center, Ra'anana, HaMerkaz; ^2^Meir Medical Center, Kfar Saba, HaMerkaz; ^3^Department of Obstetrics and Gynecology, Meir Medical Center, Kfar Saba, HaMerkaz; ^4^Meir Medical Center, Meir Medical Center, HaMerkaz

**Objective**: To evaluate whether weekend deliveries are associated with increased maternal or neonatal complications in planned vaginal twin births.

**Study Design**: This retrospective study examined all DCDA twin gestations born between 2014 to 2020 at a single tertiary center. Deliveries were stratified by weekday and weekend deliveries. Maternal characteristics, labor parameters, and neonatal outcomes were compared. The primary outcome was a composite adverse neonatal outcome (including hypoglycemia, sepsis, respiratory distress, transfusion, cerebral hemorrhage, HIE, or convulsions). Secondary outcomes included urgent cesarean delivery of the second twin following vaginal delivery of the first one, rate of cesarean deliveries, operative vaginal delivery, intrapartum fever, and NICU admission. Multivariable logistic regression was performed to adjust for confounders.

**Results**: A total of 696 twin pregnancies were included, with 174 in the weekend group and 522 in the weekday group. Demographics and baseline obstetric characteristics were similar between groups (Table 1). Rates of urgent CD for the second twin (3.7% vs 4.7%, *p* = 0.810), operative delivery, intrapartum fever, and NICU admission did not differ significantly between weekday and weekend births. Composite adverse neonatal outcome was not significantly different (10.3% weekday vs 8.6% weekend, *p* = 0.608). Multivariable regression showed that weekend delivery was not associated with increased odds of composite neonatal morbidity (aOR 0.76; 95% CI 0.41–1.41, *p* = 0.387), nor with increased risk of urgent CD for the second twin (aOR 0.70; 95% CI 0.23–2.13, *p* = 0.529).

**Conclusion**: Weekend delivery was not associated with increased maternal or neonatal complications in planned vaginal twin deliveries. These findings suggest that, in well‐organized obstetric systems, quality of care remains consistent throughout the week. This challenges the generalizability of the “weekend effect” on twin labor management and supports the safety of off‐hours care.

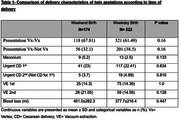

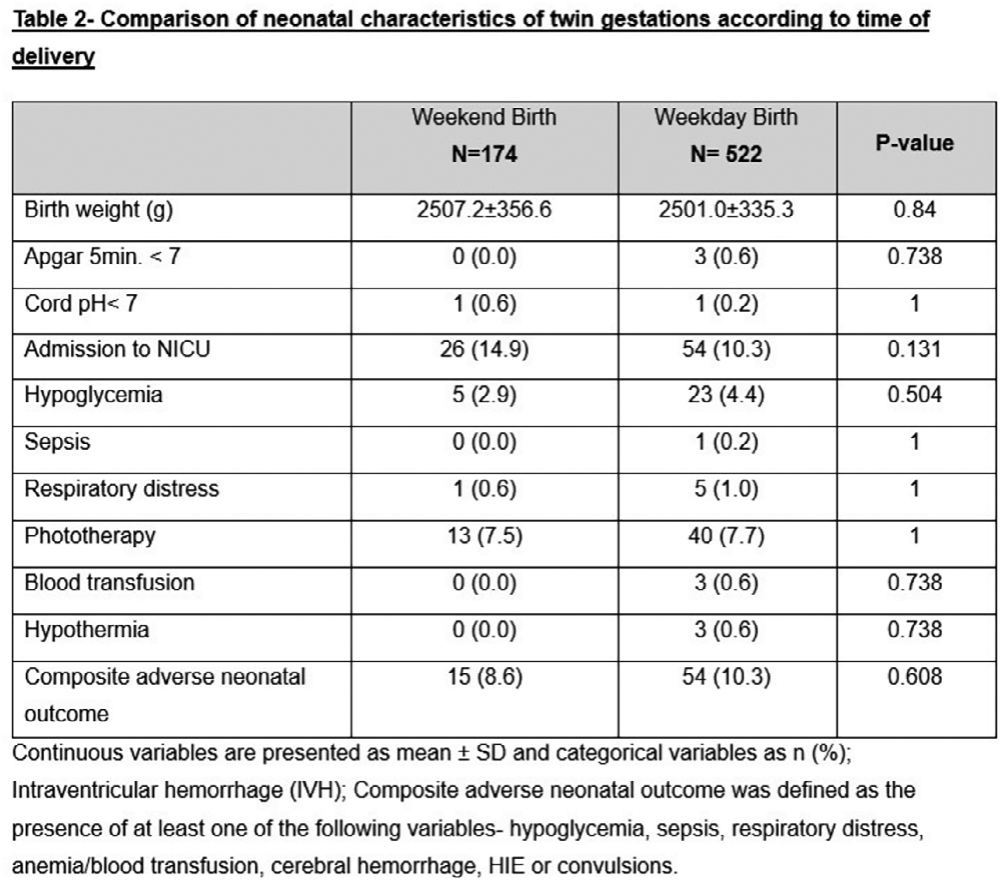

**10:30 AM – 12:00 PM**

## 321 | Perinatal Risk and Outcomes Among Individuals Insured by Medicare

Osinakachukwu Mbata^1^; Ayodamope Fawole^2^; Armand Zimmerman^2^; Maria Small^2^; Evan Myers^2^; Osondu Ogbuoji^2^

^1^Duke University School of Medicine, Durham, NC; ^2^Duke University, Durham, NC

**Objective**: Insurance status is associated with health outcomes among the general and obstetric populations. This work examines the medical conditions and perinatal outcomes for Medicare insured individuals relative to non‐Medicare insured individuals.

**Study Design**: We performed a retrospective cohort study using 2019 Healthcare Cost & Utilization Project National Inpatient Sample. We included singleton deliveries without fetal anomalies. Analyses used NIS discharge weights, strata, and hospital clusters to generate nationally representative estimates. We compared Medicare with other payer groups across maternal demographics (age and race), selected chronic medical conditions, and perinatal outcomes including severe maternal morbidity, SMM (defined by the CDC). *p* values for all outcomes compare the Medicare population with all other payers combined and a two‐sided α < 0.05 indicated statistical significance.

**Results**: The cohort included 3,697,605 deliveries, of which 28,855 deliveries were insured by Medicare. Delivering persons covered by Medicare were more likely to be Black (33.3% vs 9.5%; *p* < 0.001) and advanced maternal age (25.6% vs 23.4%; *p* < 0.001) when compared to those with private insurance. Medicare insured individuals were more likely to have chronic kidney disease (11.89% vs 9.01%; *p* < 0.001), congestive heart disease (1.39% vs 0.14%; *p* < 1), pre‐pregnancy diabetes (13.27% vs 9.53%; *p* < 0.001), and pre‐pregnancy HTN (9.91% vs 3.09; *p* < 0.001). Medicare insurance status was associated with higher rates of cesarean delivery (37.7% vs 35.1%; *p* < 0.001) and preterm birth (13.6% vs 7.5%; *p* < 0.001). Medicare status was associated with increased relative risk for severe maternal morbidity (SMM) when compared to privately insured individuals (0.28; CI 0.25–0.32).

**Conclusion**: Reproductive age individuals with Medicare insurance are more likely to enter pregnancy with chronic conditions. Medicare insured individuals were more likely to experience adverse perinatal outcomes. This highlights the need for increased investment in strategies to better support this vulnerable population.

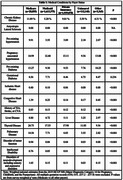

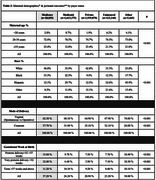

**10:30 AM – 12:00 PM**

## 322 | Bridging the Social and Clinical Divide in Congenital Major Birth Defect Etiology

Patience T. Timi^1^; Shelby A. Crants^2^; Aleksandra Polic^3^; Lynsa Nguyen^4^; Etoi Garrison^1^; Amelie Pham^1^

^1^Vanderbilt University Medical Center, Nashville, TN; ^2^Department of Obstetrics and Gynecology, MassGeneral Brigham; Harvard Medical School, Boston, MA; ^3^UPMC Magee‐Womens Hospital, Pittsburgh, PA; ^4^Valley Children's Specialty Medical Group, Madera, CA

**Objective**: Poor periconception glycemic control in pre‐existing diabetes is known to be associated with major congenital birth defects. The independent association between maternal social health drivers (SHD) and the risk of birth defects remains unclear.

**Study Design**: We evaluated all deliveries at a single academic center between 2010–2020 with periconception A1C value <6.5%. Major birth defects were identified via manual chart review of both maternal and infant charts using strict CDC and EUROCAT definitions. Deliveries affected by known aneuploidy or genetic syndromes were excluded. Poisson regression was used to assess the relationship between individual maternal SHD and any birth defects. Models were adjusted using a‐priori selected confounders including maternal age, pre‐pregnancy body mass index (BMI) and pre‐existing diabetes diagnosis.

**Results**: A total of 6436 pregnancies were identified with A1c value <6.5%. Clinical characteristics of the cohort are presented in Table 1. Crude estimates found that only social vulnerability index (SVI) subtheme 2 and BMI were associated with higher risk of birth defects (Table 2). Adjusted analyses only found a significant association between BMI and risk of any birth defects (adjusted estimate: 0.02, 95% CI: 0.01; 0.03, Table 2). In our cohort, BMI was highly associated with pre‐existing diabetes diagnosis (adjusted estimate: 0.02, 95% CI: 0.01; 0.03). SVI did have a positive association with BMI (adjusted estimate: 0.14, 95% CI: 0.11; 0.16).

**Conclusion**: No single maternal SHD accounted for the risk of any major birth defects in our cohort. Pre‐pregnancy BMI continues to be a strong risk factor for both pre‐existing diabetes and major congenital birth defects, regardless of periconception A1C, underscoring the importance of diabetes and obesity prevention and management for pregnancy. Future research should further elucidate how maternal SHDs like SVI may indirectly impact these relationships.

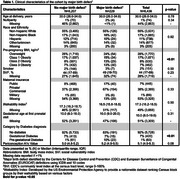

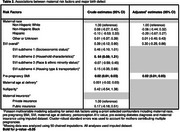

**10:30 AM – 12:00 PM**

## 323 | Adverse Obstetric and Neonatal Outcomes in Dichorionic Twins: Does Zygosity Play a Role?

Prisca C. Diala^1^; Sara Khan^2^; Tra‐Mi Bao^3^; Jared Duldulao^4^; Jiayue Chen^5^; Mehrnaz Siavoshi^6^; Lorna Kwan^6^; Neil S Silverman^7^; Ilina Pluym^3^

^1^Department of Obstetrics and Gynecology, David Geffen School of Medicine at University of California, Los Angeles (UCLA), Los Angeles, CA; ^2^Department of Pediatrics, Division of Neonatology, David Geffen School of Medicine at University of California, Los Angeles (UCLA), Los Angeles, CA; ^3^David Geffen School of Medicine at University of California, Los Angeles (UCLA), Los Angeles, CA; ^4^Department of Pediatrics, David Geffen School of Medicine at University of California, Los Angeles (UCLA), Los Angeles, CA; ^5^Department of Urology, David Geffen School of Medicine at University of California, Los Angeles (UCLA), Los Angeles, CA; ^6^Department of Urology, David Geffen School of Medicine at University of California, Los Angeles (UCLA), Los Angeles, CA; ^7^Division of Maternal Fetal Medicine, Department of Obstetrics and Gynecology, David Geffen School of Medicine at University of California, Los Angeles (UCLA), Los Angeles, CA

**Objective**: To evaluate whether zygosity is associated with preterm birth risk and perinatal outcomes in dichorionic twin pregnancies.

**Study Design**: We conducted a retrospective cohort study of dichorionic twin pregnancies delivered after 24‐week gestation at two affiliated academic urban medical centers between 2016 and 2024. Zygosity was determined by noninvasive prenatal testing (NIPT), neonatal sex at birth, or inferred from in vitro fertilization (IVF) frozen embryo transfer records. The primary outcome was preterm delivery rate in monozygotic (MZ) versus dizygotic (DZ) twins; the secondary outcome was composite neonatal morbidity. Multivariable logistic regression identified predictors of preterm delivery and neonatal morbidity after adjusting for demographic and clinical factors.

**Results**: Among 265 dichorionic twin pregnancies, 94% were DZ (*n* = 249) and 6% MZ (*n* = 16). Maternal and fetal characteristics—including age, body mass index (BMI), IVF status, hypertensive disorders (HD), gestational diabetes, fetal growth restriction (FGR), and anomalies—were similar between groups. Preterm delivery occurred in 51% of DZ and 44% of MZ pregnancies (*p* = 0.40). A neonatal morbidity event occurred in 43% of DZ and 34% of MZ pregnancies (*p* = 0.36). Severe FGR (aOR 5.09, 95% CI 1.54–16.83) and HD (aOR 1.70, 95% CI 1.16–2.51) were independent predictors of preterm delivery, while increased parity was protective (aOR 0.51, 95% CI 0.27–0.95, Fig 1). Predictors of neonatal morbidity were small for gestational age (aOR 1.77, 95% CI 1.01–3.11), IVF conception (aOR 1.72, 95% CI 1.04–2.84), HD (aOR 1.73, 95% CI 1.09–2.74), and preterm delivery (aOR 22.24, 95% CI 13.01–38.03, Fig 2).

**Conclusion**: Zygosity did not significantly impact obstetric or neonatal outcomes in dichorionic twin pregnancies. Adverse outcomes were primarily driven by pregnancy‐related factors such as FGR and HD. These findings suggest that adverse outcomes in dichorionic twin pregnancies are mainly driven by clinical factors rather than genetic twinning status.

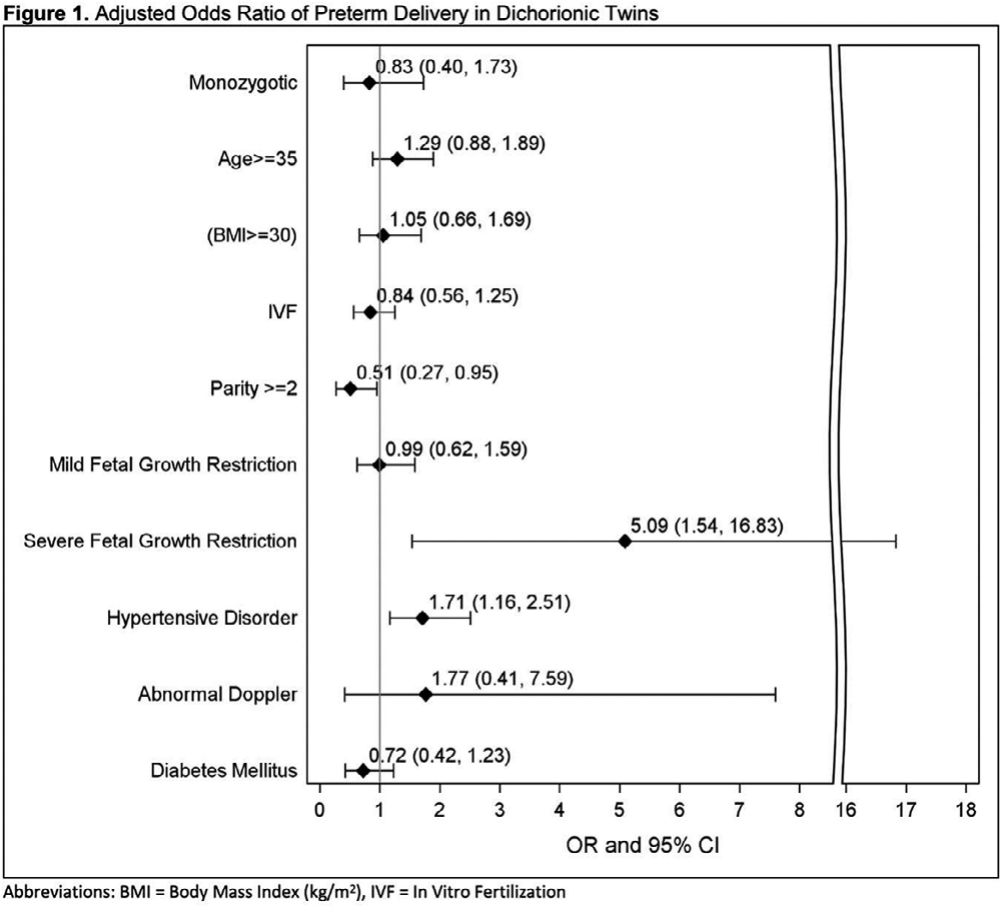

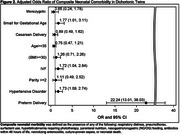

**10:30 AM – 12:00 PM**

## 324 | Association Between Low or High Dose Oxytocin Exposure in Latent Labor and Postpartum Hemorrhage

Rachel L. Bank^1^; Miriam J. Alvarez^1^; Sindhu K. Srinivas^2^; Aaron B. Caughey^3^; Alan TN Tita^4^; Methodius G. Tuuli^5^; George A. A. Macones^1^; Alison G. G. Cahill^6^

^1^Department of Women's Health, Dell Medical School at the University of Texas at Austin, Austin, TX; ^2^Department of Obstetrics and Gynecology, Perelman School of Medicine, Maternal and Child Health Research Center, Philadelphia, PA; ^3^Oregon Health & Science University, Oregon Health & Science University, OR; ^4^Center for Research in Women's Health, University of Alabama at Birmingham, Birmingham, AL; ^5^Department of Obstetrics and Gynecology, Warren Alpert Medical School at Brown University, and Women & Infants Hospital of Rhode Island, Providence, RI; ^6^Department of Women's Health, Dell Medical School at the University of Texas at Austin, Department of Women's Health, Dell Medical School at the University of Texas at Austin, TX

**Objective**: Oxytocin is commonly used for labor induction or augmentation, but the effect of latent phase exposure on postpartum hemorrhage is not known. We sought to evaluate the association between latent phase oxytocin exposure and postpartum bleeding outcomes.

**Study Design**: Secondary analysis of a multicenter randomized controlled trial of nulliparous women with term singleton pregnancies comparing immediate vs. delayed pushing in the second stage of labor. In this study, the primary exposure was oxytocin dose, categorized as no, low, or high based on maximum dose (≥20 mU/min). Exclusions were incomplete data or outlier values. The primary outcome was postpartum hemorrhage (PPH), defined as estimated blood loss >500 mL (vaginal) or >1000 mL (cesarean). Secondary outcomes were the use of additional uterotonics, blood transfusion, and a composite. Log‐binomial regression was used to estimate risk ratios (RRs) and 95% confidence intervals (CIs). Adjusted models controlled for induction, timing of maximum dose (latent vs active phase), and prolonged first stage (>95^th^ percentile).

**Results**: Among 2404 patients in the parent trial, 1495 patients were included. High‐dose oxytocin was associated with a significantly increased risk of PPH (aRR = 2.2; 95% CI: 1.2–3.8; *p*  = 0.01) and the composite outcome (aRR  = 2.1; 95% CI: 1.2–3.6; *p*  = 0.01) when compared to no exposure; high‐dose exposure was associated with a non‐significant increase in additional uterotonic use (aRR = 2.5; 95% CI: 0.9–7.1; *p*  = 0.08). Low‐dose exposure was not associated with increased risk for any outcome. A similar pattern of results was observed when defining PPH as estimated blood loss >1000 mL and when adjusting for latent phase duration rather than prolonged first stage. Risk ratios for transfusion were not calculated due to low event counts.

**Conclusion**: High‐dose latent phase oxytocin exposure was associated with an increased risk of postpartum hemorrhage and composite maternal blood loss complications when compared to no exposure. Current PPH risk scoring systems do not include high latent phase oxytocin exposure, but perhaps they should.

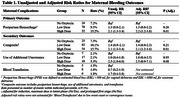

**10:30 AM – 12:00 PM**

## 325 | The association Between True Knot of Cord and Nuchal Cord and Long‐Term Offspring Cardiovascular Morbidity

Rachel Rappaport^1^; Eyal Sheiner^2^; Gali Pariente^3^; Tamar Wainstock^4^

^1^Soroka medical center, Soroka medical center, HaDarom; ^2^Soroka University Medical Center, Faculty of Health Sciences, Ben‐Gurion University of the Negev, beer sheva, HaDarom; ^3^Department of Obstetrics and Gynecology, Soroka University Medical Center, Ben‐Gurion University, beer sheva, HaDarom; ^4^Department of Epidemiology, Biostatistics and Community Health Sciences, Faculty of Health Sciences, Ben‐Gurion University of the Negev, Beer Sheva, HaDarom

**Objective**: Fetal hypoxic stress during development may disrupt vascular programming and endothelial function, potentially predisposing to long‐term cardiovascular morbidity. Nuchal cord and true knot of the umbilical cord can affect fetal cardiovascular function by compromising blood flow, thus leading to potential adverse perinatal outcomes. We aimed to study the long‐term cardiovascular morbidity in offspring born with confirmed true knot of cord or nuchal cord.

**Study Design**: A population‐based cohort study was conducted, evaluating the risk of offspring cardiovascular disease based upon the presence or absence of true knot of cord or nuchal cord at birth. Data for the incidence of cardiovascular morbidity of the offspring was extracted from community‐based clinics and hospitalization records. A Kaplan Meir survival curve was constructed to compare the cumulative incidence of cardiovascular morbidity between the study groups. Multivariable Cox regression models were used to assess the independent association between true knot of cord or nuchal cord and cardiovascular morbidity of the offspring, while controlling for confounders.

**Results**: The study included 232,476 newborns of whom 1.1% (*n* = 2537) were diagnosed with true knot of the umbilical cord and 18.2% (*n* = 42,398) were diagnosed with nuchal cording. The cumulative incidence of cardiovascular morbidity of the offspring didn't demonstrate a significant difference between offspring to women with and without true knot of cord or nuchal cord. The Cox proportional hazards models (Figure 1), controlling for mode of delivery, maternal age, gestational age at birth, and ethnicity, confirmed a lack of association between true knot of cord or nuchal cord and long‐term cardiovascular morbidity of the offspring (adjusted HR 0.93, 95% CI 0.71–2.10, *p* = 0.578 and adjusted HR 0.95, 95% CI 0.88–1.02, *p* = 0.212 for true knot of cord and nuchal cord, respectively).

**Conclusion**: In this large population‐based cohort, neither true knot of the umbilical cord nor nuchal cord at birth was associated with an increased risk of long‐term cardiovascular morbidity in offspring.

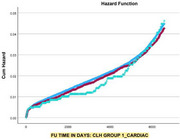

**10:30 AM – 12:00 PM**

## 326 | Preconception Counseling With a Multidisciplinary Cardio Obstetrics Team: What Does it Look Like?

Rachel L. Wiley^1^; Fayad Ammar^2^; Shayna Weinstein^3^; Lauren Tsai^2^; Cameron Salehi^4^; Erin Gallent^1^; Minhazur R. Sarker^1^; Laith Alshawabkeh^2^; Maryam Tarsa^1^

^1^Division of Maternal Fetal Medicine, Department of Obstetrics, Gynecology and Reproductive Sciences, University of California San Diego, San Diego, CA; ^2^Division of Cardiovascular Medicine, Department of Internal Medicine, University of California San Diego, San Diego, CA; ^3^New York Medical College, Valhalla, NY, Valhalla, NY; ^4^University of California San Diego School of Medicine, San Diego, CA

**Objective**: Preconception counseling (PCC) is a critical component of cardiac care for reproductive‐age individuals, yet estimates are only 1 in 10 at risk people receive PCC. This study aimed to characterize the delivery of PCC within a tertiary care center with a cardio‐obstetrics team.

**Study Design**: We conducted a retrospective cohort study of pregnant patients co‐managed by a multidisciplinary cardio‐obstetrics team at a single tertiary institution between 2020 and 2024. Inclusion criteria were nulliparous with pre‐existing cardiac disease, who established care prior to conception. PCC was defined as either a visit documenting discussion of pregnancy risks or avoidance of pregnancy with a MFM specialist or cardiology. Demographic data was collected, along with information on the content of PCC counseling.

**Results**: Of 198 pregnancies reviewed, 87 (51.4%) met inclusion criteria. Among these, 48 patients (55.2%) received PCC: 14 (29.2%) from both MFM and cardiology, 27 (56.3%) from cardiology alone, and 7 (14.5%) from MFM alone. Patients who received PCC were more likely to have private insurance (*p* = 0.04), higher pre‐pregnancy BMI (*p* = 0.02), and valvular disease (*p* = 0.01, Table 1). While overall cardiac risk stratification did not differ between those who did and did not receive PCC, patients with lower WHO or CARPREG II risk stratification were more frequently seen by cardiology than MFM (Table 2). MFM was more likely to recommend aspirin, genetic counseling, and prenatal vitamins, whereas cardiologists more often advised delaying pregnancy or updating imaging.

**Conclusion**: This study provides unique information about the real‐world delivery of cardiac PCC, indicating that inclusion of MFM increased discussion of pregnancy‐specific care. A multidisciplinary specialty team significantly increased rates of PCC, but still only half of eligible patients received PCC. These findings underscore the need for structured, multidisciplinary approaches to ensure equitable, comprehensive reproductive counseling and identifies gaps, especially in managing lower‐risk patients.

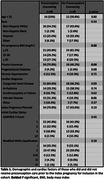

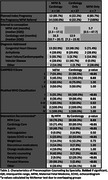

**10:30 AM – 12:00 PM**

## 327 | Widely Used, but Does it Deliver? A Closer Look at the AWHONN PPH Risk Score

Raina A. Meka^1^; Lori M. Stevenson^2^; Roxane Rampersad^3^; Megan L. Lawlor^3^; Amanda C. Zofkie^3^; Sydney M. Thayer^4^; Antonina I. Frolova^3^; Nandini Raghuraman^3^; Jeannie C. Kelly^4^

^1^Washington University School of Medicine, Washington University School of Medicine/ St. Louis, MO; ^2^Barnes‐Jewish Hospital, St. Louis, MO; ^3^Washington University School of Medicine, St. Louis, MO; ^4^Washington University School of Medicine, Washington University School of Medicine, MO

**Objective**: To evaluate the association between a widely used postpartum hemorrhage (PPH) risk assessment tool and clinically relevant hemorrhage outcomes, including blood loss, transfusion, and hemoglobin decline.

**Study Design**: This is a retrospective cohort study of all deliveries at a tertiary care center between January 2024 and July 2025. Patients were stratified into low, moderate, or high‐risk categories using our electronic medical record (Epic Systems) based on the Association of Women's Health, Obstetric, and Neonatal Nursing (AWHONN) PPH risk score. We compared the following outcomes across risk categories: estimated blood loss (EBL) ≥ 1000 mL, quantified blood loss (QBL) ≥ 1000 mL, any transfusion, and transfusion >1 unit. We also performed a subgroup analysis of hemoglobin decline in patients with a documented postpartum hemoglobin value who did not receive a transfusion. Discriminative performance was evaluated using the area under the receiver operating characteristic curve (AUC).

**Results**: Among 5540 deliveries, increasing PPH risk category was significantly associated with EBL and QBL ≥ 1000 mL, as well as increased rates of any transfusion (Table). However, discriminative performance for these outcomes was poor (AUCs: 0.60, 0.61, 0.63, and 0.69, respectively). In a subgroup of 1793 patients without transfusion and with documented postpartum hemoglobin, median hemoglobin drop was significantly greater in the low and high‐risk groups compared to the moderate group (Figure). Although hemoglobin drop ≥ 2 and ≥ 3 g/dL were significantly associated with risk category, the score failed to discriminate these outcomes better than chance (AUC 0.47 for both).

**Conclusion**: PPH risk assessment based on AWHONN scoring was statistically associated with blood loss, transfusion, and hemoglobin decline; however, the score's ability to predict adverse outcomes is poor. Despite statistical significance, the pattern of hemoglobin decline across risk categories is not clinically meaningful. These findings suggest that outcome‐based validation of hemorrhage risk tools is urgently needed.

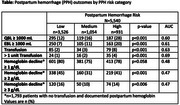

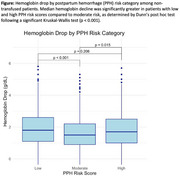

**10:30 AM – 12:00 PM**

## 328 | Unmasking Empathy and Color Evasion: A Mixed‐Methods Study of Providers’ Perceptions on Maternal Health Disparities

Ran Zhang^1^; Sheree Boulet^1^; Sierra Carter^2^; Jasmin Darville^3^; Myiera Seymour^1^; Simone Sanders^2^; Gina Northington^3^; Charisma Manley^3^; Alexis Kendall^4^

^1^Boston Medical Center, Boston, MA; ^2^Georgia State University, Atlanta, GA; ^3^Emory University, Atlanta, GA; ^4^Florida State University, Tallahassee, FL

**Objective**: Bias, racialized care, and institutional structures contribute to unequal experiences and outcomes among Black birthing people. Using a causal attribution framework, we examined obstetric providers’ perceptions and knowledge of maternal health disparity causes.

**Study Design**: We conducted a mixed‐methods study with obstetric providers (physicians and advanced practice providers) in Georgia from Aug 2023 to Jan 2024. Participants completed a 72‐item survey assessing beliefs and perceptions about causes of maternal health disparities, color evasion, and empathy. Respondents were classified by attribution of provider behavior as a cause of disparities (high vs low) and compared using chi‐square, Fisher's exact, t‐tests, or Wilcoxon rank‐sum tests. A subset of 26 respondents participated in seven focus groups using clinical vignettes to explore beliefs. Thematic and ideal‐type analyses identified emerging themes and typologies.

**Results**: Among 284 respondents (median age 41; 84.5% female), 82.3% were physicians, with 42% White and 13.9% Black. Compared to respondents with low provider attribution scores (*N* = 168), those with high scores (*N* = 112) included a higher proportion of Black providers (*p* < .05), reported greater empathy (*p*< .05), and were more likely to agree that their personal biases affect patient care (*p*< .05). Color evasion scores were high across groups. Focus groups identified three drivers of disparities: structural/systemic factors (e.g. patient blame culture, workflow issues); organizational constraints (e.g. care discontinuity, burnout); and interpersonal dynamics (e.g. rapport building, “two victims”‐providers perceived themselves and patients as system victims). Providers’ perceptions of racism and disparities fit five types: advocacy is healthcare, helpless doctor, knowledge giver, empathy as advocacy, and evasion by communication.

**Conclusion**: Findings suggest providers often use color evasion and empathy to disguise personal bias and perpetuate racist stereotypes. The typologies reflect varying approaches providers use to deflect responsibility for overcoming and addressing personal bias.

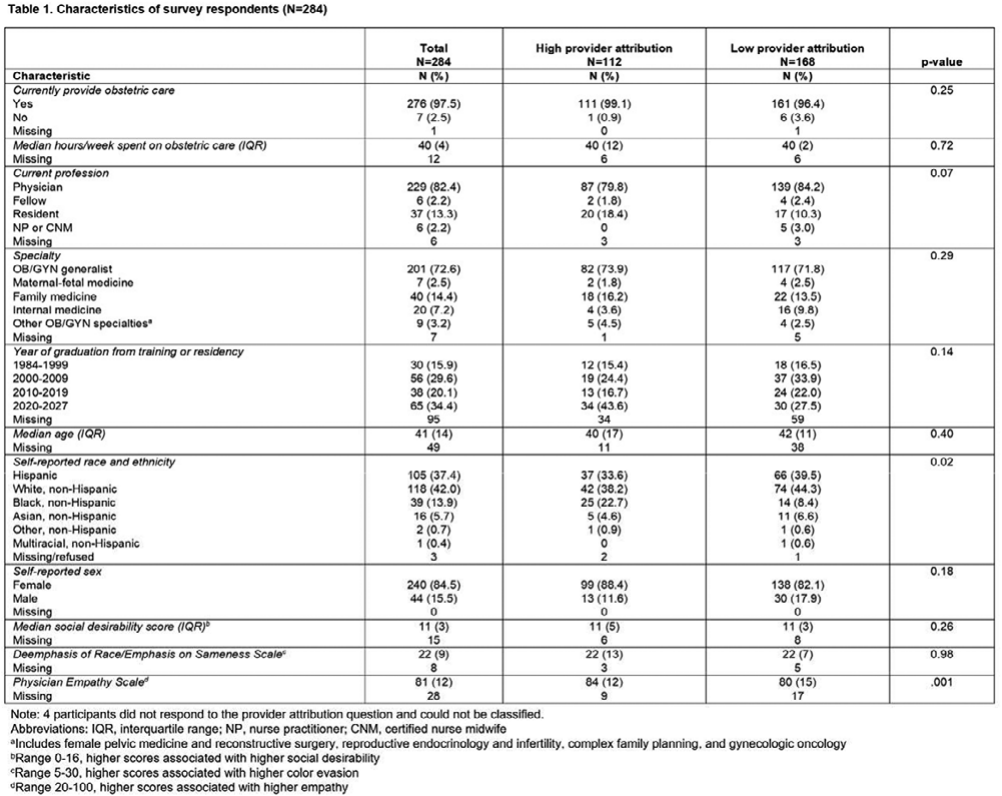

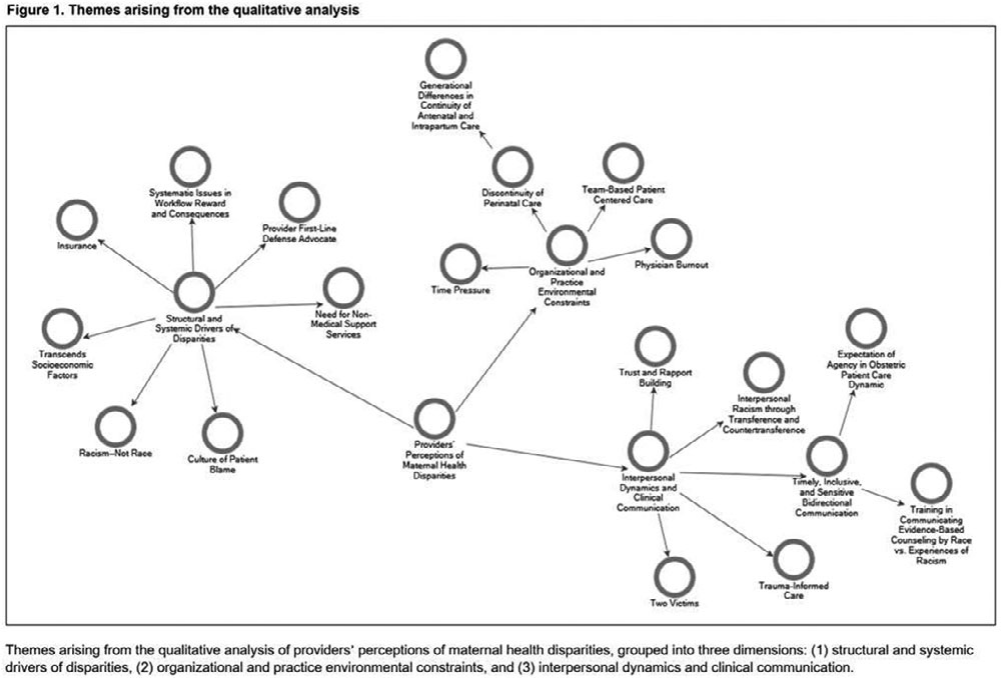

**10:30 AM – 12:00 PM**

## 329 | Clinical Chorioamnionitis Risk in Term GBS Patients With or Without Antibiotics Before Balloon Insertion

Raneen Abu Shqara^1^; Sabaa Knaneh^2^; Roni Jeremias Laskar^3^; Lior Lowenstein^4^; Enav Yefet^5^; Maya Frank Wolf^4^

^1^Galilee Medical Center, Nahariya, HaZafon; ^2^Tzafon Medical Center, Poriya, HaZafon; ^3^Galilee Medical Center, Nahariyya, HaZafon; ^4^Galilee Medical Center, Naharyia, HaZafon; ^5^Tzafon medical center, Poriya, HaZafon

**Objective**: To compare the risk of clinical chorioamnionitis among Group B Streptococcus (GBS)‐colonized pregnant patients who underwent cervical ripening with a catheter balloon, between those treated and not treated with prophylactic antibiotics prior to insertion of the catheter balloon.

**Study Design**: This is a retrospective study of term singleton pregnant women with confirmed GBS colonization who underwent induction of labor for medical indications during 2018–2023 at two tertiary centers. At one center, the patients received prophylactic intravenous ampicillin (2 g every 6h), starting 30 minutes prior to the catheter balloon insertion and continued until delivery. At the other center, routine prophylactic antibiotics were not administered; and antibiotics were initiated only at the onset of active labor. The primary outcome was clinical chorioamnionitis. Secondary outcomes included maternal postpartum infections and neonatal outcomes. Multivariate logistic regression was used to adjust for potential confounders.

**Results**: Included were 144 patients: 75 treated with prophylactic antibiotics and 69 not treated. Baseline maternal and obstetric characteristics were comparable. Among those treated compared to those not treated, the rates were lower of intrapartum fever (2.7% vs. 15.9%, *p* = 0.005) and clinical chorioamnionitis (0% vs. 8.7%, *p* = 0.002) (Figure). Statistically significant differences were not observed between the groups, in postpartum infections, cesarean delivery rates, or neonatal outcomes. In the multivariate analysis, not receiving prophylactic antibiotics prior to catheter balloon insertion was independently associated with an increased risk of clinical chorioamnionitis (*p* = 0.010) (Table).

**Conclusion**: Antibiotic prophylaxis prior to catheter balloon insertion in GBS‐colonized patients was associated with lower rates of maternal fever and clinical chorioamnionitis. Randomized controlled trials are warranted to confirm these findings and to guide clinical practice regarding the role of prophylactic antibiotic use during mechanical induction of labor.

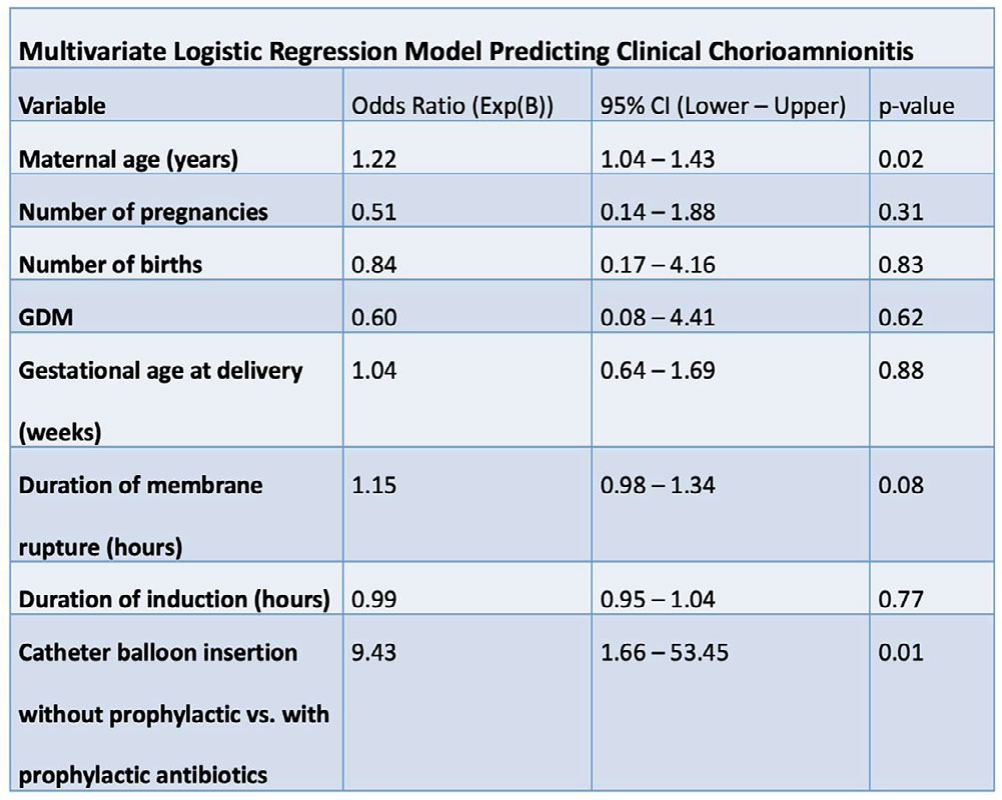

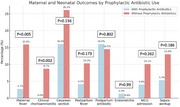

**10:30 AM – 12:00 PM**

## 330 | Clinical Interventions and Pregnancy Outcomes in Pregnancies Following Periviable Rupture of Membranes

Rebecca Crowe^1^; Julia Yerger^1^; Jessica Pittman^1^; Eliza R. McElwee^2^

^1^Medical University of South Carolina, Charleston, SC; ^2^Medical University of South Carolina, The Medical Univeristy of South Carolina, SC

**Objective**: The aim of this study was to characterize antenatal interventions and associated pregnancy outcomes in immediate subsequent pregnancies following a pregnancy affected by pre‐ and peri‐viable rupture of membranes (pPROM).

**Study Design**: This is a retrospective cohort study of immediate subsequent pregnancies following a pregnancy affected by pPROM from 2005–2024. We included patients with a prior pregnancy affected by pPROM at <25 weeks gestation and subsequent prenatal care and delivery at our institution. Subsequent pregnancy antenatal interventions and outcomes were evaluated and stratified by recurrent preterm birth (PTB). Chi Square was used for categorical variables and Kruskall Wallis as appropriate was used to compare continuous variables with significance at *p* < 0.05. A logistic regression was used to generate adjusted odds ratios (aOR) for the primary outcome of recurrent preterm birth.

**Results**: A total of 75 patients had a history of pPROM with subsequent pregnancy outcomes available for review during the study period. Of these, 64% (*n* = 48) had a subsequent term delivery and 36% (*n* = 27) had subsequent PTB. 72% (*n* = 54) had sonographic cervical length (CL) assessment and/or cerclage in subsequent pregnancies. When comparing subsequent term and PTB, there was no difference in cerclage frequency between groups (54.4 v 53.9%, *p* = 0.96). Patients with cervical shortening requiring urgent cerclage had increased rates of PTB (63%), compared to patients with a normal CL (30.8%) or those who underwent history indicated cerclage (25.9%), *p* = 0.04. There was no difference in odds of PTB in patients undergoing a history indicated cerclage compared to a referent group of patients with a normal CL (OR 0.79, 95% CI 0.18 ‐ 3.39).

**Conclusion**: Patients with a history indicated cerclage or a normal cervical length had similar rates of PTB following a pregnancy with pPROM <25w. The highest rates of PTB are in patients with cervical shortening prompting urgent cerclage. History indicated cerclage does not appear to cause harm and may provide benefit in patients with a history of pPROM.

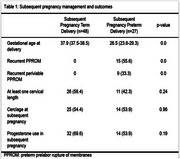

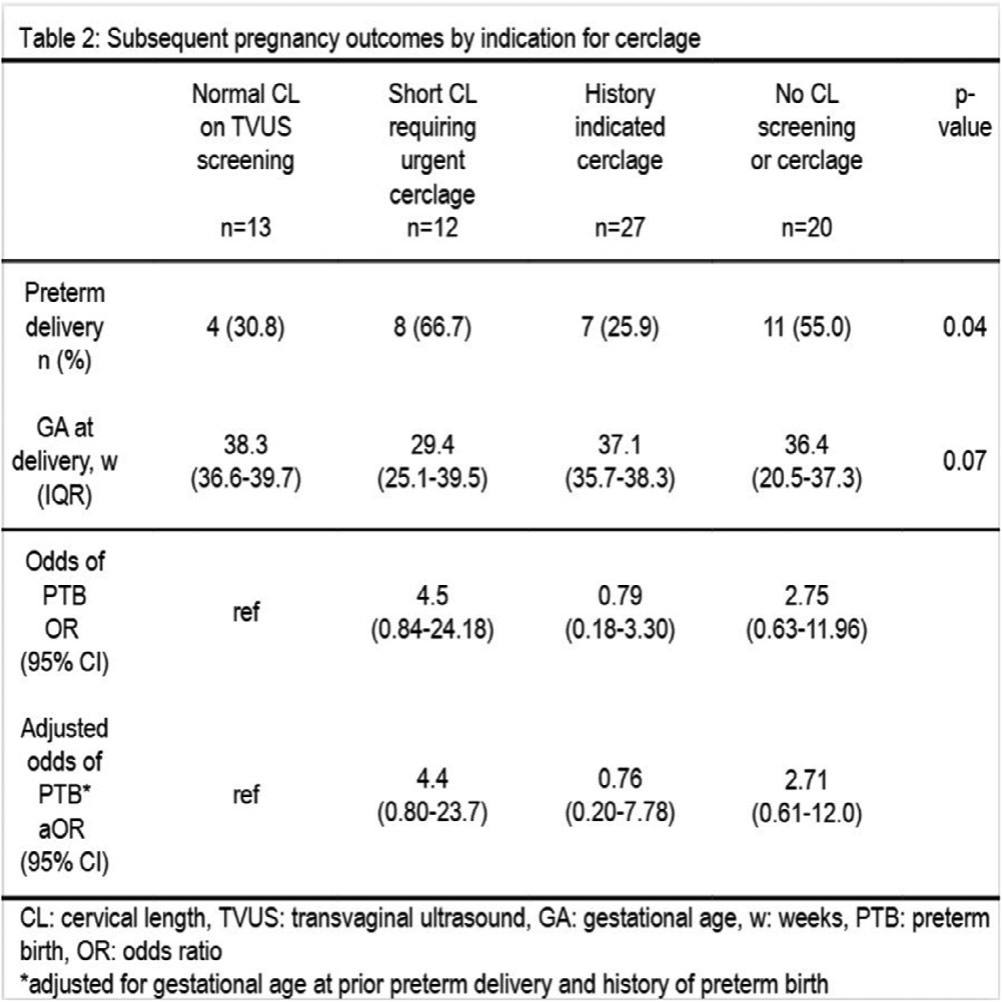

**10:30 AM – 12:00 PM**

## 331 | Impact of Structured Shoulder Dystocia Training on Rates of Neonatal Injuries at an Academic Institution

Leah Saylor^1^; Rebecca M. Dria^2^; Shannon M. Clark^3^; Luis D. Pacheco^1^

^1^University of Texas Medical Branch, Galveston, TX; ^2^UTMB Galveston, UTMB/Galveston, TX; ^3^University of Texas Medical Branch Galveston, UTMB/Galveston, TX

**Objective**: To reduce the number of neonatal injuries, namely clavicular fractures and brachial plexus injuries, associated with shoulder dystocia through structured education and simulation‐based training of OB/GYN residents and nursing.

**Study Design**: A multidisciplinary educational initiative was implemented over a 4‐month period (May‐August 2024) in response to a February 2024 NICU concern about rising rates of brachial plexus injuries and clavicular fractures in 2023. Interventions included assigned reading and video review on shoulder dystocia management at the start of each new 6‐week resident L&D rotation. In August 2024, didactic sessions and small‐group simulation stations focused on shoulder dystocia were conducted, with continued focus on hands‐on training through recurring faculty‐led shoulder dystocia drills involving OB/GYN residents and nursing.

**Results**: Ten months of delivery data before and after implementation were reviewed. The incidence of shoulder dystocia complicated by neonatal injury decreased from 0.45% to 0.18% of all deliveries (OR 2.5, 95% CI 0.84–8.97; *p* = 0.097). Notably, the number of deliveries complicated by shoulder dystocia increased from 63 pre‐intervention to 108 post‐intervention, yet the number of associated neonatal injuries (clavicular fractures and brachial plexus injuries) declined from 13 to 5 which corresponds to a 16% decline in neonatal injury in the setting of shoulder dystocia (OR 5.3, 95% CI 1.66–20.06; *p* < 0.01).

**Conclusion**: Structured and recurrent education, particularly hands‐on, simulation‐based training, was associated with an increased recognition of shoulder dystocia and a reduction in neonatal injury. These findings support the continued use of simulation‐based training to improve perinatal outcomes and support the application of this training model to other obstetric emergencies.

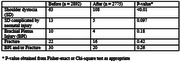

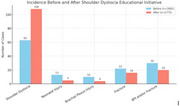

**10:30 AM – 12:00 PM**

## 332 | Maternal and Neonatal Outcomes by Cervical Status: A Secondary Analysis of the ARRIVE Trial

Rebecca Horgan^1^; Erkan Kalafat^2^; Marwan Ma'ayeh^1^; Maged M. Costantine^3^; George R. Saade^1^

^1^Eastern Virginia Medical School at Old Dominion University, Norfolk, VA; ^2^Koc University Hospital, Istanbul, Koc University Hospital, Istanbul, Istanbul; ^3^Ohio State University, Columbus, OH

**Objective**: The ARRIVE trial found that low‐risk nulliparous women induced electively at term had lower cesarean delivery and preeclampsia rates, but no difference in neonatal outcomes, compared to expectant management. Our objective was to assess the overall impact of induction on both maternal and neonatal outcomes, as well as how cervical status modifies this effect.

**Study Design**: This was a secondary analysis of the ARRIVE trial. We used multivariable multinomial regression to assess the association between labor induction and a three‐level categorical delivery outcome: uncomplicated vaginal delivery, uncomplicated cesarean section, or a composite of complicated vaginal or cesarean delivery. This composite complication outcome included, but was not limited to, maternal death, postpartum hemorrhage, uterine rupture, ICU admission, venous thromboembolism, and neonatal outcomes such as stillbirth, 5‐minute Apgar score less than 7, hypoxic‐ischemic encephalopathy, seizure, birth trauma, or mechanical ventilation. The analysis was stratified by Bishop score at randomization (0–2, 3–4, 5–6, and ≥7). Models were adjusted for maternal age, body mass index, race, ethnicity, and lifestyle factors.

**Results**: Among 5968 participants, 3005 pregnancies were induced and 2963 were managed expectantly. Among women with a Bishop score of 0–2, labor induction was associated with a significantly lower rate of uncomplicated cesarean delivery compared to expectant management (18.7% vs. 23.0%, *p* = 0.034). For those with a Bishop score of 3–4, induction was associated with a lower rate of the composite outcome of complicated vaginal or cesarean delivery (13.8% vs. 17.5%, *p* = 0.020). Across all Bishop score categories, labor induction was consistently associated with an approximately 3% absolute increase in the rate of uncomplicated vaginal delivery (Figure 1).

**Conclusion**: In low‐risk nulliparous women, labor induction at 39 weeks is associated with more favorable maternal and neonatal outcomes across multiple Bishop score categories. These findings support the benefits of induction regardless of cervical status.

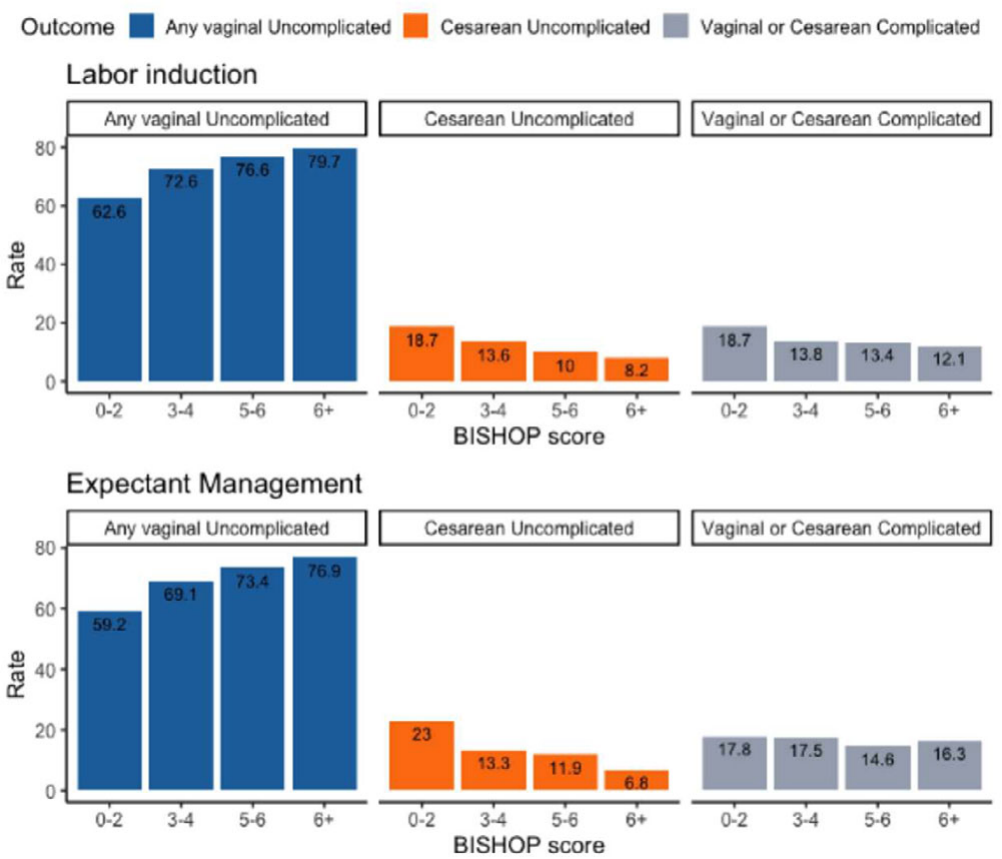

**10:30 AM – 12:00 PM**

## 333 | First‐Trimester Placental Volume is Associated With Preeclampsia and Small for Gestational Age Birthweight

Rebecca Horgan^1^; Elena Sinkovskaya^1^; Tracey DeYoung^1^; Yara Hage Diab^2^; Erkan Kalafat^3^; Alfred Abuhamad^2^; George R. Saade^1^

^1^Eastern Virginia Medical School at Old Dominion University, Norfolk, VA; ^2^Eastern Virginia Medial School at Old Dominion University, Norfolk, VA; ^3^Koc University Hospital, Istanbul, Koc University Hospital, Istanbul, Istanbul

**Objective**: To evaluate the association between first‐trimester placental volume and subsequent development of preeclampsia (PE) and small for gestational age (SGA) birthweight.

**Study Design**: Pregnant women at < 13 + 6 weeks’ gestation without fetal, umbilical cord or placental abnormalities were enrolled in this longitudinal cohort as part of the human placenta project. A 3D placental volume was obtained in the first trimester using VOCAL technique. Pregnancy outcomes were categorized into (1) PE only, (2) SGA only (birthweight <10%ile), or (3) both (PE+SGA) which were compared to unaffected pregnancies. Placental volume was analyzed both continuously and categorically (≤10th, 10th–< 25th, and ≥25th percentiles). A cause‐specific Cox proportional hazards model was used to assess the risk of each outcome. Survival analysis was performed using gestational age at delivery as the time scale.

**Results**: 368 patients had first trimester placental volume obtained. 29 patient had PE only, 27 had FGR only, 7 had FGR and PE, and 305 pregnancies were unaffected. Smaller placental volume was associated with the PE+SGA phenotype (HR: 0.26, 95% CI: 0.13–0.50, *p* < 0.001), but not with isolated PE (HR: 0.75, 95% CI: 0.51–1.09, *p* = 0.13) or isolated SGA (HR: 0.96, 95% CI: 0.64–1.43, *p* = 0.84) (Figure 1). Cumulative incidence plots demonstrated earlier delivery among those with the smallest placental volume <10th percentile.

**Conclusion**: First trimester placental volume is strongly associated with subsequent development of PE with concurrent SGA. First‐trimester placental measurements may aid in identifying pregnancies at highest risk for early‐onset, multi‐system placental disease.

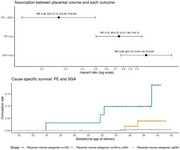

**10:30 AM – 12:00 PM**

## 334 | Preconception GLP‐1 Receptor Agonist use and the Risk of Postpartum Depression and Anxiety

Robert E. Jones; Tina Hsieh; Mckenzi Thompson; Ai‐ris Y. Collier

Beth Israel Deaconess Medical Center, Boston, MA

**Objective**: Centrally‐acting anti‐obesity medications (AOMs) have been linked to increased risks of anxiety, depression, and suicidality. While current evidence regarding mental health outcomes in non‐pregnant individuals using GLP‐1 receptor agonists (GLP‐1RAs) is reassuring, the impact on pregnant individuals remains unstudied. This study evaluates whether GLP‐1RA use before or during pregnancy affects postpartum mental health outcomes.

**Study Design**: Using the TriNetX U.S. Collaborative, we identified deliveries from adult women with a BMI ≥ 25 between May 2006–May 2024 with a GLP‐1RA or an alternative FDA‐approved AOM prescription (orlistat, phentermine‐topiramate, and bupropion‐naltrexone). Use was categorized as a prescription either within 1 year preconception only or during pregnancy. After 1:1 propensity‐score matching on demographics, comorbidities, healthcare utilization, and substance use, we calculated risk ratios (RR) and 95% confidence intervals (CIs) of mental health outcomes within 1 year postpartum between GLP‐1RA and alternative AOM users.

**Results**: We identified 792 individuals with GLP‐1 RA preconception use and 285 with use during pregnancy, each matched to an alternative AOM user (Table 1). GLP‐1RA preconception use was associated with increased risks of incident (RR 1.53, 95% CI 1.004–2.316) and recurrent postpartum anxiety (1.22, 95% CI 1.050‐1.421) (Figure 1). GLP‐1RA use during pregnancy was associated with a similar, though non‐significant, increase in incident postpartum anxiety (RR 1.80, 95% CI 0.983–3.286). No differences were observed in recurrent postpartum anxiety or incident or recurrent postpartum depression. Other mental health outcomes (suicidality, bipolar disorder, OCD, and PTSD) were too rare for analysis.

**Conclusion**: Preconception GLP1‐RA use is associated with increased risk of postpartum anxiety, but not depression. The lack of significant association with use during pregnancy suggests risk may be influenced by duration of discontinuation. Further research is needed on mental health outcomes after GLP‐1RA discontinuation in pregnant individuals.

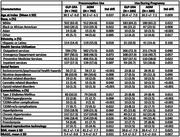

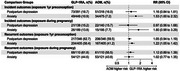

**10:30 AM – 12:00 PM**

## 335 | Vaginal Delivery in Discordant Twins With Larger Second Twin: No Increased Risk of Adverse Outcomes

Roie Alter^1^; Inbal Akavian^1^; Shlomo Yahalomy^2^; Joshua Isaac Rosenbloom^3^; Aharon Tevet^2^; Hadar Rosen^1^

^1^Hadassah Ein‐karem hospital, Obstetrics and gynecology department, Jerusalem, Jerusalem, Yerushalayim; ^2^Department of Obstetrics and Gynecology, Hadassah Hebrew University Medical Center, Ein Kerem, Jerusalem, Yerushalayim; ^3^Hadassah Medical Center and Hebrew University of Jerusalem, Jerusalem, Israel, Jerusalem, Yerushalayim

**Objective**: Vaginal delivery is a well‐established and safe mode of birth for twin pregnancies under appropriate conditions. However, when the second twin is larger, concerns arise about technical difficulty, neonatal morbidity, and combined delivery. This study evaluates whether these concerns are supported by assessing cesarean rates and neonatal outcomes in larger twin B discordancy undergoing a trial of labor

**Study Design**: This retrospective cohort study included all diamniotic twin deliveries ≥32 weeks with a cephalic first twin at a tertiary center from 1/2019 to 04/2025. Elective cesareans were excluded. Larger B Discordancy was defined as >20% weight difference with a larger second twin; concordant twins (–20% < D < 20%) were controls. The primary outcome was mode of delivery. Secondary outcomes included cesarean delivery for the second twin and 5‐minute Apgar <7

**Results**: Among 1017 twin deliveries, 885(87.0%) were concordant and 53(5.2%) were discordant with a larger second twin. The distribution of vertex–non‐vertex presentations was similar between groups (42.8 vs. 41.5%, *p* = .851). Vaginal delivery was common across all twins but more frequent in vertex–vertex compared to vertex–non‐vertex pairs (77.9% vs. 69.5%, *p* = .003).

Cesarean delivery for both twins was more common in discordant pairs (34 vs. 22.3%, *p* = .049), yet cesarean delivery for the second twin alone remained rare and was not significantly different between groups (1.9 vs. 1.1%, *p* = .474). Similarly, 5‐minute Apgar <7 rates for both first and second twins were low and comparable (0 vs. 0.9% for Twin A; 0 vs. 1.4% for Twin B; both *p* = 1).

In logistic regression, both discordancy (OR = 1.84, *p* = .043) and non‐vertex second twin (OR = 1.47, *p* = .015) were independently associated with emergency cesarean

**Conclusion**: Although discordant twins with a larger second twin had higher overall cesarean rates, this was likely due to events during labor and not delivery difficulty. The consistently low rates of second twin cesarean and reassuring neonatal outcomes support that vaginal delivery is a safe and viable option in well‐selected discordant larger second twin

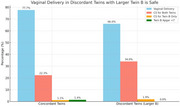

**10:30 AM – 12:00 PM**

## 336 | Association of Cerebroplacental Ratio and Fetal Heart Morphology and Function in the 3rd Trimester

Rommy H. Novoa^1^; Lina Youssef^1^; Kenny Araujo^2^; Lea Testa^2^; Ayako Nakaki^2^; Rosa Casas^3^; Leticia Benitez^1^; Irene Casas^2^; Mariona Genero^1^; Roger Borras^2^; Sara Castro‐Barquero^4^; Ramon Estruch^3^; Bart Bijnens^5^; Gabriel Bernardino^5^; Francesc Figueras^6^; Francesca Crovetto^7^; Eduard Gratacós^8^; Fatima Crispi^9^

^1^BCNatal–Barcelona Center for Maternal‐Fetal and Neonatal Medicine (Hospital Clínic and Hospital Sant Joan de Deu), IDIBAPS, University of Barcelona, Barcelona, Catalonia; ^2^BCNatal–Barcelona Center for Maternal‐Fetal and Neonatal Medicine (Hospital Clínic and Hospital Sant Joan de Deu), IDIBAPS, University of Barcelona, Barcelona, Spain., Barcelona, Catalonia; ^3^Institut d'Investigacions Biomèdiques August Pi i Sunyer (IDIBAPS), Barcelona, Spain., Barcelona, Catalonia; ^4^BCNatal Fetal Medicine Research Center, Barcelona, Catalonia; ^5^BCN Medtech, Department of Information and Communication Technologies, Universitat Pompeu Fabra, Barcelona, Spain., Barcelona, Catalonia; ^6^BCNatal–Barcelona Center for Maternal Fetal and Neonatal Medicine Hospital Sant Joan de Déu and Hospital Clínic, University of Barcelona, Barcelona, Catalonia; ^7^BCNatal–Fetal Medicine Research Center (Hospital Clínic and Hospital Sant Joan de Déu), Institut d'Investigacions Biomèdiques August Pi i Sunyer (IDIBAPS), Barcelona, Catalonia; ^8^Hospital Clínic Barcelona, BARCELONA, Catalonia; ^9^Hospital clinic Barcelona, BARCELONA, Catalonia

**Objective**: To investigate the association of cerebroplacental ratio (CPR) and fetal cardiac function independently from estimated fetal weight (EFW) in fetuses from high‐risk pregnancies.

**Study Design**: Secondary analysis of the IMPACT‐BCN randomized clinical trial including 913 singleton pregnancies at high‐risk for small for gestational age newborns. Fetal ultrasound was performed at 33–34 weeks of gestation including echocardiography and CPR (dividing the pulsatility indices of the middle cerebral artery by the umbilical artery). CPR was considered abnormal below 5^th^ centile. At delivery, N‐terminal pro B‐type natriuretic peptide (NT‐proBNP) was measured in cord blood and considered high if above 90th centile (1668 pg/mL).

**Results**: Irrespectively from estimated fetal weight, fetuses with abnormal CPR showed a higher proportion of high NT‐proBNP (20.0% vs. 7.7%, *p* = 0.007), smaller cardiac area (25.3 centile (IQR 7.5; 52.9) vs. 36.5 (14.2; 65.8), *p* = 0.024), thinner myocardial septal wall (median 51.5 centile (IQR 24.4; 75.6) vs. 67.7 (39.3; 89.3), *p* = 0.005) and higher proportion of abnormal tricuspid annular plane systolic excursion (28.2% vs. 14.2%, *p* = 0.003) as compared with those with normal CPR. When subdividing the population according to EFW and CPR, those subgroups with abnormal CPR showed the strongest association with abnormal cord blood NT‐proBNP (subgroups: fetuses with EFW < p3 and CPR < p5 66.7% vs. EFW≥p3 and CPR < p5 17.0% vs. EFW < p3 and CPR≥ p5 7.1% vs. EFW≥p3 and CPR≥p5 7.7%, *p* = 0.005) (Figure 1).

**Conclusion**: CPR is associated with worst fetal cardiac function, independently from estimated fetal weight.

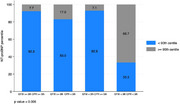

**10:30 AM – 12:00 PM**

## 337 | Early Response to Treatment of Iron Deficiency Anemia and Association with Perinatal Outcomes

Rupsa C. Boelig^1^; Michael Georgieff^2^; Simal Thind^3^; Mrutyunjaya Bellad^4^; Manjunath Somannavar^4^; Sudhir Bhandari^5^; Sudhir Mehta^5^; Seema Mehta^5^; Dharmesh Sharma^5^; Yogesh Kumar^4^; Umesh Charantimath^4^; Amaresh Patil^4^; Umesh Ramadurg^6^; Radha Sangavi^7^; Praveen Patil^7^; Subarana Roy^8^; Phaniraj Vastrad^8^; Chander Shekhar^3^; Benjamin Leiby^3^; Rebecca Hartman^3^; Zubair H. Aghai^9^; Ashalata Mallapur^6^; Richard Derman^3^

^1^Sidney Kimmel Medical College, Thomas Jefferson University, Philadelphia, PA; ^2^University of Minnesota, Minneapolis, MN; ^3^Thomas Jefferson University, Philadelphia, PA; ^4^KLE Academy of Higher Education and Research J N Medical College, Belagavi, Karnataka; ^5^Sawai Man Singh Medical College, Jaipur, Rajasthan; ^6^S Nijalingappa Medical and HSK Hospital and Research Center, Bagalkote, Karnataka; ^7^Raichur Institute of Medical Sciences, Raichur, Karnataka; ^8^Model Rural Health Research Unit, Sirwar, Karnataka; ^9^Nemours Children's Health, Philadelphia, PA

**Objective**: We aimed to determine if initial response to iron therapy was associated with reduced risk of stillbirth and other adverse outcomes in pregnant singletons with moderate iron deficiency anemia (IDA). Iron therapy will first restore hemoglobin and then replete iron stores (ferritin). There is limited data on how repletion of iron/Hb relates to perinatal outcomes.

**Study Design**: This is a secondary analysis of a multi‐center randomized controlled trial in India comparing single dose intravenous (IV) iron to oral iron for moderate IDA (Hb 7.0–9.9 g/dL) at 14–17 weeks gestation. The primary outcome for this analysis was stillbirth. The predictors of interest were maternal hemoglobin and ferritin at 20–24 weeks gestation, after randomization/treatment (14–17 weeks). Secondary outcomes were low birthweight (LBW), small for gestational age infants (SGA), and early preterm birth (PTB) <34 weeks. Poisson regression with robust standard errors was used to evaluate the relationship between each outcome and the predictors, adjusting for age, BMI, parity, and study site. Two‐sided alpha = 0.05 used for all analyses.

**Results**: 4252 participants were included; 1421, 1424, and 1407 randomized to IV ferric derisomaltose, ferric carboxymaltose, and oral iron respectively. There was a progressively reduced risk of stillbirth (*p* < 0.0001), early PTB (*p* = 0.0009), LBW (*p* = 0.0016), and SGA (*p* = 0.0072) as Hb approached normal range (11.0 g/dL) after treatment (Figure). A U‐shaped relation was observed with very high Hb trending towards increased risk of adverse outcomes. There was a similar trend between low ferritin and risk of LBW, (*p* = 0.058).

**Conclusion**: In pregnancies with moderate IDA, early improvement (20–24 weeks gestation) of hemoglobin to a normal range is associated with reduced rates of stillbirth, LBW, SGA, and early PTB. Very high Hb (≥13) likely reflects pathophysiology of hemoconcentration relating to placental insufficiency, as has been previously reported, rather than over‐correction of anemia. Our findings highlight the importance of early identification and early, adequate treatment of IDA in pregnancy

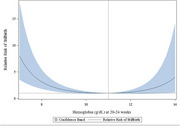

**10:30 AM – 12:00 PM**

## 338 | Isolated Small Abdominal Circumference and Timing of Delivery: Prognostic Implications for Perinatal Outcomes

Ruthly François‐Zafka; Katelyn J. Rittenhouse; Nicole Davis; Rebecca Ritter; Yuri Sebastião; Jeffrey S.A. Stringer; Elizabeth M. M. Stringer

University of North Carolina at Chapel Hill, Chapel Hill, NC

**Objective**: Expanding the definition of fetal growth restriction to include abdominal circumference (AC) <10th percentile aims to improve risk detection. We compared perinatal outcomes in pregnancies with isolated small AC (iAC), estimated fetal weight <10th percentile (EFW10), and EFW ≥ 10th percentile (EFW10+); evaluated the influence of delivery timing; and assessed predictive performance.

**Study Design**: We assembled a retrospective cohort of singleton, non‐anomalous pregnancies with ≥ 1 third trimester (≥ 28 weeks) growth ultrasound performed between 2017 and 2024 at a single academic center. A composite outcome (stillbirth, fetal demise, small for gestational age, NICU stay, Apgar score at 5 minutes <7, or C‐section) was compared using logistic regression models with generalized estimating equations, adjusting for maternal demographics, comorbidities, and gestational age. The influence of delivery timing was evaluated with stratified analyses. Predictive performance was assessed via ROC curves and AUC.

**Results**: iAC and EFW10 were identified in 2.9% (*n* = 540) and 7.1% (*n* = 1322) of 18,771 pregnancies, respectively. The composite outcome occurred in 63.3% of iAC, 77.5% of EFW10, and 40.4% of EFW10+ groups. Compared to EFW10+, iAC was associated with increased odds of adverse outcomes [adjusted OR 3.07 (95% CI: 2.41–3.92)], though lower than EFW10 [OR 8.70 (6.93–10.91)]. Compared to delivery at 37 weeks, delivery at 38–39 weeks had higher odds of adverse outcomes in both iAC and EFW10 groups. Although ROC curve analysis showed statistically significant lower AUC for iAC (0.67 vs. 0.69, *p* < 0.001), both iAC and EFW10 had high sensitivity (98%) but low specificity (4% vs. 12%).

**Conclusion**: These findings highlight the increased risk of adverse perinatal outcomes associated with isolated small AC, support increased fetal surveillance, and suggest that delivery timing may mitigate poor outcomes. Despite high sensitivity, the low specificity of iAC limits its standalone utility as a screening tool and underscores the need for careful interpretation alongside clinical context.

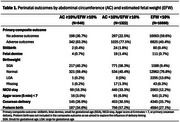

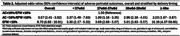

**10:30 AM – 12:00 PM**

## 339 | Adverse Birth Outcomes Among American Indian and Alaska Native Women Living on Reservations

Ryan Duggal^1^; Alexandra Sundermann^2^; Michael Kramer^3^

^1^University of California San Francisco, University of California San Francisco, CA; ^2^Vanderbilt University Medical Center, Vanderbilt University Medical Center, TN; ^3^Mercer University School of Medicine, Mercer School of Medicine, GA

**Objective**: To compare adverse birth outcomes (ABOs) among American Indian and Alaska Native (AI/AN) women living on reservations with non‐indigenous people living in the same counties.

**Study Design**: Using 2018–2022 restricted natality data from the National Vital Statistics System (NVSS) the following ABOs were analyzed: maternal morbidity, preterm birth, low birth weight, and NICU admission. By linking Public Use Microdata Areas (PUMAs) to counties and reservations using 5‐year American Community Survey (ACS) microdata, geographic shapefiles, and age‐specific fertility rates, births were estimated among AI/AN women living on a reservation. PUMAs were mapped to county race‐specific population data to estimate the probability that someone identifying as AI/AN lived on the reservation; using a data‐driven probability threshold, each AI/AN woman with a birth was determined to be living either on or off reservation in each county. Modified Poisson regression was used to estimate crude and adjusted relative risks (aRRs) for the association between ABOs and reservation status.

**Results**: There were 4,091,162 births in 548 counties containing reservations included in this cohort. Of these, 35,778 women were identified as AI/AN with a high probability of living on a reservation. Maternal morbidity and preterm birth for AI/AN women living on reservations were significantly higher (aRR 2.45, 95% confidence intervals [CI] 2.24–2.69; aRR 1.18, 95% CI 1.15–1.21) in both unadjusted and adjusted analysis than for non‐AI/AN women living in the same counties. There was no difference in the unadjusted or adjusted risk for low birth weight or NICU admission (aRR 0.96, 95% CI 0.92–1.00; aRR 0.99, 95% 0.95–1.02) between the groups.

**Conclusion**: AI/AN women living on reservations in this cohort had higher rates of maternal morbidity and preterm birth than non‐AI/AN women in the same counties but not living on reservations. These disparities highlight the urgent need for targeted, community‐informed maternal health interventions on reservations.

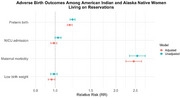

**10:30 AM – 12:00 PM**

## 340 | Curriculum Variability in Maternal‐Fetal Medicine Fellowship Programs

Ryan D. Farias; Christopher S. Ennen; Adam J. Schettler

University of Virginia School of Medicine, Charlottesville, VA

**Objective**: The Accreditation Council for Graduate Medical Education (ACGME) and the American Board of Obstetrics & Gynecology (ABOG) guidelines for Maternal‐Fetal Medicine (MFM) fellowship curricula specify rotation requirements for only 22 out of the 36 months of training. This offers considerable flexibility, but may also impact the baseline skill and knowledge of graduating fellows. The purpose of this study was to review publicly available curriculum data from fellowship program websites and compare how time is allocated across clinical and research rotations.

**Study Design**: This cross‐sectional descriptive study reviewed curriculum information from websites of all 111 ACGME‐accredited MFM fellowship programs in the United States. Data was collected on the number of months assigned to clinical and research rotations, as well as the structure of research time. Descriptive statistics were calculated including median, interquartile range (IQR), mode, range, and median deviation from ACGME minimum requirements (Table 1).

**Results**: 55% of program websites included at least some information on specific clinical rotations and 60% described the structure of the research curriculum. Ultrasound showed the greatest median deviation from ACGME requirements, with a +3‐month difference. Most programs (78%) distributed research time in blocks across the fellowship. Research time ranged from 12 to 20 months, and clinical rotation lengths also varied widely (Table 1).

**Conclusion**: There is significant variety in MFM fellowship curricula structure, particularly related to time allocated to clinical and research rotations. However, a substantial proportion of programs do not provide this information on their websites. Given the flexibility in curriculum structure, it is important for program websites to include curriculum information to help guide applicants in applying to programs aligned with their training goals. National organizations could use this data and post‐fellowship experiences to adjust requirements.

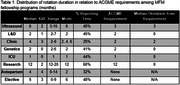

**10:30 AM – 12:00 PM**

## 341 | Two is not Better Than One Balloon for Cervical Ripening

Ryan Findlay^1^; Alfarooq Y. El‐Eishy^2^; Gustavo S. Hernandez Martinez^3^; Rachel Hartman^4^; Benjamin Spires^5^; Karin A. Fox^6^

^1^Division of Maternal Fetal Medicine, The University of Texas Medical Branch Health, League City, TX; ^2^University of Texas Medical Branch at Galveston, University of Texas Medical Branch at Galveston, TX; ^3^The University of Texas Medical Branch, Galveston, TX; ^4^University of Texas Medical Branch, University of Texas Medical Branch, TX; ^5^University of Texas Medical Branch, University of Texas Medical Branch Galveston, TX; ^6^Division of Maternal Fetal Medicine, The University of Texas Medical Branch Health, Galveston, TX

**Objective**: Cervical ripening with single‐balloon catheters is safe and effective. Shorter balloon dwell time (6 vs.12 h) shortens time from insertion to delivery without increasing cesarean rates, but optimal management after removal when the cervix remains unfavorable is unclear. Sequential placement of a second balloon has been described, but without supporting data. We hypothesized this may increase risk of cesarean delivery (CD) and infection.

**Study Design**: In this retrospective cohort study, we reviewed records of term gravidas (≥37 weeks) who underwent cervical balloon ripening at our institution between January 1, 2020, and January 1, 2025. Patients with inadequate cervical dilation (≤3 cm after initial foley balloon) were included. They were grouped by single (Group 1) or two sequential (Group 2) balloon placements, matched 3:1. The study was powered to detect a 15% increase in CD and intra‐amniotic infection rates, treated as independent primary outcomes. Secondary outcomes included time to delivery and postpartum hemorrhage. Multivariable logistic regression adjusted for maternal age, BMI, gestational age, and initial bishop score. Significance was defined as *p* < 0.05.

**Results**: During the study period, 799 participants met inclusion criteria, with 582 in group 1 and 217 in group 2. The CD (19.8% vs. 56.2% *p* < 0.0001) and chorioamnionitis (15.3% vs. 35.5%, p, 0.0001) rates were significantly lower in group 1. On multiple logistic regression, second balloon placement was independently associated with increased odds of CD (aOR 2.7, 95% CI 1.8–4.1) and intra‐amniotic infection (aOR 1.8, 95% CI 1.2–2.9). The incidence of postpartum hemorrhage was higher in group 2 (8.3% vs. 3.3%, *p* = 0.003), though this was not significant after adjusting for mode of delivery.

**Conclusion**: Our findings align with previous studies that suggest safety and efficacy of shorter balloon duration time. Based on our data, we do not recommend placement of a second sequential cervical ripening balloon, as it is associated with significantly higher odds of cesarean delivery and chorioamnionitis, and increased risk for harm.

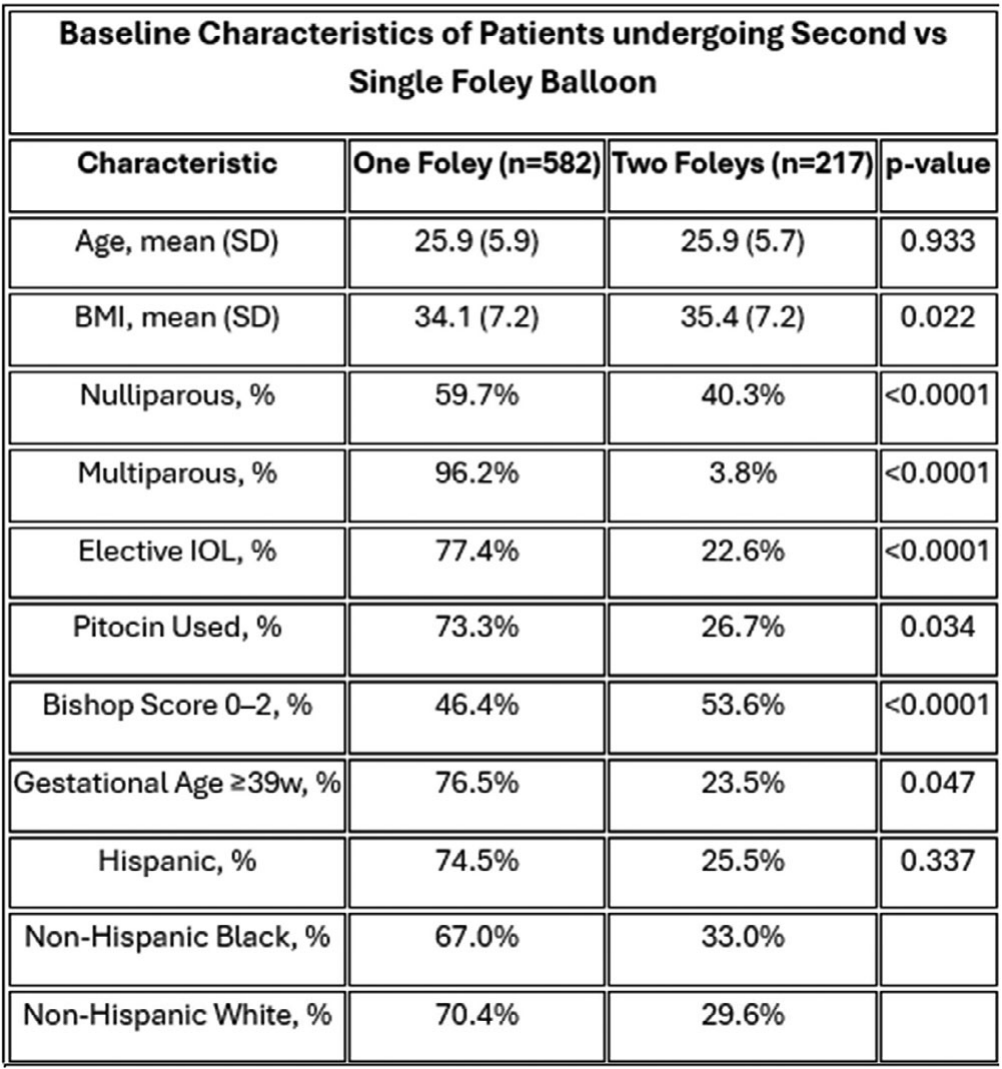

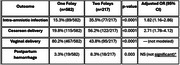

**10:30 AM – 12:00 PM**

## 342 | Variability in Obstetric Ultrasound Time: Influence of Exam Type, BMI, and Sonographer Experience

Salimah Navaz Gangji^1^; Juliana Martins^2^; Diana Aboukhater^3^; Maya Vishna^4^; Tetsuya Kawakita^5^; Alfred Abuhamad^1^

^1^Eastern Virginia Medial School at Old Dominion University, Norfolk, VA; ^2^Macon and Joan Brock Virginia Health Sciences at ODU, Virginia Beach, VA; ^3^University of Toledo Health, Toledo, OH; ^4^Macon & Joan Brock Virginia Health Sciences Eastern Virginia Medial School at Old Dominion University, Norfolk, VA; ^5^Eastern Virginia Medical School Department of Obstetrics and Gynecology, Macon & Joan Brock Virginia Health Sciences at Old Dominion University, Norfolk, VA

**Objective**: To determine the average scan duration of commonly performed obstetric ultrasound exams in a tertiary maternal‐fetal medicine (MFM) unit and assess whether maternal body mass index (BMI) and sonographer experience influence scan time

**Study Design**: This retrospective cohort included diagnostic obstetric ultrasounds performed at a single academic MFM unit from 2023–2024. Exams were grouped by clinical indication and gestational age: dating (6–10 weeks), first trimester (11–14 weeks), anatomy (18–22 weeks), fetal echocardiogram (22–24 weeks), and second/third trimester growth/Doppler scans (24–38 weeks). Scan duration was derived from image time‐stamps and log‐transformed. Linear regression estimated adjusted geometric mean ratios (GMRs).

**Results**: Among 891 scans (Dating: 48; First trimester: 50; Anatomy: 495; Echo: 98; Second/third trimester: 200), median scan time varied widely (Dating: 7.2 min [IQR 5.3–10.1]; First trimester: 18.4 [12.2–22.6]; Anatomy: 29.9 [25.8–35.3]; Echo: 28.2 [22.6–37.2]; Second/Third trimester: 12.6 [10.0–17.3]). Compared to dating scans, adjusted scan time was 2.22× longer for first trimester (95% CI 1.85–2.66), 3.98× for anatomy (95% CI 3.48–4.55), 4.41× for echo (95% CI 3.77–5.16), and 1.72× for second/third trimester (95% CI 1.49–1.97), all *p* < 0.001. BMI independently prolonged scan time by 2% per 5‐unit increase (GMR 1.02, 95% CI 1.00–1.03, *p* = 0.025). Multiple gestations were associated with 42% longer duration (GMR 1.42, 95% CI 1.19–1.70, *p* < 0.001). Compared to sonographers with < 5 years of experience, those with 5–9 years and ≥10 years had 21% (GMR 0.79, 95% CI 0.71–0.88) and 19% (GMR 0.81, 95% CI 0.76–0.86) shorter exam times, respectively (both *p* < 0.001). For fetal echocardiograms, scan time decreased 6.5% per additional year of experience (*p* < 0.001).

**Conclusion**: Obstetric ultrasound scan time varies substantially by exam type and is independently influenced by maternal BMI and sonographer experience. Incorporating these factors into scheduling models may enhance workflow and reduce staff burden in high‐volume MFM units.

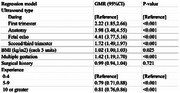

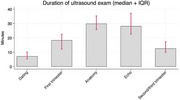

**10:30 AM – 12:00 PM**

## 343 | Term Management of the Suspected Large for Gestational Age Fetus

Samantha Krueger; Rohan D'Souza; Liz Darling; Giulia M. Muraca

McMaster University, Hamilton, ON

**Objective**: Our aim was to compare maternal and neonatal outcomes following labor induction versus expectant management of full‐term suspected large‐for‐gestational‐age (LGA) fetuses.

**Study Design**: This population‐based retrospective cohort study obtained data on births in Ontario, Canada (2012–2021) from the provincial perinatal registry linked to several health administrative databases via ICES. We included individuals with full‐term, singleton pregnancies with suspected LGA and excluded those with contraindications to vaginal delivery. The exposure was labor induction for the primary indication of LGA compared with expectant management at each gestational week from 37–41 weeks of gestation. Outcomes included composite maternal (e.g., unplanned operative delivery, postpartum hemorrhage, obstetric trauma) and neonatal (e.g., shoulder dystocia, severe birth injuries, need for neonatal resuscitation) complications. Covariates such as maternal age, parity, body mass index, diabetes and socioeconomic factors were used to create propensity scores for the probability of experiencing induction. Overlap weighting of propensity scores was used in modified Poisson regression models.

**Results**: Among 10,884 deliveries complicated by suspected LGA, induction rates increased from 1.5% at 37 weeks, to 26.8% at 41 weeks. The maternal composite outcome occurred in 46.2% and 46.5% of births with expectant management and induction, respectively, and increased with advancing gestation (Fig 1). The neonatal composite was stable across gestational weeks and occurred in 27.6% with expectant management and 26.4% with induction. While crude models suggested lower rates of maternal complications with induction (e.g. rate ratio [RR] at 39 weeks 0.90, 95% CI 0.85‐0.95), adjusted models showed no association with composite maternal or neonatal complications, irrespective of gestational week (e.g. RR at 39 weeks 1.00, 95% CI 0.90–1.10; Fig 2).

**Conclusion**: Induction of labor for suspected LGA among term pregnancies compared to expectant management did not change the risk of composite maternal or neonatal complications at any gestational week.

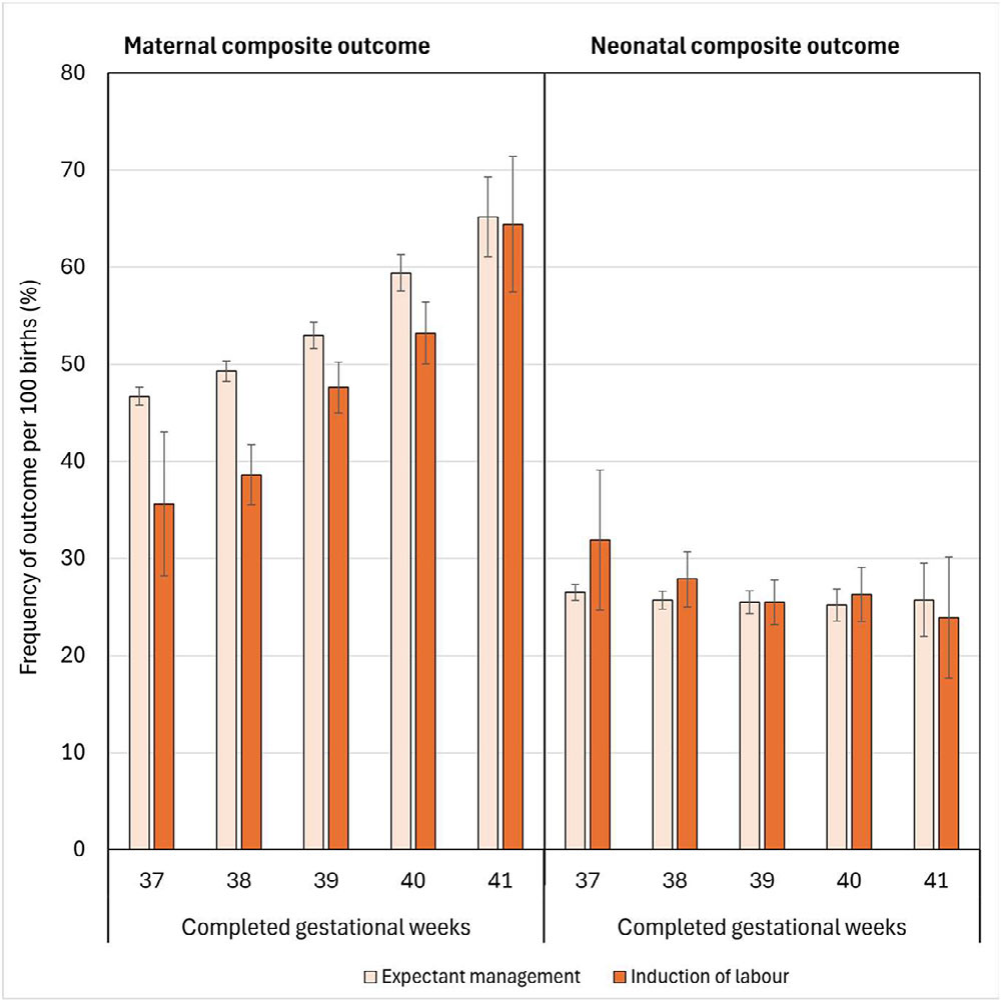

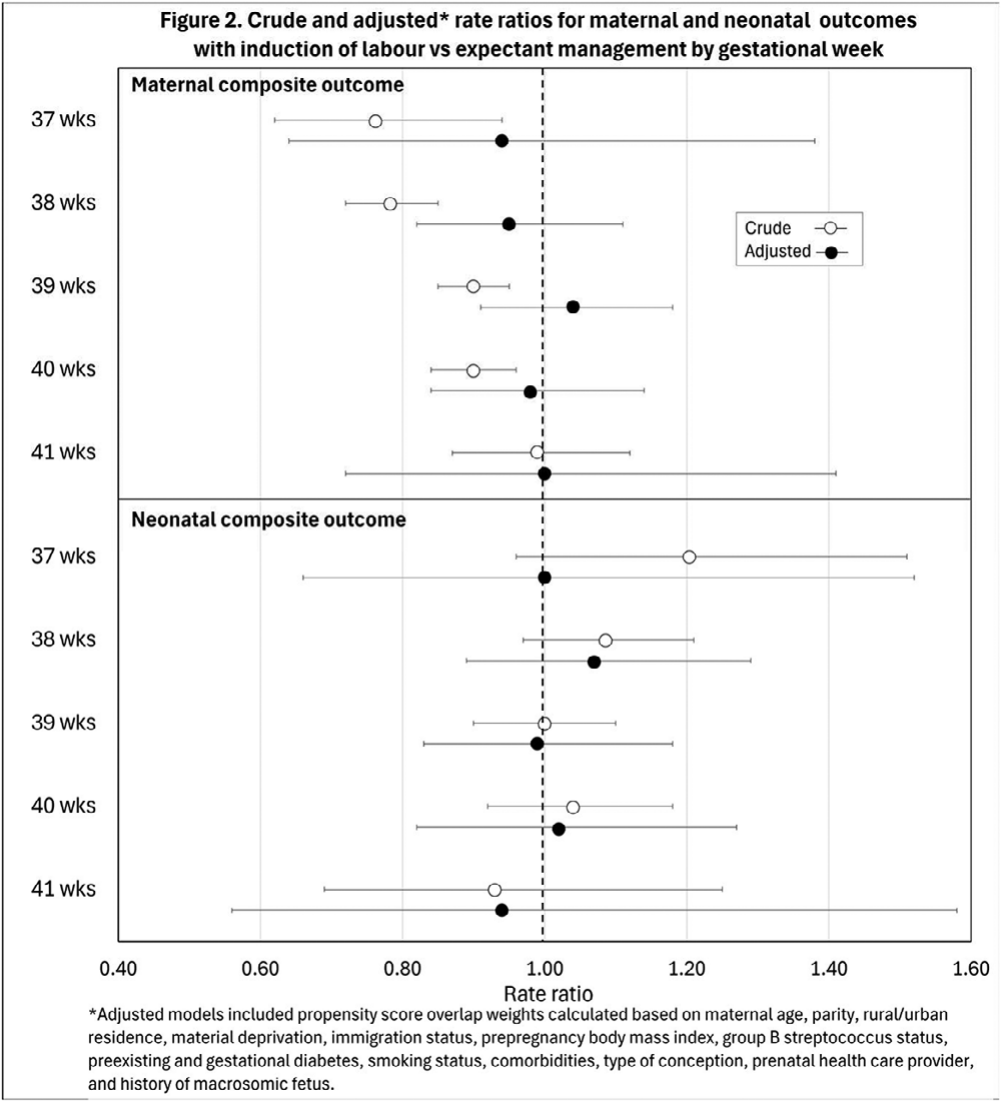

**10:30 AM – 12:00 PM**

## 344 | Long‐Term Endocrine Outcomes Following Antenatal Corticosteroid Exposure in Preterm Infants

Sapir Ellouk^1^; Gali Pariente^2^; Tamar Wainstock^3^; Eyal Sheiner^4^

^1^Department of Obstetrics and Gynecology, Soroka University Medical Center, Ben‐Gurion University, HaDarom; ^2^Department of Obstetrics and Gynecology, Soroka University Medical Center, Ben‐Gurion University, beer sheva, HaDarom; ^3^Department of Epidemiology, Biostatistics and Community Health Sciences, Faculty of Health Sciences, Ben‐Gurion University of the Negev, Beer Sheva, HaDarom; ^4^Soroka University Medical Center, Faculty of Health Sciences, Ben‐Gurion University of the Negev, beer sheva, HaDarom

**Objective**: Antenatal corticosteroids (ACS) are routinely administered to reduce complications associated with preterm birth; however, their long‐term effects on the endocrine system in offspring remain under investigation, particularly concerns regarding neonatal hypoglycemia. We investigated the long‐term endocrine morbidity of preterm offspring exposed to ACS prior to 34 weeks of gestation.

**Study Design**: We conducted a population‐based cohort study of singleton preterm deliveries between 1991 and 2021. Long‐term endocrine morbidity was compared between offspring exposed to ACS before 34 weeks of gestation and unexposed counterparts, using linked community and hospitalization databases. Cumulative incidence was assessed with Kaplan–Meier survival analysis, and a Cox proportional hazards model was used to adjust for potential confounders.

**Results**: The study included 13,580 preterm deliveries, of which 1538 (11.3%) offspring were exposed to ACS. Both the overall incidence of endocrine morbidity per 1000 person‐years (Table) and the cumulative incidence over time (log‐rank *p* < 0.001; Figure) were significantly higher among ACS‐exposed children compared to unexposed children. In addition, ACS‐exposed offspring had significantly higher rates of hypoglycemia and adrenal disorders (*p* < 0.001 for both; Table). In a Cox regression model adjusted for maternal age, gestational age, diabetes mellitus, hypertensive disorders, and mode of delivery, ACS exposure before 34 weeks was independently associated with an increased risk of long‐term endocrine morbidity (aHR 1.35; 95% CI, 1.18–1.54; *p* < 0.001).

**Conclusion**: ACS exposure before 34 weeks of gestation may be associated with an increased risk of long‐term endocrine morbidity in preterm offspring. However, the possibility of indication bias cannot be excluded. Further studies are warranted to confirm these findings and clarify the underlying mechanisms.

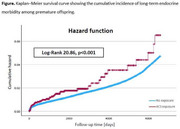

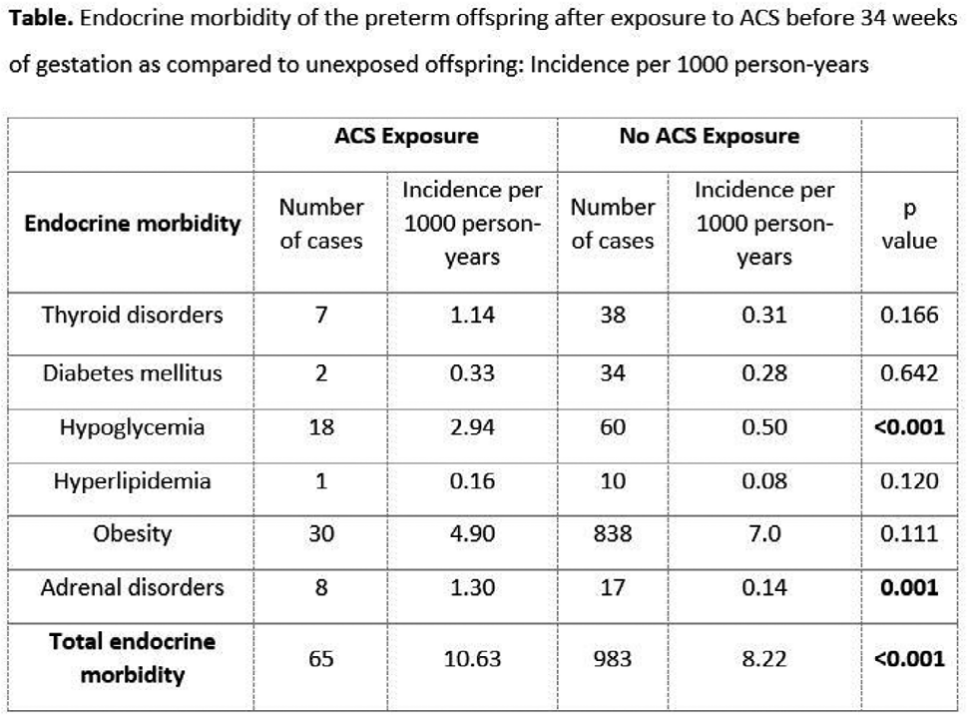

**10:30 AM – 12:00 PM**

## 345 | Parity as a Predictor of Future Endocrine Morbidity in Women

Sapir Ellouk^1^; Gil Gutvirtz^2^; Tamar Wainstock^3^; Eyal Sheiner^4^; Ruslan Sergienko^5^; Roy Kessous^6^

^1^Department of Obstetrics and Gynecology, Soroka University Medical Center, Ben‐Gurion University, HaDarom; ^2^Department of Obstetrics and Gynecology, Soroka University Medical Center, Ben‐Gurion University, Metar, HaDarom; ^3^Department of Epidemiology, Biostatistics and Community Health Sciences, Faculty of Health Sciences, Ben‐Gurion University of the Negev, Beer Sheva, HaDarom; ^4^Soroka University Medical Center, Faculty of Health Sciences, Ben‐Gurion University of the Negev, beer sheva, HaDarom; ^5^Ben‐Gurion University of the Negev, ben gurion, HaDarom; ^6^Soroka, BEER SHEVA, HaDarom

**Objective**: Reproductive years represent a critical period in women's lives, during which the body undergoes substantial physiological and hormonal changes that may provide insight into future health status. Concerns have been raised regarding the potential association between parity and the development of metabolic syndrome and insulin resistance in women. This study aimed to investigate the association between parity and subsequent maternal endocrine morbidity.

**Study Design**: We conducted a population‐based study of women who delivered between 1991–2021 at a tertiary medical center, with varying parity. We included women who had completed their reproductive period, allowing for definitive assessment of parity, and who had no known prior endocrine morbidity. Long‐term maternal endocrine morbidity was diagnosed in outpatient visits or hospitalization records and was compared between parity groups. Cumulative incidence was evaluated using Kaplan‐Meier survival curves, and a Cox proportional hazards model was used to adjust for potential confounders.

**Results**: The study included 53,794 women who had concluded childbearing. Increasing parity was associated with an increased risk of overall endocrine morbidity, including thyroid disorders, diabetes mellitus, hyperlipidemia, and obesity (Table). Kaplan‐Meier survival analysis revealed a significantly higher cumulative incidence of overall endocrine morbidity over time with increasing parity (Figure). In a Cox proportional hazards model adjusted for ethnicity and use of fertility treatments, parity remained independently associated with an increased risk of future maternal endocrine morbidity, demonstrating a dose‐response relationship (Table).

**Conclusion**: Higher parity is independently associated with an increased risk of maternal endocrine morbidity later in life, demonstrating a clear dose‐response relationship. These findings suggest that parity may serve as a marker for future endocrine health and highlight the importance of long‐term monitoring and preventive strategies for multiparous women.

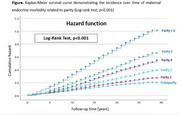

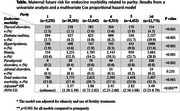

**10:30 AM – 12:00 PM**

## 346 | Neonatal Respiratory Outcomes by Delivery Indication Following Antenatal Corticosteroid Administration

Sara E. Edwards^1^; Nicola F. Tavella^2^; Grace M. DiGiovanni^3^; Sunidhi Singh^3^; Camila Johanek^3^; Sarriyah Hanif^3^; Gabriele Baptiste^4^; Dayana Mirzaliev^1^; Monica J. Patel^1^; Alexandra N. Mills^3^; Daniel Katz^3^; Chelsea A. DeBolt^1^; Angela T. Bianco^3^

^1^Division of Maternal‐Fetal Medicine, Icahn School of Medicine at Mount Sinai, New York, NY; ^2^Division of Maternal‐Fetal Medicine, Icahn School of Medicine, Icahn School of Medicine at Mount Sinai Hospital, New York, NY; ^3^Icahn School of Medicine at Mount Sinai, New York, NY; ^4^SUNY Downstate Health Sciences University, Brooklyn, NY

**Objective**: Prior studies show improved preterm neonatal respiratory outcomes with administration of antenatal corticosteroids (ACS); other studies show improved respiratory outcomes following spontaneous vs. iatrogenic preterm birth. Our study assessed neonatal respiratory outcomes with iatrogenic vs. spontaneous birth after ACS administration. We hypothesized that neonates born via iatrogenic birth would experience worse respiratory outcomes despite ACS administration.

**Study Design**: This was a retrospective cohort study of all patients delivering at a single academic institution in New York City from January 1, 2013 through December 31, 2023 who received 2 doses of betamethasone prior to delivery and delivered at <37 weeks. Patients were excluded if they received >2 doses, did not deliver at our institution, received betamethasone at >37 weeks, or experienced an intrauterine fetal demise. We assessed electronic medical records for clinical and demographic data. Our primary outcome was a neonatal adverse respiratory composite (transient tachypnea of the newborn, respiratory distress syndrome (RDS), use of CPAP for >12 hours, oxygen for >24 hours, intubation, and/or surfactant). Multivariable logistic regression models controlling for potential confounders examined association of iatrogenic vs. spontaneous birth with neonatal respiratory outcomes.

**Results**: 1661 patients met inclusion criteria for analysis. 631 neonates were born spontaneously. Those born spontaneously delivered at later gestational ages, with lower rates of hypertension and fetal growth restriction, and higher rates of vaginal delivery, preterm premature rupture of membranes, and chorioamnionitis (Table 1). After controlling for potential confounders, neonates born via iatrogenic delivery experienced significantly higher rates of the adverse respiratory composite, RDS, and need for CPAP for >12 hours, oxygen for >24 hours, and surfactant (Table 2).

**Conclusion**: Neonates born via iatrogenic delivery experienced worse respiratory outcomes than those delivering spontaneously, despite administration of ACS.

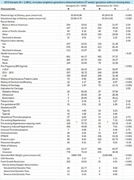

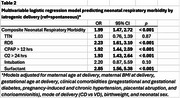

**10:30 AM – 12:00 PM**

## 347 | The Increasing Contribution of Comorbidities to Disparities in Severe Maternal Mortality in the United States

Sara I. Jones^1^; Emma Peek^2^; Lillian Boettcher^3^; Maya Nitecki^4^; Hannah Kelly^1^; Dana Hazimeh^4^; Alexandra Sundermann^5^; Jerome J. Federspiel^1^

^1^Duke University School of Medicine, Durham, NC; ^2^Duke University School of Medicine, Duke University Medical Center, NC; ^3^Department of Obstetrics and Gynecology, Duke University School of Medicine, Duke University School of Medicine/Durham, NC; ^4^Duke University Medical Center, Duke University Medical Center, NC; ^5^Vanderbilt University Medical Center, Vanderbilt University Medical Center, TN

**Objective**: Recently, the reported incidence of severe maternal morbidity (SMM) has increased in the United States, and disparities in SMM rates between Black and White birthing people persist. Given that efforts to improve quality of delivery care would be expected to ameliorate these trends, we hypothesized that worsening disparities in the burden of comorbid conditions have led to increasing and more disparate rates of SMM.

**Study Design**: We performed a retrospective cohort study of deliveries to Black and White birthing people between 2013 and 2022 using the National Inpatient Sample. The primary outcome was non‐transfusion SMM. The maternal comorbidity index (MCI) score was generated using methods for comorbidity identification and weighting from previously published criteria. We performed a mediation analysis to evaluate the effect of the MCI on the disparity between race and non‐transfusion SMM, allowing the effect of MCI on SMM to differ by race and calendar year.

**Results**: There were 4,738,497 delivery hospitalizations during the study period. SMM rates increased in the years 2020–2022 compared to prior years (Figure 1A). Compared to White patients, Black patients experienced higher rates of non‐transfusion SMM throughout the study period. MCI scores increased for both Black and White patients throughout the study period, with an enlarging disparity in mean scores (Figure 1B). For Black patients, the proportion of the racial disparity in non‐transfusion SMM incidence attributable to the MCI increased from 30.6% in 2013 to 35.7% in 2022 (Figure 2).

**Conclusion**: The MCI explains a substantial and growing disparity between Black and White birthing people in non‐transfusion SMM rates in the United States, a trend likely exacerbated by the COVID‐19 pandemic. Eradication of racial disparities in obstetric outcomes must continue to include efforts to not only eliminate racism in the intrapartum phase of care, but also to ameliorate the chronic impacts of racism on health preceding delivery.

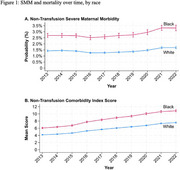

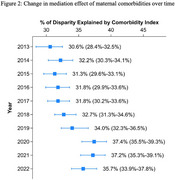

**10:30 AM – 12:00 PM**

## 348 | Association Between Mode of Delivery and Maternal and Neonatal Outcomes in Periviable Births With Malpresentation

Sara E. Post; Amanda A. Allshouse; Ann M. Bruno

University of Utah, Salt Lake City, UT

**Objective**: Active treatment of periviable neonates has increased in the past decade but data informing optimal mode of delivery are lacking. We aimed to evaluate the association between mode of delivery and maternal and neonatal outcomes in periviable births with malpresentation where resuscitation was intended.

**Study Design**: This was a serial cross‐sectional analysis of the National Vital Statistics System from 2016 to 2023. Singleton deliveries between 22w0d to 25w6d with malpresentation and intent for neonatal intervention (defined as one or more: surfactant therapy, immediate assisted ventilation, assisted ventilation >6 hours, neonatal intensive care unit [ICU] admission, antibiotic therapy) were included. Those with a known fetal anomaly or neonatal weight <400 grams were excluded. The exposure was mode of delivery. The primary outcome was a composite maternal morbidity including blood transfusion, ICU admission and unplanned hysterectomy. Secondary outcomes were a composite neonatal morbidity defined as neonatal mortality or seizures. Multivariable modeling estimated the association between mode of delivery and outcomes with odds ratios and 95% confidence intervals reported.

**Results**: Of 15,052 deliveries included, 2301 (15.3%) were vaginal and 12,751 (84.7%) were cesarean. Baseline characteristics of those undergoing cesarean compared with vaginal delivery differed (Table 1). Cesarean delivery was associated with higher odds of maternal morbidity (4.5% vs 2.0%; OR 2.37, 95% CI 1.75–3.22; aOR 2.18, 95% CI 1.60–2.97; Table 2) with all components of the composite occurring more frequently in those undergoing cesarean delivery. Cesarean compared with vaginal delivery was associated with lower odds of neonatal morbidity (11.4% vs 28.4%; OR 0.33, 95% CI 0.29‐0.36, aOR 0.42, 95% CI 0.38‐0.47).

**Conclusion**: In periviable births with malpresentation and intention to resuscitate, cesarean delivery was associated with increased maternal morbidity but decreased neonatal morbidity. Findings may inform patient counseling considering risks and benefits of both mother and perinate.

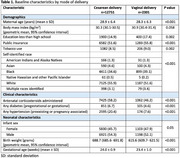

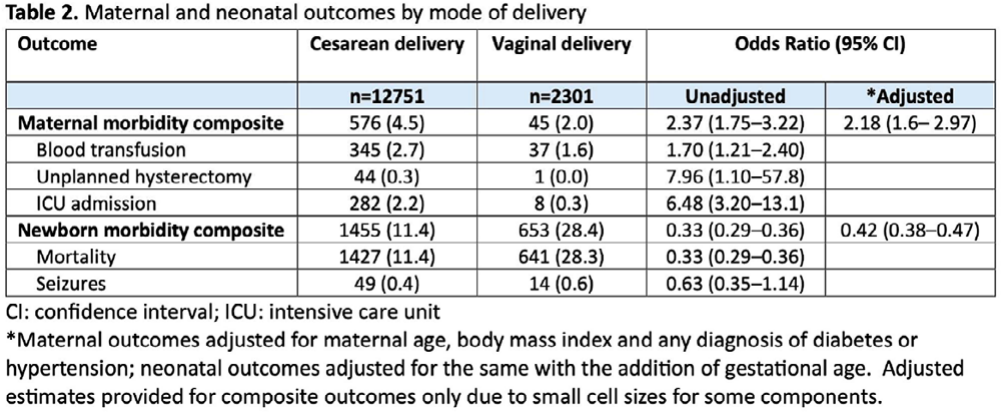

**10:30 AM – 12:00 PM**

## 349 | Impact of Inadequate Gestational Weight Gain on Pregnancy Outcomes in Patients With Inflammatory Bowel Disease

Sarah H. Abelman; Sarah Z. Wu; Isabella A. Molina; Frank I. Jackson; Oladunni Ogundipe; Jolene C. Muscat; Luis A. Bracero; Matthew J. Blitz

Northwell, New Hyde Park, NY

**Objective**: Patients with inflammatory bowel disease (IBD) are at an increased risk of inadequate gestational weight gain (GWG) compared to the general obstetric population. This study evaluated how inadequate GWG influenced pregnancy outcomes in individuals with IBD.

**Study Design**: This retrospective cohort study included all pregnant patients with IBD who had live births ≥23 weeks’ gestation between January 2019 and December 2024 across a large New York health system. GWG adequacy was classified using Institute of Medicine guidelines based on pre‐pregnancy BMI. Outcomes included preterm birth (<37 weeks), small for gestational age (SGA, <10th percentile) and neonatal intensive care unit (NICU) admission. Multivariable logistic regression was used to estimate associations between inadequate GWG and each outcome, adjusting for BMI class, type of IBD (Crohn's disease vs. Ulcerative Colitis), systemic IBD therapy, bowel resection, obstetric comorbidity index (OB‐CMI), prior preterm birth, multiparity, insurance status, and race/ethnicity. Analyses were conducted using R 4.3.1.

**Results**: A total of 319 pregnancies complicated by IBD were included, with 146 (45.8%) involving Ulcerative Colitis and 173 (54.2%) Crohn's disease. 37 (11.6%) had a history of bowel resection, and 215 (67.4%) received systemic therapy for IBD. Inadequate GWG occurred in 71 patients (22.3%). In both unadjusted and adjusted models (Table 1), inadequate GWG was not associated with significantly increased odds of preterm birth (aOR 2.37, 95% CI 0.89–6.36), SGA (aOR 1.68, 95% CI 0.56–4.88), or NICU admission (aOR 1.27, 95% CI 0.59–2.69).

**Conclusion**: While patients with IBD are at increased baseline risk for adverse pregnancy outcomes, inadequate gestational weight gain did not confer a significantly higher risk of preterm birth, SGA, or NICU admission in this cohort. These findings suggest that suboptimal weight gain may not independently worsen perinatal outcomes among patients with IBD, though continued individualized monitoring remains essential.

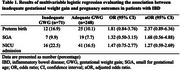

**10:30 AM – 12:00 PM**

## 350 | Impact of TNF Alpha Inhibitors on Hypertensive Disorders in Pregnancies With Autoimmune Disease

Sarah H. Abelman; Isabella A. Molina; Sarah Z. Wu; Oladunni Ogundipe; Frank I. Jackson; Luis A. Bracero; Matthew J. Blitz

Northwell, New Hyde Park, NY

**Objective**: TNF alpha is hypothesized to play a role in the pathophysiology of hypertensive disorders of pregnancy (HDP). Many patients with autoimmune disorders are treated with anti‐TNF alpha biologic therapies. This study aimed to assess whether the use of these agents impacts the rates of HDP.

**Study Design**: This was a retrospective cohort study of all pregnant patients with autoimmune diseases eligible for anti‐TNF alpha therapy with live births ≥23 weeks’ gestation, from 2019–2024 within a large health system in New York. The primary exposure was anti‐TNF alpha agent use. The primary outcome was HDP and secondary outcome was preeclampsia with severe features. Multivariable logistic regression was used, adjusting for body mass index (BMI) class, advanced maternal age, chronic hypertension, pregestational diabetes, race/ethnicity group, and low‐dose aspirin use. A subgroup analysis was performed for inflammatory bowel disease (IBD) specifically. Analyses were performed in R 4.3.1.

**Results**: 543 pregnancies complicated by eligible autoimmune diseases were included, the most common being IBD (58.7%) and rheumatoid arthritis (30.9%). Of these, 119 (21.9%) were on TNF alpha inhibitors (34.5% on adalimumab, 32.8% on infliximab, and 30.3% on certolizumab). 83 pregnancies (15.1%) were complicated by an HDP diagnosis and 49 (9.0%) by preeclampsia with severe features. In adjusted and unadjusted analyses, those on TNF alpha inhibtors had no increased risk of HDP (aOR 1.16, 95% CI 0.60–2.16) or severe preeclampsia. In the subgroup of 319 with IBD (45.8% with ulcerative colitis and 54.2% with Crohn's disease), 77 (24.1%) were on TNF alpha inhibitors. 40 (12.5%) were complicated by HDP and 26 (8.2%) by severe preeclampsia. In adjusted and unadjusted analyses, those on anti‐TNF alpha medications had no increased risk of HDP (aOR 0.90, 95% CI 0.36–2.07) or severe preeclampsia.

**Conclusion**: Although elevated levels of TNF‐alpha have been shown to be associated with preeclampsia, the use of TNF‐alpha inhibitors for the treatment of autoimmune diseases including IBD does not appear to reduce the risk of HDP.

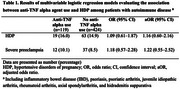

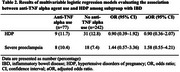

**10:30 AM – 12:00 PM**

## 351 | Microbial Signatures of Cardiometabolic Risk and Hypertensive Disorder of Pregnancy: A Prospective Cohort Study

Sarah C. Graves^1^; Uma Perni^2^; Stanley Hazen^3^; Adina R. Kern‐Goldberger^4^; Jodi Meridieth^2^; Megan R. Ansbro^5^; Xinmin Li^2^; Deepthi Mallela^6^; Madeline Joquico^2^; Cara D. Dolin^7^

^1^VCU School of Medicine, VCU Health, Richmond, VA; ^2^Cleveland Clinic, Cleveland, OH; ^3^Cleveland Clinic, Cleveland, OH; ^4^Cleveland Clinic Foundation, Cleveland Clinic Foundation, OH; ^5^Cleveland Clinic Foundation, Cleveland Clinic Foundation, Cleveland, OH; ^6^Cleveland Clinic Lerner Research Institute, Cleveland Clinic, OH; ^7^Cleveland Clinic, Clevelend, OH

**Objective**: To evaluate the association between maternal thrombosis and atherosclerosis‐related gut microbial metabolites and hypertensive disorder or pregnancy.

**Study Design**: Prospective cohort study enrolling patients ≥ 18 years with singleton pregnancy between 10–14 weeks’ gestation. Maternal blood and urine were collected at 10–14 weeks (visit 1), 24–28 weeks (visit 2), and at time of delivery (visit 3). Clinical variables including demographics, medical/obstetric history, pregnancy outcomes, and cardiovascular parameters including blood pressure and baseline labs were collected. Assays for predetermined gut microbiota‐derived metabolites, trimethylamine‐N‐oxide (TMAO), phenylacetylglutamine (PAG), indole acetic acid (IAA), and indole‐3‐propionic acid (I3PA), were performed using isotope dilution LC‐MS/MS methods. Metabolite levels were compared between patients who developed HDP and those who did not and summarized across gestation. Measure of association were assessed using multivariable logistic regression and reported as OR and adjusted OR, adjusting for potential confounders.

**Results**: Baseline characteristics were similar between groups. IAA was lower in the second trimester in those with HDP compared to no HDP [1.14 (1.01‐ 1.38) vs 1.31 (1.04‐ 1.64), *p* = 0.02]. Time of delivery I3PA was significantly lower in those with HDP vs no HDP (A non‐significant trend of lower I3PA and IAA were seen in Visit 1. Median TMAO and PAG trended higher in those with HDP though did not reach statistical significance.

**Conclusion**: Lower mid‐gestation IAA and delivery I3PA levels were associated with hypertensive disorders of pregnancy, particularly preeclampsia with severe features. These findings suggest that gut microbial indole derivatives may reflect subclinical cardiometabolic dysfunction preceding HDP onset. IAA and I3PA merit further evaluation as candidate biomarkers or therapeutic targets to improve risk stratification and understanding of HDP pathogenesis.

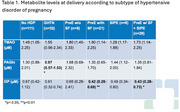

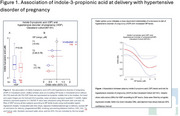

**10:30 AM – 12:00 PM**

## 352 | Maternal Microbial Metabolite Profiles and Risk of Adverse Pregnancy Outcomes

Sarah C. Graves^1^; Adina R. Kern‐Goldberger^2^; Stanley Hazen^3^; Easha Patel^4^; Megan R. Ansbro^5^; Xinmin Li^6^; Deepthi Mallela^7^; Cara D. Dolin^8^

^1^VCU School of Medicine, VCU Health, Richmond, VA; ^2^Cleveland Clinic Foundation, Cleveland Clinic Foundation, OH; ^3^Cleveland Clinic, Cleveland, OH; ^4^Cleveland Clinic Foundation, Cleveland, OH; ^5^Cleveland Clinic Foundation, Cleveland Clinic Foundation, Cleveland, OH; ^6^Cleveland Clinic, Cleveland, OH; ^7^Cleveland Clinic Lerner Research Institute, Cleveland Clinic, OH; ^8^Cleveland Clinic, Clevelend, OH

**Objective**: To evaluate the association between cardiometabolic‐related gut microbial metabolites and the pregnancy‐specific cardiovascular risk factors, small for gestational age (SGA), preterm birth (PTB), and gestational diabetes mellitus (GDM).

**Study Design**: Prospective cohort study enrolling patients ≥ 18 years with singleton pregnancy between 10–14 weeks’ gestation. Maternal blood and urine were collected at 10–14 weeks (visit 1), 24–28 weeks (visit 2), and at time of delivery (visit 3). Clinical variables including demographics, medical/obstetric history, pregnancy outcomes, and cardiovascular parameters including blood pressure and baseline labs were collected. Assays for gut microbiota‐derived metabolites, trimethylamine‐N‐oxide (TMAO), phenylacetylglutamine (PAG), indole acetic acid (IAA), and indole‐3‐propionic acid (I3PA), were performed using isotope dilution LC‐MS/MS methods. Metabolite levels were compared between individuals who developed SGA, PTB, and GDM, and those who did not across gestation, and measures of associations were assessed using multivariable logistic regression and reported as crude and adjusted OR(95% CI) after adjusting for potential confounders.

**Results**: Visit 1 I3PA levels were significant associated with development of GDM, unadjusted and adjusted OR with 95% CI: 0.19 (0.05,0.71) and 0.17 (0.05,0.66). Visit 3 I3PA showed lower levels associated with GDM but this was no longer significant after adjusting for potential confounders. There was no association with TMAO, PAG, IAA, or I3PA with SGA or PTB.

**Conclusion**: Reduced first‐trimester levels of I3PA were independently associated with GDM, suggesting early disruption in gut microbe–host metabolic signaling may contribute to dysglycemia. This is consistent with prior evidence linking higher I3PA levels to enhanced insulin sensitivity and a reduced risk of glucose intolerance. No significant associations were observed between gut microbial metabolites and SGA or PTB.

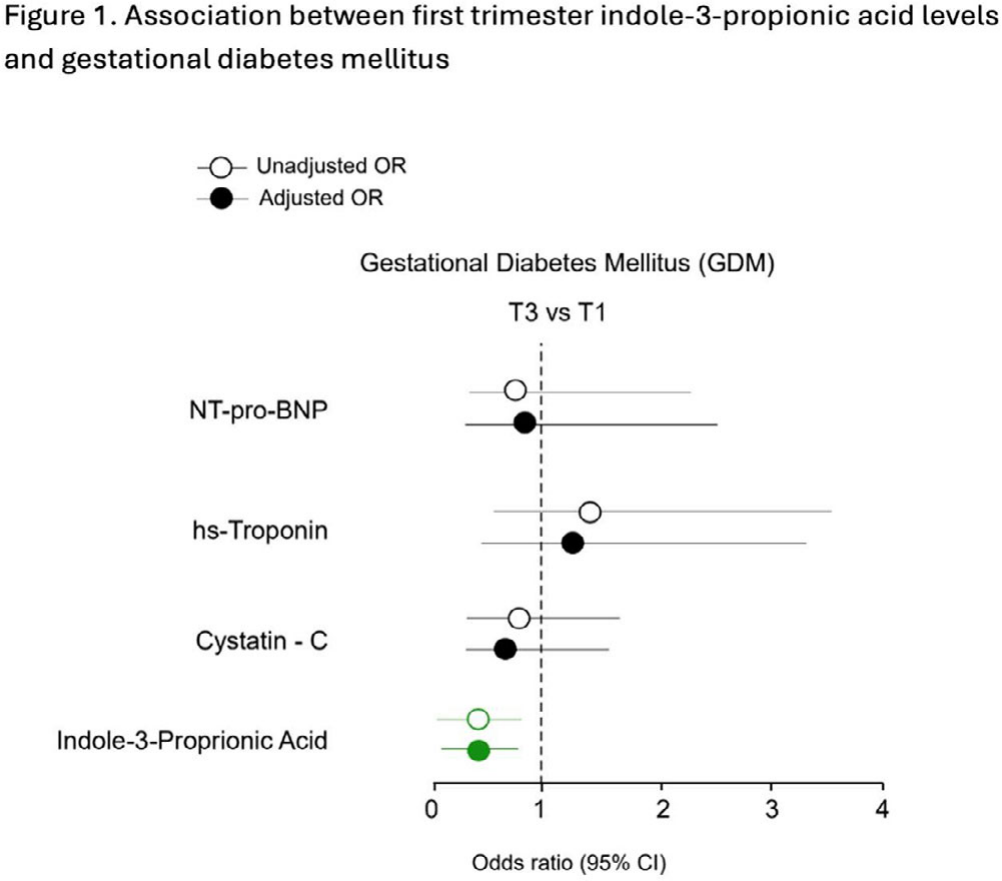

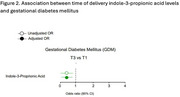

**10:30 AM – 12:00 PM**

## 353 | An Increased Multidimensional Early Pregnancy Allostatic Load is Associated With Placentally‐Mediated Adverse Outcomes

Sarah Heerboth^1^; Maura Jones^2^; Emily Gascoigne^2^; Mandy Bush^2^; Annie Dude^2^; Carmen Beamon^3^; Rebecca Fry^2^; Tracy A. Manuck^2^

^1^University of North Carolina ‐ Chapel Hill, UNC Chapel Hill, Chapel Hill, NC; ^2^University of North Carolina, Chapel Hill, NC; ^3^WakeMed, Raleigh, NC

**Objective**: Allostatic load (AL) is a multidimensional construct encompassing cumulative physiologic, psychological, and environmental stressors. An increased AL is associated with HTN and cardiovascular disease outside of pregnancy. However, it is unclear if AL is also associated with elevated CV complications in pregnancy.

**Study Design**: Secondary analysis of a prospective cohort enriched for those at high *a priori* risk of PTB. Participants identifying as Black, White, and/or Hispanic, with singleton, non‐anomalous gestations were recruited <22 weeks, 2017–2022. At enrollment, baseline demographic and health information were collected and participants provided a blood sample via standard venipuncture. We considered 6 markers previously considered in other AL indices: CV domain factors (initial prenatal systolic BP, initial prenatal diastolic BP), a metabolic factor (pre‐pregnancy BMI), and inflammation (plasma IL‐6, IL‐10, and IL‐1β levels). Each marker was classified as ‘high’ (≥75^th^ centile; 1 point) or ‘low’ (<75^th^ centile; 0 points). The total AL score was calculated by summing these 6 factors for a max AL score = 6. The primary outcome was a diagnosis of plac‐comps (hypertensive disorders of pregnancy, birthweight <10% for gestational age and sex, ± placental abruption).

**Results**: 493 participants were included; 113 (23%) had plac‐comps. Baseline / pregnancy characteristics are shown in the Table. The ≥ 75^th^ centile used to classify each AL marker as ‘high’ and add +1 point to an individual's AL score were: (1) SBP ≥125mmHg; (2) DBP ≥ 77 mmHg; (3) BMI ≥35.4 kg/m^2^; (4) IL‐6 ≥11; (5)IL‐10 ≥ 12; (6) IL‐1β ≥13. The median AL score was 1 (IQR 0, 2); rates of plac‐comps increased with higher AL scores (Figure 1a). In logistic regressions, an AL score of 0 was associated with a lower odds of plac‐comps whereas AL scores ≥1 were associated with an increased adjusted odds (Figure 1b).

**Conclusion**: A multidimensional AL score incorporating CV, metabolic health, and inflammatory biomarkers from early pregnancy is associated with the development of placentally‐mediated adverse outcomes in a dose‐dependent manner.

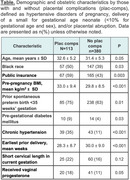

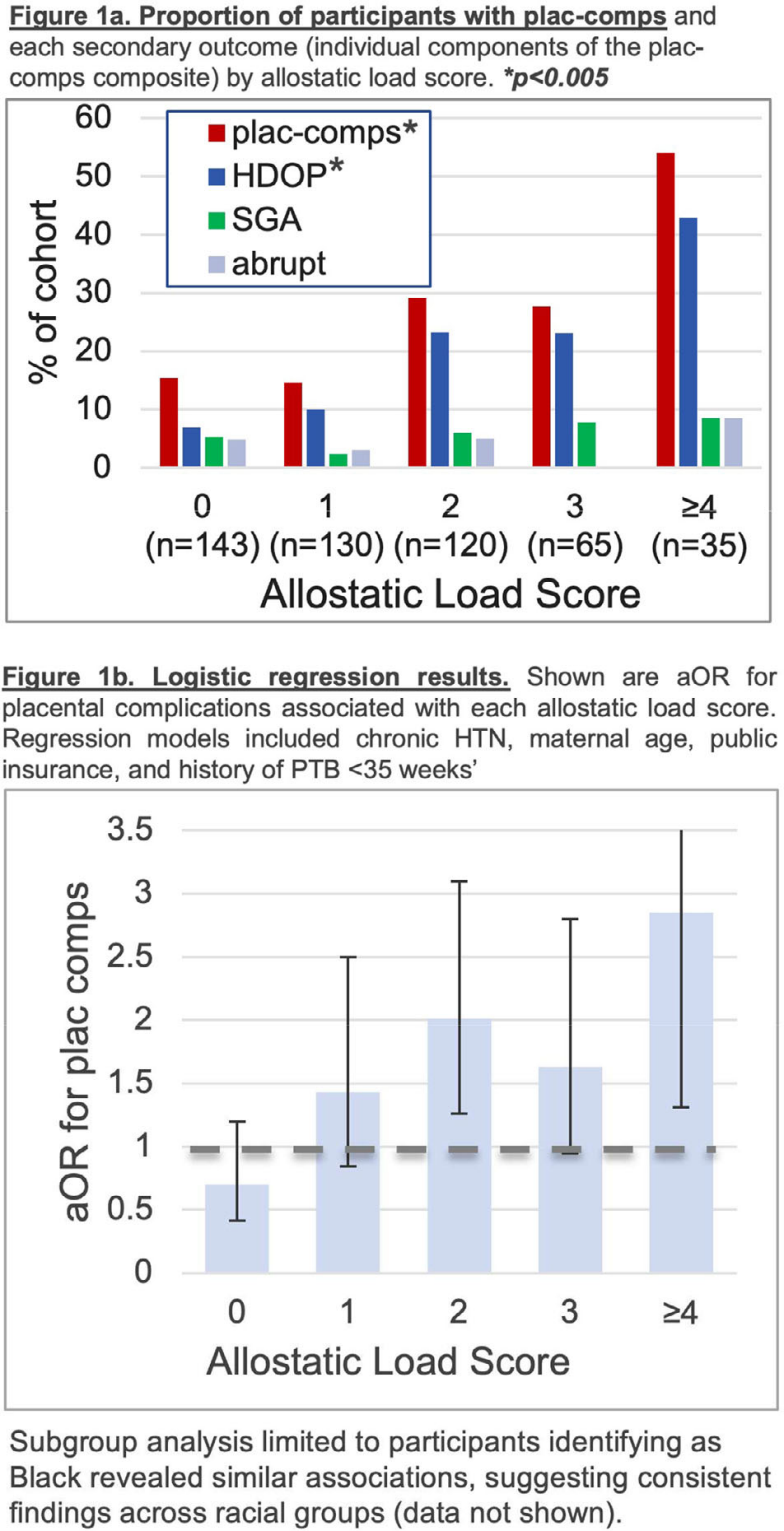

**10:30 AM – 12:00 PM**

## 354 | Outcomes of Congenital Heart Disease Deliveries at a Freestanding Children's Hospital

Sarah J. Kopp^1^; Mounira A. Habli^1^; Taylor M. Trussell^1^; Allison Divanovic^1^; Jeffrey Alten^1^; James F. Cnota^2^

^1^Cincinnati Children's Hospital Medical Center, Cincinnati, OH; ^2^Cincinnati Children's Hospital Medical Center, Cincinnati Children's, OH

**Objective**: Special delivery units (SDU) at freestanding pediatric hospitals enable infants with life‐threatening conditions, such as critical congenital heart defects (CCHD), to be born where they will receive specialty postnatal care. Outcomes data are limited. In pregnancies complicated by CCHD, we compared maternal/infant outcomes of deliveries at our institution's SDU and local level III delivery units. We also assessed how social drivers of health (SDoH) influenced SDU eligibility, given that medical comorbidities primarily determine delivery location.

**Study Design**: Retrospective cohort study of mother‐infant dyads with prenatal diagnoses of CCHD born between 2019–2025. Infant and maternal variables are detailed in Tables 1 and 2. We evaluated SDoH with geo‐coded deprivation index, insurance type, and maternal ethnicity/race. Descriptive statistics were presented as median (interquartile range) or percentages and frequencies. Continuous variables were compared between delivery location using Wilcoxon Rank‐Sum test. Categorical variables were compared using Chi‐squared test.

**Results**: Among 384 dyads, 91% of the 285 eligible mothers delivered at SDU. Aortic coarctation/hypoplasia was the most common CCHD (*n* = 142, 37%). Outcomes are summarized in Tables 1 and 2. Mothers with higher deprivation index scores were less likely to be eligible for SDU delivery (*p* = 0.032). There was a significant difference between insurance type and SDU eligibility, with higher rates of private insurance at SDU (40% vs 34%). Maternal race/ethnicity was not associated with eligibility.

**Conclusion**: SDU is associated with more frequent vaginal delivery and improved early infant outcomes, though not with later infant hospitalization measures. Breastmilk provision was higher, suggesting that the dyad interaction may enhance SDU value. SDU eligibility, reflecting maternal delivery risk, is associated with some measures of SDoH and may contribute to health disparities.

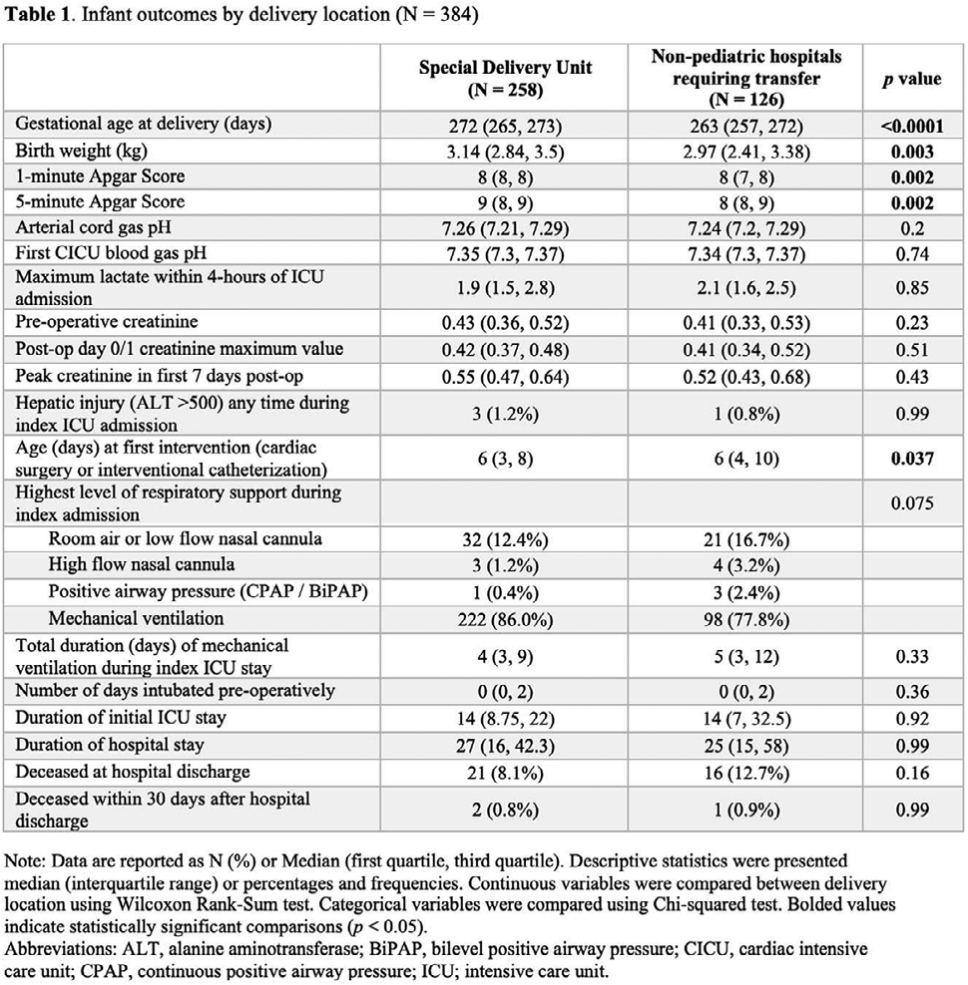

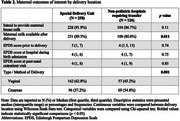

**10:30 AM – 12:00 PM**

## 355 | Cesarean Delivery Surgical Techniques and Association With Placenta Accreta Spectrum Among Pregnancies With Placenta Previa

Sarah T. Mehl^1^; Anthony Chartier^2^; Sandra Sadek^2^; Elias Kassir^1^; Diego Aviles^3^; Edgar A. Hernandez‐Andrade^1^; Megan C. Shepherd^1^; Sean C. Blackwell^1^; Baha M. Sibai^1^; Farah H. Amro^1^

^1^Division of Maternal‐Fetal Medicine, Department of Obstetrics, Gynecology and Reproductive Sciences, McGovern Medical School at UTHealth Houston, Houston, TX; ^2^Department of Obstetrics, Gynecology and Reproductive Sciences, McGovern Medical School at UTHealth Houston, Houston, TX; ^3^Division of Gynecology Oncology, Department of Obstetrics, Gynecology and Reproductive Sciences, McGovern Medical School at UTHealth Houston, Houston, TX

**Objective**: The impact of surgical technique for hysterotomy closure during cesarean delivery (CD) and risk of placenta accreta spectrum (PAS) in subsequent pregnancy has not been well studied. Optimal technique to reduce risk of PAS remains unclear. Our objective was to compare surgical technique from antecedent CD between pregnancies with placenta previa alone and previa with PAS.

**Study Design**: Retrospective cohort study of pregnancies with placenta previa in the current pregnancy with at least one prior CD from 2016–2025 that delivered within our institution's hospital system. Pregnancies were divided into Group 1: no PAS and Group 2: PAS. Maternal characteristics, antecedent CD surgical technique, delivery complications of the antecedent CD, and current pregnancy outcome of PAS severity by pathology were compared.

**Results**: A total of 317 pregnancies with placenta previa were identified with 116 having the antecedent CD operative report available. Maternal characteristics including age, BMI, pre‐gestational diabetes, and short interval pregnancy were similar between groups (Table 1). Pregnancies without PAS were more likely to have 1 prior CD (*p* < 0.02) whereas those with PAS were more likely to have ≥ 3 CD (*p* < 0.04). Antecedent CD complications including postpartum hemorrhage, intra‐amniotic infection, and preterm prelabor rupture of membranes did not differ between groups. Choice of suture and location of uterine incision were similar among both groups. Surgical technique was further evaluated among PAS by severity, with less severe PAS (accreta/increta) having more frequent two‐layer closure in the antecedent CD compared to percreta, though not statistically significant (*p* = 0.16) (Figure 1).

**Conclusion**: In this cohort of pregnancies with placenta previa, rate of PAS was not changed based on antecedent CD surgical technique or delivery complications. A trend was noted between decreased PAS severity and use of two‐layer closure, though not statistically significant. Larger trials are needed to determine the role of single versus double‐layer closure in PAS prevention.

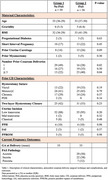

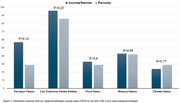

**10:30 AM – 12:00 PM**

## 356 | Risk Factors Associated With Small for Gestational Age: A Secondary Analysis of PRECISE

Sarah Nazeer^1^; Rafael Bravo^2^; Claudia Pedroza^2^; Sean C. Blackwell^2^; Baha M. Sibai^2^; Michal Fishel Bartal^2^

^1^Cleveland Clinic Lerner College of Medicine, Cleveland Clinic Lerner College of Medicine, OH; ^2^Division of Maternal‐Fetal Medicine, Department of Obstetrics, Gynecology and Reproductive Sciences, McGovern Medical School at UTHealth Houston, Houston, TX

**Objective**: Hypoglycemia has been linked to small for gestational age (SGA) infants; however, there is a paucity of data examining CGM metrics and SGA patterns. This study aimed to evaluate CGM metrics and maternal risk factors associated with SGA.

**Study Design**: This was a secondary analysis of the PRECISE trial, in which individuals with singleton pregnancies at 24–30 weeks’ gestation were randomized to either 7‐day CGM or standard 2‐step screening for GDM. A blinded CGM was placed in the CGM group (NCT05430204). This analysis included participants without GDM and compared those with SGA versus appropriate for gestational age (AGA) infants. The primary outcome was a composite of adverse neonatal outcomes including large for gestational age, shoulder dystocia, birth injury, IV/PO glucose need, respiratory distress, and fetal/neonatal death. Secondary maternal and neonatal outcomes were also assessed. CGM metrics included percentage of time in range (TIR; 60–140 mg/dL), time below range (TBR; ≤63 mg/dL), time above range (TAR; >140 mg/dL), mean glucose, and coefficient of variation. Pearson's Chi‐squared or Fisher's exact test was used with *p* < 0.05 as significant.

**Results**: Of the 814 participants, 628 (77%) met inclusion— 74 (12%) were SGA and 554 (88%) were AGA. Aside from lower obesity rates in the SGA group (43% vs 59%, *p* < 0.01), baseline characteristics were similar. No differences were observed in CGM metrics: mean glucose was 103 mg/dL vs 104 mg/dL, and TIR was 92% (±10) vs 91% (±9) in SGA and AGA, respectively (Table 1). Composite neonatal outcomes did not differ (29% vs 24%, *p* = 0.29). SGA infants had higher rates of formula feeding (32% vs 16%, *p* < 0.01) and induction of labor (64% vs 50%, *p* < 0.01), with no difference in gestational age at delivery (both 38.0 weeks) (Table 2).

**Conclusion**: These findings align with prior studies and suggest CGM metrics may not differ in SGA pregnancies. Further research is needed to define glycemic contributions to fetal growth.

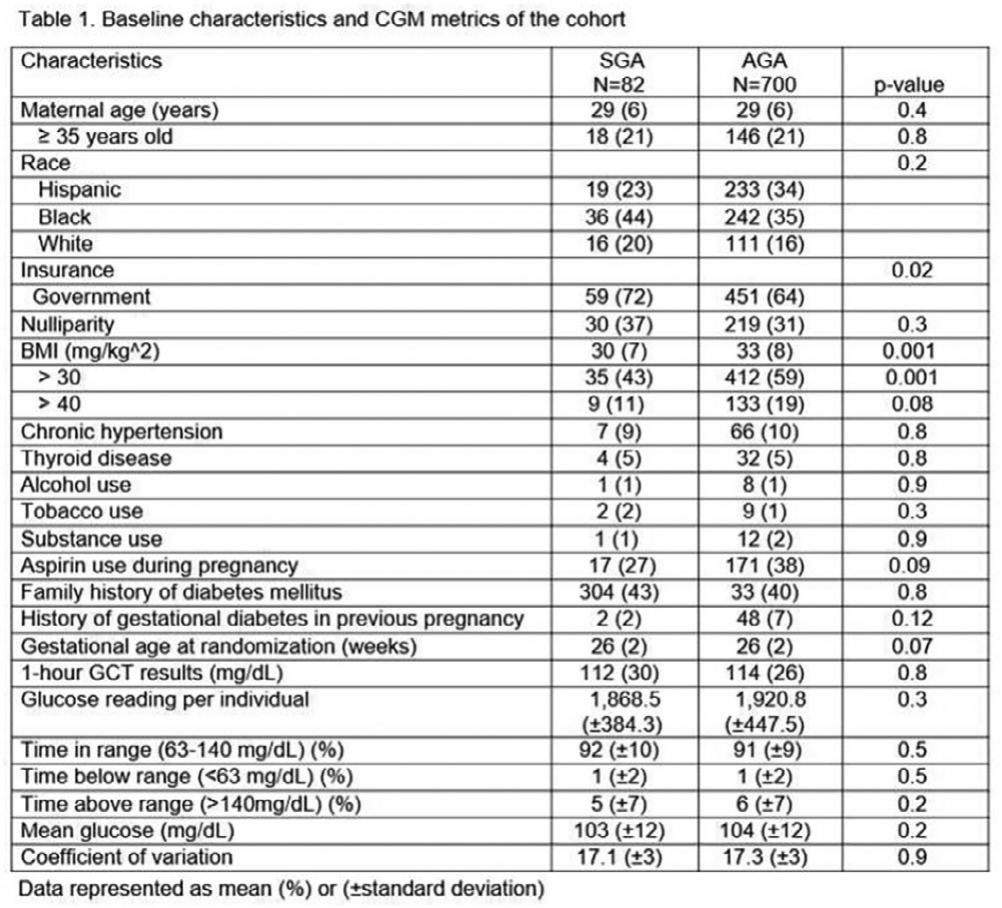

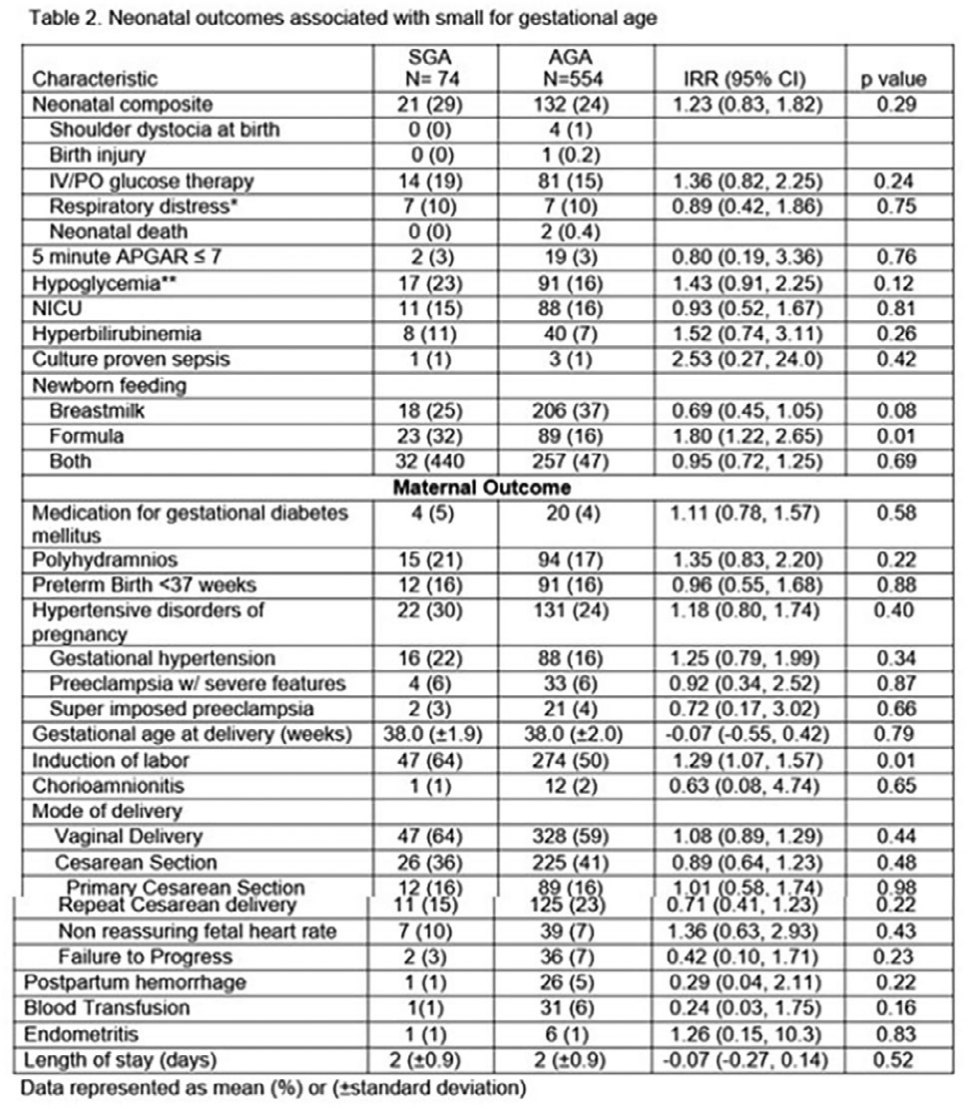

**10:30 AM – 12:00 PM**

## 357 | Correlation of Placenta Accreta Index to Surgical Outcomes for Pregnancies With Suspected Placenta Accreta Spectrum

Sarah Tounsi^1^; Kamran Hessami^1^; Michael D. Jochum, Jr.^1^; Alvin To^2^; Yamely H. Mendez^1^; Christina C. Reed^1^; Amir A. Shamshirsaz^3^; Onur Turkoglu^1^; Hendrik A. Lombaard^1^; Jessian L. Munoz^4^; Michael A. Belfort^1^; Martha Rac^1^; Alexander M. Saucedo^1^

^1^Baylor College of Medicine, Houston, TX; ^2^Brigham and Women's Hospital, Brigham and Women's Hospital, MA; ^3^Division of Maternal‐Fetal Medicine, Department of Obstetrics and Gynecology, Baylor College of Medicine, Houston, TX; ^4^Texas Children's Hospital, Texas Children's Hospital, TX

**Objective**: The Placenta Accreta Index (PAI) is a validated tool combining clinical and sonographic findings to predict placenta accreta spectrum (PAS). While effective for prenatal diagnosis in high‐risk populations, its correlation to surgical outcomes remains unclear. We evaluated whether higher PAI scores are associated with increased surgical morbidity.

**Study Design**: Single center retrospective cohort of patients referred for suspected PAS who were evaluated and delivered at a large academic center between January 2023‐July 2025. Inclusion required ≥ 1 prior cesarean and confirmed low‐lying or previa placentation. Blinded investigators reviewed third‐trimester ultrasounds to calculate PAI scores stratified into three groups: group 1 (PAI ≤3), group 2 (PAI >3 to ≤6), and group 3 (PAI >6). The primary outcome was a composite severe maternal morbidity (SMM) score. Secondary outcomes included FIGO grade, quantitative blood loss (QBL), and use of ureteral stent and aortic occlusion devices. Multivariate analyses and logistic regression were performed.

**Results**: Ninety patients were included. SMM increased significantly across PAI groups (23.5% vs. 55.6% vs. 60.9%; *p* = 0.029). Compared to group 1, the odds of SMM were 4.0x higher in group 2 (CI 1.1–15.7) and 5.1x in group 3 (CI 1.4–17.9). Increasing PAI scores were associated with less frequent low‐transverse hysterotomy (*p* < 0.001), greater incidence of FIGO grade ≥ 3 (*p* < 0.001), higher QBL (*p* = 0.038), longer operative times (*p* < 0.001), and increase perioperative placement of ureteral stents (*p* = 0.007) and aortic occlusion devices (*p* = 0.005 respectively).

**Conclusion**: Increasing PAI scores are associated with increased risk of SMM. Increased scores also demonstrate increased FIGO pathologic grading, blood loss, operative times, and use of ureteral stents and aortic occlusion devices. These findings support the use of PAI not only for prenatal diagnosis of PAS but also as a tool for preoperative planning and management of surgical complexity and morbidity in patients with suspected PAS.

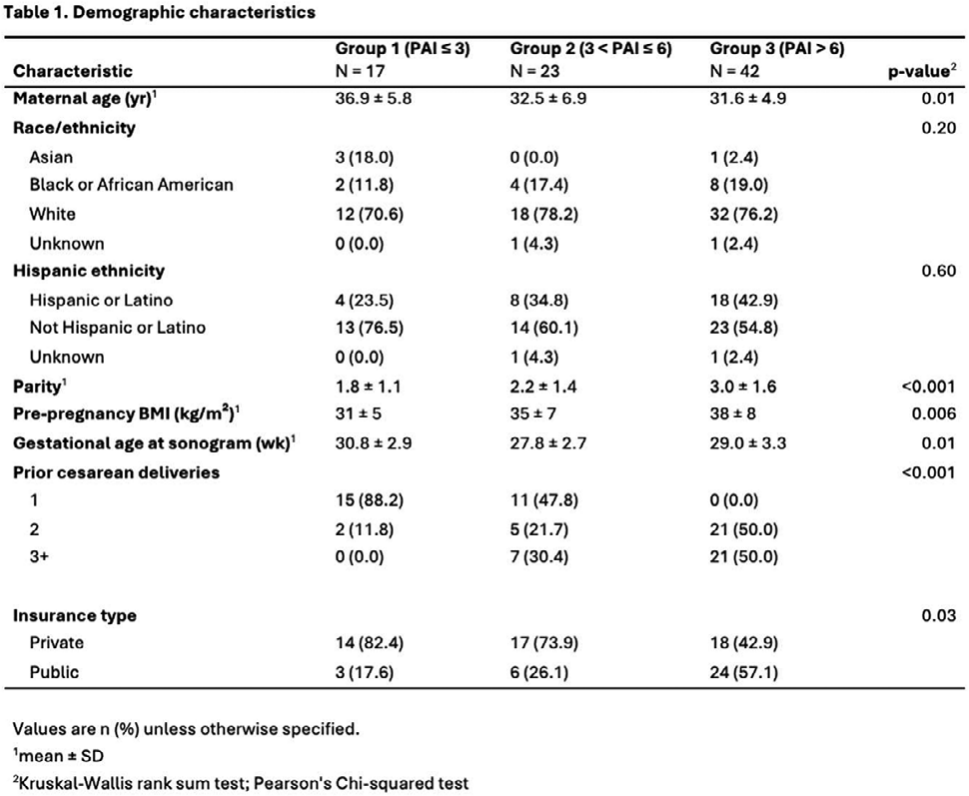

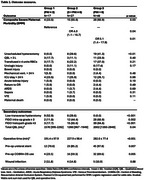

**10:30 AM – 12:00 PM**

## 358 | Feasibility and Outcomes of External Cephalic Version Among SGA Births

Satjeet K. Deol Chauhan^1^; Emily Schlussel Markovic^2^; Rodney A. McLaren, Jr^2^

^1^Bronxcare Health System/Icahn School of Medicine at Mount Sinai, Bronx, NY; ^2^Maimonides Medical Center, Brooklyn, NY

**Objective**: The American College of Obstetricians and Gynecologists recommend that external cephalic version (ECV) should be offered to patients with breech presentation. The objective of our study was to evaluate the efficacy and perinatal outcomes among births with SGA infants after an ECV.

**Study Design**: This was a population based, cross sectional study using National Vitality Statistics data of singleton, non‐anomalous, neonates born after 36 weeks of gestation, with birthweight of <2500 g between 2021 and 2023. Births with a maternal history of previous cesarean delivery (CD) were excluded. Births were divided into those who had an ECV attempt and those who had a breech CD without a trial of labor. Adverse maternal outcomes (blood transfusion, ICU admission, unplanned hysterectomy) and neonatal outcomes (assisted ventilation use, NICU admission, seizures, surfactant use, death) were compared between groups using univariable and multivariable analysis.

**Results**: A total of 8021 births met inclusion criteria. Of the 8021 births, 812 (10.1%) had an ECV attempt and 7209 (89.8%) had a breech CD without a trial of labor. There were clinically significant differences in baseline characteristics between groups (Table 1). Among those who had an ECV attempt, 338 (41.6%) had a successful ECV, of which 233 (68.9%) subsequently delivered vaginally. After adjusting for clinical differences, births that had an ECV attempt had lower odds of neonatal assisted ventilation use (aOR 0.65, 95% CI 0.46‐ 0.92) and NICU admission (aOR 0.48, 95% CI 0.40‐0.58) than those who had a CD without a trial of labor. There were no significant differences in maternal outcomes between the two groups (Table 2).

**Conclusion**: Although ECV was successful in only 41.6% of births with SGA infants, there were lower rates of assisted ventilation use and NICU admission among those who had an ECV attempt.

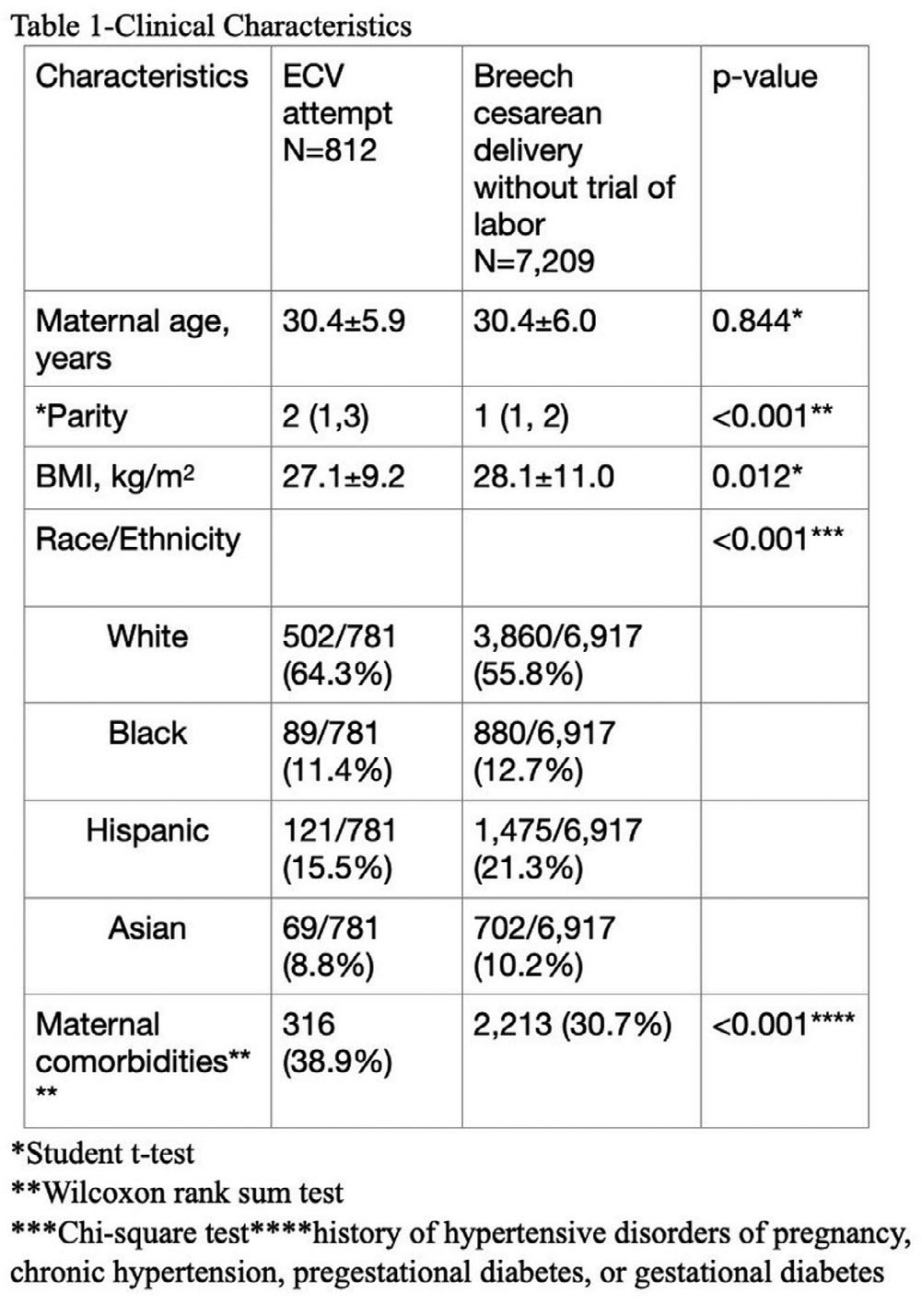

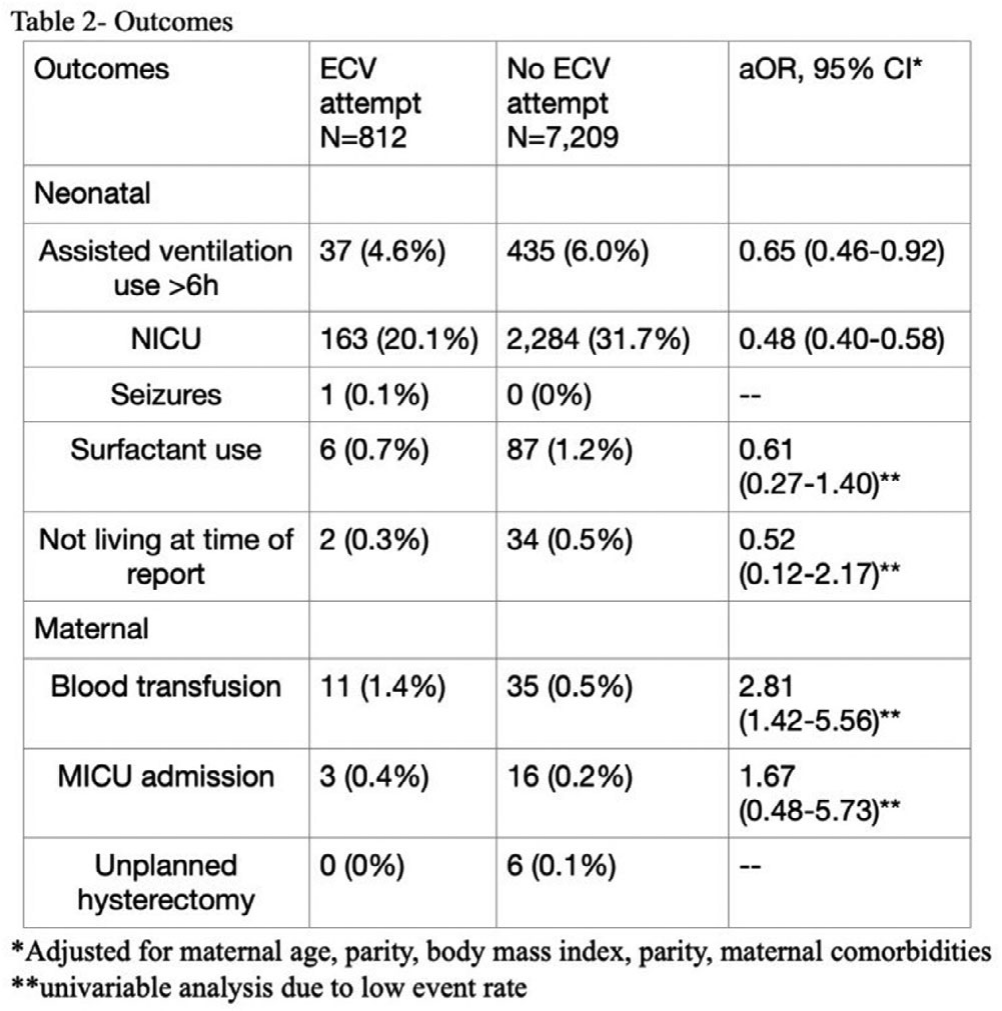

**10:30 AM – 12:00 PM**

## 359 | Triglyceride‐Glucose Index as a Novel Predictor of Insulin Therapy in Women With Gestational Diabetes

Schie Suga; Misao Fukuoka; Ichiro Yasuhi

NHO Nagasaki Medical Center, Omura, Nagasaki

**Objective**: The triglyceride‐glucose (TyG) index is an emerging surrogate marker of insulin resistance, but its utility in pregnancy is not well established. We aimed to assess whether the TyG index at the time of gestational diabetes (GDM) diagnosis independently predicts insulin therapy, and whether it performs better than fasting triglycerides (TG) and conventional glycemic markers.

**Study Design**: This prospective cohort included singleton pregnant women diagnosed with GDM at >= 24 weeks using a 75‐g OGTT. Fasting TG and glucose were measured at diagnosis, and the TyG index was calculated as ln[TG (mg/dL) × glucose (mg/dL)/2]. All women received nutritional therapy; insulin was initiated if glycemic targets were unmet. ROC analyses identified optimal cutoffs. Multivariable logistic regression evaluated the independent predictive value of ROC cutoff of TyG index and TG, adjusting for age, gestational age, HbA1c, glucose levels, and pre‐pregnancy BMI.

**Results**: Among 214 women (mean age 34 years; BMI 23.3 kg/m^2^), 45.3% required insulin. The insulin group had significantly higher TyG index (9.16 ± 0.44 vs. 8.87 ± 0.32, *p* < .0001) and TG levels (241 ± 112 vs. 188 ± 53 mg/dL, *p* < .0001). TyG index > = 9.19 (ROC cutoff) optimally predicted insulin therapy. In adjusted models, TyG index > = 9.19 was independently associated with insulin initiation (aOR 3.55, 95% CI 1.49–8.44, *p* = .0042). Compared to TG > = 269 mg/dL (ROC cutoff) (aOR 7.63, 95% CI 2.48–23.5), TyG index > = 9.19 showed higher sensitivity (75.5% vs. 40.2%) and negative predictive value (88.9% vs. 65.5%) (Table).

**Conclusion**: The TyG index is a novel and practical predictor of insulin therapy in GDM, demonstrating greater sensitivity and negative predictive value than fasting TG. As a simple and widely available metric reflecting metabolic risk, it may enhance early risk stratification and support individualized treatment approaches in GDM care.

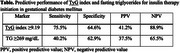

**10:30 AM – 12:00 PM**

## 360 | Association of High Allostatic Load in Early Pregnancy With Neonatal Weight and Body Composition

Sebastian Z. Ramos; Devika Lekshmi; Perrie O'Tierney‐Ginn

Tufts University School of Medicine, Boston, MA

**Objective**: Emerging evidence suggests that elevated maternal stress leads to higher fetal growth. These effects are thought to result from stress‐related alterations in neuroendocrine, metabolic, and inflammatory pathways that increase nutrient transfer to the fetus, and potentially fat accrual. However, few studies have examined the association between maternal physiological stress and neonatal body composition.

**Study Design**: This prospective cohort study included 43 low‐risk pregnant individuals. First‐trimester neuroendocrine, metabolic, and cardiovascular biomarkers associated with physiological stress were standardized using z‐scores and summed to calculate a total allostatic load score. Scores at or above the 75th percentile defined high Allostatic Load in Early Pregnancy (ALEP). High and low ALEP scores were compared to neonatal outcomes including birthweight and body composition (fat and fat‐free mass) measured via air displacement plethysmography (PeaPod). Group differences were assessed using t‐tests or Wilcoxon rank‐sum tests for continuous variables and chi‐square tests for categorical variables.

**Results**: In the 43 pregnancies, 11 (26%) had high Allostatic Load in Early Pregnancy (ALEP). Neonates born to individuals with high ALEP had significantly higher mean birthweight compared to those with low ALEP (3524 g vs. 3138 g, *p* = 0.027). Peapod‐derived measures showed a trend toward higher neonatal adiposity among the high ALEP group, including greater fat mass (*p* = 0.017) and overall body mass (*p* = 0.025), though differences in percent body fat and fat‐free mass did not reach statistical significance. No significant differences were observed in gestational age or neonatal sex distribution between groups.

**Conclusion**: Higher maternal allostatic load in early pregnancy was associated with greater birthweight and absolute neonatal fat mass in a diverse cohort of low‐risk pregnancies. Given that neonatal adiposity is correlated with childhood obesity risk, further research is needed to understand how maternal physiological stress contributes to future cardiometabolic risk in offspring.

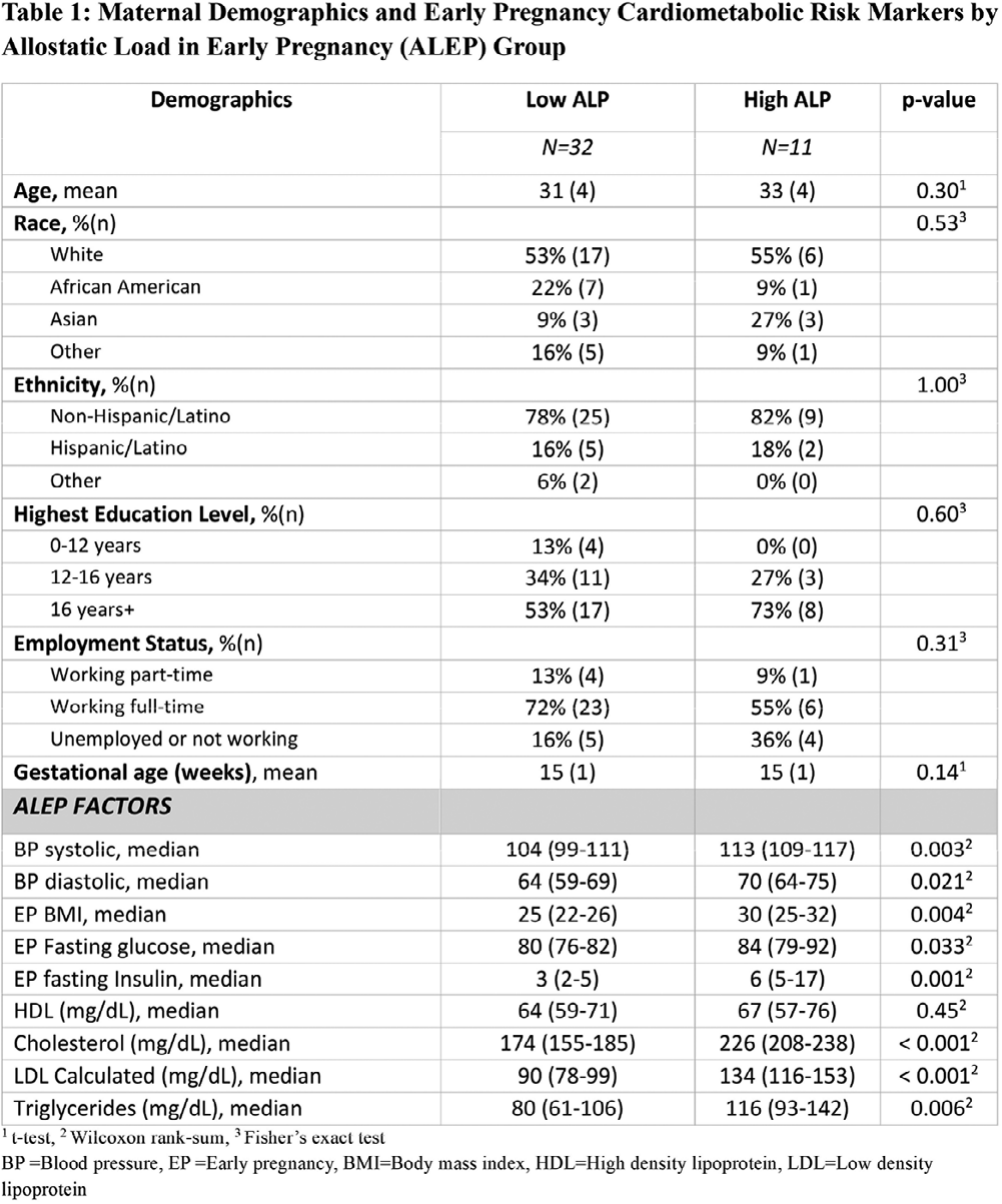

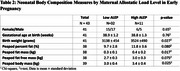

**10:30 AM – 12:00 PM**

## 361 | Premature Rupture of Membranes and Risk of Uterine Rupture in Trial of Labor After Cesarean

Shanny Kolp‐Asis^1^; Roi Yozevitch^2^; Elad Miron^3^; limor Vaknin Geron^3^; Elior Eliasi^4^; Ariel Many^5^; Tamar Tzur^5^

^1^Ma'ayanei Ha'yeshua Medical Center, bnei brak, HaMerkaz; ^2^Department of Computer and Software Engineering, Ariel University, Israel, Ariel, HaMerkaz; ^3^Department of OB&GYN, Mayaney Hayeshua Medical Center., bnei brak, HaMerkaz; ^4^Department of OB&GYN, Mayaney Hayeshua Medical Center, bnei brak, HaMerkaz; ^5^Department of OB&GYN, Mayaney Hayeshua Medical Center, bney brak, HaMerkaz

**Objective**: To evaluate whether premature rupture of membranes (PROM) is independently associated with an increased risk of uterine rupture in women undergoing a trial of labor after cesarean delivery (TOLAC).

**Study Design**: We conducted a retrospective cohort study including all singleton term pregnancies with one prior cesarean delivery attempting TOLAC at a tertiary care center between 2011 and 2023. PROM and uterine rupture cases were identified by ICD‐9 codes and validated by chart review. Multivariable logistic regression adjusted for parity, gestational age, birthweight, prior vaginal birth after cesarean (VBAC), and labor induction. Subgroup analyses compared rupture rates by PROM status within spontaneous and induced labor groups.

**Results**: Of 9551 TOLAC candidates, 1040 (10.9%) had PROM. Uterine rupture occurred in 37 cases (0.38%). The rupture rate was higher with PROM versus no PROM (1.0% vs. 0.3%; unadjusted OR 3.05, 95% CI 1.47–6.32; *p* = 0.002). PROM remained independently associated with rupture (adjusted OR 2.65, 95% CI 1.25–5.63; *p* = 0.011). Labor induction increased rupture risk (adjusted OR 2.78, 95% CI 1.41–5.46; *p* = 0.003), while higher parity was protective (adjusted OR 0.82, 95% CI 0.69–0.98; *p* = 0.026). In induced labors, rupture was more frequent with PROM (2.4% vs. 0.8%; *p* = 0.047). In spontaneous labors, PROM showed a nonsignificant trend toward increased rupture risk (0.6% vs. 0.2%; *p* = 0.073).

**Conclusion**: PROM independently increases uterine rupture risk in women undergoing TOLAC, with the greatest risk observed when induction is required. PROM should be recognized in risk stratification and counseling for TOLAC candidates.

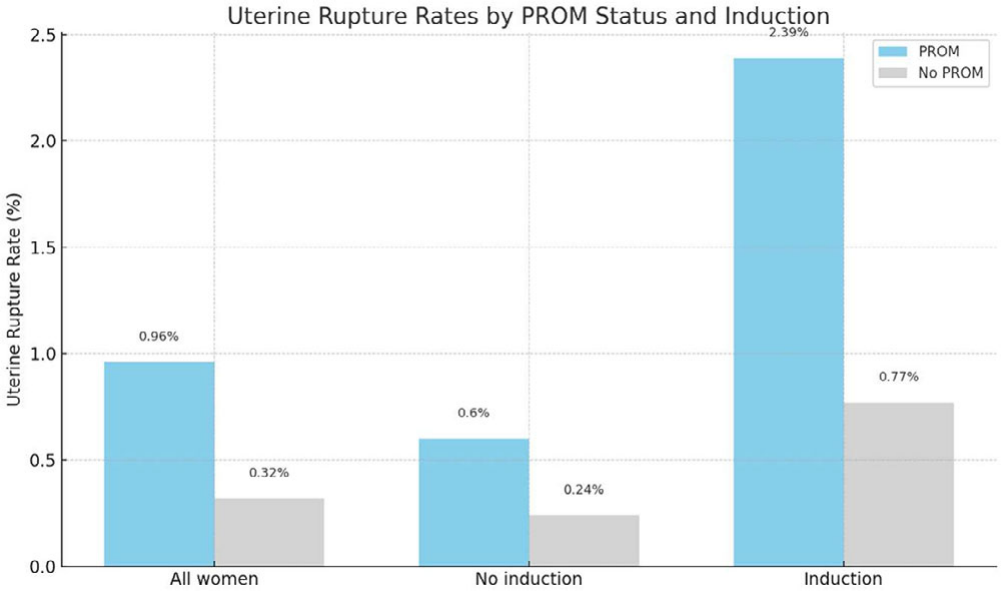

**10:30 AM – 12:00 PM**

## 362 | Impact of Antenatal Steroid Administration in the Late Preterm Period

Shay Kaufman^1^; Ran Matot^2^; Natav Hendin^3^; Ohad Houri^4^; Nir Sokolover^2^; Eran Hadar^5^; Yossi Geron^6^

^1^Rabin Medical Center, Petach‐Tikva, HaMerkaz; ^2^Rabin Medical Center, Helen Schnieder Hospital for Women, Petach‐Tikva, HaMerkaz; ^3^Rabin Medical Center, Petah Tikva, HaMerkaz; ^4^BCNatal Fetal Medicine Research Center, BARCELONA, Catalonia; ^5^Rabin Medical Center, Petach Tikva, HaMerkaz; ^6^Helen Schneider Hospital for Women, Rabin Medical Center, Petah Tikva, HaMerkaz

**Objective**: The administration of antenatal corticosteroids (ACS) during the late preterm period (34 0/7 ‐ 36 6/7 weeks) remains debated due to uncertainties regarding its efficacy in improving neonatal respiratory outcomes, particularly respiratory distress syndrome (RDS), and concerns about potential long‐term neurodevelopmental effects. This study aimed to evaluate the impact of ACS on RDS in late preterm neonates through a subgroup analysis.

**Study Design**: We conducted a retrospective cohort study at a tertiary medical center, analyzing all singleton late preterm deliveries between May 2016 and June 2023. Maternal and neonatal outcome data were extracted from electronic medical records. Women who received ACS before 34 0/7 weeks were excluded. A subgroup analysis was performed to identify clinical characteristics associated with potential ACS benefit.

**Results**: A total of 1967 late preterm neonates were included, with 372 (18.91%) receiving ACS, though only 202 (54.3%) completed the full dose. ACS did not significantly reduce RDS incidence (5.11% vs. 5.27%, *p* > 0.99). Further subgroup analyses, including gestational birth week, maternal hypertension, diabetes, preterm premature rupture of membranes, cesarean delivery, and low birth weight (<2500 g), showed no association between ACS and reduced RDS risk (Table 1).

**Conclusion**: To our knowledge, this is the largest and most recent study on this topic. Our findings suggest that ACS administration in the late preterm period does not improve lung maturity or reduce neonatal RDS risk, questioning its clinical utility in this population.

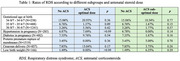

**10:30 AM – 12:00 PM**

## 363 | The Association Between Isolated Single Umbilical Artery and the Risk of Childhood Cardiac Disease

Shir Luvaton^1^; Tamar Wainstock^2^; Eyal Sheiner^3^; Gil Gutvirtz^4^

^1^Soroka Medical Center, Be'er Sheva, HaDarom; ^2^Department of Epidemiology, Biostatistics and Community Health Sciences, Faculty of Health Sciences, Ben‐Gurion University of the Negev, Beer Sheva, HaDarom; ^3^Soroka University Medical Center, Faculty of Health Sciences, Ben‐Gurion University of the Negev, beer sheva, HaDarom; ^4^Department of Obstetrics and Gynecology, Soroka University Medical Center, Ben‐Gurion University, Metar, HaDarom

**Objective**: Single umbilical artery (SUA), the most common umbilical cord abnormality, is often associated with congenital anomalies, including cardiac malformations. However, data on the long‐term cardiovascular impact of isolated SUA are limited. This study assessed whether isolated SUA is linked to an increased risk of childhood cardiovascular disease.

**Study Design**: A population‐based cohort study evaluated the risk of childhood cardiac disease (up to age 18) among infants with isolated SUA, born between 1991 and 2021 at a single medical center. Cases with fetal malformations or genetic abnormalities were excluded. Offspring cardiovascular morbidity was identified from clinic and hospital records and included decongestive heart failure, structural valvular disease, hypertension, arrhythmia, ischemic heart disease, peri‐myocarditis, rheumatic fever, pulmonary heart disease, and non‐specified disorders. A Kaplan‐Meier survival curve was used to compare the cumulative incidence of cardiovascular disorders between both study groups; a Cox proportional hazards model was applied to adjust for potential and relevant confounders.

**Results**: During the study period, 232,476 children were included, of whom 136 were diagnosed with isolated SUA. The overall cardiovascular morbidity was similar between children with or without SUA (Table). Similarly, the Kaplan‐Meier survival analysis showed comparable incidence in cumulative cardiovascular morbidity during the follow up time (Figure). The Cox proportional hazards model adjusted for maternal and gestational age, diabetes mellitus, hypertensive disorders, intrauterine growth restriction and cesarean delivery, reassured that isolated SUA was not associated with an increased risk for cardiovascular morbidity of the offspring (Table).

**Conclusion**: In this large population‐based cohort with long‐term follow‐up, isolated SUA was not associated with an increased risk of cardiovascular morbidity in offspring up to 18 years, offering reassurance for clinicians and parents regarding the long‐term cardiac prognosis of infants born with isolated SUA.

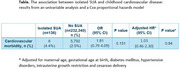

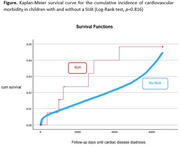

**10:30 AM – 12:00 PM**

## 364 | Transcutaneous Electrical Nerve Stimulation (TENS) for Post‐Cesarean Pain Relief: A Prospective Quasi‐Randomized Controlled Trial

Shir Zagdon Keidar^1^; Zehava Yohay^2^; Avi Barach^3^; Irena Shtilman^3^; Masho Salomon^1^; Eyal Sheiner^4^

^1^Soroka University Medical Center, Ben‐Gurion University of the Negev, Beer sheva, HaDarom; ^2^Soroka University Medical Center, Department of Obstetrics and Gynecology, Beer Sheva, Israel, Beer sheva, HaDarom; ^3^Soroka University Medical Center, Beer Sheva, Israel, Beer sheva, HaDarom; ^4^Soroka University Medical Center, Faculty of Health Sciences, Ben‐Gurion University of the Negev, beer sheva, HaDarom

**Objective**: Post‐cesarean pain management is a critical component of postoperative care, directly influencing maternal recovery. Despite widespread use of pharmacological analgesia, there is growing interest in non‐pharmacological methods such as transcutaneous electrical nerve stimulation (TENS). The present study aimed to evaluate the efficacy of TENS in reducing postoperative pain following cesarean delivery (CD), and to explore its potential impact on maternal‐infant bonding, postnatal anxiety, and depressive symptoms.

**Study Design**: A prospective quasi‐randomized controlled study included 140 women undergoing CD at a tertiary medical center. Participants received either postoperative TENS (intervention group, *n* = 70) or standard care (*n* = 70) per a predefined allocation scheme. Pain intensity was assessed before and after each TENS session using a visual analog scale (VAS). Bonding (PBQ), anxiety (STAI), and depression (EPDS) were evaluated using validated tools. The sample size was calculated to detect a 1.5‐unit difference in VAS scores (44 per group) and a 20% reduction in analgesic use (63 per group), with α = 0.05 and 80% power.

**Results**: Women in the TENS group experienced a significant reduction in VAS pain scores following each treatment session (Session 1: *p* < 0.001; Sessions 2 and 3: *p* = 0.009; Figure). A decrease was observed in the use of a specific oral analgesic (*p* = 0.012). This difference remained statistically significant after adjustment for maternal age (β = −0.2, 95% CI −0.8, −0.05; *p* = 0.029) or surgical history (β = −0.2, 95% CI −0.8,‐0.04; *p* = 0.032) using linear regression models. However, no significant differences were found between the groups in the overall proportion of women with high medication consumption during hospitalization, early postpartum mobility, maternal‐infant bonding, anxiety scores, or rates of postpartum depressive symptoms (EPDS ≥10; Table).

**Conclusion**: TENS therapy after CD significantly reduces postoperative pain, with the potential to reduce the use of oral analgesics. Larger studies are warranted to validate these findings and further explore psychosocial benefits.

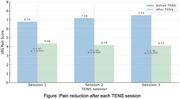

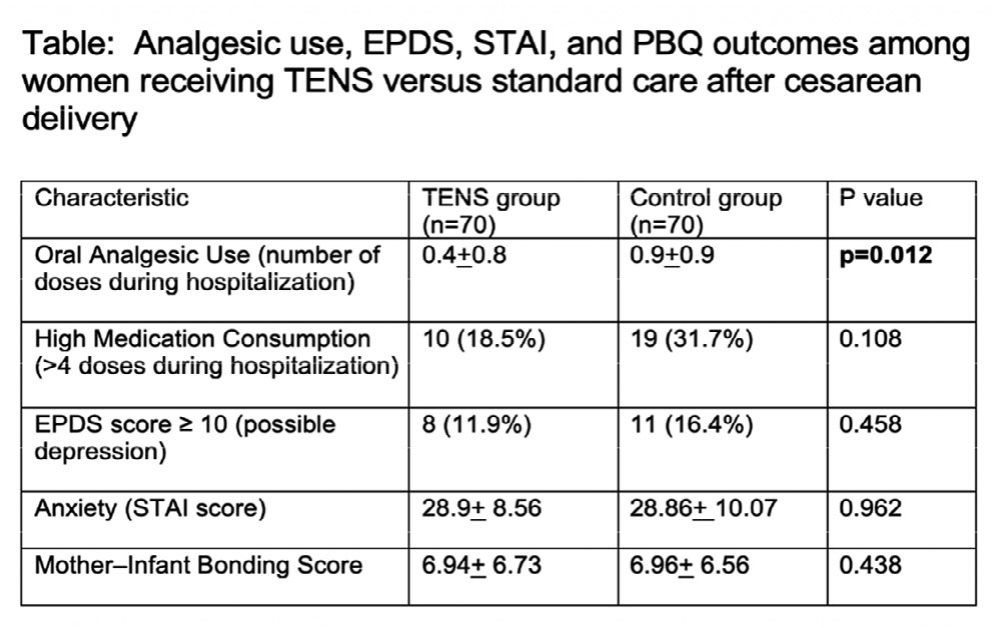

**10:30 AM – 12:00 PM**

## 365 | PPROM With Cerclage: Outcomes in the Absence of Clinical Chorioamnionitis and Insights From Amniocentesis

Shiran Bookstein^1^; Chen Lugacy^2^; Yossi Bart^3^; Sharon Amit^4^; Lital Shaham^5^; Abraham Tsur^6^; Shali Mazaki Tovi^5^; yoav yinon^1^; Michal Fishel Bartal^7^

^1^Sheba Medical Center, Ramat Gan, Israel, Ramat Gan, HaMerkaz; ^2^Sheba medical center, Ramat Gan, Tel Aviv; ^3^Division of Maternal‐Fetal Medicine, Department of Obstetrics, Gynecology and Reproductive Sciences, McGovern Medical School at UTHealth Houston, Houston, TX; ^4^Sheba Medical Center, Tel HaShomer, HaMerkaz; ^5^Sheba Medical Center, Ramat Gan, Israel, ramat gan, HaMerkaz; ^6^Department of Obstetrics and Gynecology, Sheba Medical Center, Sheba Medical Center, HaMerkaz; ^7^Division of Maternal‐Fetal Medicine, Department of Obstetrics, Gynecology and Reproductive Sciences, Sheba Medical Center, Tel Aviv University, Israel, Ramat Gan, HaMerkaz

**Objective**: Premature preterm rupture of membranes (PPROM) in patients with cerclage is associated with adverse outcomes. This study aimed to determine maternal and neonatal outcomes in individuals with cervical cerclage presenting with PPROM prior to 32 weeks, and to assess the yield of amniocentesis (AC) in identifying subclinical intra‐amniotic infection

**Study Design**: This single‐center retrospective study included individuals with cerclage and PPROM <32 weeks between 03/2010–06/2025. Exclusion criteria were clinical chorioamnionitis at presentation (fever, uterine tenderness, tachycardia, leukocytosis, or non‐reassuring fetal monitoring) or active labor. Standard practice at our center for asymptomatic patients included AC; negative AC prompted expectant inpatient care, whereas positive AC led to delivery. Maternal and neonatal outcomes were compared according to AC results

**Results**: Of 114 patients with cerclage and PPROM, 45 (39%) met eligibility criteria; 21 (47%) had positive AC. Baseline characteristics were comparable (Table 1). A wide variety of pathogens was identified, with some amniocentesis samples yielding multiple organisms. The most common organisms were Enterobacterales/other Gram‐negatives (*n* = 8), followed by *Streptococcus anginosus/milleri* (*n* = 5), anaerobes (*n* = 4), *Candida* (*n* = 2), *Staphylococcus aureus* (*n* = 2), Enterococci (*n* = 2), and GBS (*n* = 2). Positive AC was associated with higher rates of histologic chorioamnionitis (71.4% vs 33.3%, *p* = 0.01). All intrapartum fetal deaths occurred in the positive AC group. Gestational age at delivery was similar, but birthweight and 1 and 5 minute Apgar scores were lower in the positive AC group. No differences were observed in composite adverse neonatal outcomes (Table 2)

**Conclusion**: Nearly half of patients with cerclage and PPROM <32 weeks without clinical signs of infection had intra‐amniotic infection detected by AC. Despite longer latency in AC‐negative individuals managed expectantly, adverse neonatal outcomes were similar to those with positive AC, highlighting the need for further research to clarify the impact of AC‐guided management on perinatal outcomes

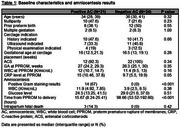

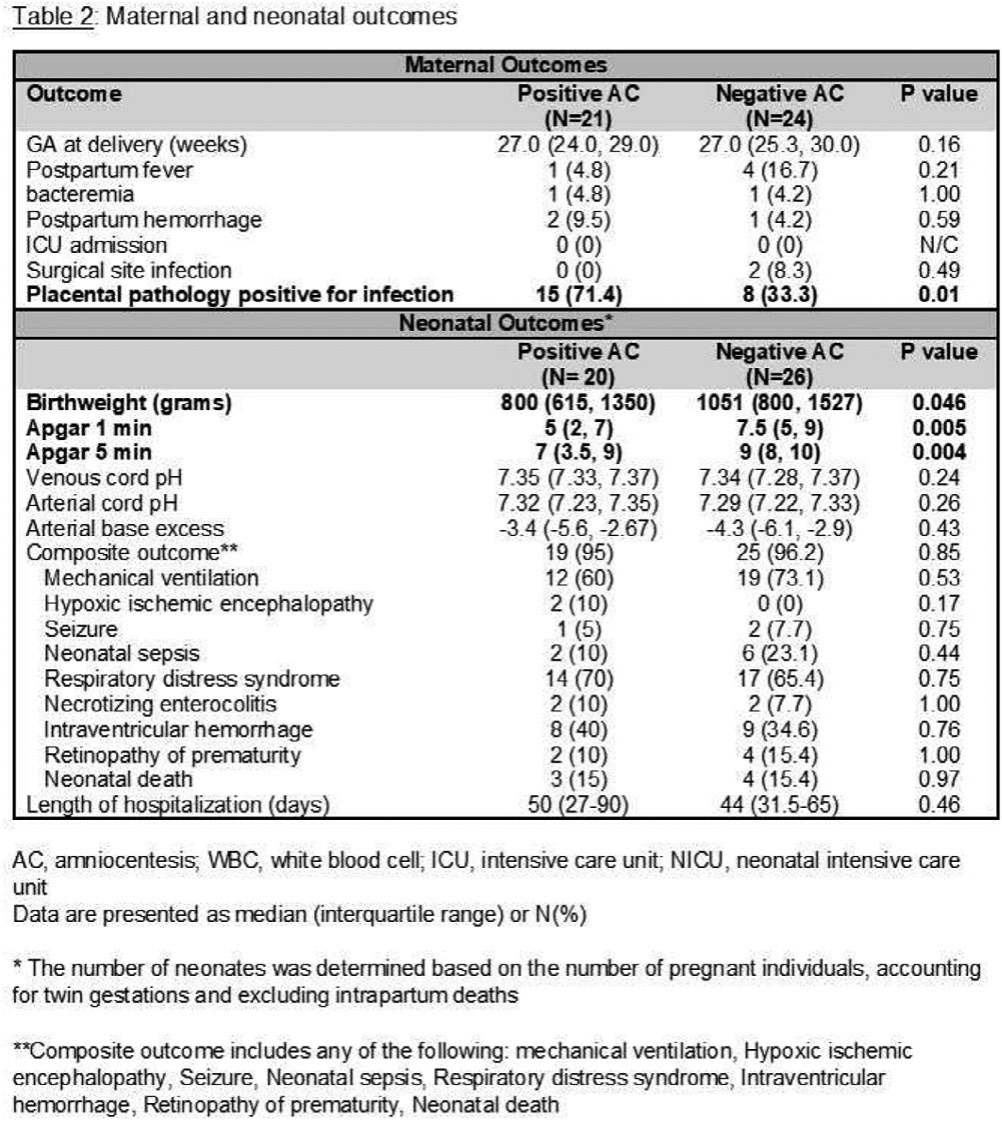

**10:30 AM – 12:00 PM**

## 366 | Nursing Perspectives About Universal Inpatient Postpartum Depression Screening

Shobha Jagannatham^1^; Anna Cavallino^2^; Marlee Madora^1^; Rubiahna L. Vaughn^1^; Kavita Vani^1^

^1^Albert Einstein College of Medicine/Montefiore Medical Center, Bronx, NY; ^2^Albert Einstein College of Medicine/Montefiore Medical Center, Brooklyn, NY

**Objective**: Nursing‐led interventions may improve screening and referrals for postpartum depression. We aimed to assess the acceptability of nursing involvement in a quality improvement intervention to enhance depression screening on inpatient postpartum units.

**Study Design**: In April 2024, postpartum nurses began administering the Edinburgh Postnatal Depression Scale (EPDS) to all delivered patients during their hospital stay. Nurses received training on EPDS administration and scoring. A positive screen (EPDS ≥10) prompted referral to social work and a request to the obstetric provider for a reproductive psychiatry consult. Following implementation, we conducted a cross‐sectional survey of postpartum nurses to assess the intervention's feasibility and acceptability. The survey included Likert‐scale items evaluating nurses’ comfort with administering, scoring, and discussing results, perceived patient comfort, and time required for screening. For analysis, the first two choices on the Likert scale were categorized as affirmative responses (i.e., indicating agreement or endorsement of the item).

**Results**: Of eligible nurses, 78.5% (*n* = 51/65) completed the survey. Most nurses reported feeling comfortable screening patients (98.0%, *n* = 50), scoring the EPDS (96.0%, *n* = 49), discussing mental health (94.1%, *n* = 48), and providing referrals (94.1%, *n* = 48). A majority preferred administering a paper version (72.5%, *n* = 37). Reported barriers to addressing mental health included patients’ focus on their newborn, confidentiality concerns, and stigma. Most nurses (78.4%, *n* = 40) reported that screening and discussing results with providers took ≤30 minutes with 45.1% (*n* = 23) indicating the process took <15 minutes.

**Conclusion**: Universal inpatient postpartum depression screening is both feasible and acceptable to nurses. In settings where providers have limited time with patients, nurses can play a key role in supporting postpartum mental health and facilitating timely referrals to behavioral health services.

**10:30 AM – 12:00 PM**

## 367 | Determinants of COVID‐19 Vaccine Uptake Among Low‐Income, Latinx Pregnant Individuals in California's San Joaquin Valley

Shreya S. Gunda^1^; Kesia K. Garibay^2^; Daisy León‐Martínez^3^; Bethany Simard^4^; Miriam Kuppermann^5^

^1^University of California, San Francisco, San Jose, CA; ^2^University of the Pacific, Stockton, CA; ^3^Division of Maternal‐Fetal Medicine at the University of California San Francisco, University of California San Francisco, CA; ^4^Department of Obstetrics, Gynecology and Reproductive Sciences, University of California, San Francisco, UCSF, CA; ^5^Department of Obstetrics, Gynecology and Reproductive Sciences, University of California, San Francisco, University of California San Francisco, CA

**Objective**: To investigate the association between COVID‐19 vaccination uptake and demographic, socioeconomic, and clinical factors among low‐income pregnant and postpartum individuals in California's San Joaquin Valley.

**Study Design**: This is a secondary analysis of data collected as part of the EMBRACE study, a randomized trial comparing the effectiveness of two enhanced prenatal care models. Participants were low‐income pregnant individuals, 72% of whom identified as Latinx. We conducted bivariate analyses and a stepwise logistic regression to determine the association between participant characteristics and COVID‐19 vaccine uptake. Covariates included age, ethnicity, birthplace, language of interviews, educational attainment, monthly household income, relationship status, parity, and diagnosis of mental health condition.

**Results**: Among the 652 study participants, 61.5% reported having received a COVID‐19 vaccine. 45.7% were vaccinated before pregnancy, 9.6% during pregnancy, and 6.1% after giving birth. In the stepwise multivariable logistic regression model, older age was associated with higher odds of being vaccinated (OR = 1.09, 95% CI: 1.04–1.14, *p* < 0.001). Participants with one to two children had significantly lower odds of vaccination compared to those without children (OR = 0.34, 95% CI: 0.20–0.56, *p* < 0.001), as did those with three or more children (OR = 0.27, 95% CI: 0.13–0.55, *p* < 0.001).

**Conclusion**: Age and parity are associated with COVID‐19 vaccination uptake in this population. Older individuals were more likely to be vaccinated. However, individuals with children had significantly lower odds of vaccine uptake, suggesting the need to explore caregiving‐related barriers. We should consider outreach strategies to improve vaccination access and acceptance among younger people and parents.

**10:30 AM – 12:00 PM**

## 368 | **The Impact of Immigration Status on Peripartum Care in the United States: A Scoping Review**

Shruthi Manjunath^1^; Douglas McHugh^2^; Sara H. Rahman^3^

^1^Frank H. Netter School of Medicine at Quinnipiac University, North Haven, CT; ^2^Frank H. Netter MD School of Medicine at Quinnipiac University., North Haven, CT; ^3^Cleveland Clinic Foundation, Cleveland, OH

**Objective**: This scoping review examines the impact of immigration status on access to and quality of peripartum care in the U.S., with a focus on federal and state policy variations.

**Study Design**: A comprehensive literature search was conducted across APA PsycINFO, CINAHL Ultimate, Cochrane Library, PubMed, and Scopus for peer‐reviewed articles published in the past 10 years. Eligible studies included empirical and theoretical research examining pregnant or postpartum individuals in the U.S. whose immigration status was explicitly identified (e.g., undocumented, DACA recipients, lawful permanent residents, refugees). Studies were required to assess the impact of one or more immigration‐ and healthcare‐related policies—drawn from a predefined list—on access to or quality of peripartum care. Exclusions included studies lacking legal status details, addressing only racial/ethnic disparities, or focusing solely on pediatric or breastfeeding outcomes. Two independent reviewers and an adjudicator screened 1602 articles; 32 met inclusion criteria. Thematic analysis was conducted in MAXQDA using deductive and inductive coding.

**Results**: Included studies varied in methods and policy focus. Peripartum care access varied widely for undocumented immigrants, with only a subset of states offering continuous coverage from pregnancy through 12 months postpartum. Undocumented and other non‐citizen individuals were more likely to receive inadequate prenatal care and face barriers to postpartum care. Anti‐immigration sentiments and policies, such as the Public Charge Rule, deterred Medicaid enrollment and were linked to adverse birth outcomes. In contrast, expanded Emergency Medicaid and state programs improved care utilization. Key facilitators included language access, culturally competent care, and extended postpartum coverage.

**Conclusion**: In the context of recent Medicaid cuts and rising anti‐immigration sentiment, this review highlights how immigration status continues to shape peripartum care access and outcomes. Inclusive policies that reduce barriers and increase coverage are essential for advancing maternal health equity.

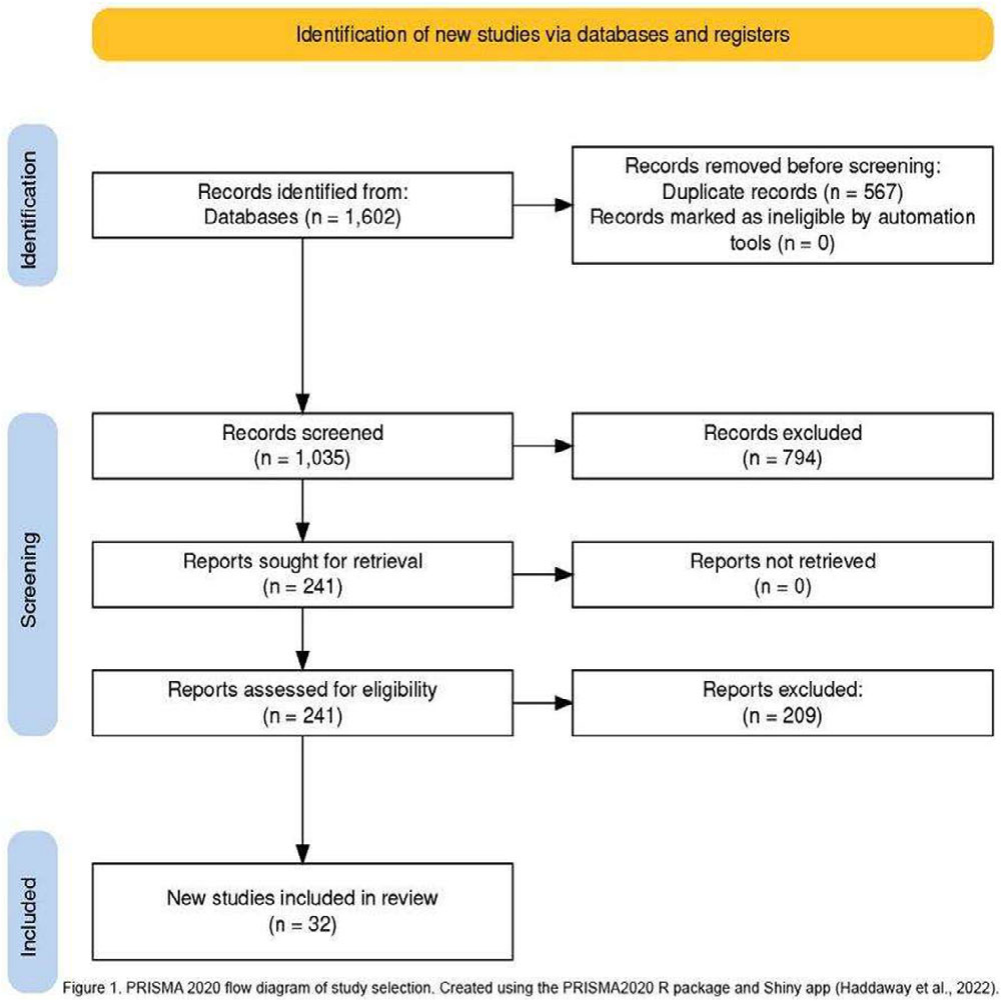

**10:30 AM – 12:00 PM**

## 369 | Obstetrical Outcomes Following External Cephalic Version (ECV)

Sivan Chocron^1^; Tamar Wainstock^2^; Eyal Sheiner^3^; Polina Schwarzman^4^

^1^Department of Obstetrics and Gynecology, Soroka University Medical Center, Ben‐Gurion University, Beer Sheva, HaDarom; ^2^Department of Epidemiology, Biostatistics and Community Health Sciences, Faculty of Health Sciences, Ben‐Gurion University of the Negev, Beer Sheva, HaDarom; ^3^Soroka University Medical Center, Faculty of Health Sciences, Ben‐Gurion University of the Negev, beer sheva, HaDarom; ^4^Soroka University Medical Center, Soroka University Medical Center, HaDarom

**Objective**: External cephalic version (ECV) is a well‐established obstetric procedure aimed at reducing the incidence of term breech presentation. Although considered safe and effective when performed according to guidelines, its impact on labor and immediate obstetrical outcomes remains debated. This study aimed to assess the association between labor following successful ECV and maternal and neonatal outcomes.

**Study Design**: A retrospective cohort study was conducted, including all singleton term deliveries at a tertiary medical center between 1991 and 2021. Deliveries following a successful ECV were compared with deliveries without prior ECV. Maternal characteristics, obstetrical complications, and neonatal outcomes were analyzed. Multivariable logistic regression model was used to adjust for potential confounders.

**Results**: Among 185,467 deliveries, 81 (0.04%) followed a successful ECV. Women delivering after ECV were more likely to be multiparous and to have gestational or pregestational diabetes (*p* = 0.000). Labor following ECV was associated with higher rates of labor induction (38.2% vs. 21.2%, *p* = 0.000) and umbilical cord prolapse (3.7% vs. 0.4%, *p* = 0.000). Cesarean delivery (CD) rates were higher following ECV (11.1% vs. 5.0%, *p* = 0.012), however, this association did not persist after adjusting for potential confounders such as labor induction, in a multivariable model (aOR 1.57, 95% CI 0.76–3.23, *p* = 0.219). No significant differences were observed in major adverse obstetrical outcomes or neonatal outcomes, including umbilical cord pH <7, fetal distress, low 5‐minute Apgar score (<7), meconium‐stained amniotic fluid, or perinatal mortality.

**Conclusion**: Successful ECV is not associated with an increased risk of severe adverse obstetrical or neonatal outcomes. Nevertheless, higher rates of umbilical cord prolapse warrant heightened vigilance during labor management following ECV.

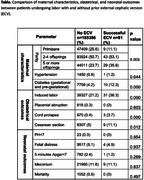

**10:30 AM – 12:00 PM**

## 370 | Hypertension‐Related Postpartum Morbidity Following Discharge: A Comparison of Nifedipine and Labetalol Monotherapy

Sonya Fabricant^1^; Natalie A. Bello^2^; Emily Seet^3^; Sarah Kilpatrick^3^; Mariam Naqvi^3^

^1^Cedars‐Sinai Medical Center, Los Angeles, CA; ^2^Department of Cardiology, Cedars‐Sinai Medical Center, Los Angeles, CA; ^3^Department of Obstetrics and Gynecology, Cedars‐Sinai Medical Center, Los Angeles, CA

**Objective**: To evaluate rates of hypertension‐related morbidity after delivery discharge among individuals on nifedipine vs labetalol monotherapy.

**Study Design**: This was a retrospective cohort study of individuals who delivered at a quaternary care center from 1/1/21–1/20/24 and were discharged on nifedipine or labetalol monotherapy. The primary outcome was hypertension‐related morbidity within 30 days of delivery discharge, defined as a new diagnosis of: preeclampsia lab abnormalities, HELLP syndrome, eclampsia, pulmonary edema/acute heart failure, cerebrovascular disorders, myocardial infarction/cardiac arrest, liver hematoma, acute renal failure and ICU admission. Secondary outcomes included 30‐day hospital re‐presentation and new‐onset severe preeclampsia. Baseline characteristics were abstracted from the medical record by an Informatics team. Chart review was performed for post‐discharge outcomes. Bivariate analysis was used to compare groups. Multivariable logistic regression was used to adjust for baseline characteristics and hypertension readmission risk factors.

**Results**: Of 361 patients meeting criteria, 33.2% were discharged on nifedipine and 66.8% on labetalol. Nifedipine recipients had lower BMI and were less likely to have preexisting renal disease or chronic hypertension compared to labetalol recipients (Table 1). Those on nifedipine had higher maximum systolic and diastolic blood pressure (BP) from delivery to discharge, but lower maximum systolic BP in the 24h prior to discharge. Individuals on nifedipine had lower rates of hospital re‐presentation (12.5 vs 4.6%, *p* = 0.006), new‐onset severe preeclampsia (2.5 vs 0%, *p* = 0.01) and hypertension‐related morbidity (3.3 vs 0.4%, *p* = 0.03) (Table 2). After controlling for covariates (renal disease, race, BMI and maximum BP in the 24h prior to discharge), nifedipine was independently associated with reduced odds of post‐discharge morbidity (aOR 0.09, 95% CI 0.01—0.8; *p* = 0.03).

**Conclusion**: Among individuals on antihypertensive monotherapy at delivery discharge, nifedipine was associated with reduced odds of post‐discharge morbidity compared to labetalol.

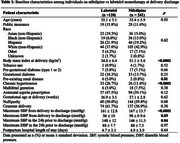

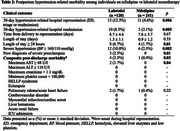

**10:30 AM – 12:00 PM**

## 371 | Outpatient Foley vs Inpatient Combined Cervical Ripening: Impact on Time to Delivery in Nulliparous Individuals

Sonya Fabricant^1^; Camelita Thrift^2^; Lauren McCarthy^2^; Glenda Marshall^2^; Mackenzie Bennett^3^; Mariam Naqvi^3^; Naomi H. Greene^2^; Emily Seet^3^

^1^Cedars‐Sinai Medical Center, Los Angeles, CA; ^2^Department of Obstetrics & Gynecology, Cedars‐Sinai Medical Center, Los Angeles, CA; ^3^Department of Obstetrics and Gynecology, Cedars‐Sinai Medical Center, Los Angeles, CA

**Objective**: To assess time to delivery with outpatient foley balloon (oFB) vs inpatient combined cervical ripening methods among low‐risk nulliparous individuals.

**Study Design**: This was a retrospective cohort study of individuals undergoing induction of labor at a quaternary care center (delivery date 8/1/2024–6/27/2025). Cases had oFB placement. Controls underwent inpatient cervical ripening via foley ballon (FB) plus prostaglandin E1, prostaglandin E2 or oxytocin. Inclusion criteria were nulliparity, singleton gestation, delivery gestational age 39w0d–41w6d, cervical dilation ≤3 cm at FB placement and low‐risk pregnancy. The primary outcome was time from admission to delivery. Secondary outcomes were time to delivery <24 hours, cesarean delivery, hospital length of stay, blood loss, clinical diagnosis of intrauterine infection, 5‐minute APGAR <7, umbilical artery pH <7 or base deficit ≥ 12 and NICU admission. Baseline characteristics and outcomes were compared between groups. Our study was powered to detect a 6‐hour difference in time to delivery using a two‐tailed test with an alpha level of 0.05 and 80% power.

**Results**: Of 197 eligible individuals, 31 (15.7%) underwent oFB placement. Baseline characteristics did not differ between cases and controls (Table 1). Cases had greater admission cervical dilation (3.8 vs 1.1 cm, *p* < 0.0001) and Bishop score (6.5 vs 4.6, *p* < 0.001). However, cases had longer duration of oxytocin administration (20.3 vs 17.0 hours, *p* = 0.04) and ruptured membranes (15.6 vs 12.2 hours, *p* = 0.02) (Table 2). Time to delivery was similar between cases and controls (22.8 vs 22.2 hours, *p* = 0.72). There was no statistically significant difference in cesarean delivery rates (cases 16.1% vs controls 20.5%, *p* = 0.58). Remaining maternal and neonatal outcomes were also similar.

**Conclusion**: Among low‐risk nulliparous individuals undergoing term induction of labor, oFB placement did not reduce time to delivery compared to inpatient combined cervical ripening methods, despite a more favorable Bishop score at admission. Larger studies are needed to determine if oFB impacts cesarean delivery rate.

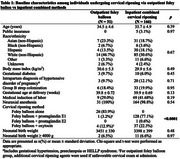

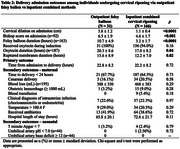

**10:30 AM – 12:00 PM**

## 372 | Cost‐Effectiveness of Maternal RSV Vaccination in Preventing RSV‐Related Hospitalizations in Infants

Sophia V. Gomez^1^; Amelia H. Gagliuso^1^; Aaron B. Caughey^2^

^1^Oregon Health and Science University, Portland, OR; ^2^Oregon Health & Science University, Oregon Health & Science University, OR

**Objective**: Respiratory syncytial virus (RSV) is a leading cause of hospitalization among infants under 6 months of age. Maternal vaccination offers passive protection to neonates through vaccine‐induced antibodies crossing the placenta. This study evaluates the cost‐effectiveness of maternal RSV vaccination compared to no vaccination in preventing RSV‐related hospitalizations in infants in the United States.

**Study Design**: A decision‐analytic model was developed to evaluate outcomes in a theoretical U.S. birth cohort of 3,664,292 individuals, the estimated number of annual live births in the United States. The model incorporated neonatal outcomes including stillbirth, RSV‐related hospitalizations, infant death, and development of asthma by 6 years of age. Input values for probabilities, costs, and utilities were derived from literature, and quality‐adjusted life years (QALYs) were discounted at an annual rate of 3%. An incremental cost‐effectiveness ratio (ICER) of less than $100,000 per QALY was considered cost effective.

**Results**: Maternal RSV vaccination prevented 63,435 RSV infections and reduced RSV‐related hospitalizations by 45,363 cases in our theoretical cohort (Table 1). Maternal RSV vaccination also reduced infant death by 100 cases and asthma diagnosis by age 6 by 13,469 cases. Vaccine‐induced maternal antibodies was the dominant strategy as it led to an additional 113,522 QALYs with a difference in cost of $124,146,213 resulting in an ICER of $1,093 per QALY.

**Conclusion**: Maternal RSV vaccination is a cost‐effective strategy for preventing and reducing RSV‐related morbidities in infants, even when accounting for the cost of vaccination. Incorporating RSV vaccination into routine prenatal care and ensuring all pregnant people are aware of its protective benefits are key to achieving better neonatal health outcomes.

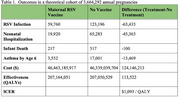

**10:30 AM – 12:00 PM**

## 373 | Impact of Maternal Obesity on Ultrasound Clearance Rates of Fetal Cardiac and Spine Anatomy

Stephanie Stokes^1^; Kathryne Kostamo^2^; Gillian Bremer^3^; Emily Wheeler^4^; Raeshmma Rajesh^1^; Erica Nicasio^5^

^1^Albany Medical Center, Albany Medical Center, NY; ^2^Albany Medical Center, Albany, NY; ^3^Albany Medical College, Albany Medical College, NY; ^4^Albany Medical College, Albany, NY; ^5^Hartford Healthcare, Hartford, CT

**Objective**: Rates of maternal obesity has risen substantially, presenting diagnostic challenges and increased risks of fetal anomalies. Cardiac and neural tube defects are frequently limited by poor acoustic windows in patients with elevated body mass index (BMI). This study aimed to evaluate the impact of maternal obesity (BMI >30) on completion rates of key cardiac and spine views before the implementation of the AIUM first‐trimester detailed protocol.

**Study Design**: This IRB‐approved retrospective cohort study included singleton pregnancies with maternal BMI >30 undergoing anatomy ultrasound between 18 to 22 + 6 weeks at Albany Medical Center and two affiliated sites from 06/01/23 to 12/01/23. Patients were stratified by BMI into 3 groups: 30.0–39.9, 40.0–49.9, and ≥50.0. Exclusion criteria were multifetal gestation, known fetal anomalies, BMI <30.0, or no follow‐up imaging. Ultrasounds were performed by registered sonographers supervised by five board‐certified maternal‐fetal medicine faculty. Cardiac views were considered complete if four‐chamber and both outflow tracts were visualized. Craniospinal anatomy was complete if the cranium, ventricles, cerebellum, cisterna magna, and full spine were seen.

**Results**: Multivariable logistic regression showed patients BMI >40 had significantly lower cardiac anatomy visualization rates, ranging from 38–70%, with the four‐chamber and outflow tract views most frequently incomplete (Figure 1). Spine visualization was also limited, with lumbar and sacral segments most commonly affected. Patients with BMI 30–39.9 served as the reference group, and those with BMI ≥40 had up to a 5.17‐fold increased odds of incomplete spine imaging (Figure 2). Among those with BMI ≥50, complete visualization occurred in only 28% of cases initially improving to 76% with multiple scans.

**Conclusion**: Maternal obesity limits cardiac and spine visualization on ultrasound, particularly BMI ≥40. Early and repeat imaging improve overall clearance rates, but limitations persist. These findings support the integration of standardized protocols and early detailed ultrasound in high‐BMI patients

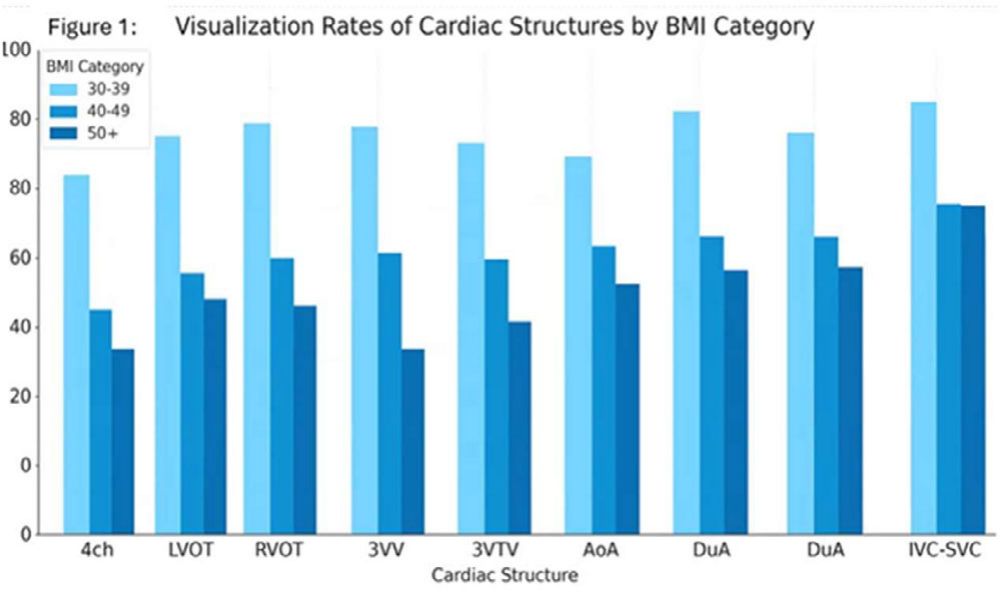

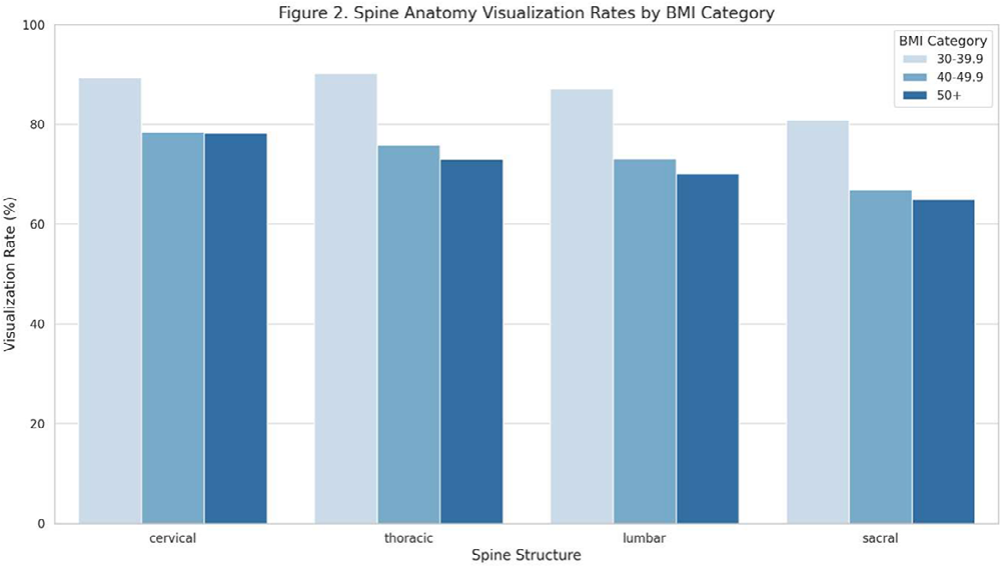

**10:30 AM – 12:00 PM**

## 374 | Beyond the Knot: VBAC Success After Knotless Barbed Versus Conventional Uterine Closure Sutures

Suha Seh

Ziv Medical Center, Israel, HaZafon

**Objective**: To evaluate whether uterine closure with knotless barbed sutures (Stratafix) during primary cesarean delivery is associated with the rate of successful vaginal birth after cesarean (VBAC), as well as the incidence of uterine rupture and dehiscence, compared to conventional smooth sutures (Vicryl), among women undergoing a subsequent delivery at the same institution.

**Study Design**: We conducted a retrospective case‐control study of women who underwent a primary cesarean at Ziv Medical Center (2015–2018) and returned for a subsequent delivery. Patients were grouped based on the suture type used for uterine closure: Vicryl (smooth) vs. Stratafix (knotless barbed). Outcomes included VBAC success (primary) and rates of uterine rupture and dehiscence (secondary).

**Results**: A total of 284 women were included: 213 had uterine closure with conventional smooth sutures (Vicryl) and 71 with knotless barbed sutures (Stratafix) during their primary cesarean. Baseline characteristics—age, gravidity, parity, BMI, and gestational age at subsequent delivery—were comparable between groups.

Elective repeat cesarean section (ERCS) was chosen by 117 women (55%) in the Vicryl group and 46 (65%) in the Stratafix group. Trial of labor after cesarean (TOLAC) was attempted by 96 (45%) in the Vicryl group and 31 (43%) in the Stratafix group.

VBAC success rates were similar: 79% in the Vicryl group vs. 80% in the Stratafix group. Surgical complications, including uterine dehiscence and rupture, were lower in the Stratafix group, though numbers were too small for statistical significance.

**Conclusion**: Uterine closure with knotless barbed sutures (Stratafix) during primary cesarean delivery was not associated with differences in VBAC success rates or the incidence of uterine rupture and dehiscence compared to conventional smooth sutures (Vicryl). However, Stratafix may offer operative advantages, including reduced surgical time and blood loss. While these findings support the safety and potential efficiency benefits of barbed sutures, larger studies are warranted to confirm these results and strengthen clinical recommendations.

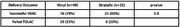

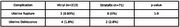

**10:30 AM – 12:00 PM**

## 375 | Patient and County‐Level Factors Associated With Trial of Labor After Cesarean Access and Success

Sumaiya M. Mubarack^1^; Sarah E. Little^2^; Taylor S. Freret^1^

^1^Beth Israel Deaconess Medical Center, Boston, MA; ^2^Beth Israel Deaconess Medical Center, Newton, MA

**Objective**: Because trial of labor after cesarean (TOLAC) carries risks, ACOG recommends TOLAC be attempted in facilities able to respond to such risks, which may limit access at the county level. We sought to analyze patient and county‐level factors associated with TOLAC access and VBAC success.

**Study Design**: This is a retrospective cross‐sectional study of county‐identified US natality data from 2016–2021. Individuals with one prior cesarean birth and a liveborn singleton, cephalic, non‐anomalous infant delivered between 39 and 42 weeks were included. Those with prior vaginal birth, diabetes, or chronic hypertension were excluded. TOLAC status was determined from the birth certificate. Overall TOLAC rates and VBAC rates among individuals who attempted TOLAC were calculated at the county level. Counties with TOLAC or VBAC rates in the top and bottom quartile were compared to each other. A patient‐level logistic regression was used to identify patient and county‐level factors associated with the primary outcomes. County‐level factors of interest were obtained from the 2021 Area Health Resource Files and standardized using z‐scores.

**Results**: Of 809,969 eligible individuals, 168,574 (20.8%) attempted TOLAC. Of the 1667 counties in the dataset, 1409 (84.5%) reported at least one TOLAC. The median TOLAC rate was 12.2% [IQR: 3.1–22.5%]; there was significant geographic variation (Figure). Factors associated with counties with high TOLAC rates included: younger age, more education, lower BMI, urban settings, more Ob‐Gyn providers and NICU beds, and lower unemployment rates (Table). The median VBAC rate was 60.4% [IQR: 46.6–70.6%]. Factors associated with lower rates of VBAC success included Black race, relatively smaller metropolitan area, fewer Ob‐Gyn providers, and higher rates of uninsurance (Table).

**Conclusion**: There is widespread variation in TOLAC access in the United States. Patient‐ and county‐level factors associated with TOLAC access and VBAC success differ, which highlights potential disparities and limitations in access to care.

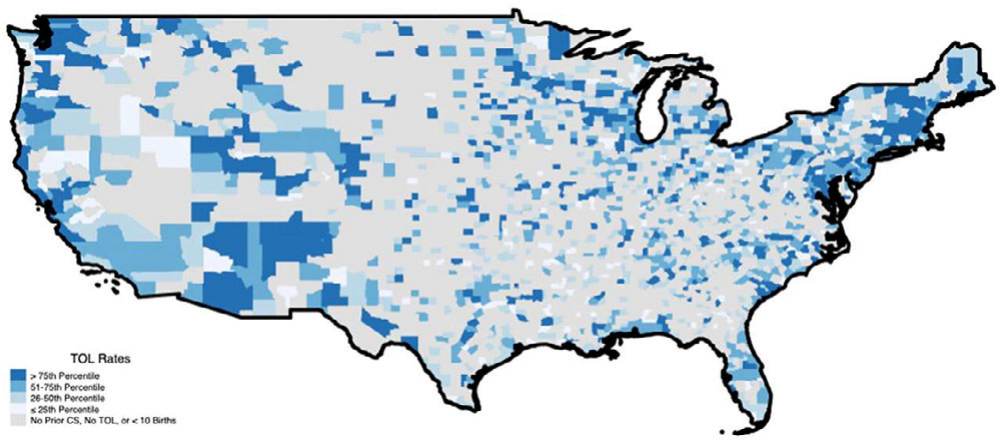

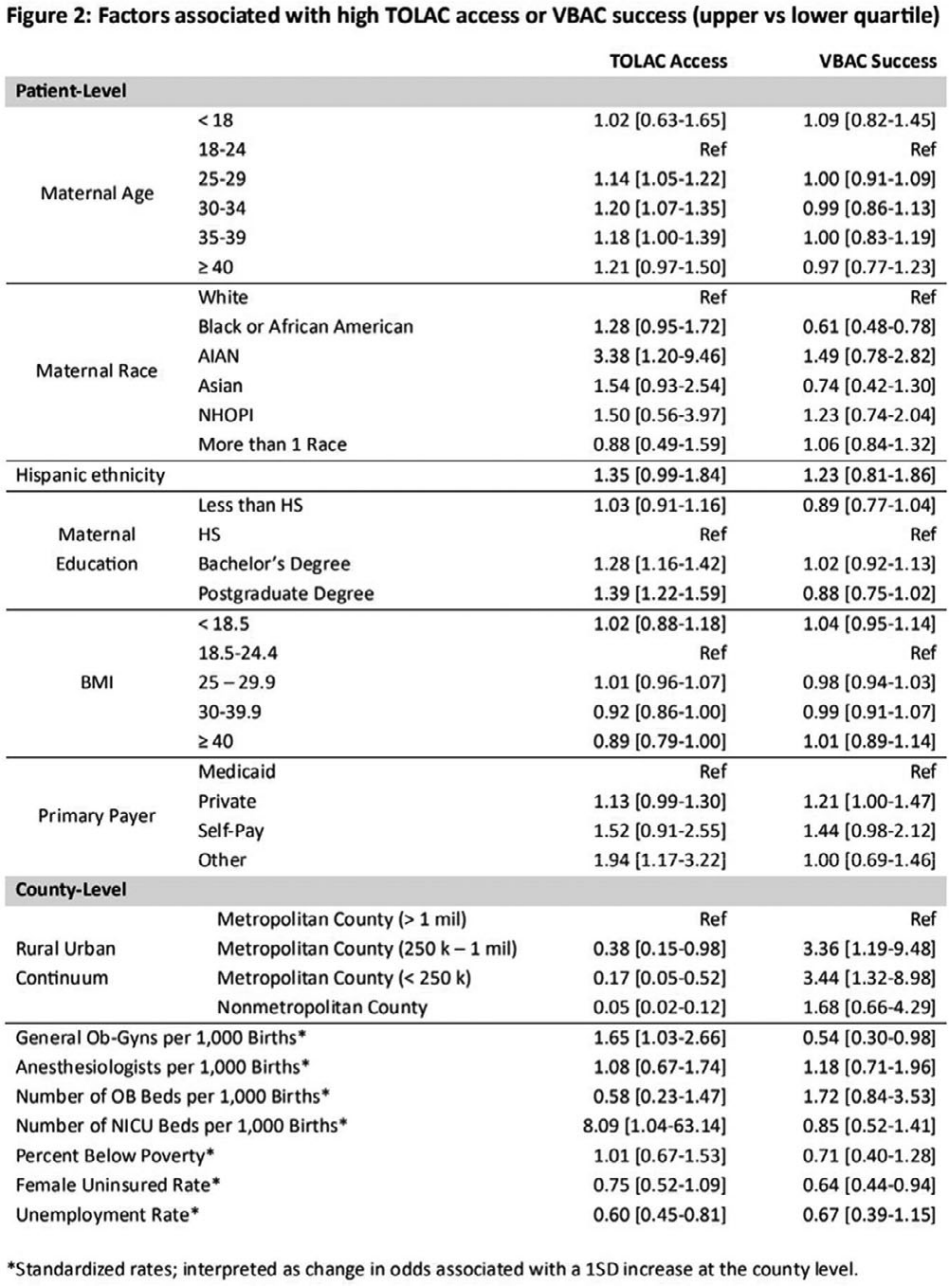

**10:30 AM – 12:00 PM**

## 376 | Hypertensive Disorders of Pregnancy and Long‐Term Cognitive Change

Sunitha Suresh^1^; Joseph Larson^2^; Erika Hripko^3^; Jerome J. Federspiel^4^; Angela Malek^5^; Stephen Rapp^6^; Laura Baker^6^; Beverly Snively^6^

^1^Endeavor Health, Endeavor Health System, IL; ^2^Fred Hutchinson Cancer Center, Fred Hutchinson Cancer Center, WA; ^3^Loyola University, Chicago, IL; ^4^Duke University School of Medicine, Durham, NC; ^5^Medical University of South Carolina, Charleston, SC; ^6^Wake Forest University School of Medicine, Winston‐Salem, NC

**Objective**: A relationship between HDP and subsequent cognitive impairment has been shown in small studies but not confirmed in large cohort studies. The purpose of this study was to utilize the Women's Health Initiative (WHI) to investigate the correlation between HDP and long‐term cognitive change.

**Study Design**: This is a secondary analysis of the WHI and three cognitive ancillary studies (WHI Memory Study, WHI Epidemiology of Cognitive Health Outcomes, WHI SleeP Hypoxia Effects on Resilience Study). Participants with self‐reported HDP and any live birth were included. Outcomes studied were (1) self‐reported cognitive change (Cognitive Change Index 12‐item short form; CCI) (2) Telephone Interview of Cognitive Status modified (TICSm), a validated measure of global cognitive function, and (3) a composite of clinician adjudicated probable dementia or mild cognitive impairment (PD/MCI). For CCI and TICSm indices, linear regression was utilized to examine differences by reported history of HDP. For PD/MCI, the time to onset was analyzed using Cox proportional hazards models. Both linear and Cox models were adjusted for demographics, lifestyle characteristics and medical conditions.

**Results**: A total of 37,238 participants were included in the CCI analysis, 4202 in the TICSm analyses and 1089 in the PD/MCI analyses (Figure). CCI scores were worse among those with self‐reported HDP history (Adjusted Risk Difference 0.43 (95% CI 0.10, 0.76, *p* = 0.01, Table). There was no difference in TICSm scores by HDP (Adjusted Risk Difference 0.01, 95% CI −0.46, 0.48, *p* = 0.97) or onset of PD/MCI (Adjusted Hazard Ratio 0.80, 95% CI 0.58, 1.09), *p* = 0.15).

**Conclusion**: There was an association between HDP and worsened perceived long‐term cognitive change. However, there was no difference in objective cognitive function as measured by a validated scale and clinician adjudicated PD/MCI. Further investigation is needed in understanding who may be at risk for cognitive change after a pregnancy with HDP.

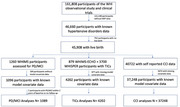

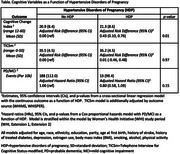

**10:30 AM – 12:00 PM**

## 377 | Peripartum Morbidity in Patients With BMI ≥ 50: Does Planned Cesarean Reduce Risk?

Sweet Hope Mapatano^1^; Sarah Heerboth^2^; Annie Dude^3^; Anna Montessi^1^; Tracy A. Manuck^4^

^1^University of North Carolina ‐ Chapel Hill, Chapel Hill, NC; ^2^University of North Carolina ‐ Chapel Hill, UNC Chapel Hill, Chapel Hill, NC; ^3^University of North Carolina, Chapel Hill, NC; ^4^Division of Maternal Fetal Medicine, Department of Obstetrics, Gynecology and Reproductive Science, University of North Carolina, Chapel Hill, NC

**Objective**: The optimal mode of delivery for patients with BMI ≥ 50kg/m2 remains unknown; some providers opt for planned primary cesarean delivery (CD) despite limited evidence supporting this approach. We sought to determine whether a planned CD is associated with reduced maternal and/or neonatal morbidity compared to planned vaginal delivery (VD) among patients with BMI ≥ 50 kg/m2.

**Study Design**: Secondary analysis of the MFMU APEX cohort. We included patients with pre‐pregnancy BMI ≥50kg/m2 and singleton, vertex pregnancies at ≥ 37 weeks. The primary outcome = composite maternal morbidity (≥1 of: PPH, blood transfusion, pulmonary edema, VTE, ICU admission, infection, wound complications). Secondary outcomes = composite non‐infectious maternal morbidity and neonatal ICU admission. Outcomes were compared, by planned mode of delivery (intent to treat), with sensitivity analysis by actual mode of delivery.

**Results**: 1208 of 118,422 APEX participants met inclusion criteria; 265 (22%) had a planned CD; 363 (38.5%) planning VD actually delivered by cesarean. Those with planned CD were more likely to have a history of prior cesarean, diabetes, and private insurance and were less likely to have a hypertensive disorder of pregnancy. (Table 1). The primary outcome of composite maternal morbidity occurred in 144 (11.9%). Planned mode of delivery was not associated with composite maternal morbidity or non‐infectious composite morbidity in adjusted models. However, actual delivery by cesarean was associated with an increased odds of composite maternal morbidity compared to actual VD (aOR 1.6, 95% CI 1.1–2.3). NICU admission did not differ by planned or actual delivery mode (Figure 1).

**Conclusion**: Among individuals with BMI ≥ 50 kg/m^2^, planned CD was not associated with reduced maternal or neonatal morbidity. Actual CD was linked to higher maternal morbidity, suggesting that unplanned cesareans may carry greater risk. Improved identification of patients likely to labor successfully may help reduce morbidity in this high‐risk group.

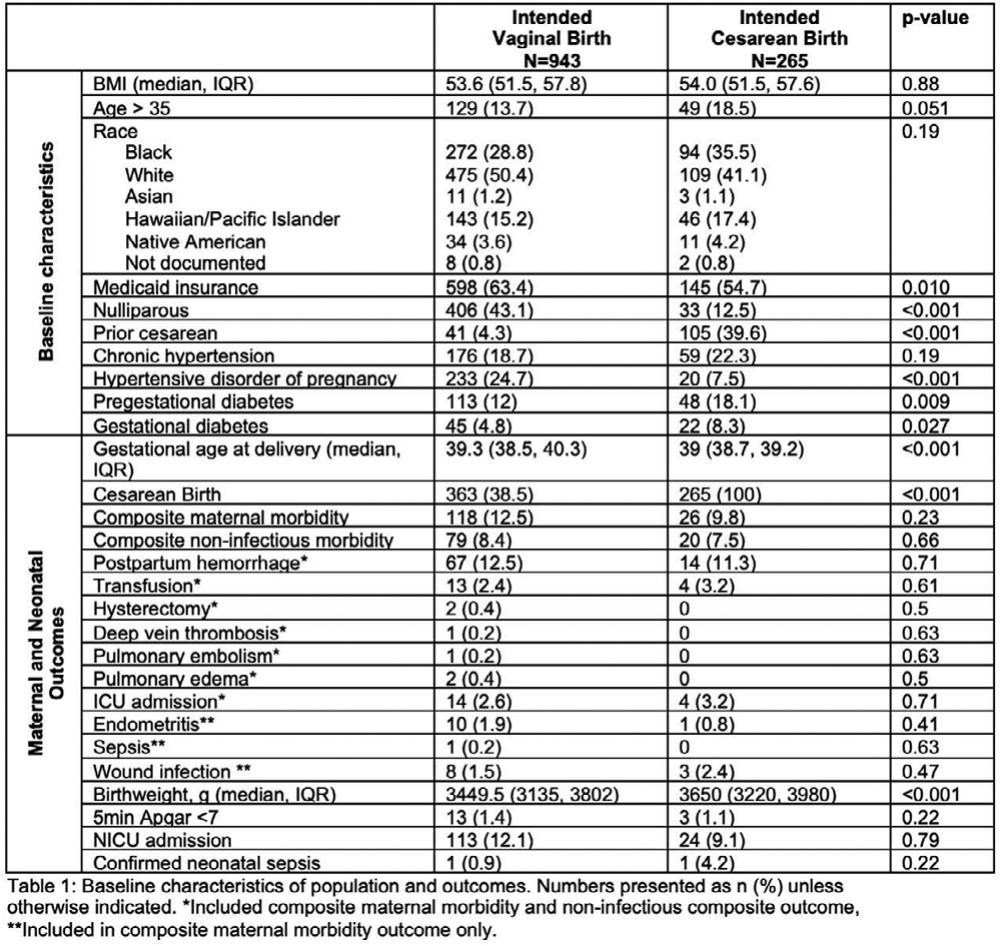

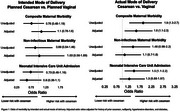

**10:30 AM – 12:00 PM**

## 378 | Factors Contributing to Breast Cancer Diagnoses During Delivery Hospitalizations: A Decomposition Analysis

Sydney Williams^1^; Yongmei Huang^2^; Logan Tom^3^; Timothy Wen^4^; Alexander M. Friedman^1^

^1^Columbia University, New York, NY; ^2^Division of Maternal‐Fetal Medicine, Department of Obstetrics and Gynecology, Columbia University Irving Medical Center, New York, NY; ^3^Northeastern University, Boston, MA; ^4^UCSD, Irvine, CA

**Objective**: This study aimed to evaluate trends in breast cancer risk among pregnant women and examine factors contributing to changes in breast cancer (BC) risk over time.

**Study Design**: We conducted a serial cross sectional study using data from the Nationwide Inpatient Sample. Women aged 15–54 years who gave birth from 2006–2022 without a history of BC were included. The primary outcome was BC during the delivery hospitalization, identified using ICD9 and 10 diagnosis codes. Joint Point regression was used to evaluate temporal trends in BC, and logistic regression models were employed to identify factors associated with BC risks. Multivariate logistic regression decomposition was applied to quantify the contribution of predictors to the difference in breast cancer risk between 2006–2008 and 2020–2022.

**Results**: A total of 13 million births were identified. BC risk increased from 6.5 per 100,000 births in 2006 to 12.3 in 2022, with an average annual percentage change of 5.1% (95% CI: 3.9–6.4, *p* < 0.0001) (Figure). Demographic and clinical characteristics of delivery hospitalizations with and without breast cancer are shown in the Table. Older maternal age, chronic hypertension, family history of breast cancer, and genetic susceptibility were positively associated with BC risk during pregnancy. From 2006 to 2022, there were significant increases in the proportion of women with older maternal age and more chronic conditions. Multivariate logistic regression decomposition analysis indicated that the difference in BC risk between 2020–2022 and 2006–2008 were attributable to changes in population characteristics (55.9%) and changes in coefficients (44.1%). Equalizing the age distribution in 2020–2022 to match that in 2006–2008 would be expected to reduce BC risk in 2020–2022 by 57%. Chronic conditions contributed little to BC risk.

**Conclusion**: Breast cancer risk among pregnancy population has increased over time, with advanced maternal age accounting for more than half of the increase in breast cancer occurrence.

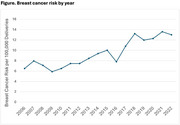

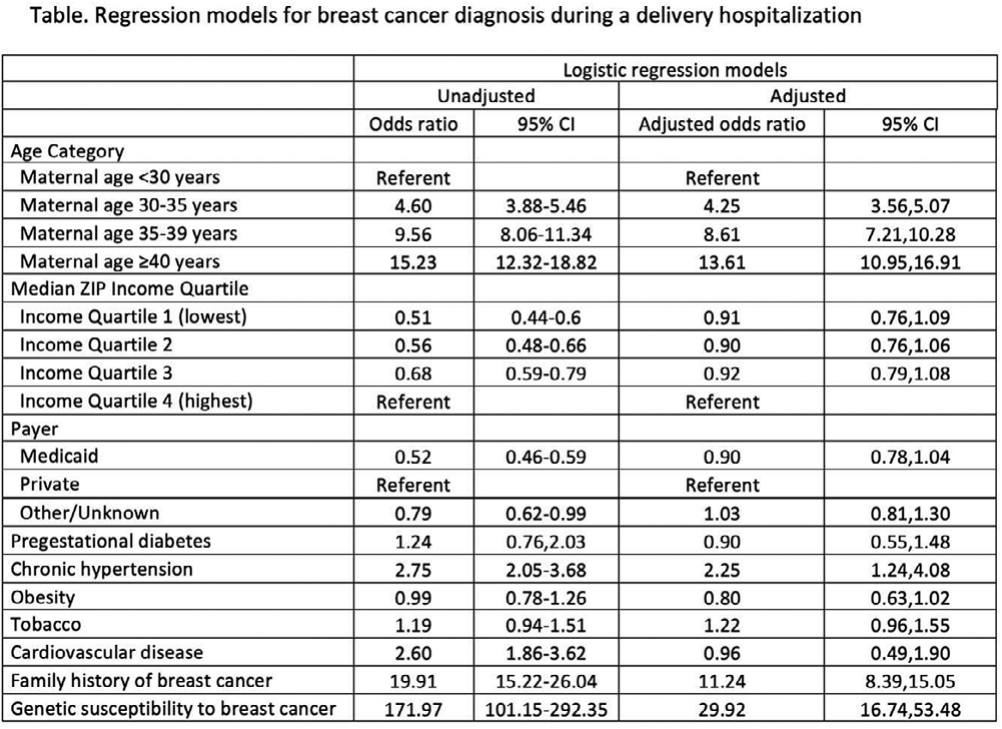

**10:30 AM – 12:00 PM**

## 379 | Effect of Misoprostol Reclassification as a Schedule IV Drug on Postpartum Hemorrhage Management

Talia Suner^1^; Elizabeth Howard^2^; Meena Mishra^3^; Charles Sisk^4^; Harsheen Chawla^4^; James D. Toppin^1^; Joseph R. Biggio, Jr.^5^; Jane K. Martin^6^; Frank B. Williams^6^

^1^Ochsner Clinic Foundation, New Orleans, LA; ^2^Ochsner Medical Center, New Orleans, LA; ^3^Ochsner Center of Outcomes & Health Services Research, New Orleans, LA; ^4^Ochsner Clinic Foundation, New Olreans, LA; ^5^Ochsner Health, New Orleans, LA; ^6^Ochsner Clinic Foundation, Ochsner Clinic Foundation, LA

**Objective**: On October 1st, 2024, Louisiana Act 246 went into effect, re‐classifying misoprostol and mifepristone as Schedule IV controlled substances. Misoprostol is frequently used for management of postpartum hemorrhage. We investigated how this reclassification affects management of postpartum hemorrhage (PPH).

**Study Design**: We performed a retrospective cohort study of all deliveries in a large regional health system from July 1, 2024, to January 1, 2025, comparing deliveries prior to and after enactment of Act 246. Patients who experienced a PPH (quantitative blood loss [QBL] >1000cc) were included. The primary outcome was use of misoprostol for management of PPH. Secondary outcomes included use of other uterotonic medications, QBL, intrauterine vacuum or tamponade device usage, severe maternal morbidity and mortality (SMM), transfusion, ICU admission, and change in hemoglobin/hematocrit. Chi‐Square test was used to determine relative risk with 95% confidence intervals.

**Results**: There were 748 deliveries complicated by PPH during the study period, 380 occurred prior to and 368 deliveries occurred after Act 246 enactment. There was no difference in misoprostol use for management of postpartum hemorrhage before and after Act 246 enactment (RR 1.02, 0.88–1.18). There was no difference in the use of methylergometrine (RR 0.93, 0.80–1.07), carboprost (RR 1.01, 0.85–1.20), PPH devices (RR 1.07, 0.82–1.40), SMM (RR 0.86, 0.57–1.29), transfusion (RR 1.03, 0.85–1.26), ICU admission (RR 0.91, 0.51–1.65), or total interventions required (*p* = 0.104). Administration of tranexamic acid increased after Act 246 enactment (RR 1.19, 1.03–1.38).

**Conclusion**: After reclassification of misoprostol as a schedule IV drug, use of tranexamic acid for management of postpartum hemorrhage increased. The use of misoprostol, other uterotonic medications, and PPH devices was unchanged. This highlights the importance of developing processes to ensure ready access to medications in the event that restrictive laws become enacted.

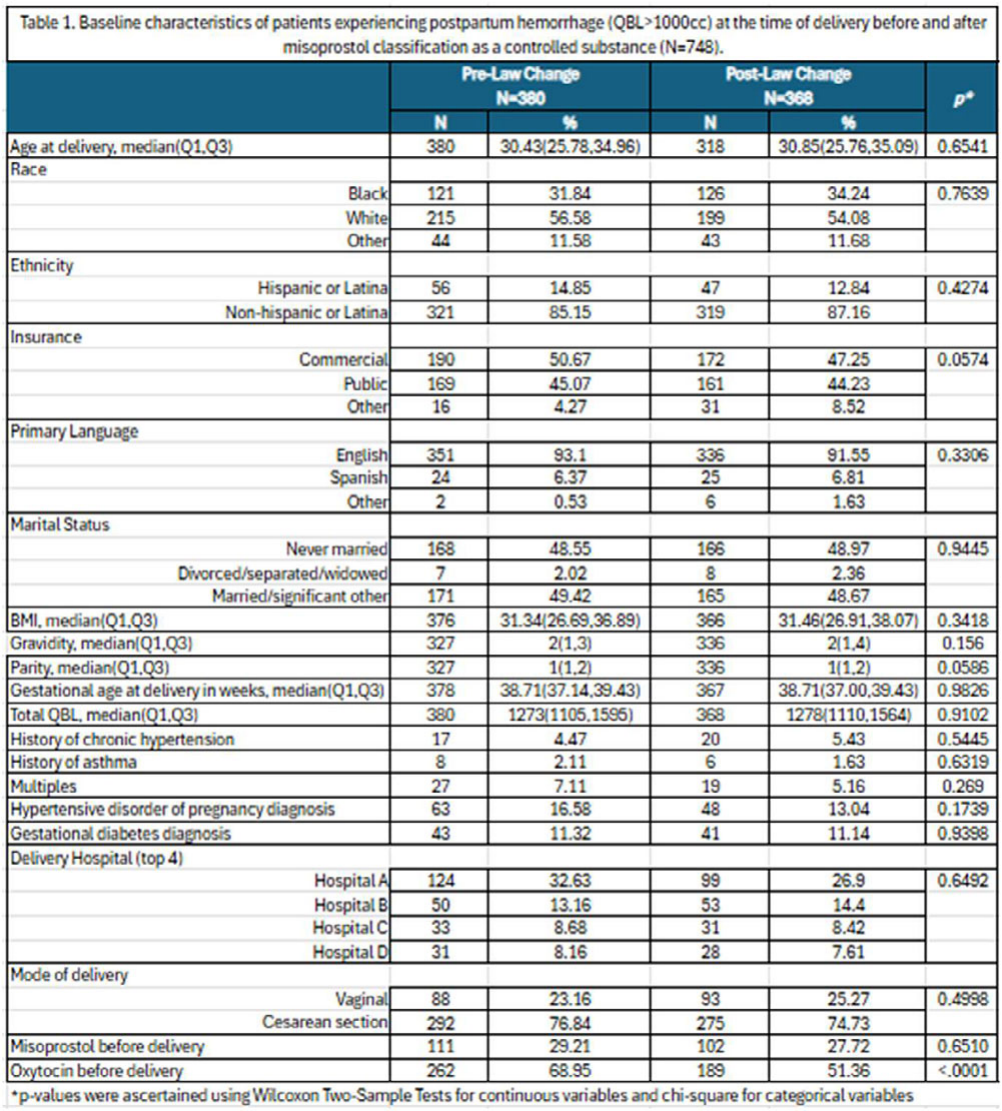

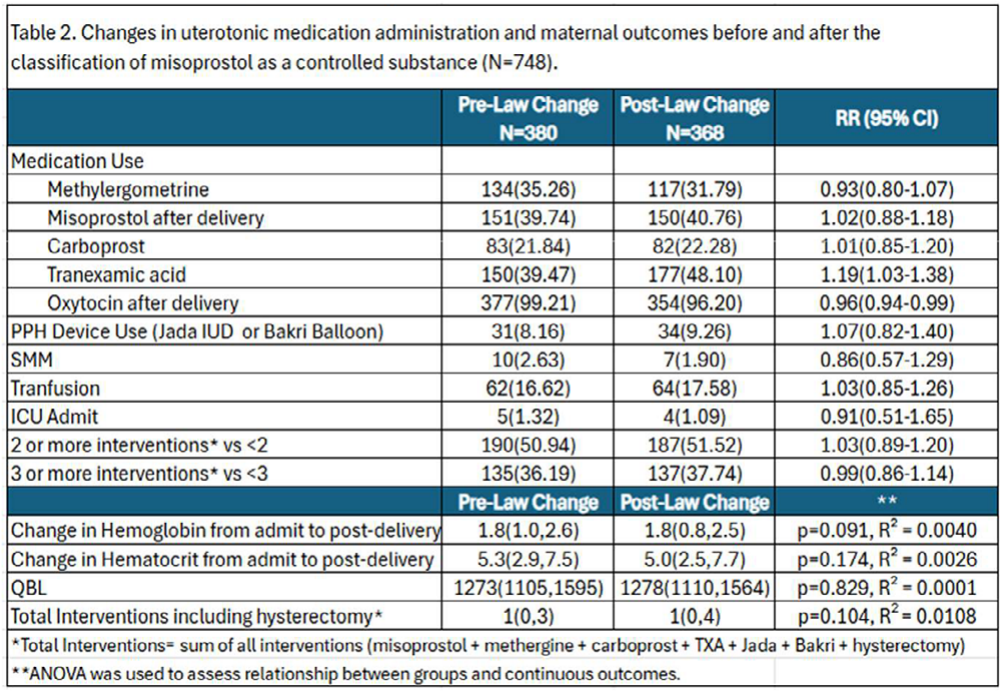

**10:30 AM – 12:00 PM**

## 380 | Breathtaking Consequences: Prenatal Armed Conflict Exposure and Offspring Respiratory Morbidity

Tamar Wainstock^1^; Polina Schwarzman^2^; Eyal Sheiner^3^

^1^Department of Epidemiology, Biostatistics and Community Health Sciences, Faculty of Health Sciences, Ben‐Gurion University of the Negev, Beer Sheva, HaDarom; ^2^Soroka University Medical Center, Soroka University Medical Center, HaDarom; ^3^Soroka University Medical Center, Faculty of Health Sciences, Ben‐Gurion University of the Negev, beer sheva, HaDarom

**Objective**: Exposure to maternal stress during pregnancy has been associated with adverse outcomes in offspring. However, little is known about the long‐term health consequences of in utero exposure to war‐related conflict. We hypothesize that in utero exposure to maternal stress induced by armed conflict, particularly during first trimester, disrupts fetal pulmonary development, thereby increasing the risk of long‐term respiratory morbidity, including asthma, in childhood.This study aimed to evaluate whether prenatal exposure to war is associated with increased risk for respiratory morbidities and complications in childhood.

**Study Design**: A population‐based study was conducted, using birth and health records from a single large medical center and the largest HMO in the country. The exposed group included children whose mothers were pregnant during periods of war conflict. We assessed diagnoses of respiratory illnesses and complications during childhood as the primary outcomes. Multivariable Cox regression models were used to estimate associations, adjusting for season of birth, maternal age, preterm delivery, and pregnancy complications. A subgroup analyses focusing on exposure limited to the first trimester was also conducted.

**Results**: Of the 130656 offspring in the study, 22481 (17.2%) were exposed in‐utero to war, and 8922 (6.8%) were exposed specifically during first trimester (Tabe). Offspring exposed in utero to war had a higher risk of respiratory morbidities (42.6% versus 34.7%, OR = 1.40; 1.34–1.46), and specifically more likely to be diagnosed with asthma (40.3% versus 32.3%, OR 1.41, 95% CI 1.37–1.45). The risk for respiratory diseases was higher, regardless of the trimester of exposure, gestational age at delivery, gestational diabetes, maternal age and ethnicity (aHR = 1.08; 1.04–1.12).

**Conclusion**: Prenatal exposure to war conflict was associated with increased risk of respiratory morbidity in offspring, persisting into childhood. These findings highlight the need for long‐term follow‐up on offspring exposed to conflict environments during early life, and targeted support for pregnant individuals.

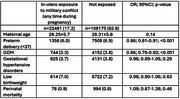

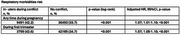

**10:30 AM – 12:00 PM**

## 381 | The Interaction of Elective Induction and Estimated Weight on Cesarean Delivery

Taylor Freret^1^; Mark A. Clapp^2^; Chloe Zera^1^; Blair J. Wylie^1^; Sarah E. Little^3^

^1^Beth Israel Deaconess Medical Center, Boston, MA; ^2^Department of Obstetrics and Gynecology, Mass General Brigham, Massachusetts General Hospital, MA; ^3^Beth Israel Deaconess Medical Center, Newton, MA

**Objective**: The optimal management of presumed macrosomia is unknown. The Lancet 2025 “Big Baby” trial found a lower risk of cesarean delivery with induction vs. expectant management. However, it is unknown whether this is meaningfully different than the expected reduction at any birthweight. This study evaluated whether the association between delivery approach (induction vs expectant management) and cesarean risk is modified by birthweight.

**Study Design**: Secondary analysis of the ARRIVE trial including nulliparas with singleton, cephalic fetuses randomized to induction or expectant management at 39 weeks. The primary exposure of interest was induction vs expectant management and estimated fetal weight at 39 weeks (approximated from 39‐week birthweight or if >39 weeks, applying the sex‐specific birthweight percentile to obtain a 39‐week estimate). Estimated fetal weight was analyzed continuously and in quartiles. The primary outcome was cesarean birth. Multivariable logistic regression was performed to analyze the interaction between estimated weight and management strategy on cesarean birth.

**Results**: Of 6074 deliveries, the risk of cesarean was significantly higher with increasing estimated fetal weight at 39 weeks: 16.1% in quartile 1 (2353–3053 grams), 17.8% Q2 (2065–3323 g), 19.7% Q3 (3323–3579 g) and 38.0% Q4 (3579–4649 g) (*p* < 0.01). The odds of cesarean birth with labor induction vs. expectant management varied significantly by estimated weight quartile: Q1 1.02 (0.78–1.34), Q2 0.89 (0.69–1.16), Q3 0.73 (0.57–0.94) and Q4 0.69 (0.55–0.87). There was a significant interaction (*p* < 0.01) between management strategy and estimated weight. Figure 1 demonstrates that induction was not associated with a significant reduction in cesarean birth until the estimated weight at 39 weeks was >3245 grams.

**Conclusion**: The “ARRIVE effect” of cesarean birth reduction with induction vs. expectant management is only seen with an estimated weight at 39 weeks >3245 grams. The “dose‐dependent” nature of the effect suggests that if resources are limited it may be optimal to target those with a larger estimated fetal weight.

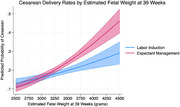

**10:30 AM – 12:00 PM**

## 382 | Genetic Evaluation and Diagnostic Findings in Pregnant Patients With Renal Disease

Taylor Moseley; Likhita Nandigam; Ansley Davis; Christian M. Parobek; Sai Kaumudi Saridey; Hendrik A. Lombaard

Baylor College of Medicine, Houston, TX

**Objective**: Renal disease in pregnancy is often attributed to lifestyle factors and other chronic diseases. However, genetic factors are estimated to contribute to as much as 40% of adult renal disease cases.^1^ We assessed the frequency of renal genetic testing in pregnant patients with chronic kidney disease (CKD) and end‐stage renal disease (ESRD) and explored the genetic diagnoses identified.

**Study Design**: This is an IRB‐approved retrospective cohort study of pregnant patients with CKD and ESRD who received prenatal care at Texas Children's Hospital in Houston, Texas, from 2011–2024. Subjects were identified by an ICD10‐based search. Demographic, clinical, and genetic data were abstracted from the electronic health record. The primary outcomes were the proportion of patients who received renal genetic evaluation and workup. Bivariate analyses were used to identify associations between demographic or clinical factors and genetic outcomes. For identified genetic diagnoses, a qualitative content analysis was performed.

**Results**: Of the 103 patients included, 93 had CKD and 10 had ESRD. Only 11 (11%) underwent a renal genetic workup before, during, or after pregnancy. Seven patients had pathogenic or likely pathogenic variants resulting in a genetic diagnosis that explained their renal disease. These occurred in seven genes and encompassed five diseases including Alport syndrome, polycystic kidney disease, nephronophthisis, Fanconi renotubular syndrome with monogenic diabetes, and congenital nephrotic syndrome. In most cases, diagnoses were made by renal panel or exome sequencing, though one was diagnosed via expanded carrier screening.

**Conclusion**: For pregnant patients with renal disease, renal genetic evaluation is uncommon despite a high burden of genetic diagnoses. Overall, the spread of these genetic diseases reflects the diversity of CKD genetic causes. These findings suggest that a comprehensive renal genetic evaluation in pregnancies affected by renal disease is underutilized, and also highlight the diagnostic potential of expanded carrier screening for pregnant patients with renal disease.

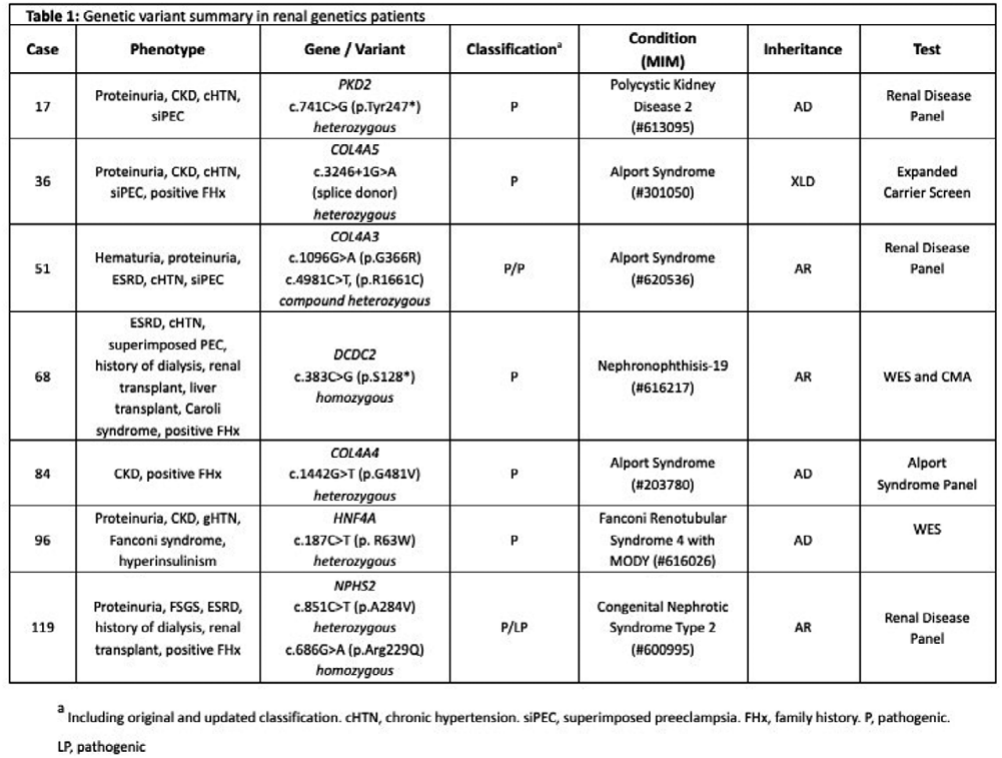

**10:30 AM – 12:00 PM**

## 383 | To Optimize Antenatal Management of PreTerm Labor With Amniocentesis‐Based Prediction Models: Pragmatic Randomized Clinical Trial

Teresa Cobo^1^; David Boada^2^; Ignacio Herraiz^3^; Cecilia Villalain^3^; Silvia Ferrero^4^; María Goya^5^; Laia Martí‐Malgosa^6^; Mª Àngels Vives^7^; mª Paz Carrillo^8^; Fernando Bugatto^9^; Carmen Garrido^10^; Carmen Medina^10^; Alicia Martínez^11^; Vicente Diago^11^; Laia Pratcorona^12^; Cristina Paulés^13^; Francisco López^14^; Marta Jané^15^; Mªdel Mar Gil^16^; Victoria Aldecoa^17^; Francesc Figueras^18^; Montse Palacio *^2^; Eduard Gratacós^18^

^1^Hospital Clinic Barcelona, Hospital Clinic (Barcelona), Catalonia; ^2^Hospital Clínic Barcelona, BARCELONA, Catalonia; ^3^Hospital 12 Octubre, Hospital 12 Octubre (Madrid), Madrid; ^4^Hospital Sant Joan de Déu, Hospital Sant Joan de Déu (Barcelona), Catalonia; ^5^Hospital Vall d'Hebrón, Hospital Vall d'Hebrón (Barcelona), Catalonia; ^6^Hospital Consorci Corporació Sanitària Parc Taulí, Hospital Consorci Corporació Sanitària Parc Taulí (Sabadell), Catalonia; ^7^Consorci Sanitari Terrassa, Consorci Sanitari Terrassa (Terrassa), Catalonia; ^8^Hospital Universitario Virgen de las Nieves, Hospital Universitario Virgen de las Nieves (Granada), Andalucia; ^9^Hospital Puerta del Mar, Hospital Puerta del Mar (Cádiz), Andalucia; ^10^Hospital de la Santa Creu i Sant Pau, Hospital de la Santa Creu i Sant Pau (Barcelona), Catalonia; ^11^Hospital La Fe, Hospital La Fe (Valencia), Comunidad Valenciana; ^12^Hospital Germans trias i Pujol, Hospital Germans trias i Pujol (Badalona), Catalonia; ^13^Hospital Clinico Universitario Lozano Blesa, Hospital Clinico Universitario Lozano Blesa (Zaragoza), Aragon; ^14^Hospital La Paz, Hospital La Paz (Madrid), Madrid; ^15^Hospital Joan 23, Hospital Joan 23 (Tarragona), Catalonia; ^16^Hospital Universitario de Torrejón, Hospital Universitario de Torrejón (Torrejón de Ardoz), Madrid; ^17^Hospital Clínic Barcelona, Hospital Clinic (Barcelona), Catalonia; ^18^Hospital Clinic, Hospital Clinic (Barcelona), Catalonia

**Objective**: Most of patients with preterm labor (PTL) don't deliver preterm. However, they are routinely managed with corticosteroids, trading off the benefit of fetal maturation for deleterious neurodevelopmental, cardiovascular and metabolic effects in fetuses finally delivered near term. We aimed to evaluate whether amniocentesis‐based management reduces the antenatal course of corticosteroids and maternal hospital stay without jeopardizing neonatal or maternal outcomes.

**Study Design**: Pragmatic randomized multicenter clinical trial in singleton pregnancies with PTL between 23 + 0–34 + 6 weeks, not in arrested labor, without clinical chorioamnionitis.

The **standard‐management** arm was managed according to institutional protocols, including a course of steroids, tocolysis, and magnesium sulfate.

The **amniocentesis‐based management** arm consisted of the use of predictor models: if low‐risk, the course of steroids was not completed, discontinuing the rest of the medication and promoting hospital discharge within 24 h. If high‐risk, antibiotics were added to the standard management.

The primary outcome was courses of corticosteroids and maternal hospital stay.

**Results**: From 2021–2024, 261 were randomized (134 in the standard and 127 in the amniocentesis‐based management arm). Maternal characteristics didn't differ between groups.

In the standard‐management arm, 96.3% of patients were treated with a course of steroids, compared to 44.9% in the amniocentesis‐based management arm. The difference (95% confidence interval (CI)) was 51.4% (40.3–61.3). Maternal stay length was significantly reduced in the amniocentesis‐based management arm (median (p25; p75)) (4 (3; 6) days vs. 2 (2; 4) days).

We did not find differences at gestational age at delivery (38.1 (34.9; 39.6) *vs*. 37.3 (34.6; 39.0) weeks), latency‐to‐delivery (7.4 (3.4; 10.4) *vs*. 6.3 (3.3; 10) weeks), spontaneous delivery within 7 days (13.4% *vs*. 11.2%) and neonatal or maternal morbidity between groups.

**Conclusion**: An amniocentesis‐based management allows optimizing antenatal steroids administration and maternal stay without worsening perinatal outcome.

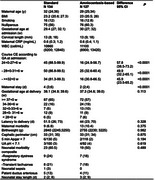

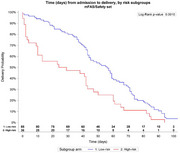

**10:30 AM – 12:00 PM**

## 384 | Risk Factors for Postpartum Readmissions due to Surgical Site Infections After Cesarean Delivery

Tetsuya Kawakita^1^; Misa Hayasaka^2^; Grace Spencer^3^

^1^Eastern Virginia Medical School Department of Obstetrics and Gynecology, Macon & Joan Brock Virginia Health Sciences at Old Dominion University, Norfolk, VA; ^2^Macon & Joan Brock Virginia Health Sciences at Old Dominion University Eastern Virginia Medical School, Norfolk, VA; ^3^Eastern Virginia Medical School at Old Dominion University, EVMS OBGYN, Virginia Health Sciences at Old Dominion University, VA

**Objective**: To identify risk factors and timing of postpartum readmissions due to surgical site infections (SSI) after cesarean delivery.

**Study Design**: This was a retrospective cohort study using the Healthcare Cost and Utilization Project (HCUP) National Readmissions Database from 2018 to 2022. We included all patients undergoing cesarean delivery. The primary outcome was readmission within 42 days postpartum for SSI, defined as wound infection, wound disruption, uterine infection, or sepsis. We used modified Poisson regression to estimate adjusted relative risks (aRR) with 95% confidence intervals (CI) for risk factors including demographics, comorbidities, cesarean indications, and neighborhood income (zip code quartiles). The timing of readmissions was plotted by days from discharge.

**Results**: Among 2,563,282 patients, 16,650 (0.7%) were readmitted for SSI. Adjusted risks are presented in Table 1. The strongest risk factors were placenta accreta (aRR 2.17; 95% CI 1.81–2.60), pregestational diabetes (aRR 1.80; 95% CI 1.68–1.92), government insurance (aRR 1.58; 95% CI 1.52–1.63), arrest of active phase labor (aRR 1.52; 95% CI 1.44–1.60), obesity (aRR 1.47; 95% CI 1.42–1.52), and preterm delivery (aRR 1.44; 95% CI 1.38–1.50). Protective factors included higher income quartile (Q2 aRR 0.92, 95% CI 0.88–0.96; Q3 aRR 0.88, 95% CI 0.85–0.92; Q4 aRR 0.79, 95% CI 0.75–0.83, compared to Q1), prior cesarean (aRR 0.80; 95% CI 0.77–0.83), and placenta previa (aRR 0.86; 95% CI 0.76–0.97). Median time to readmission was 9 days post‐discharge (Figure 1).

**Conclusion**: SSI‐related postpartum readmissions after cesarean delivery are infrequent but influenced by both clinical and social determinants of health. Most readmissions occur within the first two weeks, indicating a narrow window for intervention. Early outpatient follow‐up, tailored discharge instructions, and targeted support for high‐risk individuals—particularly those with medical comorbidities or lower socioeconomic status—may reduce the burden of readmissions and improve maternal recovery.

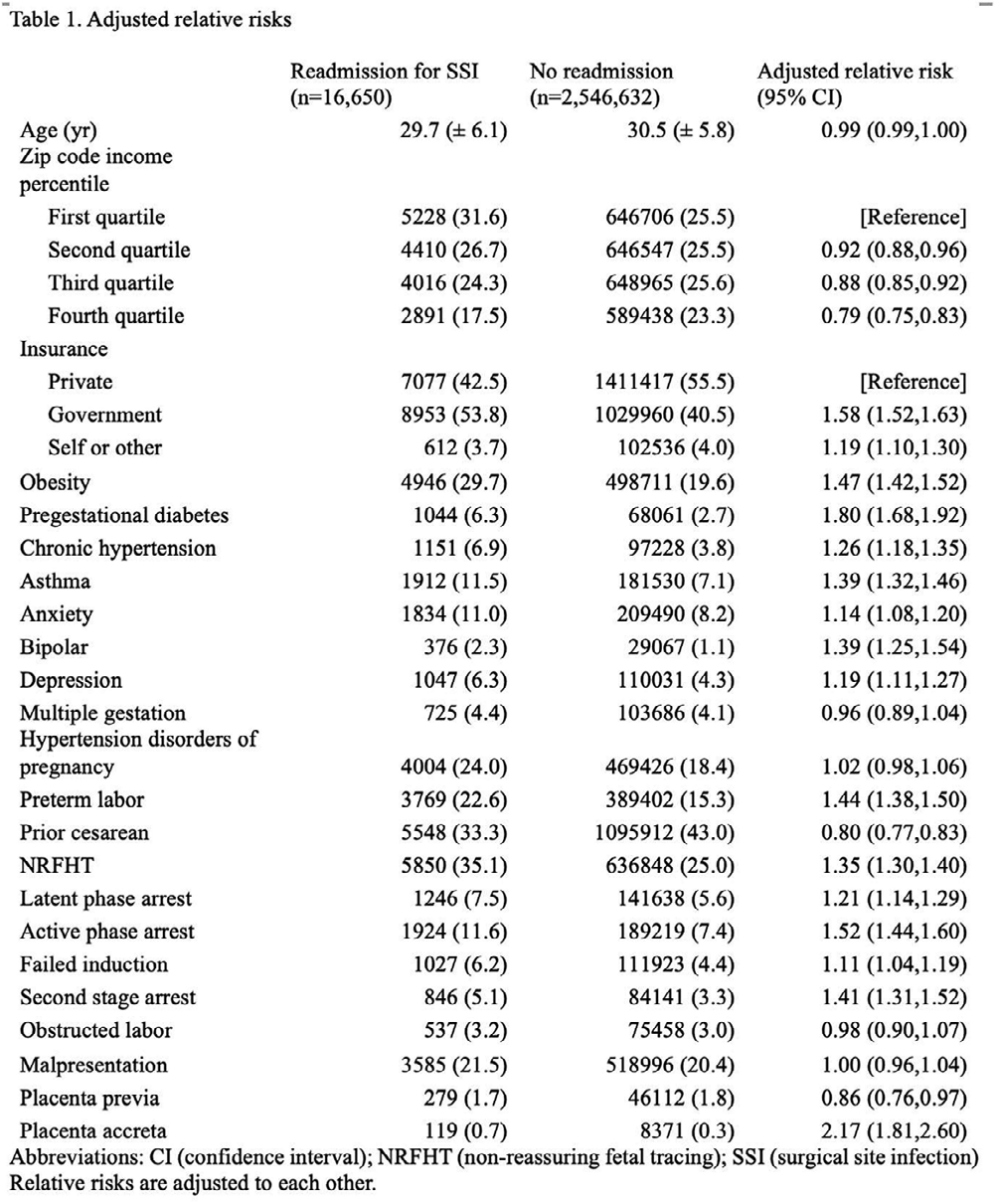

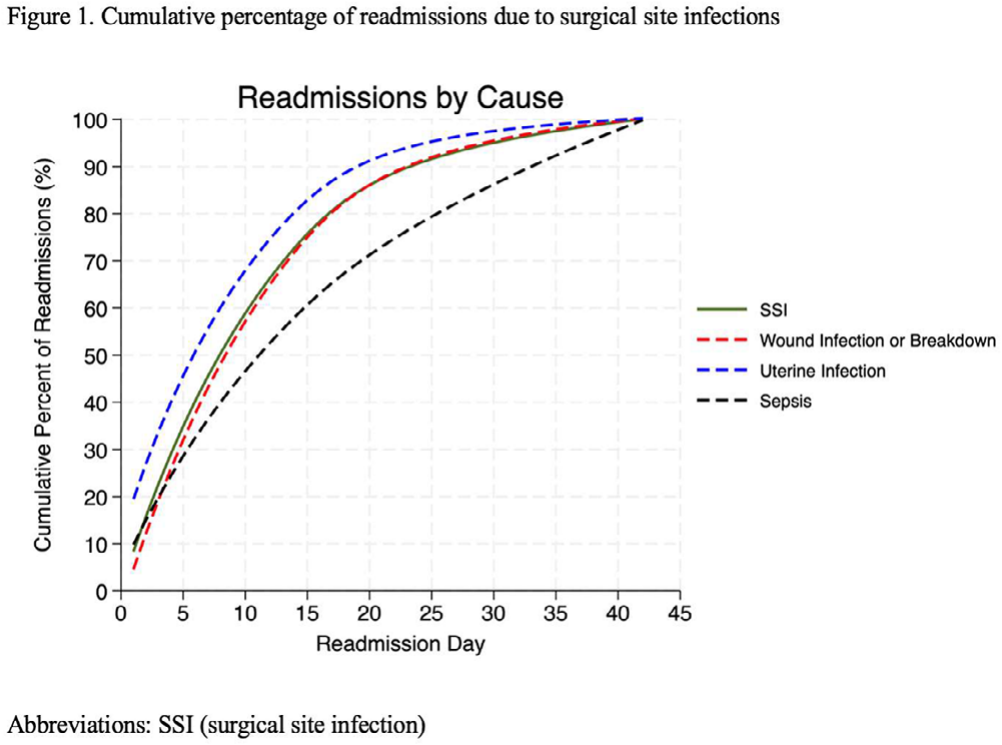

**10:30 AM – 12:00 PM**

## 385 | Prenatally‐Diagnosed Cleft Lip and Palate: Genetic Findings and Sequencing Yield in Isolated Versus Non‐Isolated Cases

Thomas P. Kishkovich^1^; Gabriela F. Tessler^2^; Meghan Angley^1^; Thomas A. Imahiyerobo^3^; Sherelle L. Laifer‐Narin^4^; Hong Ma^4^; Lynn L. Simpson^5^; Caitlin D. Baptiste^1^; Jessica L. Giordano^1^; Russell S. Miller^1^; Ronald J. Wapner^1^

^1^Division of Maternal‐Fetal Medicine, Department of Obstetrics and Gynecology, Columbia University Irving Medical Center, New York, NY; ^2^Columbia University Medical Center, New York, NY; ^3^Division of Plastic Surgery, Columbia University Irving Medical Center, New York, NY; ^4^Department of Radiology, Columbia University Irving Medical Center, New York, NY; ^5^Division of Maternal‐Fetal Medicine, Department of Obstetrics and Gynecology, Columbia University Irving Medical Center, new york, NY

**Objective**: Evaluate prevalence of genetic conditions in prenatally diagnosed isolated and non‐isolated fetal cleft lip and/or palate (CLP).

**Study Design**: Retrospective cohort study of prenatally diagnosed CLP at a single referral center between 2017–24. Inclusion required prenatal or postnatal genetic testing via karyotype, chromosomal microarray (CMA), and/or exome/genome sequencing. Prenatal imaging and genetic findings compared between isolated CLP (iCLP) and CLP with additional anomalies (CLP+). T‐tests, Wilcoxon rank‐sum, Chi‐Square, or Fisher's Exact tests used as appropriate.

**Results**: 58 pregnancies met inclusion criteria: 20 (34.5%) iCLP and 38 (65.5%) CLP+. Unilateral CLP was present in 55% of iCLP and 36.8% of CLP+ cases. There were no demographic differences between groups. Among CLP+, the most common additional prenatally detected anomalies were central nervous system (55.3%) and cardiac (52.6%). Of available postnatal evaluations, 2/15 (15.4%) of iCLP and 9/22 (40.9%) of CLP+ cases (*p* = 0.14) had additional structural anomalies identified after delivery. (Table 1)

Across the entire cohort, 21 (36.2%) pregnancies had reported findings from genetic testing, with 16 (76.2%) possessing findings likely causative of CLP. CLP+ pregnancies were more likely to have causative findings (36.8% vs 10.0%, *p* = 0.03). 11 of 16 (68.8%) likely causative findings were detected via karyotype/CMA. Among 29 patients with sequencing, 5 (17.2%) had likely causative variants not detected by karyotype and CMA. One pregnancy had dual findings, three had incidental actionable findings via sequencing, and two had uncertain results via CMA. (Table 2)

**Conclusion**: While CLP+ was associated with higher rates of genetic diagnoses, all CLP was associated with increased risk of both cytogenetic and genomic genetic diagnoses. In cases with non‐causative karyotype or CMA, sequencing revealed a causative genomic variant in approximately 1 in 6 cases. Sequencing diagnosed unsuspected, but clinically relevant findings in 3 additional cases. Evaluation with sequencing is therefore recommended for all pregnancies complicated with CLP.

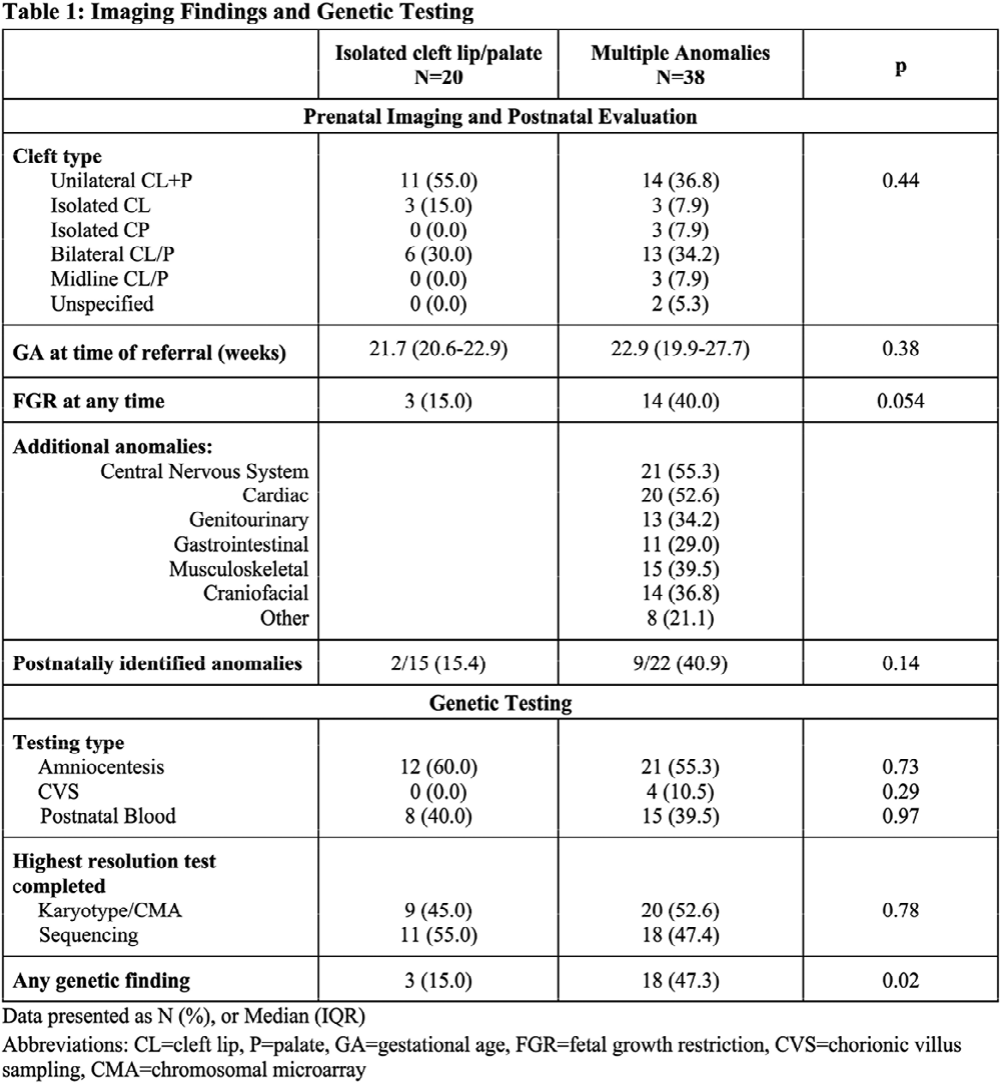

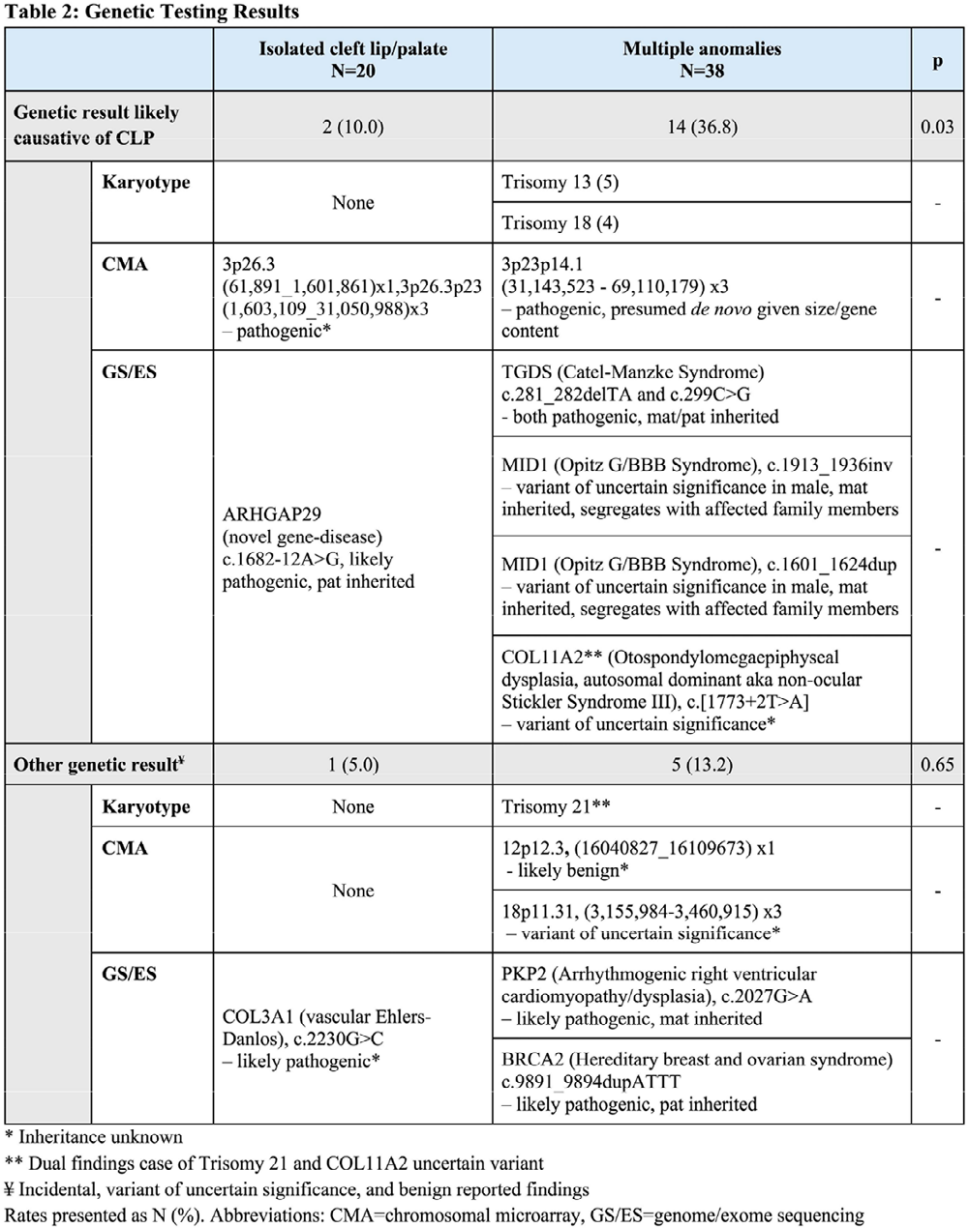

**10:30 AM – 12:00 PM**

## 386 | Highly Reliable Data Collection by a Large Language Model in Fetal Ultrasound Reports

Thomas A. Reynolds^1^; Sierra D. Land^1^; Jake Ligouri^1^; Ryan Treftz^1^; N Scott Adzick^1^; Holly L. Hedrick^1^; Juliana S. Gebb^2^

^1^Richard D. Wood, Jr Center for Fetal Diagnosis and Treatment, Children's Hospital of Philadelphia, Children's Hospital of Philadelphia, PA; ^2^Richard D. Wood Jr Center for Fetal Diagnosis and Treatment, Children's Hospital of Philadelphia, Children's Hospital of Philadelphia, PA

**Objective**: To develop a reliable and efficient data collection process for unstructured fetal ultrasound reports involving single‐ and higher‐order gestations. At our institution, documentation of middle cerebral artery (MCA) Doppler often includes more than one value and range per fetus, contributing to variability in report structure and complexity. We evaluated the performance of an off‐the‐shelf Large Language Model (LLM) integrated into our Clinical Outcomes Data Archive (CODA) platform, aiming to reduce human effort while maintaining high data fidelity.

**Study Design**: A subset of fetal ultrasound reports was processed using an LLM in CODA. For each fetus, we abstracted whether an MCA Doppler was performed and extracted the peak systolic velocity (PSV), and up to two multiple‐of‐the‐median (MoM) values when a range was reported. All outputs were reviewed by a human validator. Discrepancies were recorded, and agreement between the LLM and final validated values were calculated for each variable.

**Results**: Of 494 fetal ultrasound reports processed using an LLM in CODA, the LLM correctly identified 349 reports that had at least one MCA Doppler study. These reports included 630 fetus‐level evaluations, yielding 1249 datapoints for analysis. Among 244 studies ultimately deemed not performed, the LLM incorrectly labeled 10 as performed, though it accurately marked all three study values as missing in these cases. All three target values for the MCA Doppler parameters performed reached >99% accuracy.

**Conclusion**: An off‐the‐shelf LLM integrated into the CODA platform demonstrated high reliability in abstracting structured data from unstructured fetal ultrasound reports. The model achieved >99% accuracy for all extracted MCA Doppler values and maintained perfect performance in marking missing data. These findings support the feasibility of LLM‐assisted abstraction to reduce manual workload while preserving data quality in complex clinical documentation, including higher‐order gestations.

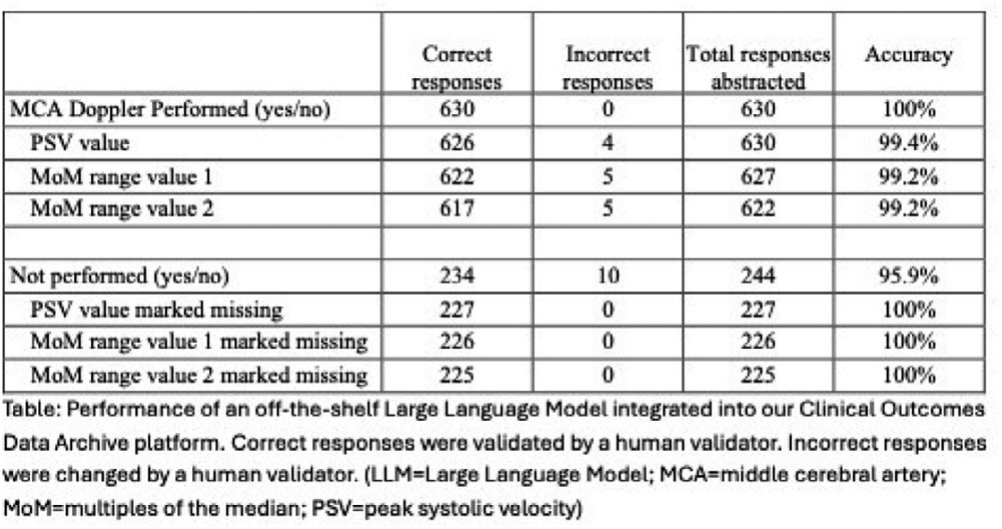

**10:30 AM – 12:00 PM**

## 387 | The Association Between Allostatic Load and Gestational Diabetes ‐Related Pregnancy Outcomes

Tinuola Oladebo^1^; Nadine Sunji^1^; Alyssa M. Hernandez^1^; Liyun Zhang^1^; Amy Y. Pan^1^; Lynn M. Yee^2^; George R. Saade^3^; David M. Haas^4^; Shannon Barnes^5^; Janet Catov^6^; Jessica Pippen^7^; Philip Greenland^8^; Uma M. Reddy^9^; Judith H. Chung^10^; Lisa D. Levine^11^; Lauren H. Theilen^12^; C. Noel Bairey Merz^13^; Jasmina Varagic^14^; Victoria L. Pemberton^14^; Nathan Vandergrift^15^; Anna Palatnik^16^

^1^Medical College of Wisconsin, Medical College of Wisconsin, WI; ^2^Northwestern University Feinberg School of Medicine, Chicago, IL; ^3^Eastern Virginia Medical School at Old Dominion University, Norfolk, VA; ^4^Indiana University School of Medicine, Indianapolis, IN; ^5^Research triangle park, Research Triangle Park, NC; ^6^University of Pittsburgh, Pittsburgh, PA; ^7^MetroHealth Medical Center, Cleveland, OH; ^8^Northwestern University, Chicago, IL; ^9^Columbia University Irving Medical Center, New York, NY; ^10^University of California Irvine Medical Center, Irvine, CA; ^11^Perelman School of Medicine, University of Pennsylvania, Philadelphia, PA; ^12^University of Utah, Salt Lake City, UT; ^13^Cedars‐Sinai Medical Center, Los Angeles, CA; ^14^National Heart, Lung, and Blood Institute, NIH, Bethesda, MD; ^15^RTI, Research triangle park, NC; ^16^Medical College of Wisconsin, MILWAUKEE, WI

**Objective**: To examine the association of allostatic load with gestational diabetes (GDM) and GDM‐related pregnancy outcomes.

**Study Design**: This was a secondary analysis of the Nulliparous Pregnancy Outcomes Study: Monitoring Mothers‐to‐be Heart Health Study, which followed nulliparas from early pregnancy to 2–7 years postpartum. The primary exposure of this analysis was high allostatic load (AL) in the first trimester, defined as having four or more of 12 biomarkers (systolic blood pressure, diastolic blood pressure, body mass index, cholesterol, low‐density lipoprotein, high‐density lipoprotein, high sensitivity C‐reactive protein, triglycerides, insulin, glucose, creatinine, and albumin) being in the highest‐risk quartile. The primary outcome was the frequency of GDM in the entire cohort. Secondary outcomes were pregnancy complications among those who developed GDM. Univariable and multivariable logistic regression models were used to evaluate association between AL and adverse pregnancy outcomes.

**Results**: A total of 1405 (38%) women had high AL. In unadjusted models, high AL was associated with increased odds of GDM (OR 2.88, 95% CI: 2.08–3.98, Table 1). After adjusting for maternal age at first prenatal visit, pre‐pregnancy BMI, education, marital status, poverty, physical activity, chronic hypertension, and polycystic ovarian syndrome, high AL remained to be significantly associated with GDM (aOR 1.66, 95% CI 1.09–2.53). Secondary analyses within the GDM subgroup (*n* = 164) demonstrated higher odds of large for gestational age (OR 3.22, 95% CI: 1.16–8.98) and preeclampsia (OR 2.87, 95% CI: 1.02–8.06) (Table 2). After controlling for first trimester BMI, presence of chronic hypertension, GDM type (A1 vs. A2), marital status, and insurance type, these associations did not persist.

**Conclusion**: High allostatic load was independently associated with GDM diagnosis. Within the GDM group, no significant association was found between high AL and incidence of pregnancy complications related to GDM after adjusting for potential confounders.

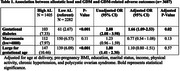

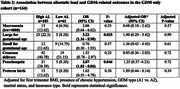

**10:30 AM – 12:00 PM**

## 388 | Quality Improvement Initiative to Reduce Wound Complications From Cesarean Delivery in Class III Obesity

Tucker E. Doiron^1^; Laurie Niewoehner^2^; Niraj R. Chavan^1^

^1^Saint Louis University, St. Louis, MO; ^2^SSM Health St. Mary's Hospital, St. Louis, MO

**Objective**: This study was undertaken to evaluate the impact of a quality improvement (QI) initiative on perioperative wound related complications after cesarean delivery (CD) in patients with a BMI ≥ 40kg/m^2^.

**Study Design**: We evaluated QI process measures and perioperative wound‐related clinical outcomes across 2 groups–patients with a BMI ≥ 40kg/m^2^ undergoing CD before (10/2023–9/2024) and after (10/2024–6/2025) implementation of a QI initiative centered on prophylactic administration of oral cephalexin and metronidazole in the 48‐hour postoperative period. Primary clinical outcomes included: rates of surgical site infection, triage visit for wound‐related problems, wound separation, readmission to the hospital, and need for antibiotic treatment. Process measures evaluated were appropriate antibiotic choice, adherence to recommended regimen, receipt of full course of antibiotics, and reasons for protocol deviations. Continuous and categorical data were compared using Student's t test and chi squared or Fisher's exact test, respectively.

**Results**: Of the 1334 CDs over the study period, 344 (25.7%) had a BMI ≥ 40kg/m^2^, of which 275 (20.6%) met inclusion criteria ‐ including 168 in the pre and 107 in the post‐implementation group. The overall BMI was 49.36 ± 7.93 kg/m2 Pertinent pregnancy complications, type of CD, and rates of preoperative antibiotic use were similar in both groups, with a significantly higher rate of repeat CDs in the post group. (50.60% vs 64.49%, *p* = 0.02). Clinical outcomes, including–wound infection [14(8.33%) vs 1(0.93%), *p* = 0.01], triage visits for wound complications [16(9.52%) vs 2(1.87%), *p* = 0.01] and need for antibiotic treatment [13(7.74%) vs 1(0.93%), *p* < 0.01] were significantly lower in the post‐ compared to pre‐implementation group. Of the 107 patients in the post group, 90 (84.11%) had an order for antibiotics, 74 (82.22%) had the appropriate order, and 63 (85.14%) received the full 48‐hour course.

**Conclusion**: Prophylactic postoperative antibiotics have the potential to significantly decrease wound related complications among patients with class III obesity undergoing CD.

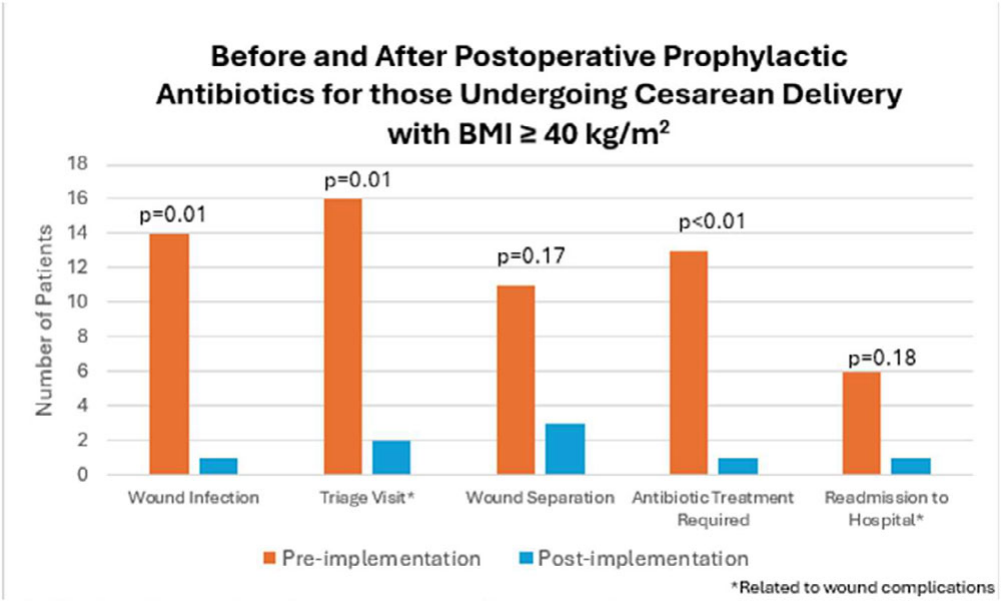

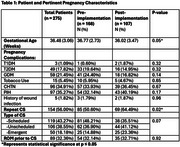

**10:30 AM – 12:00 PM**

## 389 | Additive Bayesian Network Analysis of Social Determinants of Health and Adverse Pregnancy Outcomes

Ummayhany Bharmal^1^; Grace Spencer^2^; Tetsuya Kawakita^3^

^1^Medical College Baroda, Vadodara, Gujarat; ^2^Eastern Virginia Medical School at Old Dominion University, EVMS OBGYN, Virginia Health Sciences at Old Dominion University, VA; ^3^Eastern Virginia Medical School Department of Obstetrics and Gynecology, Macon & Joan Brock Virginia Health Sciences at Old Dominion University, Norfolk, VA

**Objective**: The relationship between Social Determinants of Health (SDoH) and Adverse Pregnancy Outcomes (APO) has been well established, yet these factors interact in complex and non‐linear ways. Our objective was to use Additive Bayesian Networks to examine interrelationships between SDoH variables and APO.

**Study Design**: This was a secondary analysis of data from the Nulliparous Pregnancy Outcomes Study: Mothers‐to‐Be (nuMoM2b). Individuals with missing data or delivery <23 weeks were excluded. The primary outcome was a composite of APOs, including preterm births <37 weeks, hypertensive disorders of pregnancy, small for gestational age (<5th percentile), stillbirth, and gestational diabetes mellitus. We examined direct and indirect associations between APOs and SDoH as well as clinical factors, including body mass index (BMI), pregestational diabetes mellitus (PDM), and chronic hypertension (CHTN), using additive Bayesian network analysis. A final Directed Acyclic Graph (DAG) was derived from 1000 resamples created using non‐parametric bootstrapping. Results are reported as odds ratios (OR) along with their 95% credibility intervals (CrI).

**Results**: Of 7089, 1686 (23.7%) had APOs. The optimal model allowed a maximum of six parent nodes and initially identified 44 arcs; 40 were retained after bootstrapping in the final DAG (Figure 1). APOs were directly associated with BMI [logOR: 1.33; 95%CrI:1.26,1.41], race [logOR: 1.39; 95%CrI: 1.17, 1.64], and PDM [logOR: 3.29; 95%CrI: 2.08, 5.23]. Race was influenced by emotional support, health literacy, employment, perceived stress, anxiety, and social support. BMI mediated the effects of race, smoking history, and anxiety, and its effects on APO were also partly mediated by PDM. Associations between various variables are presented in Table 1.

**Conclusion**: A complex interplay of social, behavioral, and psychosocial factors contributes to the development of adverse outcomes. Integration of social and clinical interrelationships into risk models and intervention is necessary to predict adverse pregnancy outcomes.

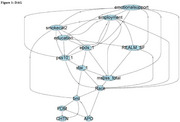

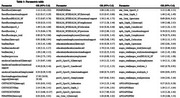

**10:30 AM – 12:00 PM**

## 390 | AI‐Derived Umbilical Cord Inflammatory Index for Identification of Fetal Inflammatory Response Syndrome

Un Yung Choi^1^; Young Min Kim^2^; Haeryoung Kim^3^; Ji Hoi Kim^1^; Eun Na Kim^3^; Youngbin Ahn^2^; Tae Kwan Lee^4^; Sun Min Kim^5^; Byoung Jae Kim^5^; Jaehee Jeong^4^; So Hee Kim^1^; Chan‐Wook Park^4^; Joong Shin Park^4^; Young‐Gon Kim^2^; Seung Mi Lee^6^

^1^Seoul National University Hospital, Seoul, Seoul‐t'ukpyolsi; ^2^Department of Transdisciplinary Medicine, Seoul National University Hospital, Seoul, Republic of Korea, Seoul, Seoul‐t'ukpyolsi; ^3^Department of Pathology, Seoul National University Hospital, Seoul National University College of Medicine, Seoul, Republic of Korea, Seoul, Seoul‐t'ukpyolsi; ^4^Department of Obstetrics and Gynecology, Seoul National University Hospital, Seoul National University College of Medicine, Seoul, Republic of Korea, Seoul, Seoul‐t'ukpyolsi; ^5^Seoul National University College of Medicine, Seoul, Seoul‐t'ukpyolsi; ^6^Department of Obstetrics and Gynecology, Seoul National University College of Medicine, Seoul, Seoul‐t'ukpyolsi

**Objective**: Artificial intelligence (AI)‐based imaging can enhance histopathologic diagnosis, but its clinical significance has not been well determined. The purpose of the current study is to compare the performance of an AI‐generated inflammation index of umbilical cord with traditional pathologic criteria (funisitis) for identifying fetal inflammatory response syndrome (FIRS).

**Study Design**: We developed an attention‐based multiple instance learning (MIL) model to quantify umbilical cord inflammation from whole‐slide images. The model was trained with patches from umbilical cord slides to localize inflammatory regions and output patch‐wise inflammation scores. The proportion of inflamed areas was calculated by binarizing patch scores using a defined threshold. The performance of AI‐generated inflammation index and the grade of funisitis were compared for identification of FIRS, which was defined as high neonatal blood CRP.

**Results**: We analyzed a cohort comprising 386 cases who were delivered before 34 weeks of gestation. The FIRS was defined as neonatal CRP >0.4385, which was defined as the optimal cutoff for early neonatal sepsis in the current study population. The AUROC of AI‐based inflammatory index was higher than that of funisitis grade for identification of FIRS (0.71 vs 0.67). Cases with high AI‐based inflammatory index (>75percentile) had lower gestational age at delivery and high frequency of histologic chorioamnionitis. Among the study population, 103 women underwent amniocentesis within 1 month of delivery. Women with AI‐based inflammatory index were more likely to have intraamniotic infection/inflammation (27.6% vs 88.2%, *p* < 0.001), and high AF WBC counts (median, 2.00 ± 10.625 vs 1700 ± 2006.25, *p* < 0.001)

**Conclusion**: The AI‐derived inflammatory index from umbilical cord demonstrated improved discriminative ability over standard pathologic evaluation (funisitis grade) in identifying FIRS and were related to intraamniotic infection/inflammation, suggesting its potential as a more objective and quantitative tool for perinatal inflammation assessment.

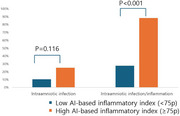

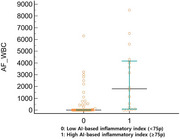

**10:30 AM – 12:00 PM**

## 391 | The Association Between sFlt‐1/PlGF Ratio and Unplanned Cesarean Delivery in Individuals With Suspected Placental Insufficiency

Uri Shemesh^1^; Keren Zloto^2^; Tal Dadon^3^; Rakeft Yoeli Ullman^4^; Abraham Tsur^4^; Shalom Mazaki‐Tovi^5^; yoav yinon^6^; Michal Fishel Bartal^7^; Noa Gonen^8^

^1^The Department of Obstetrics and Gynecology, The Chaim Sheba Medical Center, Ramat‐Gan, Israel, Tel Aviv, Tel Aviv; ^2^sheba medical center, Tel Hashomer, Tel Aviv; ^3^Sheba medical hospital, Tel Aviv, HaMerkaz; ^4^The Sheba Medical Center, The Sheba Medical Center, HaMerkaz; ^5^The Department of Obstetrics and Gynecology, The Chaim Sheba Medical Center, Ramat‐Gan, Israel, Ramat Gan, HaMerkaz; ^6^Sheba Medical Center, Ramat Gan, Israel, Ramat Gan, HaMerkaz; ^7^Division of Maternal‐Fetal Medicine, Department of Obstetrics, Gynecology and Reproductive Sciences, Sheba Medical Center, Tel Aviv University, Israel, Ramat Gan, HaMerkaz; ^8^Wolfson Medical Center, Holon, HaMerkaz

**Objective**: To determine the predictive value of an abnormal sFlt‐1/PlGF ratio for unplanned cesarean delivery (CD) in laboring individuals with suspected placental insufficiency.

**Study Design**: This retrospective cohort study included all laboring individuals ( ≥23 weeks of gestation) with suspected placental insufficiency at a tertiary care center between August 2023 and July 2025. Suspected placental insufficiency included those small for gestational age fetus (SGA) (EFW <10^th^ percentile), hypertensive disorders of pregnancy (HDP), or oligohydramnios (AFI <5 cm). An sFlt‐1/PlGF ratio was obtained at presentation, however, the result was not used to guide clinical management. Those undergoing elective cesarean delivery were excluded. The cohort was stratified by sFlt‐1/PlGF ratio (<38 vs. ≥ 38), and rates of unplanned cesarean delivery among laboring individuals were compared. Multivariate logistic regression was used to assess the independent variables associated with sFlt‐1/PlGF ratio and unplanned CD.

**Results**: Among the 818 patients, 231 (28.2%) had an elevated sFlt‐1/PlGF ratio (≥ 38). Patients with abnormal ratios were more likely to be nulliparous, older, conceived via fertility treatment, and had a higher incidence of HDP and fetal growth restriction (Table 1). The rate of unplanned CD was higher in the abnormal sFlt‐1/PlGF ratio compared to those with a normal value (41.1 vs 22.5%). Following multivariate analysis sFlt‐1/PlGF ratio did not remain a significant independent predictor of unplanned cesarean delivery (adjusted OR, 95% CI, Table 2).

**Conclusion**: In laboring individuals with suspected placental insufficiency, an elevated sFlt‐1/PlGF ratio is not an independent predictor for increased risk of unplanned cesarean delivery.

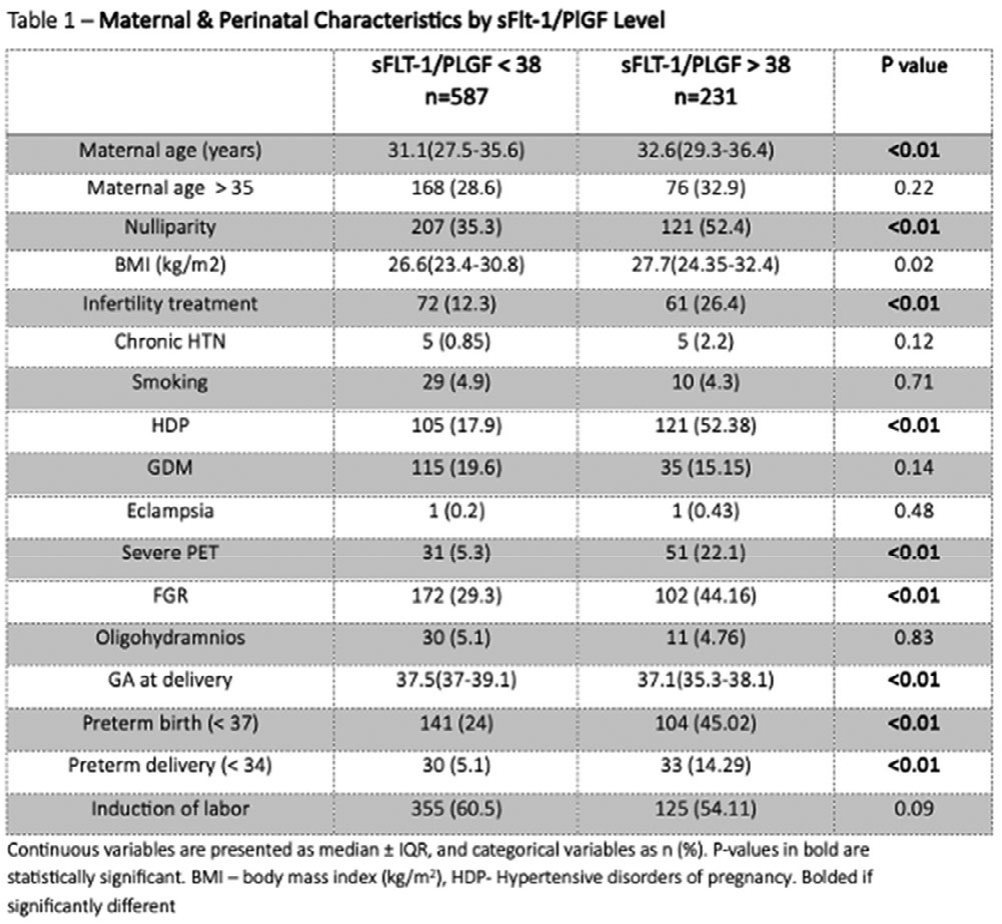

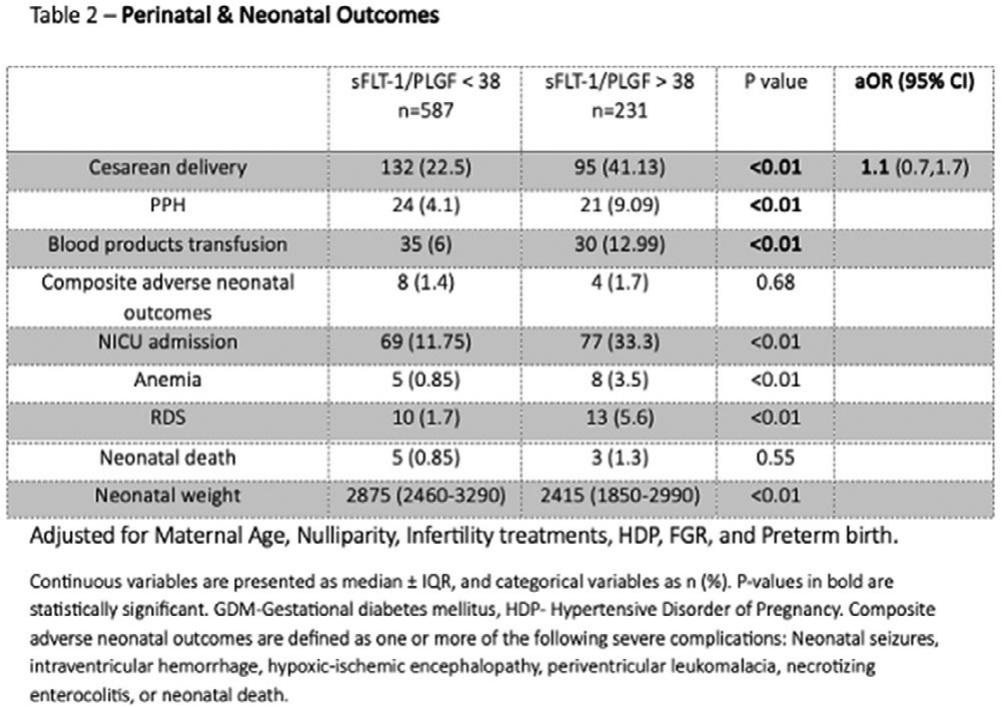

**10:30 AM – 12:00 PM**

## 392 | Risk of Maternal Cardiovascular Events During Childbirth in Twins and Triplets Versus Singletons

Virali Patel^1^; Jessica Gleason^2^; Shan‐Xuan Lim^1^; Zhen Chen^1^; Dian He^3^; Jagteshwar Grewal^1^; Katherine L. Grantz^1^

^1^Eunice Kennedy Shriver National Institute of Child Health and Human Development, National Institutes of Health, Bethesda, MD; ^2^NICHD, NIH, Bethesda, MD; ^3^The Prospective Group, Inc., Fairfax, VA

**Objective**: Physiologic changes in the maternal cardiovascular system associated with pregnancy are more pronounced in multiple gestations compared to singletons. However, it is unknown whether these changes lead to increased morbidity during childbirth. We compared the risk of cardiovascular events (CVE) during childbirth in multiple versus singleton gestations and evaluated if the observed association was independent of hypertensive disorders of pregnancy (HDP).

**Study Design**: In a secondary analysis of women in the Consortium on Safe Labor, maternal demographics, number of fetuses, HDP diagnosis, gestational age, and CVE during childbirth were collected from medical records and ICD9 codes. Multiple imputation addressed missing covariate data. Relative risks (RR, 95% CI) of CVE for twin and triplet compared to singleton pregnancies were calculated using modified Poisson regression with robust standard error adjusted for maternal age, pre‐pregnancy body mass index, race/ethnicity, parity, insurance status, marital status, site, chronic disease, and stratified by HDP status.

**Results**: Of the 191,948 deliveries, 2.2% were twin and 0.1% were triplet deliveries. CVE occurred in 0.4% of singletons, 1.0% of twins, and 2.0% of triplets (*p* < 0.001, Table). There was an elevated risk of CVE in twin and triplet gestations compared to singletons [Twins: 2.79 (2.05–3.81); Triplets: 4.90 (1.59–15.07), Figure]. Twin gestations remained at an elevated risk of CVE [2.35 (1.72–3.21)] after accounting for baseline differences and in subgroup analyses by HDP status [Twins vs Singletons: HDP: 2.75 (1.73–4.37); Without HDP: 1.69 (1.08–2.63), Figure]. Notably, triplet gestations had higher risk of CVE among women without HDP than those with HDP [Triplets vs Singletons: HDP: 3.17 (0.80–12.60); Without HDP: 2.21 (0.28–17.48), Figure].

**Conclusion**: Multiple gestation was an independent risk factor for CVE during childbirth. Given that CVE is a major risk factor for severe maternal morbidity, our findings highlight the importance of risk stratification and heightened awareness in patients with multigestation pregnancies.

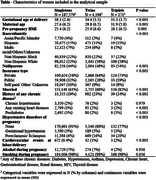

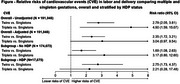

**10:30 AM – 12:00 PM**

## 393 | Predictors of Hypertensive Disorders of Pregnancy Among Patients With Inflammatory Bowel Disease

Weirui Xiao^1^; Johanna Bedoy^1^; Mehrnaz Siavoshi^2^; Lorna Kwan^2^; Thalia Mok^1^; Nirupama Bonthala^1^; Ilina Pluym^1^

^1^David Geffen School of Medicine at University of California, Los Angeles (UCLA), Los Angeles, CA; ^2^Department of Urology, David Geffen School of Medicine at University of California, Los Angeles (UCLA), Los Angeles, CA

**Objective**: To investigate predictors of hypertensive disorders of pregnancy (HDP) in patients with inflammatory bowel disease (IBD) and evaluate the use of low‐dose aspirin (LDA) prophylaxis.

**Study Design**: This was a retrospective cohort study of all singleton pregnancies with a history of IBD who delivered at a single academic center from January 2019 to December 2023. Primary outcome was development of HDP, including gestational hypertension or preeclampsia. Baseline characteristics, LDA use, pregnancy outcomes, and IBD disease activity measured by remission status or flares during the pregnancy were collected. The Kruskal‐Wallis test or Chi‐squared test of independence was performed. Multivariate logistic regression was used to identify independent predictors of HDP.

**Results**: Of 100 deliveries among 89 patients with IBD, 22 (22%) were complicated by HDP. Baseline characteristics, including maternal age (36.3 years vs 33.9 years, *p* = 0.06 and obesity (31.8% vs 21.8%, *p* = 0.33), were not significantly different between groups. HDP was more likely to occur among patients who had a history of HDP (22.7% vs 2.6%, *p* < 0.01) or concurrent gestational diabetes (3.8% vs 18.2%, *p* = 0.02) (Table 1). Fetal fraction on cell‐free fetal DNA (cfDNA) was lower in pregnancies with HDP (8.0% vs 10.3%, *p* = 0.01). HDP did not differ by clinical remission rates (86.4% vs 85.9%, *p* = 0.86) or IBD flares during pregnancy (22.7% vs 15.4%, *p* = 0.42). In multivariate analysis, prior HDP remained a significant predictor (aOR 6.98, 95% CI 1.24–39.8; Table 2). LDA for preeclampsia prevention was reported in 16% (*n* = 16) and was similar in patients who did and did not develop HDP (22.7% vs 14.1%, *p* = 0.33)

**Conclusion**: HDP occurred in over one in five pregnancies among patients with IBD and did not appear to be related to disease activity. Despite elevated risk, LDA prophylaxis appeared underutilized in this cohort. Further studies are needed to determine the risk‐benefit ratio of LDA use in pregnancies with IBD.

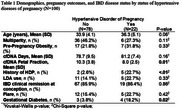

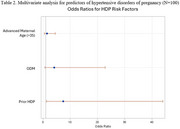

**10:30 AM – 12:00 PM**

## 394 | The Incidence of Uterine Windows With Higher Order Cesareans: Should We Be Delivering Them Earlier?

Xiteng Yan^1^; Joyce Tumfour^1^; Andrei Rebarber^2^; Samuel Bender^1^; Stephanie Melka^1^; Nathan S. Fox^1^

^1^Icahn School of Medicine, Mount Sinai West, New York, NY; ^2^Icahn School of Medicine, Mount Sinai West, Mount Sinai West, NY

**Objective**: A uterine dehiscence or window refers to the clinically occult separation of a uterine scar. This study's objective was to determine risk factors for windows and the likelihood of windows based on number of prior cesareans.

**Study Design**: This was a retrospective study of patients who underwent a planned Cesarean in a single, large, academic, MFM practice from 2005 to 2025. We excluded patients who attempted labor or trial of labor after Cesarean. We compared patients with and without a uterine window at delivery. Characteristics of interest include total prior Cesareans, prior window or rupture, and whether labor occurred prior to scheduled delivery. We also evaluated the likelihood of a window based on number of total prior cesareans. Bivariate analysis was performed using T‐tests, Mann Whitney‐U tests and chi‐square tests.

**Results**: A total of 3295 patients were included‐ 216 (6.6 %) patients had windows, 3079 (93.4%) did not. Clinical features associated with windows are in Table 1. Unintended labor prior to cesarean was not associated with finding a window. On regression analysis, factors associated with a window were having three or more prior Cesareans (aOR 3.39, 95% CI 2.44–4.7) and a history of window or rupture (aOR 5.38, 95% CI 3.8–7.63). The frequency of dehiscence increased with each number of Cesarean (Table 2). Amongst patients without a prior window or rupture, the likelihood of finding a window was 0.3% at the first cesarean, 2.7% at second cesarean, 8.7% at third cesarean, 15.3% at fourth cesarean, 9.5% at fifth cesarean, and 19.1% at the sixth or higher cesarean (*p* < 0.001).

**Conclusion**: There is an approximately 1‐in‐10 risk of uterine window in patients with three or more prior Cesareans. This risk increases further with the number of prior Cesareans. Because these patients are at high risk for dehiscence, even if delivered before the onset of labor or had no prior history of dehiscence, delivery should be considered in the early term period (i.e., before ACOG's recommendation for elective delivery at 39 weeks).

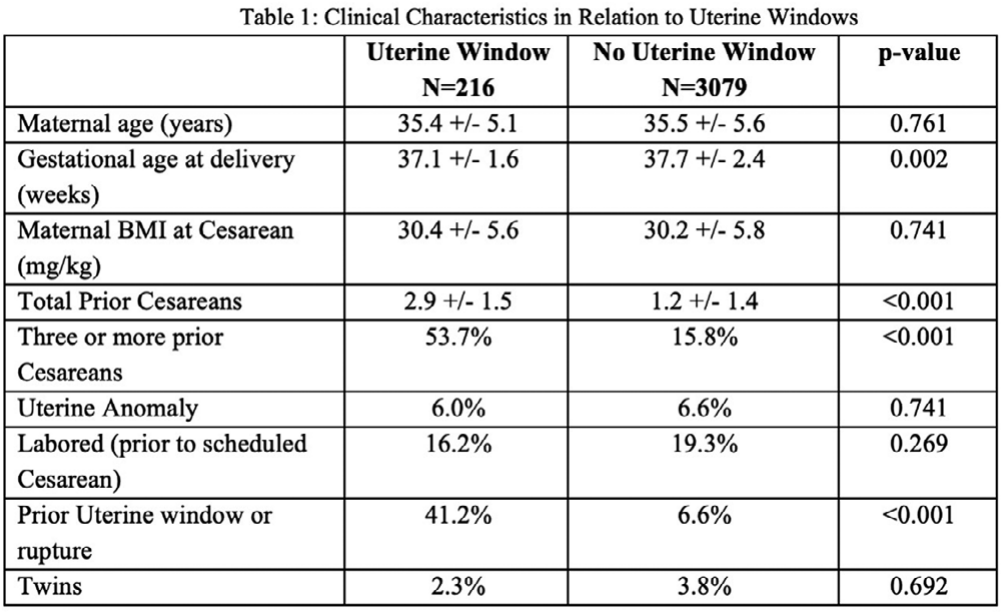

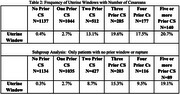

**10:30 AM – 12:00 PM**

## 395 | Four Critical Weeks: GDM Screening at 24 vs. 28 Weeks in Singleton and Twin Pregnancies

Yaniv Zipori^1^; Jacob Segal^2^; Guy Melamed^3^; Sivan Gazit^3^; Tal Patalon^3^; Anat Reiner‐Benaim^4^; Ron Beloosesky^5^

^1^Rambam Health Care Campus, Rambam Health Care Campus, HaZafon; ^2^Maccabi Health Care services, Tel Aviv, Israel, Maccabi Health Care services, Tel Aviv, Israel, HaMerkaz; ^3^Maccabi Health Care services, Maccabi Health Care services, HaMerkaz; ^4^Ben‐Gurion University of the Negev, Ben‐Gurion University of the Negev, HaDarom; ^5^Rambam Health Care Campus, Rambam Health Care Campus, Hefa

**Objective**: Regardless of plurality, the glucose challenge test (GCT) and oral glucose tolerance test (OGTT) are recommended at 24–28 weeks for the diagnosis of gestational diabetes mellitus (GDM). However, there is no consensus on the optimal timing within this four‐week window to optimize GDM detection rates. We assessed gestational age‐specific GDM rates in singleton and twin pregnancies.

**Study Design**: This retrospective study included nulliparous women from Maccabi Healthcare Services (2012–2022). The primary outcome of our study was to compare the rate of GDM diagnosis by OGTT timing (24–28 weeks) between singleton and twin pregnancies.

**Results**: Among 84,620 pregnancies (82,786 singletons; 1834 twins), twins had higher maternal age (30.5 ± 5.4 vs. 28.2 ± 5.0 years, *p* < 0.0001) and BMI (24.7 ± 5.3 vs. 23.8 ± 4.8, *p* < 0.0003). GDM was diagnosed in 4.9% of singleton pregnancies and 9.6% of twin pregnancies (*p* < 0.001), with comparable rates of GDMA1 and GDMA2 in both groups. Figure 1 shows that twin pregnancies also exhibited higher GCT levels than singleton pregnancies (126 ± 30 vs. 112 ± 29.0 mg/dL, *p* < 0.0001). This difference remained significant across all weeks within the 24–28‐week testing time frame. Figure 2 illustrates the rates of GDM at the lower and upper limits of the 24–28‐week gestational testing period. In singleton pregnancies, the diagnosis rate of GDM was 20% higher at 28 weeks compared to 24 weeks (5.0% vs. 4.2%, *p* = 0.004), whereas GDMA2 was more prevalent at 24 weeks than at 28 weeks (16.9% vs. 12.6%, *p* = 0.05). In twins, neither the rate of GDM diagnosis (6.9% vs. 8.6%, *p* = 0.70) nor that of GDMA2 (14.3% vs. 11.8%, *p* = 0.99) differed significantly between the two specified gestational ages.

**Conclusion**: Twins had higher GCT levels and double the GDM rate of singletons. Singletons exhibited gestational age variability (higher GDM at 28 weeks, GDMA2 at 24 weeks), while twins showed no such pattern. We propose: (1) GCT at 24 weeks for singleton pregnancies when the GDMA2 risk peaks; and (2) maintaining flexibility for twin pregnancies between 24–28 weeks.

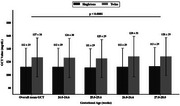

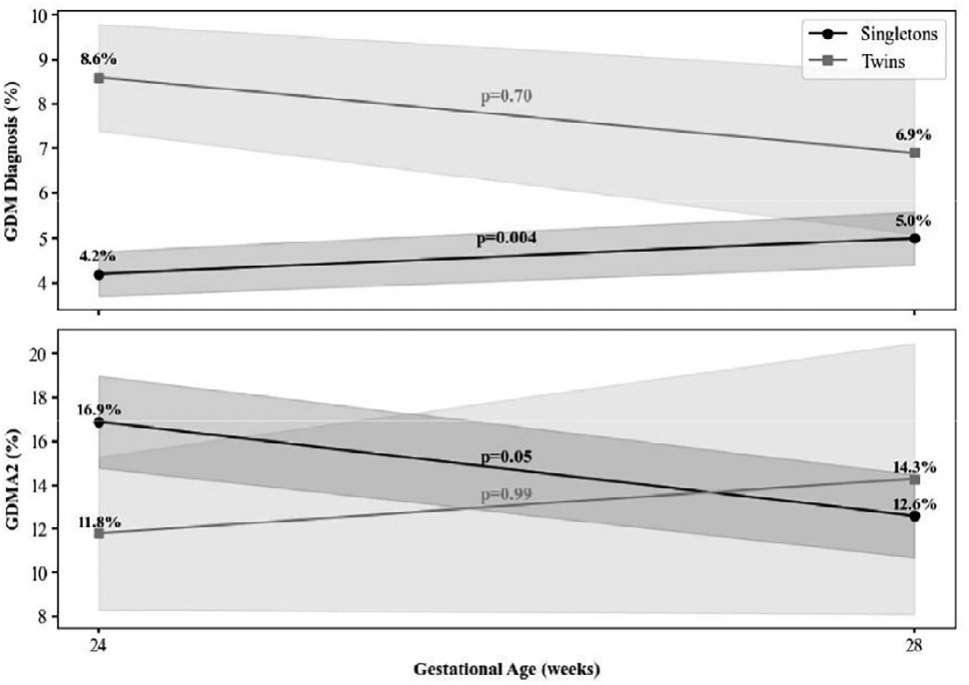

**10:30 AM – 12:00 PM**

## 396 | Performance of AI for Automatic Detection and Localization of Fetal Ultrasound Views and Structures

Clementine Morisset^1^; Celia Amabile^1^; Frederic Loge^1^; Julie Signeux^1^; Yinka Oyelese^2^

^1^Sonio, Paris, Ile‐de‐France; ^2^Beth Israel Deaconess Medical Center, Boston, MA

**Objective**: Quality control in fetal ultrasound requires both comprehensive documentation of standard planes and verification that expected anatomical structures and features are visible. This study assessed the performance of an AI software (not FDA cleared) for quality control of routine fetal imaging by ultrasound, through automatic detection and localisation of:

• 20 first‐trimester (T1) and 41 second/third‐trimester (T2/T3) standard planes, covering the full fetal anatomy;

• 76 anatomical structures grouped by region: brain (11), thorax & heart (40), placenta (4), sagittal face (12), coronal face (6), and spine (3); and automatic detection of: placental location and fetal sex.

**Study Design**: 34,001 US images were retrospectively collected from 69 centers in the USA, Europe, and Africa. 18 expert sonographers established the ground truth for views and structures. Sensitivity and specificity were assessed by comparing the AI software outputs to the ground truth. Subgroup analyses evaluated performance across ultrasound machine manufacturers, geographical regions, maternal age, gestational age, BMI, and race/ethnicity.

**Results**: The sensitivity of AI for view detection was 0.896 (95% CI: 0.890–0.902) at T1 and 0.906 (95% CI: 0.902–0.909) at T2/T3. Performance for view localisation, anatomical structure detection and localisation and characteristic detection is reported in Table 1. Robust performance of AI was consistently observed across all predefined subgroups.

**Conclusion**: This study demonstrates that AI can effectively support quality assurance in fetal ultrasound by automatically verifying the presence and documentation of standard views and key anatomical structures. By reducing repetitive manual tasks and promoting completeness of examinations, AI software has the potential to enhance both imaging quality and clinical workflow efficiency.

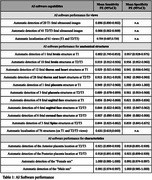

**10:30 AM – 12:00 PM**

## 397 | Impact of Parity on Perinatal Outcomes Following Shoulder Dystocia

Yossi Bart; Hector Mendez‐Figueroa; Sean C. Blackwell; Baha M. Sibai; Michal Fishel Bartal

Division of Maternal‐Fetal Medicine, Department of Obstetrics, Gynecology and Reproductive Sciences, McGovern Medical School at UTHealth Houston, Houston, TX

**Objective**: Shoulder dystocia (SD) remains an obstetric emergency with potential for significant neonatal and maternal harm. While fetal size and delivery mode are often emphasized, the role of parity is less understood. This study aimed to evaluate perinatal outcomes following SD, comparing nulliparas and multiparas.

**Study Design**: A secondary analysis of the Assessment of Perinatal Excellence (APEX) database, including all singleton deliveries ≥ 37 weeks complicated by SD. Individuals with fetal malformations or fetal demise were excluded. The primary outcome was a composite neonatal adverse outcome (CNAO), including cardiorespiratory resuscitation within 24 hours, birth‐related neonatal injury, and neurological outcomes. The secondary outcome was a composite of maternal adverse outcomes (CMAO), including 3rd or 4th degree perineal tear, cervical tear, estimated blood loss ≥ 1000 cc, and blood transfusion. Poisson regression was used to calculate adjusted relative risk (aRR) with 95% confidence intervals (CI).

**Results**: Of the 115,502 patients in the parent study, 2091 (2%) met inclusion criteria: 753 (36%) were nulliparous, while 1338 (64%) were multiparous. Nulliparas were younger, had lower rates of gestational diabetes mellitus (GDM) and neonatal macrosomia, as well as higher rates of operative delivery and later gestational age at delivery. CNAO rates were higher among nulliparas (aRR 1.49, 95% CI 1.20–1.84), including a higher rate of brachial plexus injury (aRR 2.60, 95% CI 1.52–4.46; Table 1). Lower Apgar scores and higher rates of neonatal intensive care unit admission were noted as well. CMAO rates was higher among nulliparas (aRR 2.40, 95% CI 1.86–3.10), driven mainly by higher rates of 3rd or 4th degree tear (Table 2).

**Conclusion**: Among term singletons with SD, nulliparity was associated with higher rates of adverse neonatal and maternal outcomes, including over twice the rate of brachial plexus injury as well as a 3rd/4th degree tear in 1 of 6 cases. These findings underscore the importance of individualized risk assessment and may support enhanced preparedness during delivery for nulliparas.